# Facial Selectivity in Mechanical Bond Formation: Axially Chiral Enantiomers and Geometric Isomers from a Simple Prochiral Macrocycle

**DOI:** 10.1021/jacs.3c14329

**Published:** 2024-03-20

**Authors:** Peter R. Gallagher, Andrea Savoini, Abed Saady, John R. J. Maynard, Patrick W. V. Butler, Graham J. Tizzard, Stephen M. Goldup

**Affiliations:** † Chemistry, University of Southampton, University Road, Southampton, SO17 1BJ, U.K.; ‡ School of Chemistry, University of Birmingham, Edgbaston, Birmingham B15 2TT, U.K.

## Abstract

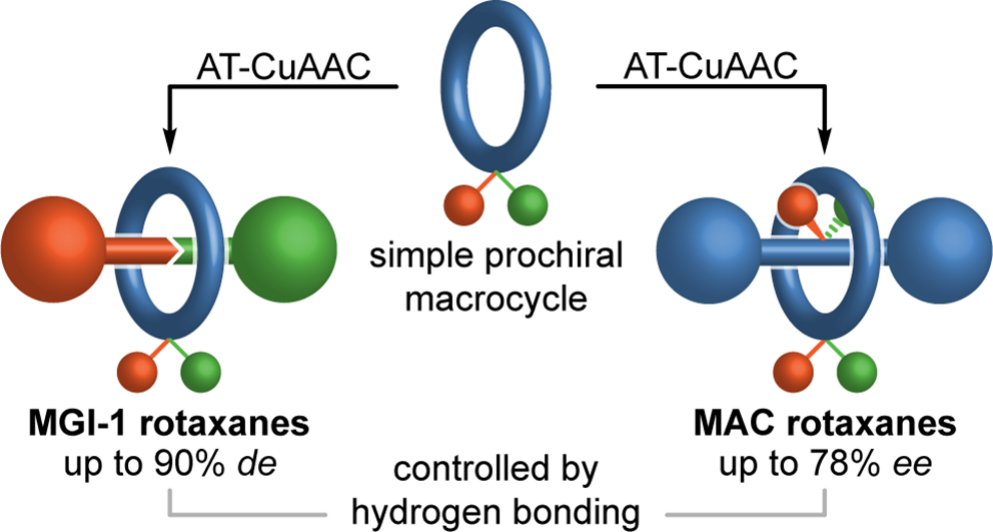

In 1971, Schill recognized that a prochiral macrocycle encircling an oriented axle led to geometric isomerism in rotaxanes. More recently, we identified an overlooked chiral stereogenic unit in rotaxanes that arises when a prochiral macrocycle encircles a prochiral axle. Here, we show that both stereogenic units can be accessed using equivalent strategies, with a single weak stereodifferentiating interaction sufficient for moderate to excellent stereoselectivity. Using this understanding, we demonstrated the first direct enantioselective (70% *ee*) synthesis of a mechanically axially chiral rotaxane.

## Introduction

Early in the development of the chemistry of the mechanical bond,^1^ Schill recognized that when a macrocycle containing a prochiral center such that its faces are distinguishable encircles an axle with distinguishable ends, the rotaxane can exist as distinct geometric isomers even though the individual components are stereochemically trivial.^2^ Although molecules that correspond to the type 1^3^ mechanical geometric isomers (MGI-1) of rotaxanes have been reported, the vast majority where the mechanical bond provides the sole stereogenic unit^4^ are constructed from calixarenes^5^ or similar macrocycles^6^ whose facial dissymmetry arises from the fixed cone-shaped conformation of the threaded ring.^7^ The same is true of the corresponding catenane stereogenic unit first reported by Gaeta and Neri.^8^ In these cases, facial dissymmetry is expressed over the whole macrocycle, which has been shown to lead to the stereoselective formation of the corresponding rotaxanes. However, to our knowledge, the only MGI-1 rotaxanes in which a single covalent prochiral center differentiates the faces of the ring,^9^ as envisaged by Schill, were reported by Bode and Saito,^10^ where no stereoselectivity was reported.

More recently,^11^ we identified that when a facially dissymmetric macrocycle encircles a prochiral axle, an overlooked mechanically axially chiral (MAC)^12^ stereogenic unit arises that is analogous to the MAC stereogenic unit of catenanes identified by Wasserman and Frisch over 60 years earlier.^13^ Having made this observation, we demonstrated that such molecules can be synthesized using a diastereoselective co-conformational chiral auxiliary^14^ active template^15^ Cu-mediated alkyne–azide cycloaddition (AT-CuAAC)^16,17^ approach with a ring whose facial dissymmetry arises from a single prochiral sulfoxide unit.

**Figure 1:**
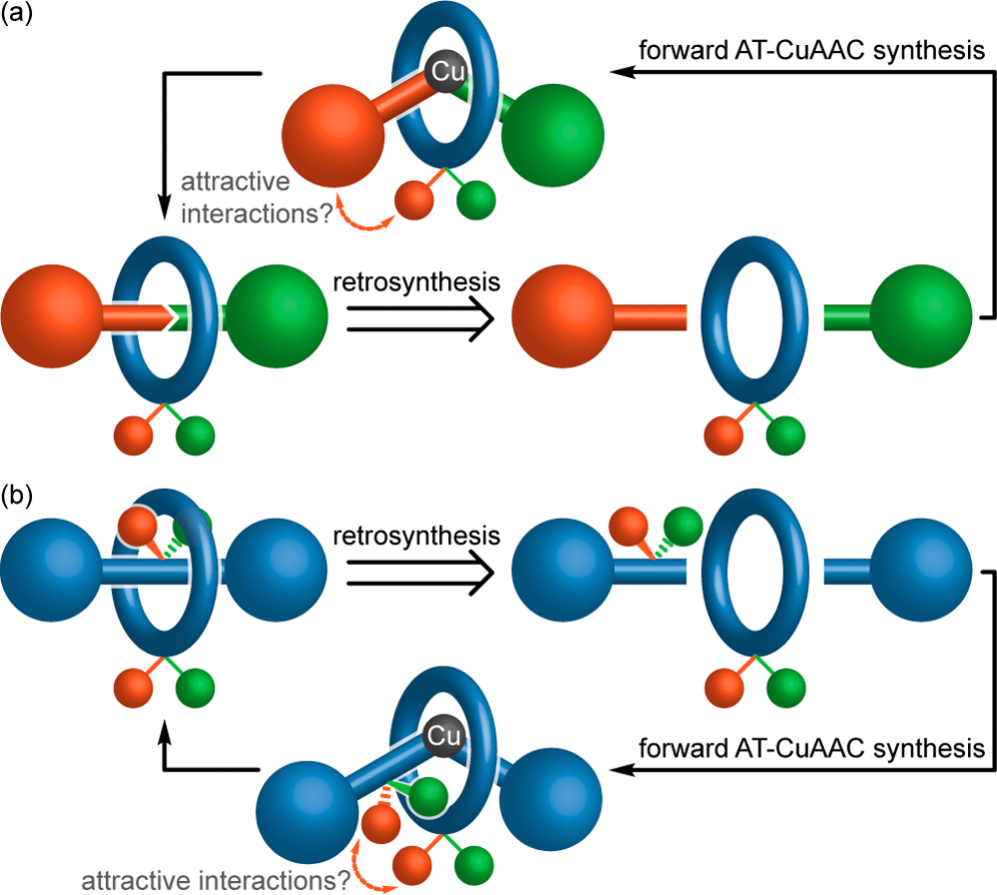
Schematic active template retrosyntheses of the mechanical (a) type 1 geometric isomers and (b) axially chiral enantiomers of rotaxanes, highlighting the need to control facial selectivity in the mechanical bond-forming step and the potential for attractive interactions between one face of the macrocycle and one of the half-axles to provide this control.

If we consider a schematic AT-CuAAC retrosynthesis of MGI-1 isomers (Figure 1a) and MAC enantiomers (Figure 1b), in which the axle is divided into two components that couple through the macrocycle in the forward synthesis, the common challenge involved in the stereoselective synthesis of both becomes obvious; we must control which face of the macrocycle is oriented toward which half-axle component in the mechanical bond-forming step.

Here, by re-examining our stereoselective synthesis of MAC rotaxanes, we identify that a single H-bond between the sulfoxide unit and one of the two half-axle components appears to play a key role in the reaction outcome. We use this understanding to develop a stereoselective approach to rotaxane MGI-1 isomers that can be extended directly to their catenane counterparts. Finally, we apply these principles to the direct synthesis of MAC rotaxanes without the need to produce diastereomeric intermediates.

## Results and Discussion

### Effect of the Conditions and Substrate Structure in the Synthesis of MAC Rotaxanes **4**

**Scheme 1:**
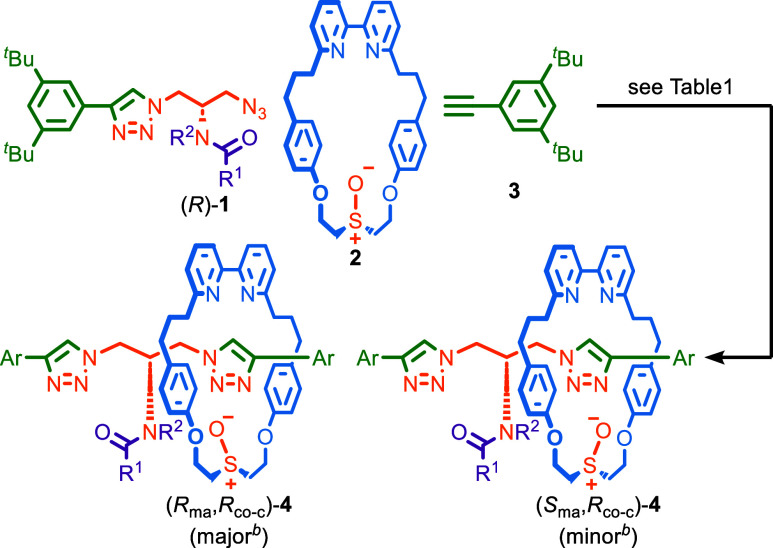
Synthesis of Rotaxanes **4** Reagents and conditions (see also Table 1): (*R*)-**1** (1.1 equiv), **2** (1 equiv), **3** (1.1 equiv), [Cu(CH_3_CN)_4_]PF_6_ (0.96 equiv), ^*i*^Pr_2_NEt (2 equiv). Determined by SCXRD for **1a**(11) and **1d** (Figure 1); **1b**, **c**, and **e** are presumed. Ar = 3,5-di-^*t*^Bu-C_6_H_3_.

Previously,^11^ we found that the AT-CuAAC reaction of azide (*R*)-**1a**, macrocycle **2**, and alkyne **3** gave rotaxane diastereomers (*R*_ma_,*R*_co–c_)^18^-**4a** (major) and (*S*_ma_,*R*_co–c_)-**4a** (minor) in 50% *de* (Scheme 1 and Table 1, entry 1). These products have the same co-conformational covalent configuration^19^ (set by the configuration of **1a**) but opposite mechanical axial configuration. They are separable because the steric bulk of the NHBoc group prevents the epimerization of the covalent stereocenter by shuttling of the macrocycle between triazole-containing compartments. The solid-state structure obtained by single-crystal X-ray diffraction (SCXRD) of an analogous catenane^11^ contained a close contact between the polarized NH of the carbamate unit and the O atom of the sulfoxide unit, which suggested that an H-bond between these groups may play a role in the observed stereoselectivity.^20^

**Table 1:** Effect of the Reaction Conditions and Substrate on the AT-CuAAC Diastereoselective Synthesis of Rotaxanes **4**

entry	substrate	conditions	selectivity^a^
1^11^	**1a** (R^1^ = O^*t*^Bu, R^2^ = H)	CH_2_Cl_2_, rt	50% *de*
2	**1a** (R^1^ = O^*t*^Bu, R^2^ = H)	EtOH, rt	14% *de*
3	**1b** (R^1^ = Me, R^2^ = H)	CH_2_Cl_2_, rt	36% *de*
4	**1c** (R^1^ = CCl_3_, R^2^ = H)	CH_2_Cl_2_, rt	48% *de*
5	**1d** (R^1^ = CF_3_, R^2^ = H)	CH_2_Cl_2_, rt	70% *de*
6	**1d** (R^1^ = CF_3_, R^2^ = H)	EtOH, rt	16% *de*
7	**1e** (R^1^ = CF_3_, R^2^ = Me)	CH_2_Cl_2_, rt	10% *de*
8	**1a** (R^1^ = O^*t*^Bu, R^2^ = H)	CH_2_Cl_2_, –40 °C	72% *de*
9	**1a** (R^1^ = O^*t*^Bu, R^2^ = H)	CH_2_Cl_2_, –78 °C	80% *de*
10	**1d** (R^1^ = CF_3_, R^2^ = H)	CH_2_Cl_2_, –40 °C	82% *de*
11	**1d** (R^1^ = CF_3_, R^2^ = H)	CH_2_Cl_2_, –78 °C	70% *de*

a Determined by ^1^H NMR analysis of the crude reaction product.

**Figure 2:**
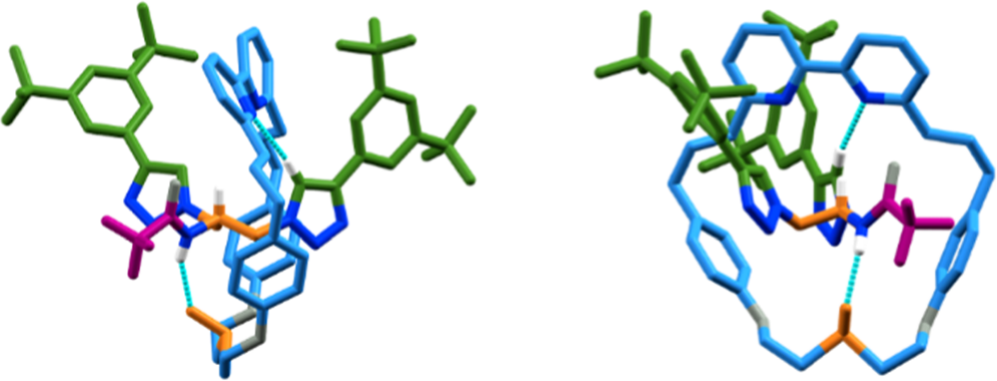
SCXRD structure of [(*R*_ma_,*R*_co–c_)-**4d** (major isomer), with key intercomponent interactions highlighted. Colors as in Scheme 1, including the sulfoxide (SO) moiety to highlight the differentiation of the macrocycle faces, except N [dark blue], O [gray], and H [white]. The majority of H was omitted.

To test this proposal, we first compared the outcome of reactions performed in CH_2_Cl_2_ and EtOH, the latter being a more competitive H-bonding solvent, and found that the stereoselectivity was indeed reduced to 14% *de* (entry 2). Furthermore, the reactions of azides **1b**–**d** to give rotaxanes **4b**–**d** (entries 3–5) proceeded with selectivities that paralleled the polarization of the N–H unit; trifluoroacetamide **1d** produced rotaxane **4d** in the highest selectivity (70% *de*), followed by trichloroacetamide **1c** (48% *de*) then acetamide **1b** (36% *de*). The SCXRD structure of the major isomer of **4d** (Figure 2) revealed the same (*R*_ma_,*R*_co–c_) configuration as that of **4a**, with an NH···O H-bond observed between the amide NH and sulfoxide units. Methylated trifluoroacetamide rotaxane **4e** was produced in 10% *de* (entry 6), which, although consistent with the key role of the NH···O H-bond, suggests that there is some inherent facial bias between the azide and alkyne half-axles in the AT-CuAAC reactions of **2**.

**Scheme 2:**
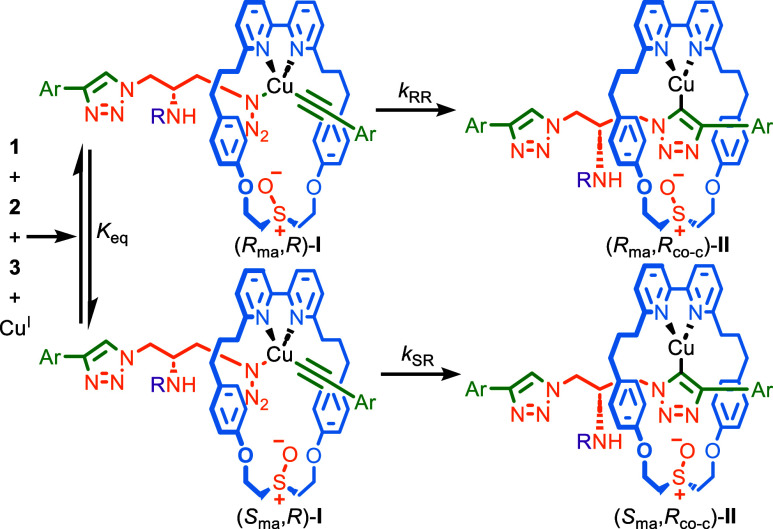
Proposed AT-CuAAC Mechanism Highlighting Pre-Equilibrium and Kinetic Resolution Steps

The effect of the temperature on the stereoselectivity of the reactions of **1a** and **1d** was more complicated. Whereas reducing the reaction temperature in the synthesis of **4a** from rt (entry 1) to −40 °C (entry 8) and −78 °C (entry 9) increased the observed selectivity, that for **4d** was higher at −40 °C (entry 10) and then fell at −78 °C (entry 11). We suggest that this slightly counterintuitive observation can be rationalized in broad terms by considering that the AT-CuAAC reaction takes place over several steps,^21^ which include an equilibrium between diastereomeric azide/acetylide complexes **I**, followed by irreversible formation of the corresponding triazolides **II** (Scheme 2).^22^ The observed stereoselectivity is thus a composite function of the pre-equilibrium step (*K*_eq_) and the relative rates (*k*_RR_, *k*_SR_) at which intermediates **I** progress to triazolides **II**. The effect of temperature on the reaction to produce **4d** suggests the pre-equilibrium and kinetic resolution steps respond differently to changes in temperature, resulting in the observed behavior.^23^

### Stereoselective Synthesis of MGI-1 Rotaxanes

Having demonstrated that a single H-bond between the sulfoxide unit and one of the incoming half-axle components appears to be important in the synthesis of rotaxanes **4**, we turned our attention to the synthesis of analogous rotaxanes expressing the MGI-1 stereogenic unit.

**Scheme 3:**
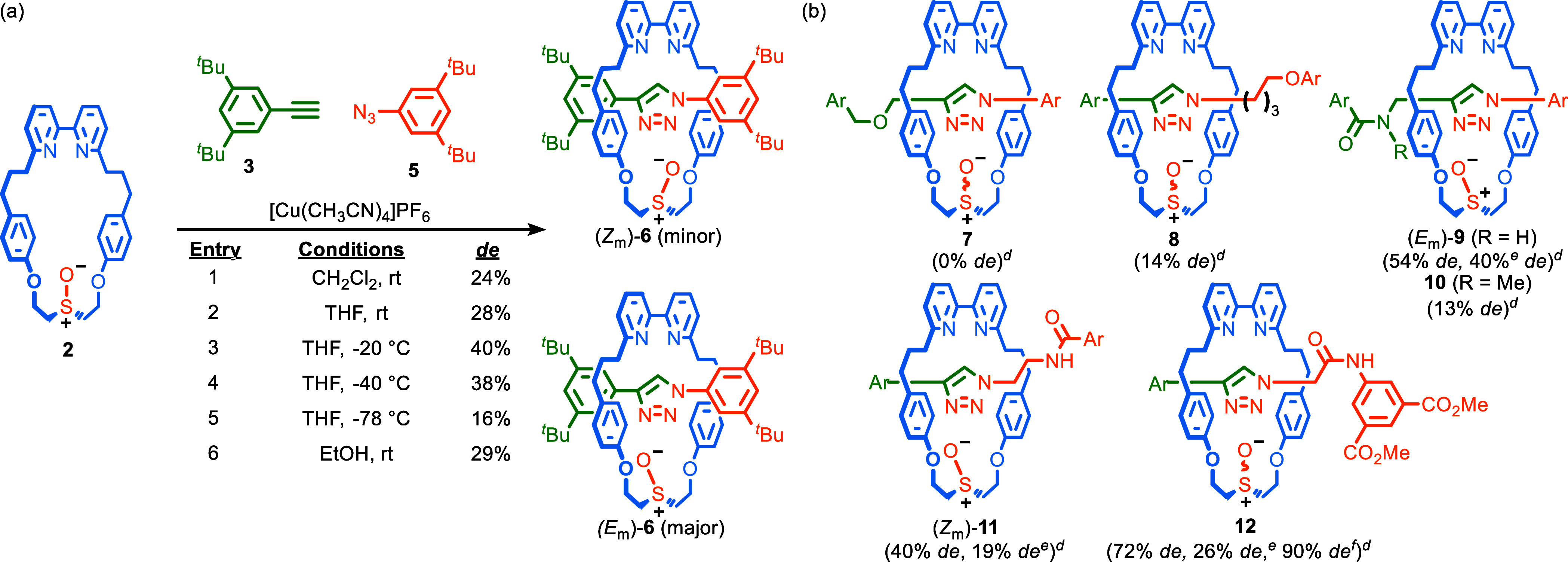
AT-CuAAC Synthesis of Rotaxane Geometric Isomers of Type 1. (a) Effect of Conditions on the Formation of Rotaxanes **6**. (b) Effect of the Half-Axle Structure, on the Stereoselectivity of Mechanical Bond Formation with Macrocycle 2, Reagents and conditions: 2 (1 equiv), 3 (1.1 equiv), 5 (1.1 equiv), [Cu(CH_3_CN)_4_]PF_6_ (0.96 equiv), *^i^*Pr_2_EtN (2 equiv). Synthesized in THF at rt (Scheme 3a, entry 2) unless otherwise stated. Stereochemistry of the major isomer indicated where determined. Determined by ^1^H NMR analysis of the crude reaction product. Synthesized in EtOH. Synthesized at –40 °C in THF. Ar = 3,5-di-*^t^*Bu-C_6_H_3_.

**Figure 3:**
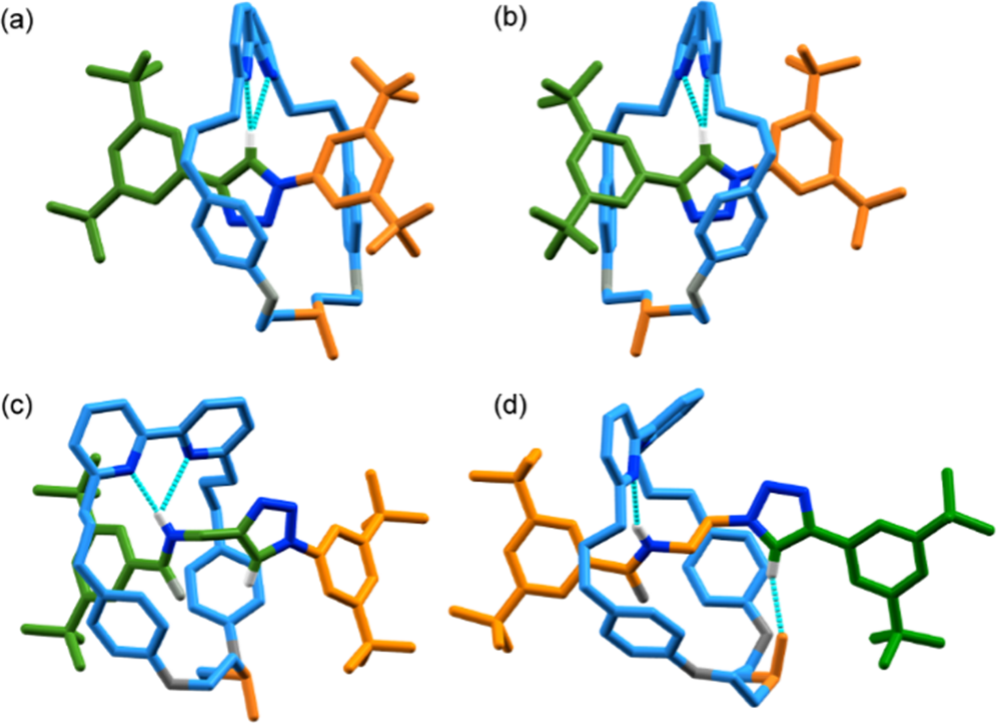
(a) Solid-state structures of (a) (*Z*_m_)-**6**, (b) (*E*_m_)-**6**, (c) (*Z*_m_)-**9**, and (d) (*E*_m_)-**11** with key intercomponent interactions highlighted. Colors as in Scheme 1, including the sulfoxide (SO) moiety to emphasize the macrocycle faces, except for O (gray), N (dark blue), and H (white). The majority of H was omitted for clarity.

Intrigued by the small but measurable stereoselectivity observed in the formation of **4e**, which cannot arise due to the proposed stereodifferentiating NH···O interaction, we examined the AT-CuAAC coupling between macrocycle **2**, and half-axles **3** and **5**, neither of which contain a directing group. At rt in CH_2_Cl_2_ (Scheme 3a, entry 1), geometric isomers (*E*_m_)-**6** and (*Z*_m_)-**6** were formed in low but significant stereoselectivity (24% *de*), confirming that the AT-CuAAC reactions of **2** are not only biased by the H-bond identified in the case of rotaxanes **4**.^24^ Analysis of the separated isomers of **6** by SCXRD allowed their absolute stereochemistry to be determined (Figure 3a,b). Replacing the solvent with THF marginally improved the selectivity (28% *de*, entry 2), as did lowering the reaction temperature to −20 °C (40% *de*, entry 3), but, as with **4d**, reduced selectivity was observed at lower temperatures (entries 4 and 5). Using EtOH as a solvent was comparable to THF (entry 6).^25^

Although the selectivities observed in the formation of **4e** and **6** are consistent with some inherent facial bias between the azide and alkyne half-axles in the mechanical bond-forming step, when a propargylic alkyne was employed with aryl azide **5** to generate rotaxane **7**, no stereoselectivity was observed (Scheme 3b). In contrast, the reaction of an alkyl azide and aryl acetylene **3** to give rotaxane **8** proceeded in an appreciable stereoselectivity (14% *de*). Thus, although it is clearly possible to achieve low selectivities in the AT-CuAAC reactions of **2** in the absence of obvious directing interactions, this is highly substrate-dependent, and its origins are unclear at this time.^26^

Returning to our H-bonding-directed approach, when a propargylic amide was reacted with **2** to give **9**, significantly improved stereoselectivity (54% *de*) was obtained, which was reduced in EtOH (40% *de*). The corresponding *N*-methyl amide gave rise to rotaxane **10** in low selectivity (13% *de*). The AT-CuAAC coupling of **3** and an alkyl azide bearing a simple amide gave rotaxane **11** in moderate stereoselectivity (40% *de*), which was reduced in EtOH (19% *de*). Thus, the amide can be placed in either coupling partner. Finally, rotaxane **12**, whose amide NH is expected to be more polarized than that of **11**, was produced in good selectivity (72% *de*) at rt, which was improved (90% *de*) when the same reaction was conducted at −40 °C. Reducing the temperature further did not improve the observed stereocontrol and led to a slow reaction. Replacing the reaction solvent with EtOH once again led to reduced selectivity (26% *de*).

As in the case of rotaxanes **4**, the high selectivity observed in the synthesis of **9**, **11**, and **12** is consistent with the key role of an NH···O interaction between the macrocycle and half-axle in controlling the facial selectivity in the AT-CuAAC reactions of macrocycle **2**. However, we previously observed^11^ this interaction in the solid-state structures of both diastereomers of epimeric MAC catenanes even though, in principle, in one diastereomer, the S–O bond could be expected to project away from the NH unit, which is possible due to the flexible nature of macrocycle **2**. The major isomers of rotaxanes **9** and **11** determined by SCXRD (Figure 3c,d, respectively) highlight the importance of this flexibility; although both were formed selectively, counterintuitively, the ring is oriented in opposite directions with respect to the amide in the major diastereomer of each. Thus, although the NH···O interaction appears able to direct the synthesis of MGI-1 isomers, the major product depends on the detailed structure of the half-axles used.^27^ We also note that whereas an NH···O interaction is observed in the SCXRD structure of **4d**, in the case of **9** and **11**, this is replaced by an NH···N interaction between the amide proton and one of the bipyridine N atoms, with the SO unit instead interacting with the polarized C–H of the triazole moiety in an inter- or intramolecular manner, respectively, presumably because the NH unit is geometrically accessible to the macrocycle in rotaxanes **9** and **11** whereas it is not in the case of **4d**.

### Stereoselective Synthesis of an MGI Catenane

**Scheme 4:**
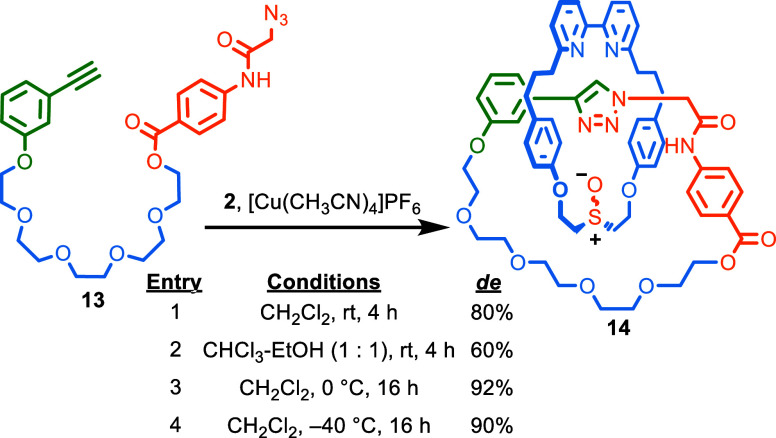
Stereoselective Synthesis of Catenane **14** Reagents and conditions: **13** (2 equiv) was added over the time stated using a syringe pump to **2** (1 equiv), [Cu(CH_3_CN)_4_]PF_6_ (0.97 equiv), ^*i*^Pr_2_EtN (4 equiv).

Having established that a polarized NH unit appears sufficient to control the synthesis of MGI-1 rotaxanes with macrocycle **2**, we briefly investigated whether the same approach could be applied to the related isomers of catenanes. Pre-macrocycle **13**, which contains an activated amide unit analogous to that of **12**, reacted with **2** under our AT-CuAAC catenane-forming conditions (Scheme 4)^28^ to give **14** with good stereocontrol (80% *de*, entry 1). The same reaction in CHCl_3_-EtOH gave reduced selectivity (60% *de*, entry 2), whereas performing the reaction at 0 °C in CH_2_Cl_2_ increased the selectivity (92% *de*, entry 3). Lowering the temperature further (−40 °C) had no significant effect (90% *de*, entry 4). Thus, unsurprisingly, given the similarity of their stereogenic units, MGI-1 rotaxanes and MGI catenanes can be made with good stereocontrol using equivalent strategies.

### Direct Enantioselective Synthesis of MAC Rotaxanes

**Scheme 5:**
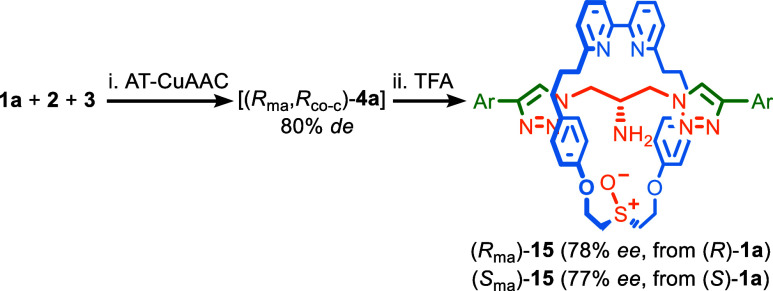
Two-Step, One-Pot Synthesis of Enantioenriched MAC Rotaxanes **15**, Reagents and conditions: i. **1a** (1.1 equiv), **2** (1 equiv), **3** (1.1 equiv), [Cu(CH_3_CN)]PF_6_ (0.96 equiv), ^*i*^Pr_2_EtN (2 equiv), CH_2_Cl_2_, 16 h; ii. TFA, CH_2_Cl_2_, –78 °C to rt, 6 h. Determined by analytical CSP-HPLC. Ar = 3,5-di-^*t*^Bu-C_6_H_3_.

Finally, we returned to apply our findings to the stereoselective synthesis of the enantiomers of MAC rotaxanes. In our original report,^11^ we separated the diastereomers of epimeric rotaxanes **4a** before removing the Boc group to generate rotaxane **15** (Scheme 5), in which the MAC stereogenic unit is the only fixed source of stereochemistry. This was necessary as the AT-CuAAC reaction only proceeded in 50% *de*; the ultimate purpose of developing methodologies to produce stereochemically complex mechanically interlocked molecules is so that they can then be investigated in applications such as sensing^29^ or catalysis,^30^ for which they must be of high stereopurity.

Trivially, our optimized conditions for the diastereoselective formation of **4a** (Table 1, entry 9) removes the need for the separation of the MAC epimers and so allows the synthesis of highly enantioenriched samples of rotaxane **15** in a two-step, one-pot manner (Scheme 5); AT-CuAAC coupling of (*R*)-**1a** followed by TFA-mediated removal of the Boc group gave rotaxane (*R*_ma_)-**15** in good stereoselectivity (78% *ee*) in agreement with that observed for **4a** (80% *de*). The same reaction with (*S*)-**1a** gave (*S*_ma_)-**5** (77% *ee*).

**Figure 4:**
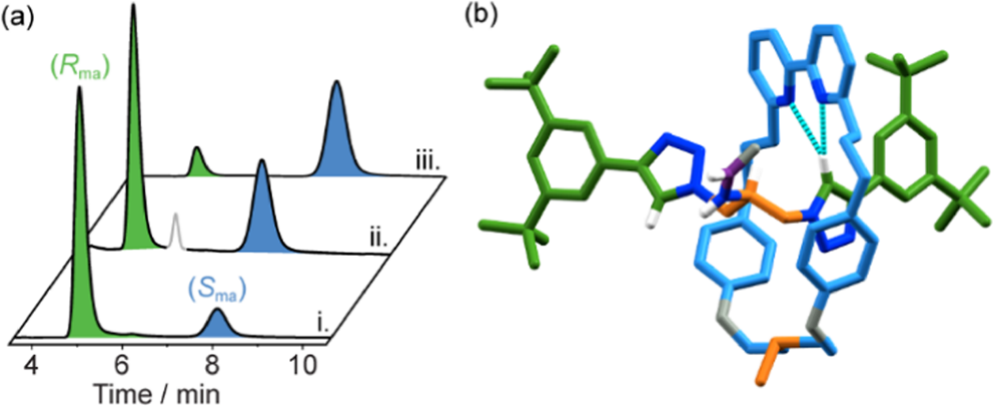
(a) CSP-HPLC analysis of i. (*R*_ma_)-**16** (67% *ee*) produced from (*R*)-**1g**; ii. (*R*_ma_)-**16** (21% *ee*) produced from (*R*_ma_)-**15** (21% ee; minor impurity highlighted in gray), and iii. (*S*_ma_)-**16** (70% *ee*) produced from (*S*)-**1g**. (b) Solid-state structure of *rac*-**16**, in which the N–H···O bond between the SO unit and the amide is intermolecular (colors as in Scheme 6, including the sulfoxide (SO) moiety to highlight the differentiation of the macrocycle faces, except N [dark blue], O [gray], and H [white]). The majority of H was omitted for clarity.

**Scheme 6:**
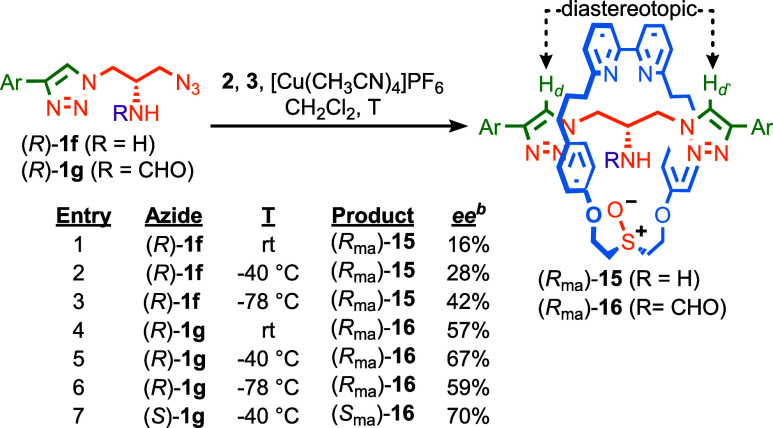
Direct Synthesis of Enantioenriched Mechanically Axially Chiral Rotaxanes **15** and **16** Reagents and conditions: i. **1** (1.1 equiv), **2** (1 equiv), **3** (1.1 equiv), [Cu(CH_3_CN)]PF_6_ (0.96 equiv), ^*i*^Pr_2_EtN (2 equiv), CH_2_Cl_2_, 16 h. Determined by analytical CSP-HPLC. Ar = 3,5-di-^*t*^Bu-C_6_H_3_.

More excitingly, the high stereoselectivity observed in the AT-CuAAC reaction of azides **1** bearing a polarized NH presents the opportunity for the direct synthesis of MAC rotaxanes without the need for first forming separable co-conformational diastereomers; if the N substituent is too small to trap the macrocycle in one triazole-containing compartment, the only fixed stereochemistry in the product is provided by the MAC stereogenic unit. Thus, the reaction of primary amine-containing azide (*R*)-**1e** with macrocycle **2** and alkyne **3** at rt gave MAC rotaxane **15** directly but in low stereoselectivity (16% *ee*, Scheme 6, entry 1), which increased when the reaction was performed at −40 °C (28% *ee*, entry 2) and improved further still at −78 °C (42% *ee*, entry 3). CSP-HPLC analysis of a sample of rotaxane (*R*_ma_)-**15** produced from (*R*)-**1a** (Scheme 5) and comparison with the same product from (*R*)-**1f** confirmed that the latter also produces (*R*_ma_)-**15** as the major product (Figure 4a).

When instead formamide-containing azide (*R*)-**1g** was reacted with **2** and **3**, even at rt rotaxane **16**(31) was obtained in reasonable stereopurity (57% *ee*, entry 3), which was improved further at −40 °C (67% *ee*, entry 4). Conducting this reaction at −78 °C reduced the observed stereoselectivity (59% *ee*, entry 5), suggesting that, as with azide **1d**, the pre-equilibrium and kinetic resolution steps result in an unusual temperature dependence. CSP-HPLC analysis of a sample of rotaxane **16** produced by formylation of a sample of rotaxane (*R*_ma_)-**15** of known stereopurity and comparison with the same compound produced from (*R*)-**1g** confirmed that the latter produces (*R*_ma_)-**16** as the major stereoisomer. When (*S*)-**1g** was reacted instead, (*S*_ma_)-**16** was produced (70% *ee*, entry 6). The solid-state structure of **16** obtained by SCXRD (Figure 4b) did not display the expected intermolecular NH···O H-bond; instead, the same interaction was found to occur in an intermolecular fashion within the unit cell.

The different co-conformational behaviors of **4a**, **15**, and **16** are clear from the analysis of their respective ^1^H NMR spectra. Diastereomers (*R*_ma_,*R*_co–c_)-**4a** and (*S*_ma_,*R*_co–c_)-**4a** are separable species; heating a mixture of diastereomers **4a** resulted in no change in their ratio (Figure S47), confirming that the macrocycle cannot shuttle between the two compartments due to the large NHBoc unit. In contrast, the diastereotopic triazole resonances H_*d*_^32^ of amine rotaxane **15** appear as two sharp singlets at 298 K, indicating that diastereomeric co-conformations (*R*_ma_,*R*_co–c_)-**15** and (*S*_ma_,*R*_co–c_)-**15** are in fast exchange on the ^1^H NMR timescale through rapid shuttling of the macrocycle between the two triazole-containing compartments (Figure S190). The same resonances for formamide rotaxane **16** are broad at 298 K, although once again, only two signals are observed (Figure S200). This observation is consistent with (*R*_ma_,*R*_co–c_)-**16** and (*S*_ma_,*R*_co–c_)-**16** exchanging on the ^1^H NMR timescale, albeit more slowly than (*R*_ma_,*R*_co–c_)-**15** and (*S*_ma_,*R*_co–c_)-**15**, in keeping with the larger steric bulk of the formamide group of **16**. Accordingly, increasing the temperature resulted in the sharpening of the two resonances corresponding to protons H_*d*_ (Figure S211).

## Conclusions

In conclusion, we have demonstrated that type 1 rotaxane mechanical geometric isomers and mechanically axially chiral enantiomers can be obtained by controlling facial selectivity in an AT-CuAAC synthesis. Specifically, we show that an H-bonding interaction between a prochiral macrocycle and a functional group contained in one of the two half-axles (rotaxane synthesis) or unsymmetrically disposed in the corresponding pre-macrocycle structure (catenane synthesis) appears to be sufficient to control the reaction outcome. Although the focus of our discussion has been on reaction stereoselectivities, it should be noted that, as is typically the case for AT-CuAAC reactions mediated by bipyridine macrocycles,^33^ all of the interlocked structures reported were obtained in good to excellent isolated yield (50–90%, see the SI for details). The high selectivity observed with optimized substrates allowed us to design a direct enantioselective synthesis of mechanically axially chiral rotaxanes, only the second^34a^ example of a direct stereoselective synthesis of a mechanically chiral molecule and the first of this recently identified stereogenic unit. To date, type 1 mechanical geometric isomers of rotaxanes based on calixarenes and similar cone-shaped macrocycles,^5,8b,6d,6e^ as well as structures expressing combinations of mechanical and covalent stereochemistry^4h^ have been investigated as components of molecular switches and motors. Here, we have demonstrated that such isomerism can be expressed and controlled in much simpler macrocycles, opening up new motifs for study. Similarly, mechanically planar chiral molecules, for which stereoselective methods are known,^14,26,34^ have been investigated as enantioselective sensors,^29^ catalysts,^30^ and chiroptical switches.^35^ With methodological concepts now in hand to efficiently synthesize their mechanically axially chiral cousins in high stereopurity, we eagerly anticipate the chemical applications to which molecules containing this stereogenic unit will soon be put.

## Data Availability

Characterization data for reported compounds is available from the University of Birmingham UBIRA eData repository at https://doi.org/10.25500/edata.bham.00001077.

## Supporting Information Available

Procedures and full characterization data (NMR, MS, CD, SCXRD, HPLC as appropriate) for all novel compounds and discussion (PDF)
