# Supplementary material for: Facial Selectivity in Mechanical Bond Formation: Axially Chiral Enantiomers and Geometric Isomers from a Simple Prochiral Macrocycle
Source: J Am Chem Soc. 2024 Mar 20;146(13):9134–41. doi: 10.1021/jacs.3c14329 (PMC10996000; doi:10.1021/jacs.3c14329)
Supplement: Supplementary file 1 — ja3c14329_si_001.pdf [file ja3c14329_si_001.pdf]

Electronic Supporting Information

Facial Selectivity in Mechanical Bond Formation: Axially Chiral Enantiomers and Geometric Isomers from a Simple Prochiral Macrocycle

Peter R. Gallagher,[1,‡] Andrea Savoini,[1,‡] Abed Saady,[1,2] John R. J. Maynard,[1] Patrick W. V. Butler,[1] Graham J. Tizzard,[1] and Stephen M. Goldup[1,2,*]

[1] Department of Chemistry, University of Southampton, Highfield, Southampton, SO17 1BJ, U.K.
[2] School of Chemistry, University of Birmingham, University Rd W, Birmingham B15 2TT, U.K.
[‡]These authors contributed equally
*s.m.goldup@bham.ac.uk

1. General Experimental Information **3**

2. Synthesis of sulfide macrocycle precursor S3 **5**

3. Rotaxanes 4a-e and their precursors (Scheme 1, main text) **6**

Boc rotaxanes 4a 32

Acetamide rotaxanes 4b 34

Trichloroacetamide rotaxanes 4c 39

Trifluoroacetamide rotaxanes 4d 44

Methylated trifluoroacetamide rotaxanes 4e 53

4. Synthesis of rotaxanes 6-12 and associated compounds (Scheme 3, main text) **60**

Rotaxanes (E_m)-6 and (Z_m)-6 60

Ether rotaxanes (E_m)-7 and (Z_m)-7 68

Phenolic Rotaxanes (E_m)-8 and (Z_m)-8 76

Amide Rotaxanes (E_m)-9 and (Z_m)-9 82

Methylated Amide Rotaxanes (E_m)-10 and (Z_m)-10 93

Amide Rotaxanes (E_m)-11 and (Z_m)-11 97

Aniline Rotaxanes (E_m)-12 and (Z_m)-12 104

5. Synthesis of catenane 14 and associated compounds (Scheme 4, main text) **110**

Macrocycle precursor 13 119

Catenanes (E_m)-14 and (Z_m)-14 122

6. Rotaxanes 15 and 16, and their precursors (Schemes 5 and Scheme 6, main text) **128**

Amine rotaxane (R_{ma})-15 from (*R*)-1a 128

Amine rotaxane (R_{ma})-15 from (*R*)-1f 130

Formamide rotaxane (R_{ma})-16 135

7. Determination of the major stereoisomer of rotaxane 16 produced from 1g **141**

Rotaxanes 16 *via* formylation of rotaxanes 15 141

8. Absolute stereochemical assignment of interlocked compounds **142**

8.1. A note on the assignment of the MAC stereogenic unit – the need for revision 142

8.2. Revised rules for the assignment of mechanically axially chiral catenanes 143

8.3. Revised rules for the assignment of mechanically axially chiral rotaxanes 145

8.4. Absolute stereochemical assignment of type I rotaxane mechanical geometric isomers 147

8.5. Absolute stereochemical assignment of catenane mechanical geometric isomers 148

9. Effect of temperature and solvent on the diastereoselective synthesis of rotaxanes 6 **151**

10. Effect of conditions on the enantioselectivity of the AT-CuAAC reaction (Scheme 6) **152**

10.1. Effect of temperature on the direct enantioselective synthesis of rotaxanes 15 152

10.2. Effect of temperature on the enantioselective synthesis of rotaxanes 16 154

11. Variable temperature NMR analysis of rotaxane 16 **156**

12. Single Crystal X-ray Diffraction Analysis **158**

12.1. Single Crystal X-ray Diffraction Data for rotaxane (R_{ma},$R_{co\text{-}c}$)-4d 158

12.2. Single Crystal X-ray Diffraction Data for rotaxane (Z_m)-6 160

12.3. Single Crystal X-ray Diffraction Data for rotaxane (E_m)-6 162

12.4. Single Crystal X-ray Diffraction Data for rotaxane (Z_m)-9 164

12.5. Single Crystal X-ray Diffraction Data for rotaxane (E_m)-11 166

12.6. Single Crystal X-ray Diffraction Data for rotaxane *rac*-16 168

13. References **170**

1. General Experimental Information

Unless otherwise stated, all reagents were purchased from commercial sources (Acros Organics, Alfa Aesar, Fisher Scientific, FluoroChem, Sigma Aldrich and VWR) and used without further purification. $[Cu(CH_3CN)_4]PF_6$ was prepared as described by Pigorsch and Köckerling.[1] Anhydrous solvents were purchased from Acros Organics. Petrol refers to the fraction of petroleum ether boiling in the range 40-60 °C. IPA refers to isopropanol. THF refers to tetrahydrofuran. EDTA-NH_3 solution refers to an aqueous solution of NH_3 (17% w/w) saturated with sodium-ethylenediaminetetraacetate. $CDCl_3$ (without stabilising agent) was distilled over $CaCl_2$ and K_2CO_3 prior to use. Unless otherwise stated, all reaction mixtures were performed in oven dried glassware under an inert N_2 atmosphere with purchased anhydrous solvents. Unless otherwise stated experiments carried out in sealed vessels were performed in CEM microwave vials, with crimped aluminium caps, with PTFE septa. Young's tap vessels and Schlenk techniques were used where specified.

Flash column chromatography was performed using Biotage Isolera-4 or Isolera-1 automated chromatography system. SiO_2 cartridges were purchased commercially Biotage (SNAP or ZIP (50 µm), or Sfär (60 µm) irregular silica, default flow rates). Neutralised SiO_2 refers to ZIP cartridges which were eluted with petrol-NEt_3 (99 : 1, 5 column volumes), followed by petrol (5 column volumes). Analytical TLC was performed on pre-coated silica gel plates on aluminum (0.25 mm thick, 60F254, Merck, Germany) and observed under UV light (254 nm) or visualised with $KMnO_4$ stain.

All melting points were determined using a Griffin apparatus. NMR spectra were recorded on Bruker AV400 or AV500 instrument, at a constant temperature of 298 K. Chemical shifts are reported in parts per million from low to high field and referenced to residual solvent. Coupling constants (*J*) are reported in Hertz (Hz). Standard abbreviations indicating multiplicity were used as follows: m = multiplet, quint = quintet, q = quartet, t = triplet, d = doublet, s = singlet, app. = apparent, br = broad, sept = septet. Signal assignment was carried out using 2D NMR methods (COSY, NOESY, HSQC or HMBC) where necessary. In some cases, complex multiplets with multiple contributing proton signals, exact assignment was not possible. In interlocked compounds, all proton signals corresponding to axle components are in lower case, and all proton signals corresponding to the macrocycle components are in upper case.

Many of the signals analysed to determine diastereopurity were close in ppm and/or broad, which limited the potential to use Q-NMR methodology (https://nmrweb.chem.ox.ac.uk/Data/Sites/70/userfiles/pdfs/quantitative-nmr.pdf). For this reason, we systematically applied the "peak integration" function implemented in MestReNova (v11.0.4, Mestrelab Research S. L.) combined with the GSD peak modelling function (4 rounds of refinement, optimised for broad peaks), which has been shown to be comparable in accuracy to sum integration even when peaks are overlapping (qGSD - quantitative Global Spectral Deconvolution - Mestrelab Resources). Prior to integration, the default polynomial baseline correction was applied. Where possible, the values obtained were improved by comparison of multiple signals. The corresponding values obtained by sum integration are provided for comparison. Residual intensity is included for all peak integrations. Integral curves are included for sum integrations. Full details are included in the captions of the corresponding spectra.

Low resolution mass spectrometry was carried out by the mass spectrometry services at University of Southampton (Waters TQD mass spectrometer equipped with a triple quadrupole analyser with UHPLC injection [BEH C18 column; CH_3CN -H_2O gradient (0.2% formic acid)]). High resolution mass spectrometry was either carried out by the mass spectrometry services at the University of Southampton (MaXis, Bruker Daltonics, with a Time of Flight (TOF) analyser; samples were introduced to the mass spectrometer via a Dionex Ultimate 3000 autosampler and uHPLC pump in a gradient of 20% CH_3CN in *n*-hexane to 100% acetonitrile (0.2% formic acid) over 5-10 min at 0.6 mL/min; column: Acquity UPLC BEH C18 (Waters) 1.7 micron 50 × 2.1mm) or services at University of Birmingham (Waters Synapt G2-S mass spectrometer fitted with a TOF detector).

Circular dichroism spectra were either acquired on an Applied Photo-physics Chirascan spectropolarimeter, recorded using Applied Photophysics software Ver. 4.2.0 or a Jasco J-1500spectropolarimeter in dried spectroscopic grade $CHCl_3$ in a quartz cell of 1 cm path length, at a temperature of 293 K.

Stereochemical purity was determined by Chiral Stationary Phase HPLC on a Waters Acquity Arc Instrument at 303 K, with *n*-hexane-^{i}PrOH or *n*-hexane-EtOH isocratic eluents. Regis Technologies (S,S)-Whelk-O1 (1-(3,5-dinitrobenzamido)-1,2,3,4-tetrahydrophenanthrene stationary phase), RegisCell (tris-(3,5-dimethylphenyl) carbamoyl cellulose stationary phase), Regis Reflect I-Cellulose B (3,5-dimethylphenylcarbamate stationary phase), and Regis Reflect I-Cellulose Z (3-chloro-4-methylphenylcarbamate stationary phase) columns were used throughout (5 micron, column dimensions 25 cm x 4.6 mm). Racemic samples were prepared employing the same synthetic procedures and but starting from *rac*-**1a**.

The following compounds were synthetized according to literature procedures: **1a** ((*R*)-**1a** and (*S*)-**1a**)),[2] **2**,[2] **3**,[3] and **5**.[4]

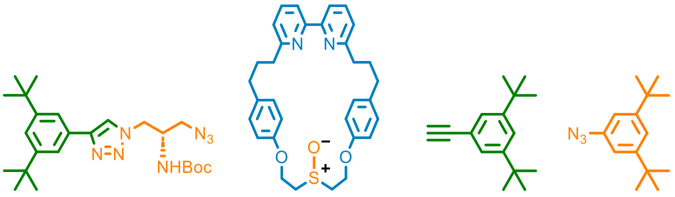

1a **2** **3** **5**

2. Synthesis of sulfide macrocycle precursor S3

Macrocycle **2** was synthesised from precursor **S3** according to our previously reported procedure.[2] However, precursor **S3** was produced using a new procedure (below) that avoids the isolation of the corresponding dibromide of **S1**, which is a bifunctional electrophile and thus raises toxicity concerns.

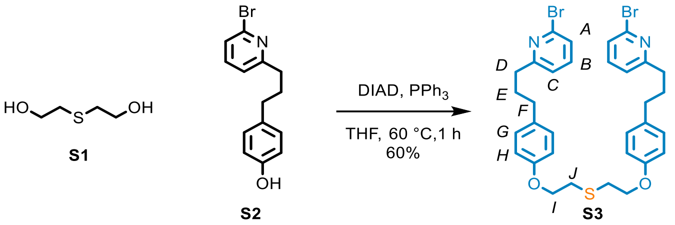

To a solution of PPh_3 (2.6 g, 10.0 mmol) and **S2** (2.9 g, 10.0 mmol) in THF (36 mL) was added **S1** (360 µL, 3.6 mmol), followed by DIAD (1.4 mL, 7.2 mmol) dropwise then the reaction was stirred at 60 °C for 1 h. After cooling to rt, the reaction mixture was washed with H_2O. The aqueous and organic phases were separated, and the aqueous phase was then extracted with EtOAc (3 x 50 mL). The combined organic extracts were washed with brine (100 mL), dried ($MgSO_4$) and concentrated *in vacuo*. Chromatography (petrol:CH_2Cl_2 1 : 1) gave **S3** as a white solid (1.4 g, 60%).

All spectroscopic data is consistent with literature reported data.[2]

^{1}H NMR (400 MHZ, $CDCl_3$) δ 7.42 (t, *J* = 7.7, 2H, H_B), 7.29 (d, *J* = 7.9, 2H, H_A), 7.12 – 7.03 (m, 6H, H_C, H_G), 6.86 – 6.77 (m, 4H, H_H), 4.16 (t, *J* = 6.6, 4H, H_I), 3.00 (t, *J* = 6.7, 4H, H_J), 2.81 – 2.73 (m, 4H, H_D), 2.65 – 2.57 (m, 4H, H_F), 2.06 – 1.94 (m, 4H, H_E)

^{13}C NMR (100 MHz, CDCl3) δ 163.9, 156.9, 141.7, 138.7, 134.6, 129.6, 125.5, 121.7, 114.7, 68.2, 37.6, 34.7, 31.9, 31.6.

3. Rotaxanes 4a-e and their precursors (Scheme 1, main text)

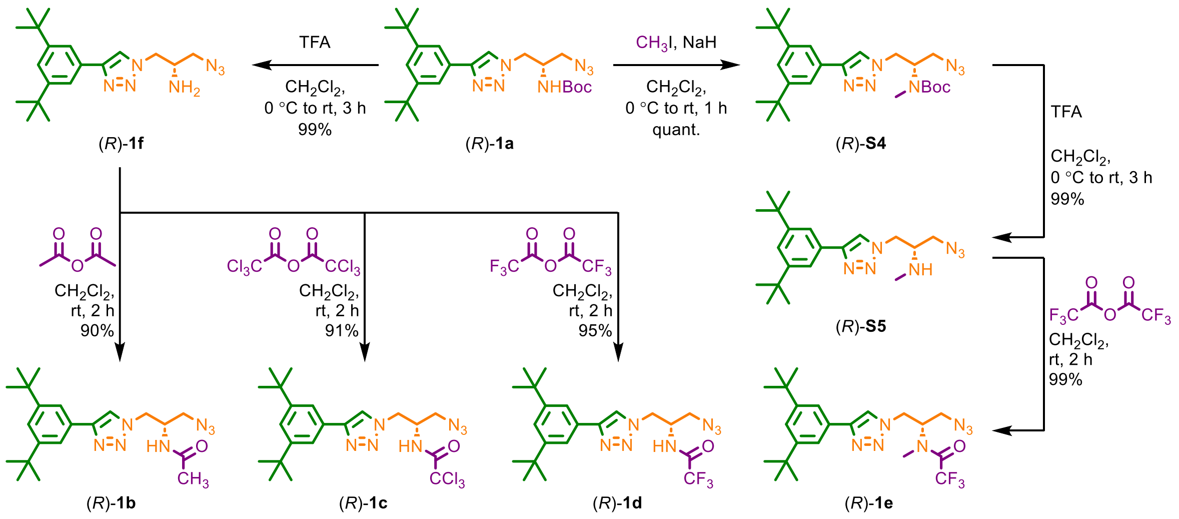

Scheme S1. Synthetic route to azides (*R*)-**1b-e**.

Amine azide (*R*)-1f

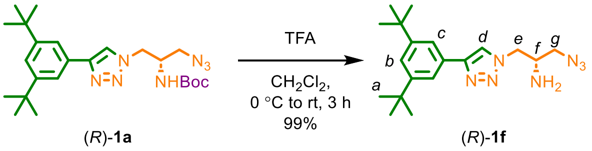

To a solution of (*R*)-**1a** (100.7 mg, 0.22 mmol) in CH_2Cl_2 (2.2 mL) was added TFA (170 µL, 2.22 mmol) at 0 °C. The reaction mixture was allowed to warm to rt and stirred for 3 h. The reaction mixture was then diluted with CH_2Cl_2 (10 mL) and poured slowly into saturated $NaHCO_3$ (10 mL). The aqueous and organic phases were separated, and the aqueous phase was then extracted with CH_2Cl_2 (3 x 5 mL). The combined organic extracts were washed with brine (10 mL), dried ($MgSO_4$) and concentrated *in vacuo* to afford (*R*)-**1f** (78.1 mg, 99%) as a white solid without further purification.

Amine azide (*S*)-**1f** (78.1 mg, quant.) was synthesised using an identical procedure starting from (*S*)-**1f** (100.7 mg, 0.22 mmol). Analytical data was identical to (R)-**1f** with the exeption of their their CD spectra (Figure S6) and CSP-HPLC (Figure S7).

^{1}H NMR (400 MHz, CDCl$_3$) δ: 7.85 (s, 1H, H_d), 7.67 (d, *J* = 1.8, 2H, H_c), 7.43 (t, *J* = 1.8, 1H, H_b), 4.47 (dd, *J* = 13.8, 4.9, 1H, H_e), 4.36 (dd, *J* = 13.8, 6.8, 1H, $H_{e'}$), 3.54 – 3.39 (m, 2H, H_f, H_g), 3.35 (dd, *J* = 12.1, 5.7, 1H, $H_{g'}$), 1.53 (br. s, 2H, NH_2), 1.37 (s, 18H, H_a);

^{13}C NMR (101 MHz, CDCl$_3$) δ: 151.6, 148.9, 129.7, 122.7, 120.9, 120.3, 55.1, 54.0, 51.4, 35.1, 31.6;

HR-ESI-MS (+ve) m/z = 356.2566 $[M+H]^+$ (calc. m/z for $C_{19}H_{30}N_7$ 356.2557);

$[\alpha]_D^{23}$+0.6 ((*R*)-**1f**, c 0.72, CHCl$_3$), -0.5 ((*S*)-**1f**, c 0.74, CHCl$_3$);

Melting point 122-124 °C.

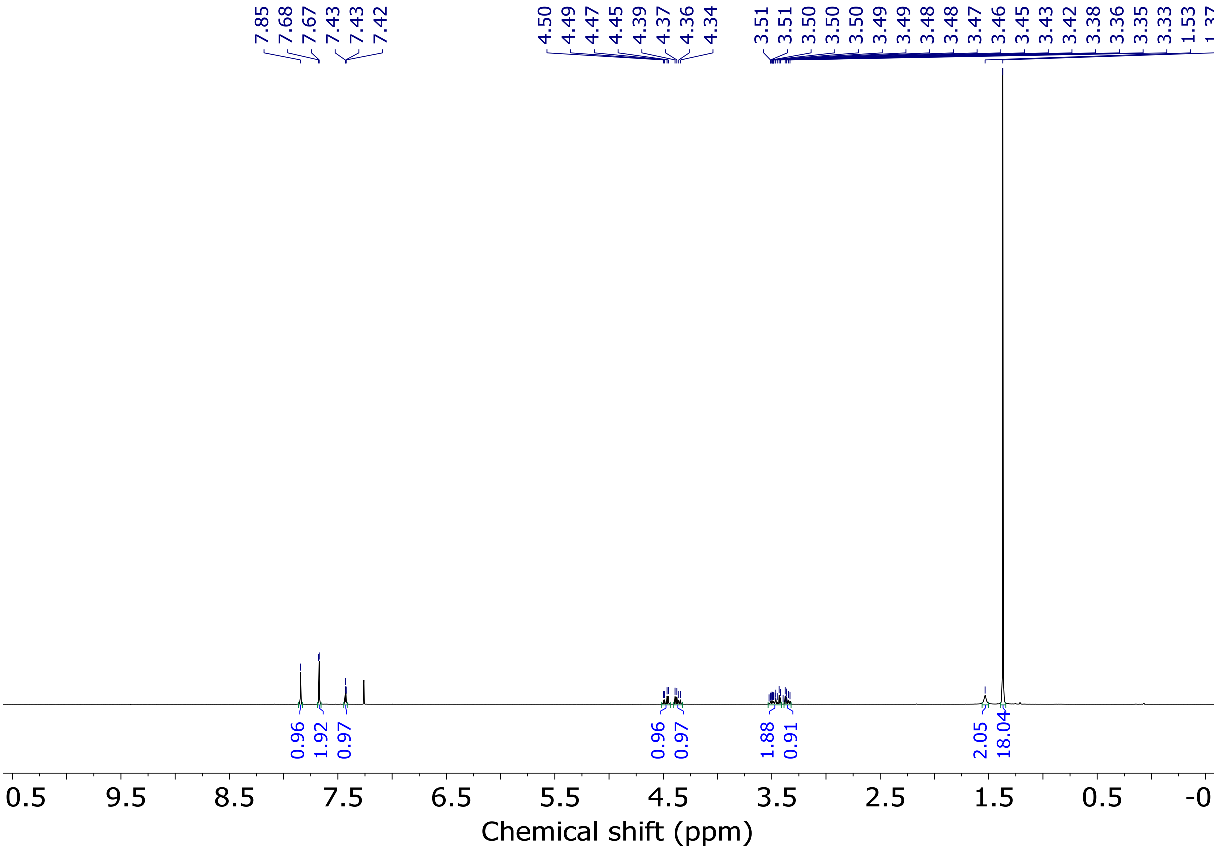

Figure S1. ^{1}H NMR ($CDCl_3$, 400 MHz) of (*R*)-**1f**.

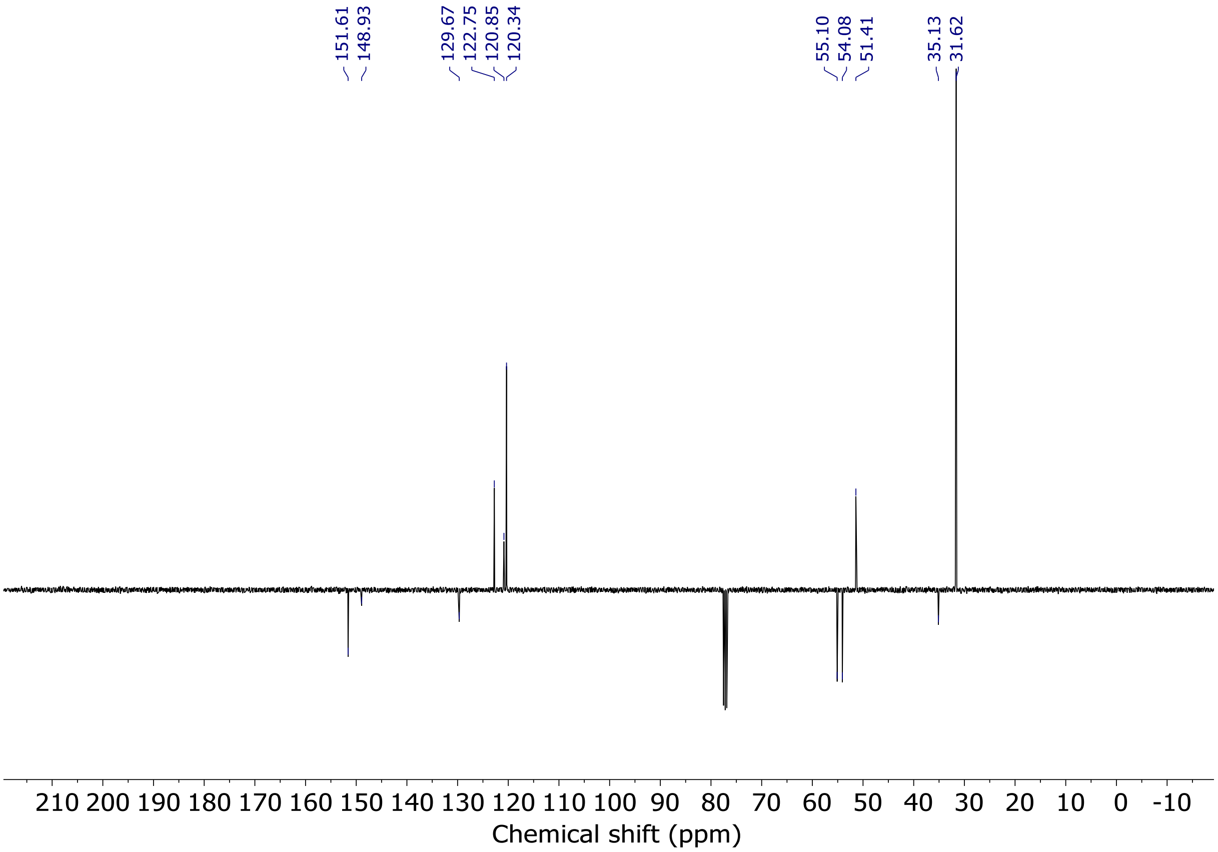

Figure S2. JMOD NMR ($CDCl_3$, 101 MHz) of (*R*)-**1f**.

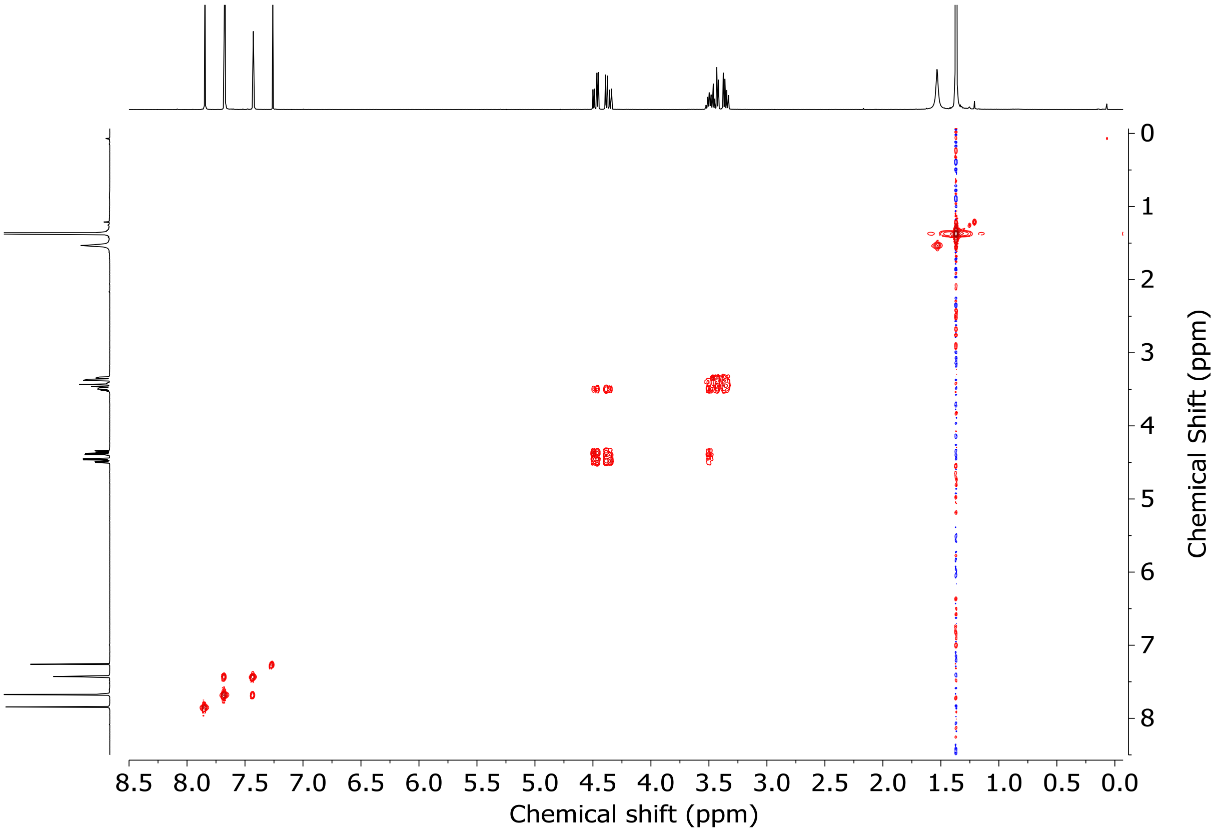

Figure S3. ^{1}H COSY NMR ($CDCl_3$) of (*R*)-**1f**.

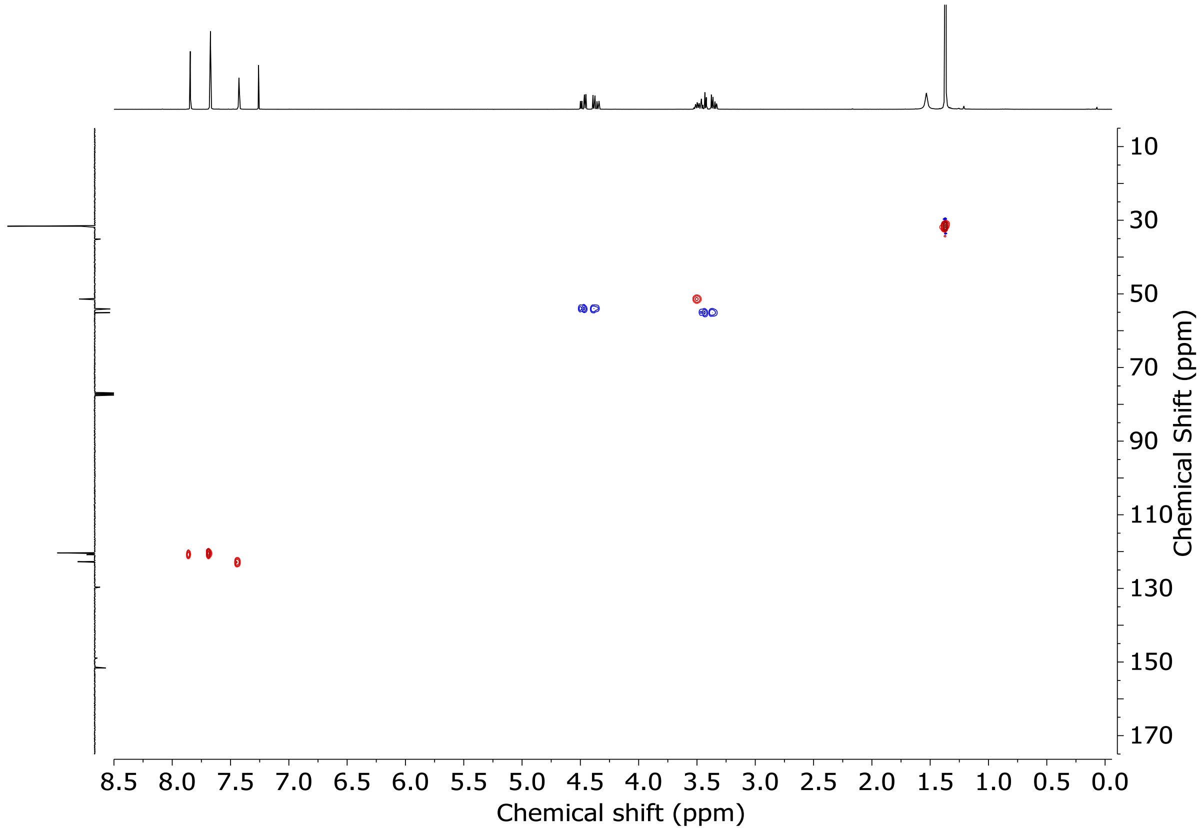

Figure S4. HSQC NMR ($CDCl_3$) of (*R*)-**1f**.

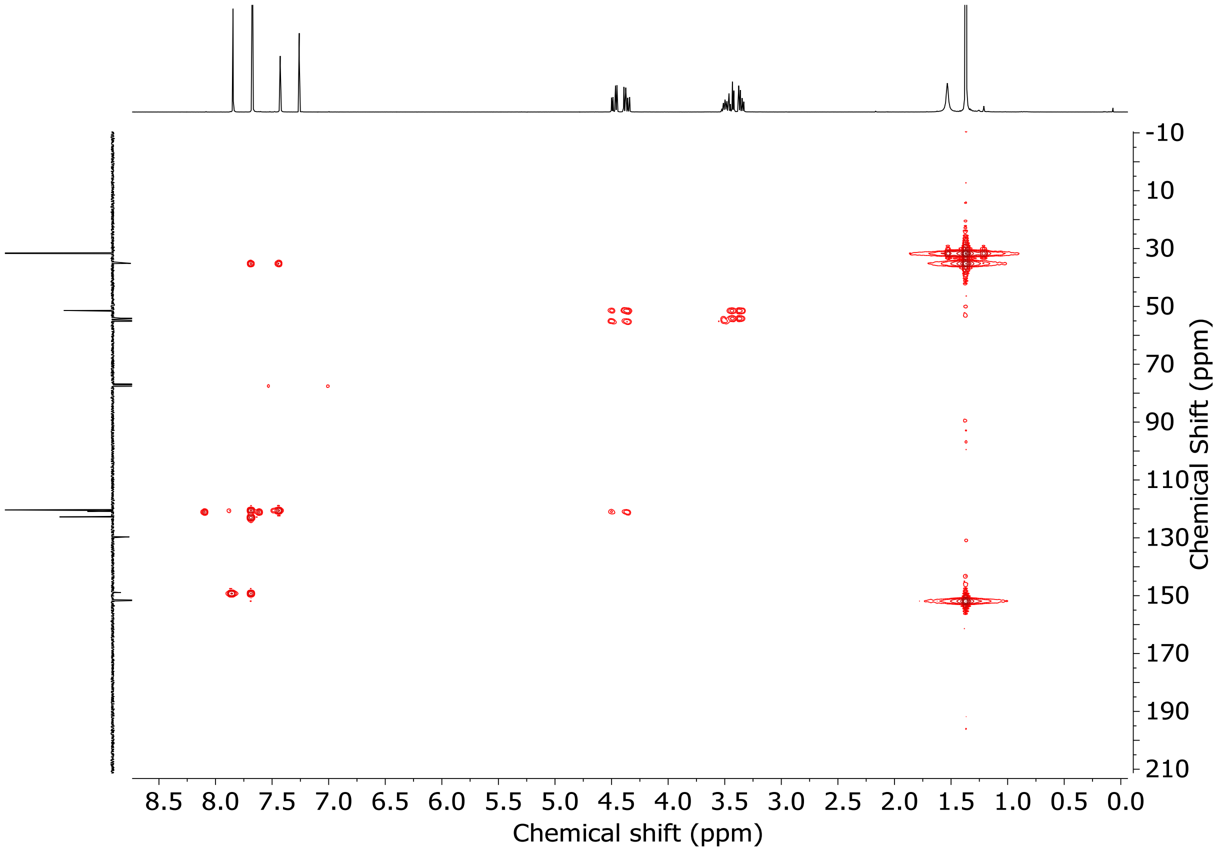

Figure S5. HMBC NMR ($CDCl_3$) of (*R)*-**1f**

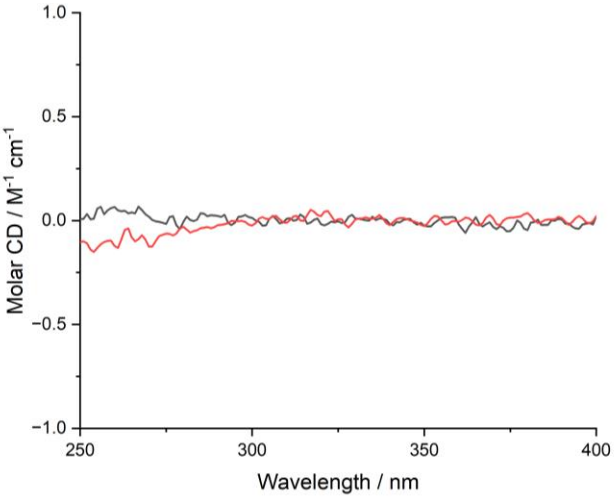

Figure S6. Circular Dichroism Spectra of (*R*)-**1f** (69 µM, grey) and (*S*)-**1f** (69 µM, red) at 293 K in $CHCl_3$. No measurable CD response was observed so the $[\alpha]_D$ of (*R*)-**1f** and (*S*)-**1f** wer measured.

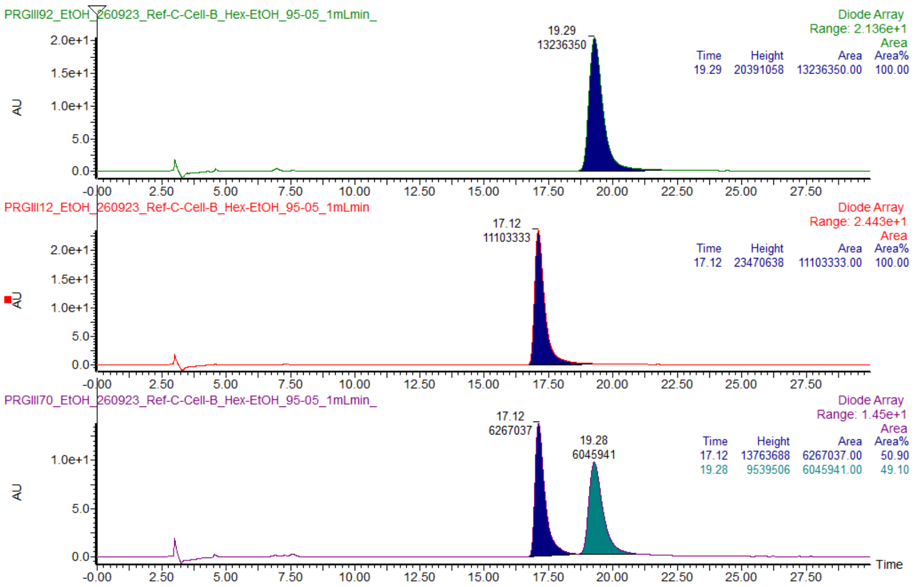

Figure S7. CSP-HPLC of **1f** (loaded in EtOH). Regis Reflect I-Cellulose B, *n*-hexane-EtOH 95 : 5, flowrate 1 mLmin^{-1}. (top) (*R*)-**1f**; (*S*)-**1f** (not observed), (*R*)-**1f** (19.29 min, 13236350, >99.9%). (middle) (*S*)-**1f**; (*S*)-**1f** (17.12 min, 11103333, >99.9%), (*R*)-**1f** (not observed). (bottom) *rac*-**1f**; (*S*)- 11f (17.12 min, 6267037, 50.9%), (*S*)-**1f** (19.28 min, 6045941, 49.1%).

Acetamide azide (*R*)-1b

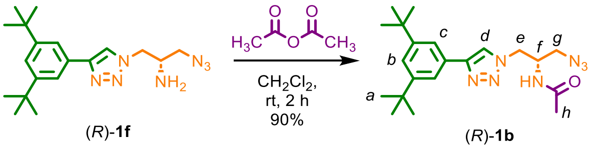

To a solution of (*R*)-**1f** (50.0 mg, 0.14 mmol) in CH_2Cl_2 (1.0 mL) was added acetic anhydride (19.0 µL, 0.20 mmol) dropwise, then allowed to stir at rt for 2 h. The reaction mixture was then diluted with CH_2Cl_2 (2 mL) and poured slowly into sat. $NaHCO_{3\,(aq)}$ (5 mL). The aqueous and organic phases were separated, and the aqueous phase was then extracted with CH_2Cl_2 (3 x 5 mL). The combined organic extracts were washed with brine (5 mL), dried ($MgSO_4$) and concentrated *in vacuo*. Chromatography (petrol-EtOAc 0→50%) gave (*R*)-**1b** (50.1 mg, 90%) as a white solid.

^{1}H NMR (400 MHz, CDCl$_3$) δ: 7.95 (s, 1H, H$_d$), 7.66 (d, *J* = 1.8, 2H, H$_c$), 7.44 (t, *J* = 1.9, 1H, H$_b$), 6.41 (d, *J* = 8.2, 1H, NH), 4.63 (dd, *J* = 5.4, 14.0, H$_e$), 4.56 (dd, *J* = 5.1, 14.0, H$_{e'}$), 4.48 (app ddt, *J* = 10.0, 5.4, 7.4, 1H, H$_f$), 3.58 (dd, *J* = 7.4, 12.2, H$_g$) , 3.25 (dd, *J* = 7.4, 12.2, H$_{g'}$), 2.01 (s, 3H, H$_h$), 1.37 (s, 18H, H$_a$) ;

^{13}C NMR (101 MHz, CDCl$_3$) δ: 170.3, 151.6, 149.0, 129.2, 122.8, 121.0, 120.2, 50.6, 50.0, 48.8, 35.0, 31.4, 23.3;

HR-ESI-MS (+ve) m/z = 398.2666 [M+H]$^+$ (calc. 398.2333 m/z for $C_{21}H_{32}N_7O$);

Melting point = 118-120 °C.

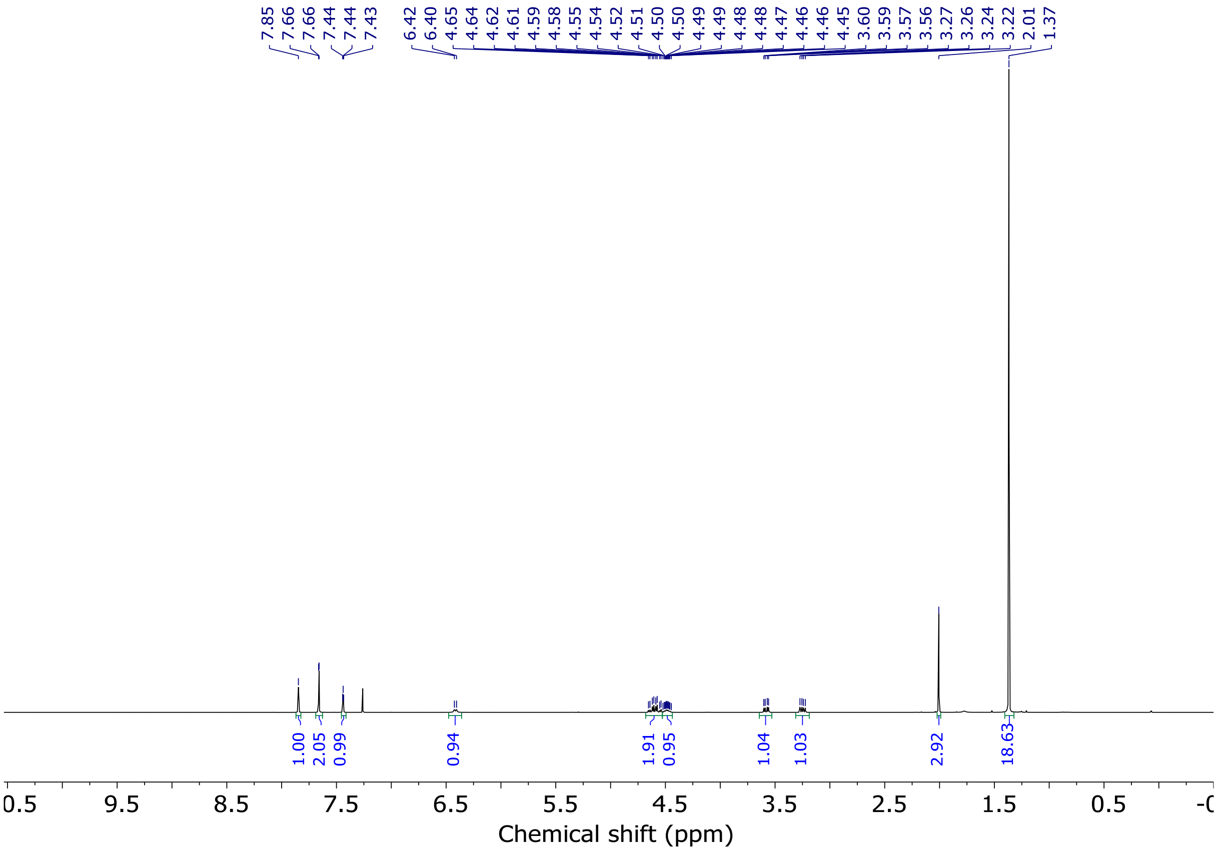

Figure S8. ^{1}H NMR ($CDCl_3$, 400 MHz) of (*R)*-**1b**.

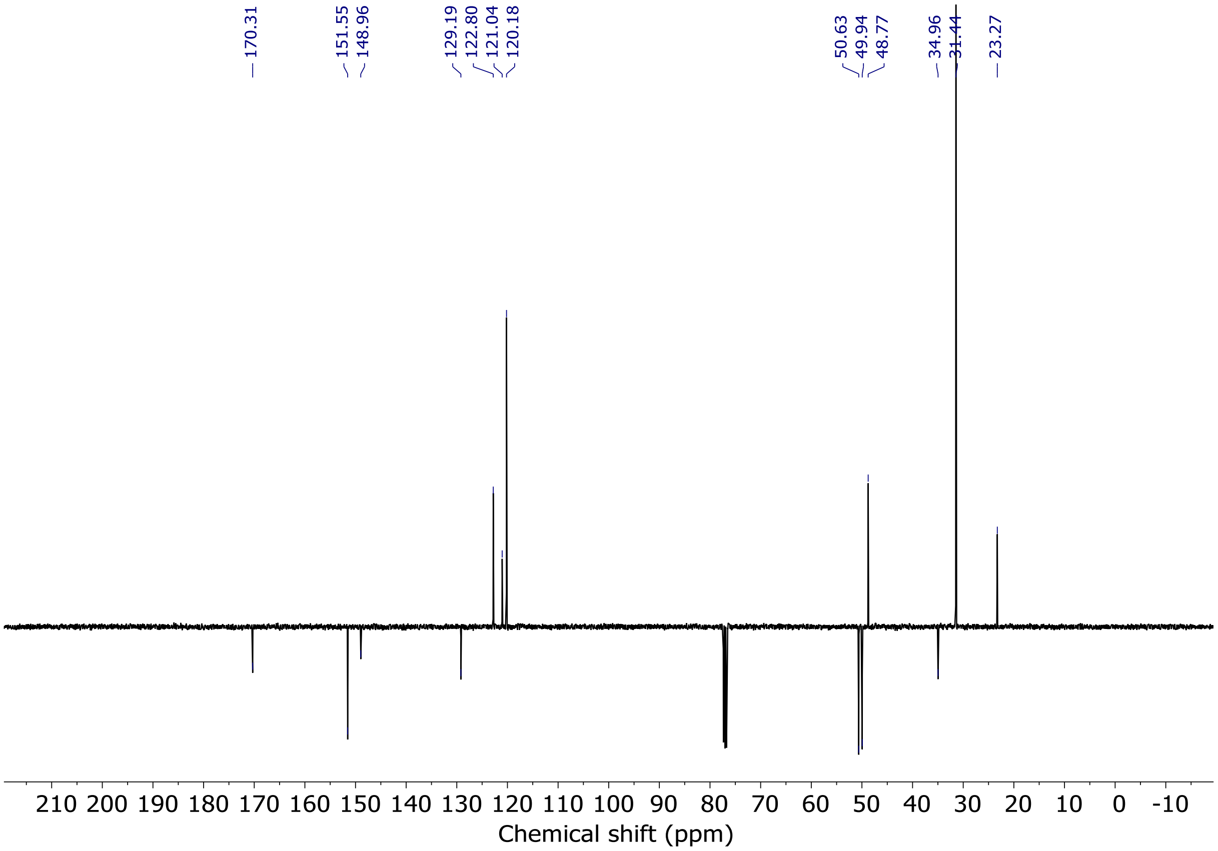

Figure S9. JMOD NMR ($CDCl_3$, 101 MHz) of (*R)*-**1b**

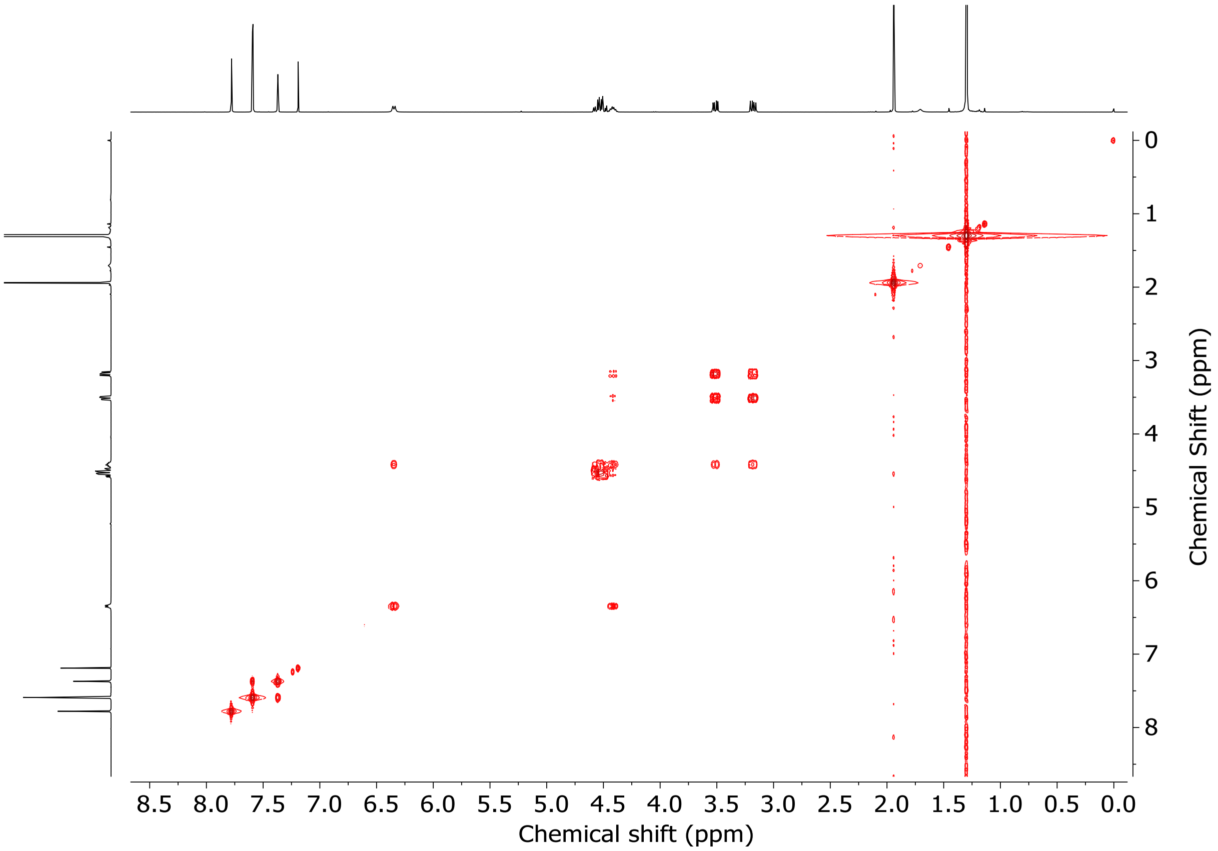

Figure S10. COSY NMR ($CDCl_3$) of (*R*)-**1b**

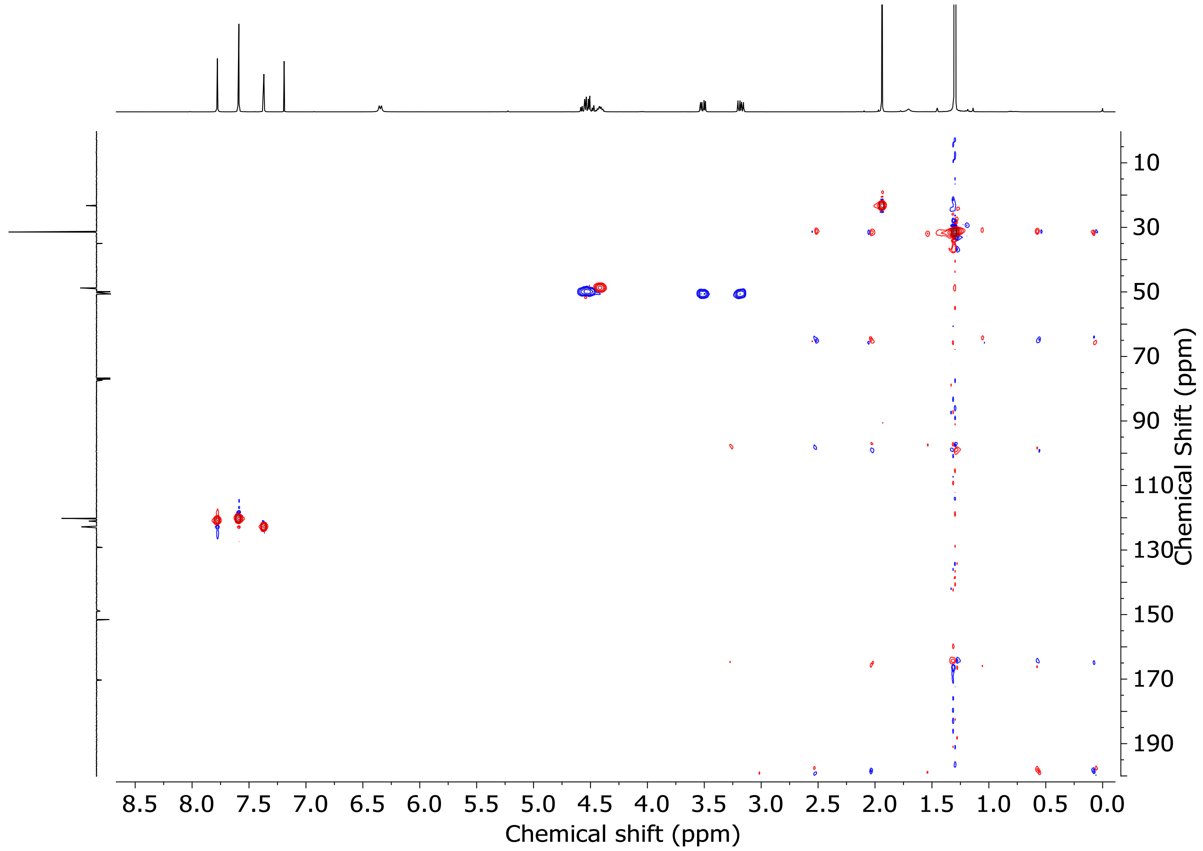

Figure S11. HSQC NMR ($CDCl_3$) of (*R)*-**1b**

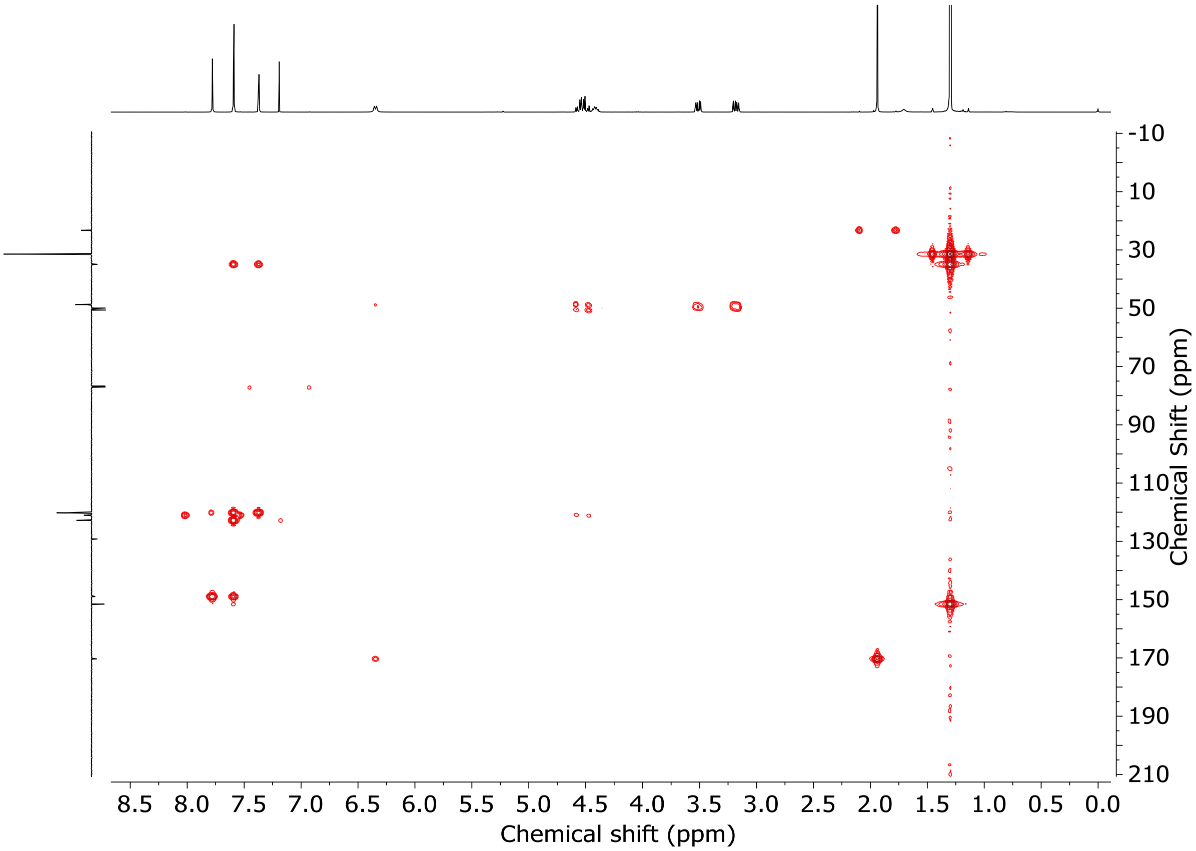

Figure S12. HMBC NMR ($CDCl_3$) of (*R)*-**1b**.

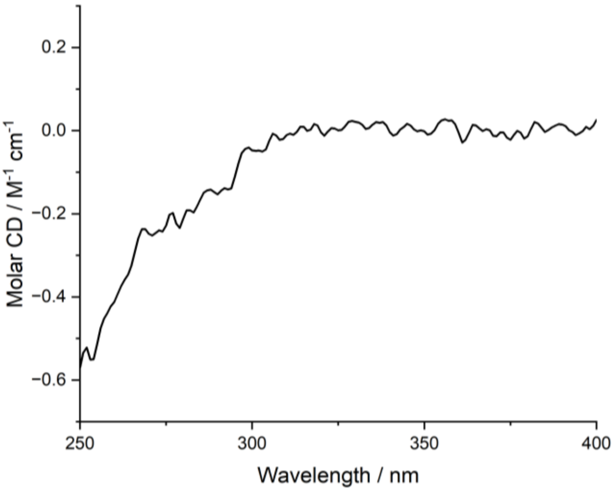

Figure S13. Circular Dichroism Spectra of (R)-**1b** (88 μM) at 293 K in $CHCl_3$.

Trichloroacetamide azide (*R*)-1c

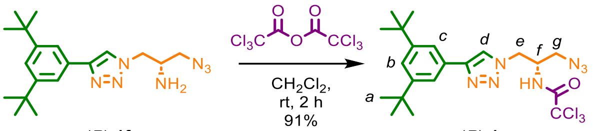

(*R*)-**1f** (*R*)-**1c**

To a solution of (*R*)-**1f** (50.0 mg, 0.14 mmol) in CH_2Cl_2 (1.0 mL) was added trichloroacetic anhydride (38.1 µL, 0.20 mmol) dropwise, then allowed to stir at rt for 2 h. The reaction mixture was then diluted with CH_2Cl_2 (2 mL) and poured slowly into sat. $NaHCO_{3\ (aq)}$ (5 mL). The aqueous and organic phases were separated, and the aqueous phase was then extracted with CH_2Cl_2 (3 x 5 mL). The combined organic extracts were washed with brine (5 mL), dried ($MgSO_4$) and concentrated *in vacuo*. Chromatography (petrol-EtOAc 0→50%) gave (*R*)-**1c** (63.8 mg, 91%) as a white solid.

^{1}H NMR (400 MHz, CDCl$_3$) δ:7.86 (s, 1H, H$_d$), 7.80 (d, *J* = 7.9, 1H, NH), 7.66 (d, *J* = 1.8, 2H, H$_c$), 7.45 (t, *J* = 1.8, 1H, H$_b$), 4.74 (dd, *J* = 5.2, 14.4, H$_e$), 4.70 (dd, *J* = 5.2, 14.4, H$_{e'}$), 4.51-4.41 (m, 1H, H$_f$), 3.69 (dd, *J* = 5.1, 12.2, H$_g$) , 3.36 (dd, *J* = 7.2, 12.3, H$_{g'}$), 1.37 (s, 18H, H$_a$);

^{13}C NMR (101 MHz, CDCl$_3$) δ: 162.3, 151.6, 149.1, 129.0, 122.0, 121.3, 120.2, 91.9, 50.7, 50.0, 49.3, 35.0, 31.5;

HR-ESI-MS (+ve) m/z = 500.1493 [M+H]$^+$ (calc. 500.1494 m/z for $C_{21}H_{29}Cl_3N_7O$);

Melting point = 146-148 °C.

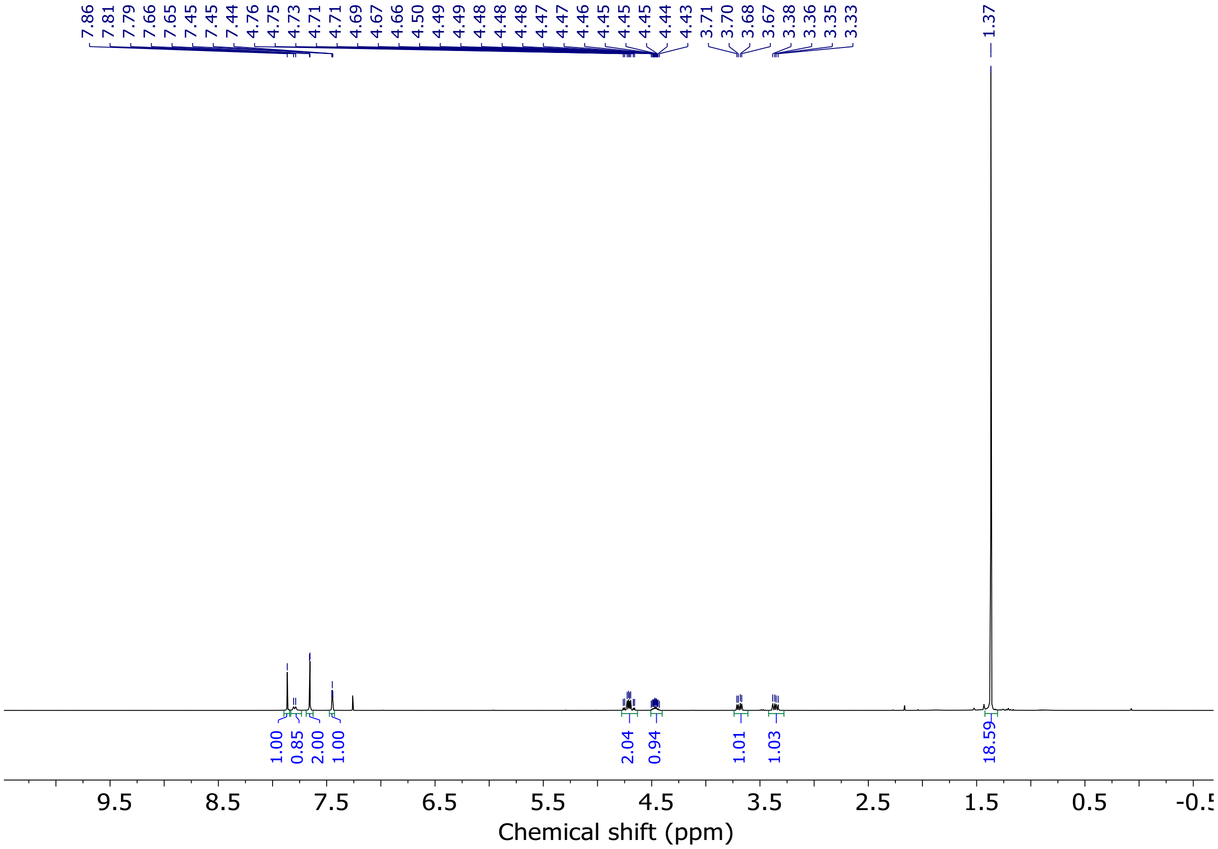

Figure S14. ^{1}H NMR (CDCl$_3$, 400 MHz) of (*R)*-**1c**.

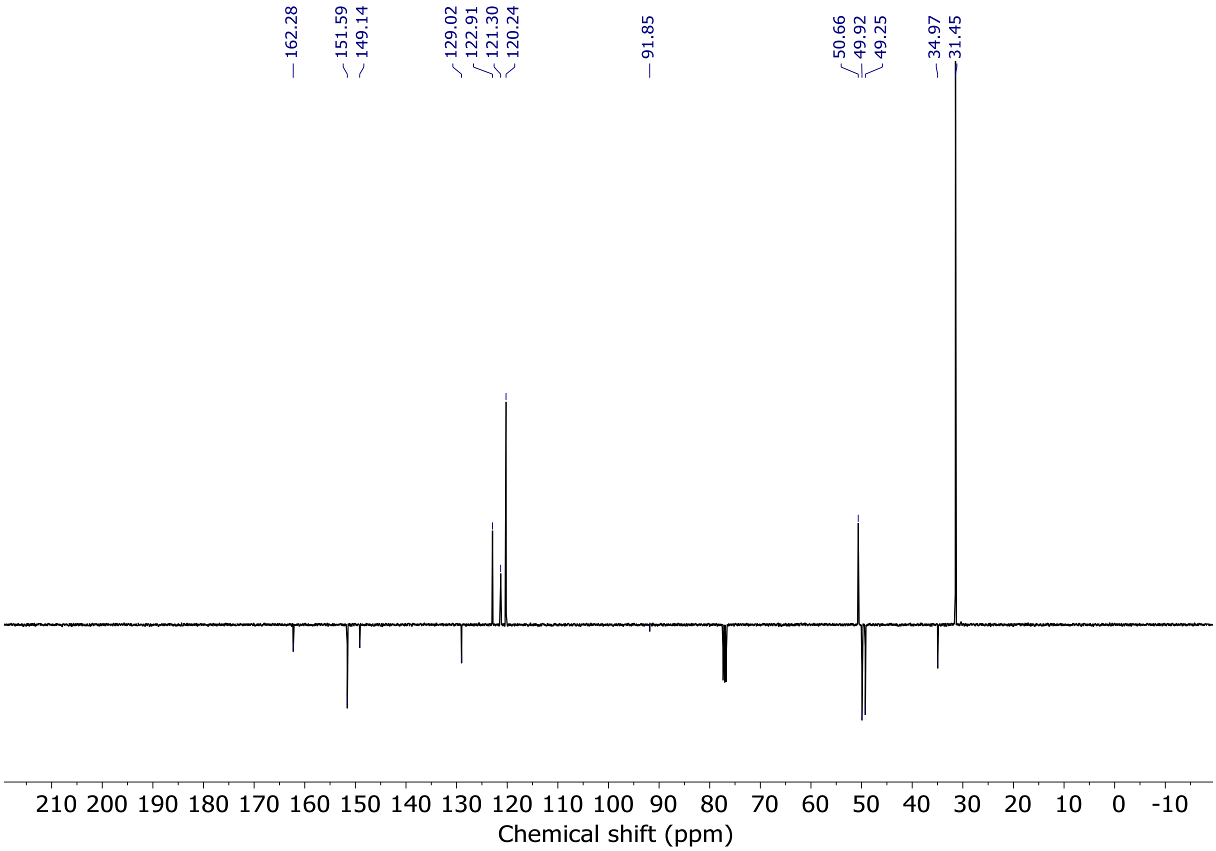

Figure S15. JMOD NMR ($CDCl_3$, 101 MHz) of (*R*)-**1c**.

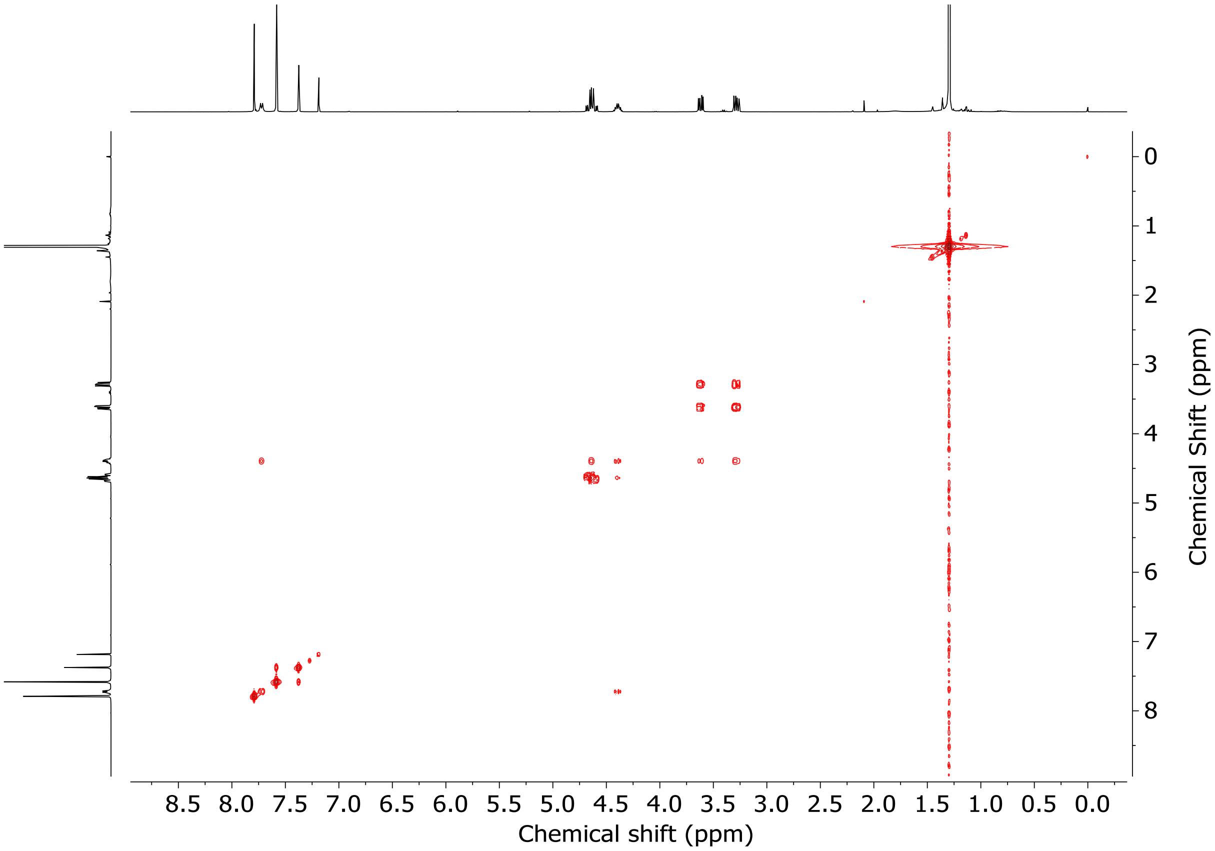

Figure S16. COSY NMR ($CDCl_3$) of (*R*)-**1c**.

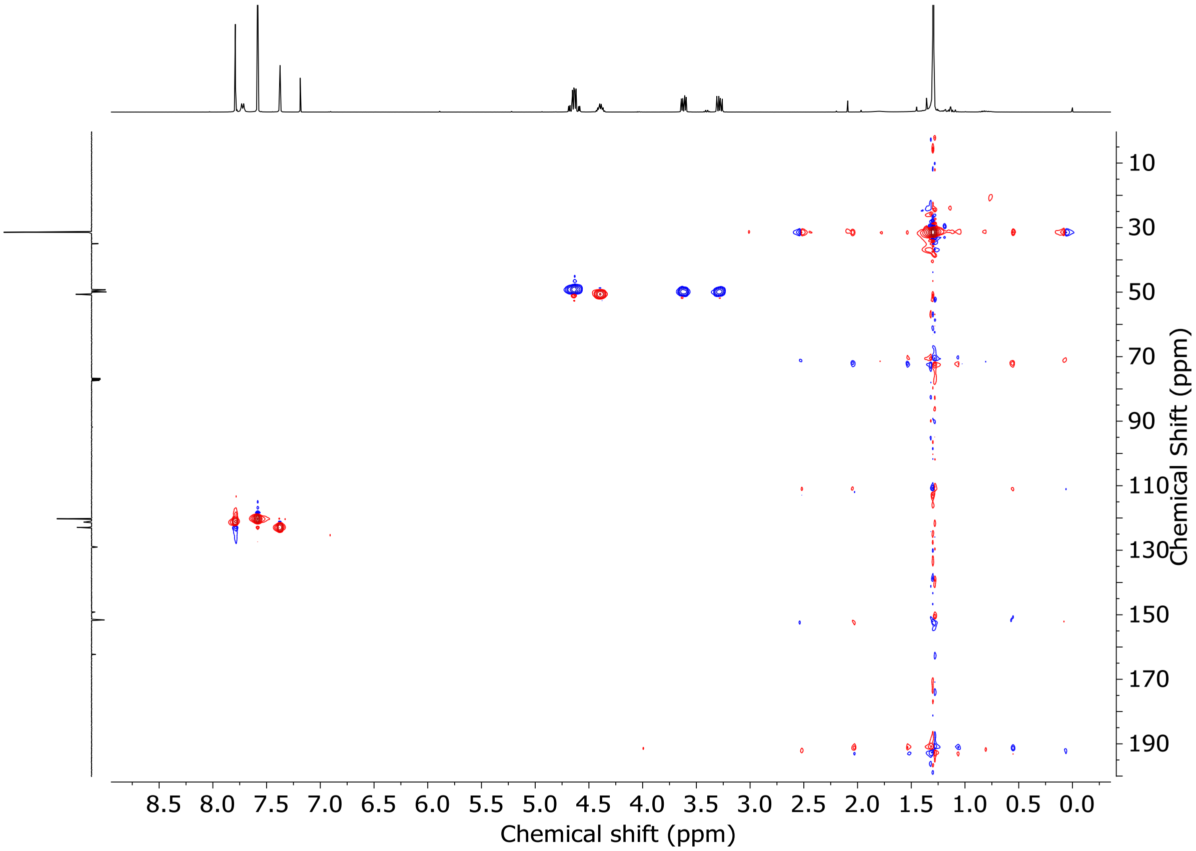

Figure S17. HSQC NMR ($CDCl_3$) of (*R)*-**1c**.

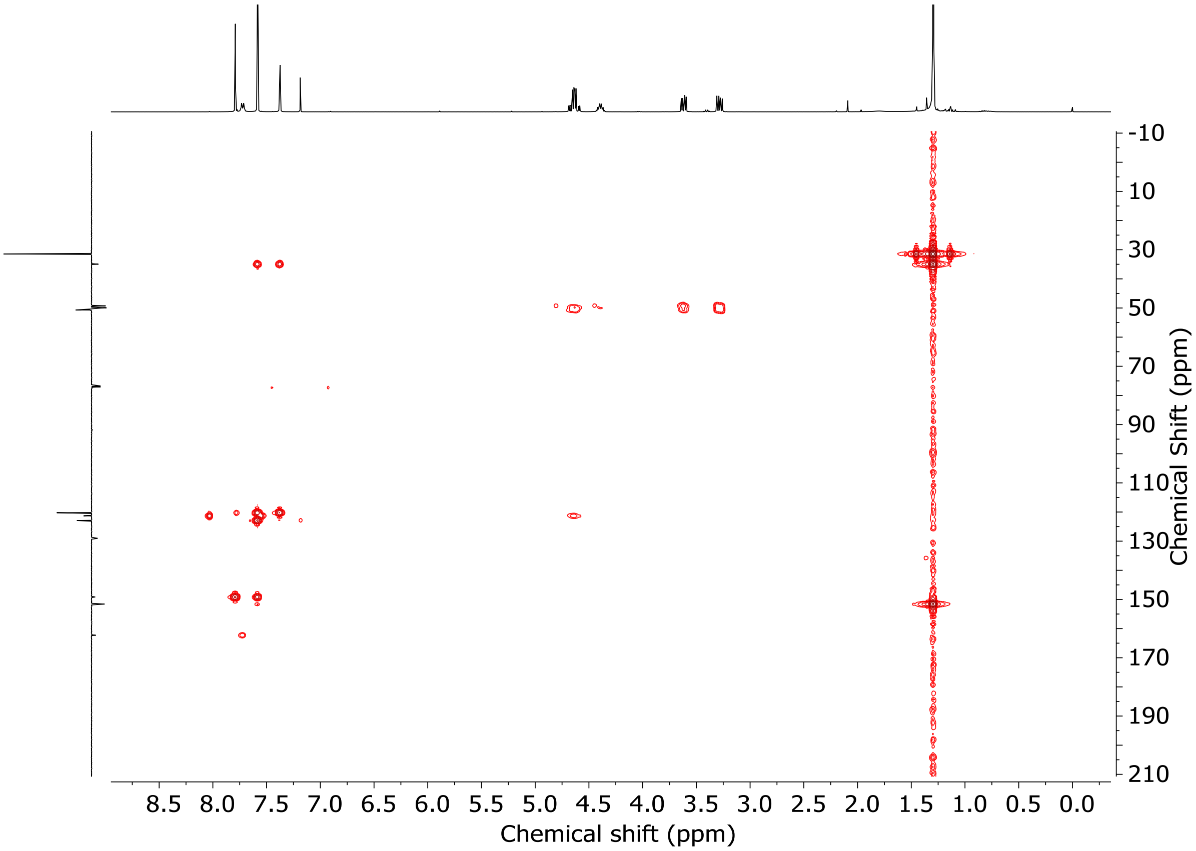

Figure S18. HMBC NMR ($CDCl_3$) of (*R)*-**1c**.

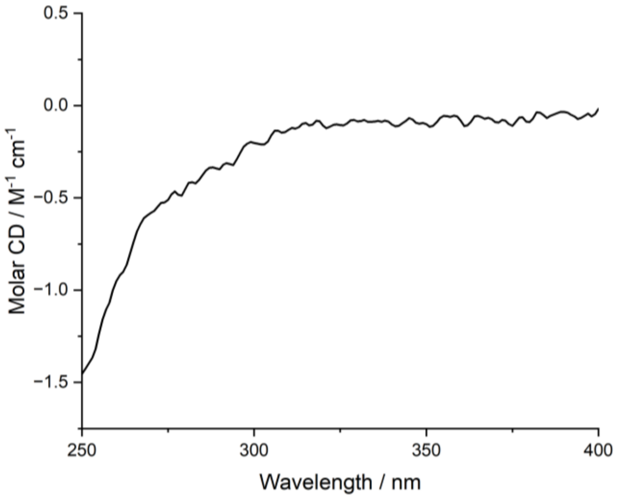

Figure S19. Circular Dichroism Spectra of (*R*)-**1c** (60 µM) at 293 K in $CHCl_3$.

Trifluoroacetamide azide (*R*)-1d

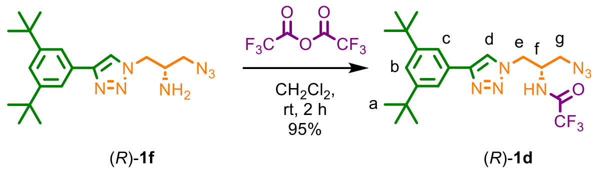

To a solution of (*R*)-**1f** (50.1 mg, 0.14 mmol) in CH_2Cl_2 (0.7 mL) was added trifluoroacetic anhydride (27.4 µL, 0.21 mmol) dropwise, then allowed to stir at rt for 2 h. The reaction mixture was then diluted with CH_2Cl_2 (2 mL) and poured slowly into sat. $NaHCO_{3\ (aq)}$ (5 mL). The aqueous and organic phases were separated, and the aqueous phase was then extracted with CH_2Cl_2 (3 x 5 mL). The combined organic extracts were washed with brine (5 mL), dried ($MgSO_4$) and concentrated *in vacuo*. Chromatography (petrol-EtOAc 0→100%) gave (*R*)-**1d** (60.5 mg, 95%) as a white solid.

^{1}H NMR (400 MHz, $CDCl_3$) δ: 7.98 (d, *J* = 8.1, 1H, NH), 7.88 (s, 1H, H_d), 7.64 (d, *J* = 1.8, 2H, H_c), 7.46 (t, *J* = 1.8, 1H, H_b), 4.67 (qd, *J* = 14.3, 5.4, 2H, H_e), 4.59 – 4.48 (m, 1H, H_f), 3.66 (dd, *J* = 12.6, 5.5, 1H, H_g), 3.39 (dd, *J* = 12.6, 7.1, 1H, $H_{g'}$), 1.36 (s, 18H, H_a);

^{13}C NMR (101 MHz, $CDCl_3$) δ: 157.72 (q, *J* = 38.1), 151.75, 149.28, 129.08, 123.11, 121.39, 120.39, 115.64 (q, *J* = 287.7), 50.11, 49.73, 49.38, 35.07, 31.52;

^{19}F NMR (376 MHz, $CDCl_3$) δ -75.8;

HR-ESI-MS (+ve) m/z = 452.2391 $[M+H]^+$ (calc. m/z for $C_{21}H_{29}F_3N_7O$ 452.2380);

Melting point = 165-167 °C.

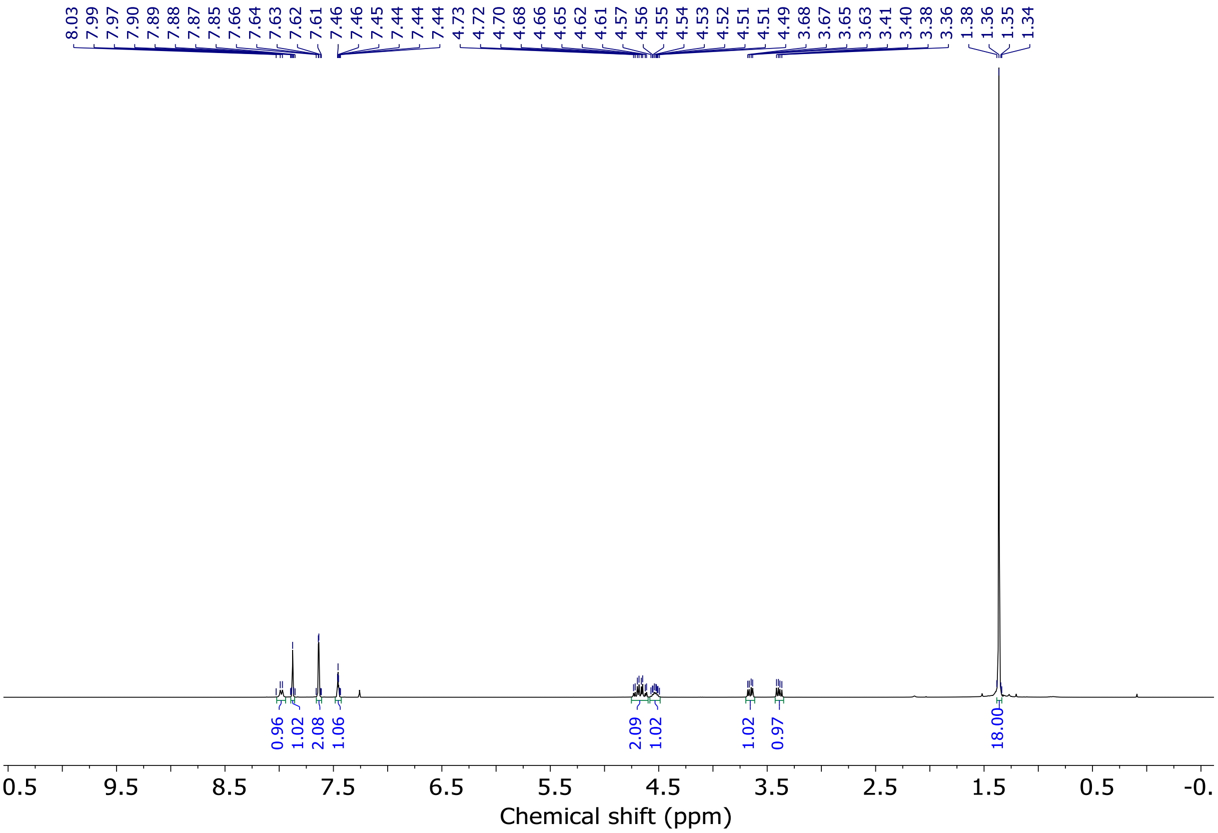

Figure S20. ^{1}H NMR ($CDCl_3$, 400 MHz) of (*R)*-**1d**.

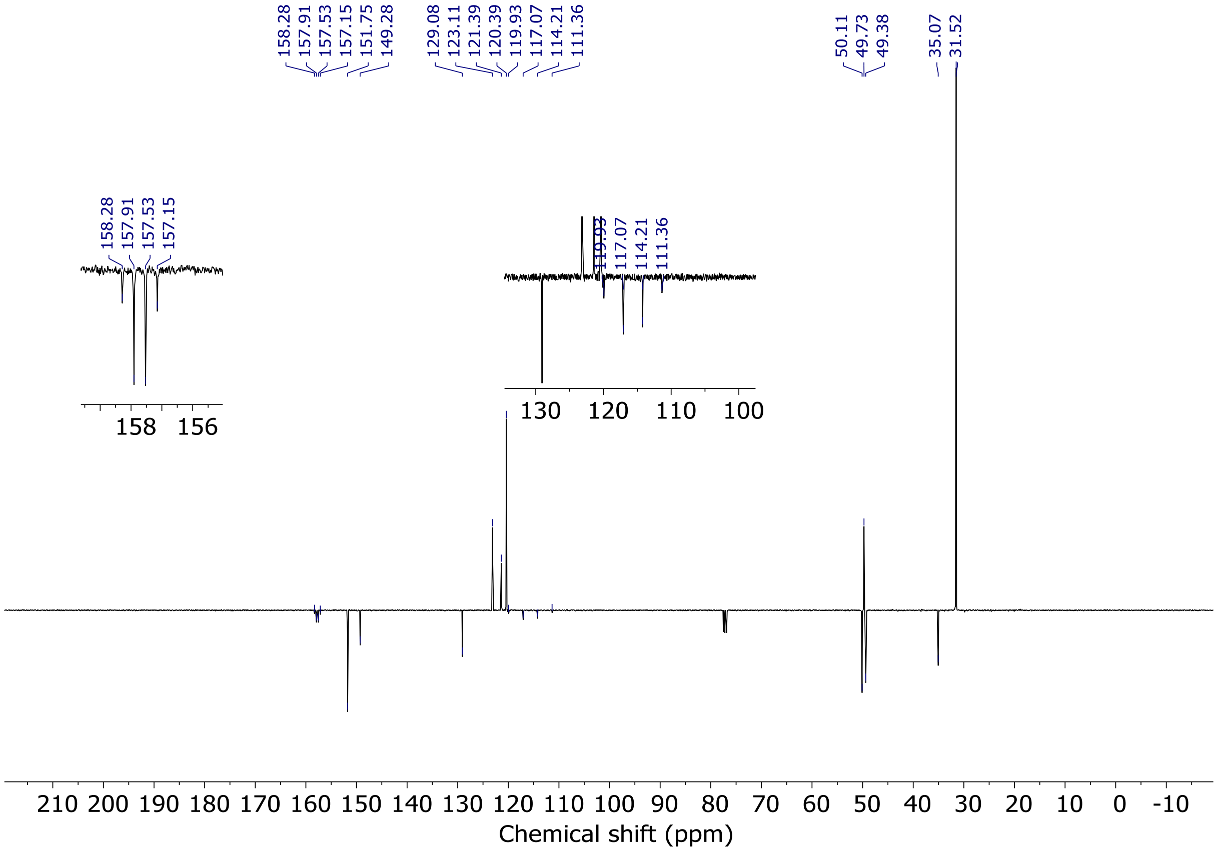

Figure S21. JMOD NMR ($CDCl_3$, 101 MHz) of (*R)*-**1d**.

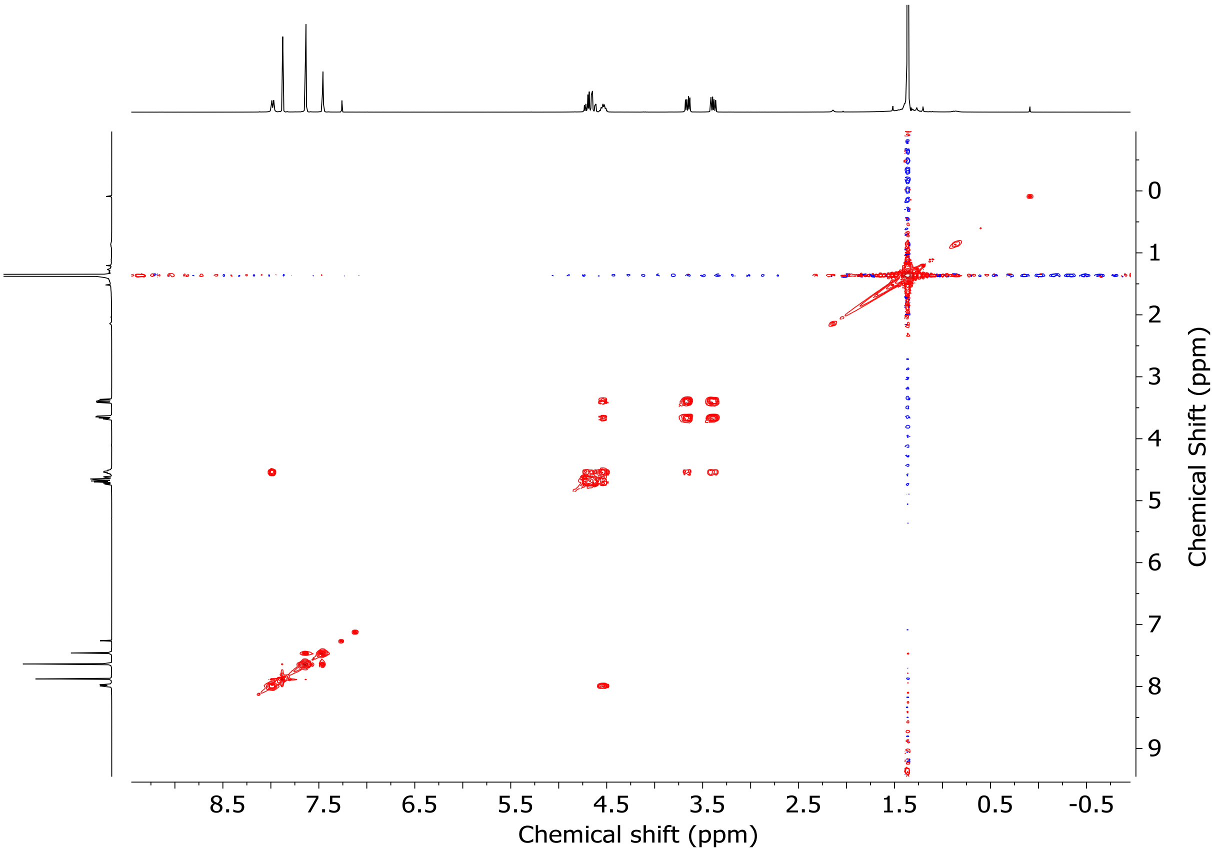

Figure S22. COSY NMR ($CDCl_3$) of (*R*)-**1d**.

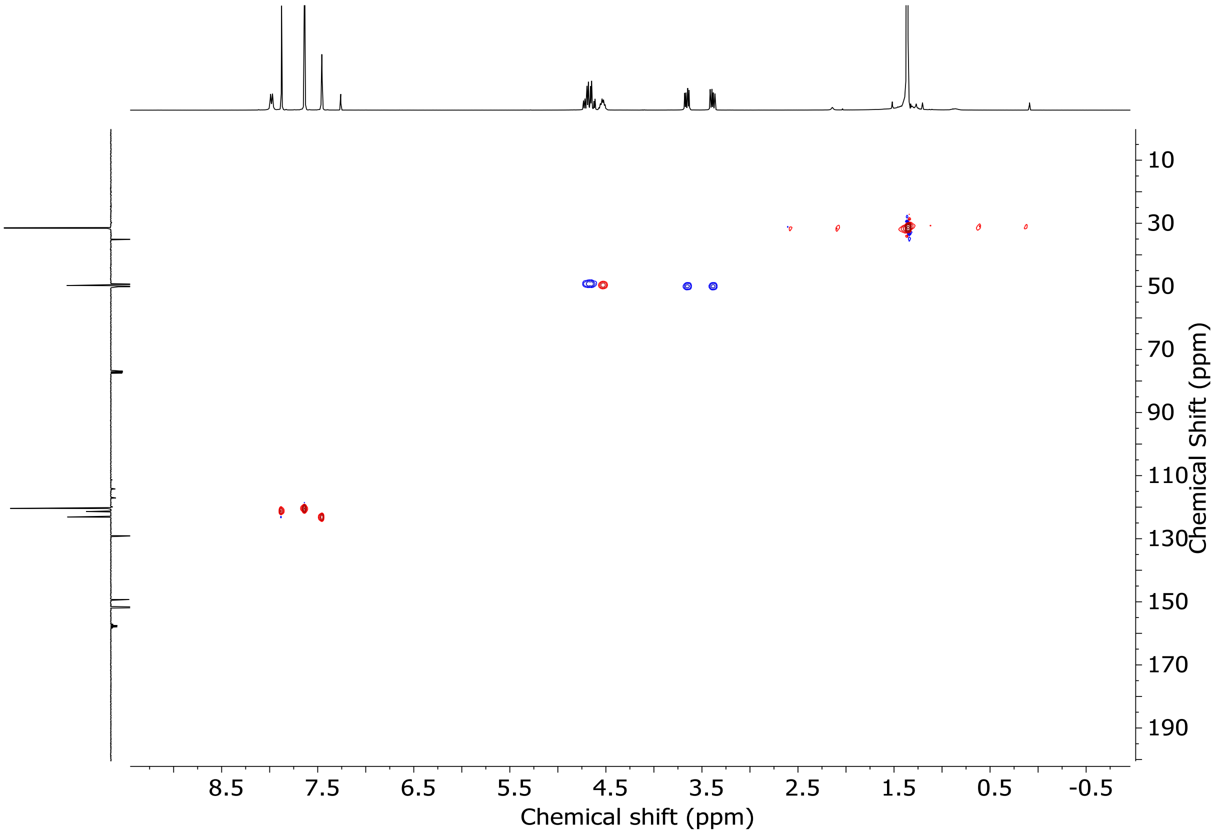

Figure S23. HSQC NMR ($CDCl_3$) of (*R*)-**1d**.

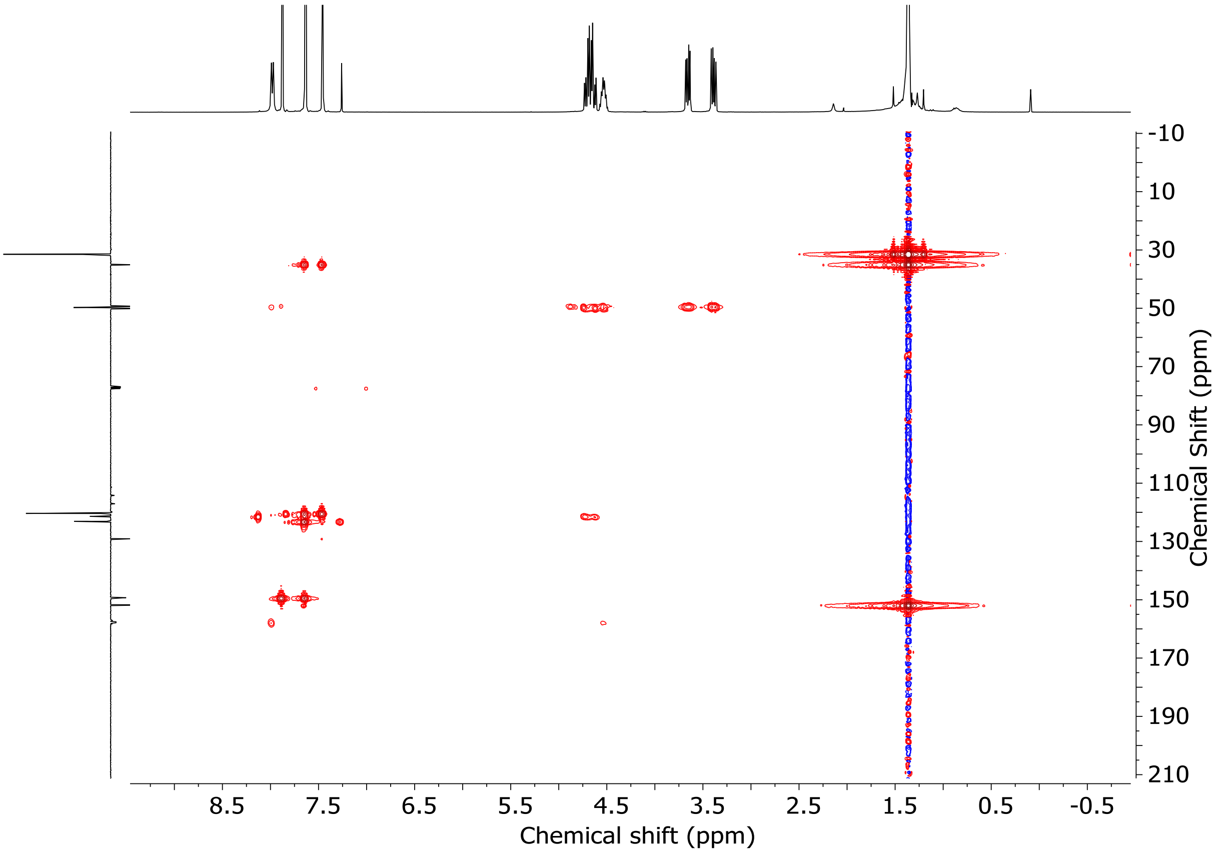

Figure S24. HMBC NMR ($CDCl_3$) of (*R*)-**1d**

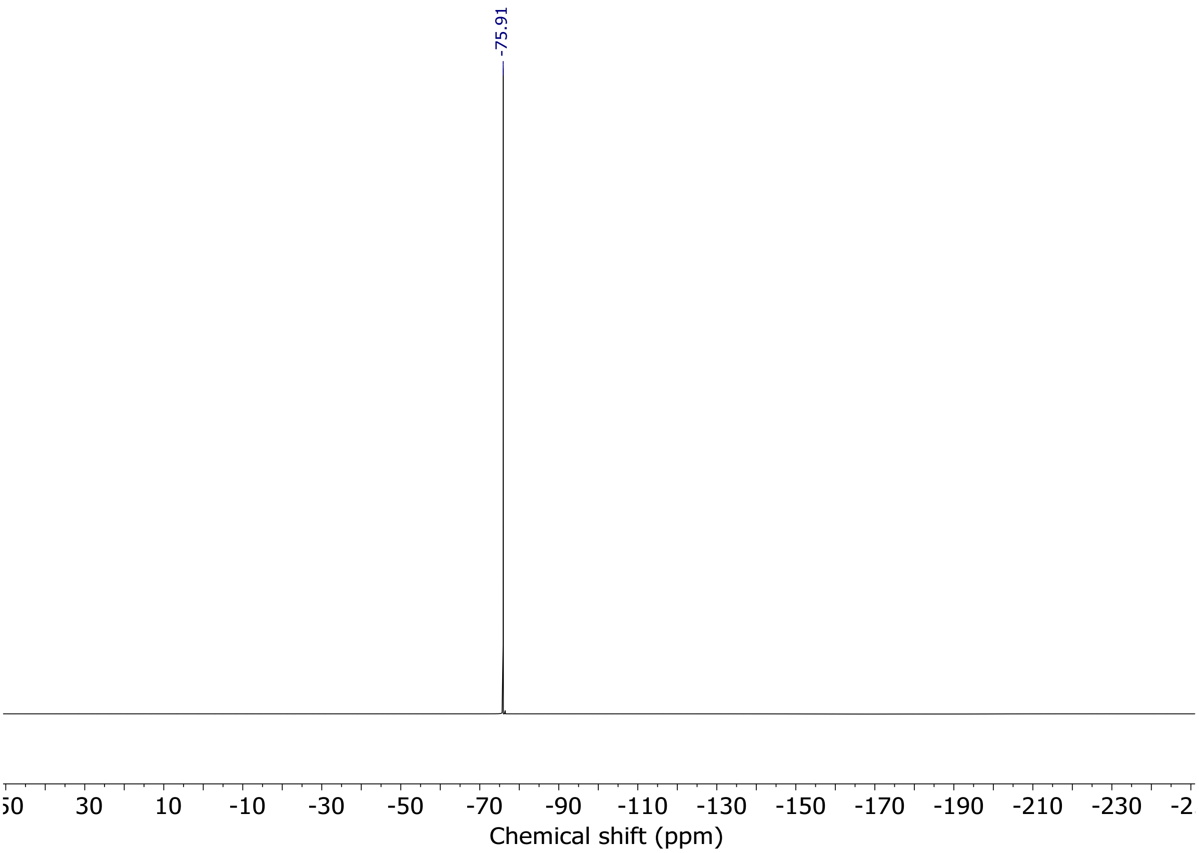

Figure S25. ^{19}F NMR ($CDCl_3$, 376 MHz) of (*R*)-**1d**.

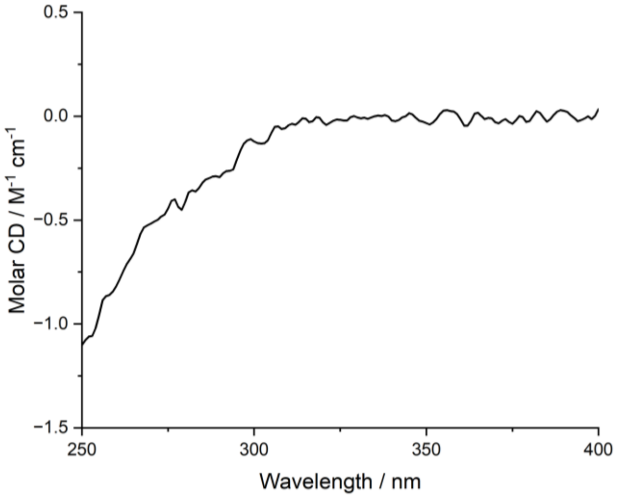

Figure S26. Circular Dichroism Spectra of (*R*)-**1d** (59 µM) at 293 K in $CHCl_3$.

Methylated Boc azide (*R*)-S4

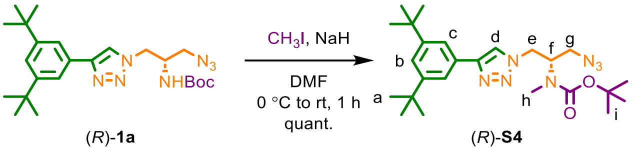

To a suspension of NaH (60% in mineral oil, 17.8 mg, 0.44 mmol) in DMF (2.5 mL) was added (*R*)-**1a** (100.0 mg, 0.22 mmol) in DMF (2.5 mL) dropwise at 0 °C. The reaction mixture was stirred for 10 minutes, then a solution of CH_3I (20 µL, 0.33 mmol) was added dropwise over 10 minutes. The reaction mixture was allowed to warm to rt and stirred for 1 h. The reaction mixture was then quenched by adding H_2O (10 mL) dropwise. The aqueous and organic phases were separated, and the aqueous phase was then extracted with Et_2O (3 x 20 mL). The combined organic extracts were washed with brine (10 mL), dried ($MgSO_4$) and concentrated *in vacuo*. Chromatography (petrol-Et_2O 5→10%) gave (*R*)-**S4** as a colourless oil (103 mg, quant.) as a mixture of rotamers.

^{1}H NMR (400 MHz, $CDCl_3$) δ: 7.81-7.58 (m, 6H, H_c major, H_c minor, H_d major, H_d minor), 7.41 (app t, 2H, H_b) 2.81 (s, 3H, H_h minor), 2.67 (s, 3H, H_h major), 1.48-1.38 (m, 18H, H_i major, H_i minor), 1.36 (app s, 36H, H_a major, H_a minor)

^{13}C NMR (101 MHz, $CDCl_3$) δ: 155.4, 151.4, 148.7, 129.6, 122.5, 120.4, 120.2, 120.1, 80.7, 58.8, 56.7, 50.5, 50.0, 49.2, 48.8, 34.9, 34.5, 31.5, 28.3.

$[\boldsymbol{a}]_D^{23}$-1.4 (c 0.62, $CHCl_3$)

HR-ESI-MS (+ve) m/z = 470.3252 $[M+H]^+$ (calc. 470.3243 m/z for $C_{25}H_{40}N_7O$);

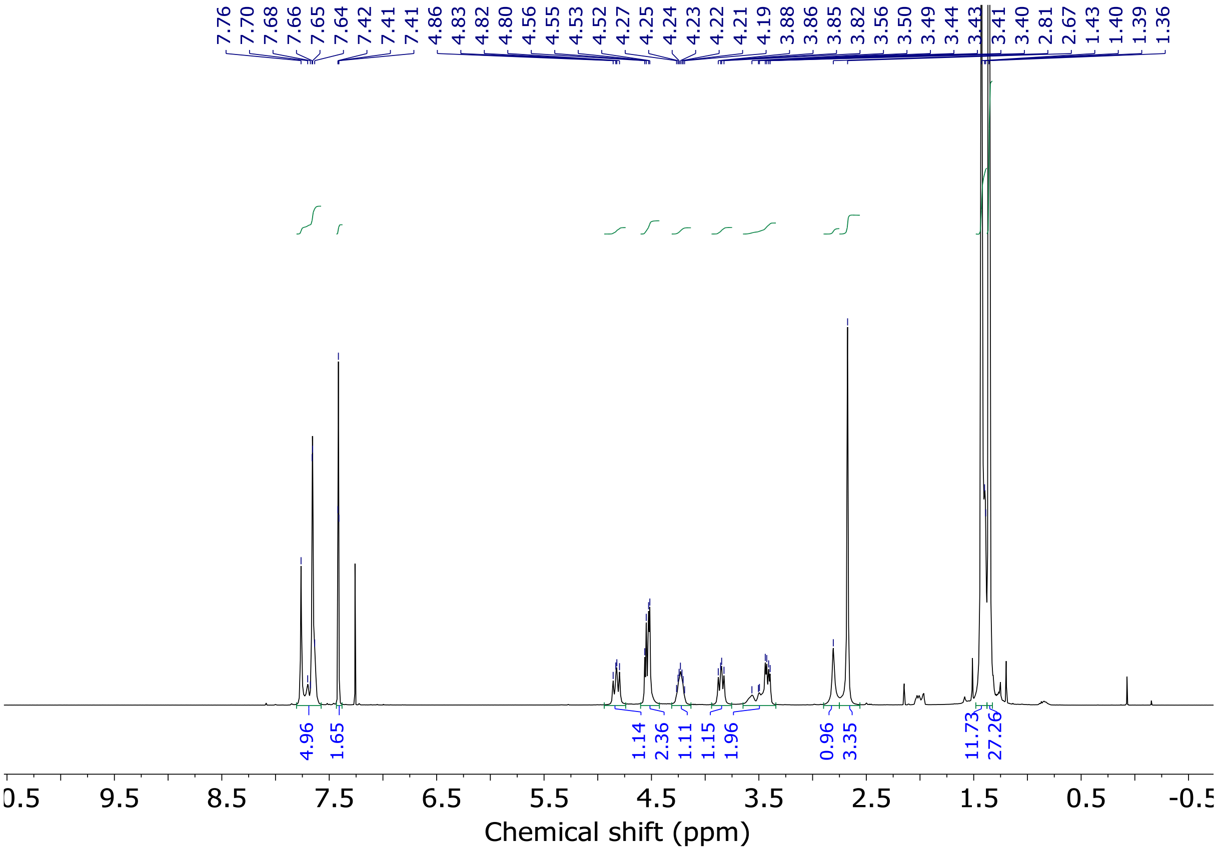

Figure S27. ^{1}H NMR ($CDCl_3$, 400 MHz) of (*R*)-**S4**.

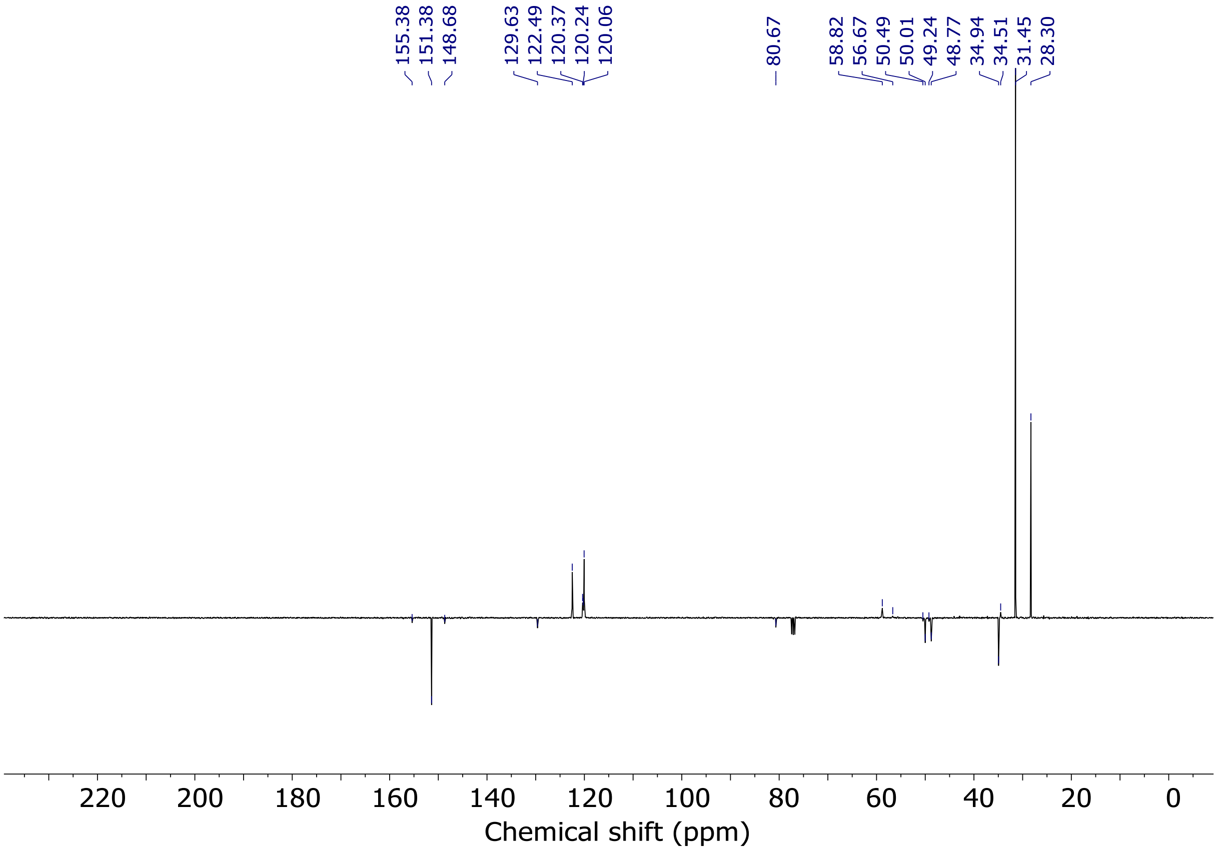

Figure S28. JMOD NMR ($CDCl_3$, 101 MHz) of (*R*)-**S4**.

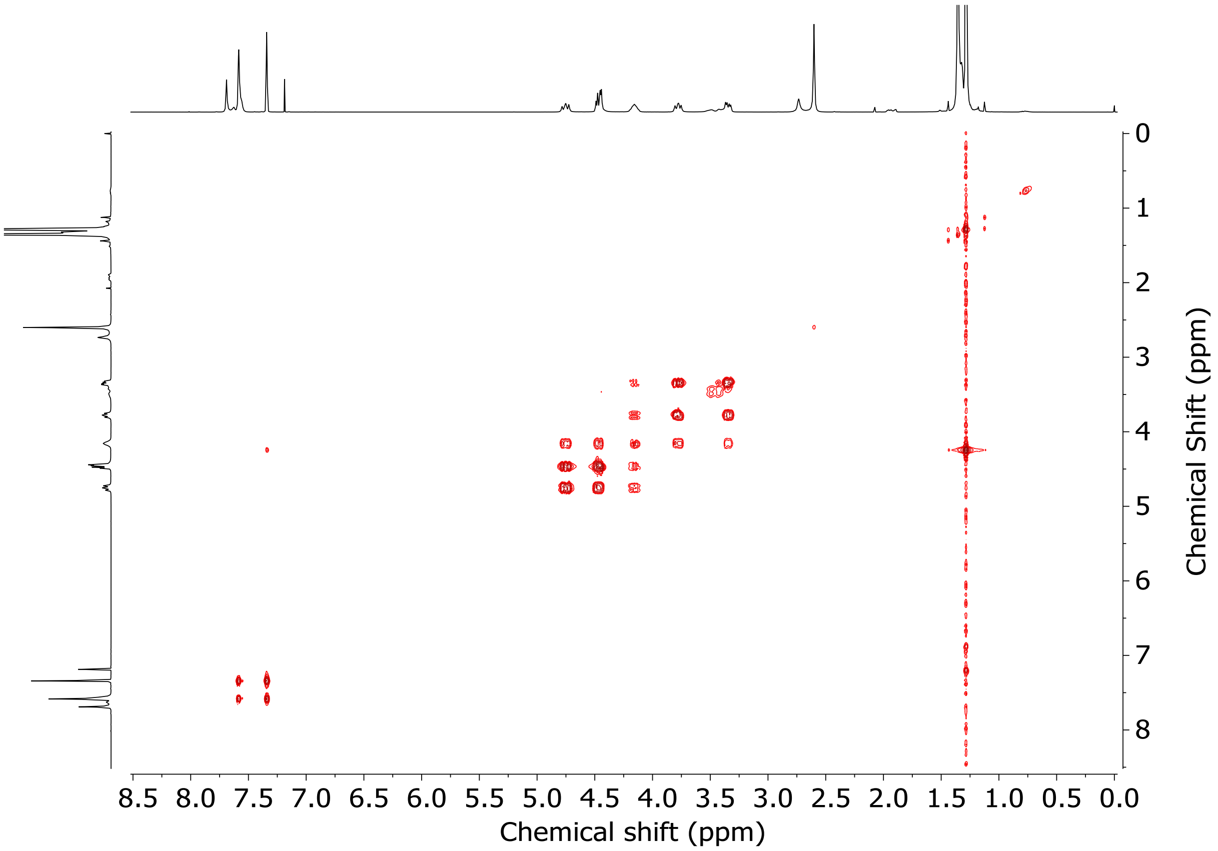

Figure S29. COSY NMR ($CDCl_3$) of (*R*)-**S4**.

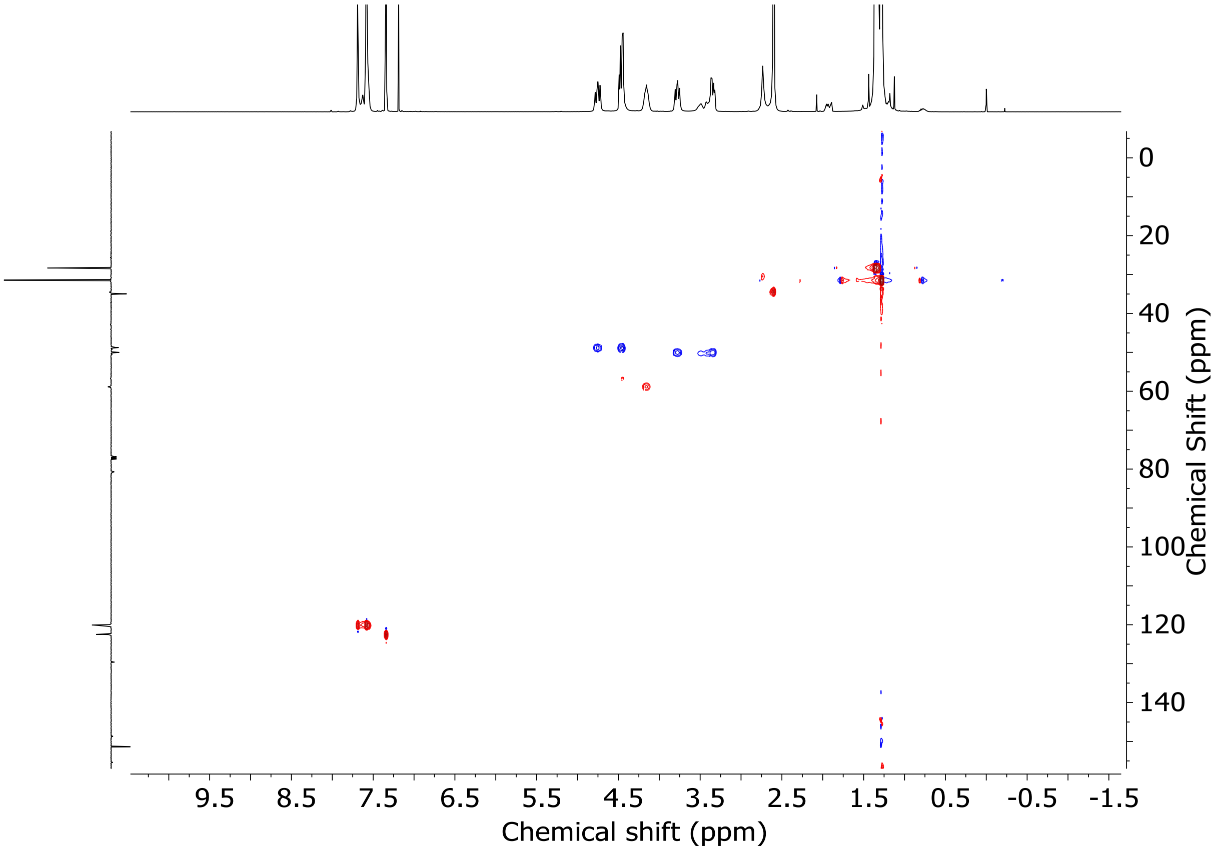

Figure S30. HSQC NMR ($CDCl_3$) of (*R*)-**S4**.

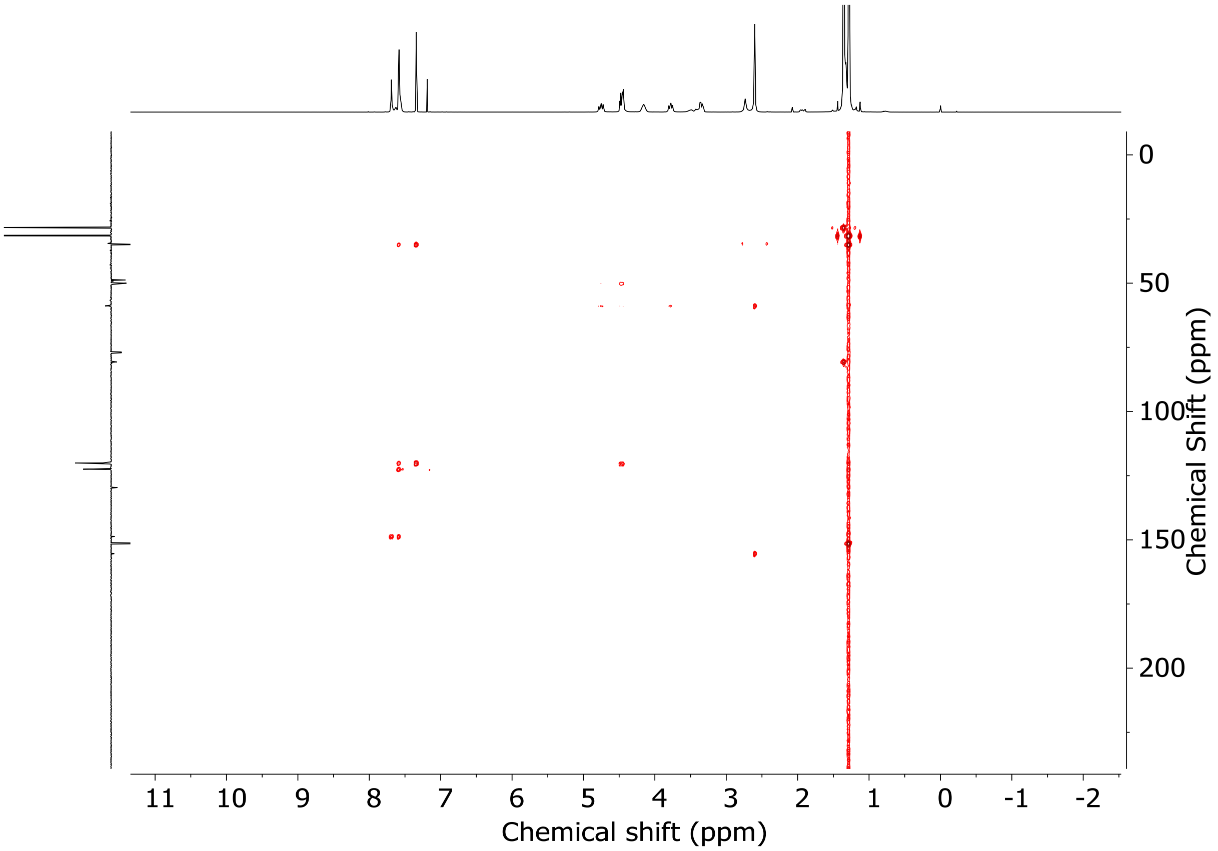

Figure S31. HMBC NMR ($CDCl_3$) of (*R*)-**S4**.

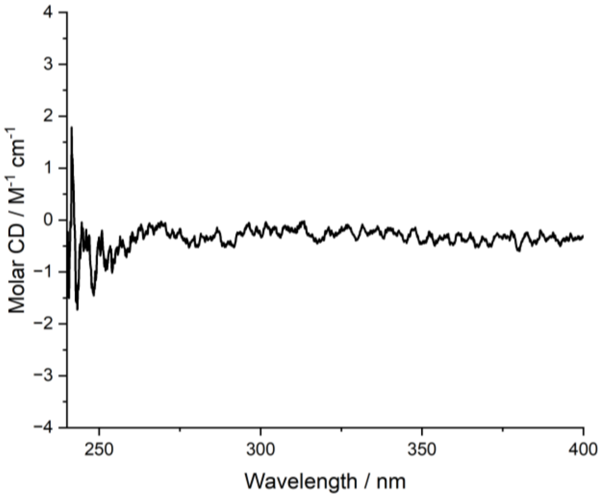

Figure S32. Circular Dichroism Spectra of (*R*)-**S4** (35 μM) at 293 K in $CHCl_3$. No measurable CD response was observed so the $[\alpha]_D$ of (*R*)-**S4** was measured.

Methylated amine azide (*R*)-S5

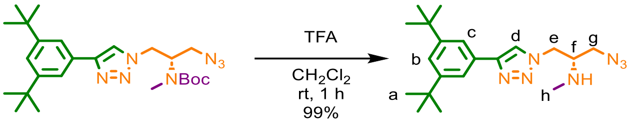

(*R*)-**S4** (*R*)-**S5**

To a solution of (*R*)-**S4** (103 mg, 0.22 mmol) in CH_2Cl_2 (2.2 mL) was added TFA (0.17 mL, 2.2 mmol). The solution was stirred at rt for 1 h. The solution was diluted with CH_2Cl_2 (5 mL) and poured carefully onto a saturated solution of $NaHCO_3$ (15 mL). The aqueous and organic phases were separated, and the aqueous phase was then extracted with CH_2Cl_2 (3 x 10 mL). The combined organic extracts were washed with brine (10 mL), dried ($MgSO_4$) and concentrated *in vacuo* to afford (*R*)-**S5** (81.2 mg, 99%) as a white solid without further purification.

^{1}H NMR (400 MHz, $CDCl_3$) δ: 7.83 (s, 1H, H$_d$), 7.68 (d, *J* = 1.9, 2H, H$_c$), 7.43 (t, *J* = 2.0, 1H, H$_b$), 4.53-4.40 (m, 2H, H$_e$), 3.44 (dd, *J* = 5.7, 12.7, 1H, H$_g$), 3.31 (dd, *J* = 5.0, 12.8, 1H, H$_{g'}$), 3.13 (app quint., *J* = 5.6, 1H, H$_f$), 2.50 (s, 3H, H$_h$), 1.37 (s, 18H, H$_a$)

^{13}C NMR (101 MHz, $CDCl_3$) δ: 151.4, 148.7, 129.6, 122.5, 120.8, 120.2, 59.0, 51.3, 50.7, 35.0, 34.0, 31.5.

$[\alpha]_D^{23}$ -0.8 (c 0.84, $CHCl_3$)

HR-ESI-MS (+ve) m/z = 370.2750 $[M+H]^+$ (calc. 370.2719 m/z for $C_{20}H_{32}N_7$);

Melting point 133-135°C.

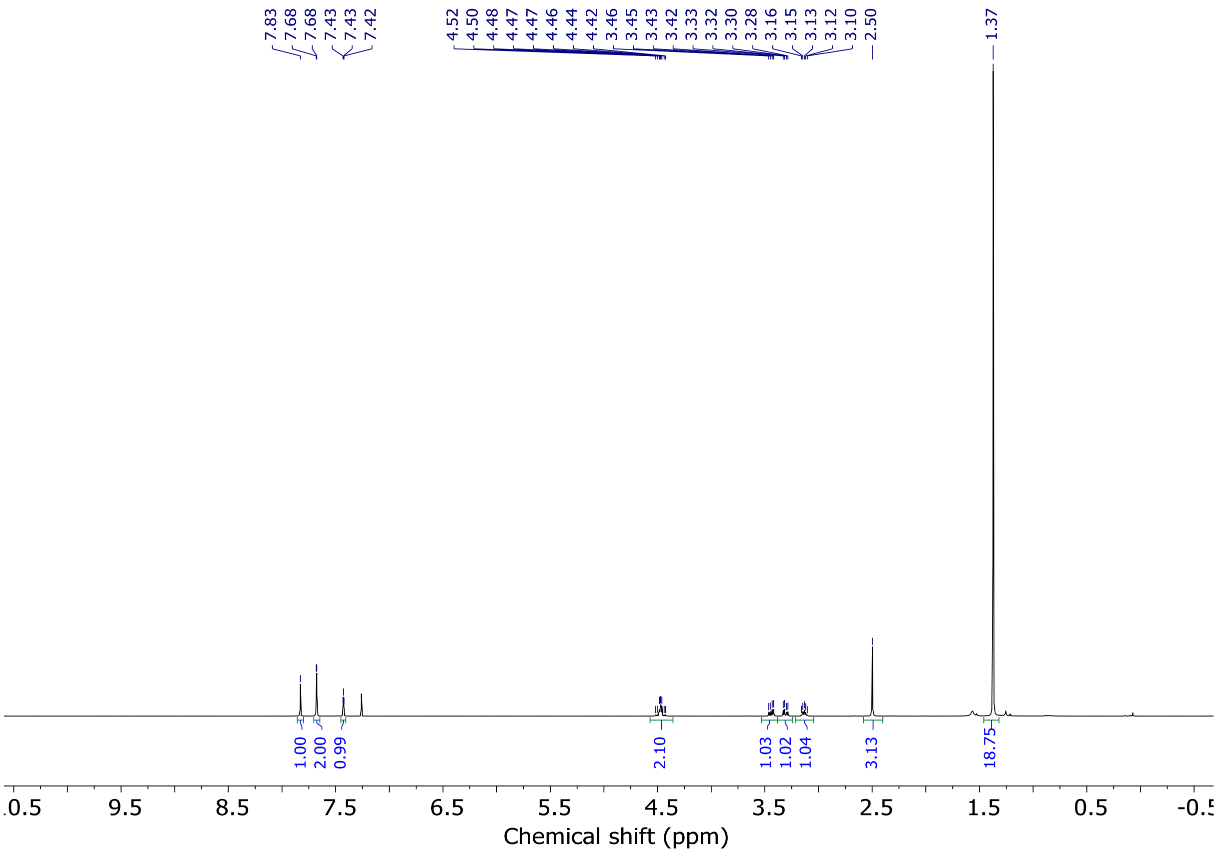

Figure S33. ^{1}H NMR ($CDCl_3$, 400 MHz) of (*R*)-**S5**.

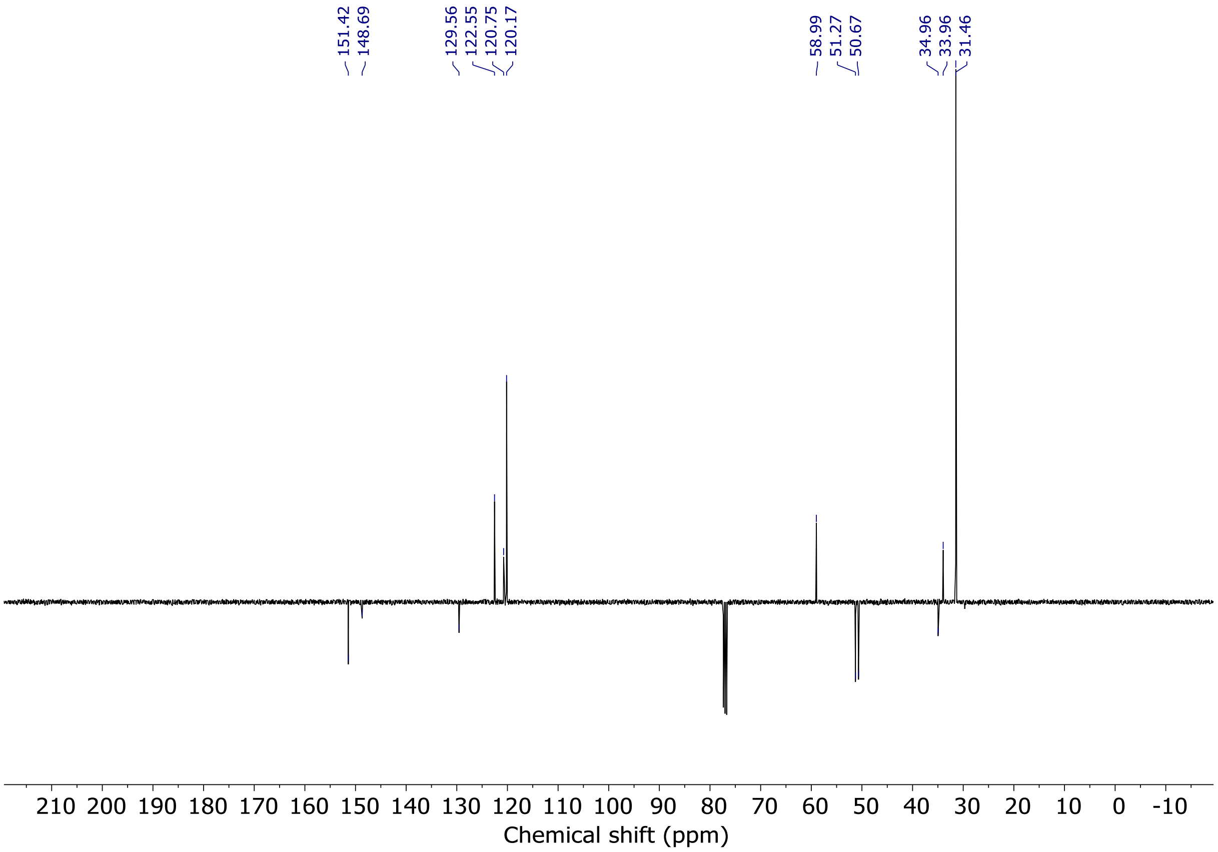

Figure S34. JMOD NMR ($CDCl_3$, 101 MHz) of (*R*)-**S5**.

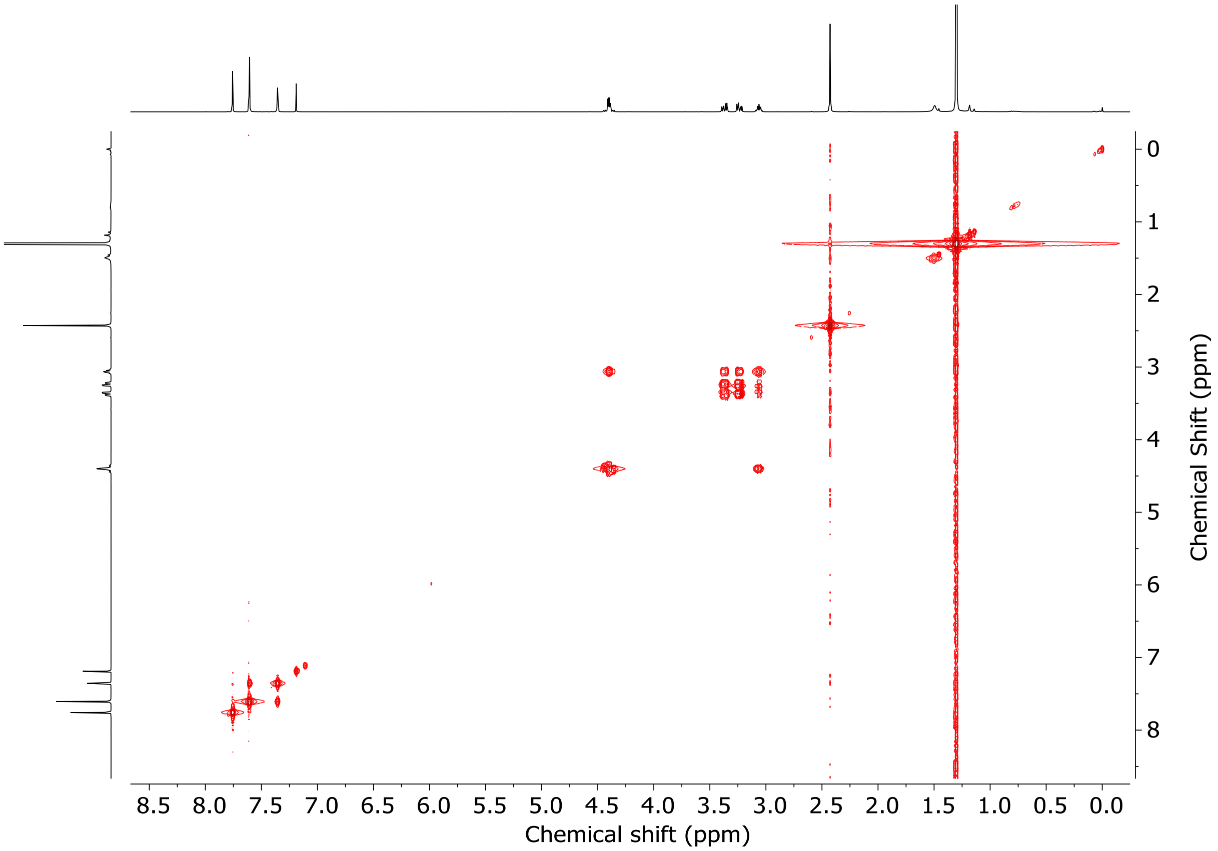

Figure S35. COSY NMR ($CDCl_3$) of (*R*)-**S5**.

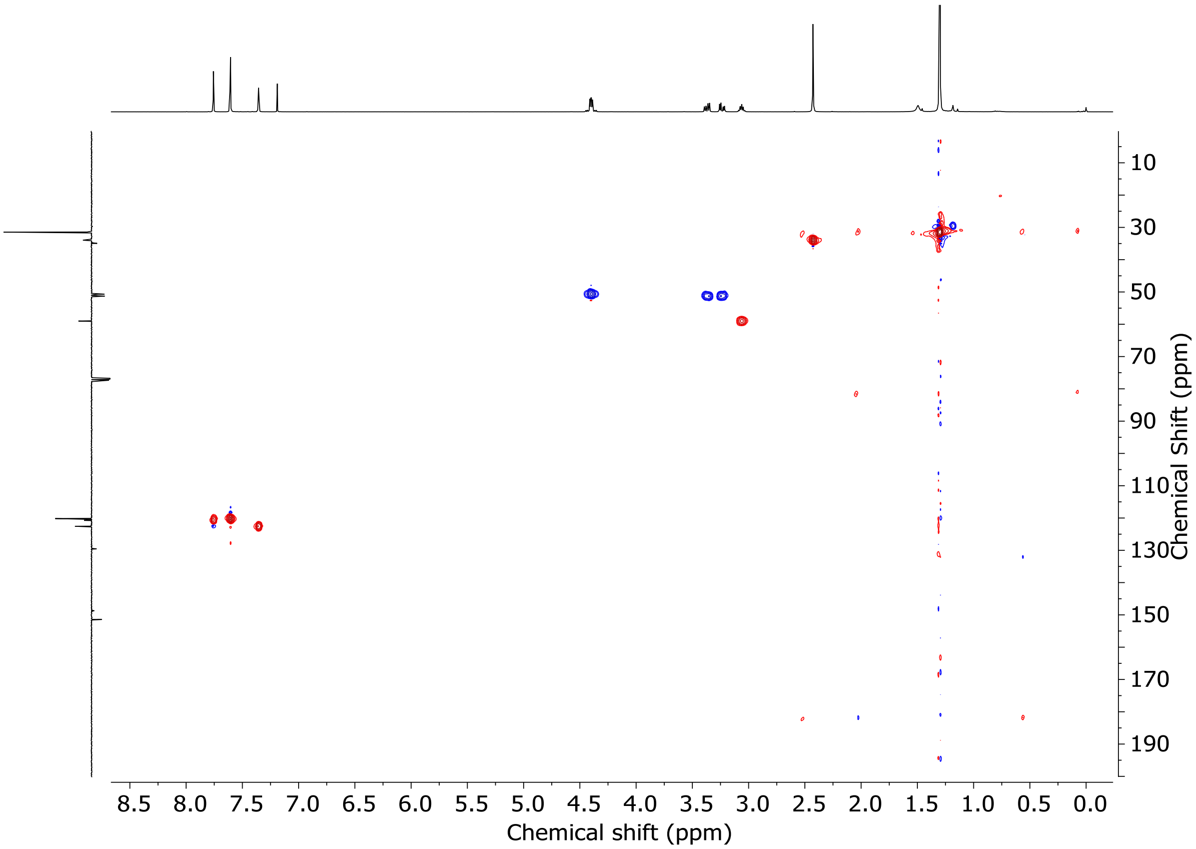

Figure S36. HSQC NMR ($CDCl_3$) of (*R*)-**S5**.

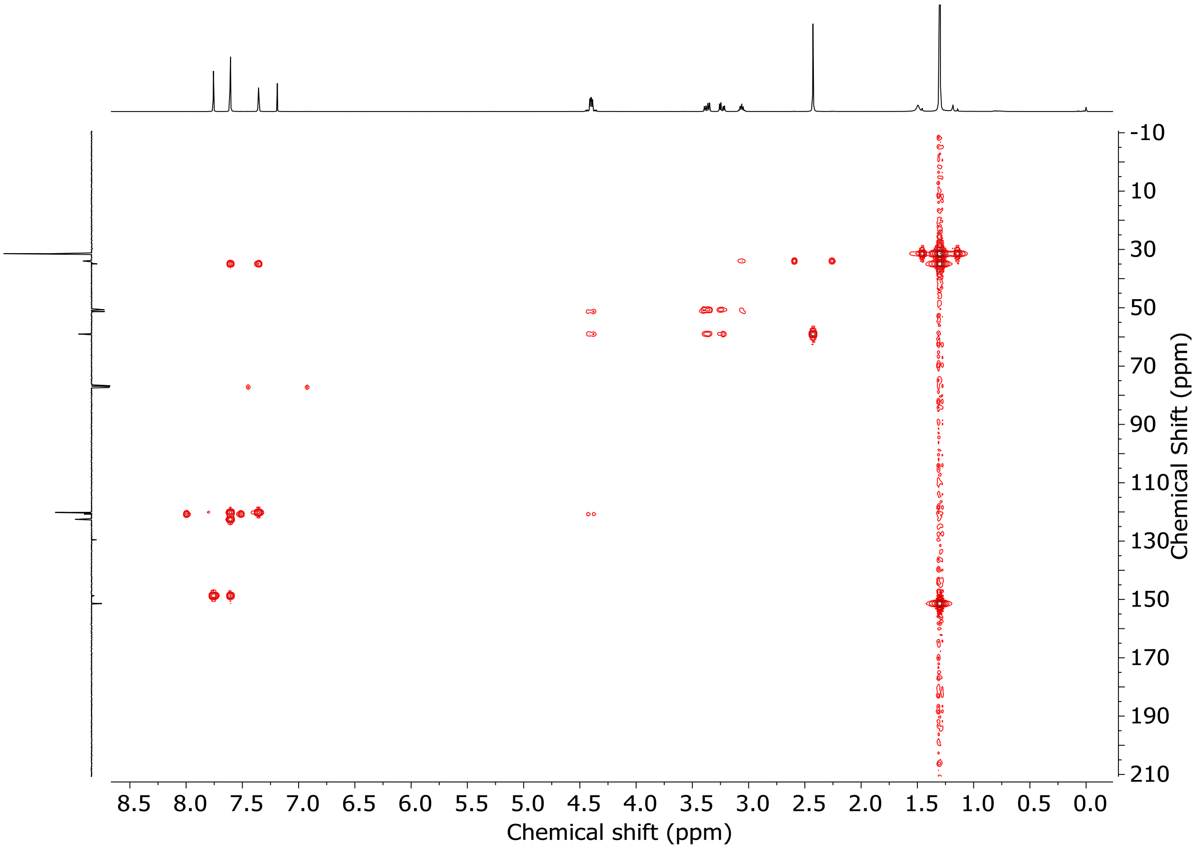

Figure S37. HMBC NMR ($CDCl_3$) of (*R*)-**S5**.

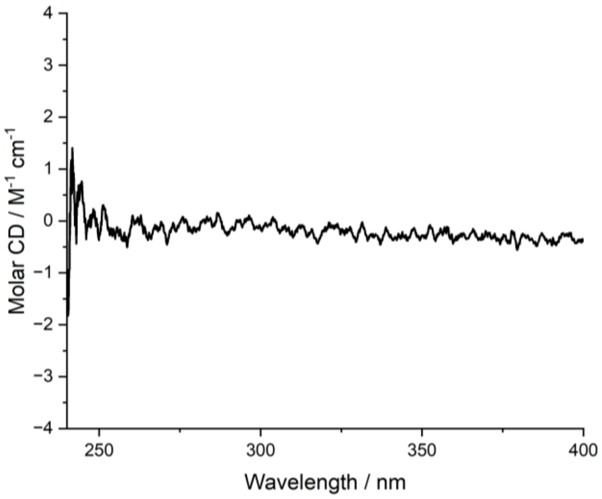

Figure S38. Circular Dichroism Spectra of (*R*)-**S5** (30 µM) at 293 K in $CHCl_3$. No measurable CD response was observed so the $[\alpha]_D$ of (*R*)-**S5** was measured.

Methylated trifluoroacetamide azide (*R*)-1e

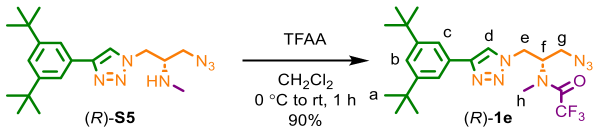

To a solution of (*R*)-**S5** (81.2 mg, 0.22 mmol) in CH_2Cl_2 (2.2 mL) was added trifluoroacetic anhydride (46.6 µL, 0.33 mmol) dropwise, then allowed to stir at rt for 1 h. The reaction mixture was then diluted with CH_2Cl_2 (5 mL) and poured slowly into sat. $NaHCO_{3(aq)}$ (10 mL). The aqueous and organic phases were separated, and the aqueous phase was then extracted with CH_2Cl_2 (3 x 10 mL). The combined organic extracts were washed with brine (15 mL), dried ($MgSO_4$) and concentrated *in vacuo*. Chromatography (petrol-EtOAc 0→100%) gave (*R*)-**1e** (92.1 mg, 90%) as a white foam.

^{1}H NMR (400 MHz, CDCl$_3$) δ: 7.73 (s, 1H, H_d), 7.65 (d, *J* = 1.9, 2H, H_c), 7.44 (t, *J* = 1.9, 1H, H_b), 4.93 (dd, *J* = 8.9, 14.2, 1H, H_e), 4.66 (dd, *J* = 5.8, 14.2, 1H, $H_{e'}$), 4.39 (tt, *J* = 9.2, 5.5, 1H, H_f), 4.01 (dd, *J* = 8.5, 12.6, 1H, H_g), 3.69 (dd, *J* = 5.6, 12.9, 1H, $H_{g'}$), 2.98 (s, 3H, H_h), 1.37 (s, 18H, H_a)

^{13}C NMR (101 MHz, CDCl$_3$) δ: 158.0 (app. d, J_{C-F} = 39.8) 157.9, 151.5, 149.2, 129.2, 122.8, 120.4, 120.2, 115.8 (app. d, J_{C-F} = 290.8), 61.0, 49.1, 47.7, 35.7, 35.0, 31.4.

^{19}F NMR (376 MHz, CDCl$_3$) δ -70.7

HR-ESI-MS (+ve) m/z = 466.2575 [M+H]$^+$ (calc. 466.2542 m/z for $C_{22}H_{30}F_3N_7O$);

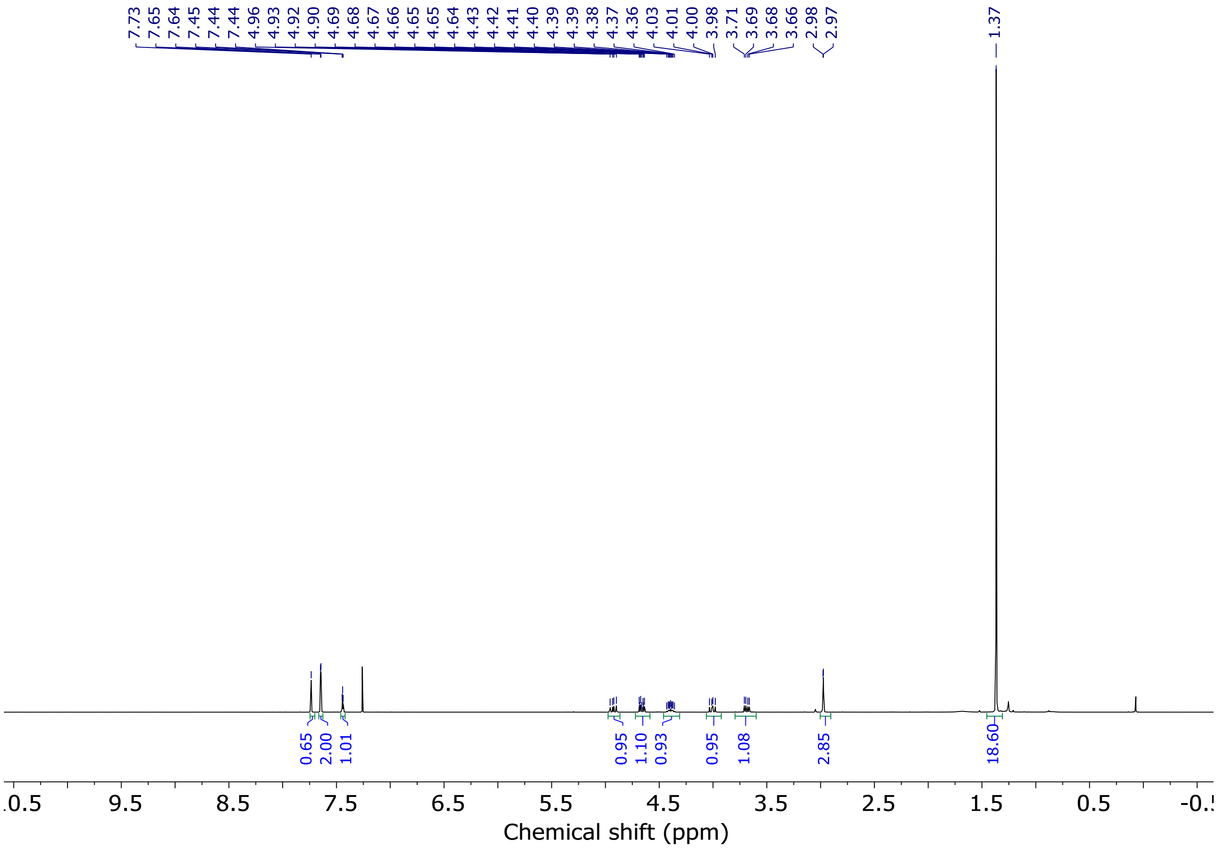

Figure S39. ^{1}H NMR ($CDCl_3$, 400 MHz) of (*R*)-**1e**.

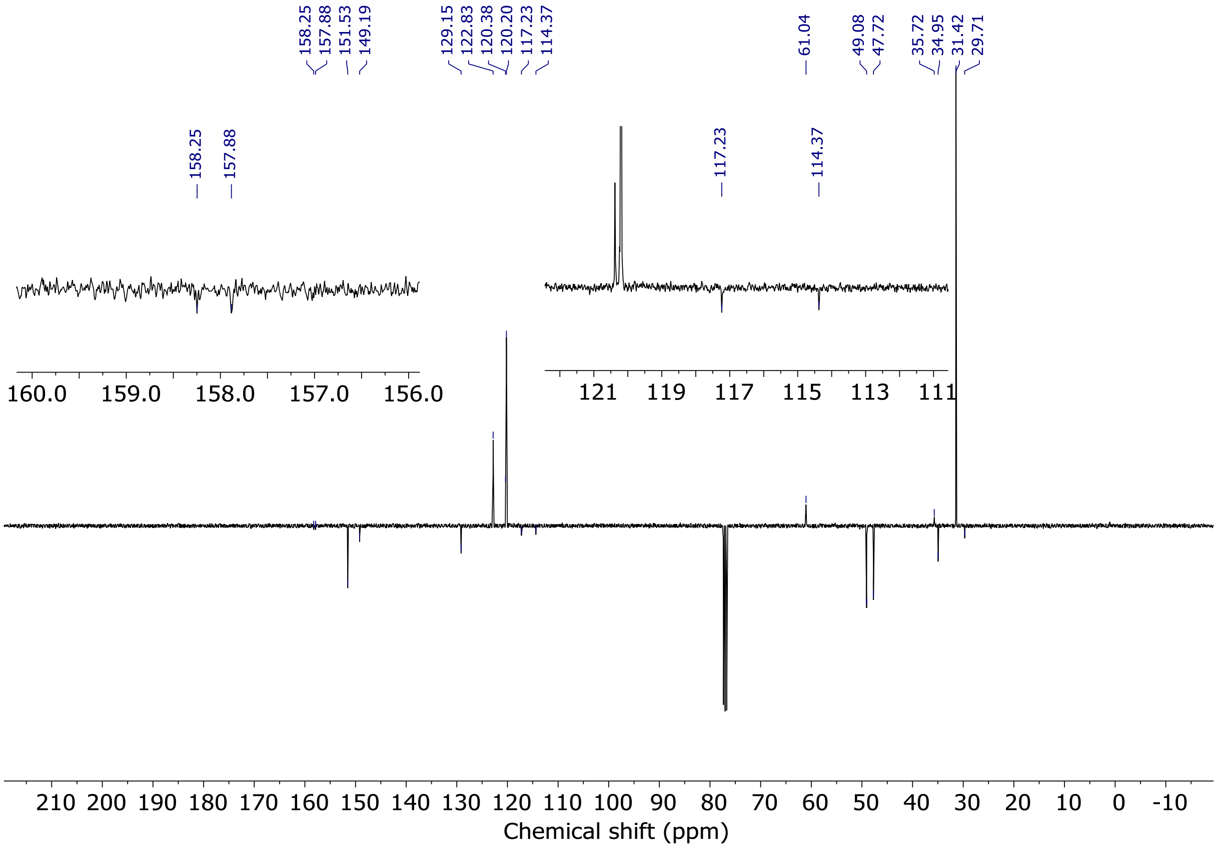

Figure S40. JMOD NMR ($CDCl_3$, 101 MHz) of (*R*)-**1e**.

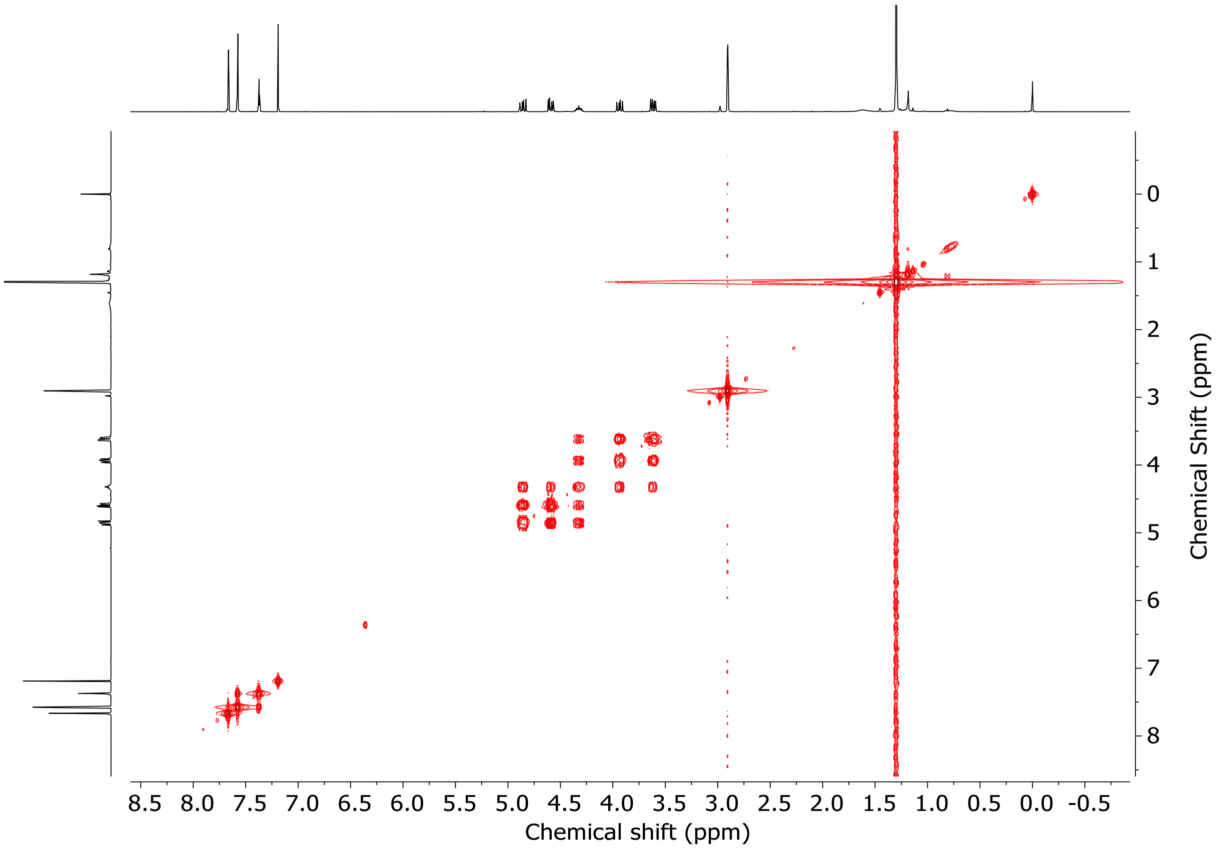

Figure S41. COSY NMR ($CDCl_3$) of (*R*)-**1e**.

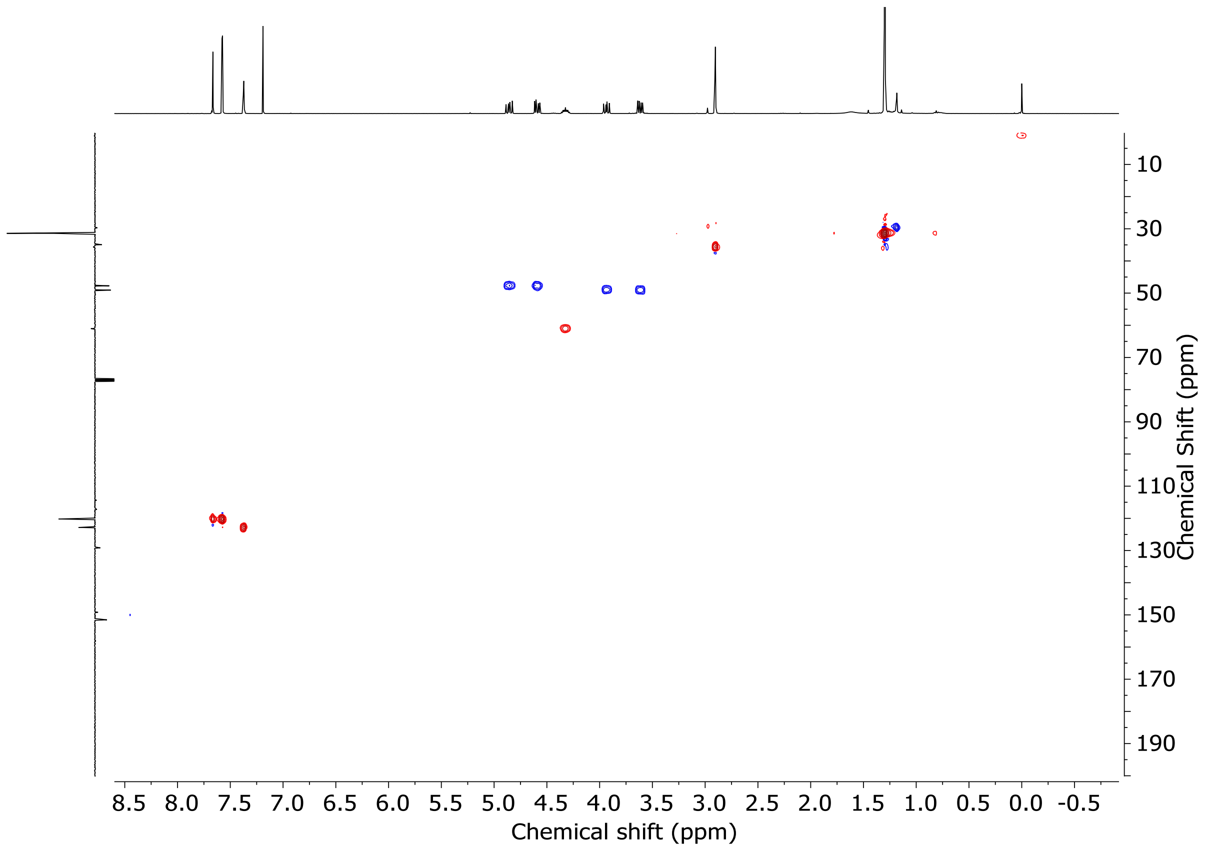

Figure S42. HSQC NMR ($CDCl_3$) of (*R*)-**1e**.

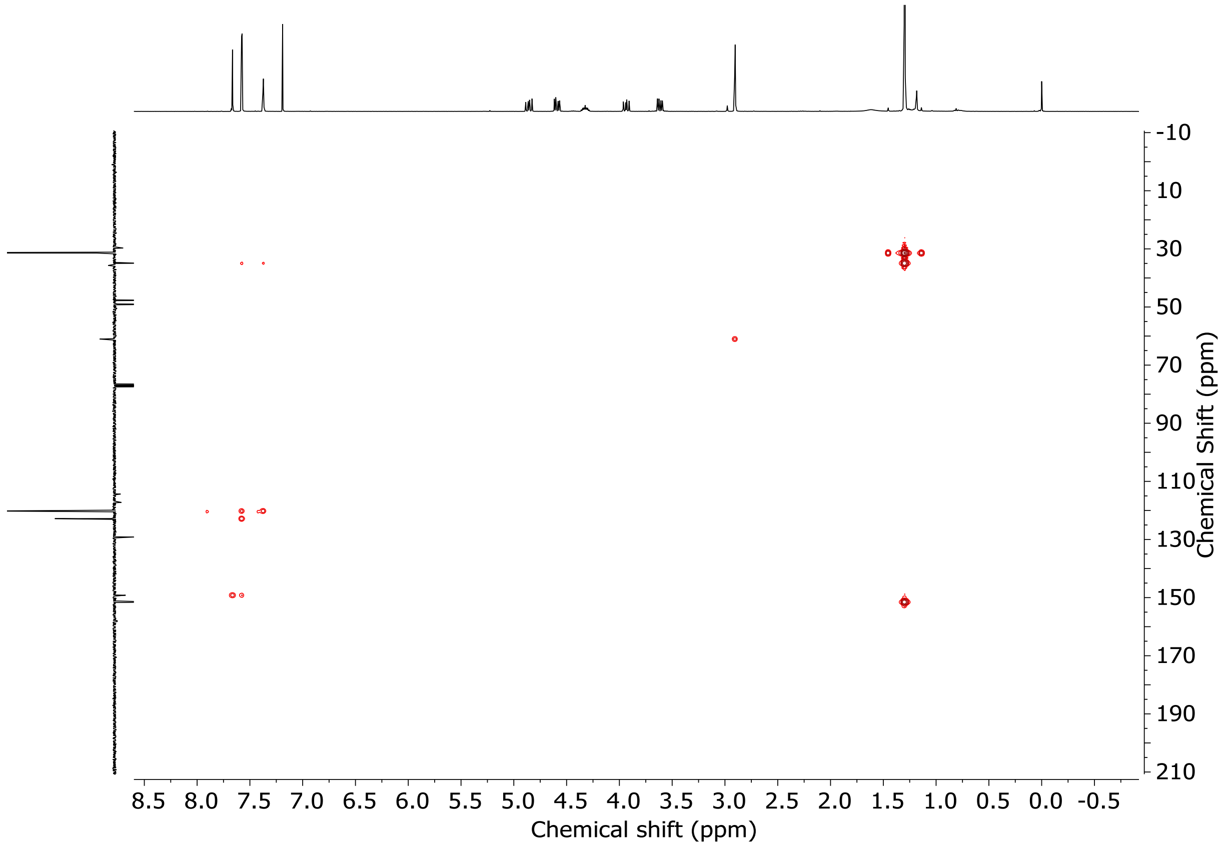

Figure S43. HMBC NMR (CDCl3) of (*R*)-**1e**.

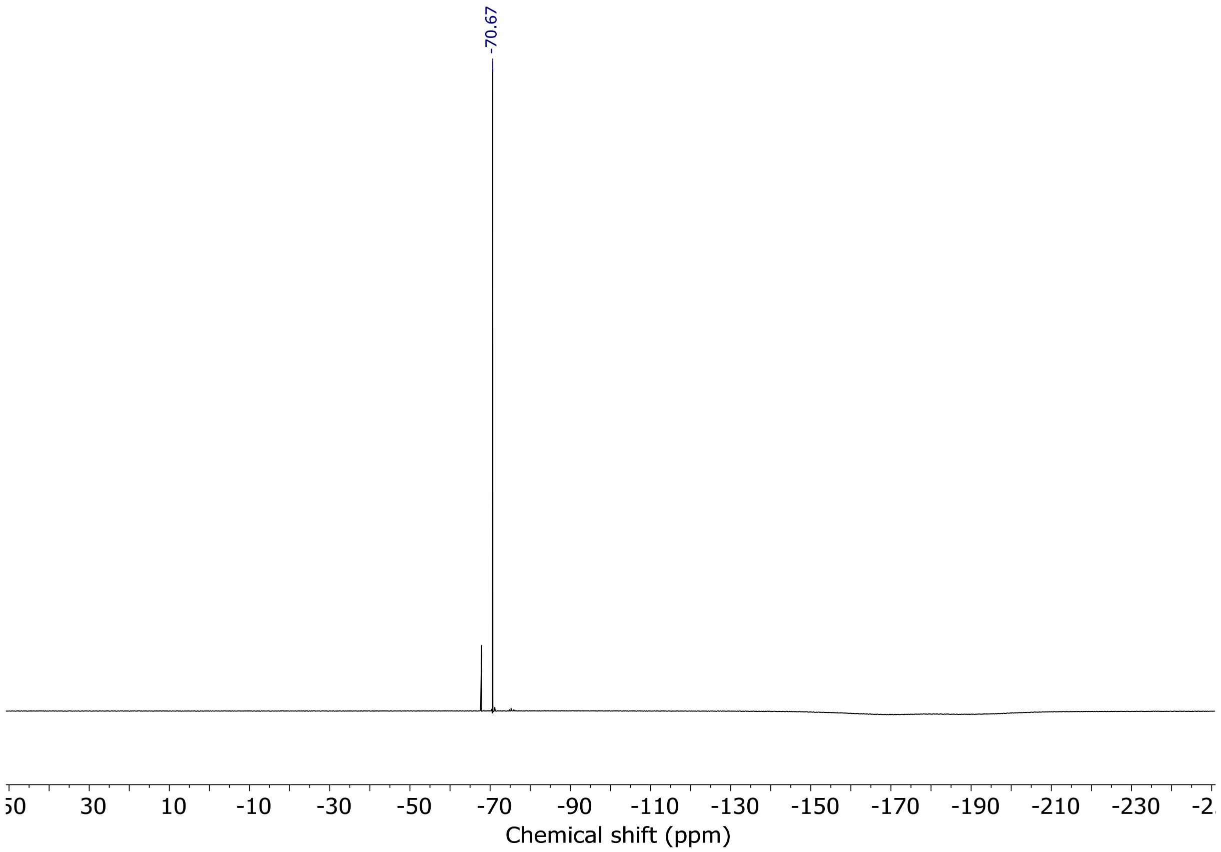

Figure S44. ^{19}F NMR ($CDCl_3$, 376 MHz) of (*R*)-**1e**.

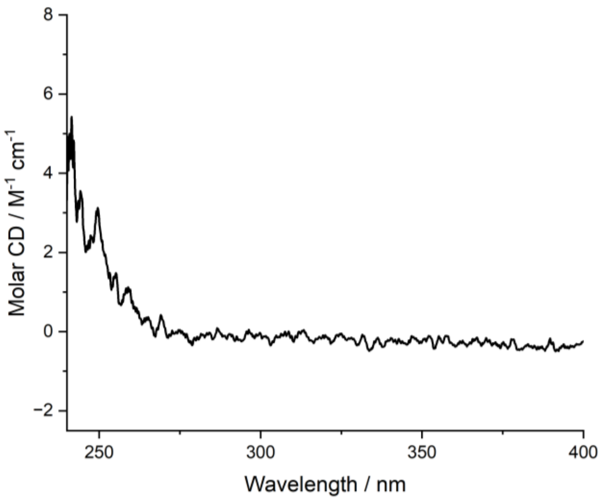

Figure S45. Circular Dichroism Spectra of (*R*)-**1e** (91 µM) at 293 K in $CHCl_3$.

Boc rotaxanes 4a

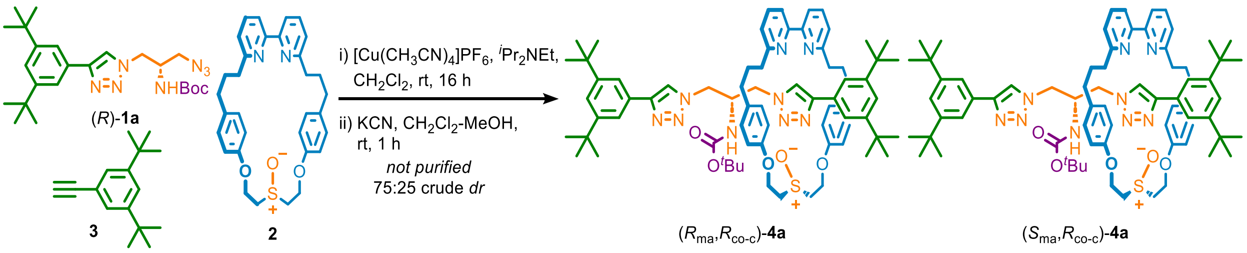

Using the previously reported procedure:[2]

In a CEM vial were added **3** (4.8 mg, 22.4 µmol), (*R*)-**1a** (10.2 mg, 22.4 µmol), **2** (10.5 mg, 19.9 µmol) and $[Cu(CH_3CN)_4PF_6]$ (7.2 mg, 19.3 µmol). The vial was sealed and purged with N_2, then CH_2Cl_2 was added (1.0 mL), followed by $^i Pr_2NEt$ (7.0 µL, 39.8 µmol). The solution was stirred at rt for 16 h. MeOH (1 mL) and KCN as a solid (13 mg, 0.20 mmol) were added and the resulting mixture was stirred vigorously for until colourless. The crude mixture was diluted with CH_2Cl_2 (5 mL) and washed with H_2O (5 mL) then EDTA-NH_3 (5 mL), with separation of aqueous and organic phases. The combined aqueous phase was then extracted with CH_2Cl_2 (3 x 5 mL) and the combined organic extracts were washed with brine (10 mL), dried ($MgSO_4$) and concentrated *in vacuo* to give a sample containing **4a** as a mixture of diastereomers (75 : 25 *dr*, Figure S46) that was analysed without further purification.

Characterisation data is identical to previously reported data.[2]

To demonstrate the role of the NHBoc unit as a barrier to co-conformational exchange, the mixture of diastereomers was dissolved in DMSO-d_6 and annealed at 100 °C for 16 h. No change in their ratio was observed, confirming that the macrocycle cannot shuttle between the two triazole containing compartments due to the steric bulk of the NHBoc unit (Figure S47).

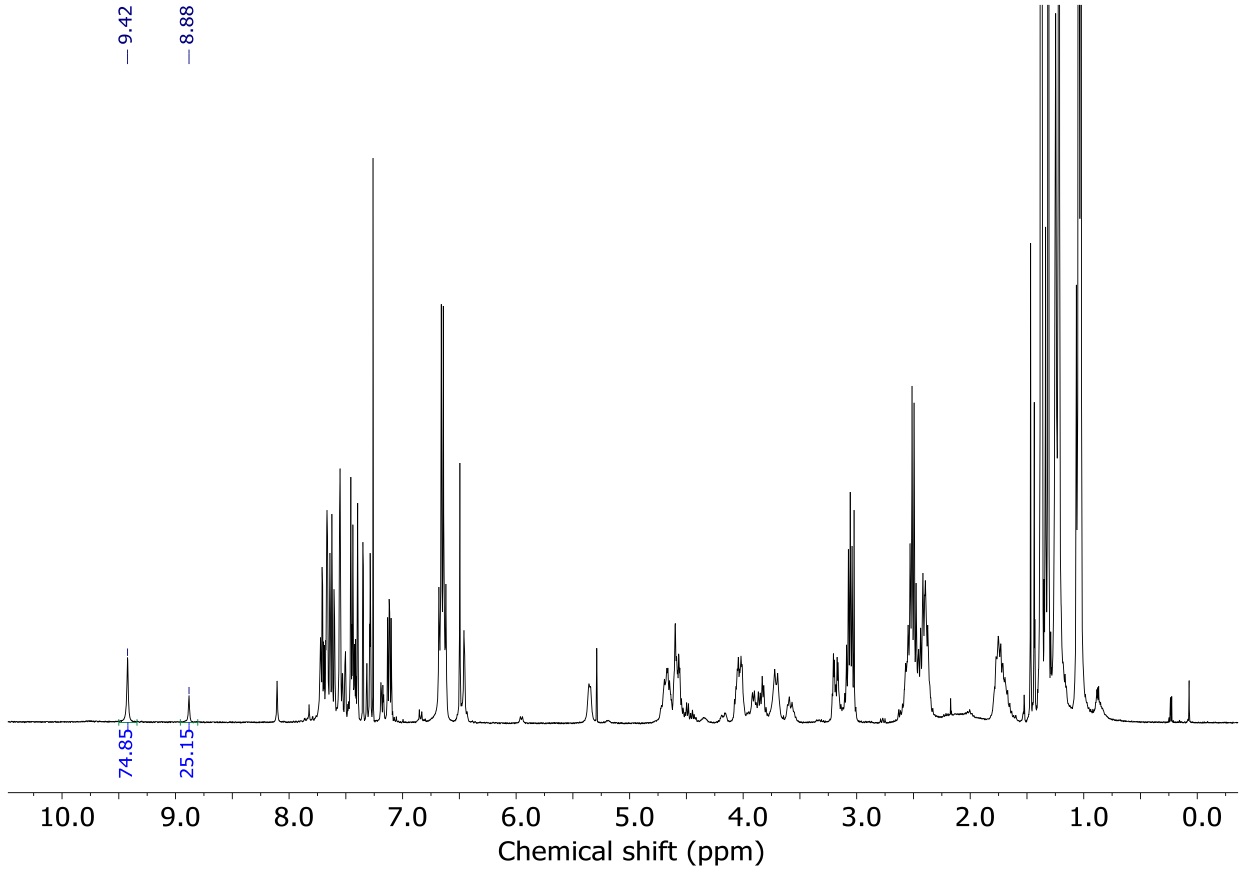

Figure S46. ^{1}H NMR ($CDCl_3$, 400 MHz) of (R_{ma},$R_{co\text{-}c}$)-**4a** and (S_{ma},$R_{co\text{-}c}$)-**4a** analysed without purification.

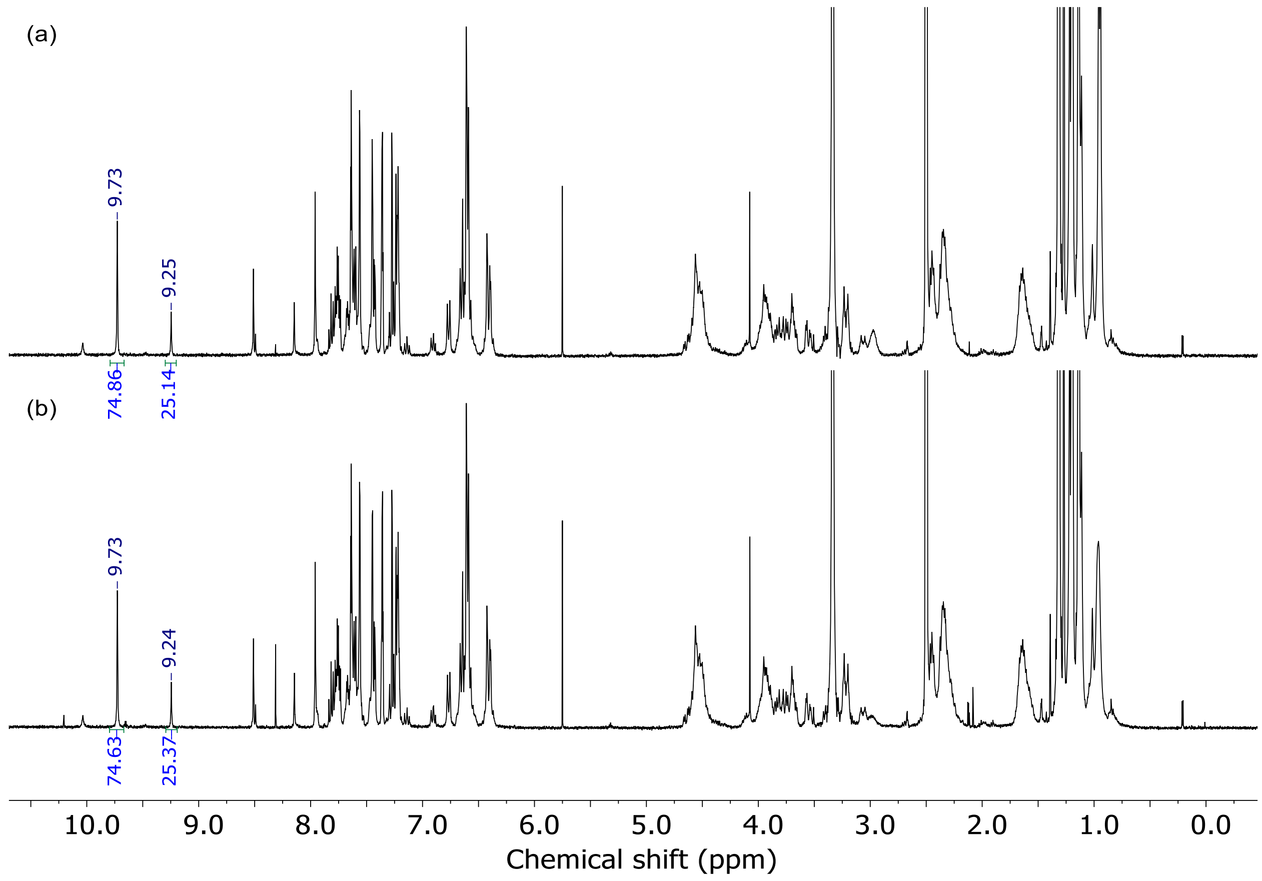

Figure S47. Stacked ^{1}H NMR (DMSO-d_6, 400 MHz) of (R_{ma},$R_{co\text{-}c}$)-**4a** and (S_{ma},$R_{co\text{-}c}$)-**4a**. (a) 75 : 25 *dr* mixture of diastereomers given from the AT-CuAAC synthesis of **4a** (b) 75 : 25 *dr* mixture of rotaxanes **4a** after heating at 100 °C for 16 h.

Acetamide rotaxanes 4b

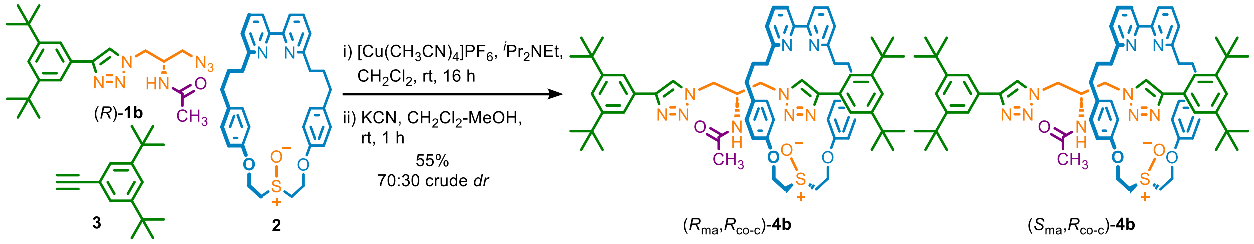

In a CEM vial were added **3** (9.4 mg, 41.7 µmol), (*R*)-**1b** (16.6 mg, 41.7 µmol), **2** (20.0 mg, 38.0 µmol) and [$Cu(CH_3CN)_4PF_6$] (13.6 mg, 36.0 µmol). The vial was sealed and purged with N_2, then CH_2Cl_2 was added (1.0 mL), followed by $^i Pr_2NEt$ (13.2 µL, 75.9 µmol). The solution was stirred at rt for 16 h. MeOH (2 mL mL) and KCN as a solid (24.7 mg, 0.38 mmol) were added and the resulting mixture was stirred vigorously for until colourless. The crude mixture was diluted with CH_2Cl_2 (5 mL) and washed with H_2O (5 mL) then EDTA-NH_3 (5 mL), with separation of aqueous and organic phases. The combined aqueous phase was then extracted with CH_2Cl_2 (3 x 5 mL) and the combined organic extracts were washed with brine (10 mL), dried ($MgSO_4$) and concentrated *in vacuo* to give a sample containing **4b** as a mixture of diastereomers (70 : 30 *dr*, Figure S48). Chromatography (CH_2Cl_2-CH_3CN 0→100%) gave **4b** as a white foam (23.8 mg, 55%) as a mixture of diatereomers (2.2 : 1, Figure S49).

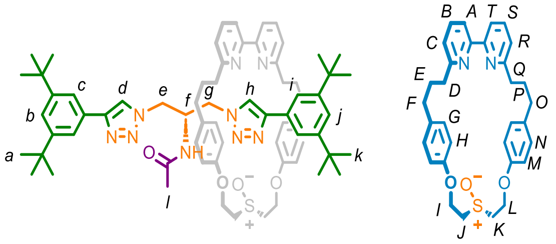

Major diastereoisomer

^{1}H NMR (400 MHz, CDCl$_3$) δ: 9.61 (s, 1H, H_h), 7.73-7.56 (m, 6H, H_B, H_b, H_c, H_d, H_S), 7.52 (d, *J* = 1.8, 2H, H_i), 7.43-7.37 (m, 2H, H_A, H_T), 7.27 (t, *J* = 1.8, 1H, H_j), 7.13-7.07 (m, 2H, H_C, H_R), 6.75 (d, *J* = 8.7, H_G or H_N), 6.71-6.61 (m, 7H, H_G or H_N, H_H, H_M, NH), 4.80-4.42 (m, 4H, H_I, H_L), 4.14-3.87 (m, 4H, H_e or H_g, H_f, H_J, H_K), 3.85-3.62 (m, 2H, H_e or H_g), 3.57 (dd, *J* = 14.5, 4.1, 1H, $H_{e'}$ or $H_{g'}$), 3.19-3.00 (m, 2H, $H_{J'}$, $H_{K'}$), 2.66-2.23 (m, 8H, H_D, H_F, H_O, H_Q), 1.90-1.61 (m, 4H, H_E, H_P superimposed with H_2O), 1.52 (s, 3H, H_l), 1.36 (s, 18H, H_a or H_k), 1.21 (s, 18H, H_a or H_k)

^{13}C NMR (101 MHz, CDCl$_3$) δ: 170.6, 162.9, 162.8, 157.4, 157.1, 156.0, 155.9, 151.2, 151.2, 150.7, 148.2, 147.5, 137.1, 137.0, 133.6, 133.4, 130.4, 129.8, 129.7, 129.4, 128.9, 122.2, 122.0, 121.5, 121.5, 120.5, 120.4, 120.4, 120.1, 119.8, 114.6, 114.5, 60.7, 60.6, 53.5, 53.3, 50.2, 50.0, 49.7, 37.0, 36.7, 34.5, 34.5, 31.6, 31.6, 31.5, 30.6, 30.0, 29.7.

HR-ESI-MS (+ve) m/z = 1138.7 $[M+H]^+$ (calc. m/z for $C_{69}H_{87}N_9O_4S$ 1138.6680);

Minor diastereoisomer

^{1}H NMR (400 MHz, CDCl$_3$) δ: 9.29 (s, 1H, H$_h$), 7.73-7.56 (m, 3H, H$_B$, H$_d$, H$_S$), 7.54 (d, J = 1.9, 2H, H$_i$), 7.54-7.47 (m, 2H, H$_A$, H$_T$), 7.43-7.37 (m, 3H, H$_b$, H$_c$), 7.30 (t, J = 1.9, 1H, H$_j$), 7.10-7.13 (m, 2H, H$_C$, H$_R$), 6.72-6.61 (m, 1H, NH), 6.62-6.50 (m, 8H, H$_G$, H$_H$, H$_M$, H$_N$), 4.80-4.42 (m, 4H, H$_I$, H$_L$), 4.14-3.87 (m, 3H, H$_e$ or H$_g$, H$_f$), 3.85-3.62 (m, 4H, H$_e$ or H$_g$, H$_J$, H$_K$), 3.45-3.25 (m, 2H, H$_{J'}$, H$_{K'}$), 2.66-2.23 (m, 8H, H$_D$, H$_F$, H$_O$, H$_Q$), 1.90-1.61 (m, 4H, H$_E$, H$_P$ superimposed with H_2O), 1.48 (s, 3H, H$_l$), 1.38 (s, 18H, H$_a$ or H$_k$), 1.27 (s, 18H, H$_a$ or H$_k$)

^{13}C NMR (101 MHz, CDCl$_3$) δ: 170.5, 162.9, 162.8, 157.6, 157.4, 155.4, 155.3, 151.4, 150.7, 148.4, 147.7, 137.3, 137.2, 133.5, 133.4, 130.2, 129.6, 128.9, 123.9, 123.2, 122.5, 122.3, 122.0, 121.4, 120.7, 120.5, 120.1, 120.0, 115.1, 115.0, 61.6, 61.1, 51.8, 51.8, 49.8, 49.8, 49.6, 37.2, 34.9, 34.9, 34.8, 31.4.

HR-ESI-MS (+ve) m/z = 1138.6711 [M+H]$^+$ (calc. m/z for $C_{69}H_{87}N_9O_4S$);

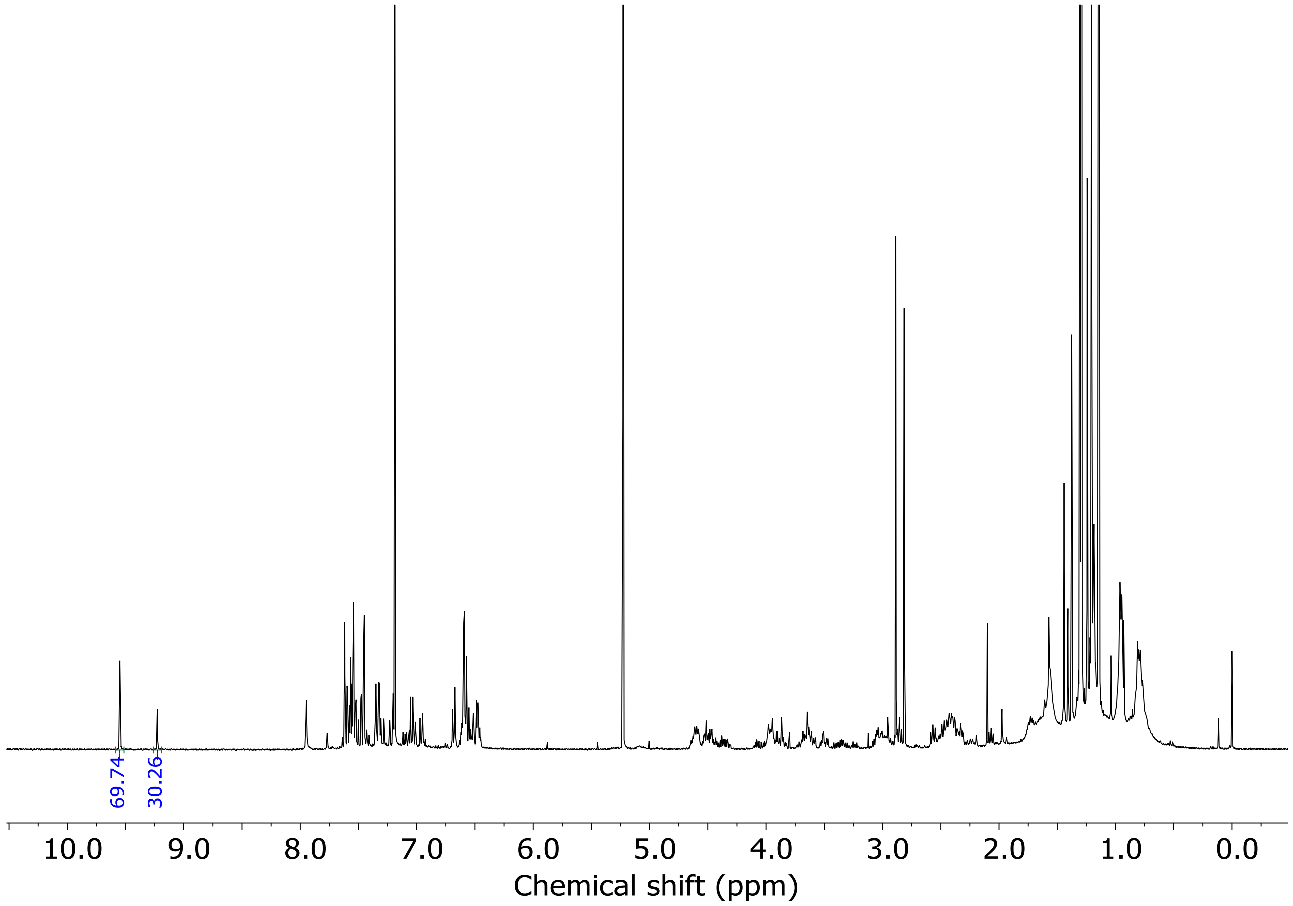

Figure S48. ^{1}H NMR (CDCl$_3$, 400 MHz) of (R_{ma},$R_{co\text{-}c}$)-**4b** and (S_{ma},$R_{co\text{-}c}$)-**4b** prior to purification (70 : 30 *dr*).

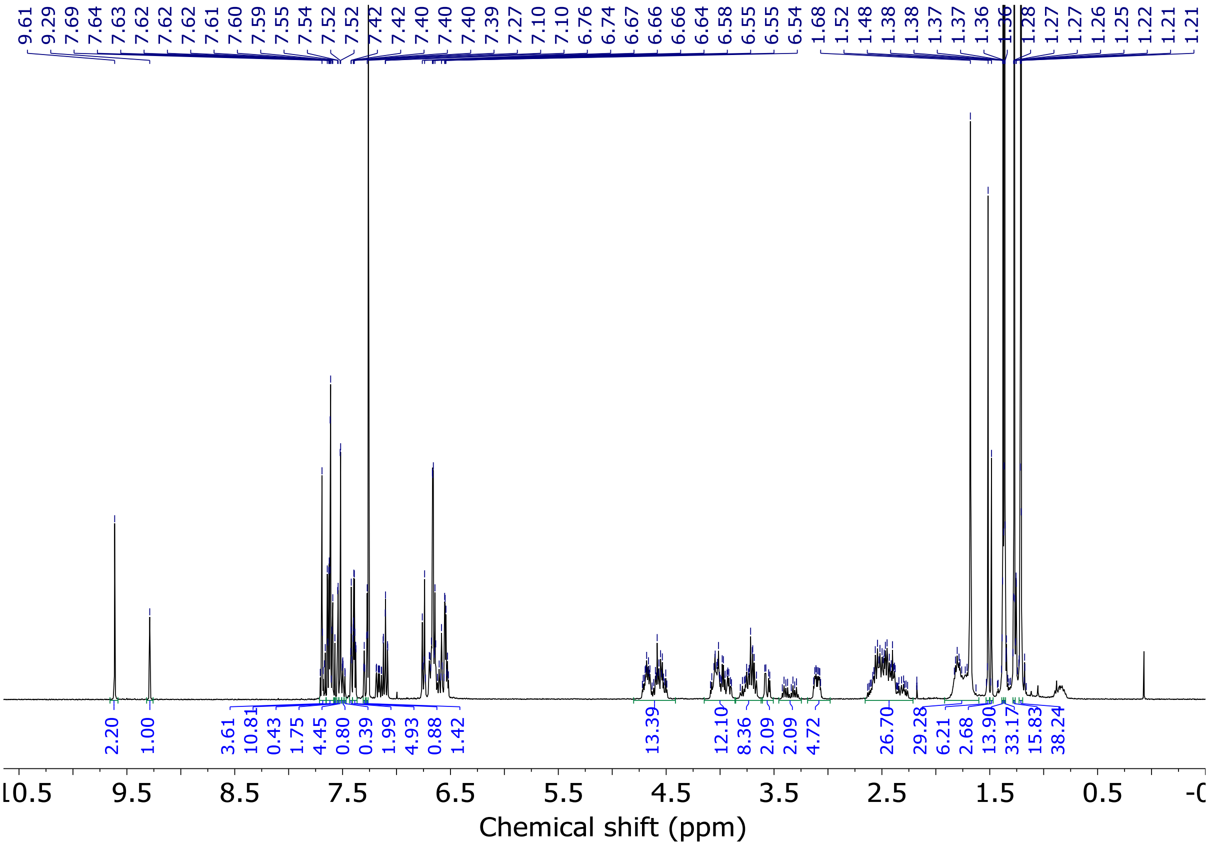

Figure S49. ^{1}H NMR ($CDCl_3$, 400 MHz) of (R_{ma},$R_{co\text{-}c}$)-**4b** and (S_{ma},$R_{co\text{-}c}$)-**4b** (2.2 : 1 *dr*).

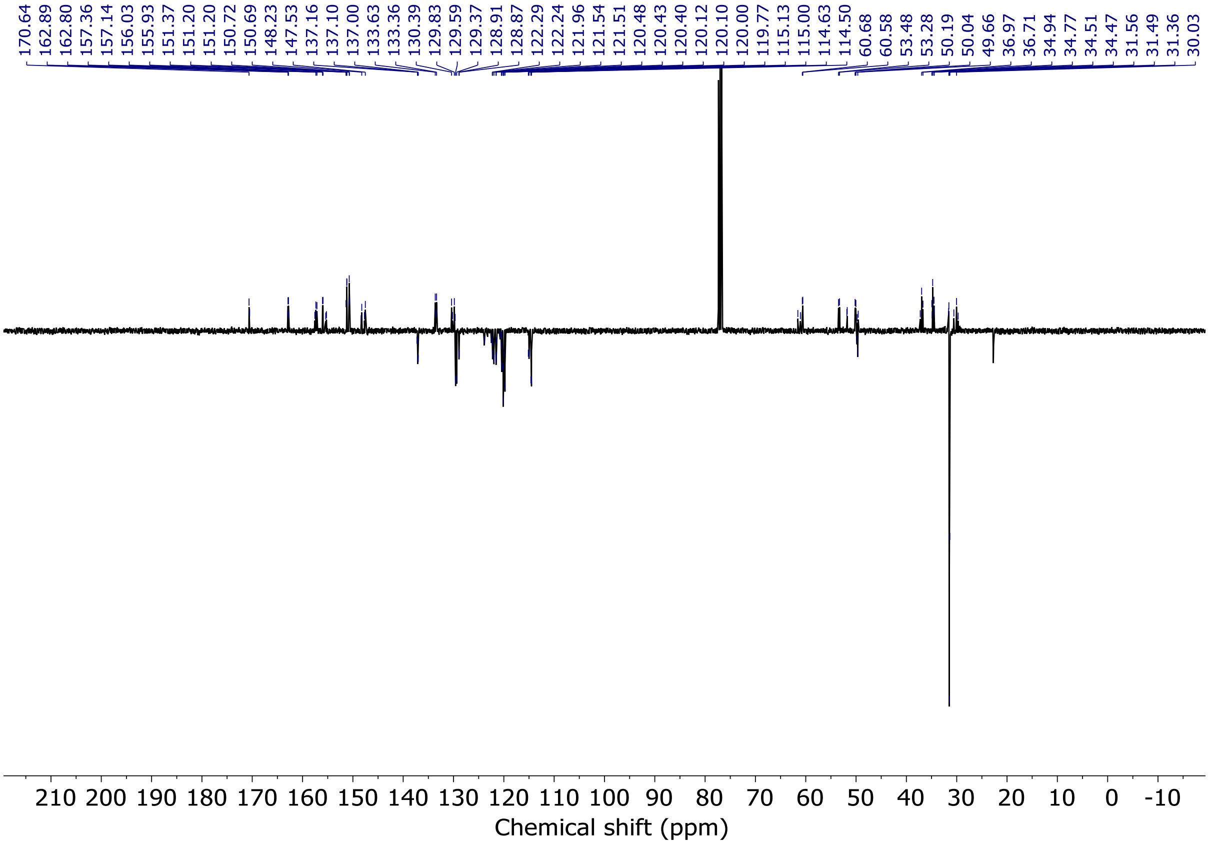

Figure S50. JMOD NMR ($CDCl_3$, 101 MHz) of (R_{ma},$R_{co\text{-}c}$)-**4b** and (S_{ma},$R_{co\text{-}c}$)-**4b** (2.2 : 1 *dr*).

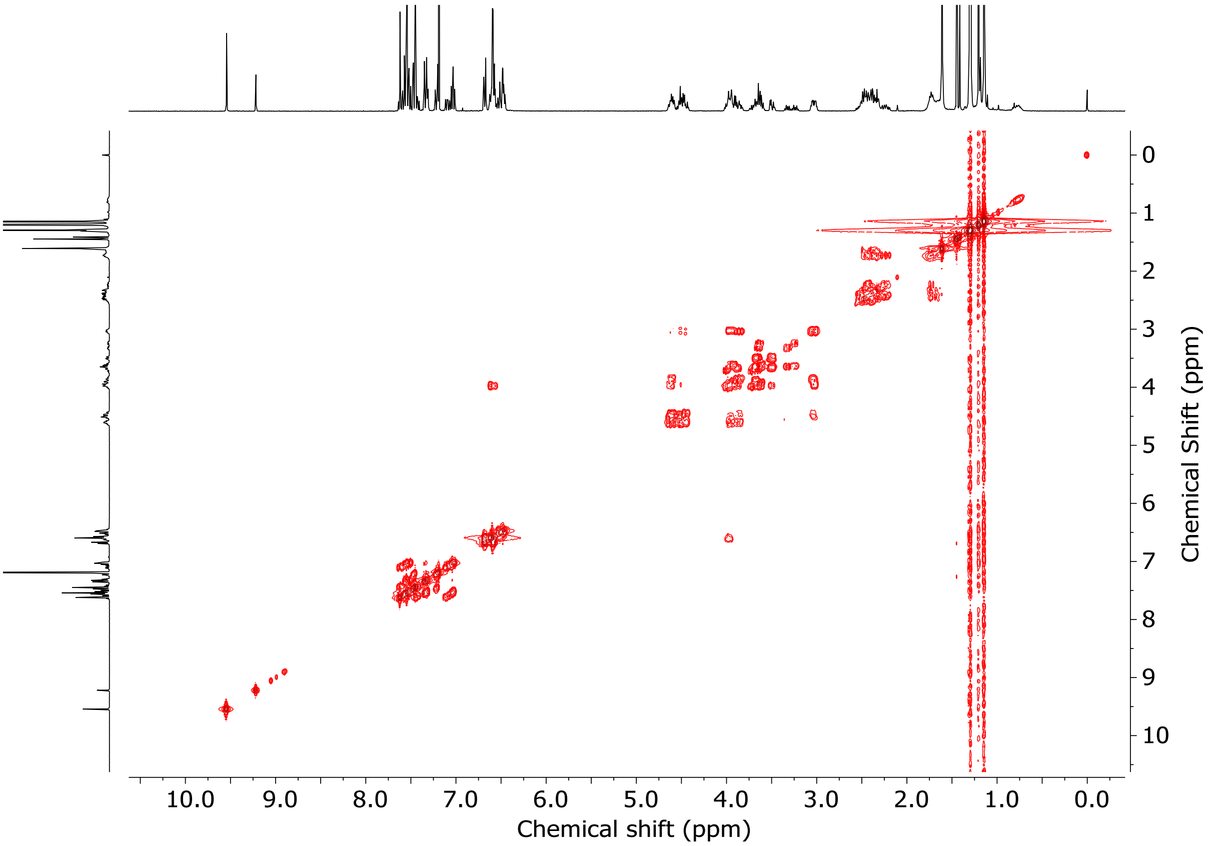

Figure S51. COSY NMR ($CDCl_3$, 400 MHz) of (R_{ma},$R_{co\text{-}c}$)-**4b** and (S_{ma},$R_{co\text{-}c}$)-**4b** (2.2 : 1 *dr*).

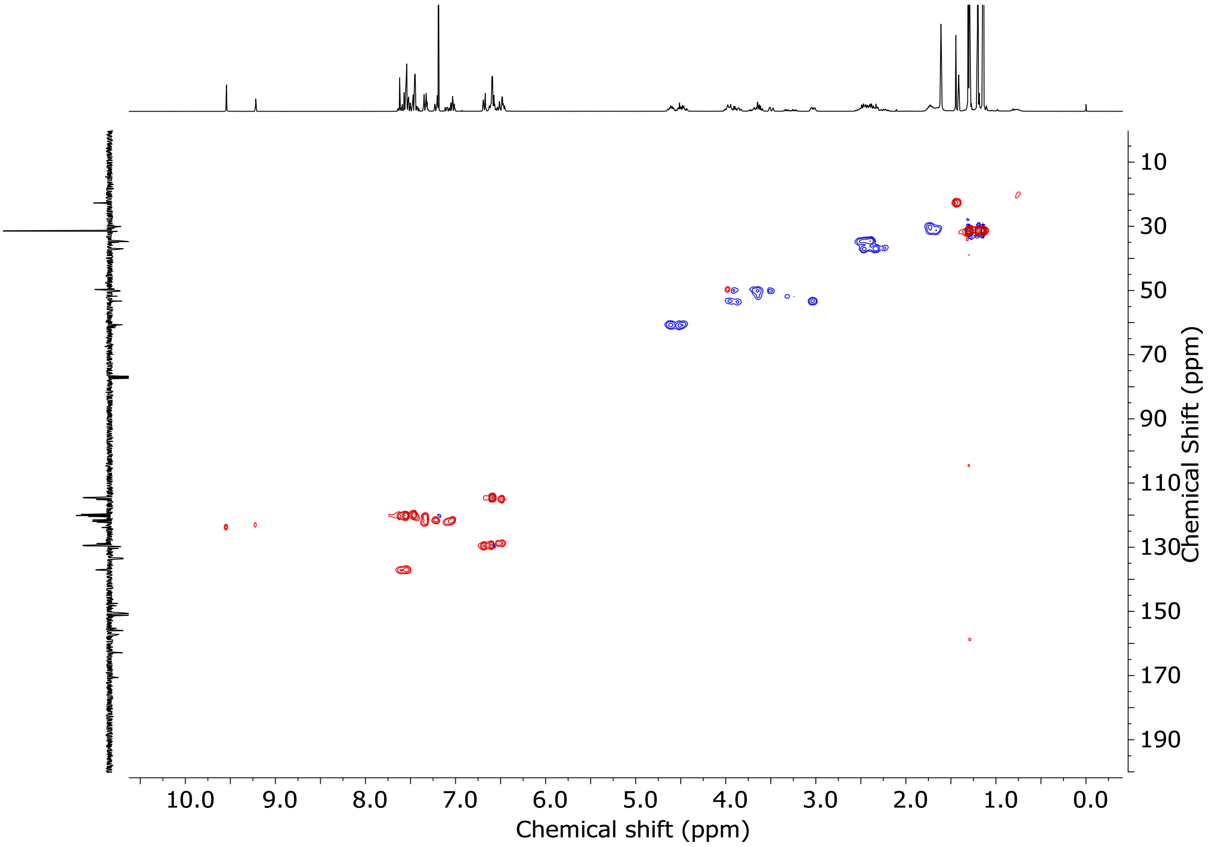

Figure S52. HSQC NMR ($CDCl_3$, 400 MHz) of (R_{ma},$R_{co\text{-}c}$)-**4b** and (S_{ma},$R_{co\text{-}c}$)-**4b** (2.2 : 1 *dr*).

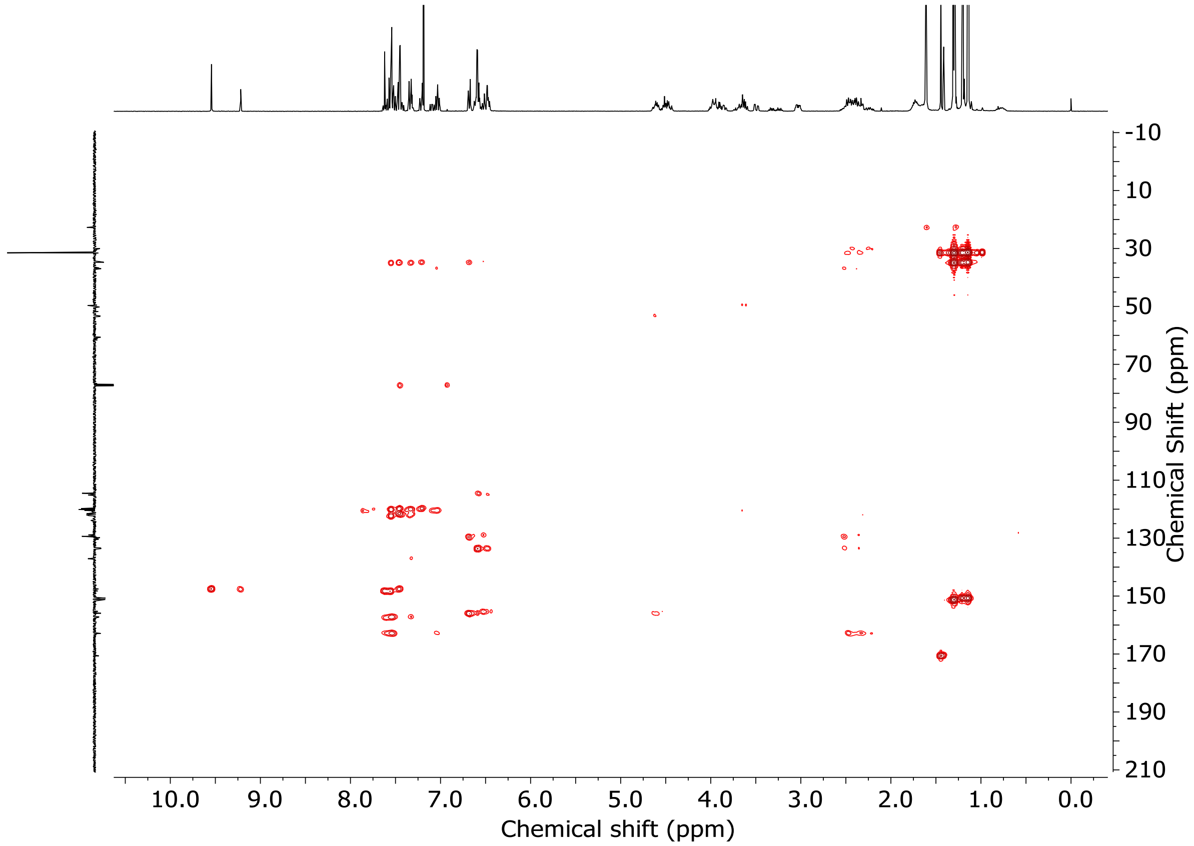

Figure S53. HMBC NMR ($CDCl_3$, 400 MHz) of (R_{ma},$R_{co\text{-}c}$)-**4b** and (S_{ma},$R_{co\text{-}c}$)-**4b** (2.2 : 1 *dr*).

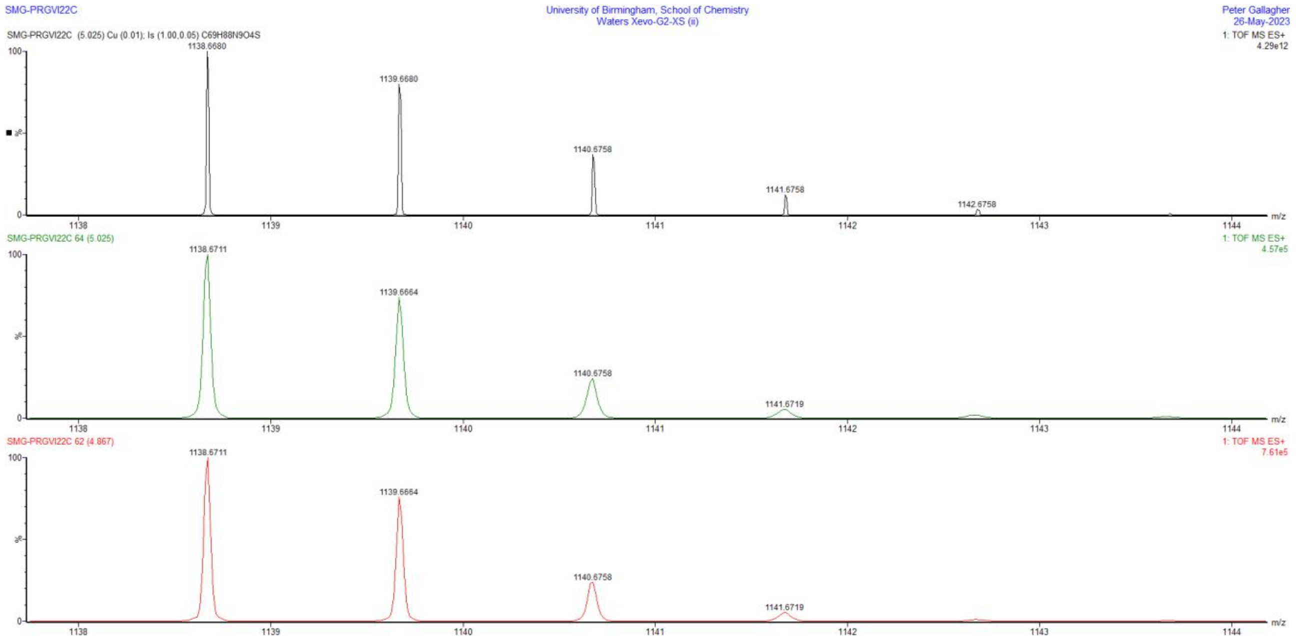

Figure S54. Calculated (top) and observed (middle, bottom) isotopic patterns for rotaxanes **4b**.

Trichloroacetamide rotaxanes 4c

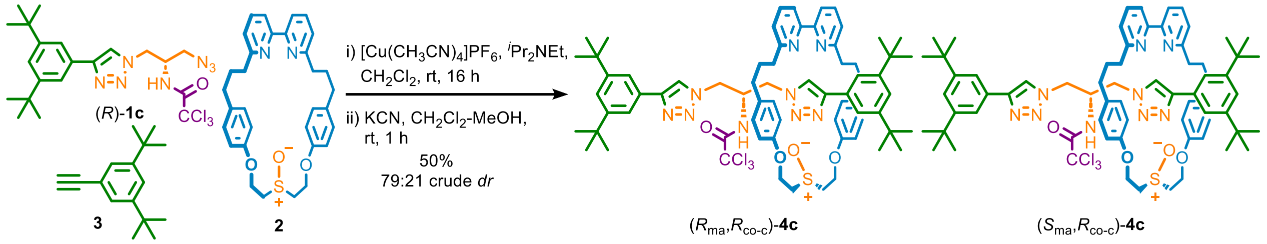

In a CEM vial were added **3** (9.4 mg, 41.7 µmol), (*R*)-**1c** (16.6 mg, 41.7 µmol), **2** (20.0 mg, 38.0 µmol) and [$Cu(CH_3CN)_4PF_6$] (13.6 mg, 36.0 µmol). The vial was sealed and purged with N_2, then CH_2Cl_2 was added (1.0 mL), followed by $^iPr_2NEt$ (13.2 µL, 75.9 µmol). The solution was stirred at rt for 16 h. MeOH (2 mL mL) and KCN as a solid (24.7 mg, 0.38 mmol) were added and the resulting mixture was stirred vigorously for until colourless. The crude mixture was diluted with CH_2Cl_2 (5 mL) and washed with H_2O (5 mL) then EDTA-NH_3 (5 mL), with separation of aqueous and organic phases. The combined aqueous phase was then extracted with CH_2Cl_2 (3 x 5 mL) and the combined organic extracts were washed with brine (10 mL), dried ($MgSO_4$) and concentrated *in vacuo* to give a sample containing **4c** as a mixture of diastereomers (79 : 21 *dr*, Figure S55). Chromatography (CH_2Cl_2-CH_3CN 0→100%) gave **4c** as a white foam (23.5 mg, 50%) as a mixture of diastereoisomers (3.8 : 1 *dr*, Figure S56).

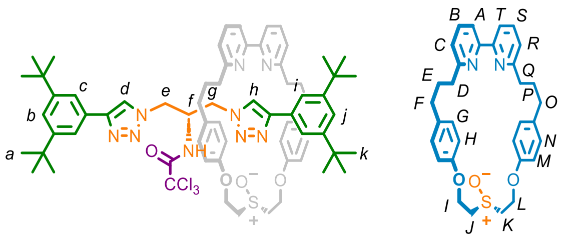

Major Diastereoisomer

¹H NMR (500 MHz, CDCl₃) δ: 9.58 (s, 1H, H_h), 7.82 (d, *J* = 8.4, 1H, NH), 7.77-7.57 (m, 5H, H_B, H_d, H_i, H_S), 7.54 (d, *J* = 1.8, 2H, H_c), 7.50-7.44 (m, 2H, H_A, H_T), 7.37 (t, *J* = 1.8, 1H, H_b), 7.31 (t, *J* = 2.0, 1H, H_j), 6.74-6.66 (m, 4H, H_G, H_H, H_M or H_N), 6.66-6.58 (m, 4H, H_G, H_H, H_M or H_N), 4.76-4.62 (m, 2H, H_I, H_L), 4.62-4.50 (m, 2H, $H_{I'}$, $H_{L'}$), 4.15 (app. 9plet, *J* = 4.1, 1H, H_f), 4.03-3.61 (m, 5H, H_e, H_g, H_J, H_K), 3.39 (dd, *J* = 14.2, 4.8, 1H, $H_{g'}$), 3.27-3.08 (m, 2H, $H_{J'}$, $H_{K'}$) 2.68-2.23 (m, 8H, H_D, H_F, H_O, H_Q), 1.91-1.51 (m, 4H, H_E, H_P), 1.33 (s, 18H, H_a), 1.24 (s, 18H, H_k)

¹³C NMR (126 MHz, CDCl₃) δ: 162.7, 161.6, 157.5, 157.5, 156.1, 156.0, 151.2, 150.8, 148.3, 147.6, 137.1, 137.1, 133.6, 130.4, 129.7, 129.4, 129.3, 123.6, 122.2, 121.9, 121.8, 121.5, 120.9, 120.5, 120.4, 120.2, 120.0, 114.8, 114.6, 92.0, 60.7, 60.4, 53.2, 52.8, 50.3, 49.2, 49.1, 37.1, 36.9, 34.9, 34.8, 34.7, 34.6, 31.8, 31.5, 31.4, 31.1

Minor Diastereoisomer

^{1}H NMR (500 MHz, CDCl$_3$) δ: 9.02 (s, 1H, H_h), 7.77-7.57 (m, 9H, H_A, H_B, H_c, H_i, H_S, H_T, NH), 7.52 (s, 1H, H_d), 7.42 (t, *J* = 1.7, 1H, H_b), 7.38-7.36 (m, 1H, H_j), 7.21 (d, *J* = 7.7, 1H, H_C), 7.19-7.15 (m, 1H, H_R), 6.56-648. (m, 4H, H_G, H_H, H_M or H_N), 6.48-6.42 (m, 4H, H_G, H_H, H_M or H_N) 4.76-4.62 (m, 2H, H_I, H_L), 4.62-4.50 (m, 2H, $H_{I`}$, $H_{L`}$), 4.07 (dd, *J* = 14.2, 6.5, 1H, H_e or H_g), 4.03-3.61 (m, 6H, H_J, H_K, H_f and H_e and $H_{g`}$ or $H_{e`}$ and H_g), 3.27-3.08 (m, 2H, $H_{J`}$, $H_{K`}$), 2.68-2.23 (m, 8H, H_D, H_F, H_O, H_Q), 1.91-1.51 (m, 4H, H_E, H_P), 1.36 (s, 18H, H_a), 1.34 (s, 18H, H_k)

^{13}C NMR (126 MHz, CDCl$_3$) δ: 162.8, 162.6, 161.8, 157.8, 155.1, 155.1, 151.5, 150.9, 148.6, 147.7, 137.3, 137.3, 133.5, 133.5, 129.9, 129.4, 128.8, 128.7, 123.2, 122.6, 122.3, 122.3, 121.7, 121.0, 120.5, 120.4, 120.3, 120.1, 115.0, 115.0, 91.9, 61.1, 61.1, 52.0, 51.7, 50.9, 48.7, 48.5, 37.4, 37.3, 35.0, 35.0, 34.6, 34.3, 31.9, 31.6, 31.5, 31.0

HR-ESI-MS (+ve) m/z = 1240.5483 [M+H]$^+$ (calc. m/z for C$_{69}$H$_{85}$Cl$_3$N$_9$O$_4$S 1240.5510);

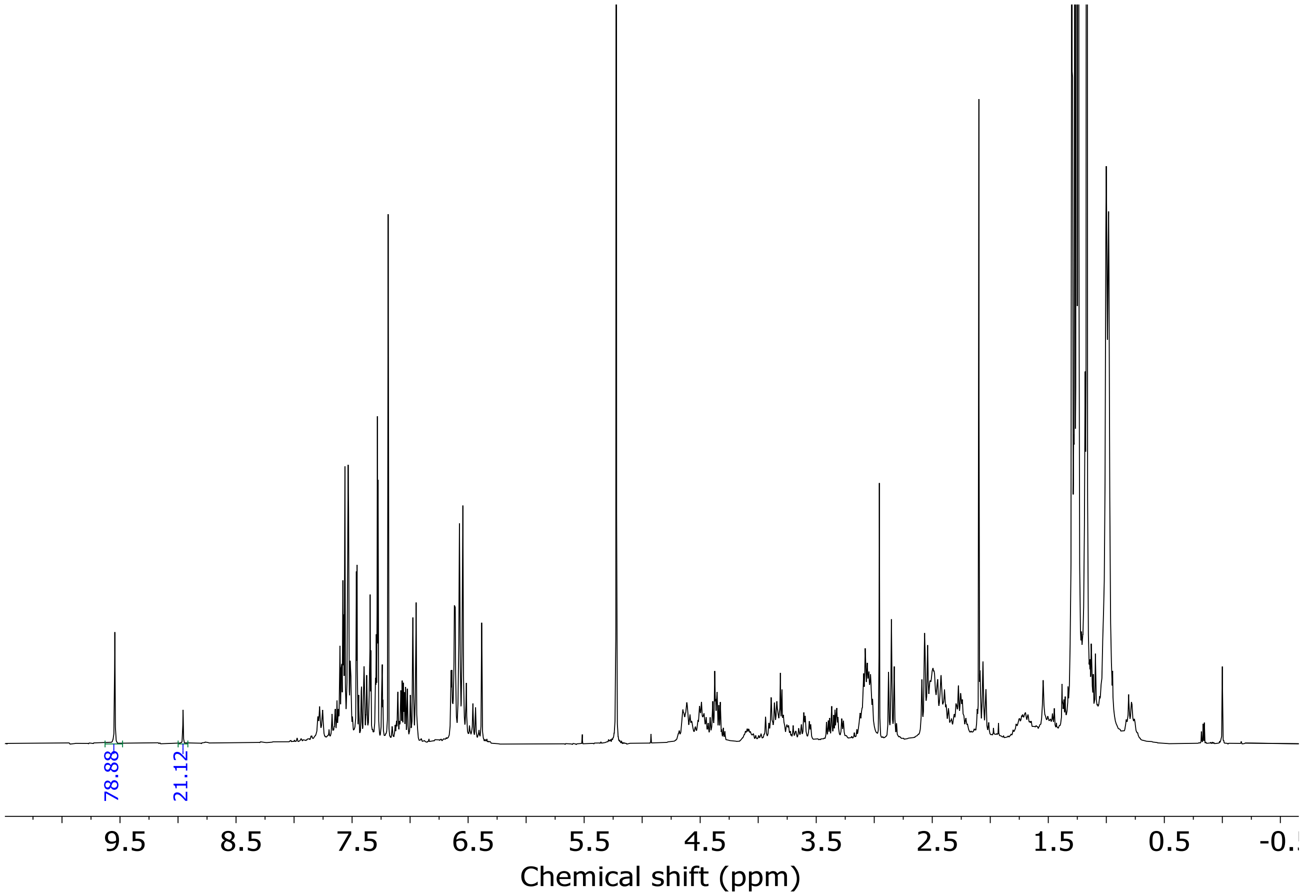

Figure S55. ^{1}H NMR (CDCl$_3$, 400 MHz, 298 K) of (R_{ma},R_{co-c})-**4c** and (S_{ma},R_{co-c})-**4c** prior to purification (79 : 21 *dr*).

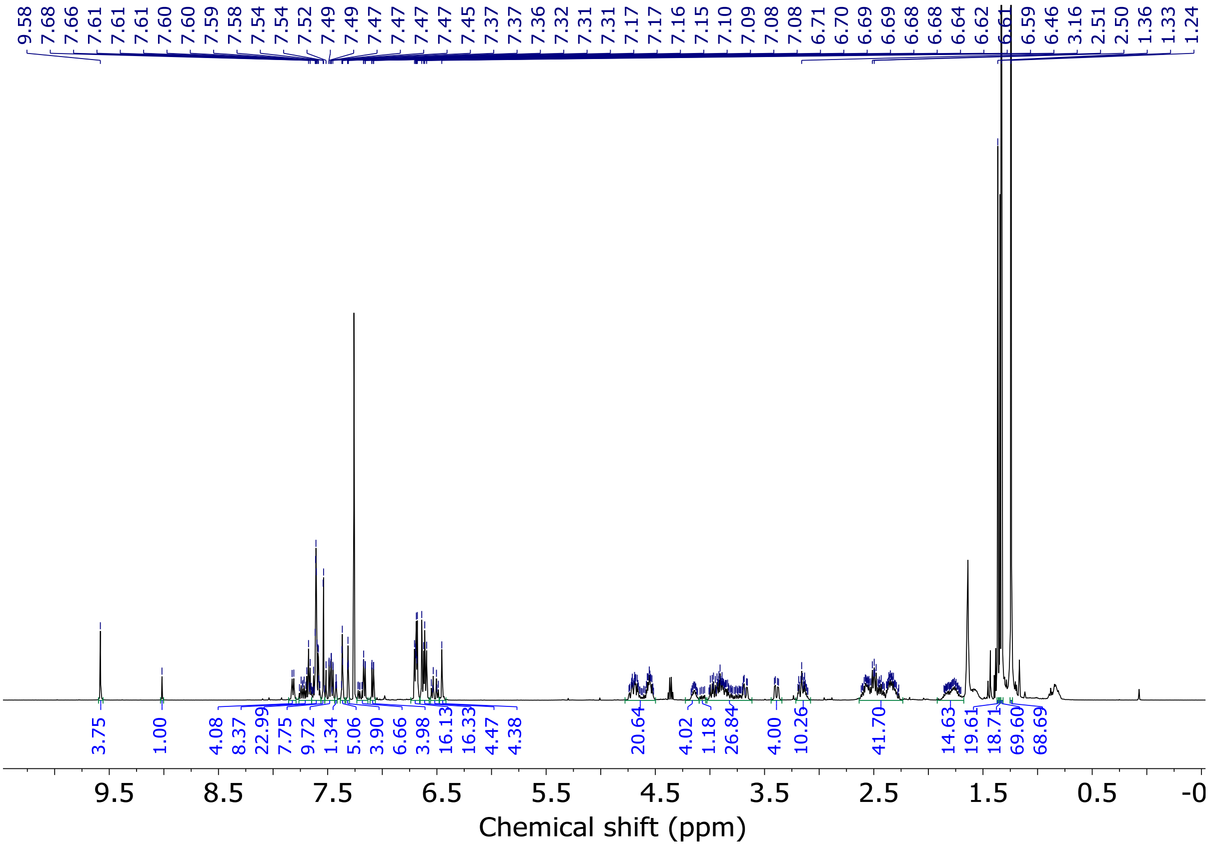

Figure S56. ^{1}H NMR ($CDCl_3$, 500 MHz) of (R_{ma},$R_{co\text{-}c}$)-**4c** and (S_{ma},$R_{co\text{-}c}$)-**4c** (3.8 : 1 *dr*).

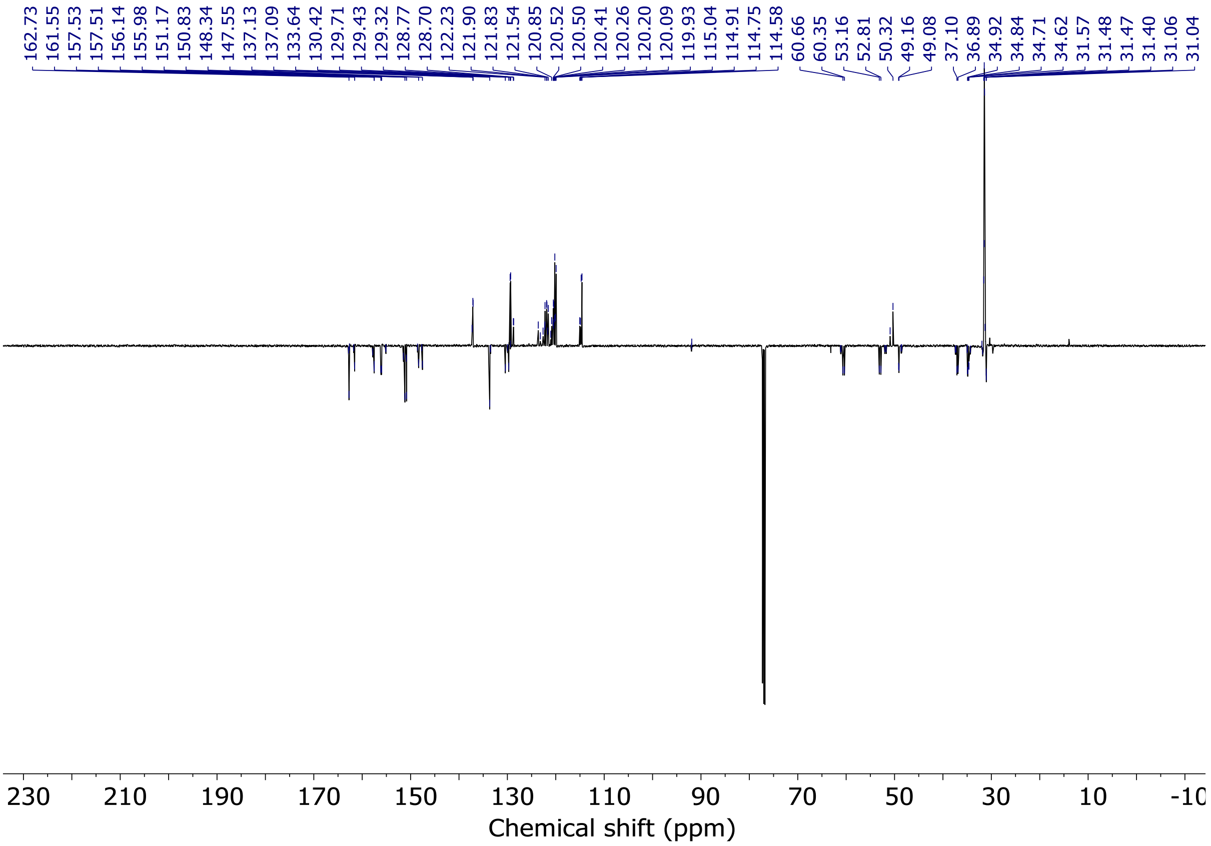

Figure S57. JMOD NMR ($CDCl_3$, 126 MHz) of (R_{ma},$R_{co\text{-}c}$)-**4c** and (S_{ma},$R_{co\text{-}c}$)-**4c** (3.8 : 1 *dr*).

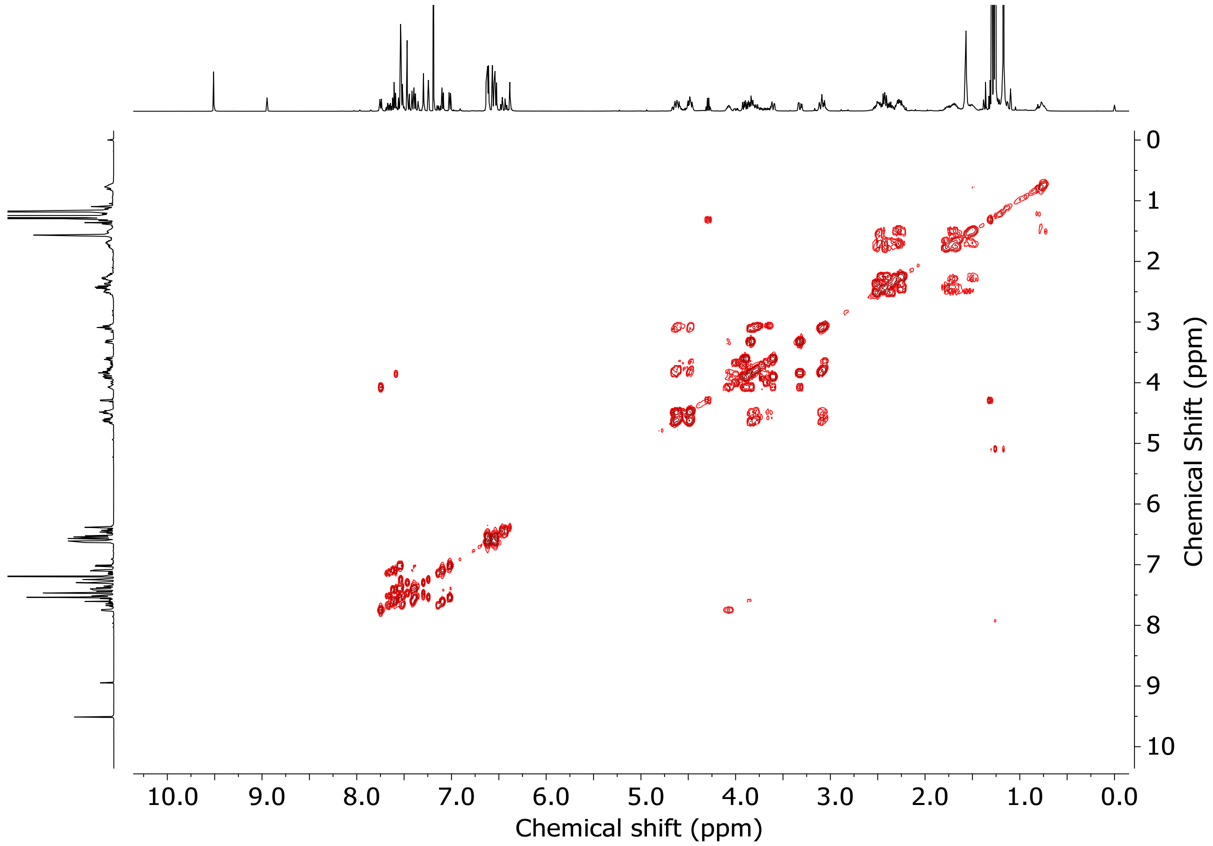

Figure S58. COSY NMR ($CDCl_3$) of (R_{ma},R_{co-c})-**4c** and (S_{ma},R_{co-c})-**4c** (3.8 : 1 *dr*).

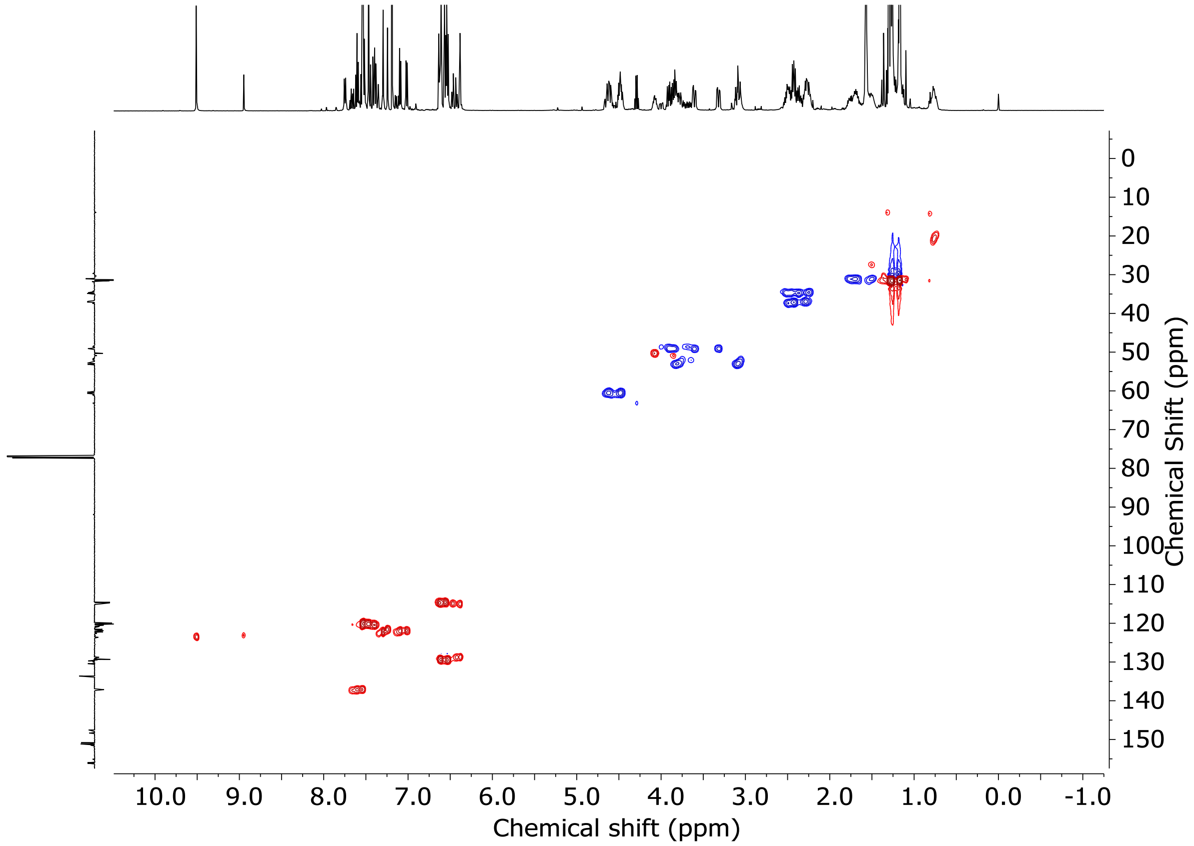

Figure S59. HSQC NMR ($CDCl_3$) of (R_{ma},R_{co-c})-**4c** and (S_{ma},R_{co-c})-**4c** (3.8 : 1 *dr*).

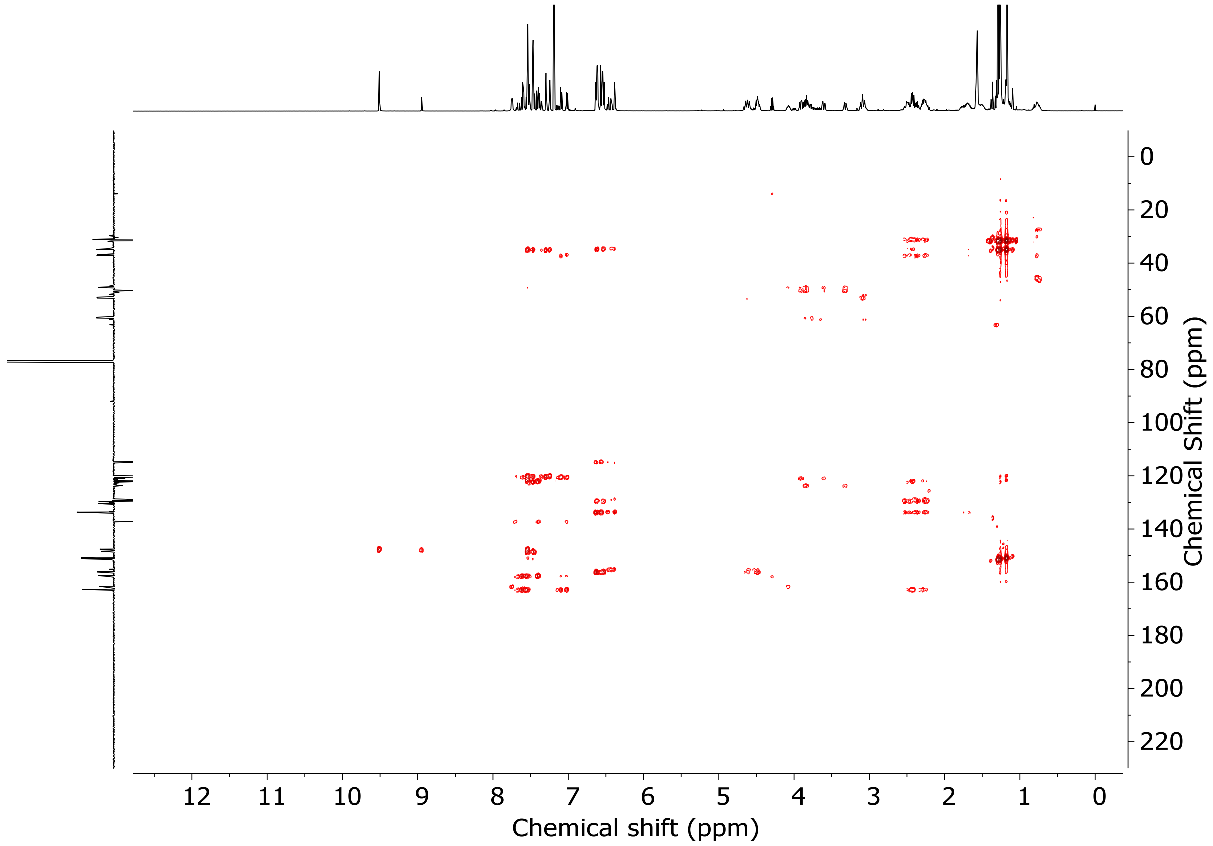

Figure S60. HMBC NMR ($CDCl_3$) of (R_{ma},$R_{co\text{-}c}$)-**4c** and (S_{ma},$R_{co\text{-}c}$)-**4c** (3.8 : 1 *dr*).

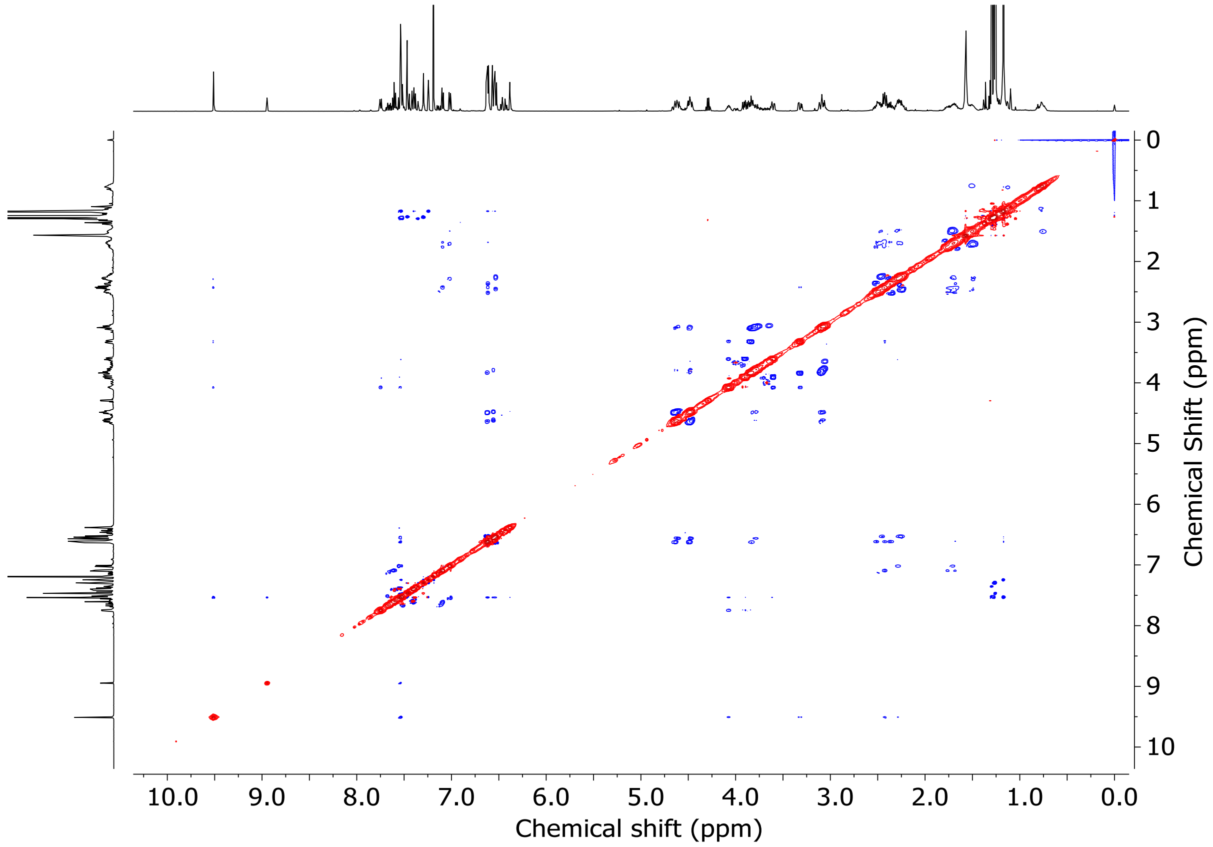

Figure S61. NOESY NMR ($CDCl_3$) of (R_{ma},$R_{co\text{-}c}$)-**4c** and (S_{ma},$R_{co\text{-}c}$)-**4c** (3.8 : 1 *dr*).

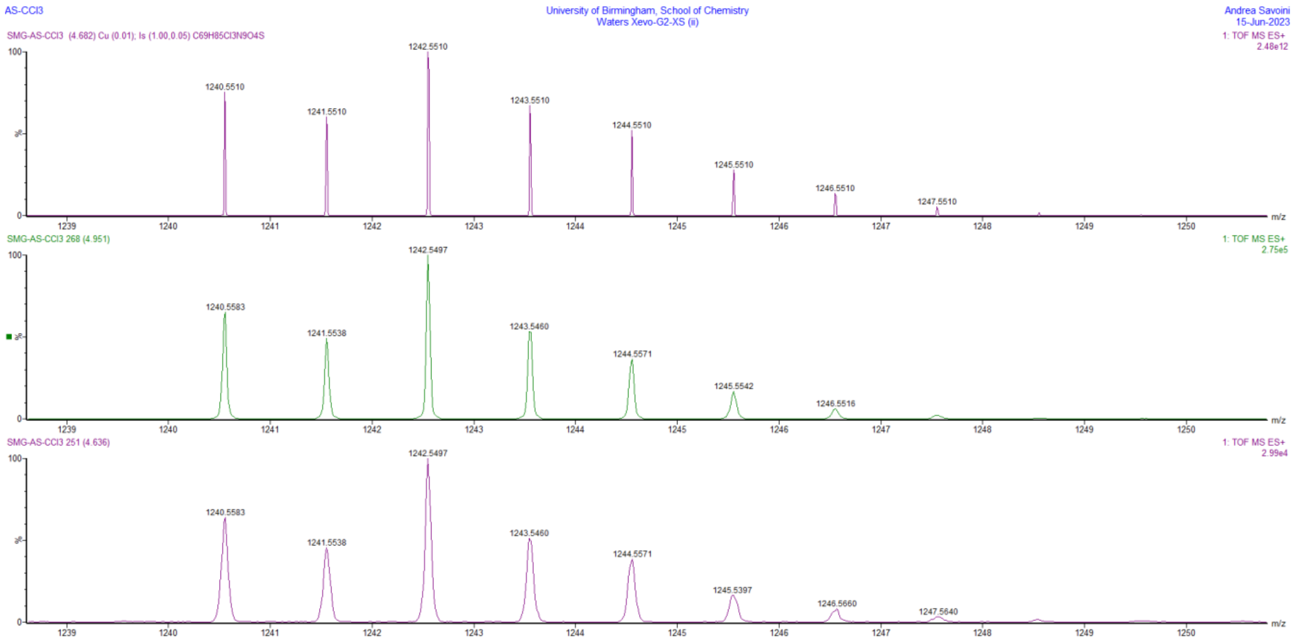

Figure S62. Calculated (top) and observed (middle, bottom) isotopic patterns for rotaxanes **4c**.

Trifluoroacetamide rotaxanes 4d

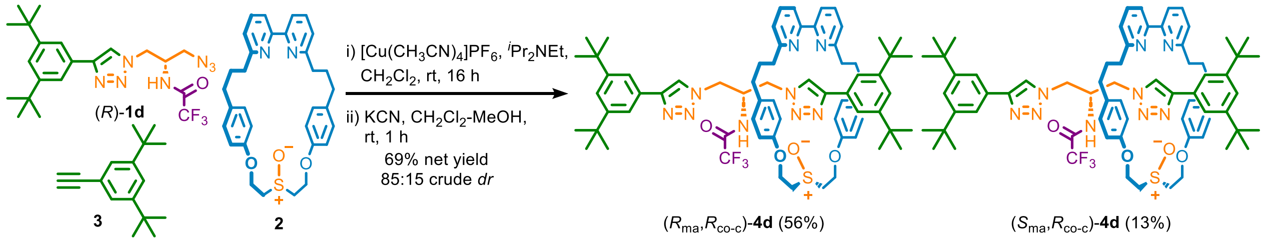

In a CEM vial were added the **3** (18.6 mg, 86.8 μmol), (*R*)-**1d** (39.0 mg, 86.4 μmol), **2** (40.3 mg, 76.5 μmol) and [$Cu(CH_3CN)_4PF_6$] (27.9 mg, 74.9 μmol). The vial was sealed and purged with N_2, then CH_2Cl_2 was added (1.9 mL), followed by ^{i}Pr_2NEt (27 μL, 155 μmol). The solution was stirred at rt for 16 h. MeOH (2 mL mL) and KCN as a solid (49.8 mg, 765 μmol) were added and the resulting mixture was stirred vigorously for until colourless. The crude mixture was diluted with CH_2Cl_2 (5 mL) and washed with H_2O in two portions (10 mL and 5 mL), with separation of aqueous and organic phases. The combined aqueous phase was then extracted with CH_2Cl_2 (3 x 5 mL) and the combined organic extracts were washed with brine (10 mL), dried ($MgSO_4$) and concentrated *in vacuo* to give a sample containing **4d** as a mixture of diastereomers (85 : 15 *dr*, Figure S63). Chromatography (CH_2Cl_2-CH_3CN 0→25% then 0→5% MeOH) gave rotaxanes **4d** as white foams; (R_{ma},R_{co-c})-**4d** (51.1 mg, 42.8 μmol, 56%), (S_{ma},R_{co-c})-**4d** (12.1 mg, 13%).

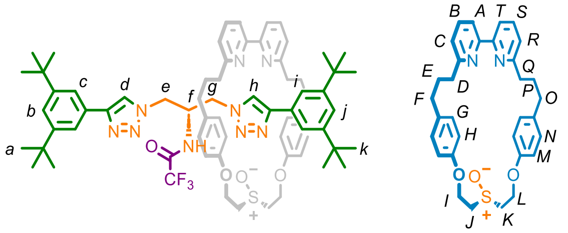

(R_{ma},$R_{co\text{-}c}$)-**4d** - Major diastereoisomer

^{1}H NMR (400 MHz, CDCl$_3$) δ: 10.0 (s, 1H, H$_h$), 8.30 (d, *J* = 8.4, 1H, NH), 7.69 (t, *J* = 7.9, 1H, H$_B$), 7.60 (d, *J* = 1.9, 2H, H$_c$ or H$_i$), 7.55 (t, *J* = 7.7, 1H, H$_S$), 7.47 (d, *J* = 7.7, 1H, H$_A$), 7.44 (s, 1H, H$_d$), 7.43-7.39 (m, 3H, H$_T$, H$_c$ or H$_i$), 7.33 (t, *J* = 1.9, 1H, H$_b$ or H$_j$), 7.28 (t, *J* = 1.9, 1H, H$_b$ or H$_j$), 7.21 (d, *J* = 7.5, 1H, H$_C$), 7.00, (d, *J* = 7.7, 1H, H$_R$), 6.86 (d, *J* = 8.9, 2H, H$_H$ or H$_M$), 6.83-7.83 (m, 4H, H$_G$ and H$_H$ or H$_M$ and H$_N$), 6.65 (d, *J* = 8.5, 2H, H$_G$ or H$_N$), 4.82-4.64 (m, 2H, H$_I$, H$_L$), 4.59-4.39 (m, 3H, H$_f$, H$_{I'}$, H$_{L'}$), 3.90 (dd, *J* = 14.3, 10.0, 1H, H$_g$), 3.78-3.62 (m, 3H, H$_e$, H$_J$, H$_K$), 3.38 (dd, *J* = 14.3, 3.6, 1H, H$_{e'}$), 3.19-3.01 (m, 3H, H$_{e'}$, H$_{J'}$, H$_{K'}$), 2.74-2.62 (m, 1H, H$_F$ or H$_O$), 2.60-2.42 (m, 4H, H$_D$ or H$_Q$, H$_F$ or H$_O$ and H$_{F'}$ or H$_{O'}$), 2.36-2.09 (m, 2H, H$_D$ or H$_O$), 2.09-1.66 (m, 4H, H$_E$, H$_P$), 1.31 (s, 18H, H$_a$ or H$_k$), 1.21 (s, 18H, H$_a$ or H$_k$).

^{13}C NMR (101 MHz, CDCl$_3$) δ: 162.9, 162.7, 157.3, 157.1, 156.9, 156.7 (q, $J_{C\text{-}P}$ = 36.5) 156.6, 156.3, 151.0, 150.8, 148.1, 148.1, 137.3, 137.0, 134.0, 133.8, 130.5, 129.9, 129.7, 129.6, 123.5, 122.0, 121.8, 121.7, 121.5, 120.7, 120.3, 120.1, 120.0, 120.0, 119.9, 115.4 (q, $J_{C\text{-}P}$ = 287.3), 115.0, 114.6, 60.5, 60.1, 53.1, 52.7, 49.6, 49.2, 48.2, 36.8, 36.7, 34.9, 34.8, 34.7, 31.6, 31.5, 31.4, 31.4, 31.4, 31.3, 30.3.

LR-ESI-MS (+ve) m/z = 1192.6 [M+H]$^+$ (calc. m/z for $C_{69}H_{84}F_3N_9O_4S$);

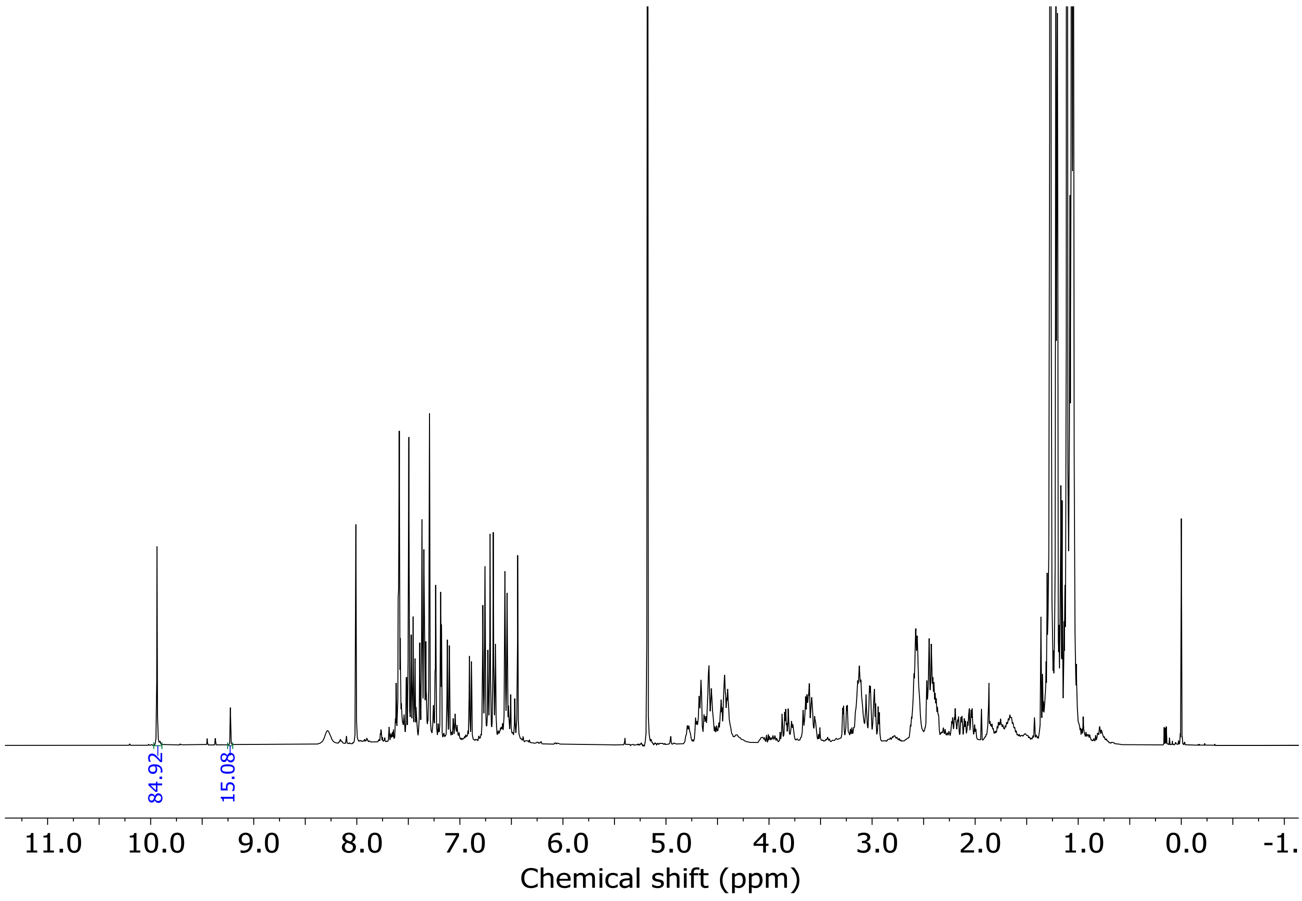

Figure S63. ^{1}H NMR (CDCl$_3$, 400 MHz) of (R_{ma},$R_{co\text{-}c}$)-**4d** and (S_{ma},$R_{co\text{-}c}$)-**4d** prior to purification (85 : 15 *dr*).

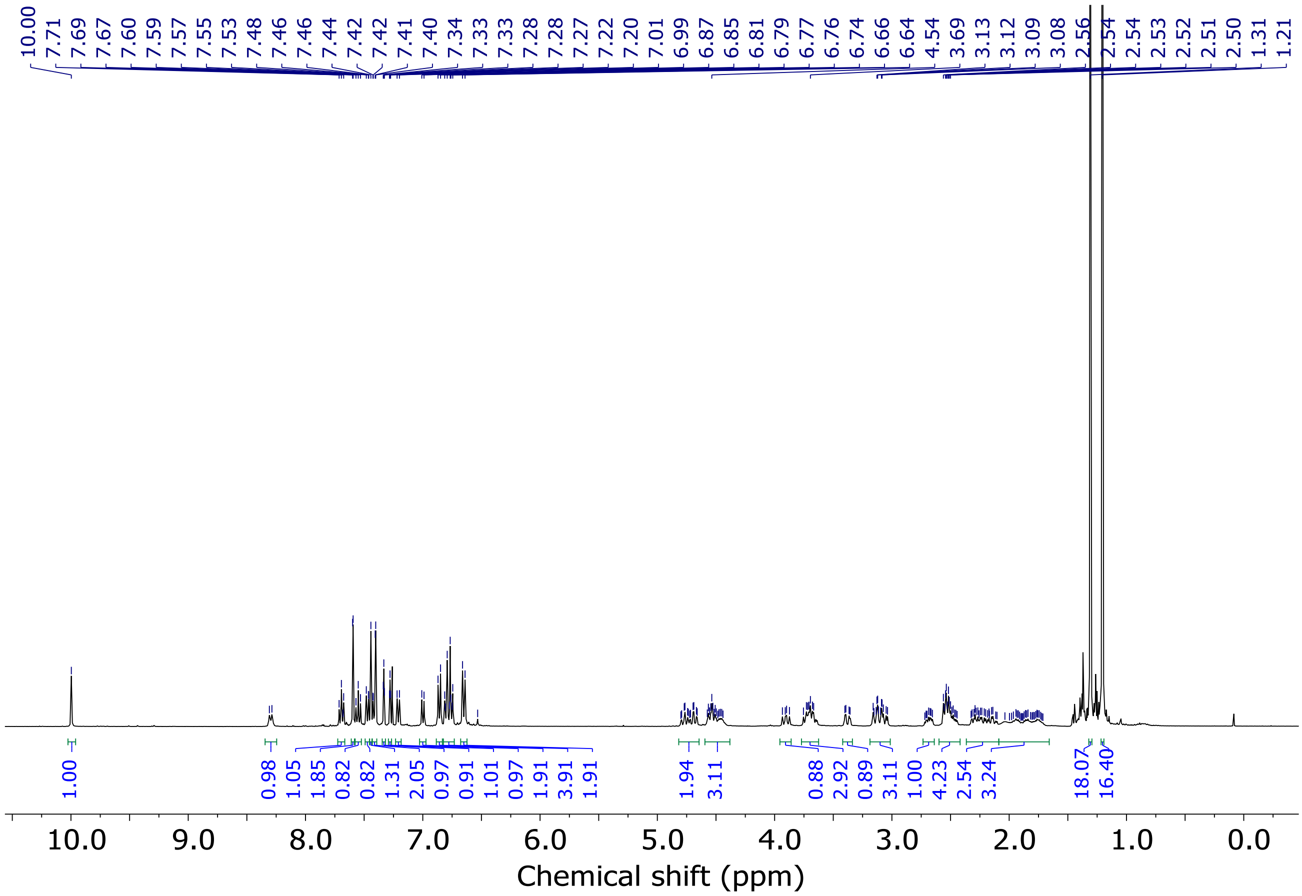

Figure S64. ^{1}H NMR ($CDCl_3$, 400 MHz) of (R_{ma},$R_{co\text{-}c}$)-**4d**.

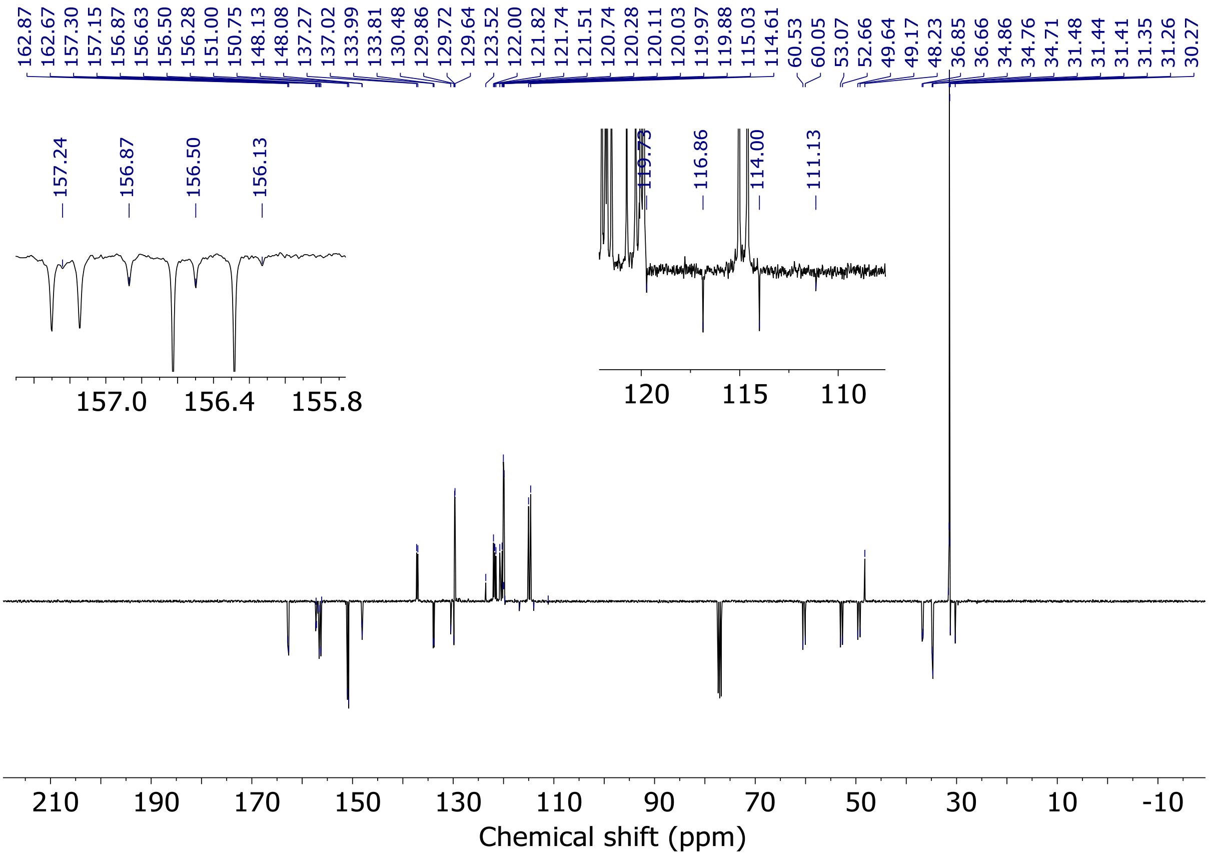

Figure S65. JMOD NMR ($CDCl_3$, 101 MHz) of (R_{ma},$R_{co\text{-}c}$)-**4d**.

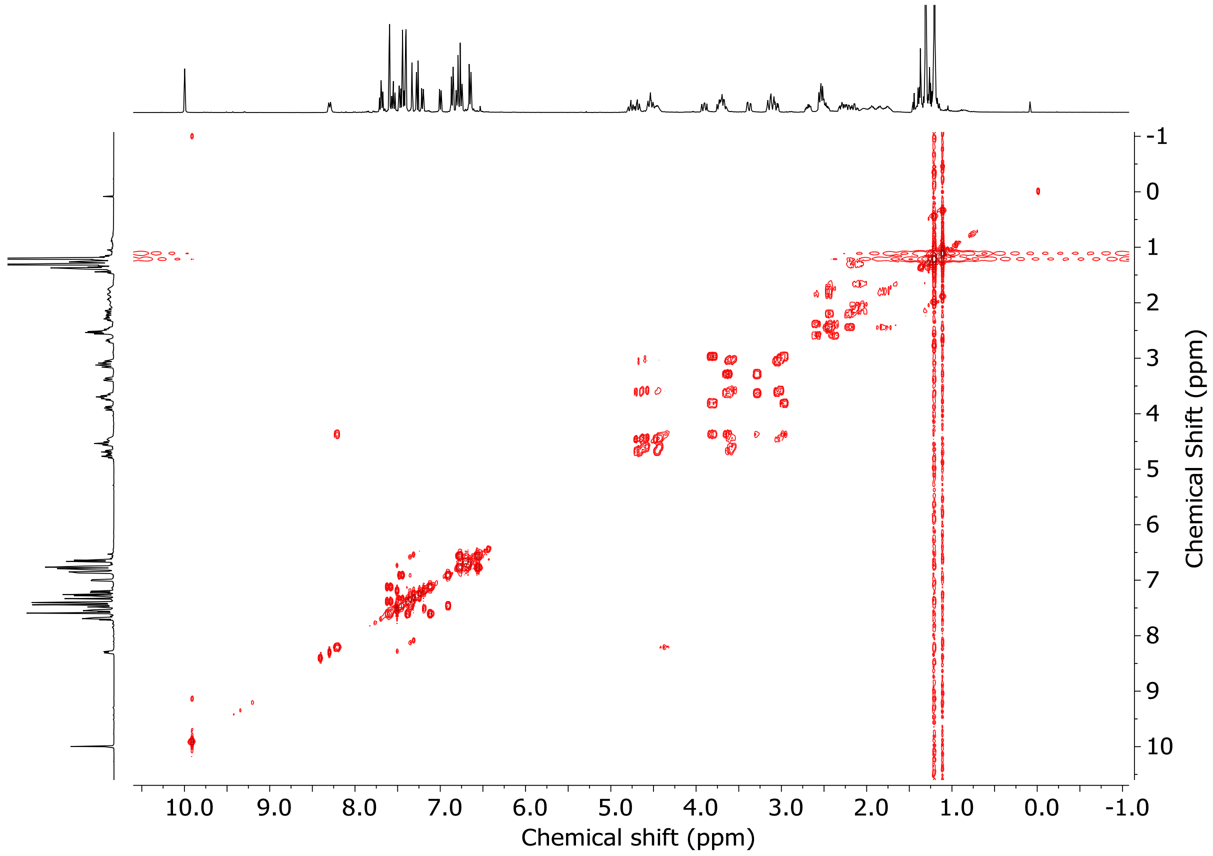

Figure S66. COSY NMR ($CDCl_3$) of (R_{ma},$R_{co\text{-}c}$)-**4d**.

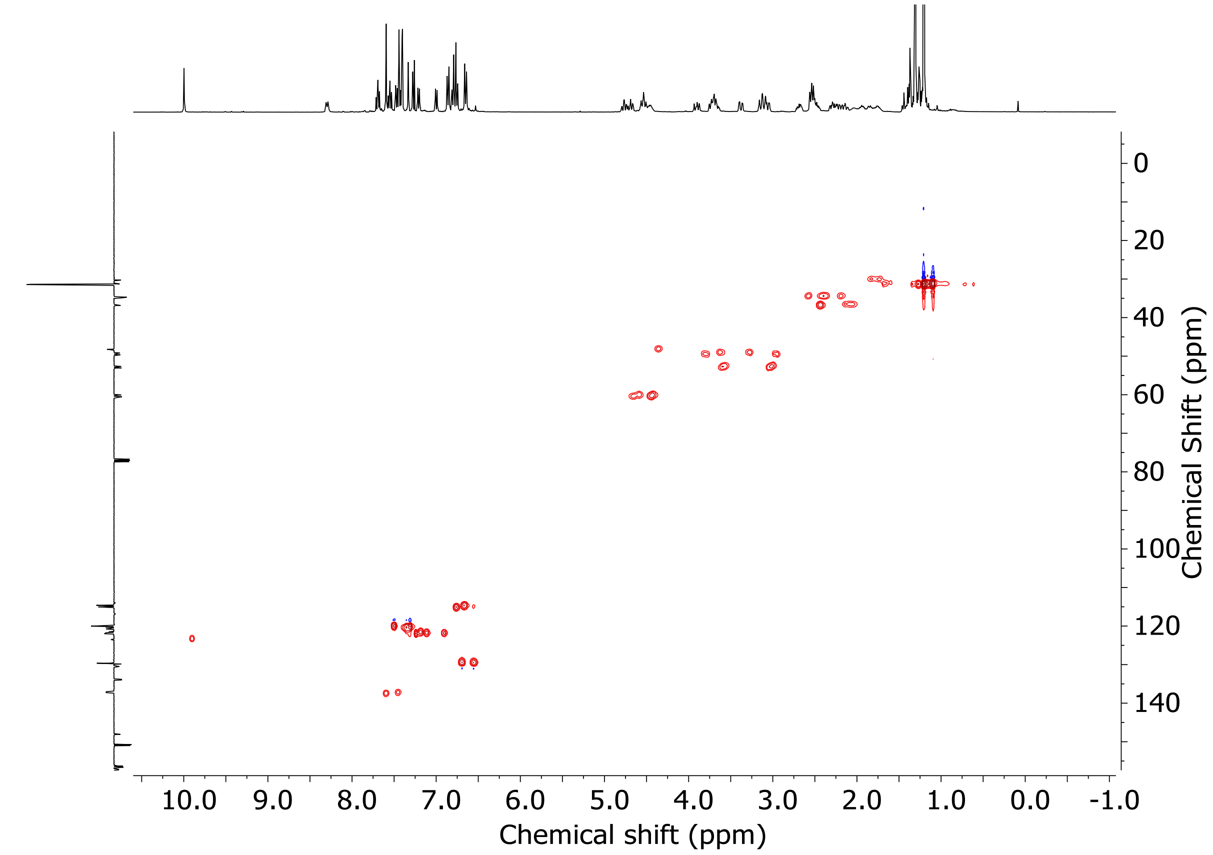

Figure S67. HSQC NMR ($CDCl_3$) of (R_{ma},$R_{co\text{-}c}$)-**4d**.

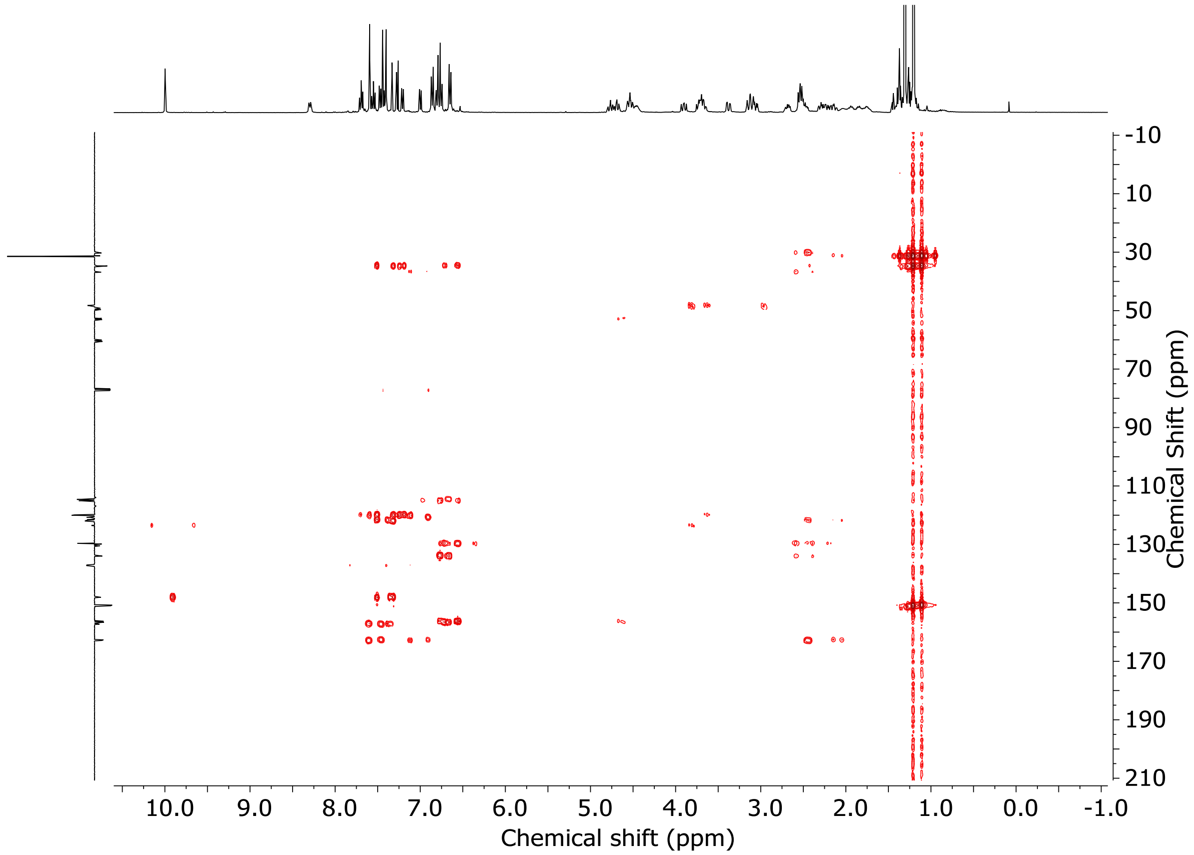

Figure S68. HMBC NMR ($CDCl_3$) of (R_{ma},$R_{co\text{-}c}$)-**4d**.

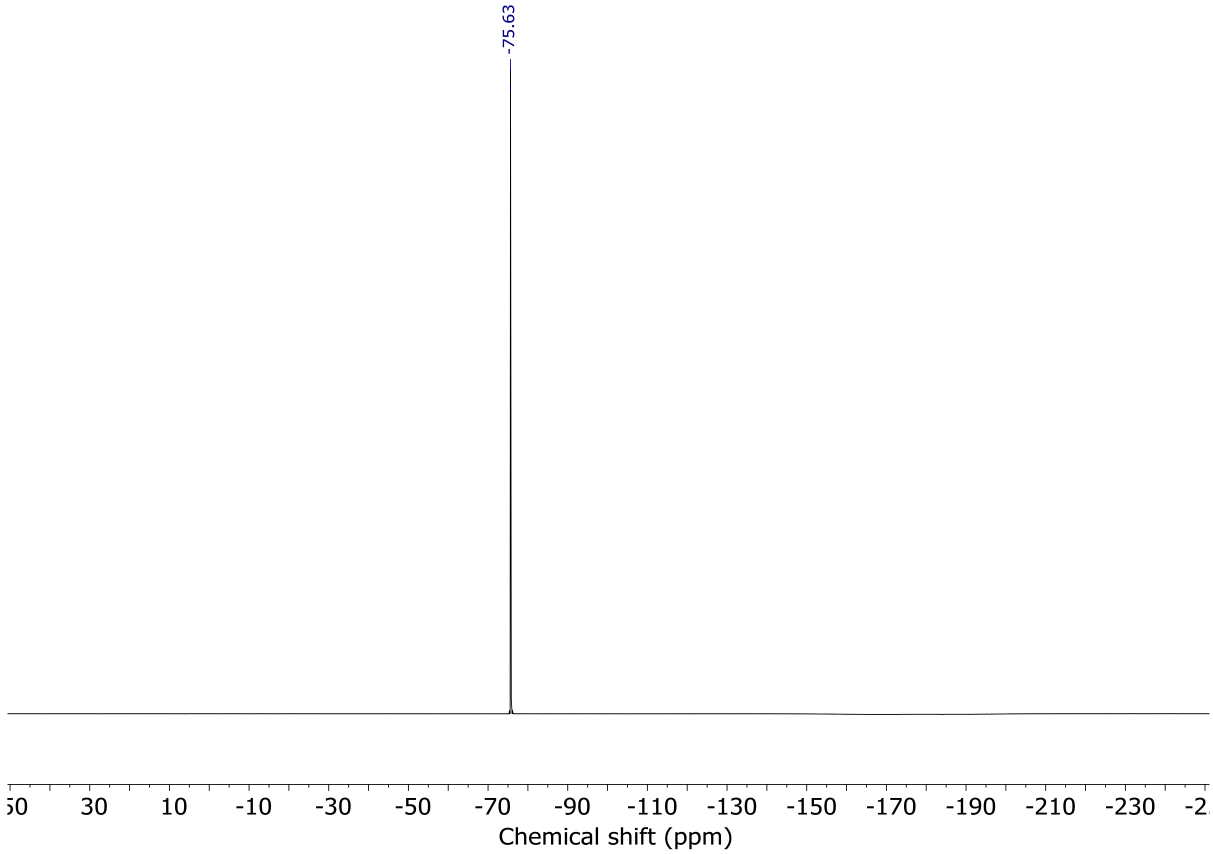

Figure S69. ^{19}F NMR ($CDCl_3$, 376 MHz) of (R_{ma},$R_{co\text{-}c}$)-**4d**.

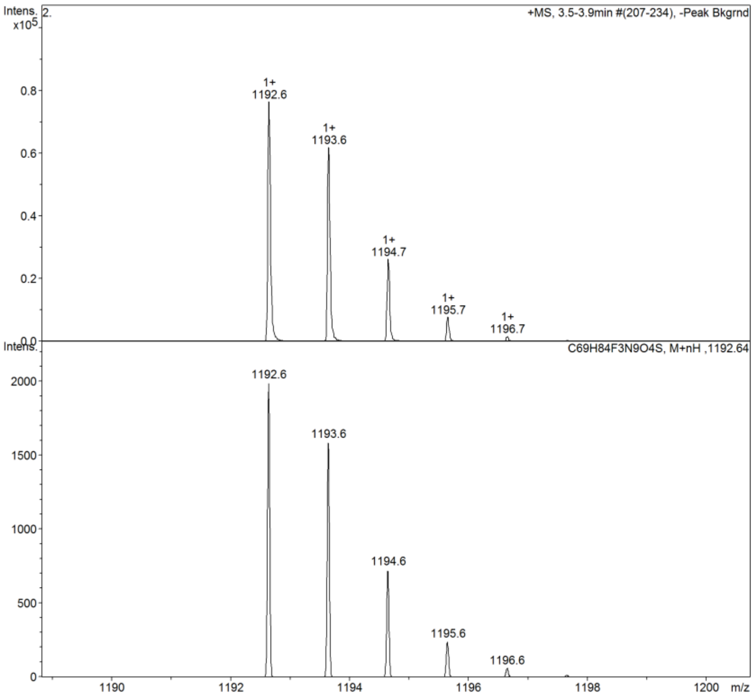

Figure S70. Observed (top) and calculated (bottom) isotopic patterns for rotaxanes (R_{ma},$R_{co\text{-}c}$)-**4d**.

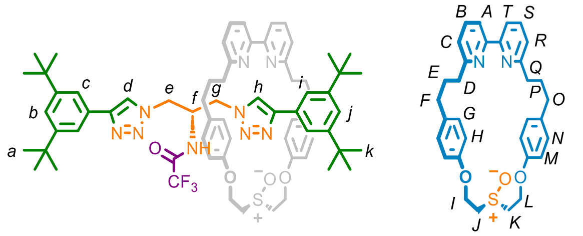

(S_{ma},$R_{co\text{-}c}$)-**4d** - Minor diastereoisomer

^{1}H NMR (500 MHz, CDCl$_3$) δ: 9.28 (s, 1H, H_h), 7.79 (d, J = 7.4, 1H, NH), 7.73 (t, J = 7.9, 1H, H_B), 7.69 (t, J = 7.7, 1H, H_S), 7.60 (d, J = 1.8, 2H, H_i), 7.58-7.55 (m, 3H, H_A, H_c), 7.54 (t, J = 1.1, 1H, H_T), 7.46 (s, 1H, H_d), 7.42 (t, J = 1.7, 1H, H_b), 7.35 (t, J = 1.8, 1H, H_j), 7.21 (d, J = 7.9, 1H, H_C), 7.16 (d, J = 7.9, 1H, H_R), 6.63-6.50 (m, 8H, H_G, H_H, H_M, H_N), 4.74-4.48 (m, 4H, H_I, H_L), 4.12-4.05 (m, 1H, H_f), 4.01 (dd, J = 14.4, 6.6, 1H, H_g), 3.88 (dd, J = 14.6, 4.1, 1H, H_g), 3.77 (dd, J = 14.6, 6.9, 1H, $H_{g'}$), 3.68 (dt, J = 13.3, 6.5, 1H, H_J or H_K), 3.66-3.55 (m, 2H, $H_{e'}$, H_J or H_K), 3.39-3.24 (m, 2H, $H_{J'}$, $H_{K'}$), 2.62-2.29 (m, 8H, H_D, H_F, H_O, H_Q), 1.87-1.52 (m, 4H, H_E, H_P), 1.37 (s, 18H, H_a), 1.30 (s, 18H, H_k)

^{13}C NMR (126 MHz, CDCl$_3$) δ: 163.0, 162.8, 157.7, 157.7, 157.2 (q, $J_{C\text{-}F}$ = 37.3), 155.7, 155.6, 154.0, 151.5, 151.3, 151.0, 148.7, 147.9, 137.8, 137.4, 134.7, 133.8, 133.8, 130.8, 130.2, 129.6, 129.2, 129.0, 128.3, 125.2, 125.1, 123.4, 122.7, 122.4, 122.3, 121.8, 120.8, 120.7, 120.6, 120.3, 120.3, 118.8, 115.5 (q, $J_{C\text{-}F}$ = 286.2) 115.4, 115.2, 61.5, 61.3, 52.0, 51.3, 49.8, 48.8, 48.7, 37.3, 37.3, 35.1, 35.1, 34.7, 34.6, 31.6, 31.5, 31.4, 29.9, 29.9.

LR-ESI-MS (+ve) m/z = 1192.6 $[M+H]^+$ (calc. m/z for $C_{69}H_{84}F_3N_9O_4S$);

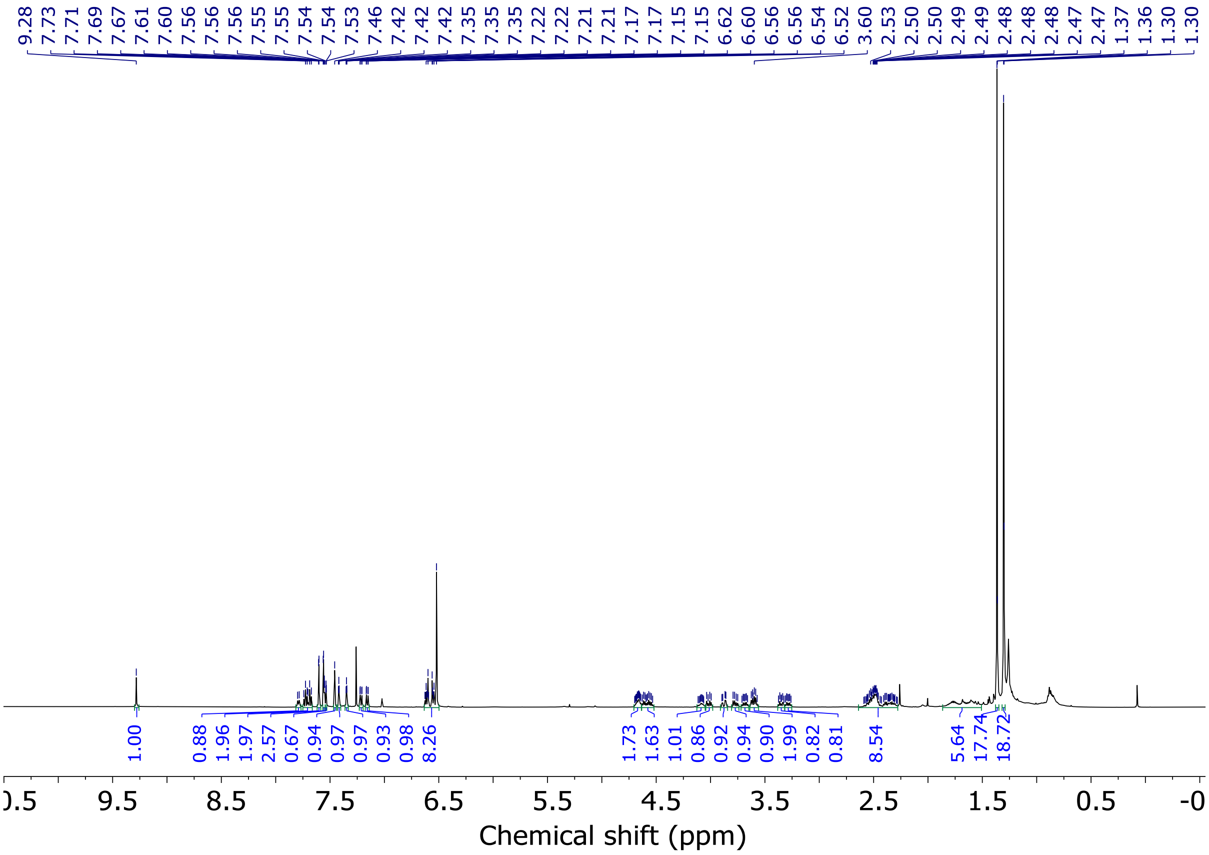

Figure S71. ^{1}H NMR ($CDCl_3$, 500 MHz) of (S_{ma},$R_{co\text{-}c}$)-**4d**.

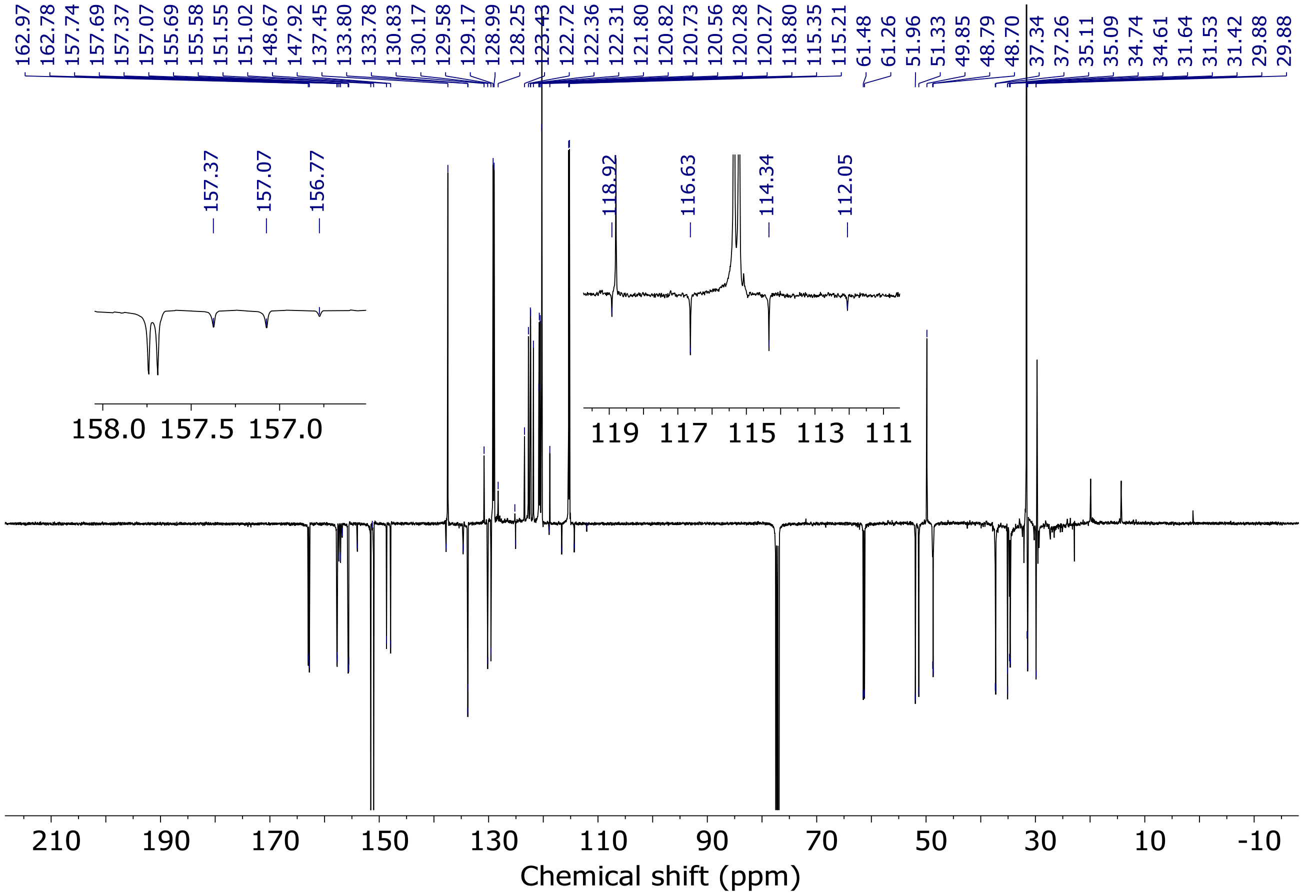

Figure S72. JMOD NMR ($CDCl_3$, 126 MHz) of (S_{ma},$R_{co\text{-}c}$)-**4d**.

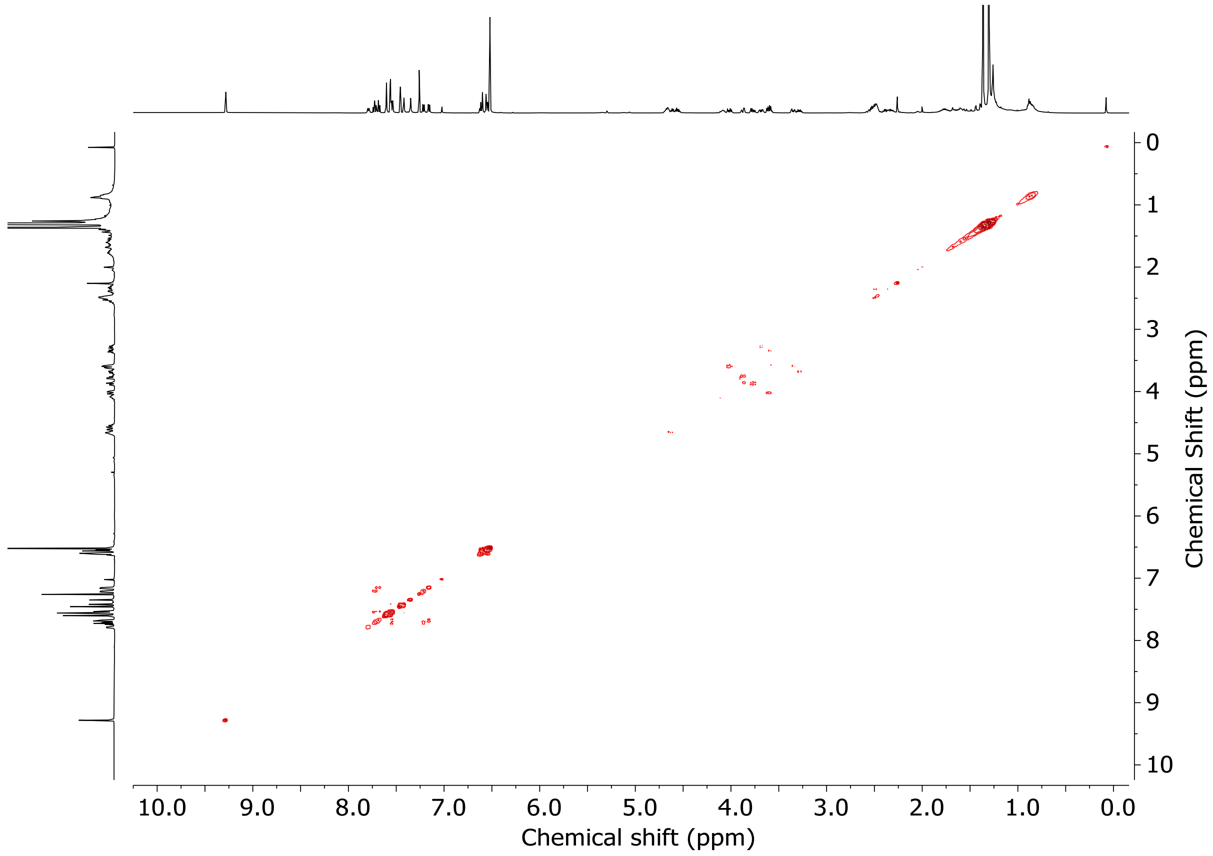

Figure S73. COSY NMR ($CDCl_3$) of (S_{ma},$R_{co\text{-}c}$)-**4d**.

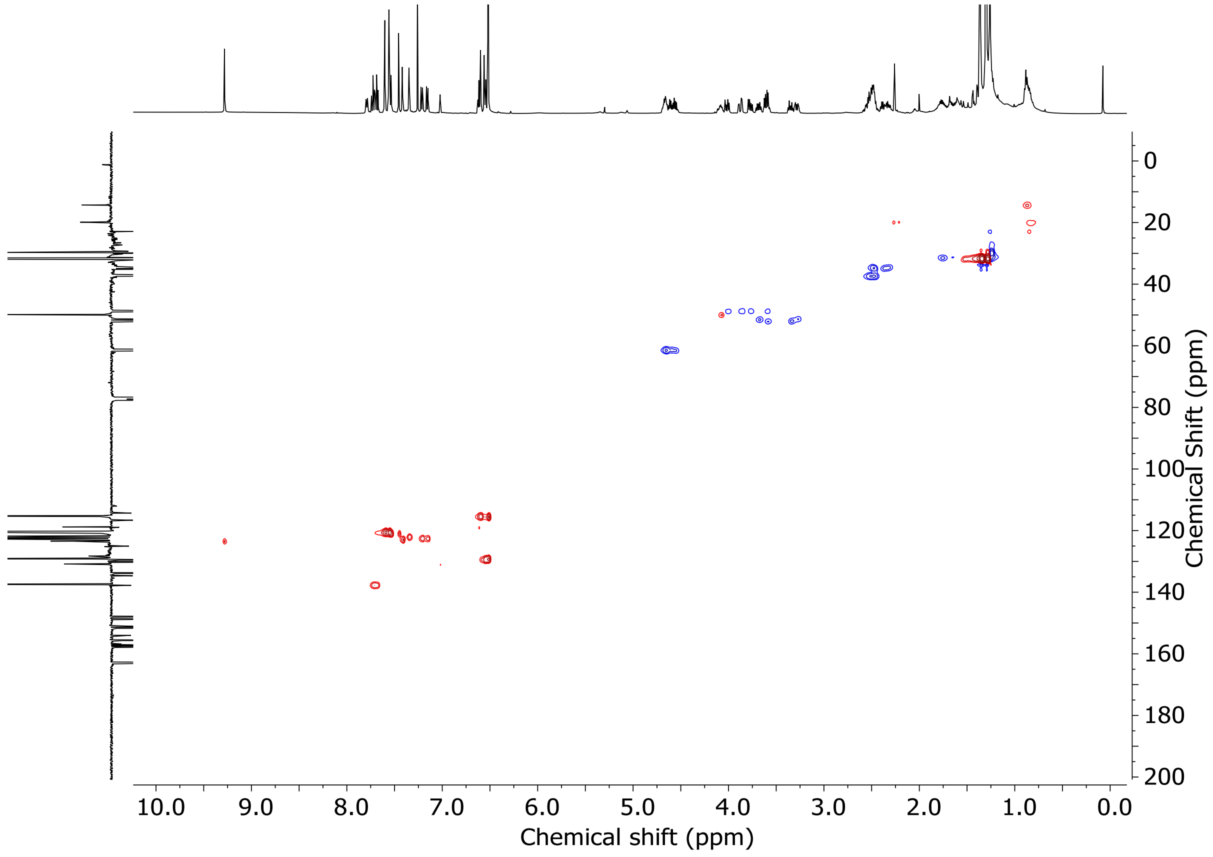

Figure S74. HSQC NMR ($CDCl_3$) of (S_{ma},$R_{co\text{-}c}$)-**4d**.

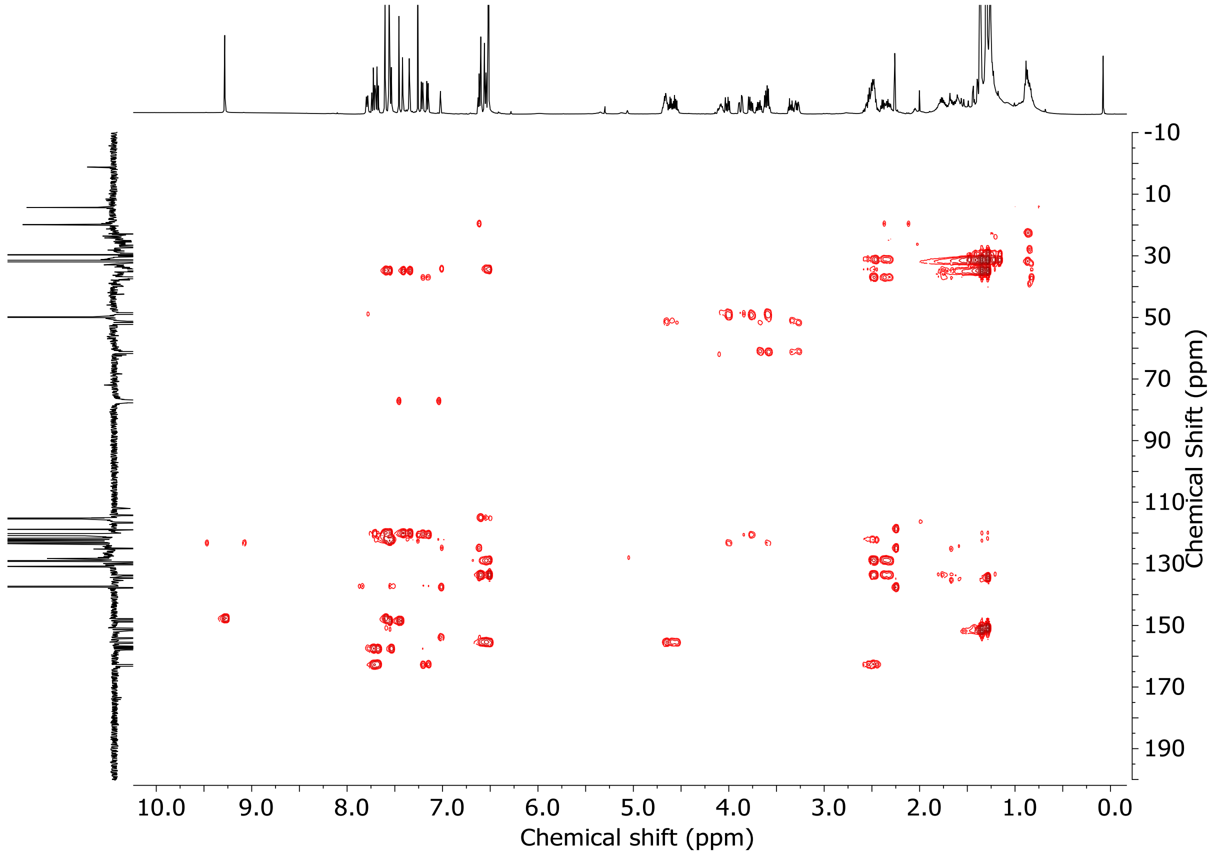

Figure S75. HMBC NMR ($CDCl_3$) of (S_{ma},$R_{co\text{-}c}$)-**4d**.

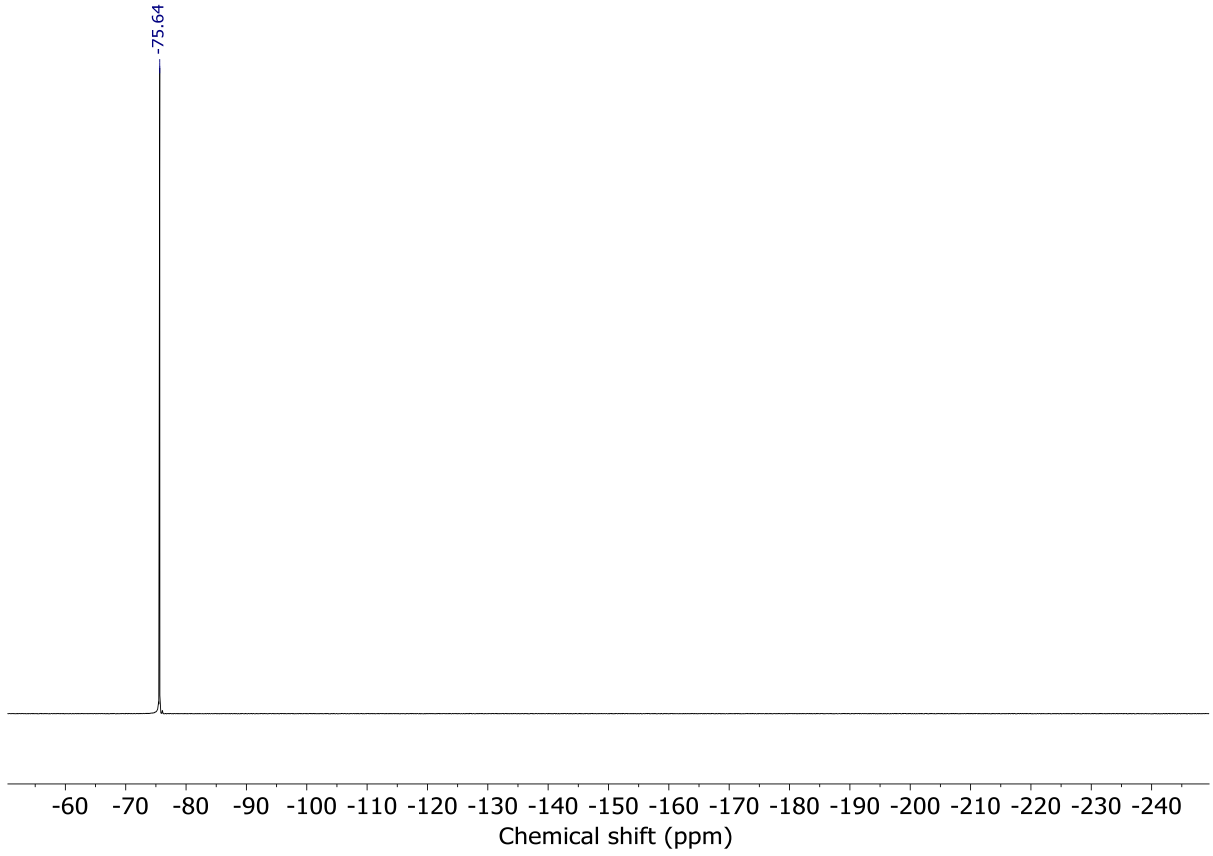

Figure S76. ^{19}F NMR ($CDCl_3$, 470 MHz) of (S_{ma},$R_{co\text{-}c}$)-**4d**.

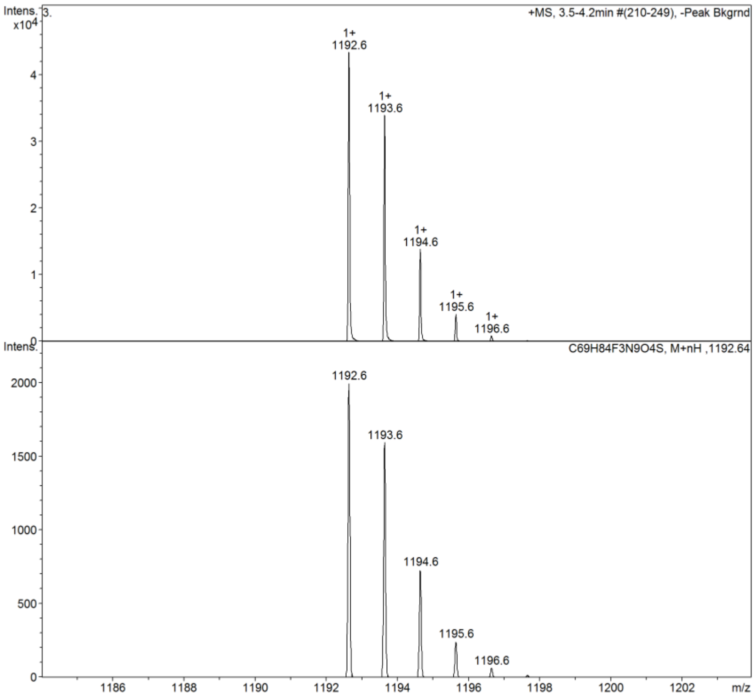

Figure S77. Observed (top) and calculated (bottom) isotopic patterns for (S_{ma},$R_{co\text{-}c}$)-**4d**.

Methylated trifluoroacetamide rotaxanes 4e

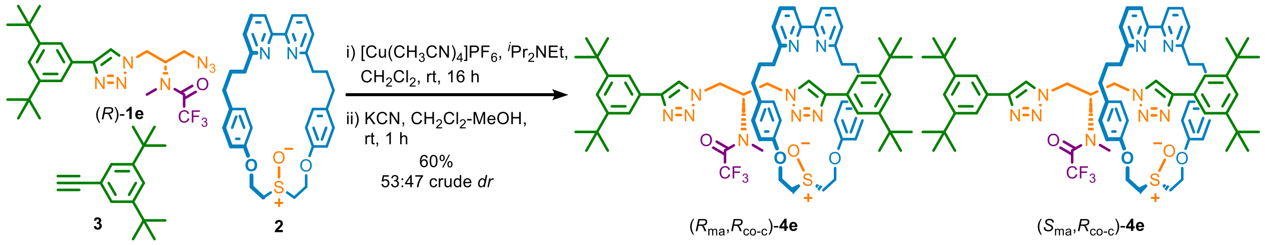

In a CEM vial were added the **3** (11.6 mg, 54.3 µmol), (*R*)-**1e** (25.3 mg, 54.3 µmol), **2** (26.0 mg, 49.4 µmol) and [Cu(CH_3CN)$_4$$PF_6$] (17.7 mg, 47.4 µmol). The vial was sealed and purged with N_2, then CH_2Cl_2 was added (1.5 mL), followed by $^iPr_2NEt$ (0.17 mL, 98.8 µmol). The solution was stirred at rt for 16 h. MeOH (2 mL mL) and KCN as a solid (31.9 mg, 0.49 mmol) were added and the resulting mixture was stirred vigorously for until colourless. The crude mixture was diluted with CH_2Cl_2 (5 mL) and washed with H_2O (5 mL) then EDTA-NH_3 (5 mL), with separation of aqueous and organic phases. The combined aqueous phase was then extracted with CH_2Cl_2 (3 x 5 mL) and the combined organic extracts were washed with brine (10 mL), dried ($MgSO_4$) and concentrated *in vacuo* to give a sample containing **4e** as a mixture of diastereomers (53 : 47 *dr*, Figure S78). Chromatography (CH_2Cl_2-CH_3CN 0→100%) gave **4e** as a colourless oil (36.0 mg, 60%) as a mixture of diastereoisomers (1.1 : 1, Figure S79) and a 97 : 3 mixture of rotamers (the rotameric nature of the compound is highlighted by the EXSY correlation peaks observed in the NOESY spectrum).

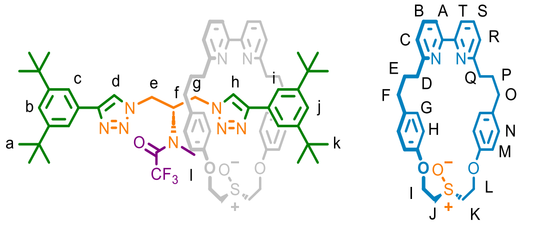

Major Diastereoisomer

^{1}H NMR (500 MHz, CDCl$_3$) δ: 9.44 (s, 1H, H_h), 7.80 (t, *J* = 7.9, 1H, H_B), 7.72-7.64 (m, 3H, H_i, H_S), 7.63-7.56 (m, 3H, H_A, H_c), 7.54 (s, 1H, H_d), 7.48-7.42 (m, 2H, H_b, H_T), 7.40 (t, *J* = 1.8, 1H, H_j), 7.30 (d, *J* = 7.9, 1H, H_C), 7.18 (d, *J* = 7.8, 1H, H_R), 6.72 (d, *J* = 8.6, 2H, H_G or H_M), 6.68 (d, *J* = 8.6, 2H, H_H or H_N), 6.62-6.56 (m, H_G and H_H or H_M and H_N), 4.81-4.40 (m, 5H, H_f, H_I, H_L), 4.20-4.07 (m, 1H, H_J or H_K), 4.07-3.87 (m, 2H, H_g and H_J or H_K), 3.83-3.48 (m, 3H, H_e, $H_{g'}$), 3.27-3.16 (m, 2H, $H_{J'}$ or $H_{K'}$), 2.69-2.24 (m, 11H, H_D, H_F, H_l, H_O, H_Q), 1.93-1.54 (m, 4H, H_E, H_P), 1.39 (s, 18H, H_k), 1.37 (s, 18H, H_a)

^{19}F NMR (470 MHz, CDCl$_3$) δ: 70.79

HR-ESI-MS (+ve) m/z = 1206.6515 [M+H]$^+$ (calc. 1206.7 m/z for $C_{70}H_{87}F_3N_9O_4S$ 1206.6554);

Minor Diastereoisomer

^{1}H NMR (500 MHz, CDCl$_3$) δ: 8.81 (s, 1H, H_h), 7.75 (t, *J* = 7.8, 1H, H_B), 7.72-7.64 (m, 3H, H_i, H_S), 7.63-7.56 (m, 3H, H_A, H_c), 7.49 (s, 1H, H_d), 7.48-7.42 (m, 1H, H_T), 7.41 (t, *J* = 1.9, 1H, H_b), 7.35 (t, *J* = 1.9, 1H, H_j), 7.23 (d, *J* = 7.7, 1H, H_C), 7.20 (d, *J* = 7.8, 1H, H_R), 6.54-6.49 (m, H_G and H_H or H_M and H_N), 6.46-6.38 (m, H_G and H_H or H_M and H_N), 4.81-4.40 (m, 6H, H_e, H_f, H_I, H_L), 4.20-4.07 (m, 2H, H_g), 4.07-3.87 (m, 1H, H_J or H_K), 3.83-3.48 (m, 2H, $H_{e'}$ and H_J or H_K), 3.12-3.01 (m, 2H, $H_{J'}$ or $H_{K'}$), 2.69-2.24 (m, 8H, H_D, H_F, H_O, H_Q), 1.98 (bs, 3H, H_l), 1.93-1.54 (m, 4H, H_E, H_P), 1.36 (s, 18H, H_a), 1.27 (s, 18H, H_k)

^{19}F NMR (470 MHz, CDCl$_3$) δ: 70.69

HR-ESI-MS (+ve) m/z = 1206.6515 [M+H]$^+$ (calc. 1206.7 m/z for $C_{70}H_{87}F_3N_9O_4S$ 1206.6554);

It was not possible to attribute each carbon peak to a single isomer unambiguously, so the complete list of observed peaks is reported below.

^{13}C NMR (126 MHz, CDCl$_3$) δ: 162.9, 162.9, 162.8, 162.8, 157.9, 157.8, 157.8, 157.6, 157.2 (q, J_{C-F} = 36.0) (x2), 155.9, 155.8, 155.0, 155.0, 151.5, 151.3, 151.0, 151.0, 148.6, 148.2, 147.8, 147.3, 137.6, 137.3, 137.2, 137.2, 133.7, 133.5, 133.5, 133.4, 130.2, 130.1, 129.6, 129.4, 129.3, 129.3, 128.8, 128.6, 123.3, 122.7, 122.5, 122.4, 122.4, 122.3, 122.0, 122.0, 121.8, 121.7, 120.8, 120.6, 120.5, 120.5, 120.3, 120.2, 120.2, 120.1, 120.1, 120.0, 119.8, 115.5 (q, J_{C-F} = 289.9), 115.5 (q, J_{C-F} = 289.9), 115.1, 115.1, 114.9, 114.6, 61.3, 61.0, 60.9, 60.8, 53.4, 53.3, 52.4, 51.2, 47.8, 47.2, 47.1, 46.3, 37.6, 37.5, 37.1, 37.1, 36.4 (HMBC), 35.0, 35.0, 34.9, 34.7, 34.7, 34.7, 34.4, 32.4, 31.7, 31.6, 31.5, 31.5, 31.4, 31.4, 31.0, 30.3, 29.7.

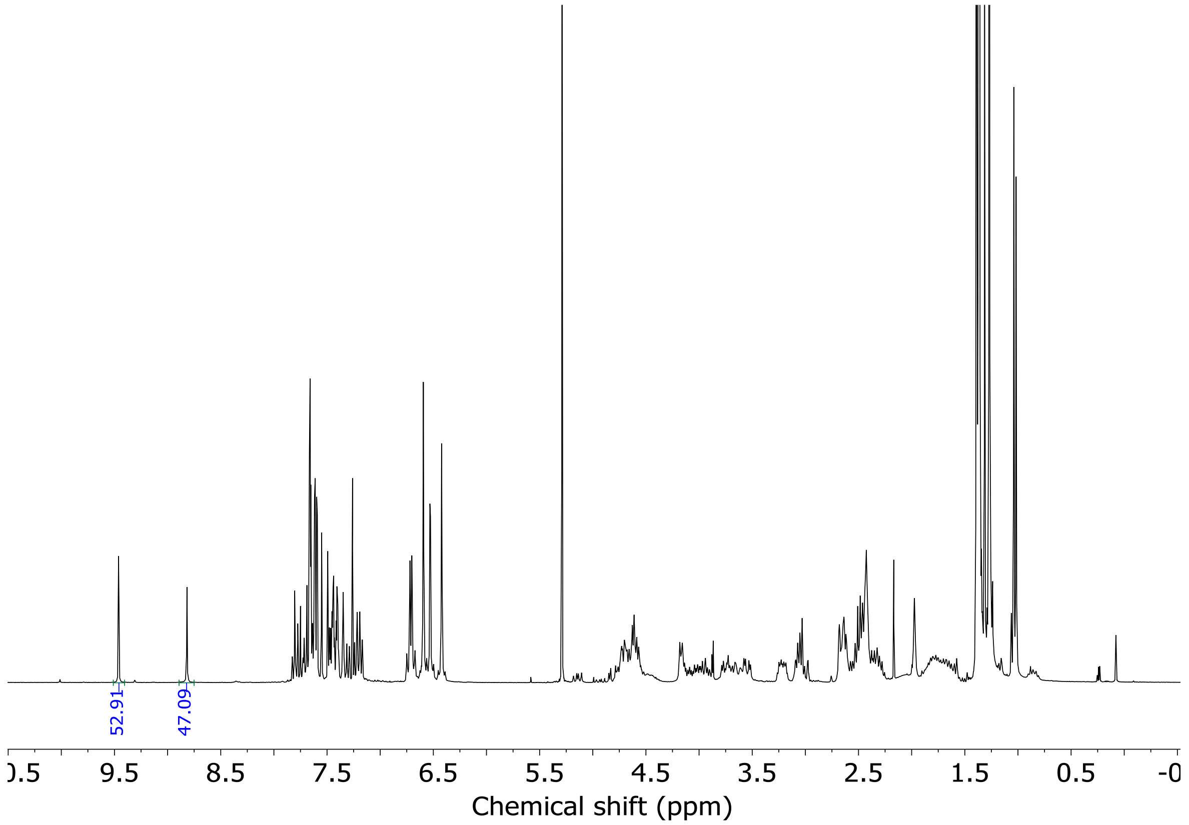

Figure S78. ^{1}H NMR ($CDCl_3$, 400 MHz) of (R_{ma},R_{co-c})-**4e** and (S_{ma},R_{co-c})-**4e** prior to purification (97 : 3 mixture of rotamers, 53: 47 *dr*).

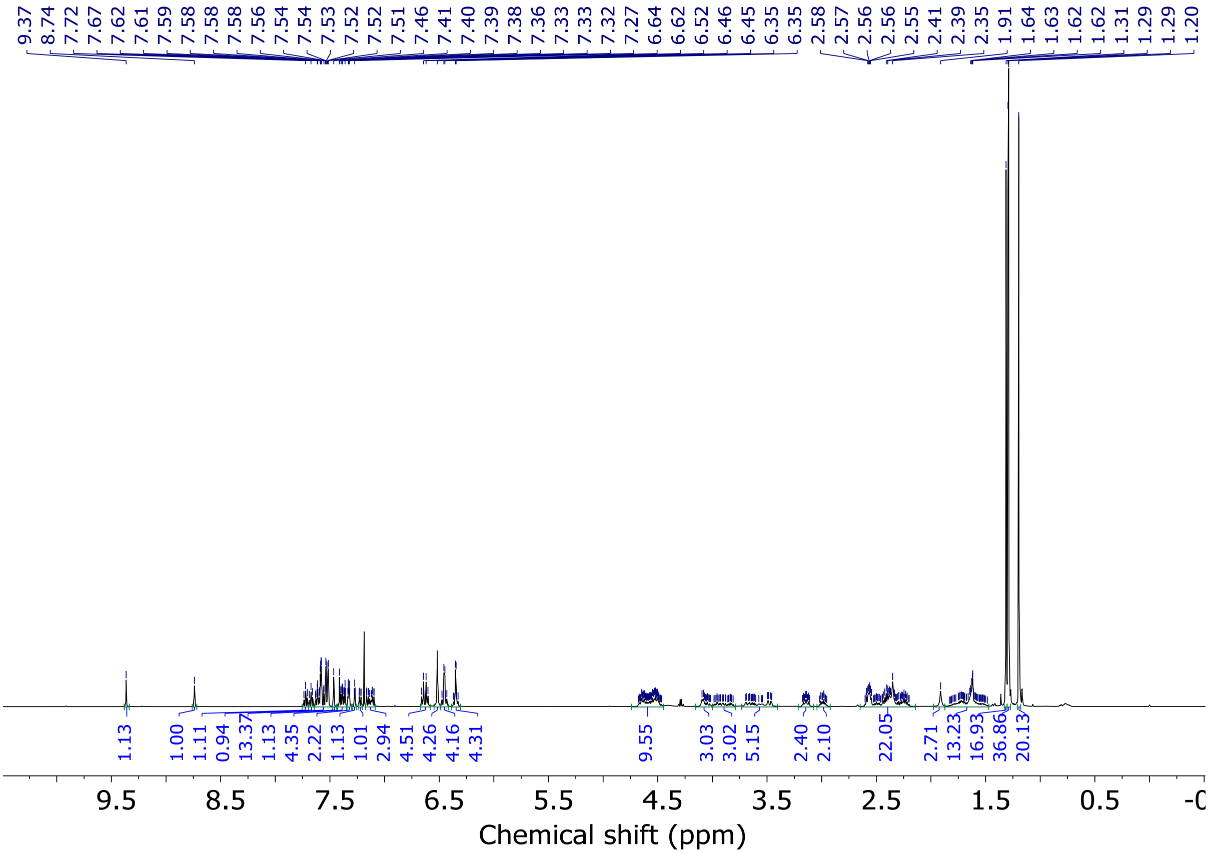

Figure S79. ^{1}H NMR ($CDCl_3$, 500 MHz) of (R_{ma},R_{co-c})-**4e** and (S_{ma},R_{co-c})-**4e** (97 : 3 mixture of rotamers, 53: 47 *dr*).

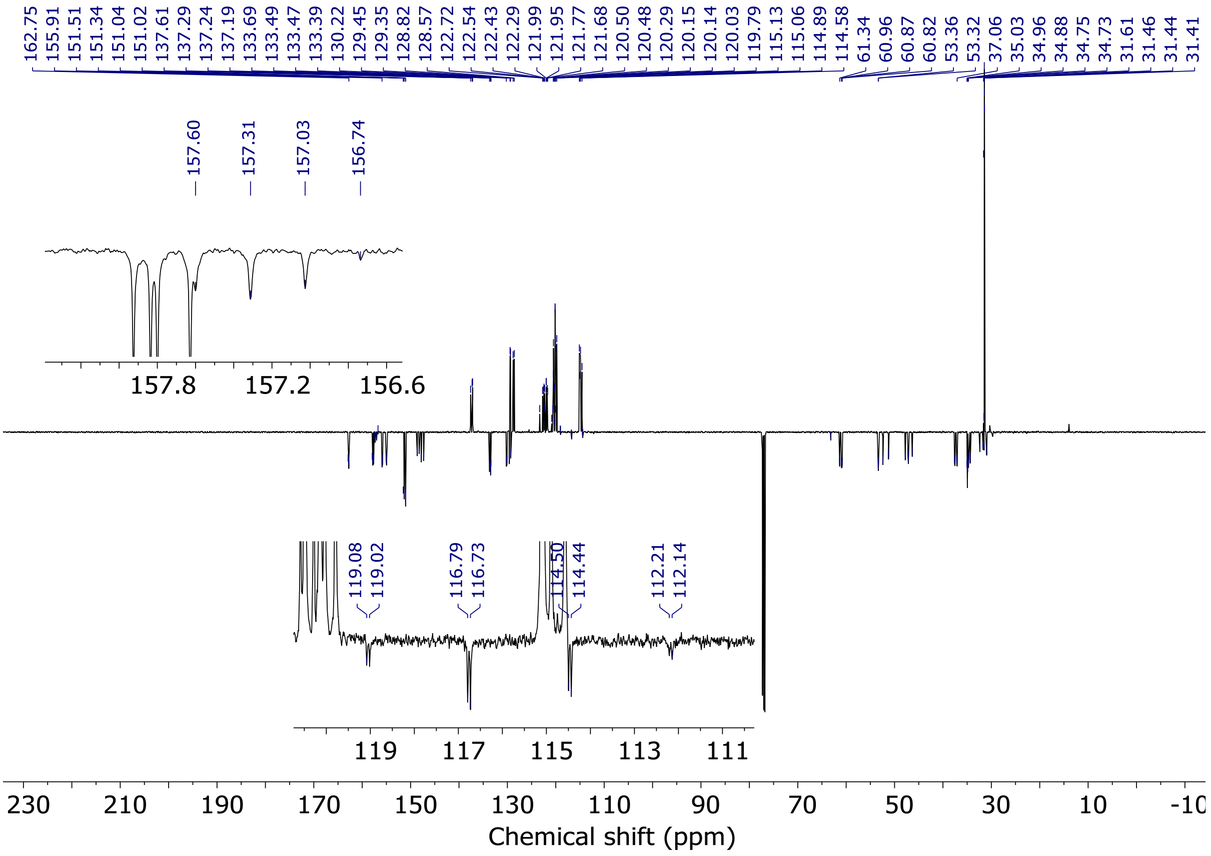

Figure S80. JMOD NMR ($CDCl_3$, 126 MHz) of (R_{ma},$R_{co\text{-}c}$)-**4e** and (S_{ma},$R_{co\text{-}c}$)-**4e** (97 : 3 mixture of rotamers, 53: 47 *dr*).

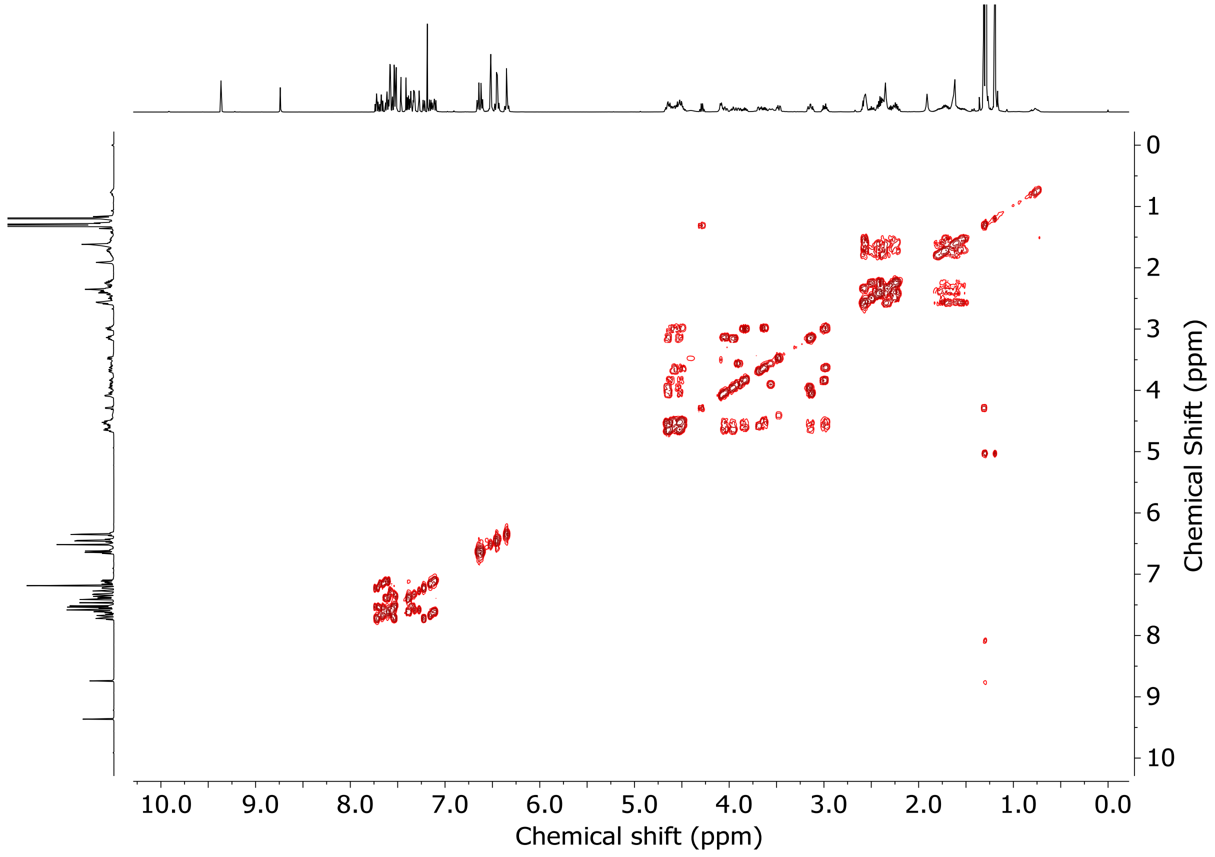

Figure S81. COSY NMR ($CDCl_3$) of (R_{ma},$R_{co\text{-}c}$)-**4e** and (S_{ma},$R_{co\text{-}c}$)-**4e** (97 : 3 mixture of rotamers, 53: 47 *dr*).

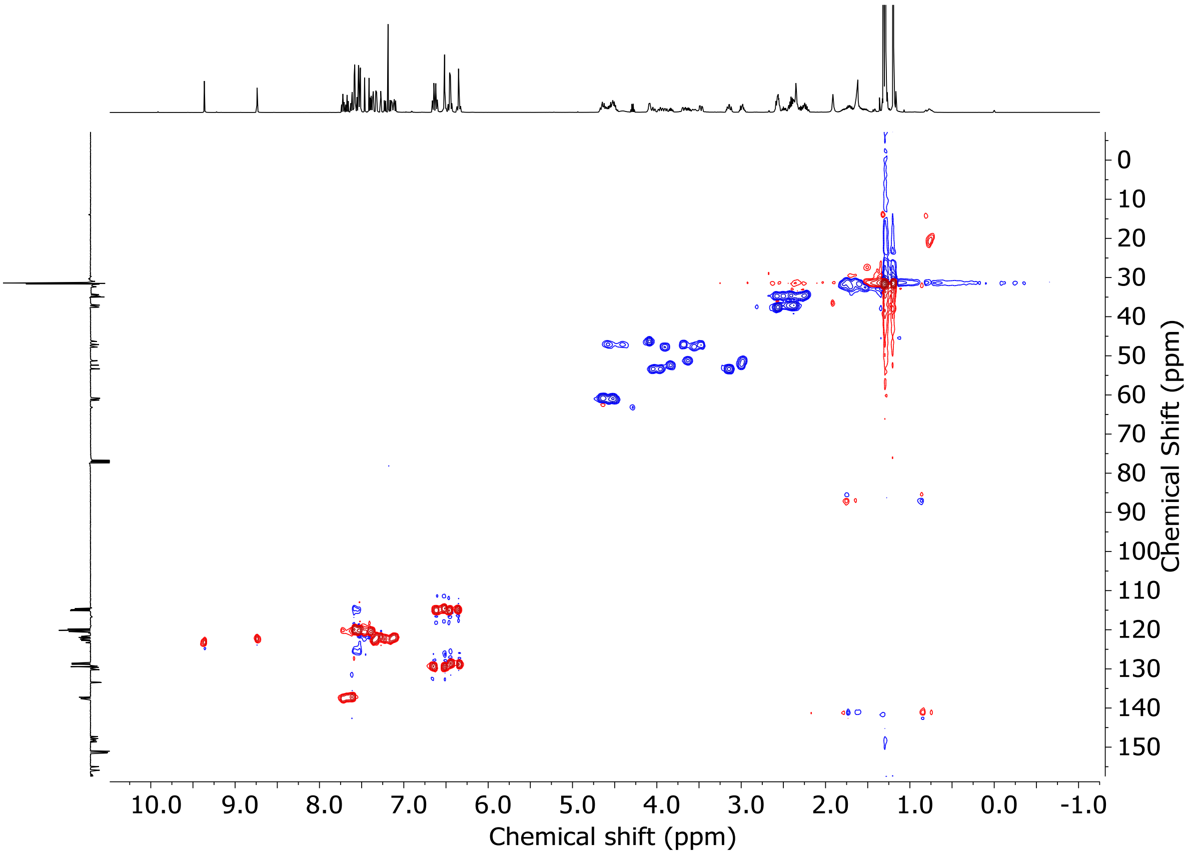

Figure S82. HSQC NMR ($CDCl_3$) of (R_{ma},$R_{co\text{-}c}$)-**4e** and (S_{ma},$R_{co\text{-}c}$)-**4e** (97 : 3 mixture of rotamers, 53: 47 *dr*).

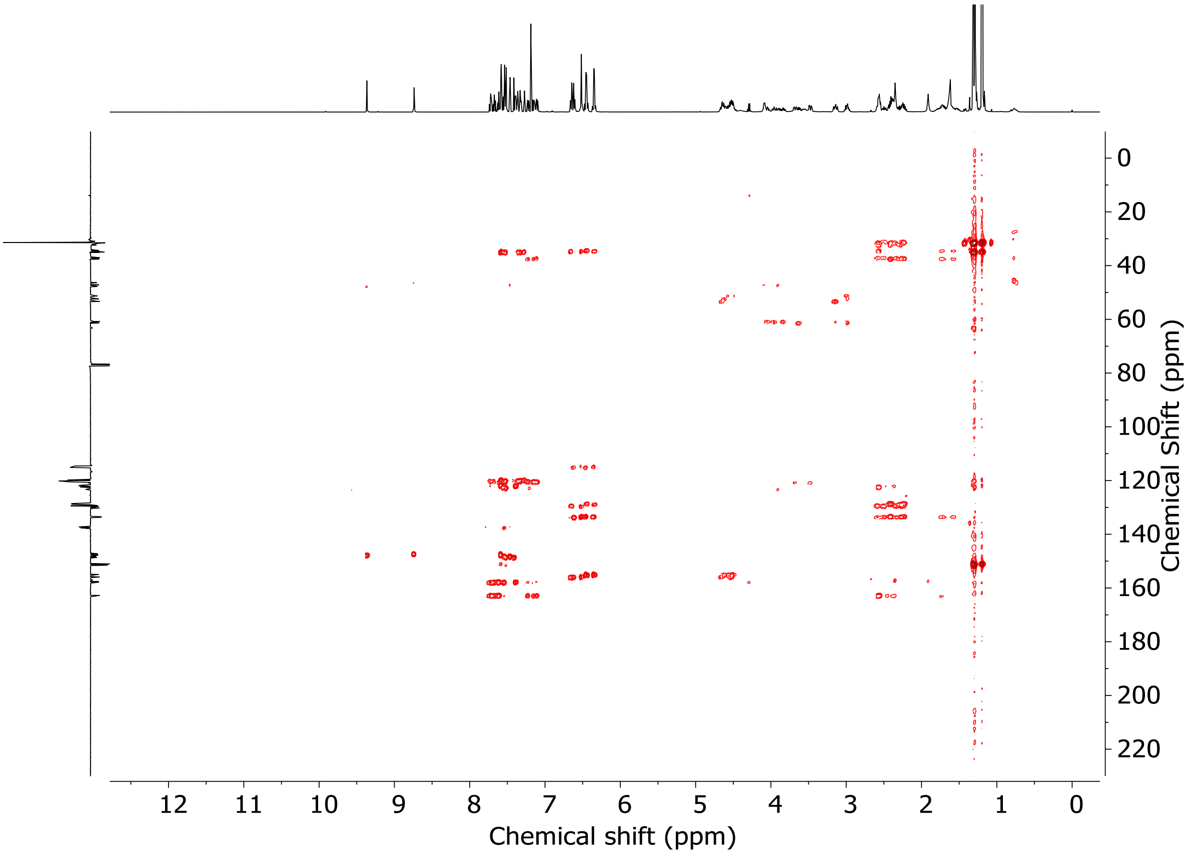

Figure S83. HMBC NMR ($CDCl_3$) of (R_{ma},$R_{co\text{-}c}$)-**4e** and (S_{ma},$R_{co\text{-}c}$)-**4e** (97 : 3 mixture of rotamers, 53: 47 *dr*).

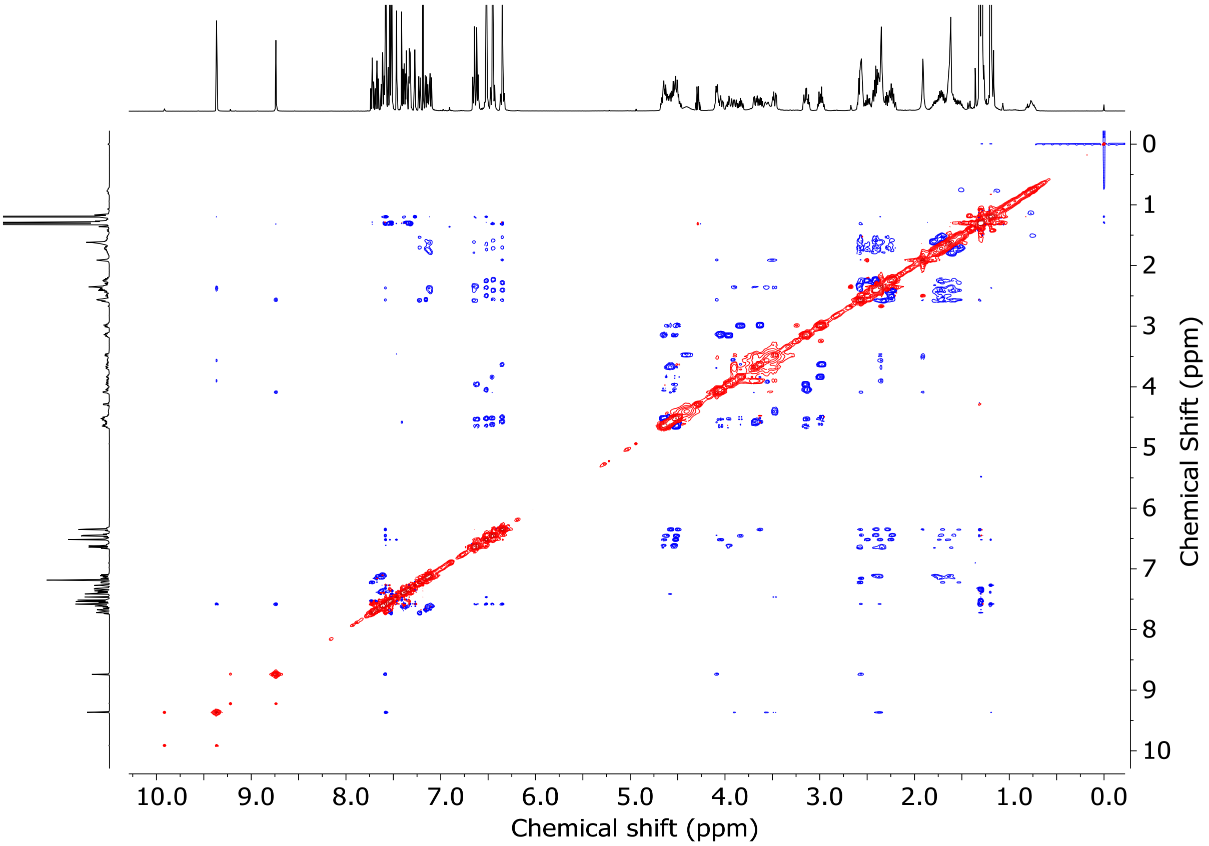

Figure S84. NOESY NMR ($CDCl_3$) of (R_{ma},$R_{co\text{-}c}$)-**4e** and (S_{ma},$R_{co\text{-}c}$)-**4e** (97 : 3 mixture of rotamers, 53: 47 *dr*).

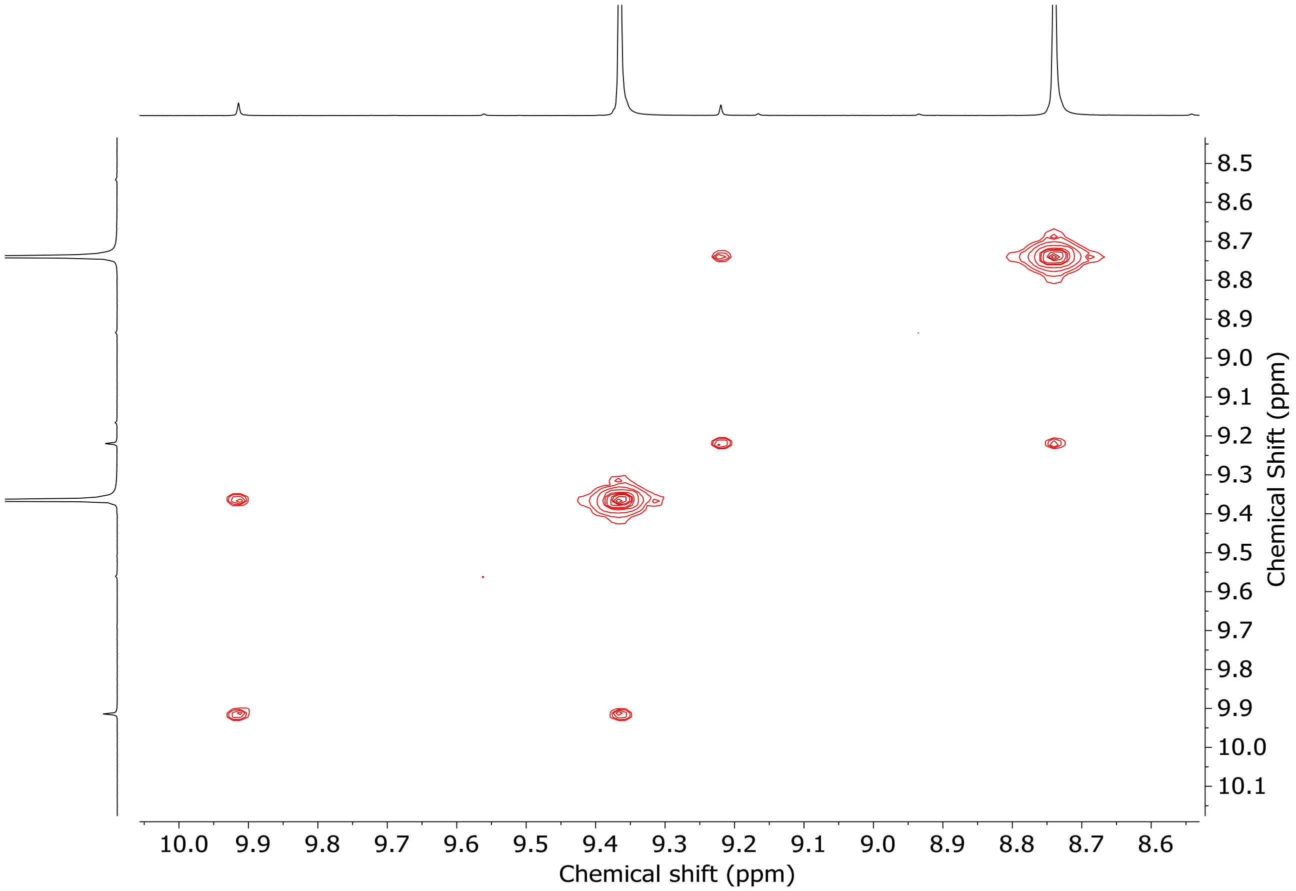

Figure S85. Expansion of NOESY NMR (Figure S84) of **4e** highlighting the EXSY cross-peaks observed.

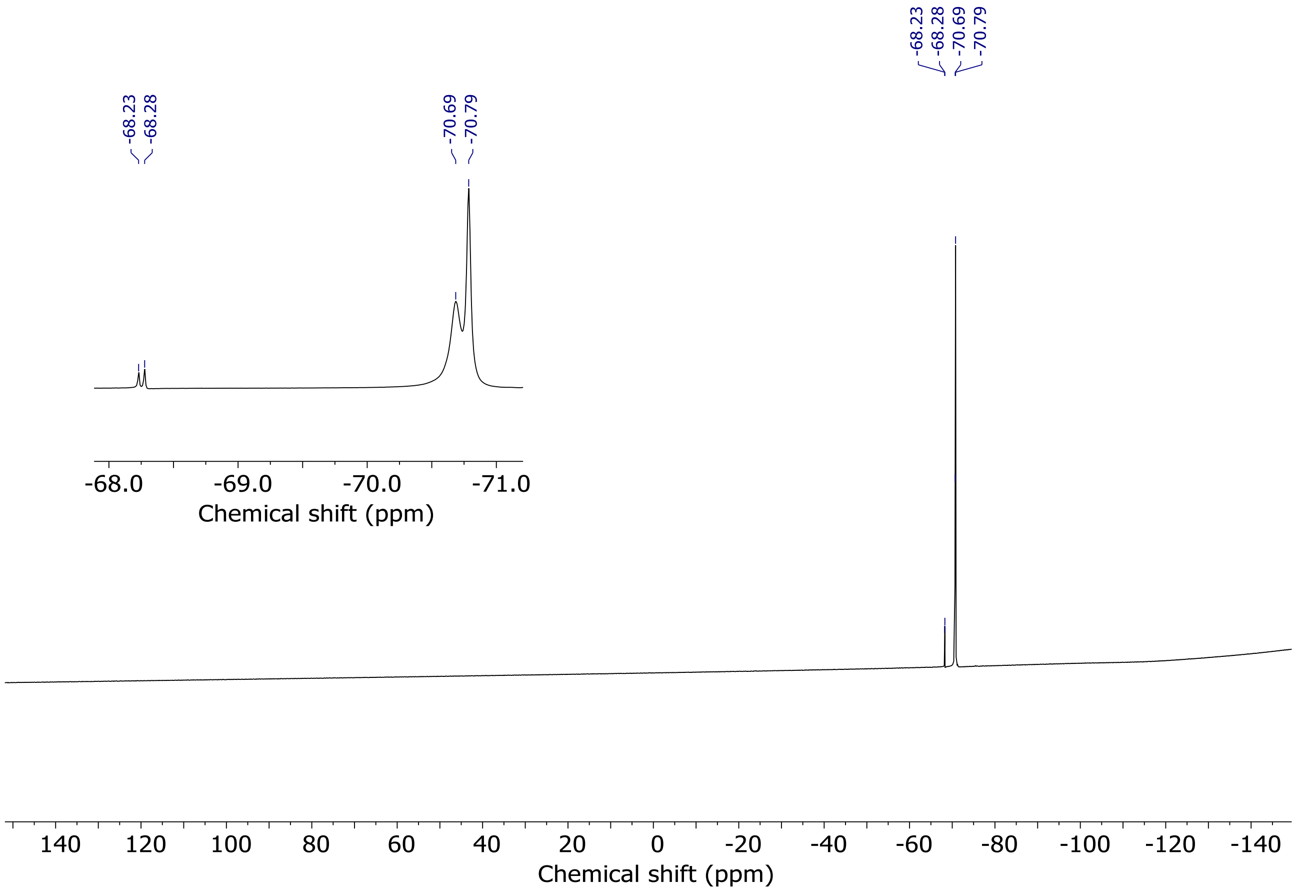

Figure S86. ^{19}F NMR ($CDCl_3$, 470 MHz) of (R_{ma},$R_{co\text{-}c}$)-**4e** and (S_{ma},$R_{co\text{-}c}$)-**4e** (97 : 3 mixture of rotamers, 53: 47 *dr*).

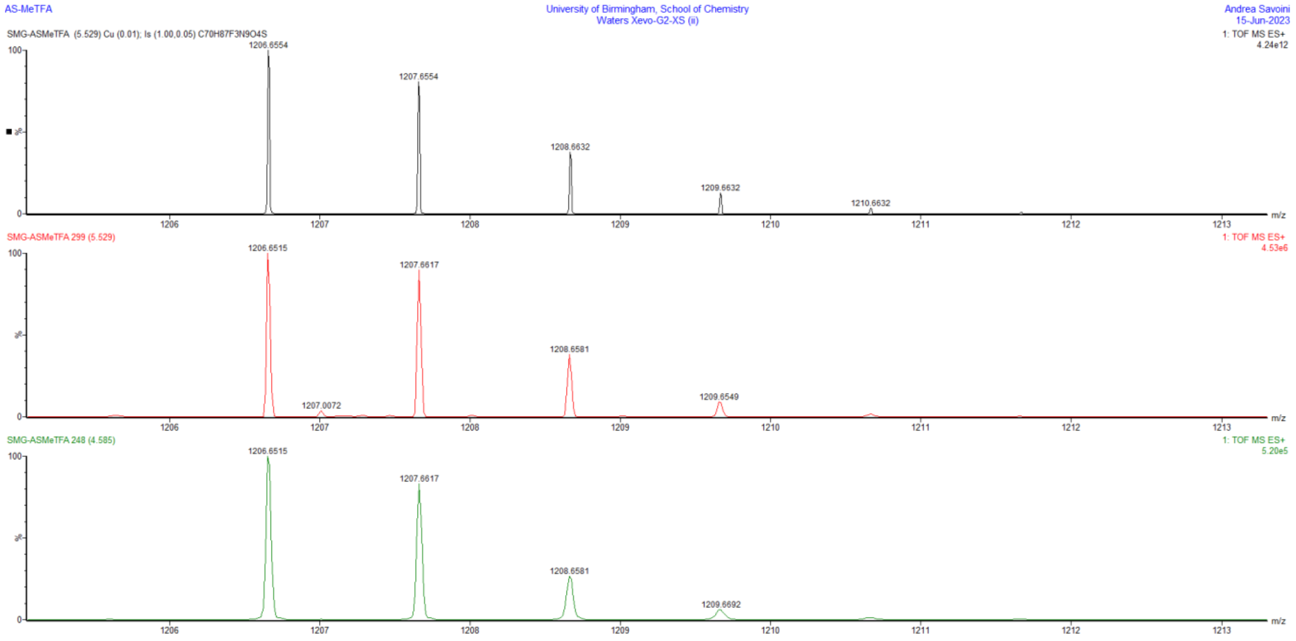

Figure S87. Calculated (top) and observed (middle, bottom) isotopic patterns for rotaxanes **4e**.

4. Synthesis of rotaxanes 6-12 and associated compounds (Scheme 3, main text)

Rotaxanes (E_m)-6 and (Z_m)-6

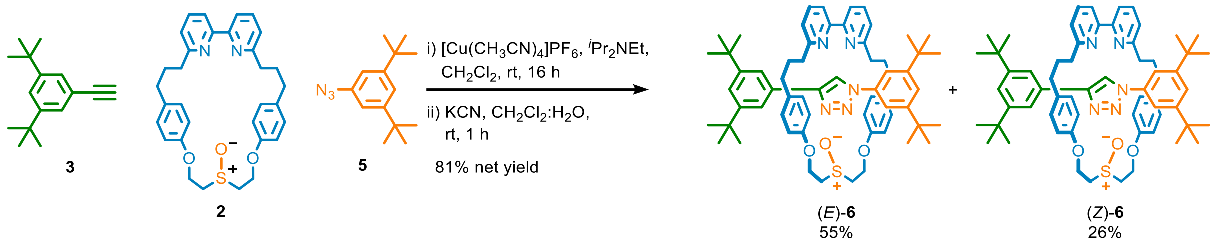

In a CEM vial were added **3** (9.0 mg, 41.7 µmol), **5** (9.7 mg, 41.7 µmol), **2** (20.0 mg, 38.0 µmol) and [$Cu(CH_3CN)_4PF_6$] (13.6 mg, 36.5 µmol) the vial was sealed and purged with N_2, then CH_2Cl_2 was added (1.0 mL), followed by $^i Pr_2NEt$ (13.3 µL, 75.9 µmol). The solution was stirred at rt for 16 h. MeOH (2 mL) and KCN as a solid (24.7 mg, 0.38 mmol) were added and the resulting mixture was stirred vigorously until colourless. The mixture was diluted with CH_2Cl_2 (5 mL) and washed with H_2O (5 mL) then EDTA-NH_3 (5 mL), with separation of aqueous and organic phases. The combined aqueous phase was then extracted with CH_2Cl_2 (3 x 5 mL) and the combined organic extracts were washed with brine (10 mL), dried ($MgSO_4$) and concentrated *in vacuo* to give a residue containing rotaxane **6** (28% de, Figure S88). Chromatography (CH_2Cl_2-CH_3CN 0→30%) gave (*E*)-**6** (20.4 mg, 55%) as a white foam and (*Z*)-**6** (9.7 mg, 26%) as a white foam.

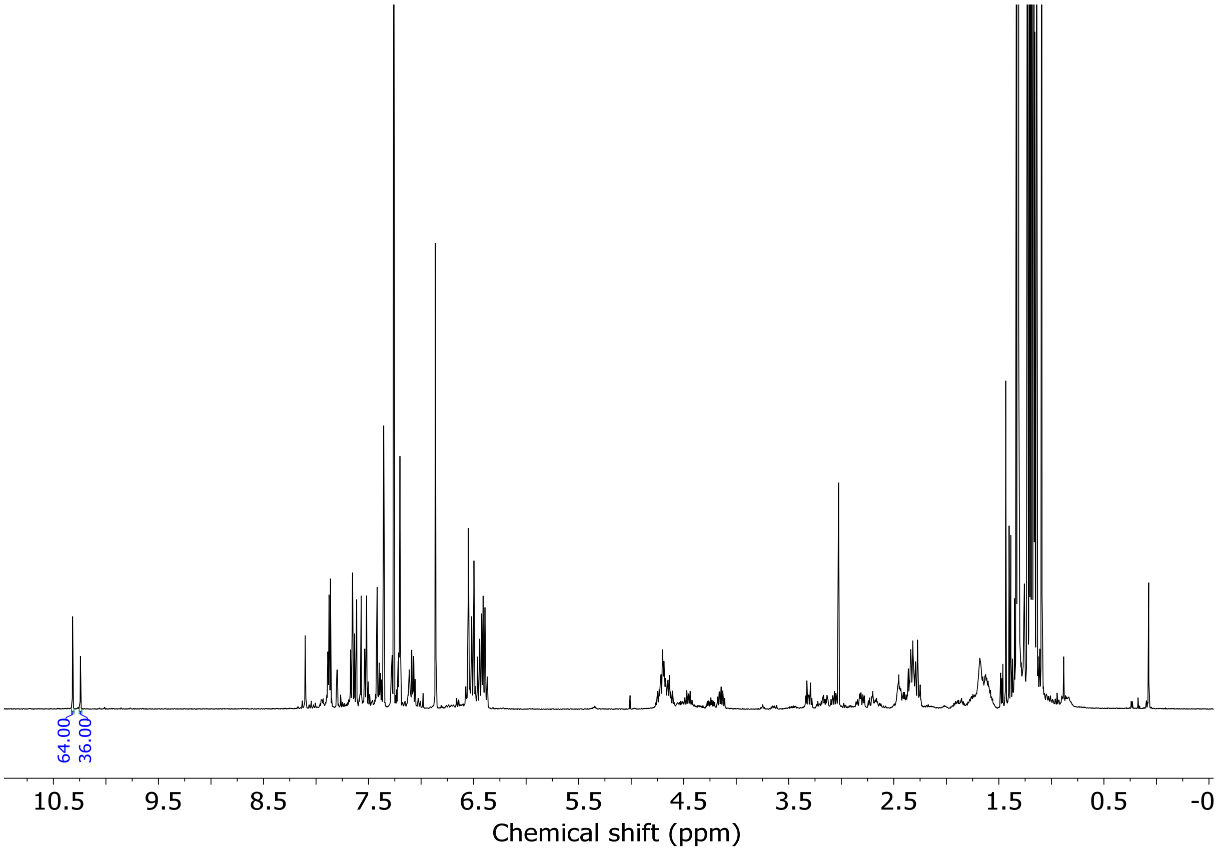

Figure S88: ^{1}H NMR ($CDCl_3$, 400 MHz) of (E_m)-**6** and (Z_m)-**6** prior to chromatography.

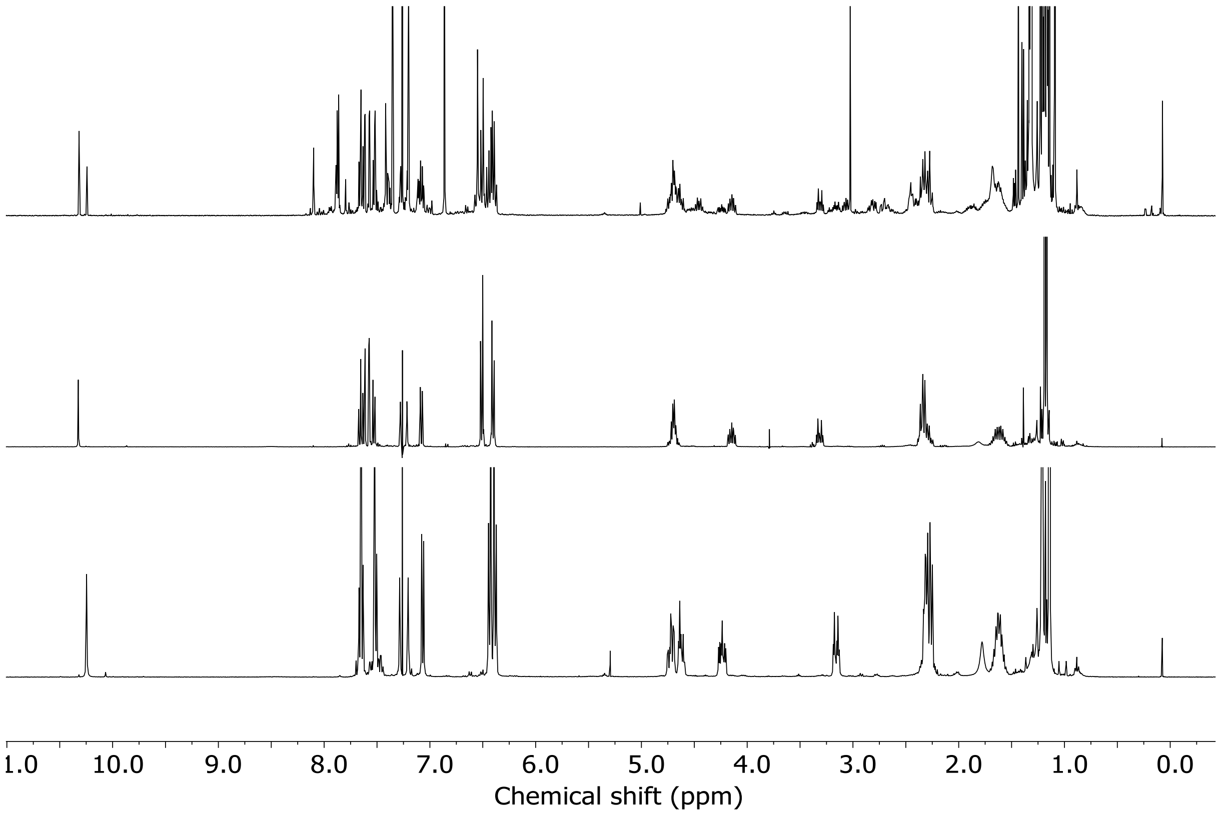

Figure S89: ^{1}H NMR ($CDCl_3$, 400 MHz) of (E_m)-**6** and (Z_m)-**6** prior to chromatography (top), (E_m)-**6** (middle) and (Z_m)-**6** (bottom).

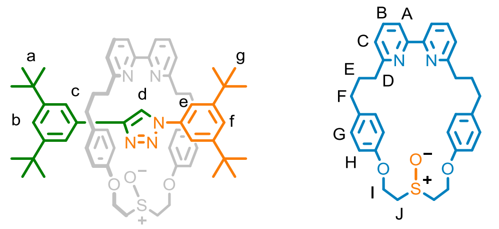

Rotaxane (E_m)-**6**

^{1}H NMR (400 MHz, $CDCl_3$) δ 10.32 (s, 1H, H_d), 7.65 (t, J = 7.8, 2H, H_B), 7.61 (d, J = 1.6, 2H, H_e), 7.58 (d, J = 1.7, 2H, H_c), 7.53 (d, J = 7.7, 2H, H_A), 7.31-7.25 (m, 1H, H_f), 7.24-7.19 (m, 1H, H_b), 7.08 (d, J = 7.6, 2H, H_C), 6.51 (d, J = 8.5, 4H, H_G), 6.40 (d, J = 8.4, 4H, H_H), 4.76-4.59 (m, 4H, H_I), 4.15 (dt, J = 13.4, 6.8, 2H, H_J), 3.31 (dt, J = 13.2, 5.2, 2H, H_J), 2.45 – 2.23 (m, 8H, H_D, H_F), 1.63 (dq, J = 13.9, 8.0, 4H, H_E), 1.19 (s, 19H, H_a), 1.17 (s, 18H, H_g).

^{13}C NMR (101 MHz, $CDCl_3$) δ 163.5, 157.5, 155.2, 151.8, 150.4, 147.3, 137.3, 136.9, 133.0, 130.9, 128.7, 128.7, 122.0, 121.7, 121.1, 120.7, 120.5, 120.0, 114.9, 114.9, 114.6, 60.6, 52.0, 37.2, 35.1, 35.0, 34.9, 31.6, 31.3, 31.1.

HR-ESI-MS (+ve) m/z = 972.5824 $[M+H]^+$ (calc. m/z for $C_{62}H_{78}N_5O_3S$ 972.5820).

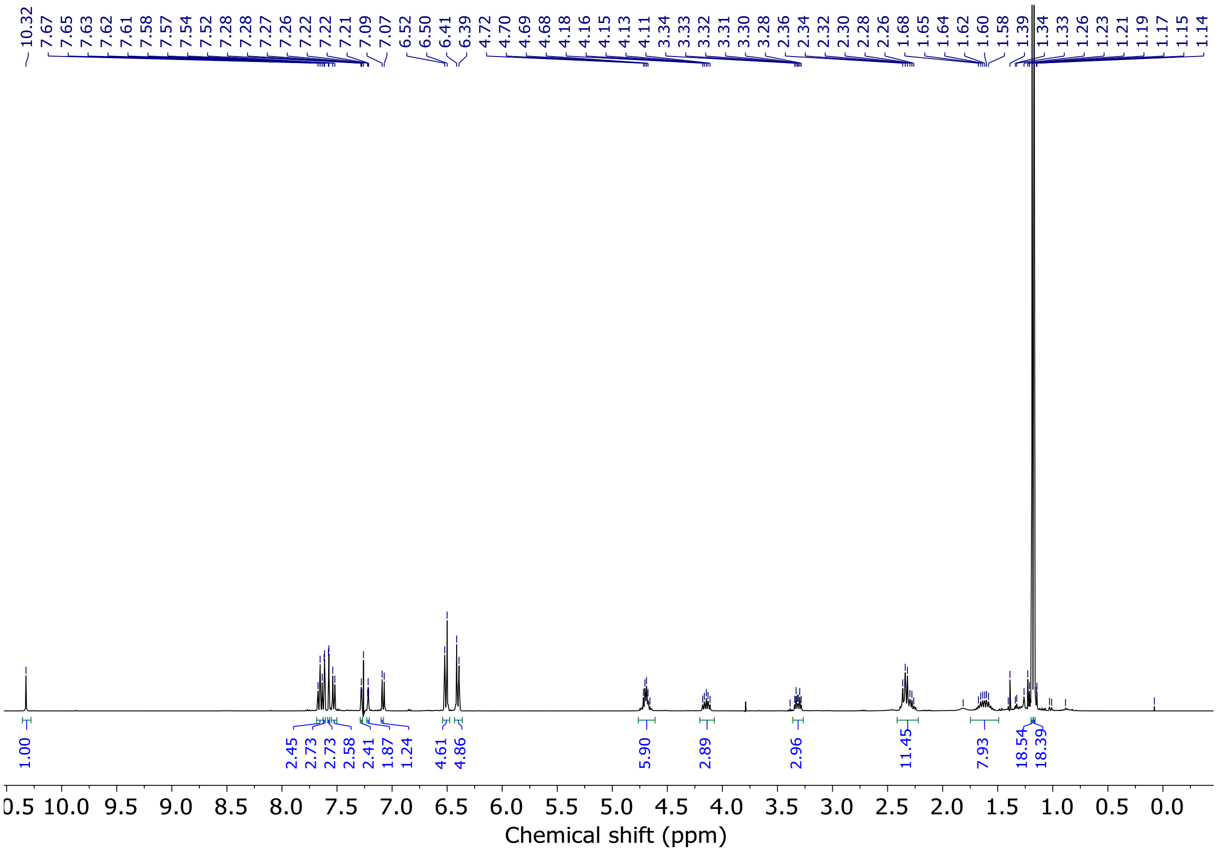

Figure S90: ^{1}H NMR ($CDCl_3$, 400 MHz) of (E_m)-**6**.

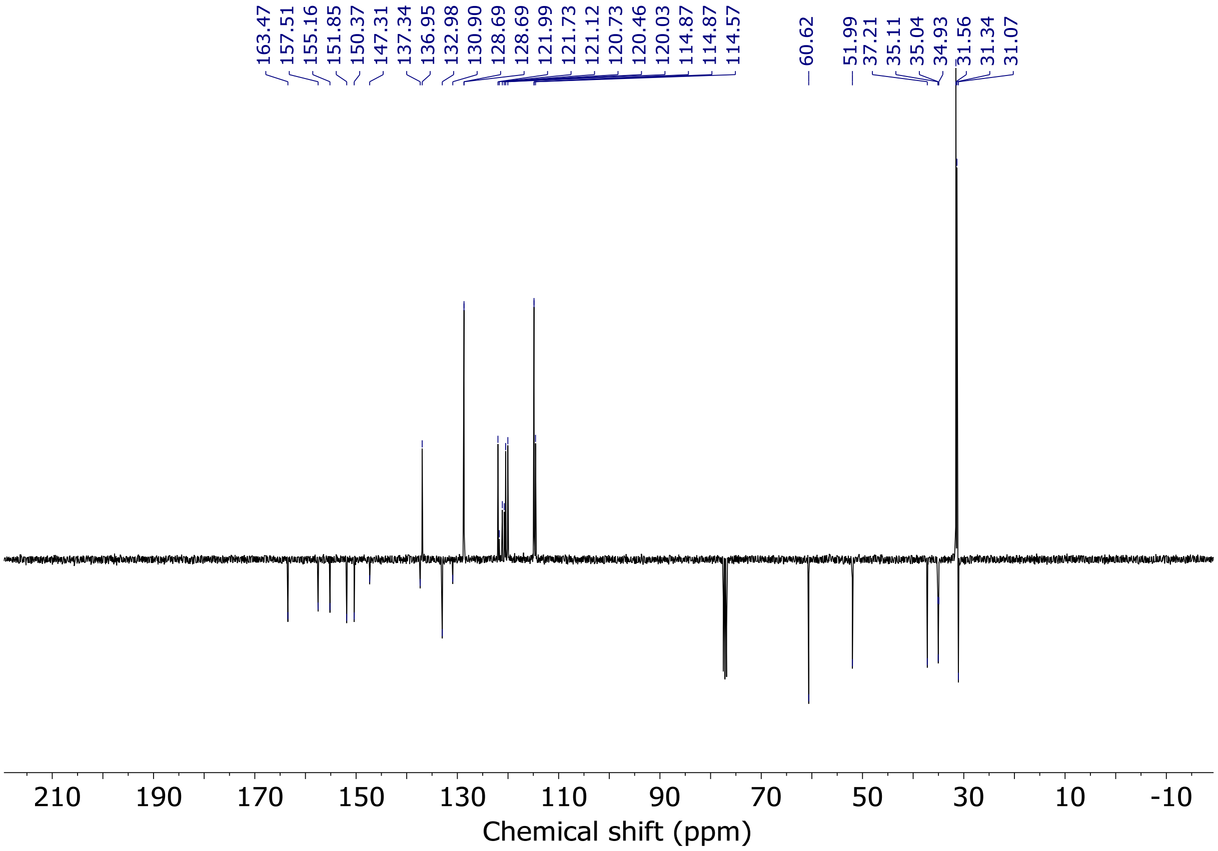

Figure S91: JMOD NMR ($CDCl_3$, 101 MHz) of (E_m)-**6**.

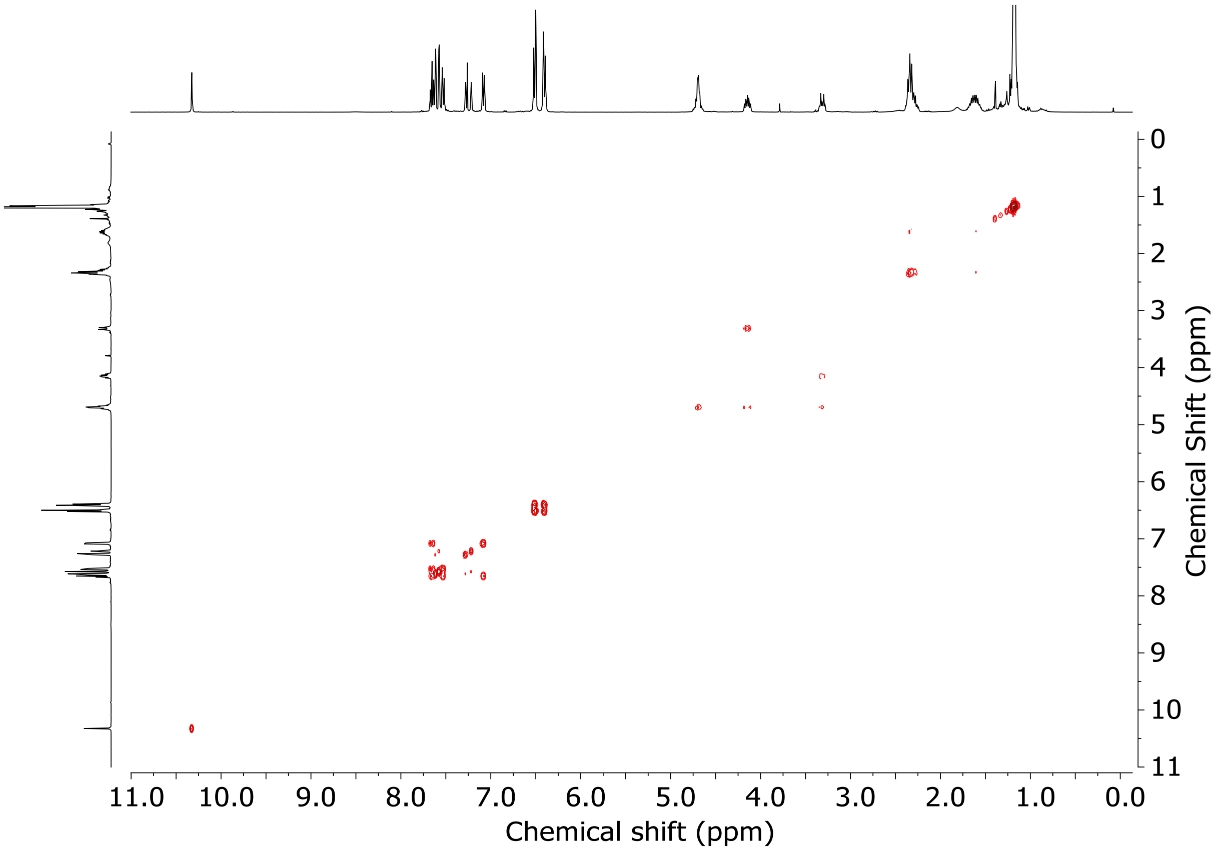

Figure S92: COSY NMR ($CDCl_3$) of (E_m)-**6**.

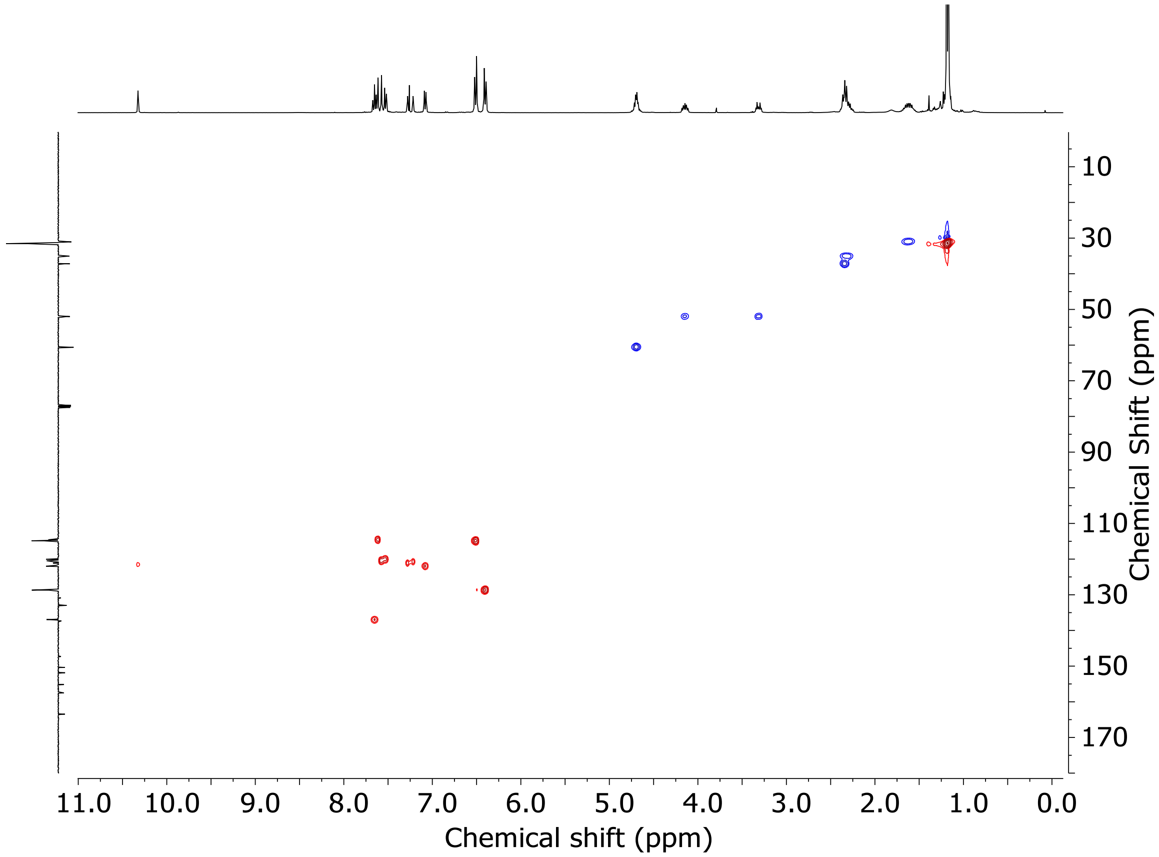

Figure S93: HSQC NMR ($CDCl_3$) of (E_m)-**6**.

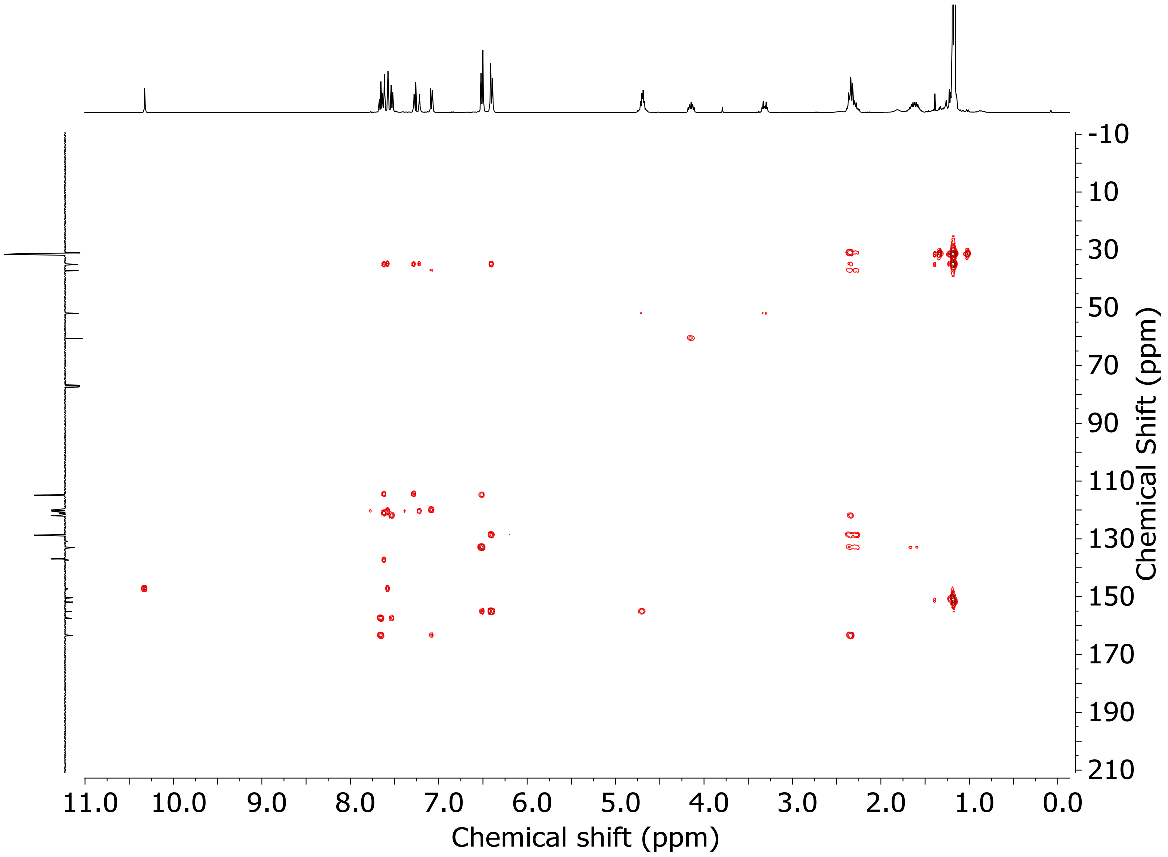

Figure S94: HMBC NMR ($CDCl_3$) of (E_m)-**6**.

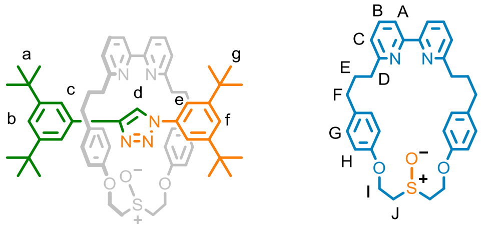

Rotaxane (Z_m)-**6**

^{1}H NMR (400 MHz, CDCl$_3$) δ 10.25 (s, 1H, H_d), 7.72-7.61 (m, 4H, H_B, H_c), 7.54-7.50 (m, 4H, H_A, H_e), 7.29 (t, J = 1.5, 1H, H_b), 7.21 (t, J = 1.5, 1H, H_f), 7.07 (d, J = 7.6, 2H, H_C), 6.43 (d, J = 8.7, 4H, H_G), 6.38 (d, J = 8.7, 4H, H_H), 4.80-4.68 (m, 2H, H_I), 4.66-4.57 (m, 2H, H_I), 4.24 (ddd, J = 13.5, 9.3, 4.5, 2H, H_J), 3.16 (dt, J = 13.2, 4.2, 2H, H_J), 2.28 (dd, J = 17.9, 9.2, 8H, H_D, H_F), 1.62 (dd, J =16.3, 6.2, 4H, H_E), 1.21 (s, 18H, H_a), 1.14 (s, 18H, H_g).

^{13}C NMR (101 MHz, CDCl$_3$) δ 163.6, 157.6, 155.1, 151.6, 150.6, 147.1, 137.2, 137.0, 132.7, 132.3, 132.2, 132.1, 132.1, 131.1, 128.8, 128.8, 128.7, 128.6, 122.1, 121.1, 120.9, 120.1, 120.0, 114.7, 114.5, 60.5, 52.9, 37.3, 35.2, 35.2, 34.8, 31.5, 31.5, 31.5.

HR-ESI-MS (+ve) m/z = 972.5804 $[M+H]^+$ (calc. m/z for $C_{62}H_{78}N_5O_3S$ 972.5820).

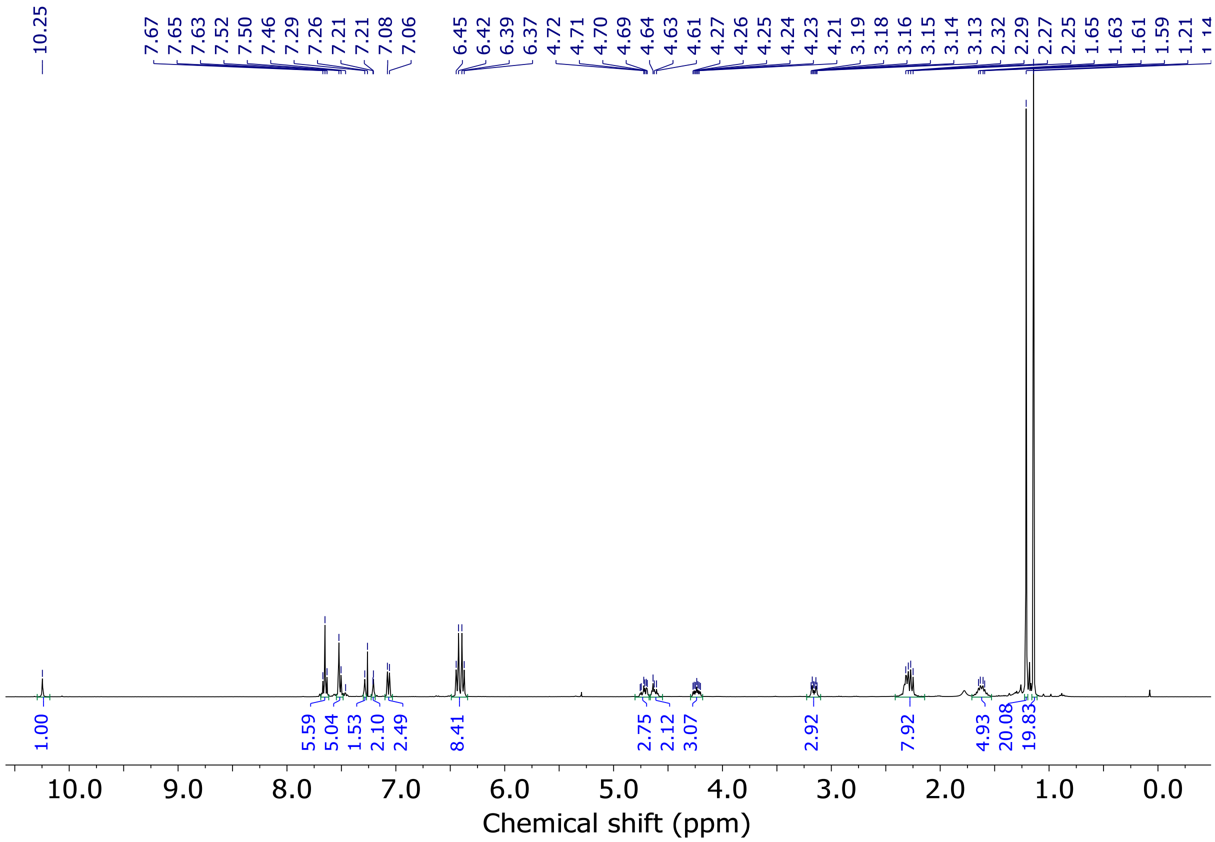

Figure S95: ^{1}H NMR ($CDCl_3$, 400 MHz) of (Z_m)-**6**.

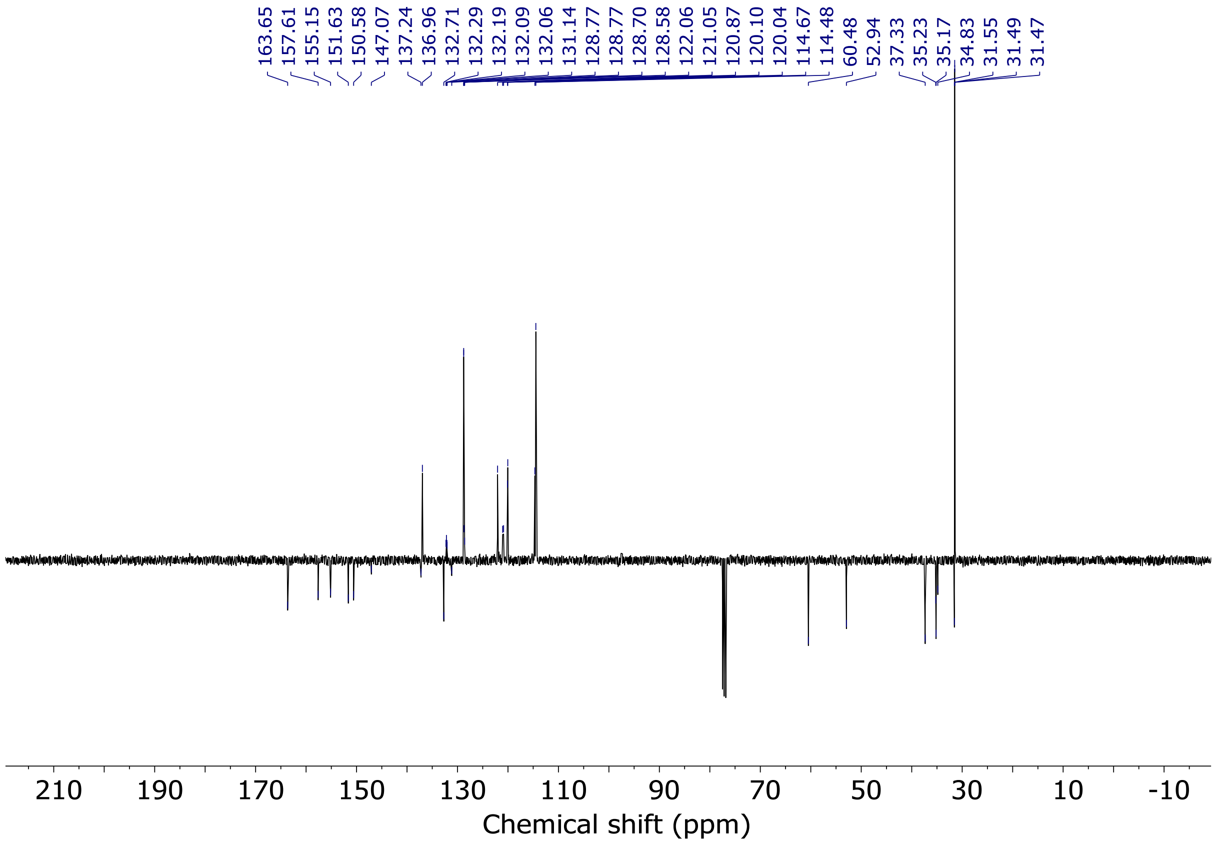

Figure S96: JMOD NMR ($CDCl_3$, 101 MHz) of (Z_m)-**6**.

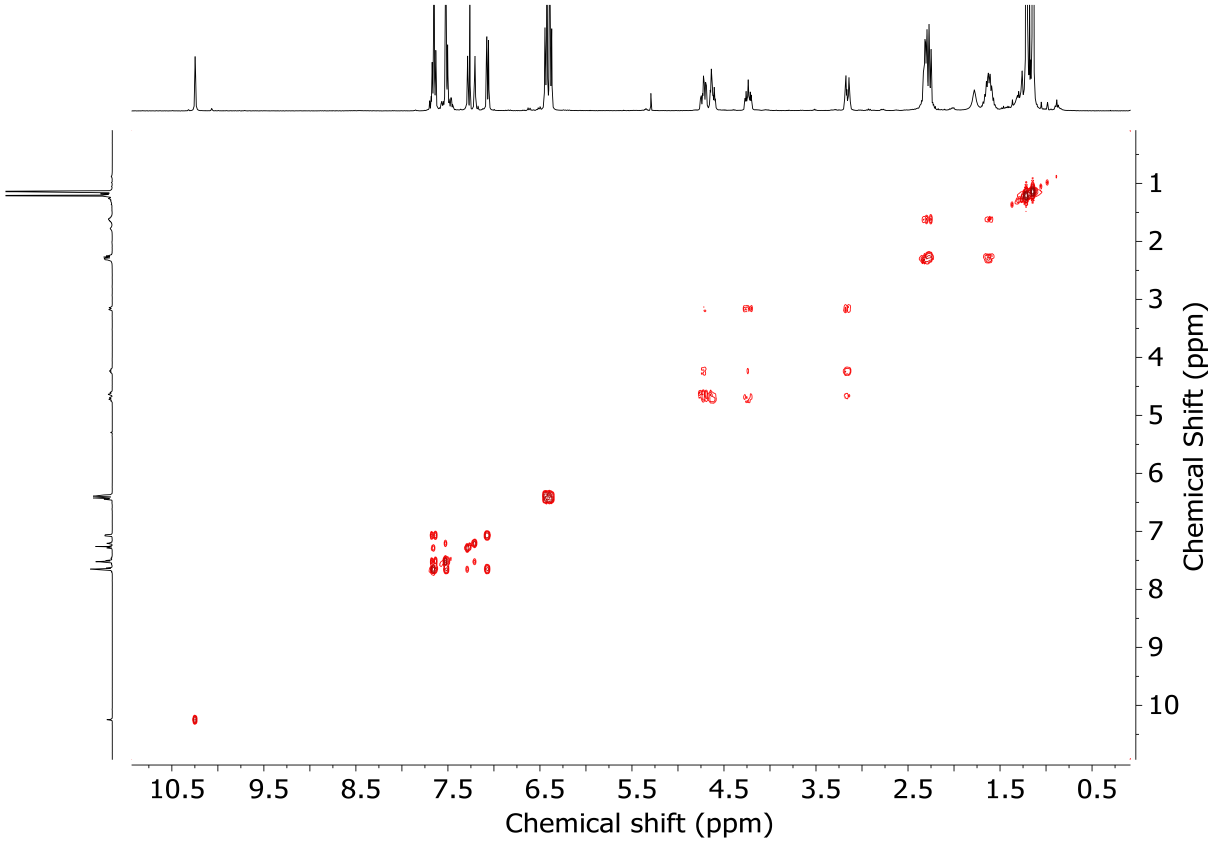

Figure S97: COSY NMR ($CDCl_3$) of (Z_m)-**6**.

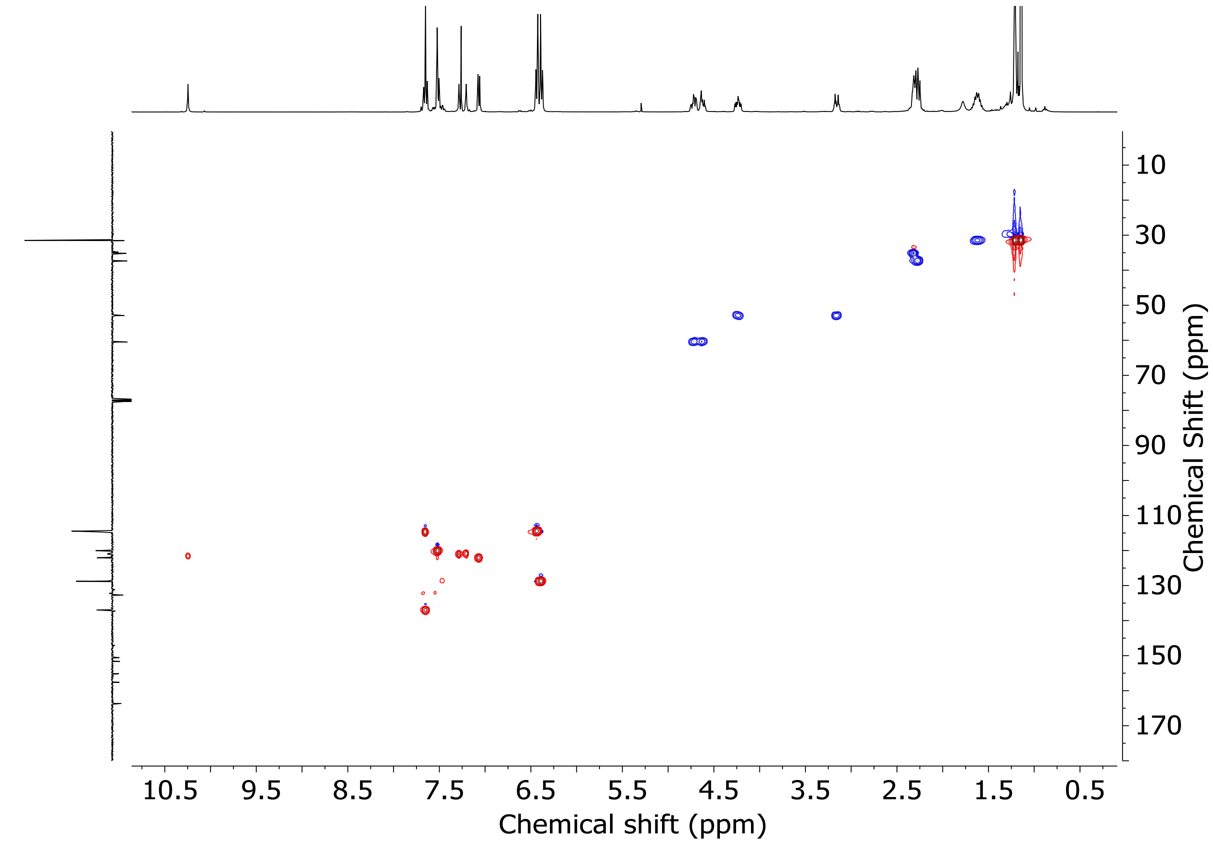

Figure S98: HSQC NMR ($CDCl_3$) of (Z_m)-**6**.

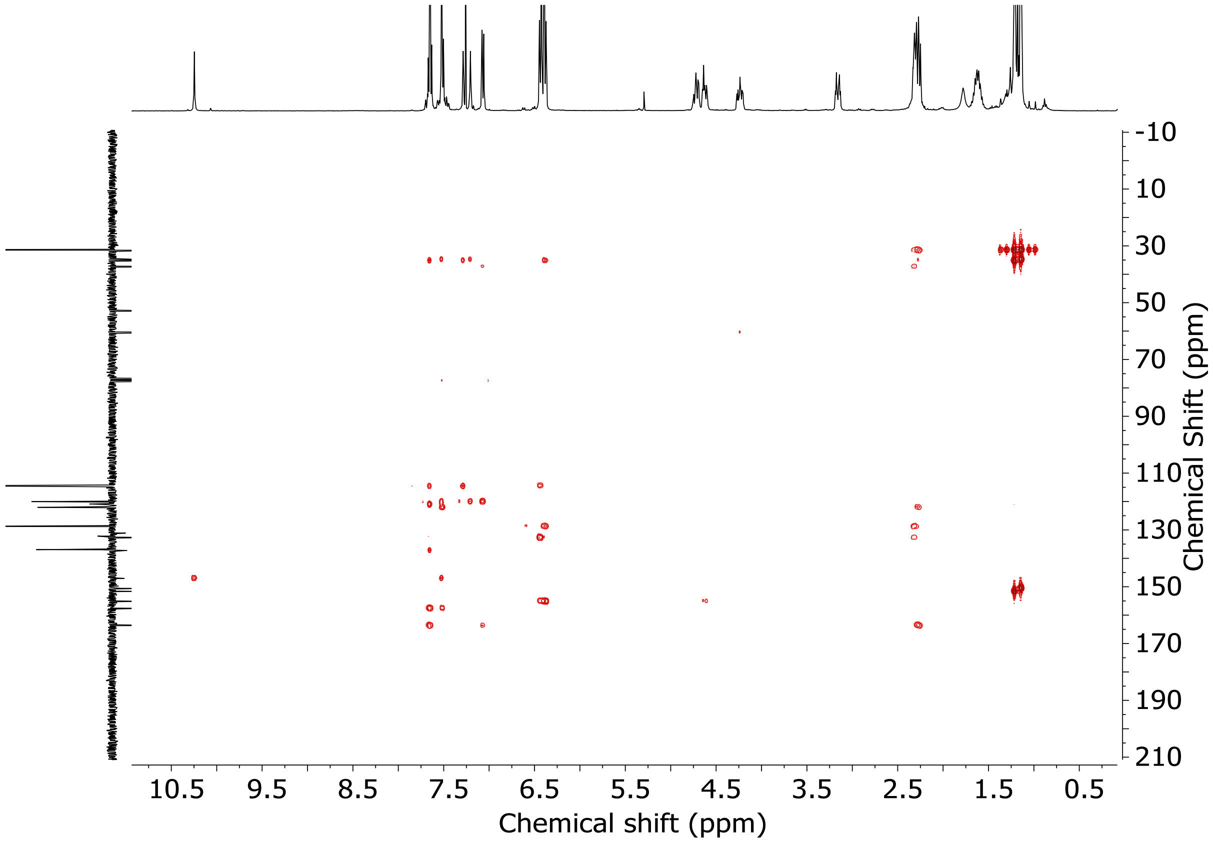

Figure S99: HMBC NMR ($CDCl_3$) of (Z_m)-**6**.

Ether alkyne S8

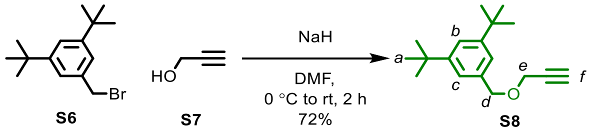

To a suspension of NaH (60% in mineral oil, 320 mg, 8.0 mmol) in DMF (15 mL) was added **S6** (250 mL, 4.0 mmol) dropwise at 0 °C. The reaction mixture was stirred for 10 minutes, then a solution of **S7** (566.4 mg, 2.0 mmol) in DMF (5 mL) was added dropwise over 10 minutes. The reaction mixture was allowed to warm to rt and stirred for 2 h. The reaction mixture was then quenched by adding H_2O (10 mL) dropwise. The aqueous and organic phases were separated, and the aqueous phase was then extracted with Et_2O (3 x 20 mL). The combined organic extracts were washed with brine (10 mL), dried ($MgSO_4$) and concentrated *in vacuo*. Chromatography (petrol-Et_2O 5→10%) gave **S8** as a colourless oil (372.1 mg, 72%).

All spectroscopic data in consitsant with the reported data.[5]

^{1}H NMR (400 MHz, $CDCl_3$) δ: 7.37 (t, *J* = 1.9, 1H, H_b), 7.19 (dt, *J* = 2.0, 0.5, 2H, H_c), 4.60 (d, *J* = 0.5, 2H, H_d), 4.19 (d, *J* = 2.4, 2H, H_e), 2.47 (t, *J* = 2.3, 1H, H_f), 1.33 (s, 18H, H_a)

^{13}C NMR (101 MHz, $CDCl_3$) δ: 150.9, 136.3, 122.5, 122.0, 80.4, 74.5, 72.4, 57.2, 34.8, 31.5.

Ether rotaxanes (E_m)-7 and (Z_m)-7

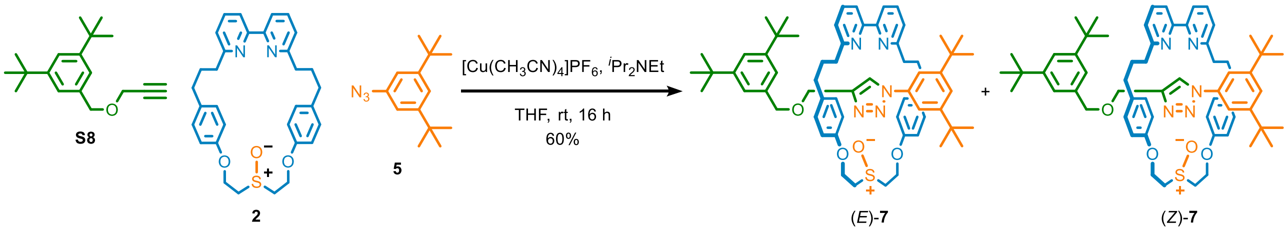

In a CEM vial were added **S8** (10.8 mg, 41.7 µmol), **5** (9.7 mg, 41.7 µmol), **2** (20.0 mg, 38.0 µmol) and $[Cu(CH_3CN)_4PF_6]$ (13.6 mg, 36.5 µmol). The vial was sealed and purged with N_2, then THF was added (1.0 mL), followed by $^iPr_2NEt$ (13.3 µL, 75.9 µmol). The solution was stirred at rt for 16 h. the solution was diluted with CH_2Cl_2 (2 mL), then EDTA-NH_3 (5 mL) was added. The solution was vigorously stirred until complete decolouration. The aqueous and organic phases were separated, and the aqueous phase was then extracted with CH_2Cl_2 (3 x 10 mL). The combined organic extracts were washed with brine (10 mL), dried ($MgSO_4$) and concentrated *in vacuo* to give a sample containing **7** as a mixture of diastereomers (49 : 51 *dr*, Figure S100). Chromatography (CH_2Cl_2-CH_3CN 0→100% then CH_3CN-MeOH 0→20%) gave **7** as an off-white foam (23.1 mg, 60%) as a mixture of diastereoisomers (1 : 0.9 *dr*, Figure S101).

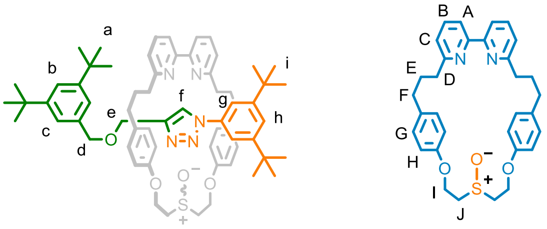

1st diastereoisomer

^{1}H NMR (400 MHz, $CDCl_3$) δ: 9.39 (s, 1H, H_f), 7.65 (t, *J* = 7.5, 2H, H_B), 7.55-7.47 (m, 4H, H_A, H_g), 7.32 (t, *J* = 1.8, 1H, H_h) 7.30 (t, *J* = 1.9, 1H, H_b), 7.08 (d, *J* = 5.4, 2H, H_C) ,7.02 (d, *J* = 1.8, 2H, H_c), 6.46-6.34 (m, 8H, H_G, H_H) 4.70-4.46 (m, 4H, H_I), 4.11 (s, 2H, H_e), 4.08 (s, 2H, Hd), 3.98 (ddd, *J* = 14.2, 9.3, 4.5, 2H, H_J) 3.01 (app dt, *J* = 13.6, 4.5, 2H, $H_{J'}$), 2.53-2.23 (m, 8H, H_D, H_F), 1.77-1.54 (m, 4H, H_E), 1.28 (s, 18H, H_i), 1.27 (s, 18H, H_a)

^{13}C NMR (101 MHz, $CDCl_3$) δ: 163.0, 157.6, 155.2, 154.9, 151.9, 150.4, 143.2, 137.5, 132.9, 128.8, 123.7, 121.9, 121.9, 120.0, 115.2, 115.0, 114.6, 114.4, 72.3, 72.2, 63.1, 63.0, 60.4, 52.8, 37.3, 35.2, 34.7, 34.6, 31.7, 29.7.

LR-ESI-MS (+ve) *m/z* = 1016.6 $[M+H]^+$, for isotopic pattern see Figure S107.

2nd diastereoisomer

^{1}H NMR (400 MHz, $CDCl_3$) δ: 9.19 (s, 1H, H_f), 7.59 (t, *J* = 7.7, 2H, H_B), 7.55-7.47 (m, 2H, H_A), 7.38 (t, *J* = 1.8, 1H, H_h) 7.28 (t, *J* = 1.9, 1H, H_b), 7.06 (d, *J* = 5.4, 2H, H_C) ,6.96 (d, *J* = 1.8, 2H, H_c), 6.56 (app. s, 8H, H_G, H_H) 4.70-4.46 (m, 4H, H_I), 3.91 (s, 2H, H_e), 3.83 (s, 2H, H_d), 3.76 (ddd, *J* = 14.2, 9.3, 4.5, 2H, H_J) 3.15 (app dt, *J* = 13.6, 4.5, 2H, $H_{J'}$), 2.53-2.23 (m, 8H, H_D, H_F), 1.77-1.54 (m, 4H, H_E), 1.27 (s, 18H, H_i), 1.23 (s, 18H, H_a)

^{13}C NMR (101 MHz, $CDCl_3$) δ: 162.7, 157.6, 155.2, 151.6, 150.5, 143.9, 136.7, 133.6, 128.5, 123.7, 121.9, 121.8, 121.7, 121.33, 120.0, 115.2, 115.0, 114.6, 114.4, 72.3, 72.2, 63.1, 63.0, 60.7, 51.8, 37.2, 35.2, 34.8, 31.4, 29.7.

LR-ESI-MS (+ve) *m/z* = 1016.6 $[M+H]^+$, for isotopic pattern see Figure S107.

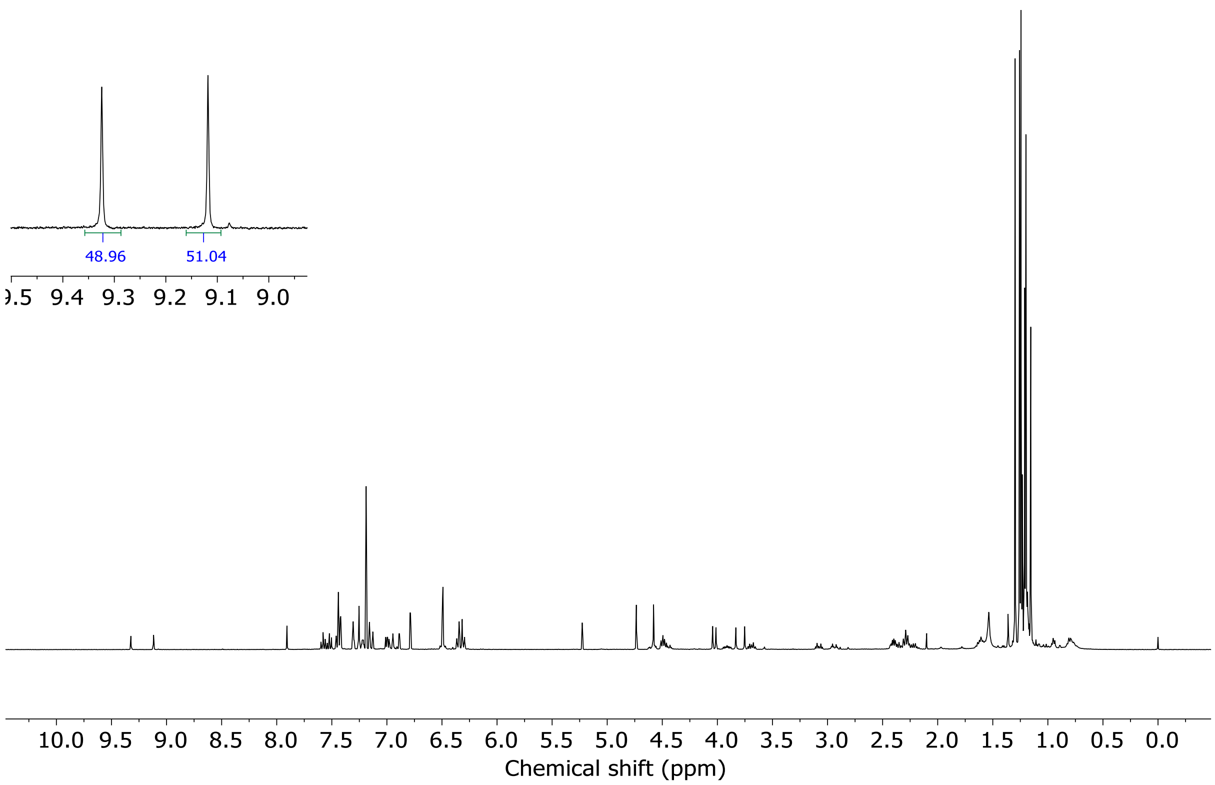

Figure S100: ^{1}H NMR ($CDCl_3$, 400 MHz) of of (E_m)-**7** and (Z_m)-**7** prior to chromatography (49 : 51 *dr*).

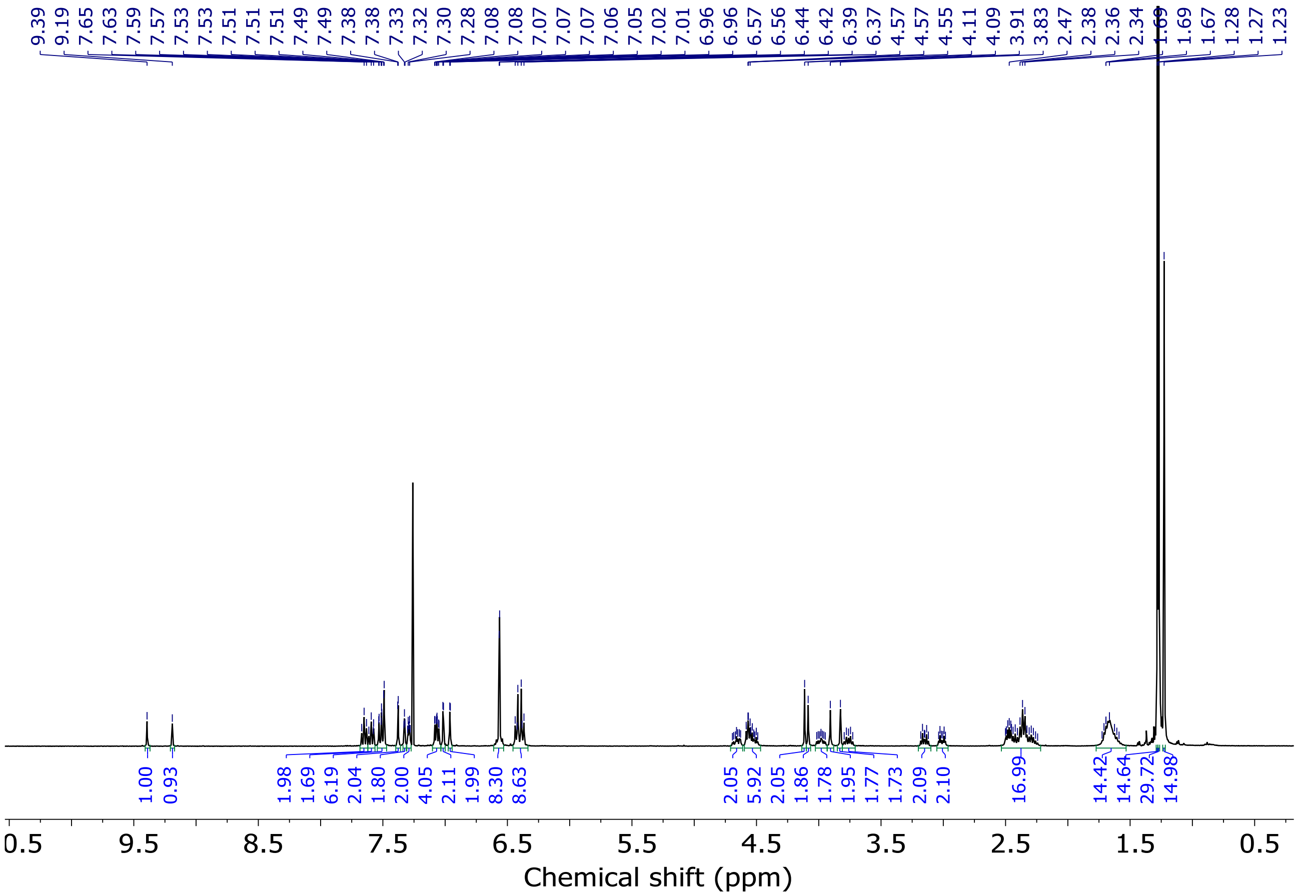

Figure S101: ^{1}H NMR ($CDCl_3$, 400 MHz) of (E_m)-**7** and (Z_m)-**7** (1 : 0.9 *dr*).

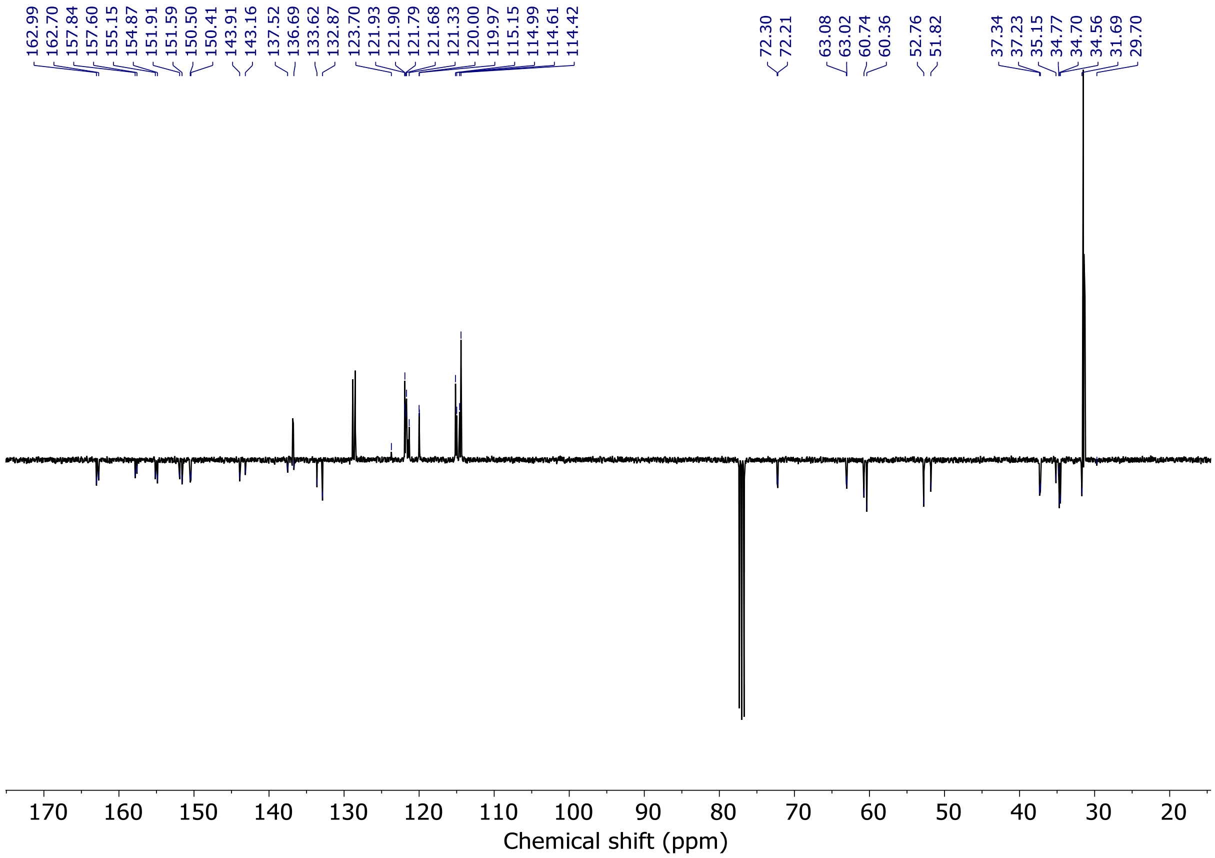

Figure S102: JMOD NMR ($CDCl_3$, 101 MHz) of (E_m)-**7** and (Z_m)-**7** (1 : 0.9 *dr*).

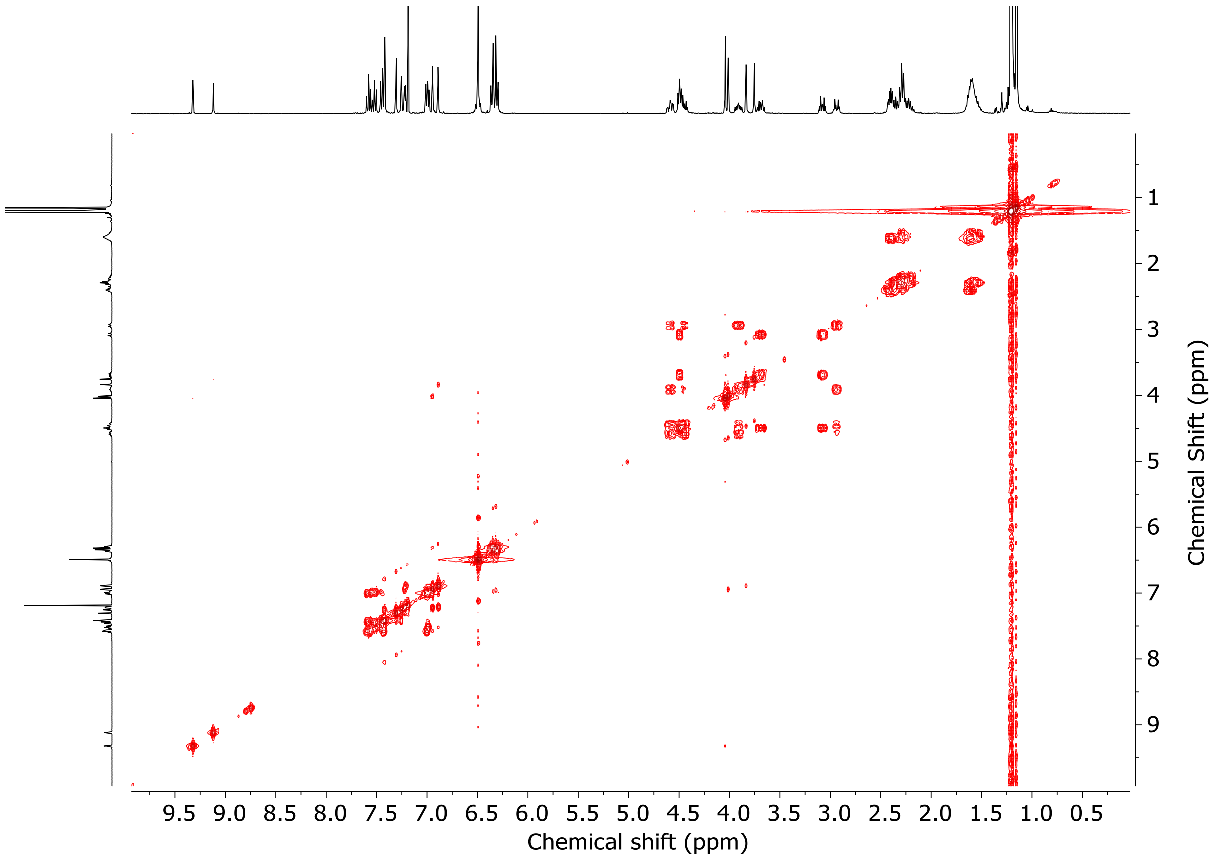

Figure S103: COSY NMR ($CDCl_3$) of (E_m)-**7** and (Z_m)-**7** (1 : 0.9 *dr*).

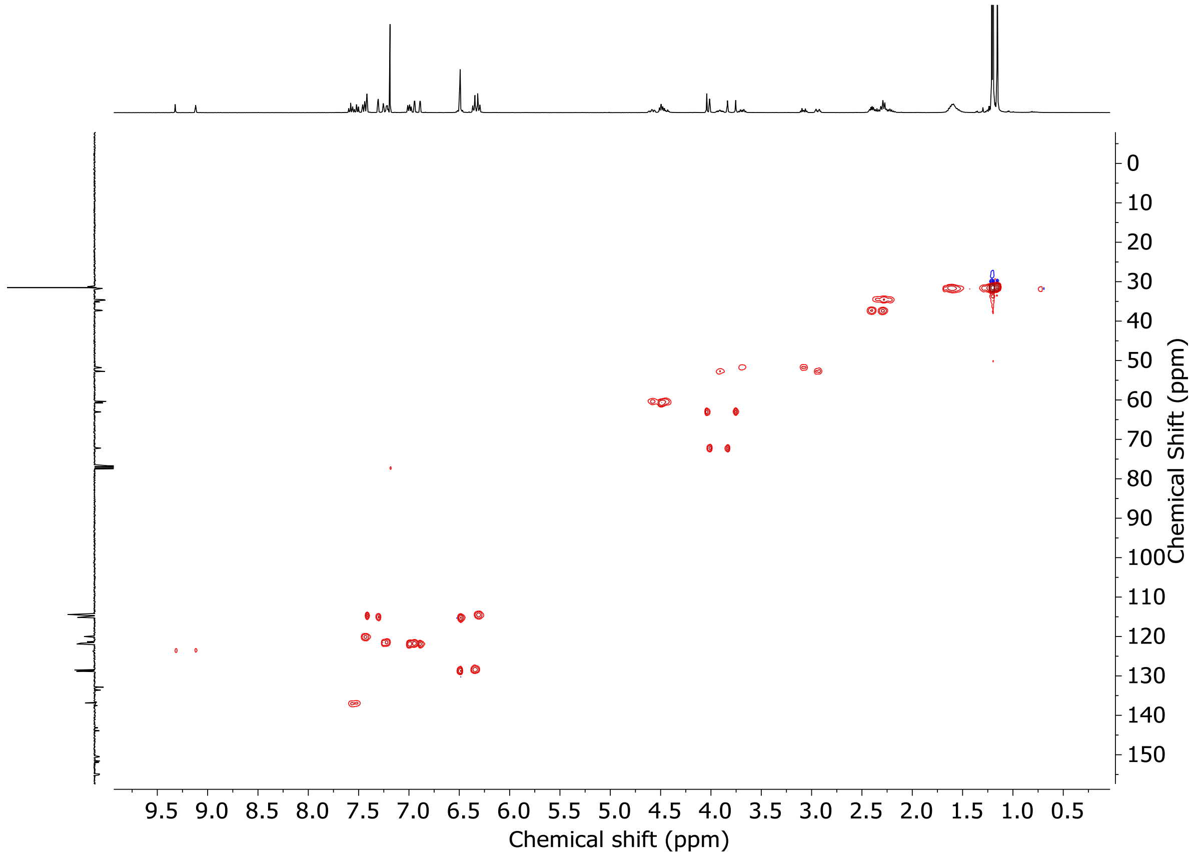

Figure S104: HSQC NMR ($CDCl_3$) of (*E*$_m$)-**7** and (*Z*$_m$)-**7** (1 : 0.9 *dr*).

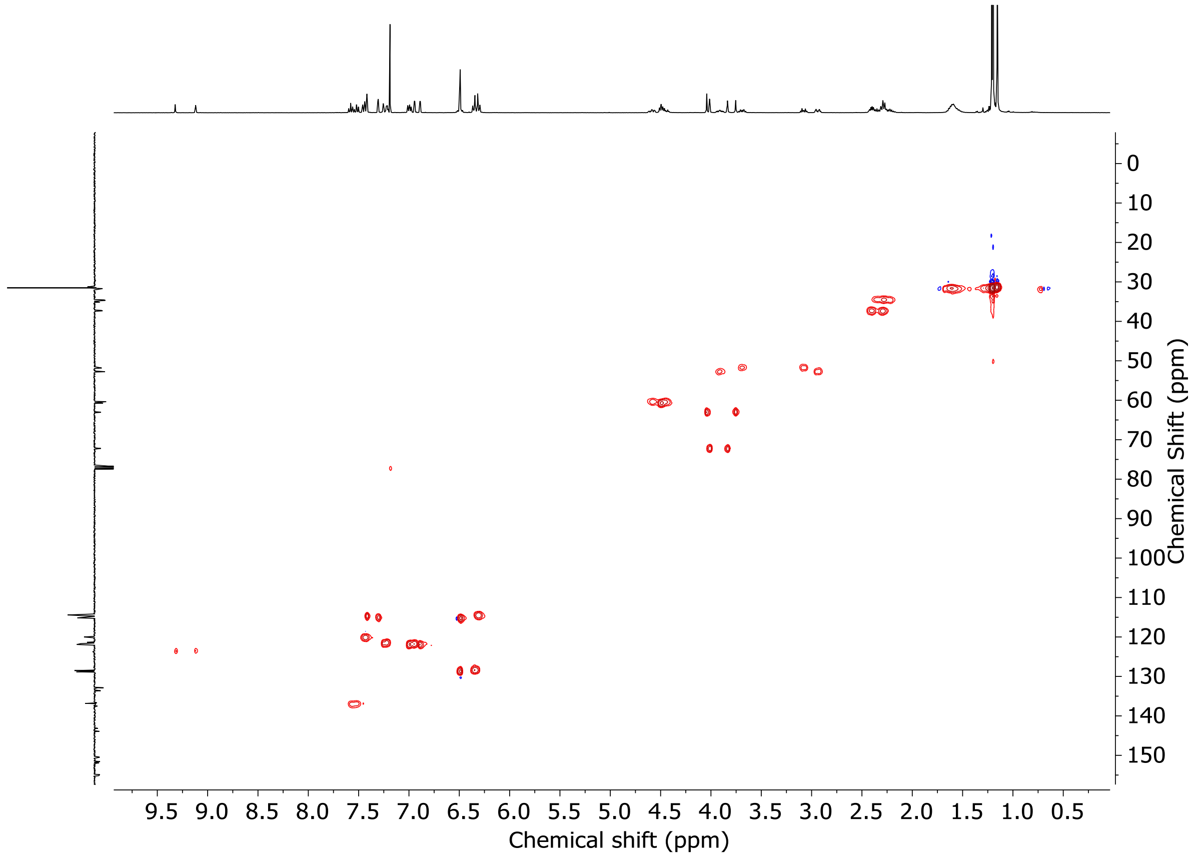

Figure S105: HMBC NMR ($CDCl_3$) of (*E*$_m$)-**7** and (*Z*$_m$)-**7** (1 : 0.9 *dr*).

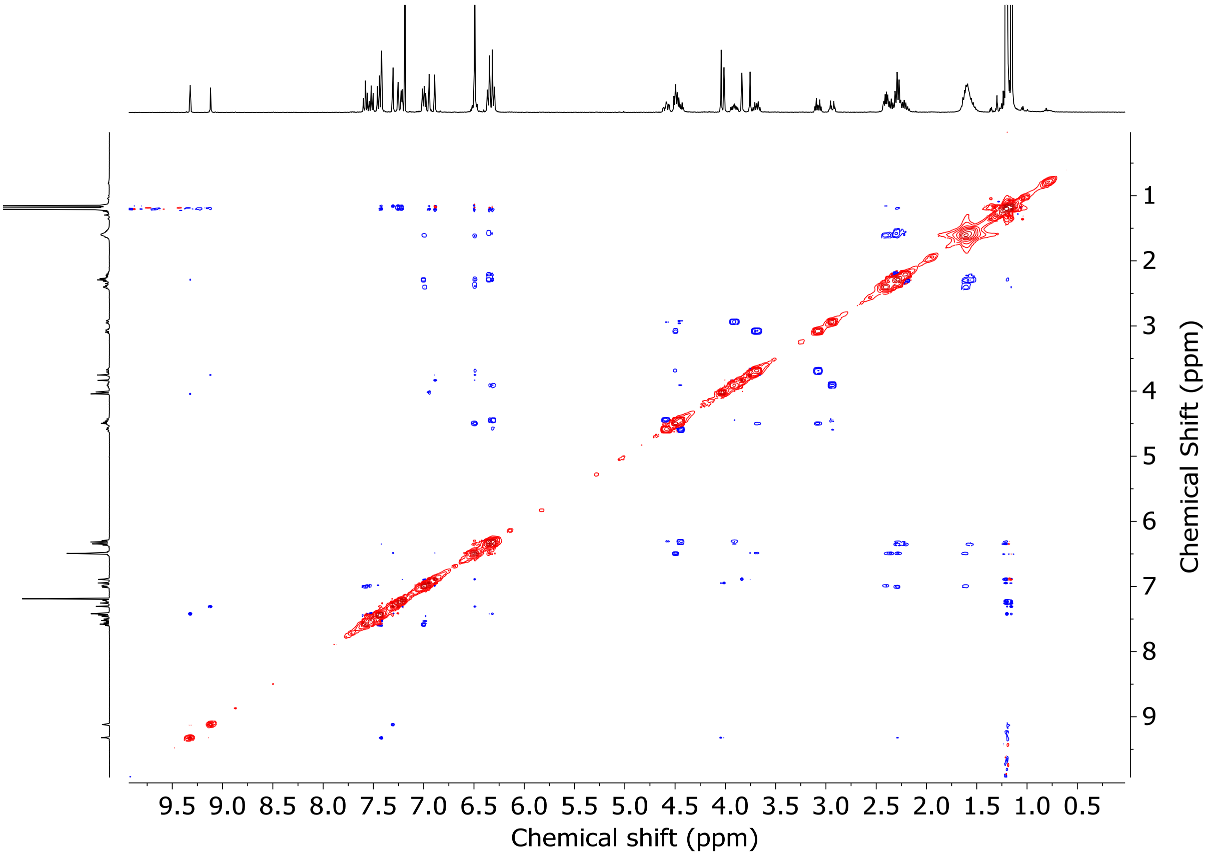

Figure S106: NOESY NMR ($CDCl_3$) of (*E*m)-**7** and (*Z*m)-**7** (1 : 0.9 *dr*).

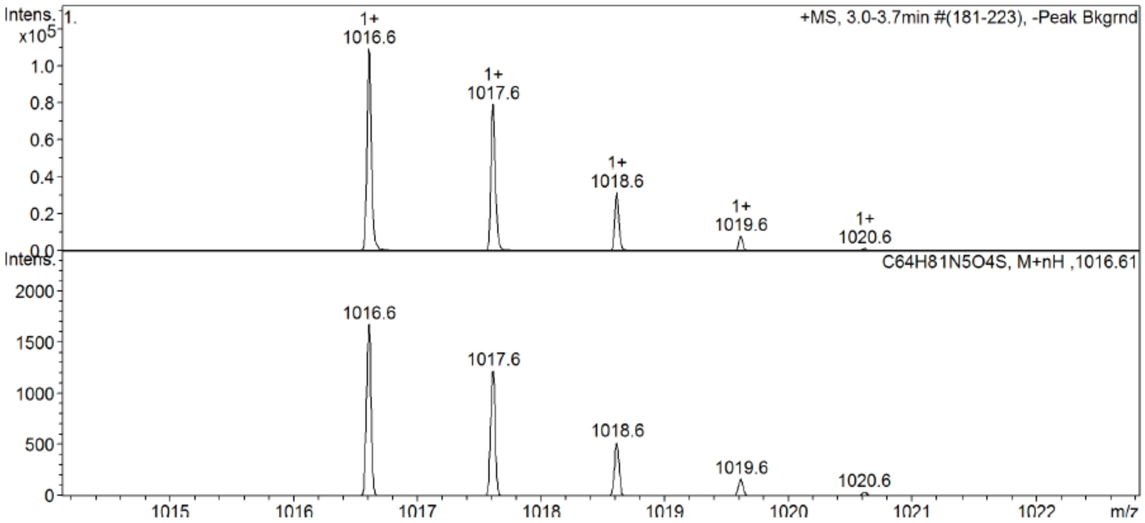

Figure S107: Calculated (top) and observed (bottom) isotopic patterns for (*E*m)-**7** and (*Z*m)-**7**.

Alkyl bromide S10

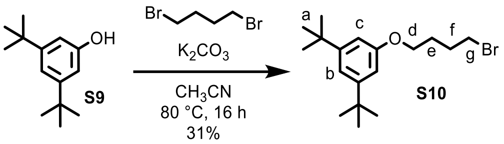

A suspension of **S9** (206 mg, 1.0 mmol), K_2CO_3 (552.8 mg, 4.0 mmol) and 1,4-dibromobutane (459 µL, 4.0 mmol) was stirred at 85 °C for 16 h. H_2O (10 mL) was added, then the aqueous and organic phases were separated, and the aqueous phase was then extracted with CH_2Cl_2 (3 x 20 mL). The combined organic extracts were washed with brine (10 mL), dried ($MgSO_4$) and concentrated *in vacuo*. Chromatography (petrol-EtOAc 0→10%) gave **S10** as a colourless liquid (106 mg, 31%).

^{1}H NMR (400 MHz, $CDCl_3$) δ: 7.02 (t, *J* = 1.6, 1H, H_b), 6.76 (d, *J* = 1.7, 2H, H_c), 4.01 (t, *J* = 6.1, 2H, H_d), 3.51 (t, *J* = 6.7, 2H, H_g), 2.09 (tt, *J* = 9.8, 6.5, 2H, H_f), 1.95 (tt, *J* = 5.9, 8.6, 2H, H_e), 1.32 (s, 18H, H_a)

^{13}C NMR (101 MHz, $CDCl_3$) δ: 158.4, 152.2, 115.1, 108.8, 66.6, 35.0, 33.6, 31.5, 29.6, 28.1.

HR-EI-MS (+ve) *m/z* = 340.1398 $[M+H]^+$ (calc. 340.1402 *m/z* for $C_{18}H_{29}BrO$).

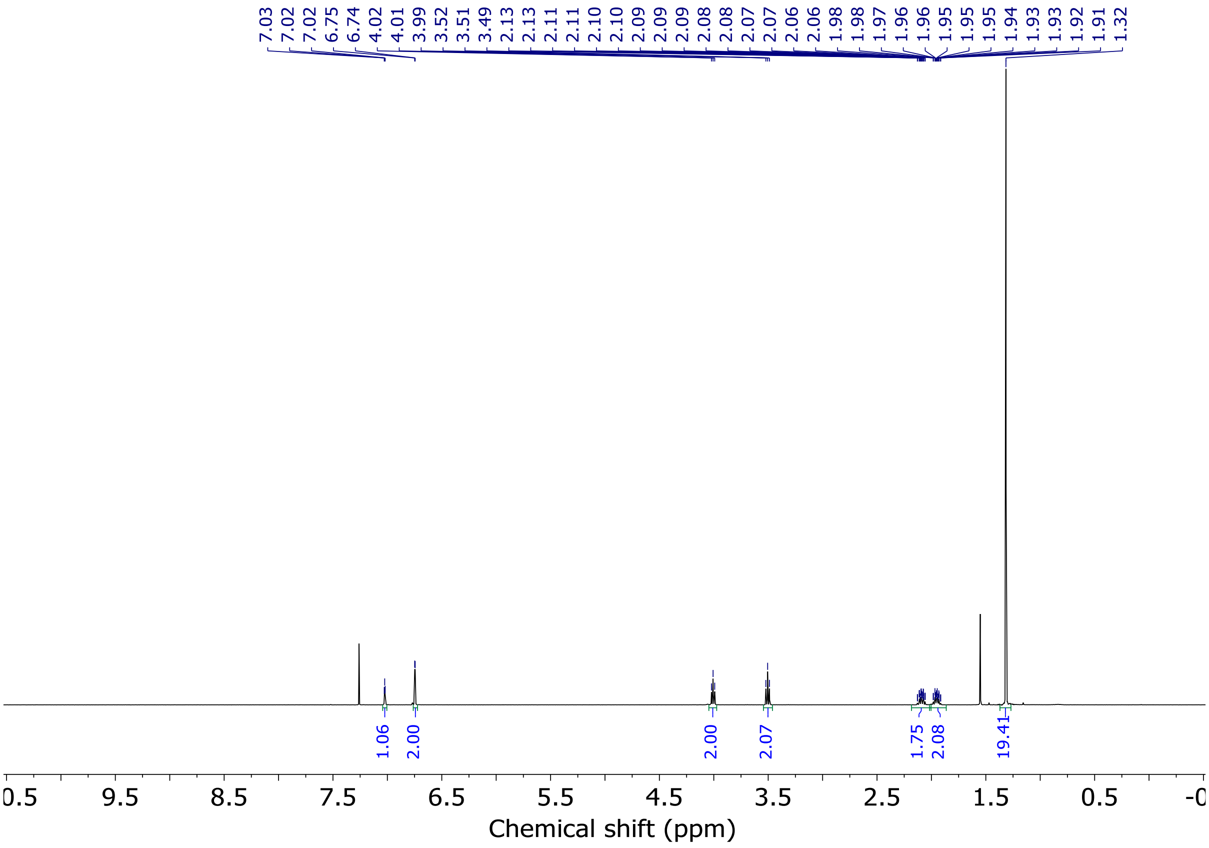

Figure S108: ^{1}H NMR ($CDCl_3$, 400 MHz) of **S10**.

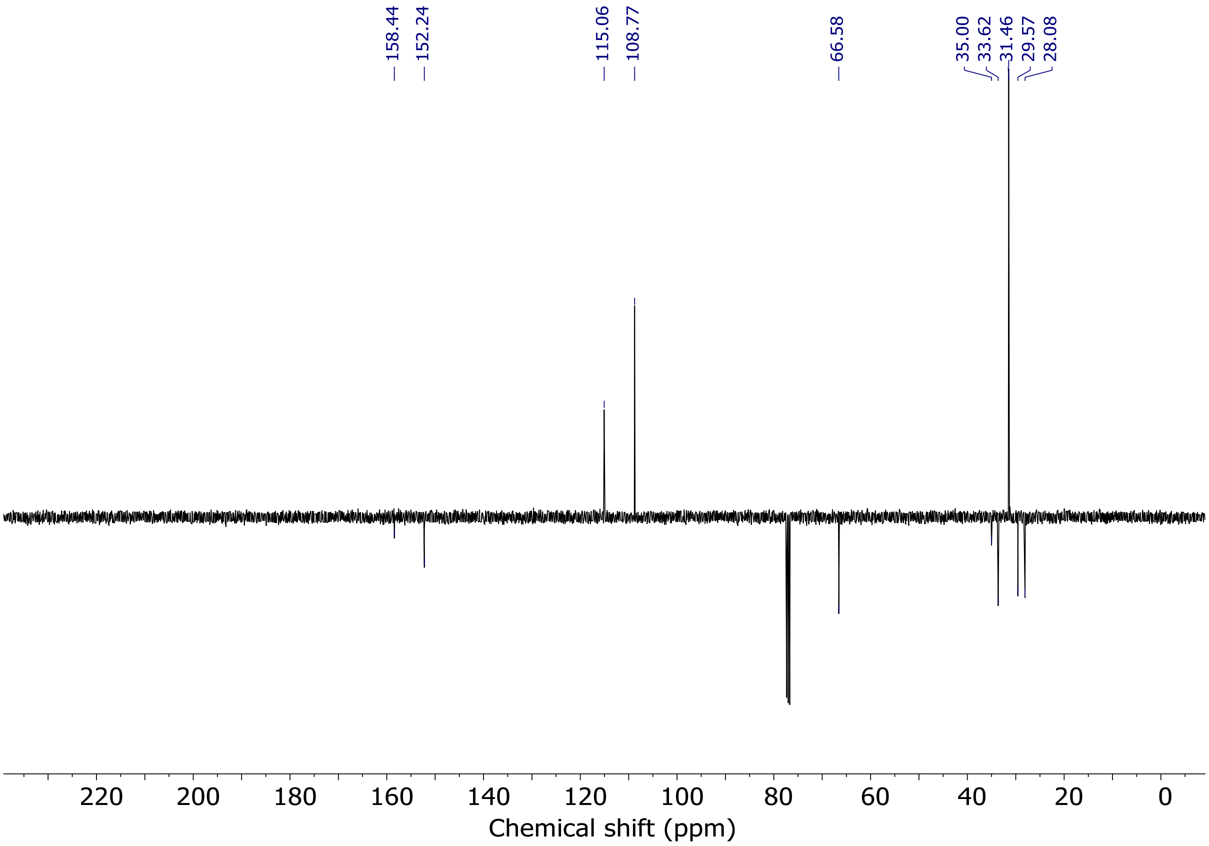

Figure S109: JMOD NMR ($CDCl_3$, 101 MHz) of **S10**.

Azide S11

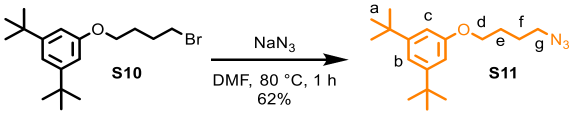

A suspension of **S10** (108.1 mg, 0.31 mmol) and NaN_3 (80.6 mg, 1.24 mmol) in DMF (2 mL) was stirred ad 80 °C for 1 h. 5% $LiCl_{(aq)}$ (5 mL) was added, then the aqueous and organic phases were separated, and the aqueous phase was then extracted with EtOAc (3 x 10 mL). The combined organic extracts were washed with brine (10 mL), dried ($MgSO_4$) and concentrated *in vacuo*. Chromatography (petrol-EtOAc 90 : 10) gave **S11** as a colourless liquid (58.0 mg, 62%).

^{1}H NMR (400 MHz, $CDCl_3$) δ: 7.05 (t, *J* = 1.6, 1H, H_b), 6.77 (d, *J* = 1.7, 2H, H_c), 4.02 (t, *J* = 6.0, 2H, H_d), 3.39 (t, *J* = 6.5, 2H, H_g), 1.95-1.77 (m, 4H, H_e, H_f), 1.33 (s, 18H, H_a).

^{13}C NMR (101 MHz, $CDCl_3$) δ: 158.5, 152.2, 115.1, 108.8, 66.9, 51.3, 35.0, 31.5, 26.7, 25.9.

HR-EI-MS (+ve) *m/z* = 303.2306 $[M+H]^+$ (calc. 303.2311 *m/z* for $C_{18}H_{29}N_3O$).

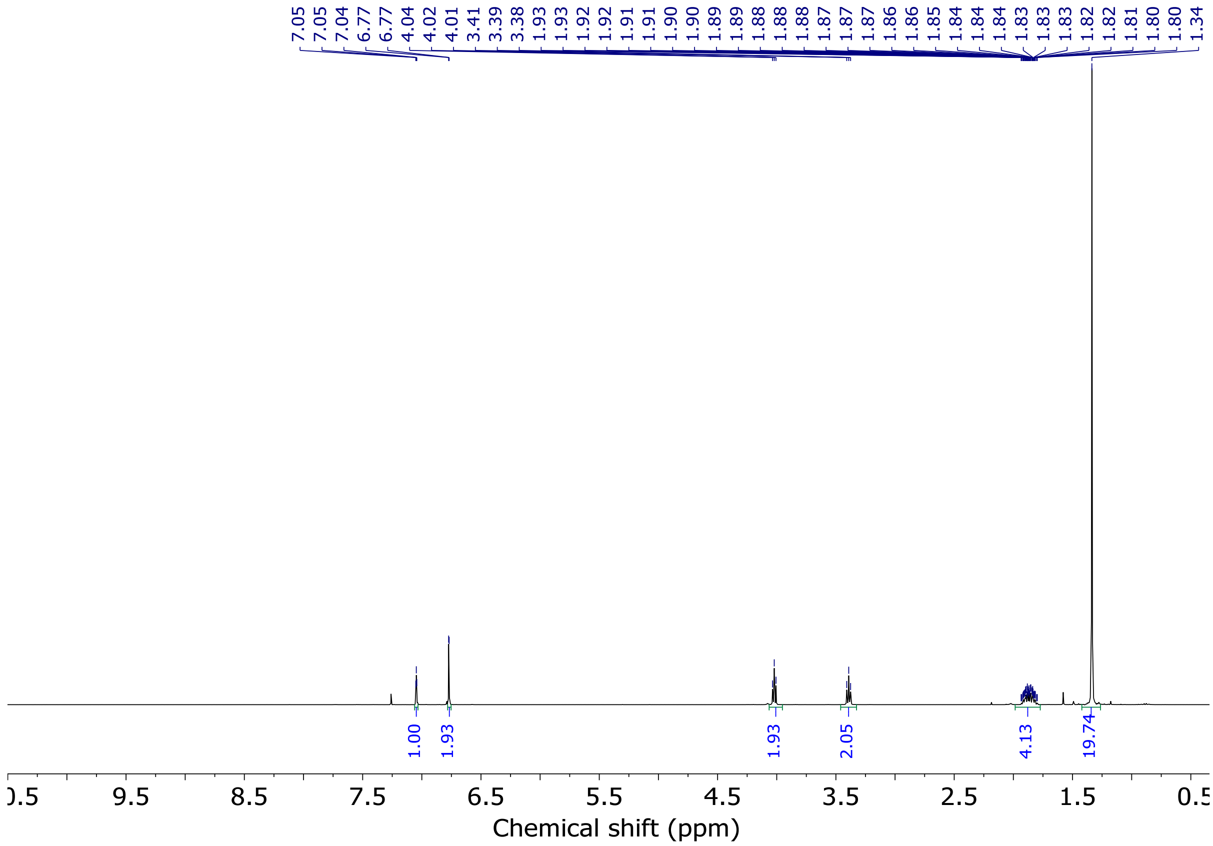

Figure S110: ^{1}H NMR ($CDCl_3$, 400 MHz) of **S11**.

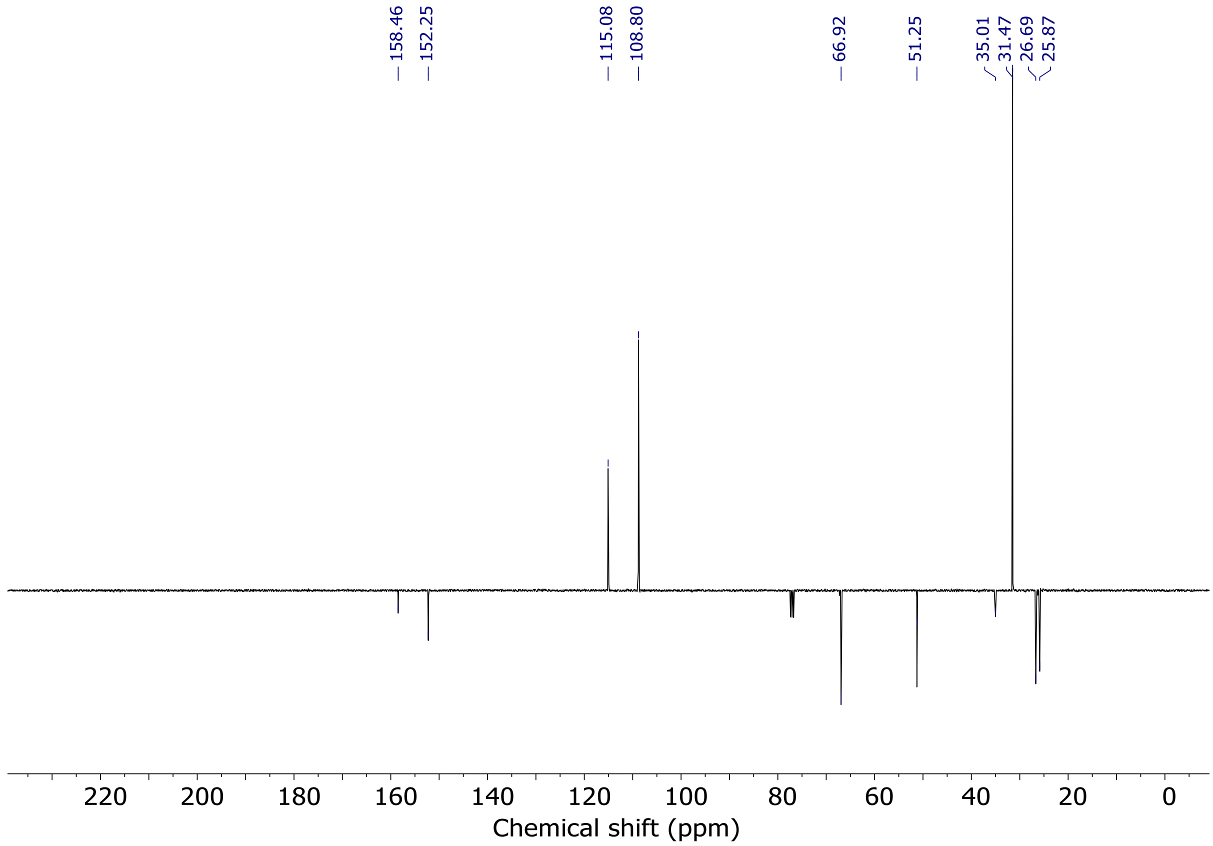

Figure S111: JMOD NMR ($CDCl_3$, 101 MHz) of **S11**.

Phenolic Rotaxanes (E_m)-8 and (Z_m)-8

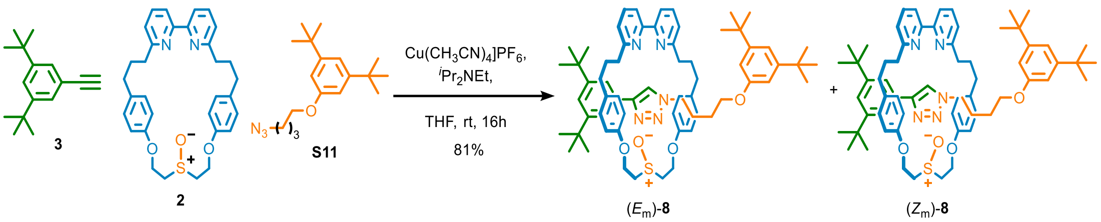

In a CEM vial were added **3** (2.1 mg, 10.3 µmol), **S11** (3.1 mg, 10.3 µmol), **2** (5.0 mg, 9.5 µmol) and [$Cu(CH_3CN)_4PF_6$] (3.4 mg, 9.1 µmol). The vial was sealed and purged with N_2, then THF was added (1.0 mL), followed by $^iPr_2NEt$ (3.3 µL, 19.0 µmol). The solution was stirred at rt for 16 h. the solution was diluted with CH_2Cl_2 (2 mL), then EDTA-NH_3 (5 mL) was added. The solution was vigorously stirred until complete decolouration. The aqueous and organic phases were separated, and the aqueous phase was then extracted with CH_2Cl_2 (3 x 10 mL). The combined organic extracts were washed with brine (10 mL), dried ($MgSO_4$) and concentrated *in vacuo* to give a sample containing **8** as a mixture of diastereomers (61 : 39 *dr*, Figure S112). Chromatography (CH_2Cl_2-CH_3CN 0→100%) gave **8** as a colourless oil (8.1 mg, 82%) as a mixture of diastereoisomers (1.4 : 1 *dr*, Figure S113).

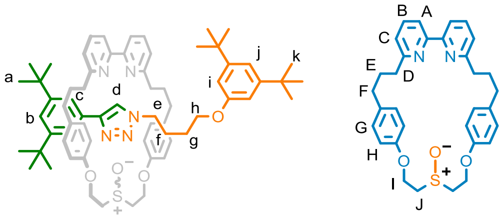

1st diastereoisomer

^{1}H NMR (500 MHz, $CDCl_3$) δ: 8.60 (s, 1H, H_d), 7.63-7.54 (m, 4H, H_B, H_C), 7.51 (d, *J* = 7.6, 2H, H_A), 7.31(t, *J* = 1.8, 1H, H_b), 7.10 (d, *J* = 7.7, 2H, H_C), 7.04-7.00 (m, 1H, H_j), 6.70 (d, *J* = 8.0, 4H, H_G), 6.67 (d, *J* = 1.6, 2H, H_i), 6.62 (d, *J* = 8.0, 4H, H_H), 4.65-4.58 (m, 2H, H_J), 4.57-4.48 (m, 2H, $H_{J'}$), 3.79 (ddd, *J* = 14.3, 9.8, 4.5, 2H, H_I), 3.56-3.48 (m, 2H, H_h), 3.10-2.98 (m, 4H, $H_{I'}$, H_e), 2.61-2.31 (m, 8H, H_D, H_F), 1.86-1.62 (m, 4H, H_E), 1.34 (s, 18H, H_k), 1.26 (s, 18H, H_a), 1.21-0.75 (m, 4H, H_f, H_g).

^{13}C NMR (126 MHz, $CDCl_3$) δ: 162.6, 158.5, 157.8, 155.5, 152.1, 150.9, 147.5, 136.9, 133.9, 139.6, 129.3, 122.1, 121.9, 121.5, 120.2, 119.8, 114.7, 114.7, 108.7, 66.5, 60.3, 53.1, 48.6, 37.4, 35.0, 34.5, 31.9, 31.5, 31.4, 26.2, 26.0

LR-ESI-MS (+ve) *m/z* = 1044.6 $[M+H]^+$, for isotopic pattern see Figure S119.

2nd diastereoisomer

^{1}H NMR (500 MHz, $CDCl_3$) δ: 8.25 (s, 1H, H_d), 7.65 (t, *J* = 7.6, 2H, H_B), 7.63-7.54 (m, 4H, H_A, H_c), 7.33 (t, *J* = 1.4, H_b), 7.12 (d, *J* = 7.7, 2H, H_C), 7.04-7.00 (m, 1H, H_j), 6.65 (d, *J* = 1.6, 2H, H_i), 6.53 (app. s, 8H, H_G, H_H), 4.65-4.58 (m, 2H, H_J), 4.57-4.48 (m, 2H, $H_{J'}$), 3.72 (dt, *J* = 13.6, 7.0, 2H, H_I), 3.56-3.48 (m, 2H, H_e), 3.40 (t, *J* = 7.5, 2H, H_h), 3.10-2.98 (m, 2H, $H_{I'}$), 2.64 (t, *J* = 8.5, 4H, H_D), 2.61-2.31 (m, 4H, H_F), 1.35 (s, 18H, H_a), 1.86-1.62 (m, 4H, H_E), 1.33 (s, 18H, H_k) 1.21-0.75 (m, 4H, H_f, H_g)

^{13}C NMR (126 MHz, $CDCl_3$) δ: 162.6, 158.4, 157.9, 155.0, 152.2, 150.7, 147.2, 136.9, 133.9, 130.7, 128.7, 122.0, 121.2, 121.2, 120.2, 120.1, 115.2, 114.9, 108.6, 66.5, 61.0, 51.8, 48.8, 37.6, 35.0, 34.3, 32.0, 31.6, 31.5, 26.2, 26.1

HR-ESI-MS (+ve) m/z = 1044.6 $[M+H]^+$, for isotopic pattern see Figure S119.

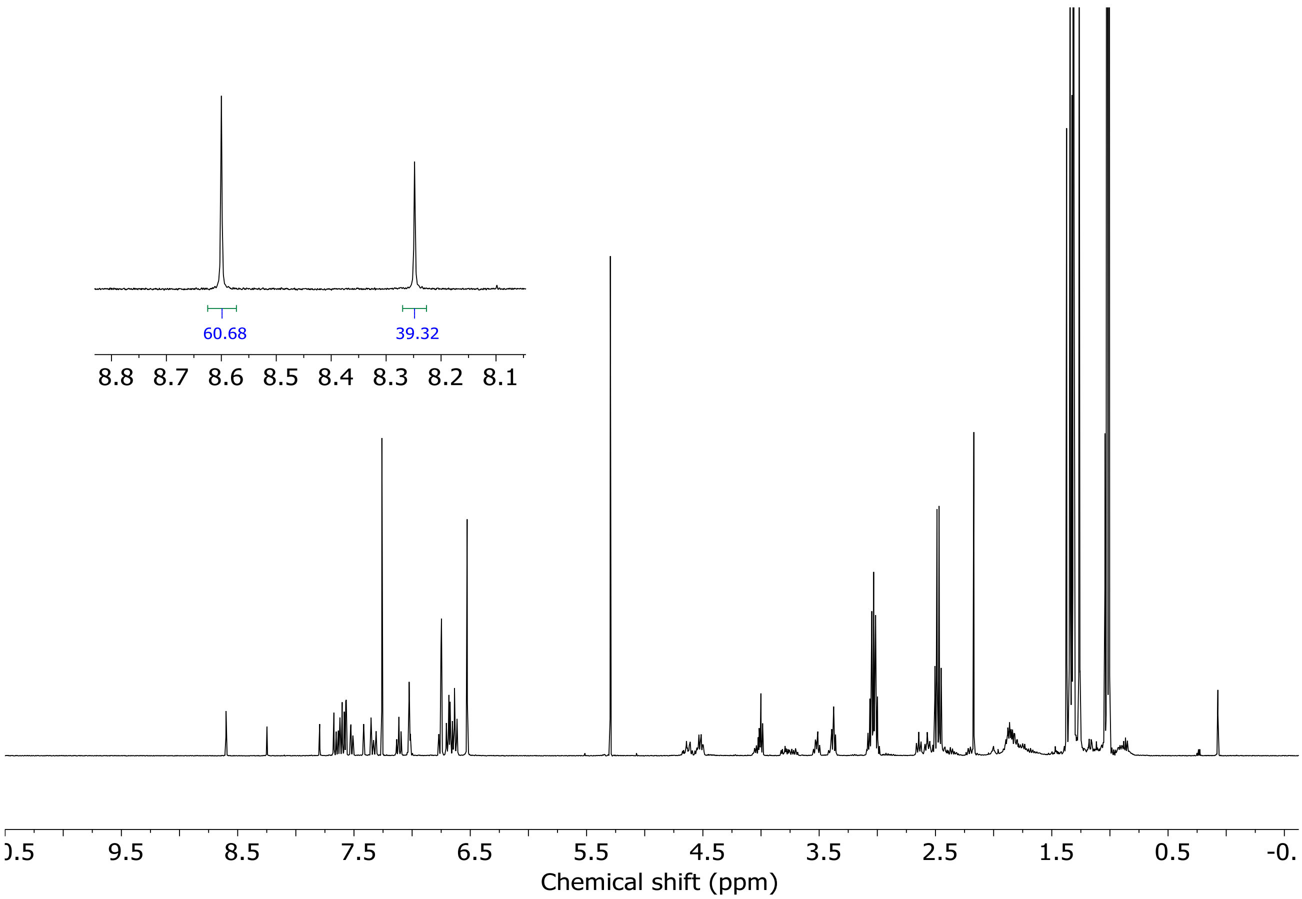

Figure S112: ^{1}H NMR ($CDCl_3$, 400 MHz) of of (E_m)-**8** and (Z_m)-**8** prior to chromatography (61 : 39 *dr*).

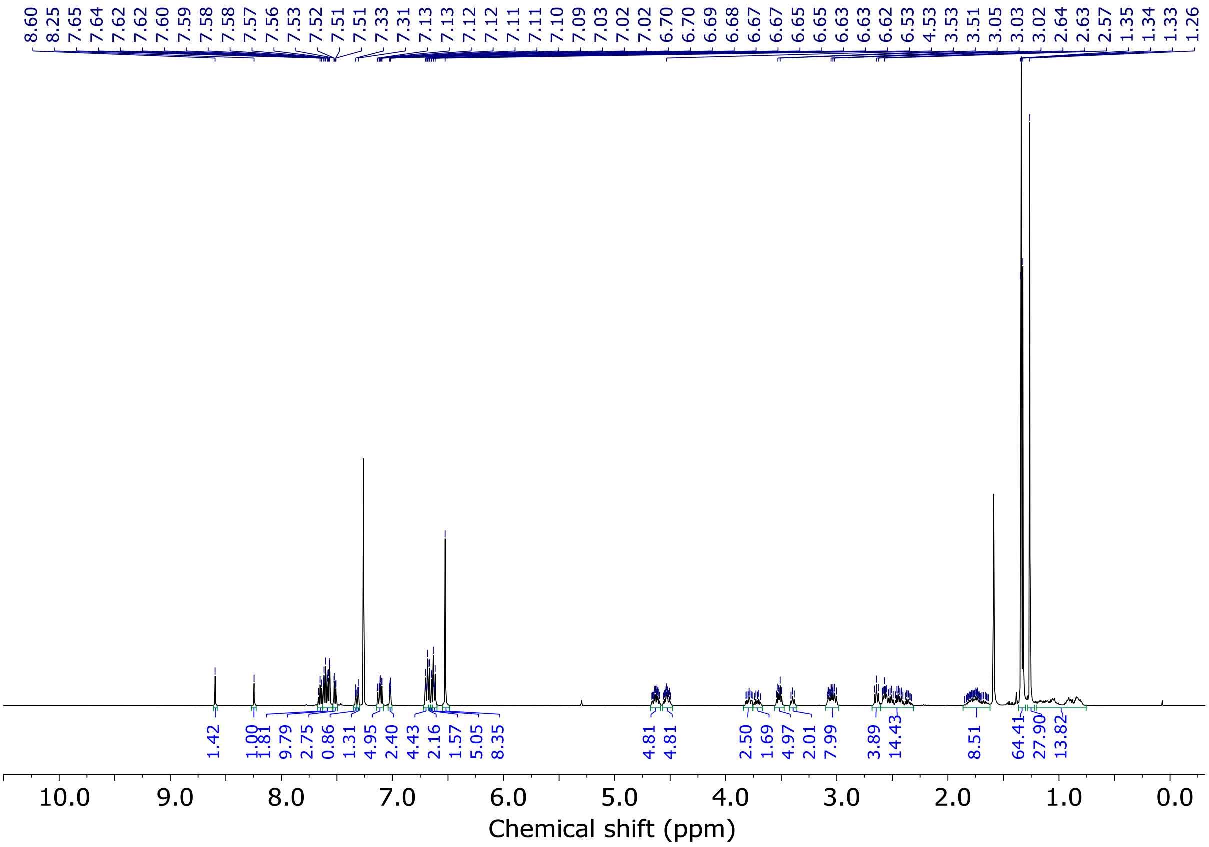

Figure S113: ^{1}H NMR ($CDCl_3$, 500 MHz) of (E_m)-**8** and (Z_m)-**8** (1.4 : 1 *dr*).

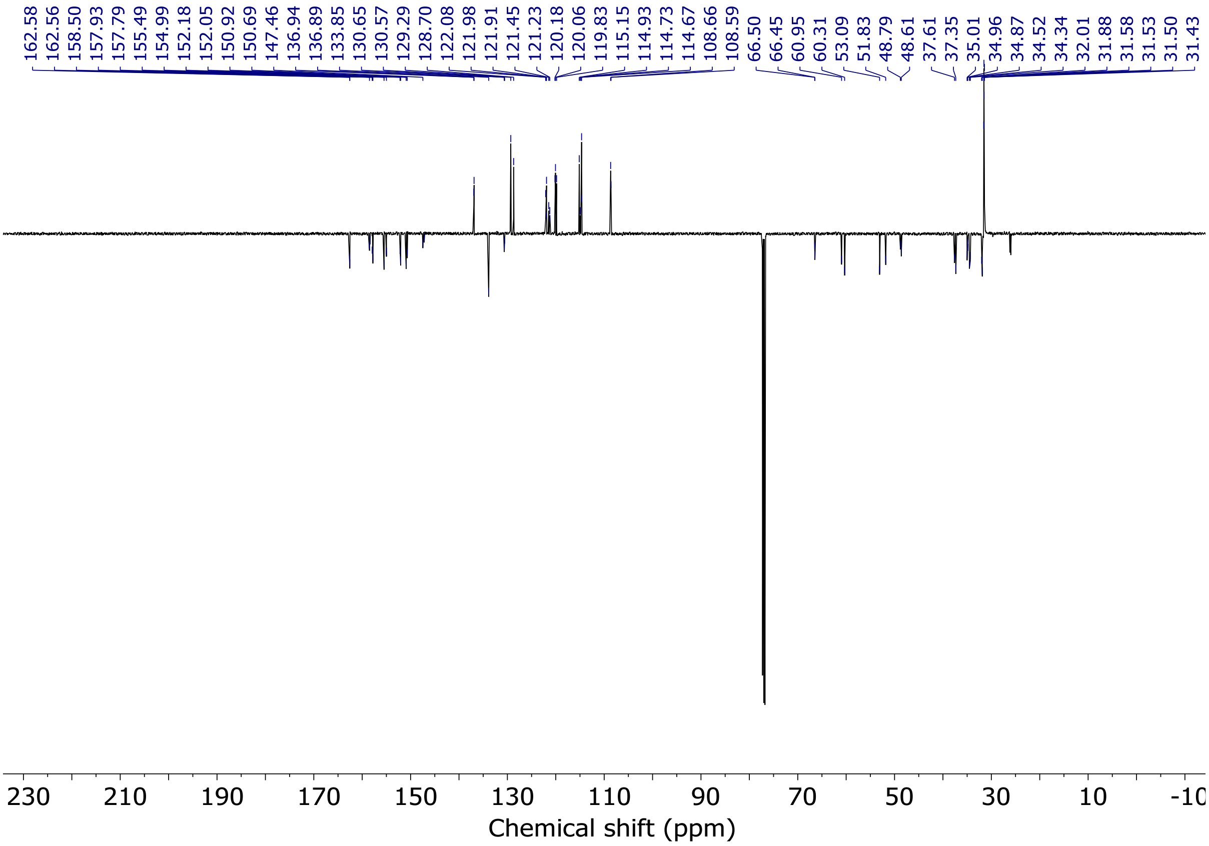

Figure S114: JMOD NMR ($CDCl_3$, 126 MHz) of (E_m)-**8** and (Z_m)-**8** (1.4 : 1 *dr*).

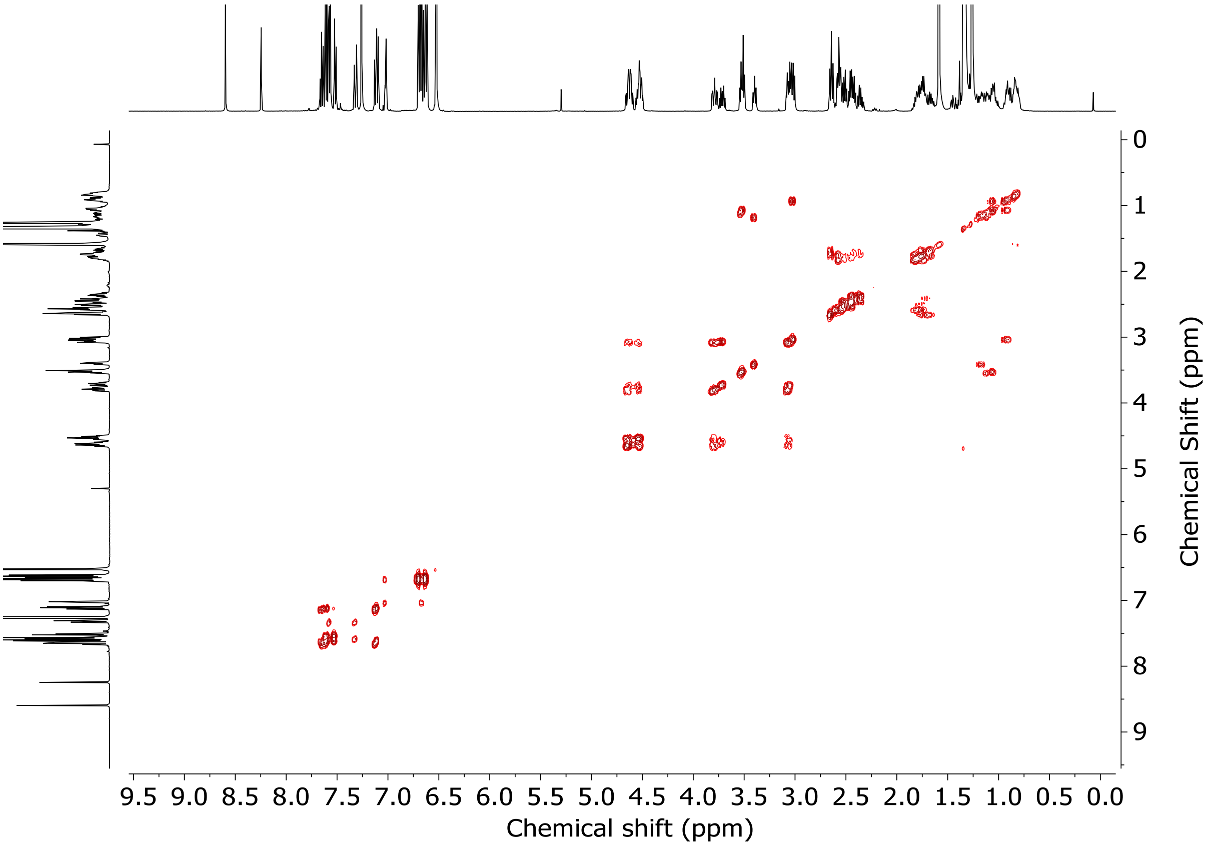

Figure S115: COSY NMR ($CDCl_3$) of (E_m)-**8** and (Z_m)-**8** (1.4 : 1 *dr*).

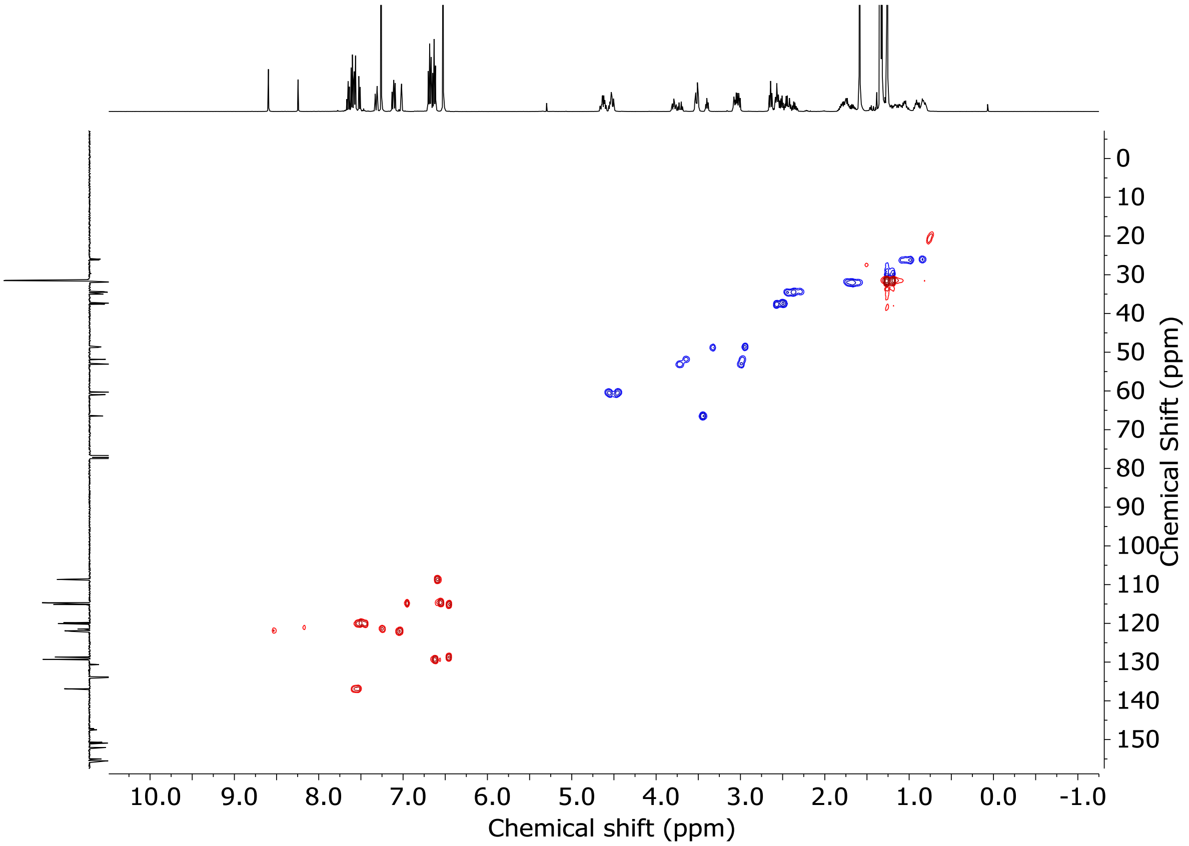

Figure S116: HSQC NMR ($CDCl_3$) of (E_m)-**8** and (Z_m)-**8** (1.4 : 1 *dr*).

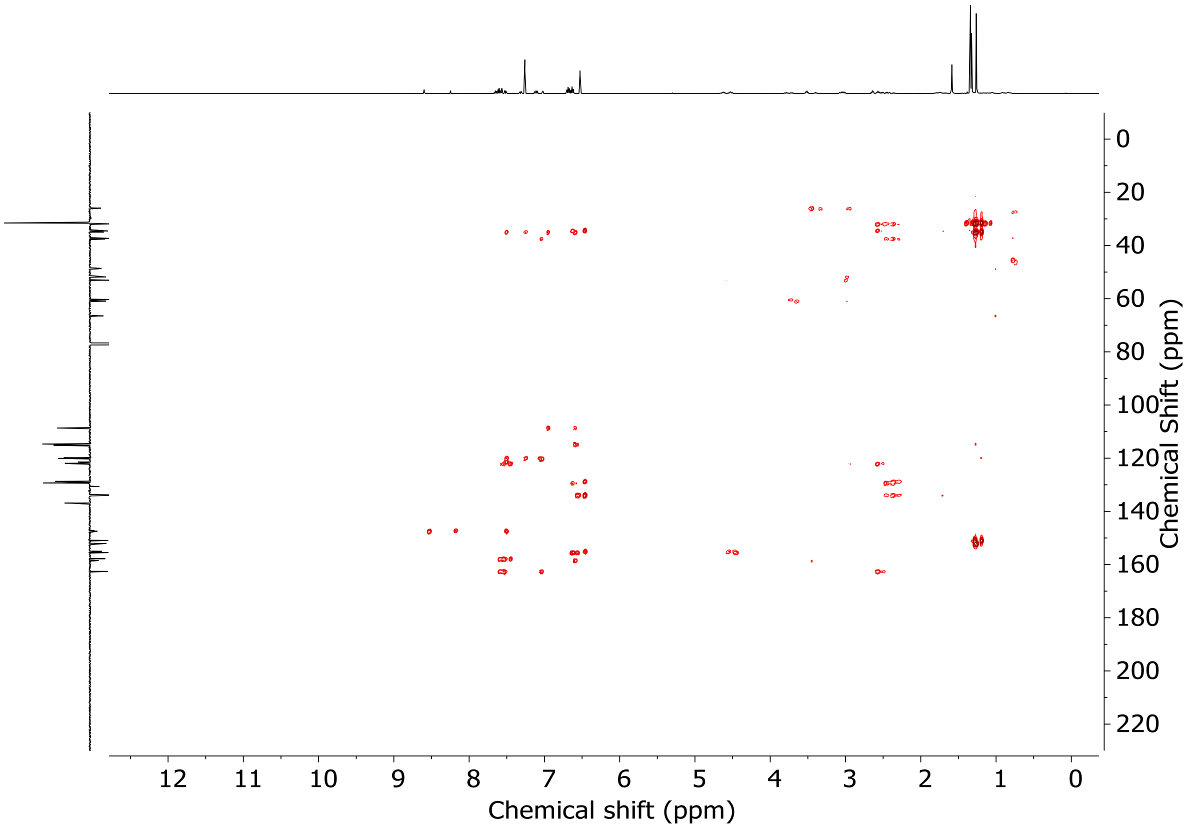

Figure S117: HMBC NMR ($CDCl_3$) of (E_m)-**8** and (Z_m)-**8** (1.4 : 1 *dr*).

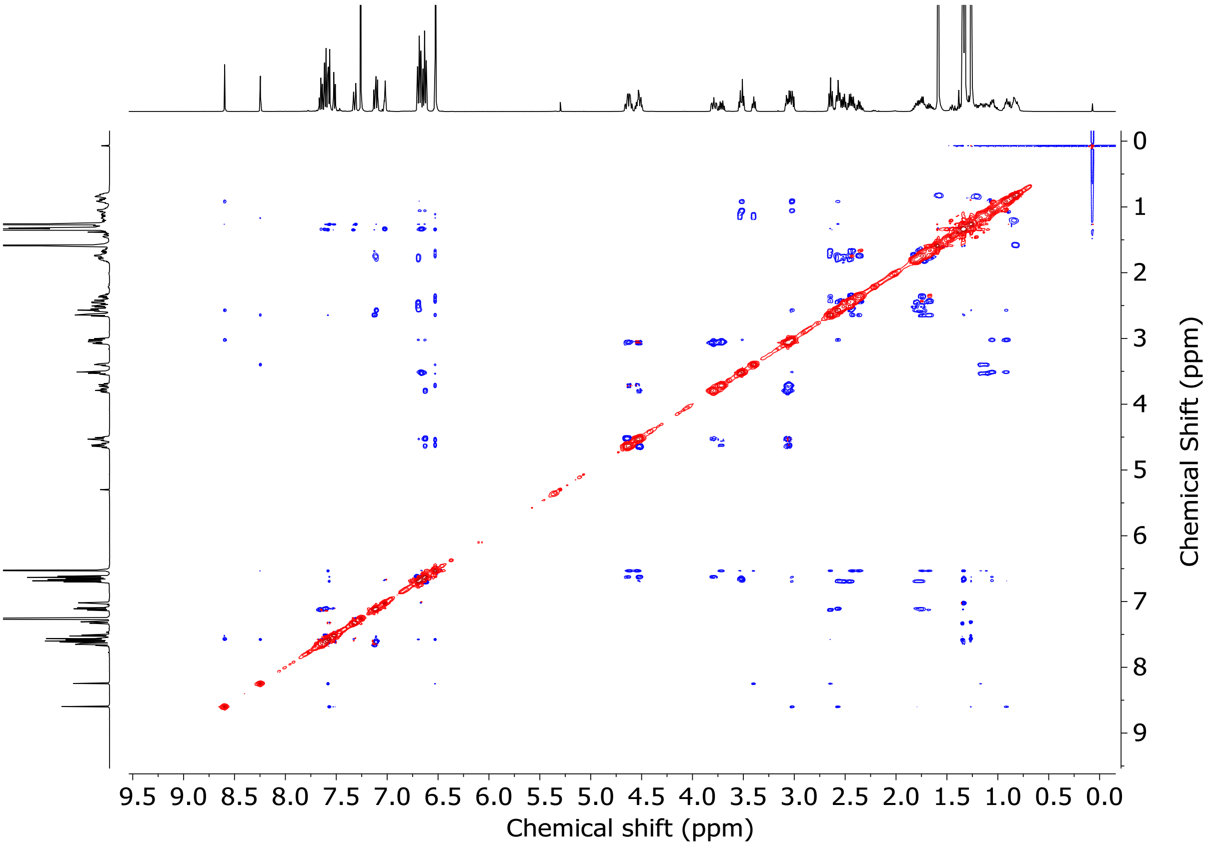

Figure S118: NOESY NMR ($CDCl_3$) of (E_m)-**8** and (Z_m)-**8** (1.4 : 1 *dr*).

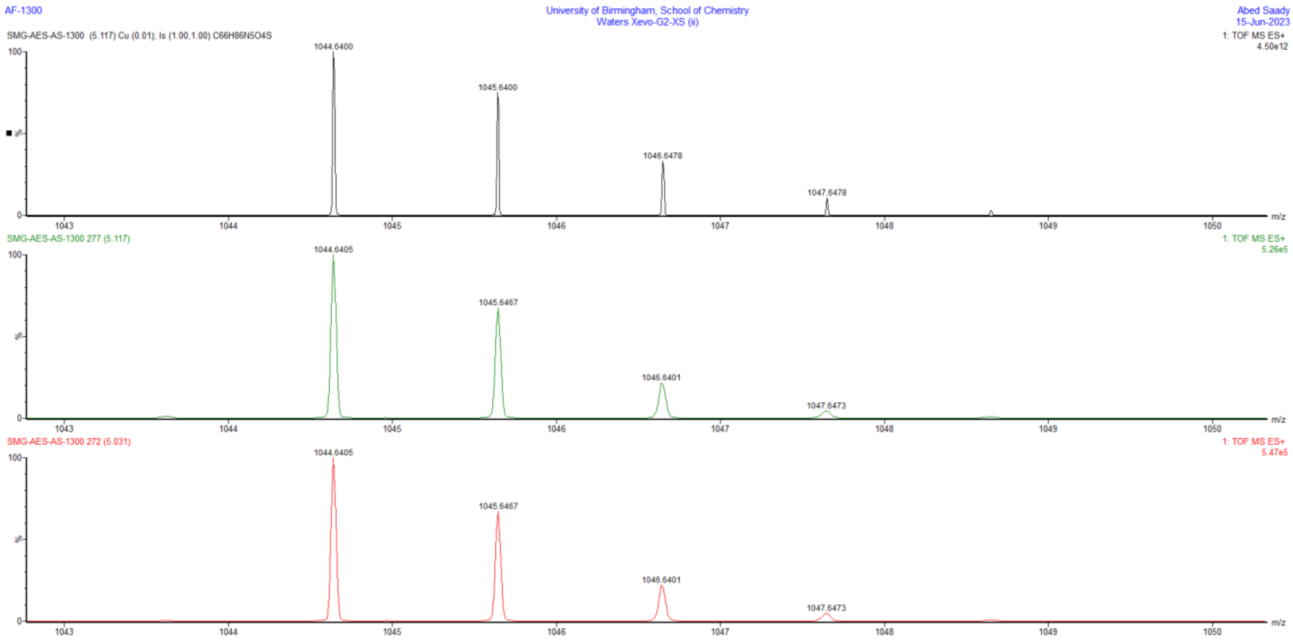

Figure S119: Calculated (top) and observed (middle, bottom) isotopic patterns for (E_m)-**8** and (Z_m)-**8**.

Amide functionalised alkyne S14

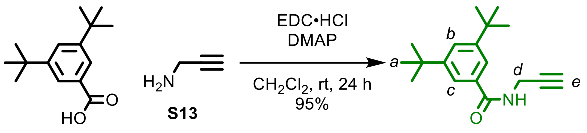

A CEM MW vial was charged with **S12** (140.6 mg, 0.60 mmol), **S13** (40.5 µL, 0.50 mmol), EDC · HCl (191.7 mg, 1.0 mmol), DMAP (2 crystals) and CH_2Cl_2 (5 mL). The reaction mixture was stirred at rt for 24 h. H_2O (10 mL) was added, then the aqueous and organic phases were separated, and the aqueous phase was then extracted with CH_2Cl_2 (3 x 20 mL). The combined organic extracts were washed with brine (10 mL), dried ($MgSO_4$) and concentrated *in vacuo*. Chromatography (petrol-EtOAc 0→60%) gave **S14** as a white solid (141 mg, 95%).

^{1}H NMR (400 MHz, CDCl$_3$) δ: 7.59 (t, *J* = 1.8, 1H, H$_b$), 7.57 (d, *J* = 1.8, 2H, H$_c$), 6.34 (t, *J* = 5.2, 1H, NH), 4.26 (dd, *J* = 2.5, 5.4, 2H, H$_d$), 2.28 (t, *J* = 2.5, 1H, H$_e$), 1.34 (s, 18H, H$_a$).

^{13}C NMR (101 MHz, CDCl$_3$) δ: 168.2, 151,4, 133.4, 126.0, 121.2, 79.8, 71.7, 35.0, 31.4, 29.7.

HR-ESI-MS (+ve) *m/z* = 294.1841 [M+Na]$^+$ (calc. 294.1828 *m/z* for $C_{18}H_{25}NaNO$).

Melting point 135-137 °C.

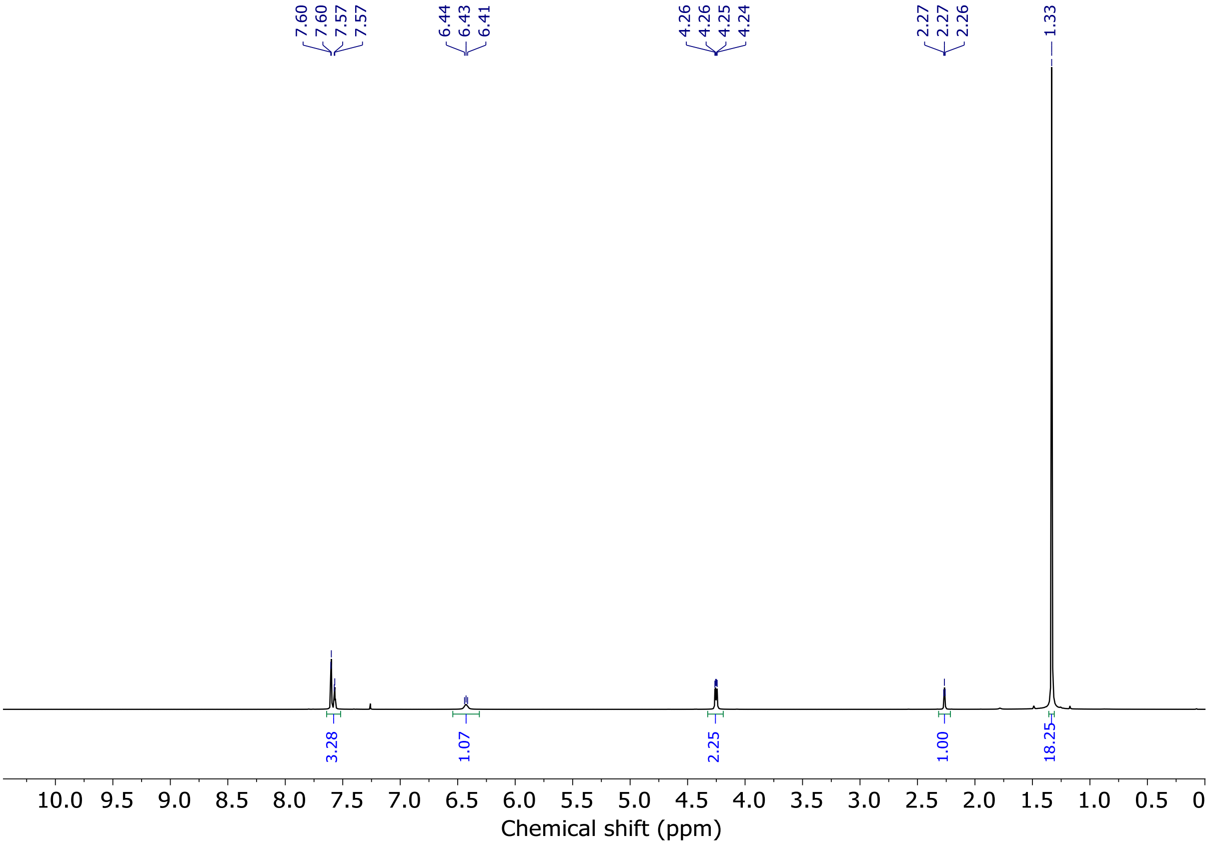

Figure S120: ^{1}H NMR (CDCl$_3$, 400 MHz) of **S14**.

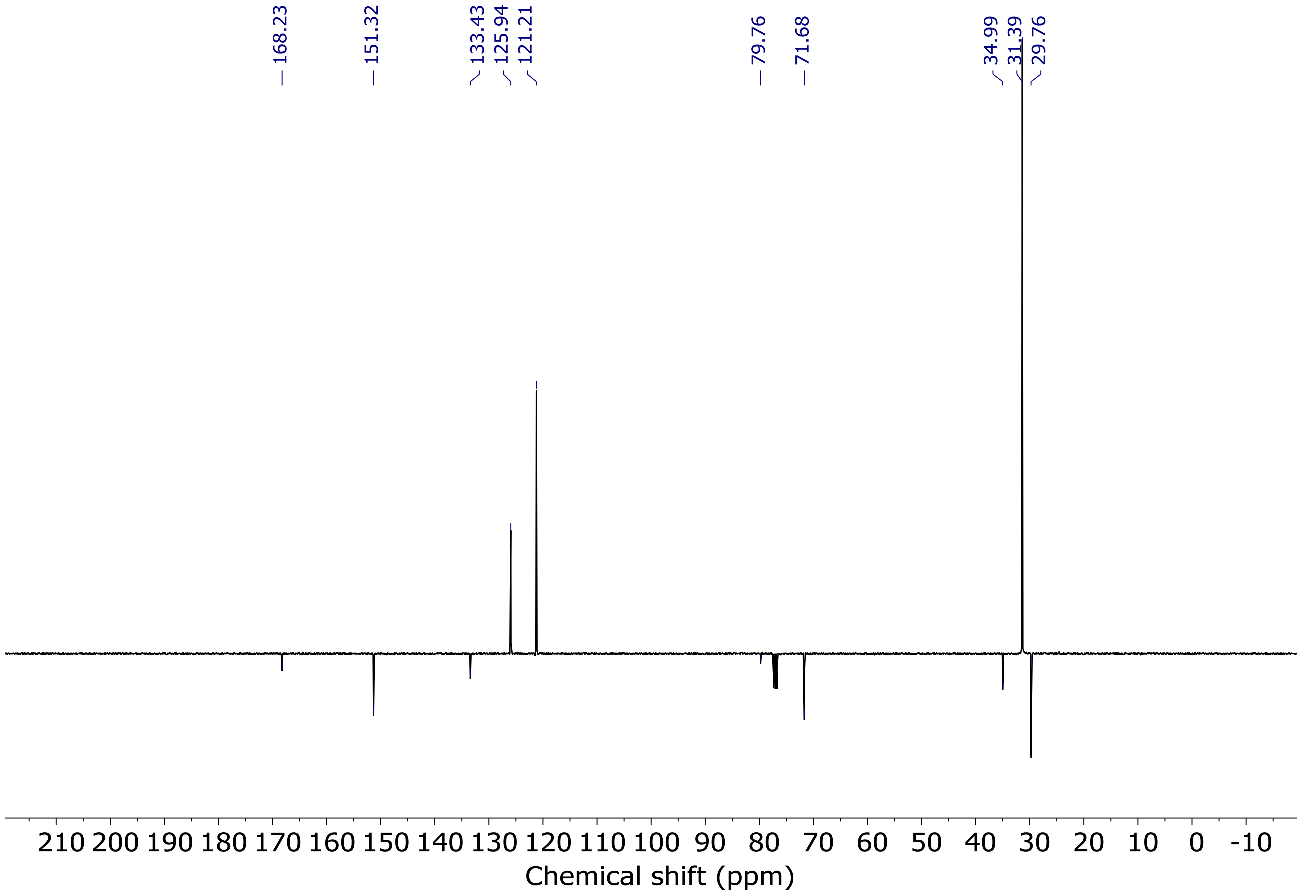

Figure S121: JMOD NMR ($CDCl_3$, 101 MHz) of **S14**.

Amide Rotaxanes (E_m)-9 and (Z_m)-9

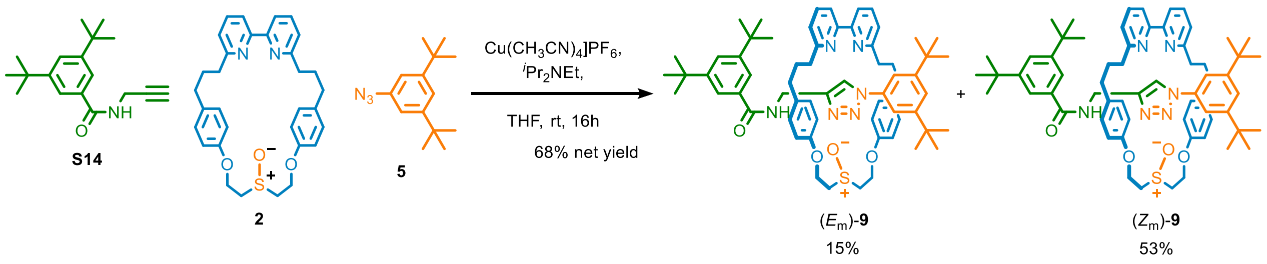

In a CEM vial were added **S14** (11.3 mg, 41.7 µmol), **5** (9.7 mg, 41.7 µmol), **2** (20.0 mg, 38.0 µmol) and [$Cu(CH_3CN)_4PF_6$] (13.6 mg, 36.5 µmol). The vial was sealed and purged with N_2, then THF was added (1.0 mL), followed by ^{i}Pr_2NEt (13.3 µL, 75.9 µmol). The solution was stirred at rt for 16h. the solution was diluted with CH_2Cl_2 (10 mL), then EDTA-NH_3 (5 mL) was added. The solution was vigorously stirred until complete decolouration. The aqueous and organic phases were separated, and the aqueous phase was then extracted with CH_2Cl_2 (3 x 10 mL). The combined organic extracts were washed with brine (10 mL), dried ($MgSO_4$) and concentrated *in vacuo* to give a sample containing **9** as a mixture of diastereomers (19 : 81 *dr*, Figure S122). Two rounds of chromatography (CH_2Cl_2-CH_3CN 0→100% then CH_3CN-MeOH 0→20% followed by petrol-acetone 0→100%) gave (E_m)-**9** as a white foam (5.9 mg, 15%) and (Z_m)-**9** as a white foam (20.6 mg, 53%).

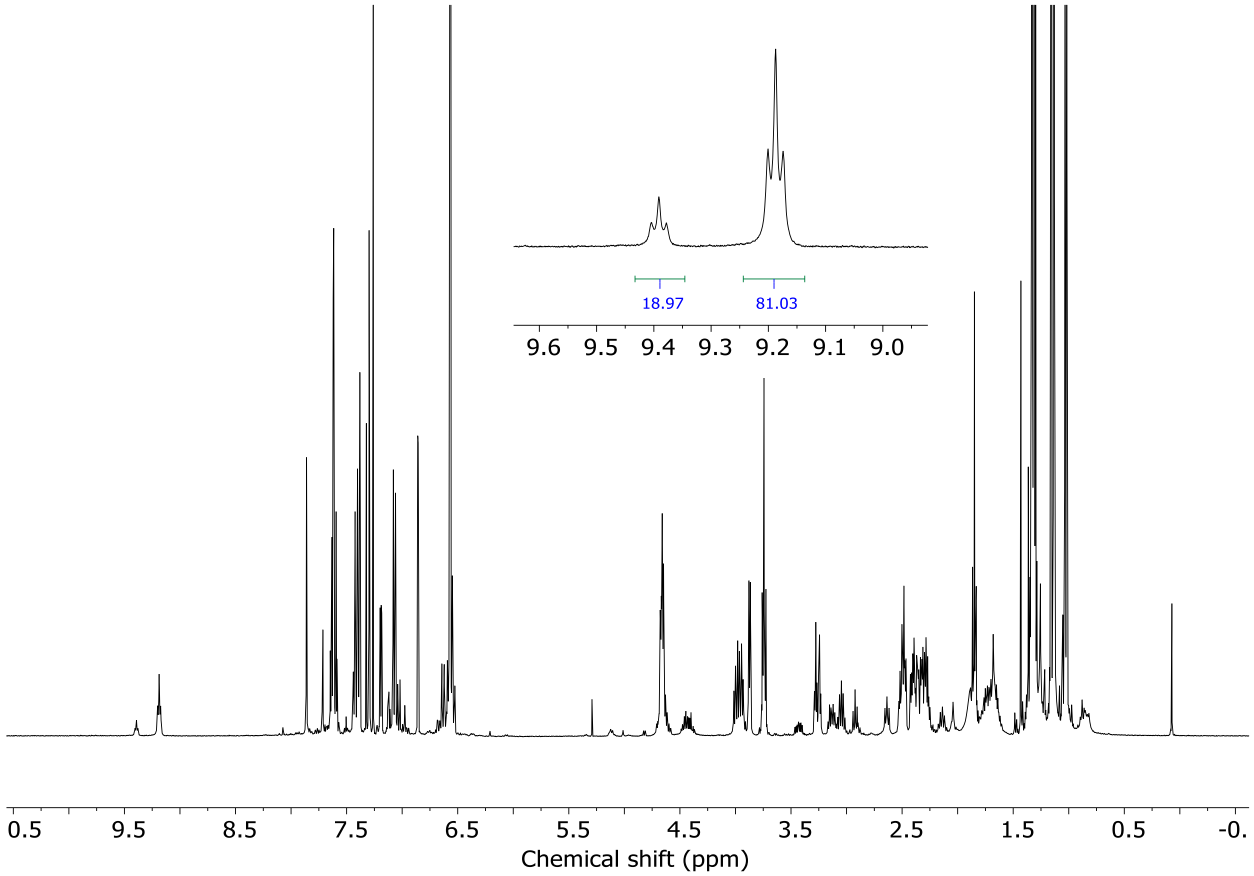

Figure S122: ^{1}H NMR ($CDCl_3$, 400 MHz) of (E_m)-**9** and (Z_m)-**9** prior to chromatography (19 : 81 *dr*).

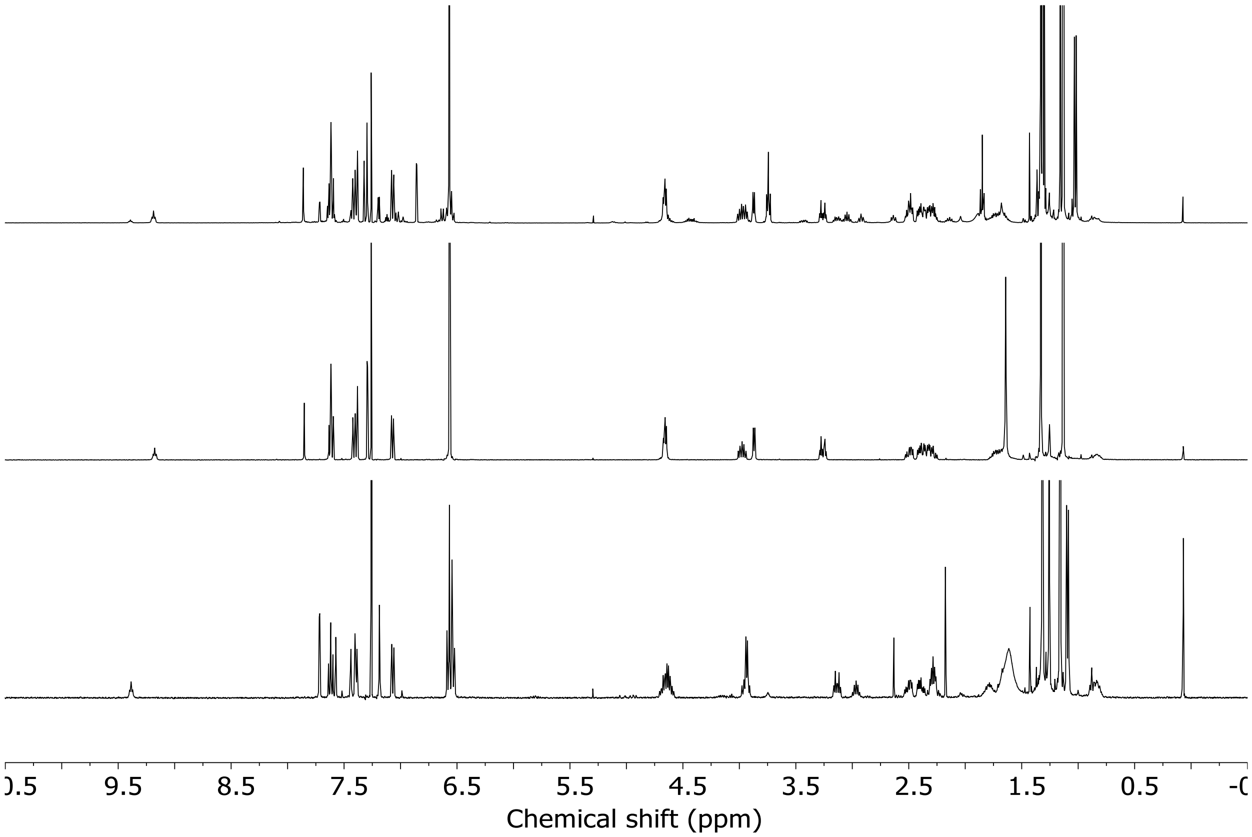

Figure S123: ^{1}H NMR ($CDCl_3$, 400 MHz) of (E_m)-**9** and (Z_m)-**9** prior to chromatography (top), (E_m)-**9** (middle) and (Z_m)-**9** (bottom).

(Z_m)-**9**

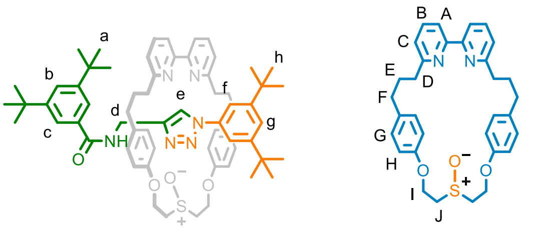

^{1}H NMR (400 MHz, CDCl$_3$) δ: 9.18 (t, J = 6.0, 1H, NH), 7.85 (s, 1H, H_e), 7.64-7.58 (m, 4H, H_c, H_B), 7.42 (t, J = 1.8, H_g), 7.41-7.37 (m, 3H, H_A, H_b), 7.29 (d, J = 1.8, H_f), 7.07 (dd, J = 7.6, 0.9, H_C), 6.56 (s, 8H, H_G, H_H), 4.71-4.61 (m, 4H, H_I), 3.98 (ddd, J = 13.8, 7.8, 6.1, 2H, H_J), 3.87 (d, J = 5.0, 2H, H_d), 3.26 (app. dt, J = 13.8, 4.2, 2H, $H_{J'}$), 2.54-2.44 (m, 2H, H_F), 2.44-2.22 (m, 6H, H_D, $H_{F'}$), 1.81-1.59 (m, 4H, H_E), 1.33 (s, 9H, H_h), 1.13 (s, 9H, H_a).

^{13}C NMR (101 MHz, CDCl$_3$) δ: 167.0, 163.2, 156.9, 155.2, 152.3, 150.0, 145.4, 137.1, 136.9, 134.2, 133.1, 129.2, 124.8, 122.2, 122.0., 121.9, 121.4, 120.0, 115.3, 114.7, 59.8, 51.3, 36.5, 35.1, 35.0, 34.8, 34.8, 31.5, 31.4, 31.3, 29.7.

LR-ESI-MS (+ve) m/z = 1029.6 [M+H]$^+$, for isotopic pattern see Figure S130.

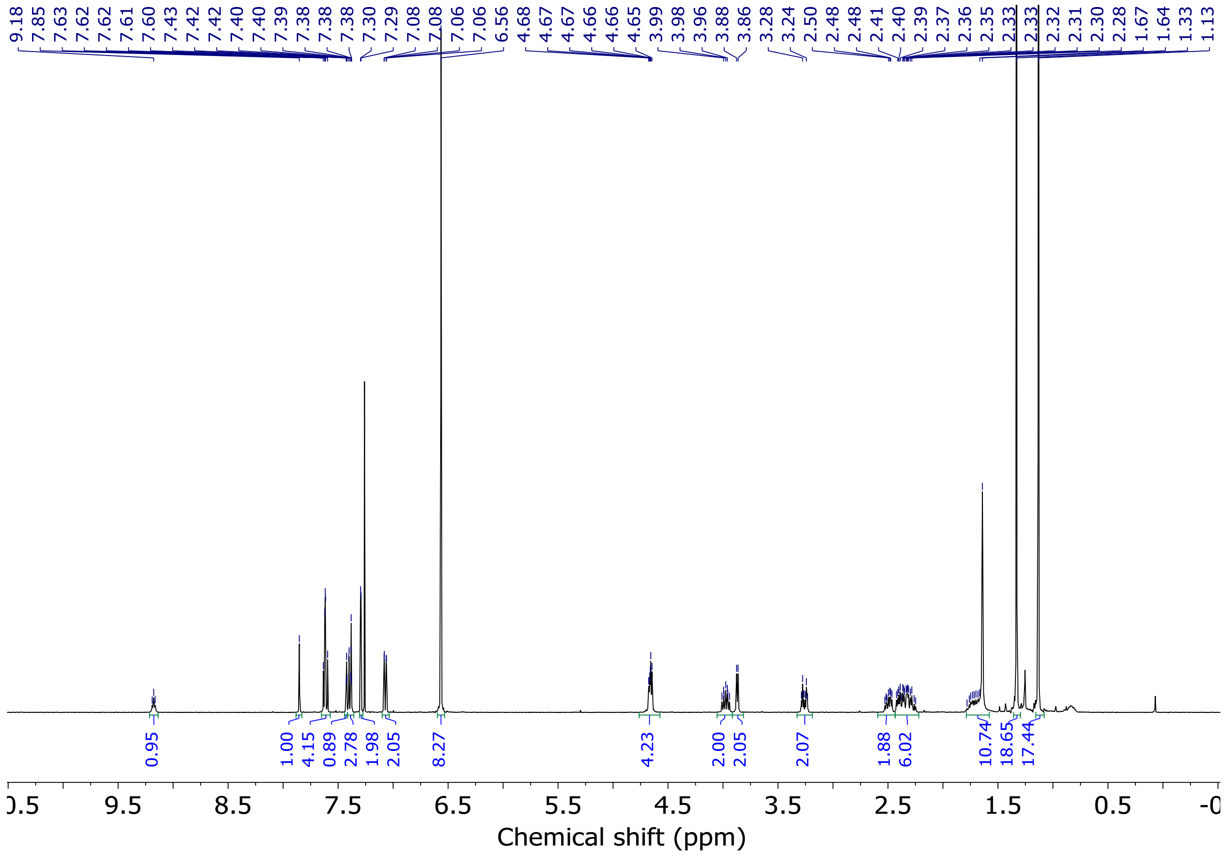

Figure S124: ^{1}H NMR (CDCl$_3$, 400 MHz) of (Z_m)-**9**.

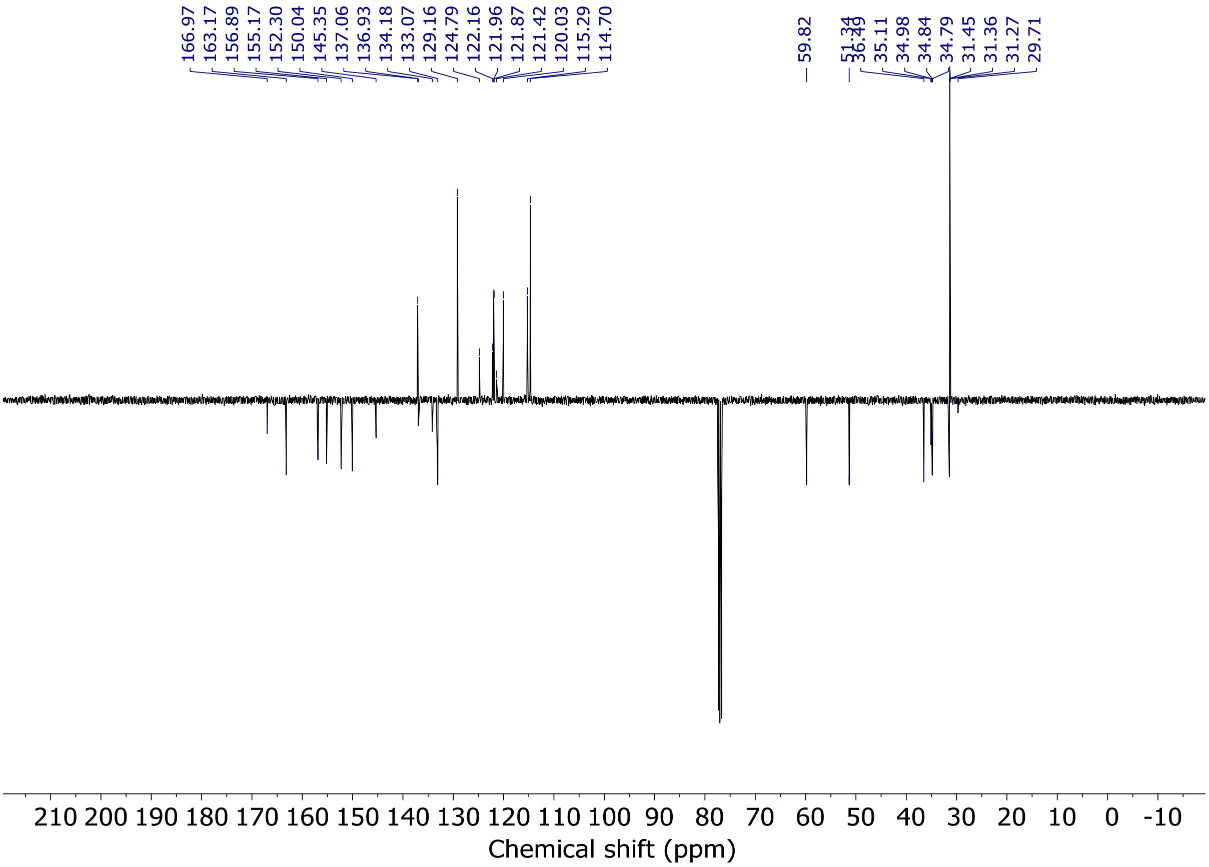

Figure S125: JMOD NMR ($CDCl_3$, 101 MHz) of (Z_m)-**9**.

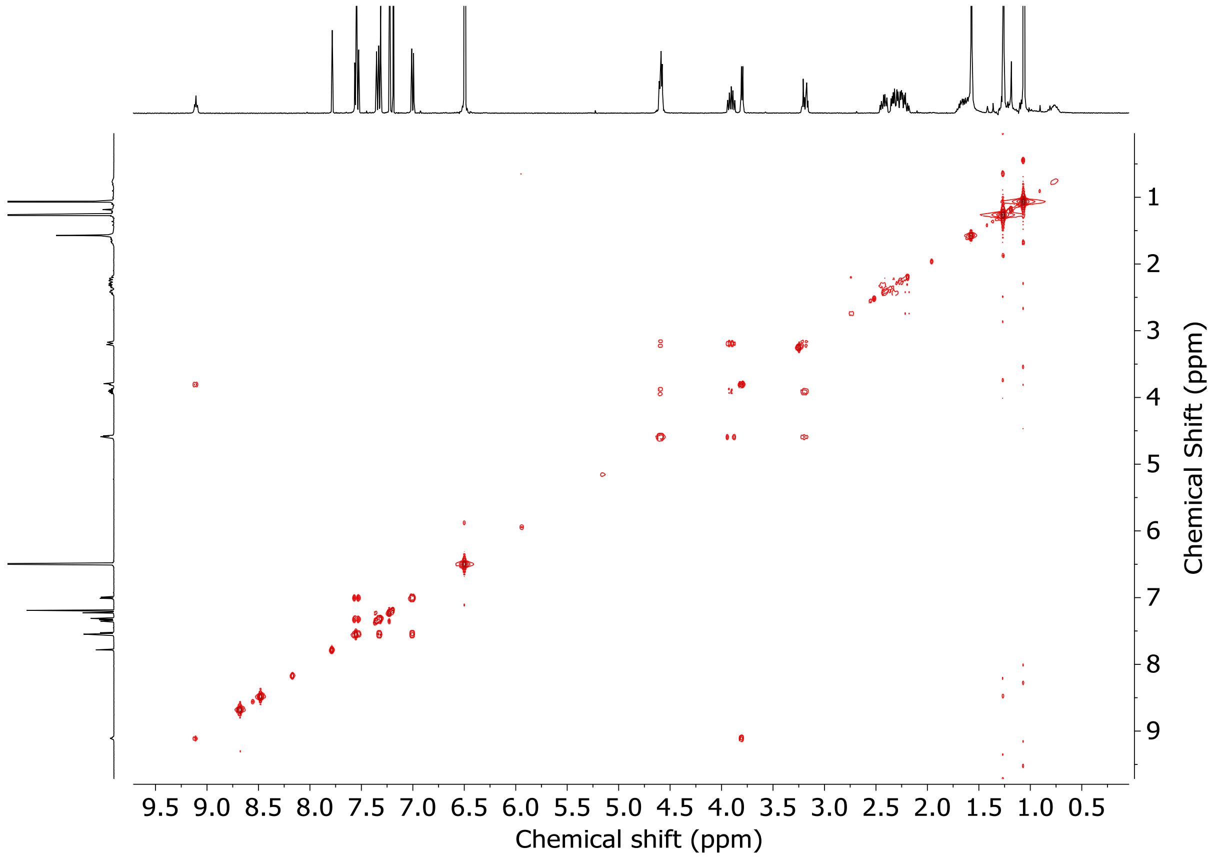

Figure S126: COSY NMR ($CDCl_3$) of (Z_m)-**9**.

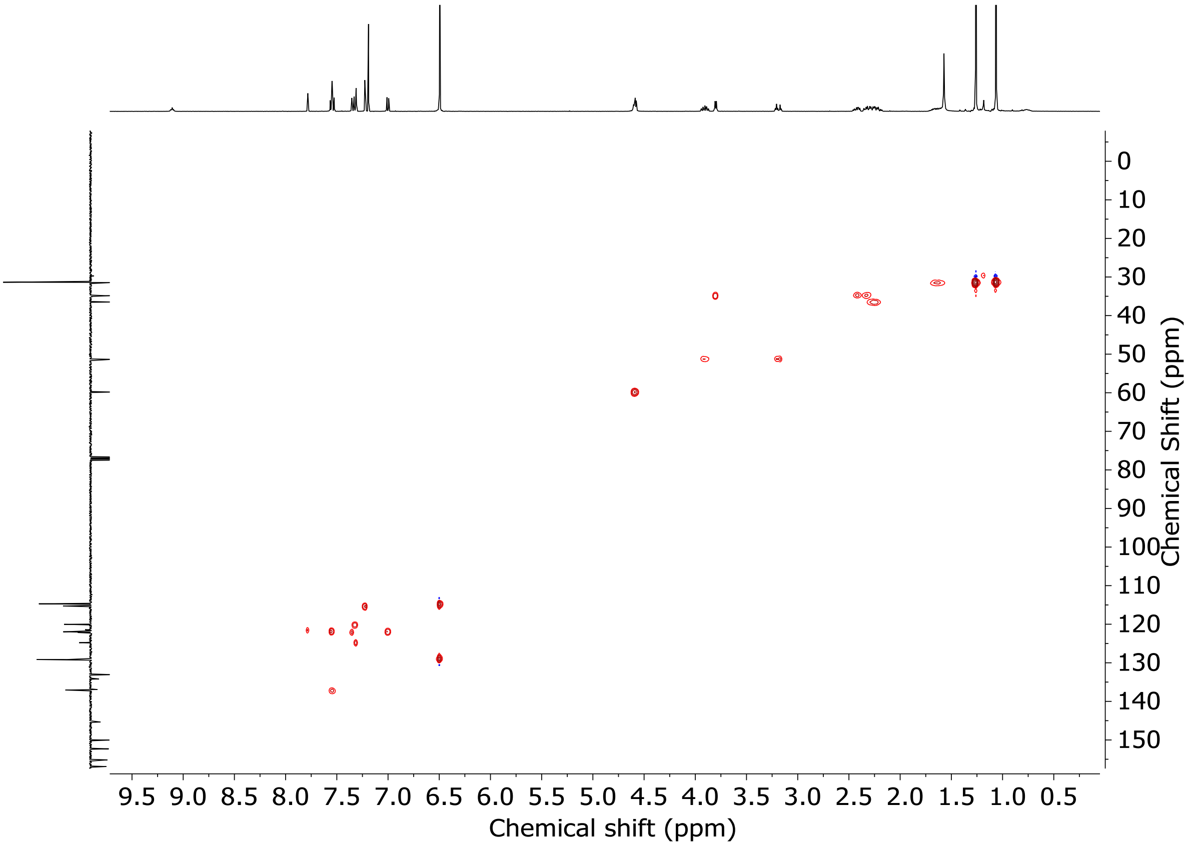

Figure S127: HSQC NMR ($CDCl_3$) of (Z_m)-**9**.

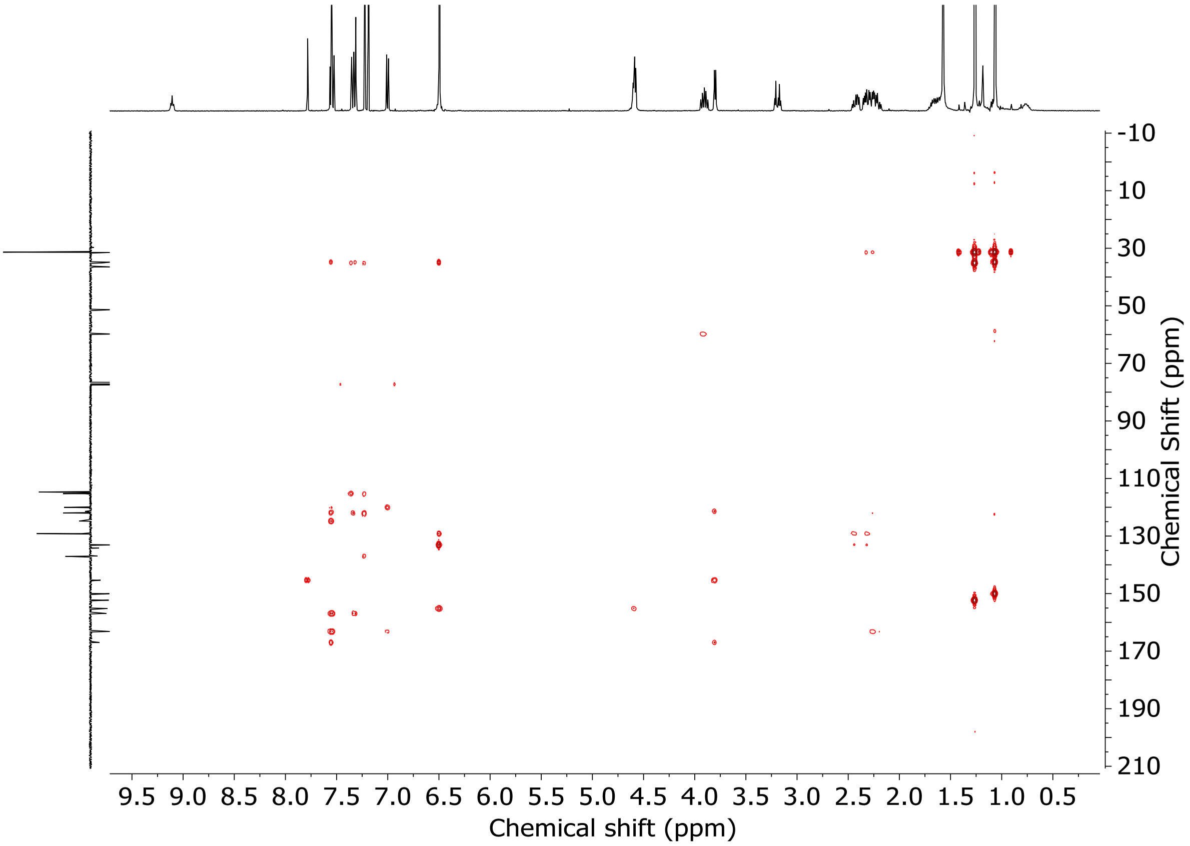

Figure S128: HMBC NMR ($CDCl_3$) of (Z_m)-**9**.

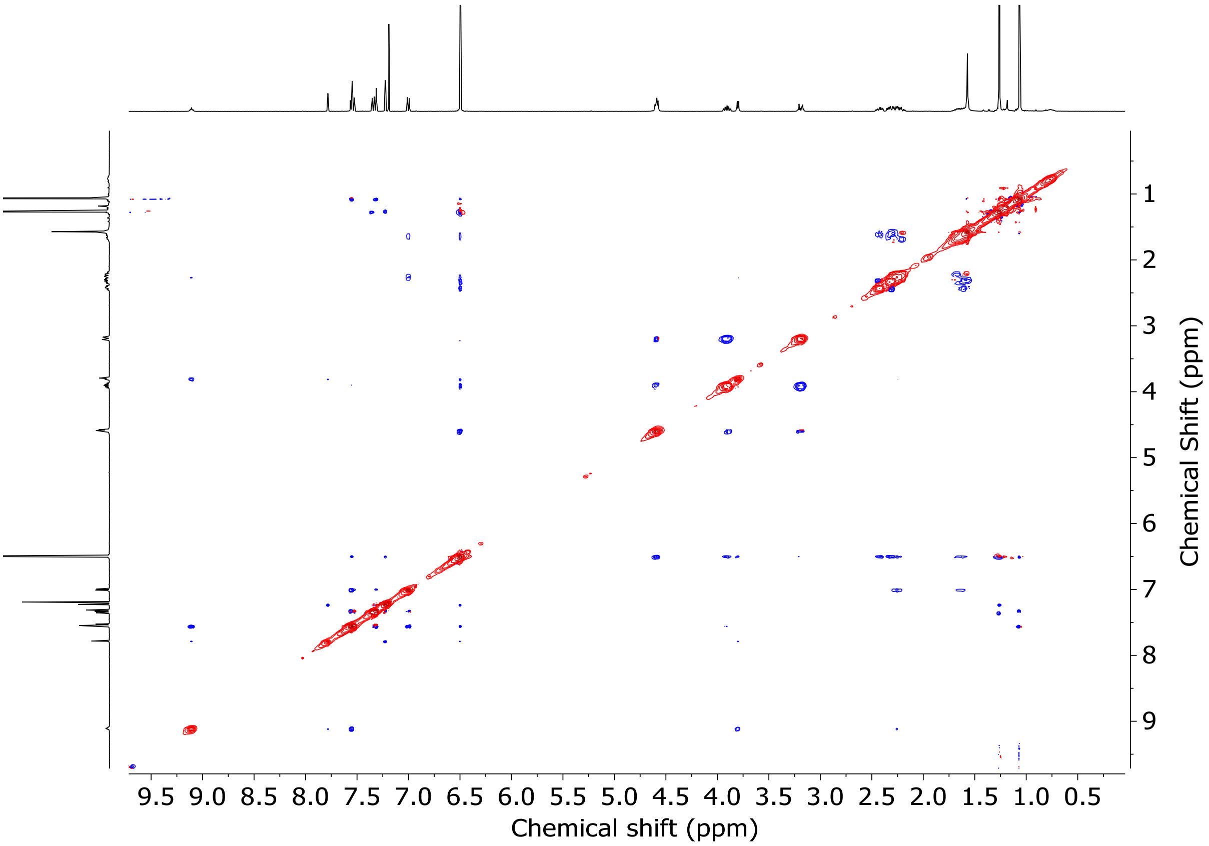

Figure S129: NOESY NMR ($CDCl_3$) of (Z_m)-**9**.

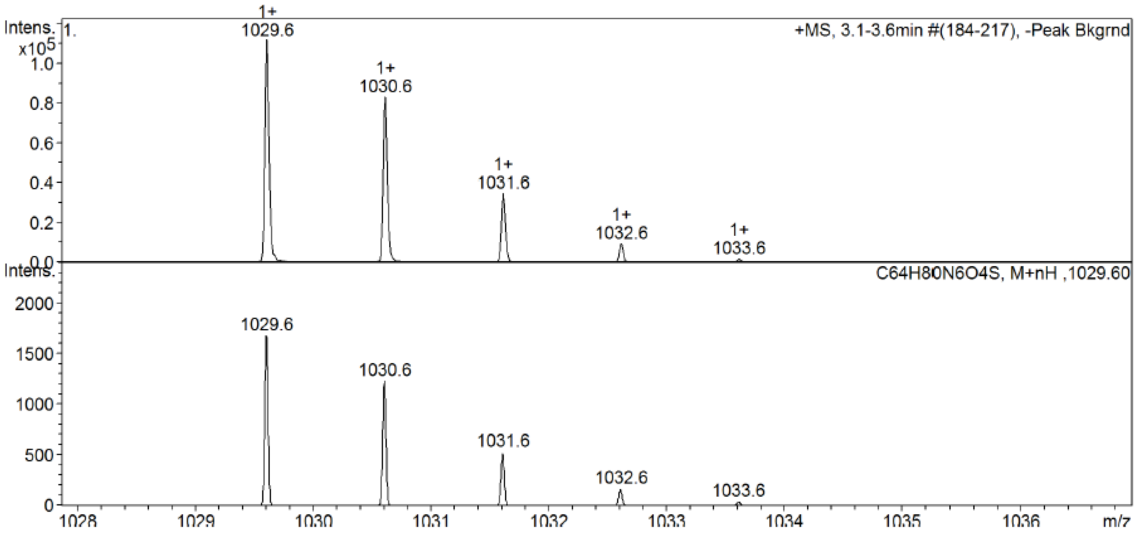

Figure S130: Calculated (top) and observed (bottom) isotopic patterns (Z_m)-**9**.

(*E*$_m$)-**9**

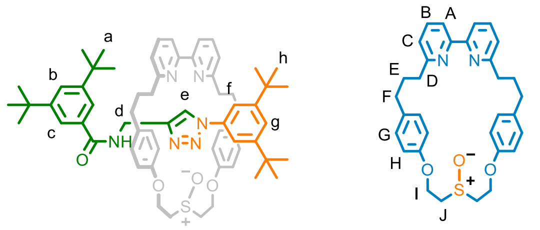

^{1}H NMR (400 MHz, CDCl$_3$) δ: 9.18 (t, *J* = 5.8, 1H, NH), 7.72 (d, *J* = 2.0, 2H, H$_c$), 7.62 (t, *J* = 7.9, 2H, H$_B$), 7.57 (s, 1H, H$_e$), 7.44 (t, *J* = 2.0, 1H, H$_g$), 7.42-7.36 (m, 3H, H$_b$, H$_A$), 7.19 (d, *J* = 1.7, 2H, H$_f$), 7.07 (d, *J* = 8.0, 2H, H$_C$), 6.61 (m, 8H, H$_G$, H$_H$), 5.73-4.56 (m, 4H, H$_I$), 4.00-3.89 (m, 4H, H$_d$, H$_J$), 3.13 (app dt, *J* = 13.0, 5.8, 2H, H$_{J'}$), 2.56-2.45 (m, 2H, H$_F$), 2.45-2.23 (m, 6H, H$_D$ H$_{F'}$), 1.86-1.59 (m, 4H, H$_E$), 1.32 (s, 18H, H$_h$), 1.18 (s, 18H, H$_a$).;

^{13}C NMR (101 MHz, CDCl$_3$) δ: 166.8, 163.2, 156.7, 155.1, 152.4, 149.9, 146.1, 137.1, 133.8, 133.0, 129.1, 124.8, 122.5, 122.2, 121.9, 120.0, 115.5, 115.1, 60.7, 51.5, 36.5, 35.3, 35.1, 34.9, 34.9, 31.4, 31.4, 31.1, 29.3.

LR-ESI-MS *m/z* = 1029.6 [M+H]$^+$, for isotopic pattern see Figure S137.

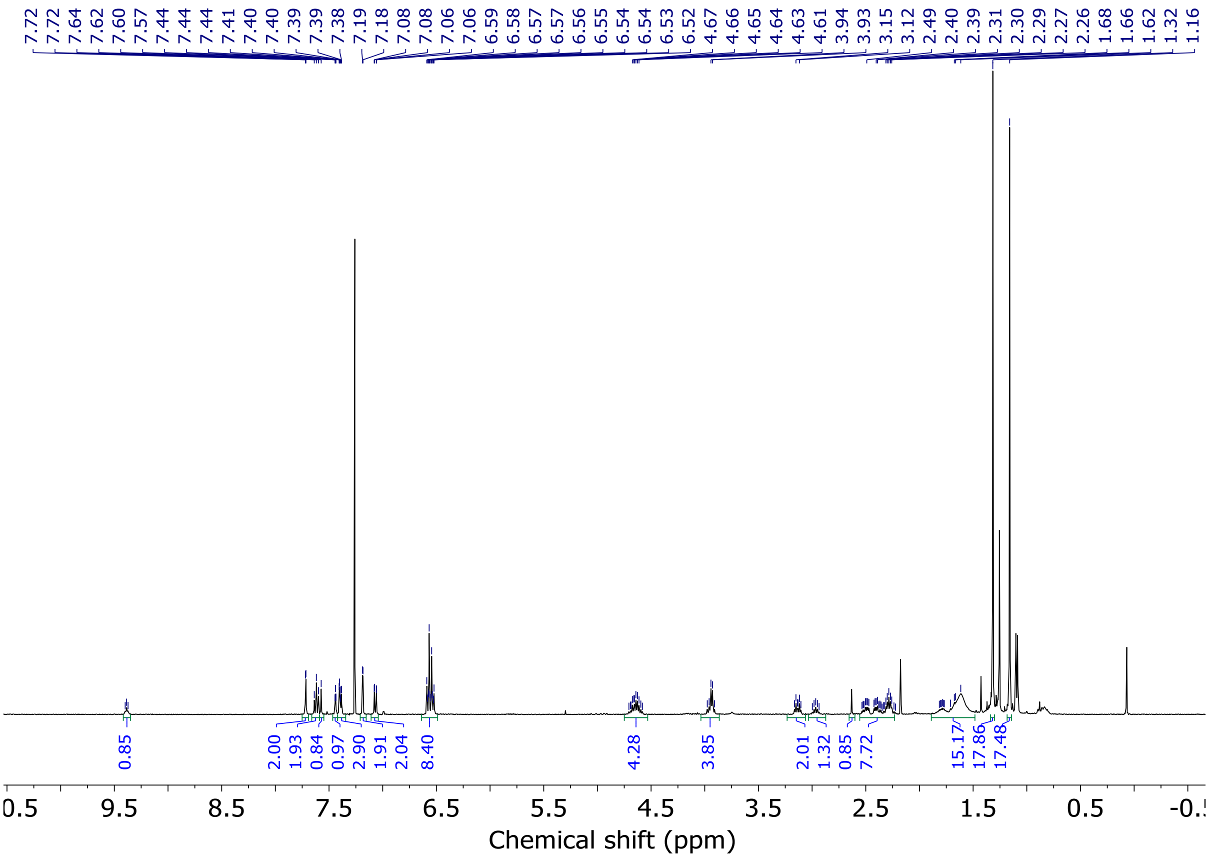

Figure S131: ^{1}H NMR (CDCl$_3$, 400 MHz) of (*E*$_m$)-**9**.

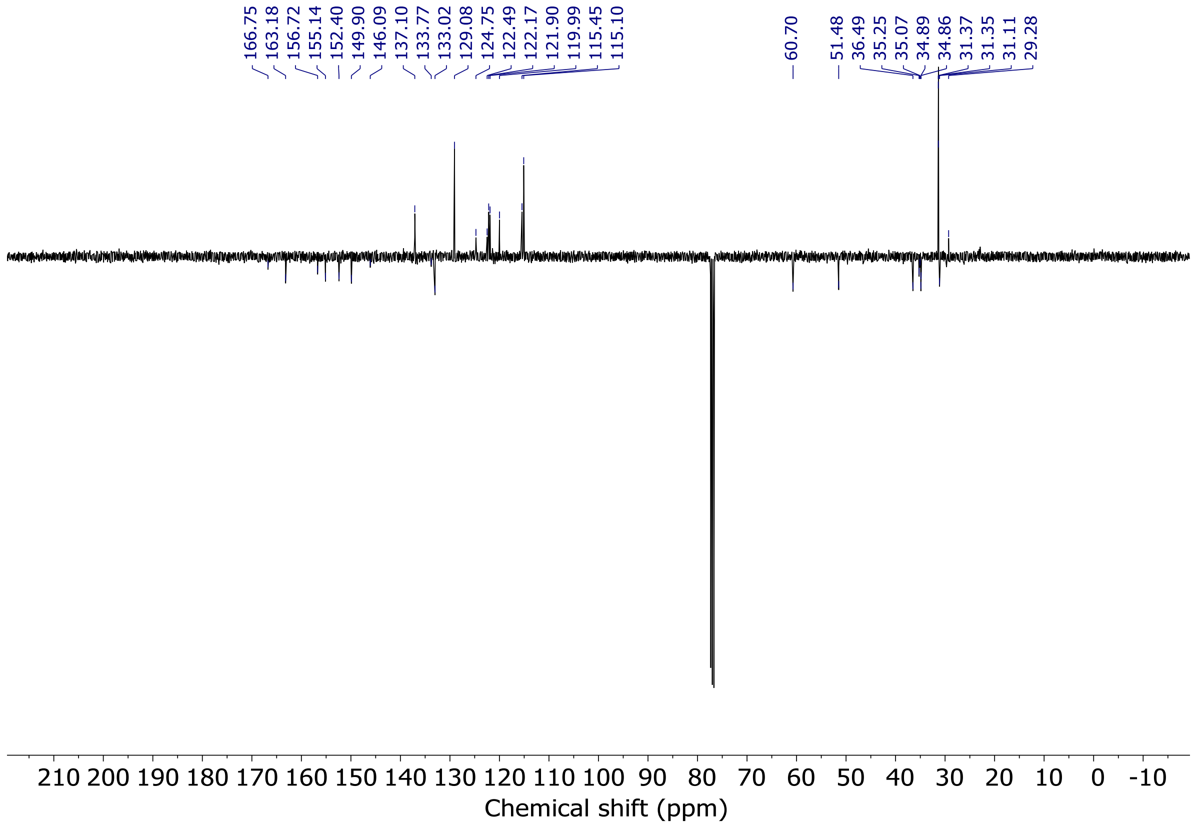

Figure S132: JMOD NMR ($CDCl_3$, 101 MHz) of (E_m)-**9**.

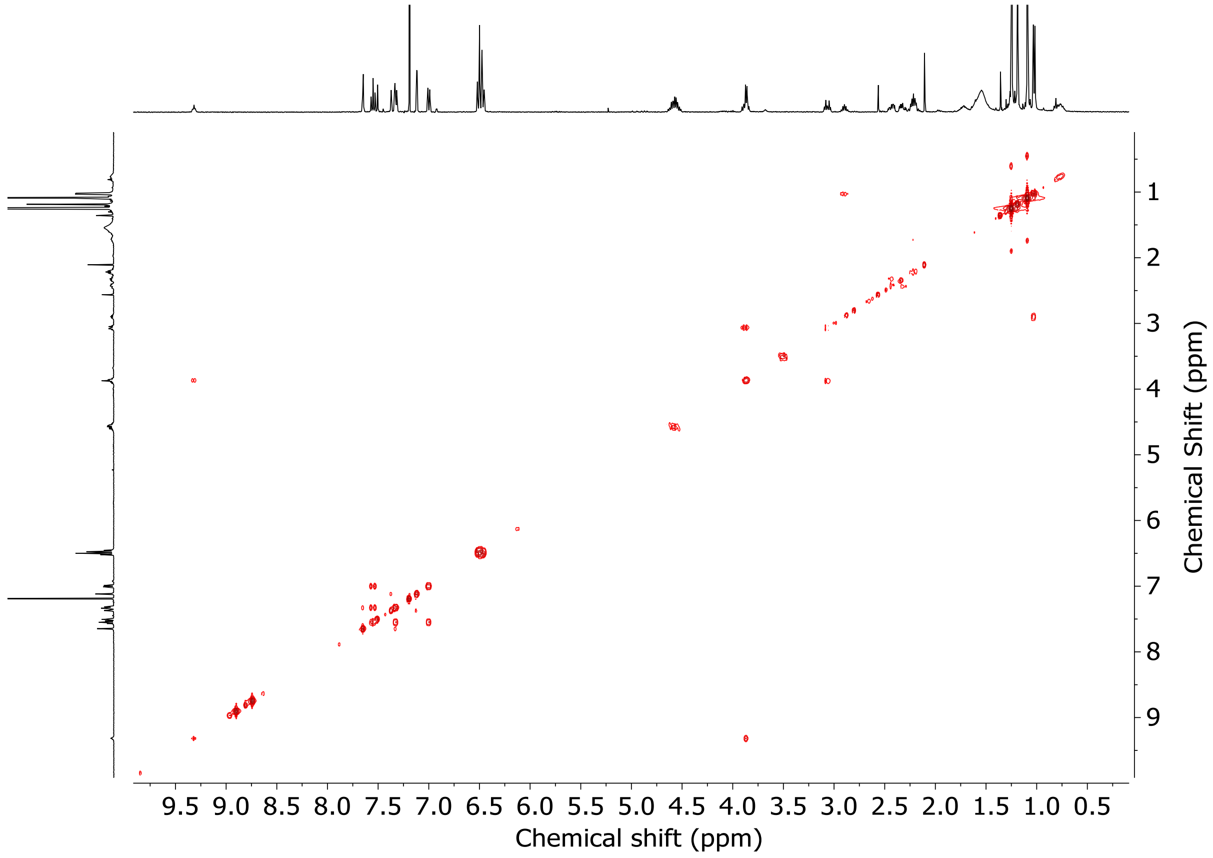

Figure S133: COSY NMR ($CDCl_3$) of (E_m)-**9**.

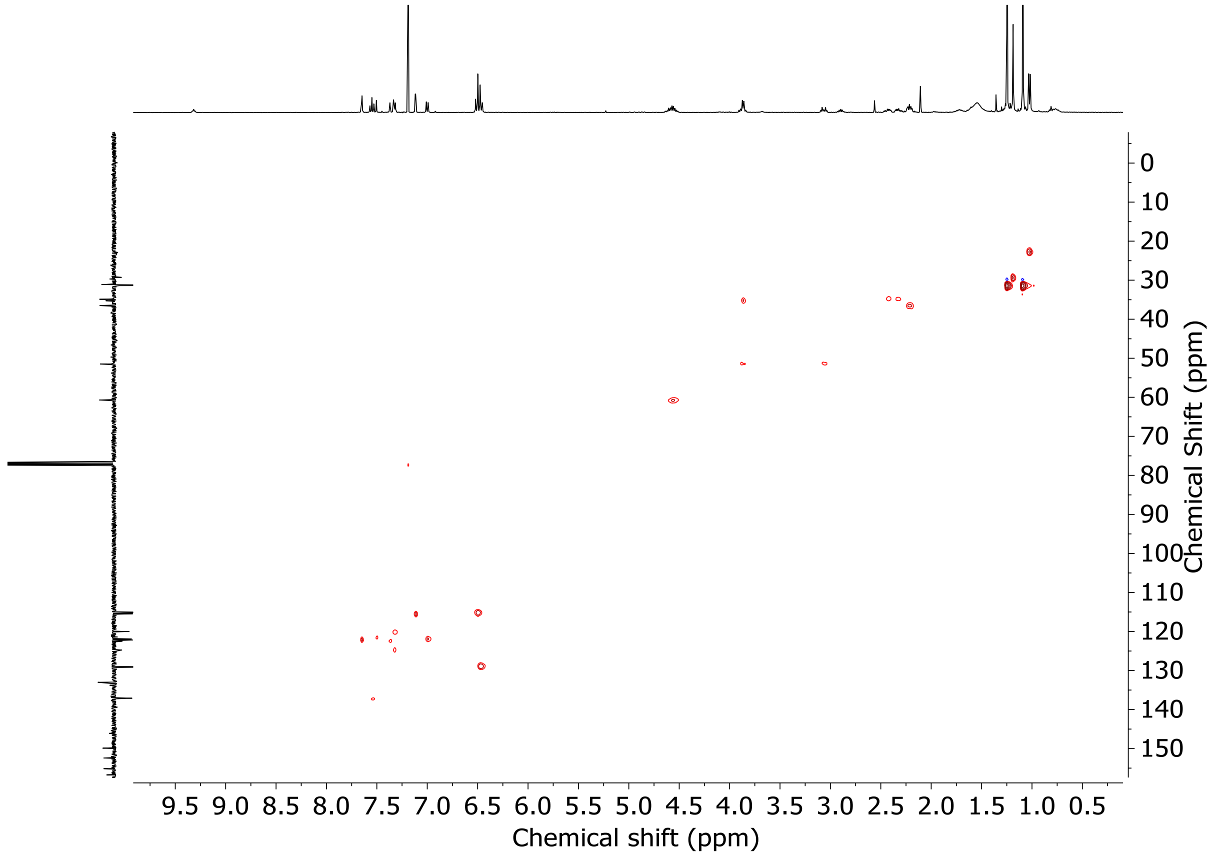

Figure S134: HSQC NMR ($CDCl_3$) of (E_m)-**9**.

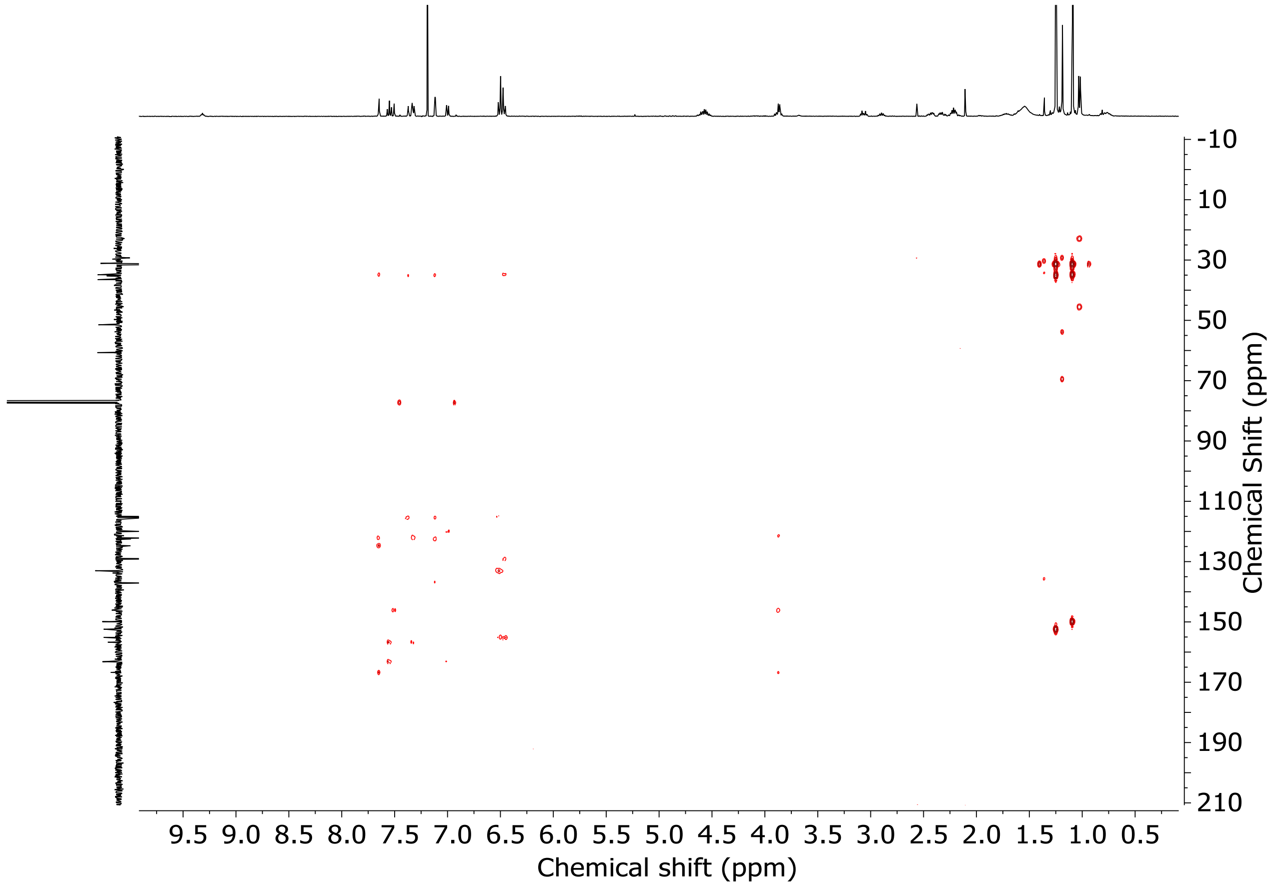

Figure S135: HMBC NMR ($CDCl_3$) of (E_m)-**9**.

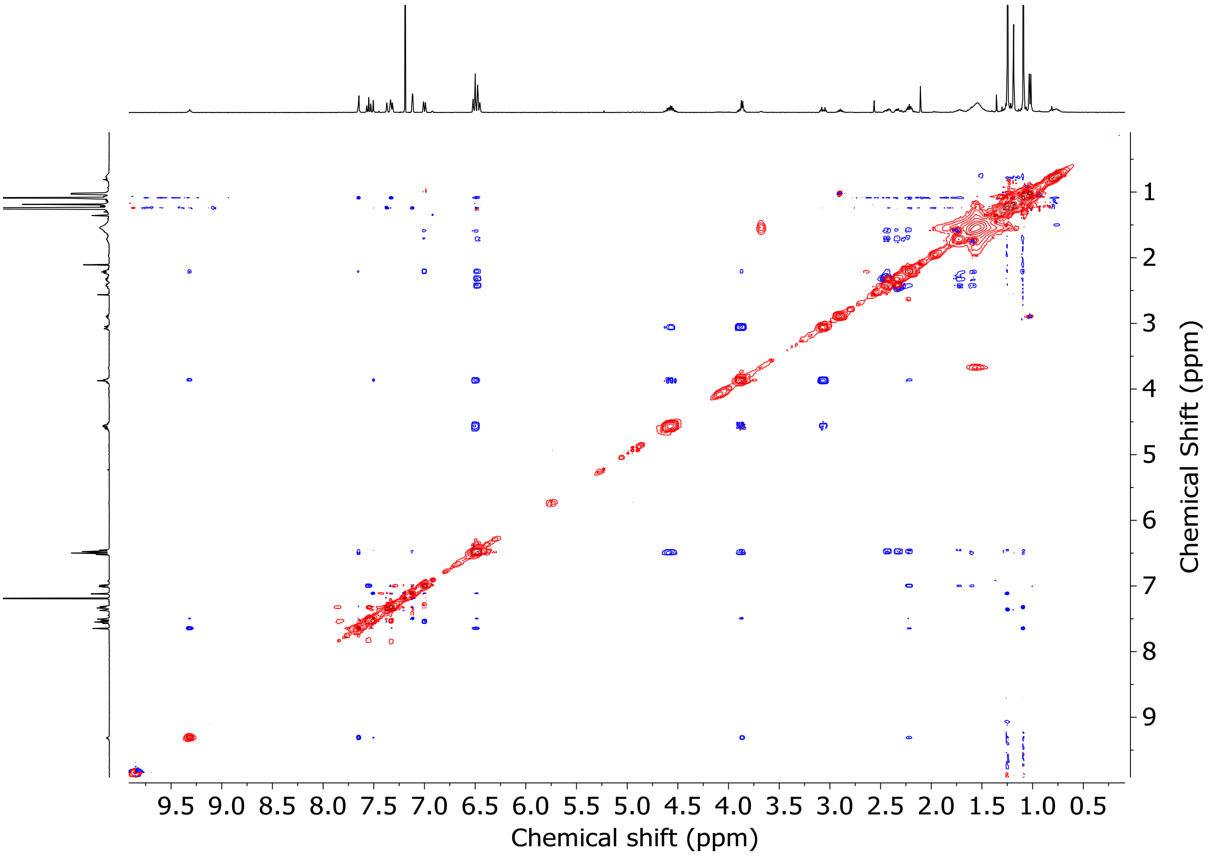

Figure S136: NOESY NMR ($CDCl_3$) of (E_m)-**9**.

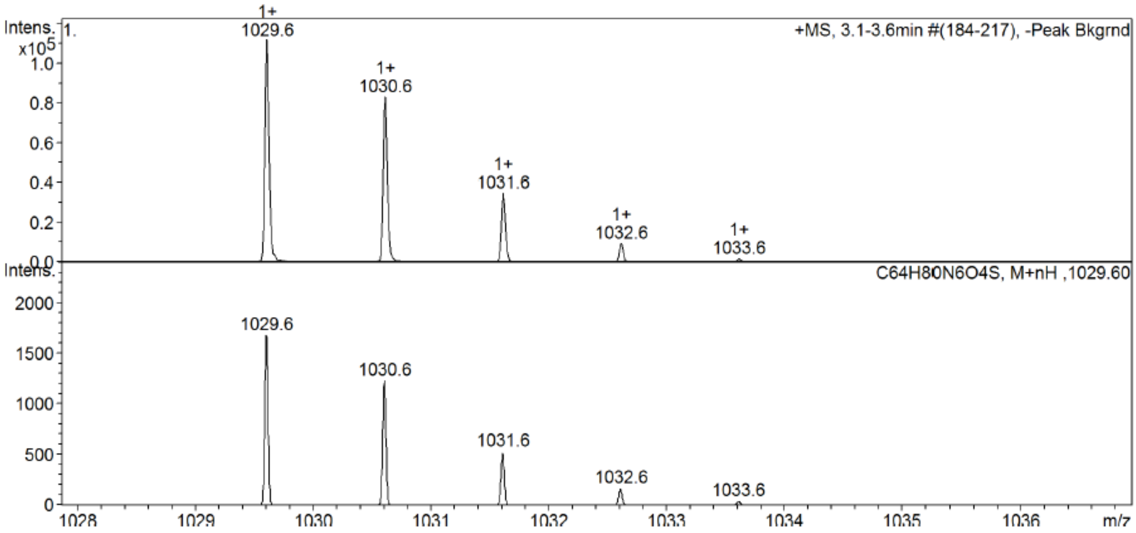

Figure S137: Calculated (top) and observed (middle, bottom) isotopic patterns for (E_m)-**9**.

Methylated amide alkyne S15

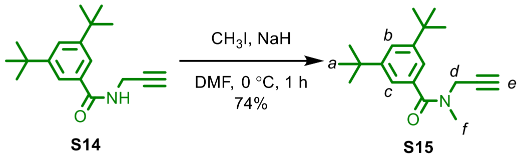

To a suspension of NaH (60% in mineral oil, 12.6 mg, 0.37 mmol) in DMF (2 mL) was added a solution of **S14** (50 mg, 0.18 mmol) in DMF (1 mL) dropwise at 0 °C. The reaction mixture was stirred for 10 minutes, then CH_3I (17.2 µL, 0.27 mmol) was added. The reaction mixture was allowed to warm to rt and stirred for 1 h. The reaction mixture was then quenched by adding H_2O (5 mL) dropwise. The aqueous and organic phases were separated, and the aqueous phase was then extracted with Et_2O (3 x 20 mL). The combined organic extracts were washed with brine (10 mL), dried ($MgSO_4$) and concentrated *in vacuo*. Chromatography (petrol-EtOAc 0→100%) gave **S15** as a colourless oil (37.9 mg, 74%) as a mixture of rotamers.

^{1}H NMR (400 MHz, CDCl$_3$) δ: 7.45 (t, *J* = 2.0, 2H, H_b, $H_{b'}$), 7.41-7.13 (m, 4H, H_c, $H_{c'}$), 4.53-3.79 (m, 4H, H_d, $H_{d'}$), 3.23-2.89 (m, 6H, H_f, $H_{f'}$), 2.43-2.20 (m, 2H, H_f, $H_{f'}$), 1.30 (s, 36H, H_a, $H_{a'}$)

^{13}C NMR (101 MHz, CDCl$_3$) δ: 172.3, 150.9, 134.6, 124.0, 121.3, 78.9, 72.9, 72.1, 72.1, 41.7, 36.9, 36.4, 35.0, 32.8, 31.4.

HR-ESI-MS (+ve) *m/z* = 286.2167 [M+H]$^+$ (calc. 286.2165 *m/z* for $C_{18}H_{28}NO$).

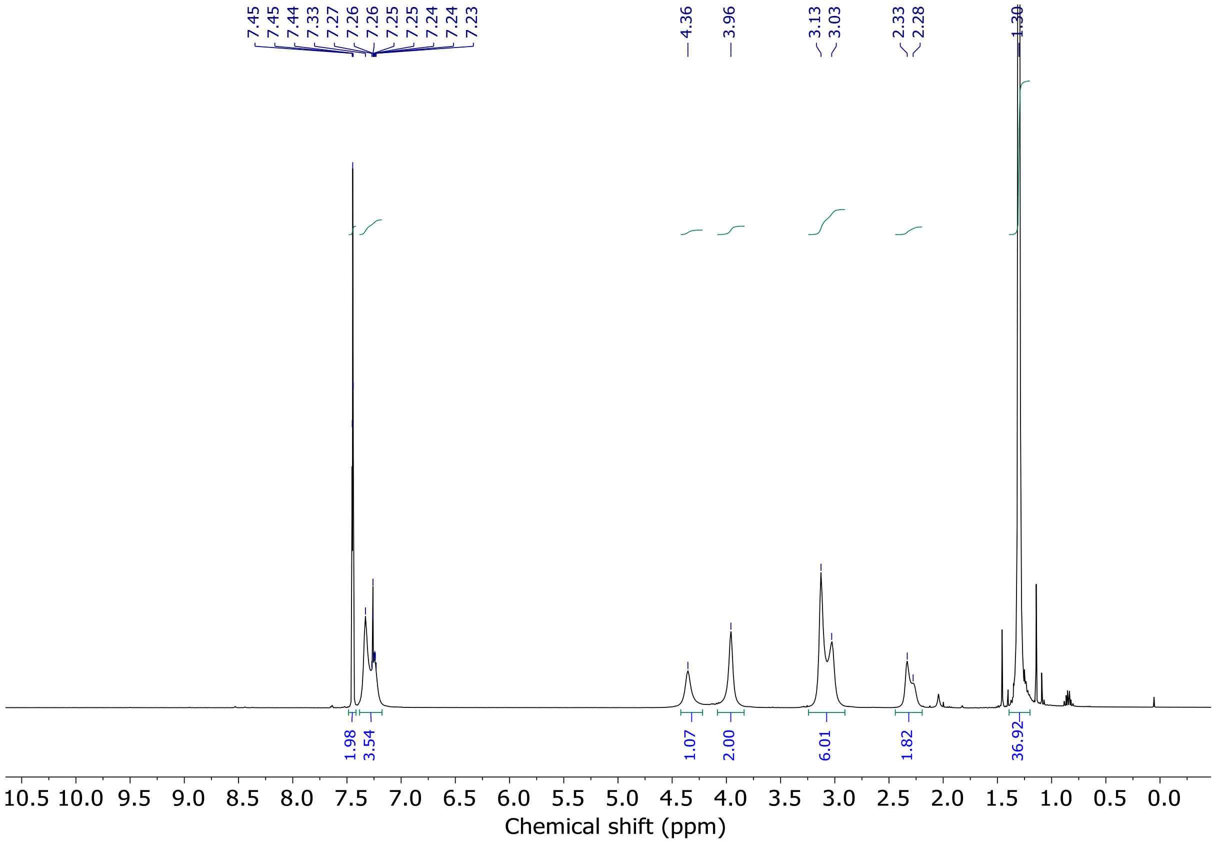

Figure S138: ^{1}H NMR (CDCl$_3$, 400 MHz) of **S15**.

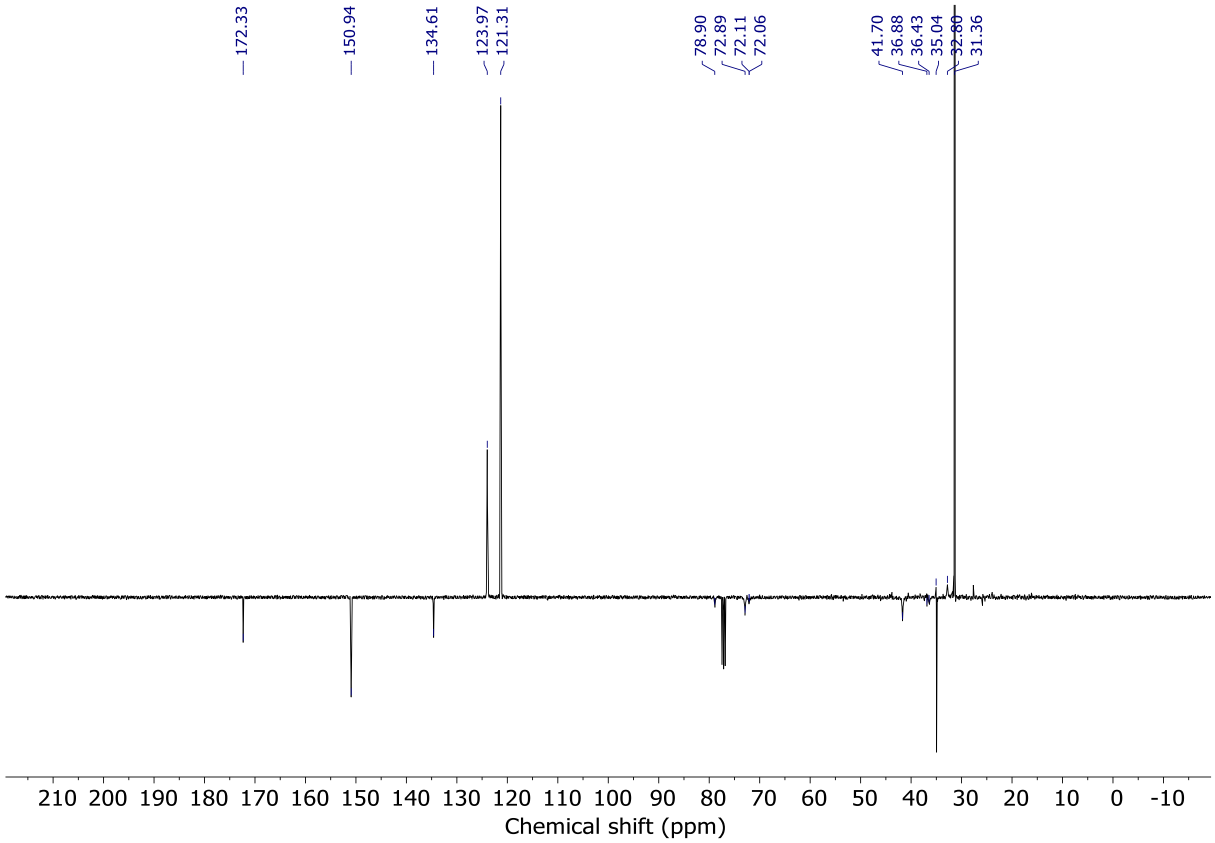

Figure S139: JMOD NMR ($CDCl_3$, 101 MHz) of **S15**.

Methylated Amide Rotaxanes (E_m)-10 and (Z_m)-10

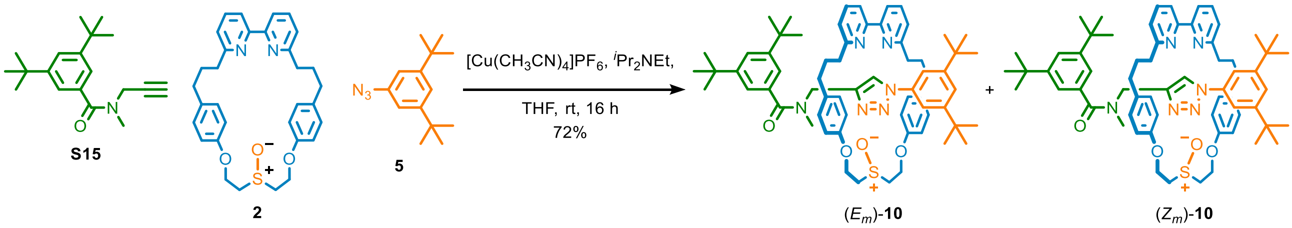

In a CEM vial were added the **S15** (11.9 mg, 41.7 µmol), **5** (9.7 mg, 41.7 µmol), **2** (20.0 mg, 38.0 µmol) and [$Cu(CH_3CN)_4PF_6$] (13.6 mg, 36.5 µmol). The vial was sealed and purged with N_2, then THF was added (1.0 mL), followed by ${}^{i}Pr_2NEt$ (13.3 µL, 75.9 µmol). The solution was stirred at rt for 16h. the solution was diluted with CH_2Cl_2 (5 mL), then EDTA-NH_3 (5 mL) was added. The solution was vigorously stirred until complete decolouration. The aqueous and organic phases were separated, and the aqueous phase was then extracted with CH_2Cl_2 (3 x 10 mL). The combined organic extracts were washed with brine (10 mL), dried ($MgSO_4$) and concentrated *in vacuo* to give a sample containing **10** as a mixture of diastereomers (44 : 56 *dr*, Figure S140). Chromatography (CH_2Cl_2-CH_3CN 0→100% then CH_3CN-MeOH 0→20%) gave rotaxane **10** as a mixture of (E_m)-**10** and (Z_m)-**10** (28.6 mg, 72%) as a colourless oil. Analytical samples of the single diastereoisomers were obtained *via* column chromatography (*n*-hexane-acetone 0→100%).

Due to the presence of complex rotameric mixture it wasn't possible to assign the NMR spectra of the compounds. However, the purity of the compound was confirmed by LR-MS of the separated fractions (Figure S143). The diastereoisomeric ratios observed in the solvent screening were assigned by integrating the de-shielded peaks corresponding to the rotameric mixture of the isolated species.

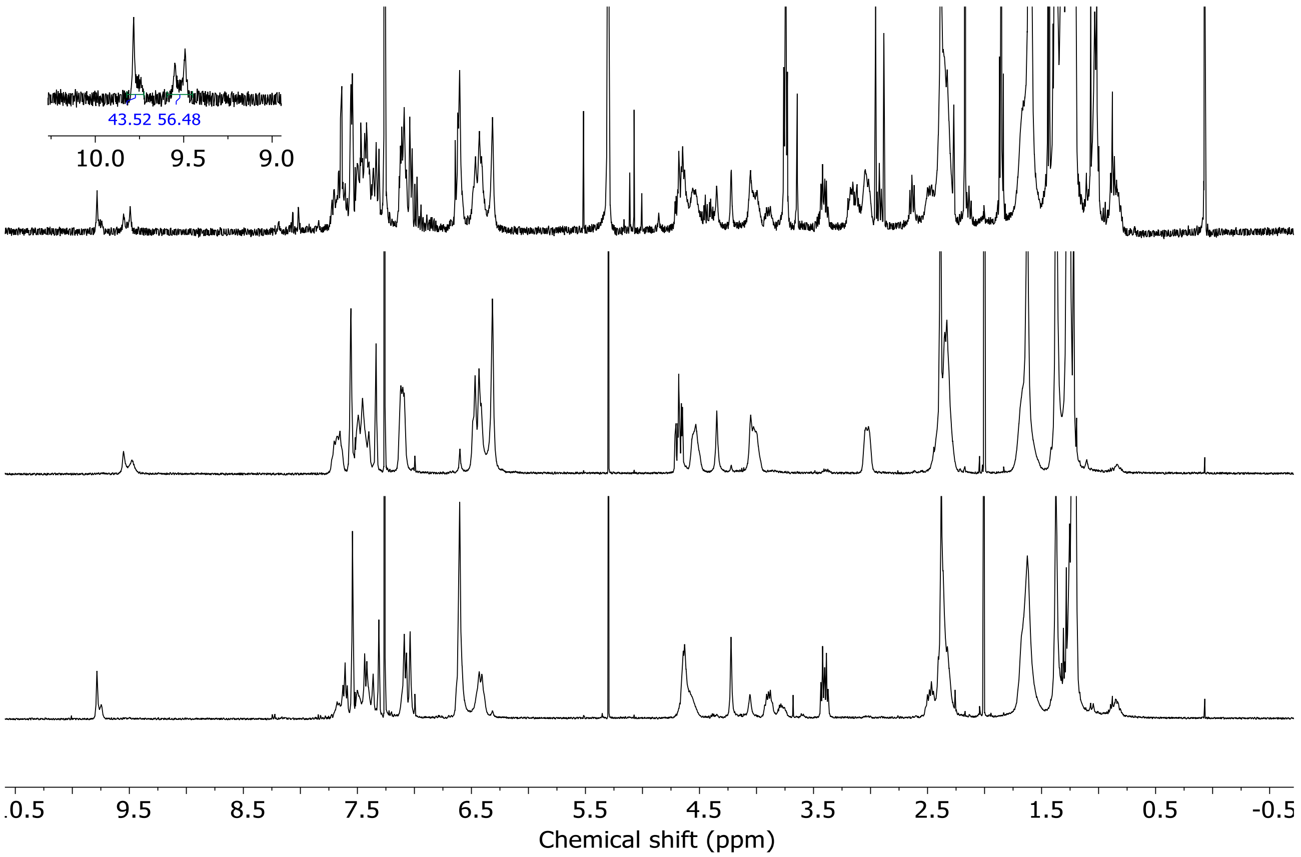

Figure S140. ^{1}H NMR ($CDCl_3$, 400 MHz) of (E_m)-**10** and (Z_m)-**10** prior to chromatography (top), (E_m)-**10** (middle) and (Z_m)-**10** (bottom).

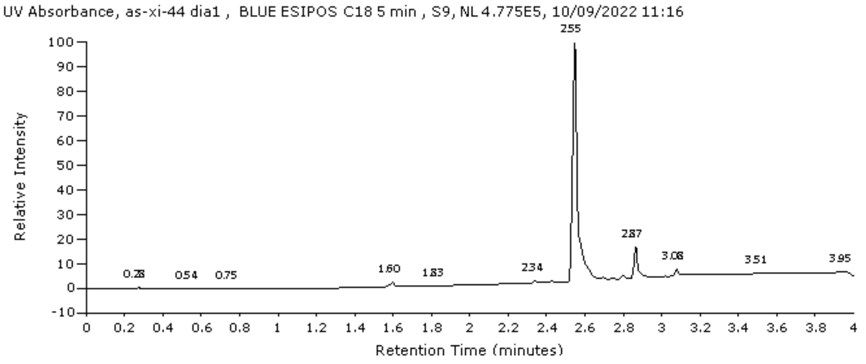

Figure S141. LC-MS of **10** (retention times: 2.55 mins, *m/z* = 1044.0 and 2.87 mins, *m/z* = 1044.0)

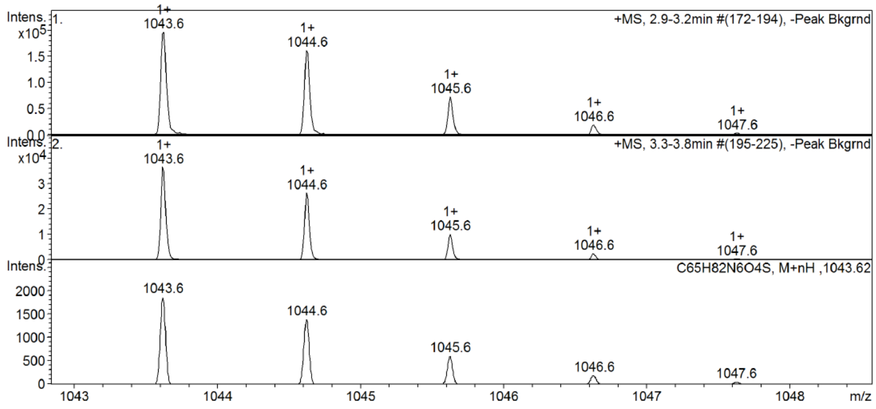

Figure S142. ESI-HR-MS of one the isolated isomer for **10** and experimanetal and calculated isotopic pattern.

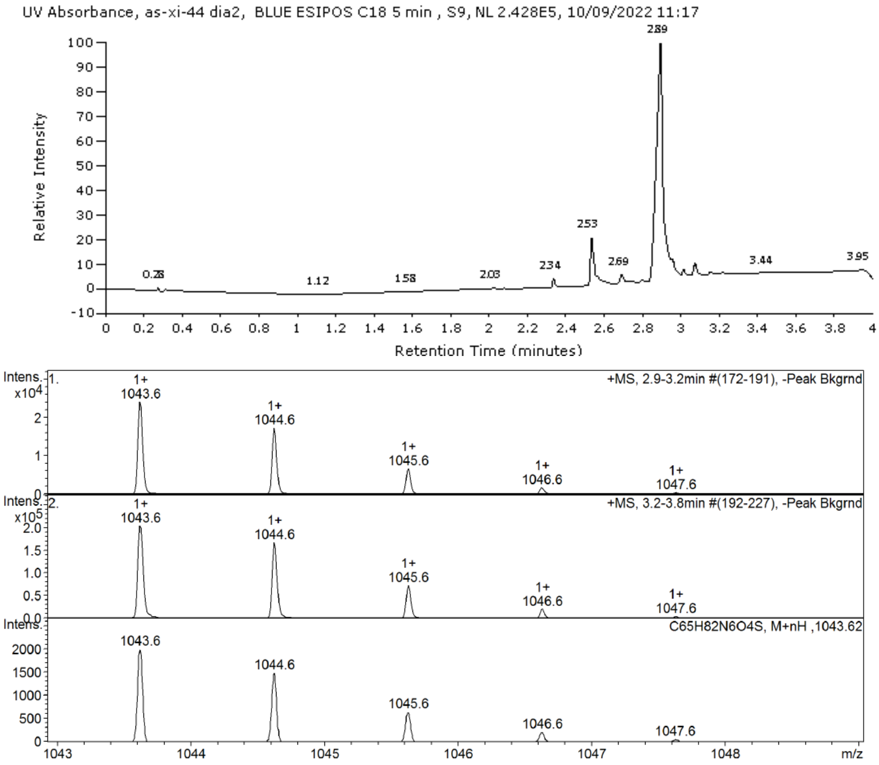

Figure S143. LC-MS (retention times: 2.53 mins, *m/z* = 1044.0 and 2.89 mins, *m/z* = 1044.0) and ESI-HR-MS of one the isolated isomer for **10** and experimental and calculated isotopic pattern.

Amide azide S17

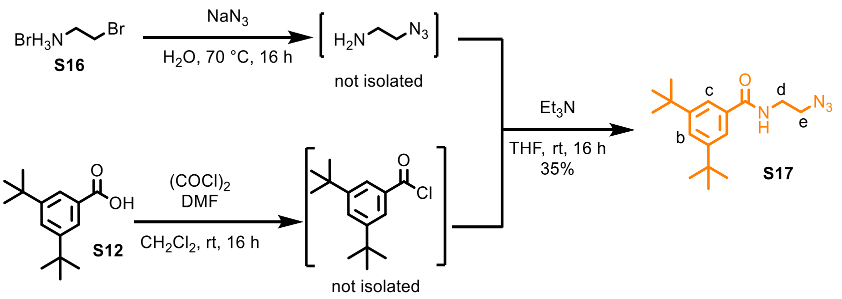

A solution of NaN_3 (476.2 mg, 7.3 mmol) and **S16** (500.0 mg, 2.4 mmol) in H_2O (5 mL) was heated at 70 °C for 16 h. The solution was cooled to 0 °C, then KOH (224 mg, 4.0 mmol) was added portion-wise and the resulting solution stirred at 0 °C for 15 min. The aqueous phases were extracted with Et_2O (3 x 5 mL), dried ($MgSO_4$) and filtered. The product, 2 azido-ethylamine, was stored as a solution of Et_2O at 0 °C. The yield was assumed to be quantitative.

In separate CEM vial was added **S12** (703 mg, 3.0 mmol), the vial sealed and flushed with N_2, then CH_2Cl_2 (6 mL), $(COCl)_2$ (0.77 mL, 9.0 mmol) and DMF (1 drop) were added. The solution was stirred at rt for 16 h, then the solvent and the excess $(COCl)_2$ were removed *in vacuo*. The residue was dissolved in THF (3 mL), the solution of 2-azidoethylamine prepared above and Et_3N (1.30 mL, 9.0 mmol) were added, and the solution was stirred at rt for 16 h, then cooled at 0 °C and H_2O added (5 mL). The phases were separated, and the aqueous phase was then extracted with EtOAc (3 x 20 mL). The combined organic extracts were washed with brine (20 mL), dried ($MgSO_4$) and concentrated *in vacuo*. Chromatography (petrol-EtOAc 0→50%) gave **S17** as a white foam (254.1 mg, 35%).

^{1}H NMR (400 MHz, $CDCl_3$) δ: 6.63-7.55 (m, 3H, H_b, H_c), 6.47 (t, *J* = 6.1, 1H, NH), 3.68-3.61 (m, 2H, H_d), 3.60-3.54 (m, 2H, H_e), 1.34 (s, 18H, H_a)

^{13}C NMR (101 MHz, $CDCl_3$) δ: 168.8, 151.4, 133.8, 125.9, 121.1, 51.1, 39.6, 35.0, 31.4.

HR-ESI-MS (+ve) *m/z* = 303.2190 $[M+H]^+$ (calc. 303.2180 *m/z* for $C_{17}H_{27}N_4O$);

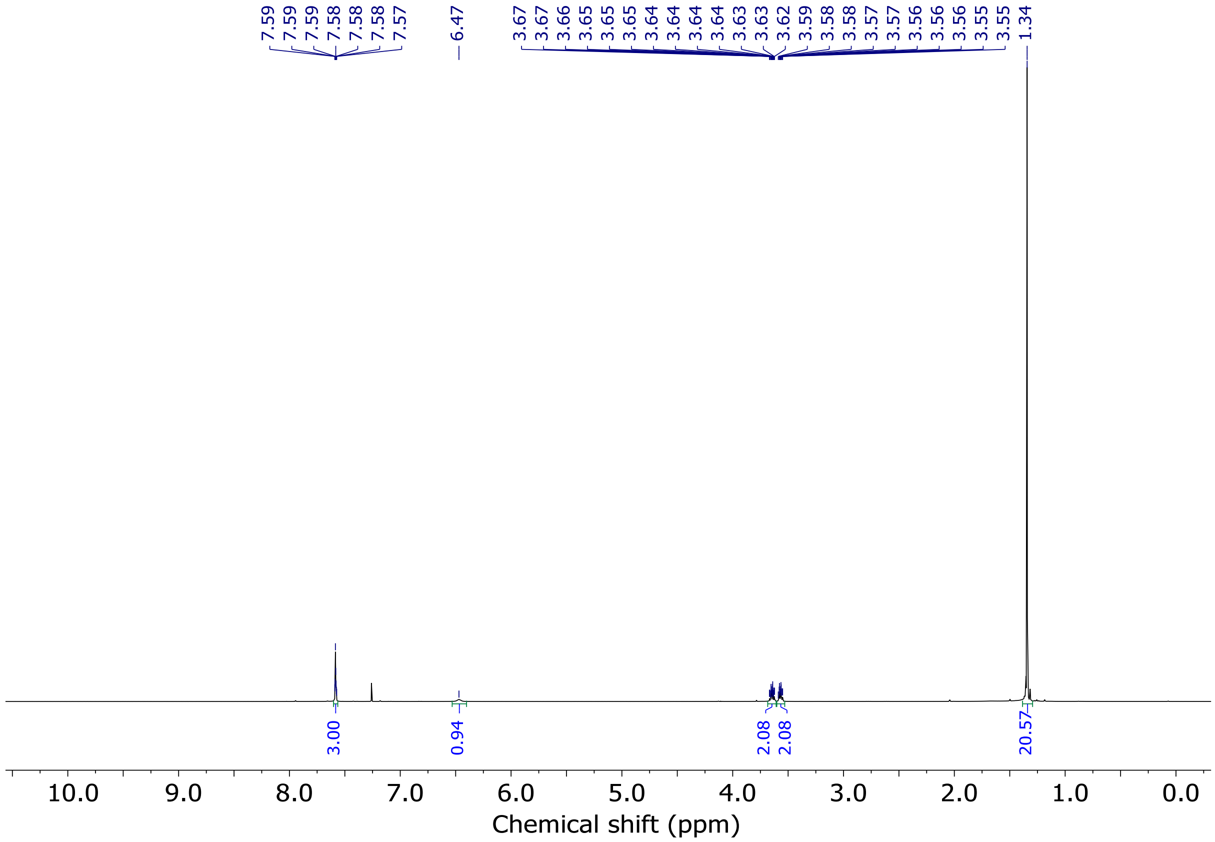

Figure S144: ^{1}H NMR ($CDCl_3$, 400 MHz) of **S17**.

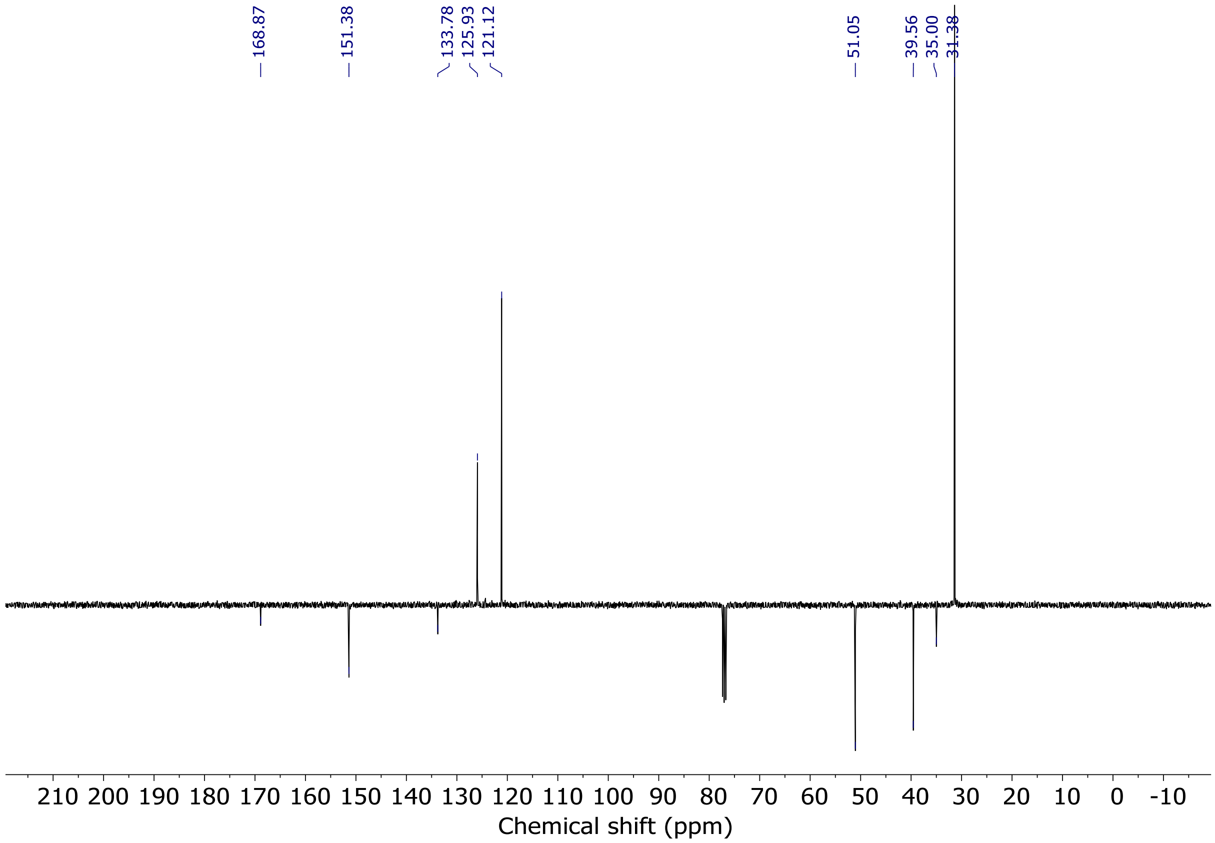

Figure S145: JMOD NMR ($CDCl_3$, 101 MHz) of **S17**.

Amide Rotaxanes (E_m)-11 and (Z_m)-11

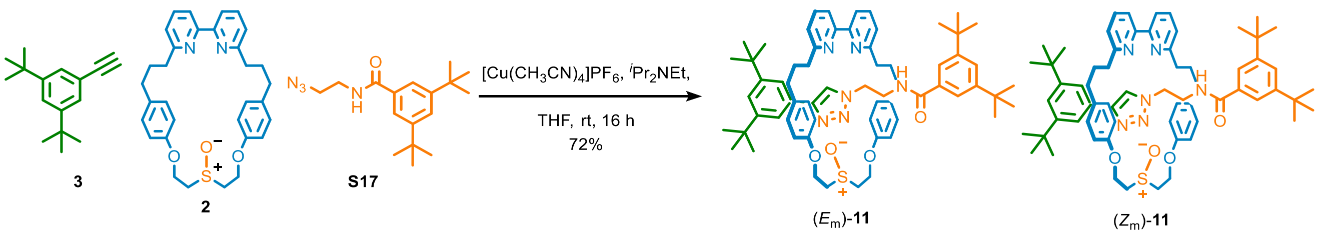

In a CEM vial were added **3** (8.9 mg, 41.7 µmol), **S17** (12.6 mg, 41.7 µmol), **2** (20.0 mg, 38.0 µmol) and [$Cu(CH_3CN)_4PF_6$] (13.6 mg, 36.5 µmol). The vial was sealed and purged with N_2, then THF was added (1.0 mL), followed by $^{i}Pr_2NEt$ (13.3 µL, 75.9 µmol). The solution was stirred at rt for 16 h. the solution was diluted with CH_2Cl_2 (5 mL), then EDTA-NH_3 (5 mL) was added. The solution was vigorously stirred until complete decolouration. The aqueous and organic phases were separated, and the aqueous phase was then extracted with CH_2Cl_2 (3 x 10 mL). The combined organic extracts were washed with brine (10 mL), dried ($MgSO_4$) and concentrated *in vacuo* to give a sample containing **11** as a mixture of diastereomers (72 : 28 *dr*, Figure S146). Chromatography (CH_2Cl_2-CH_3CN 0→100%) gave **11** as a colourless oil as (28.5 mg, 72%) as a mixture of diastereoisomers (2.4: 1 *dr*, Figure S147). An analytical sample of the major diastereoisomer was obtained *via* column chromatography (*n*-hexane-acetone 50→80%)

Major diastereoisomer (*E*$_m$)-11

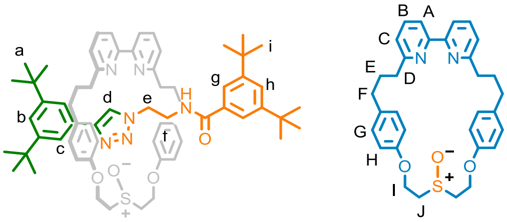

^{1}H NMR (500 MHz, CDCl$_3$) δ: 9.41 (t, *J* = 5.8, 1H, NH), 7.75 (s, 1H, H_d), 7.68 (d, *J* = 1.8, 2H, H_g), 7.66-7.60 (m, 2H, H_B), 7.53 (d, *J* = 1.9, 2H, H_c), 7.45-7.39 (m, 3H, H_A, H_h), 7.36 (t, *J* = 1.9, 1H, H_b), 7.11 (d, *J* = 7.7, 2H, H_C), 6.74-6.69 (m, 8H, H_G, H_H), 4.69-4.61 (m, 4H, H_I), 3.80 (dt, *J* = 13.0, 6.7, 2H, H_J), 3.52 (dd, *J* = 8.3, 6.6, 2H, H_e), 3.30 (dt, *J* = 13.0, 6.7, 2H, $H_{J'}$), 2.91 (dt, *J* = 8.0, 6.0, 2H, H_f), 2.75-2.49 (m, 2H, H_F), 2.50-2.39 (m, 4H, $H_{F'}$, H_D), 2.39-2.27 (m, 2H, $H_{D'}$), 1.91-1.80 (m, 2H, H_E), 1.80-1.67 (m, 2H, $H_{D'}$), 1.33 (s, 18H, H_a), 1.15 (s, 18H, H_i)

^{13}C NMR (126 MHz, CDCl$_3$) δ: 167.2, 163.0, 156.7, 155.4, 151.2, 150.1, 148.2, 137.2, 133.4, 133.3, 130.0, 129.5, 125.0, 122.2, 122.1, 122.0, 120.3, 120.0, 115.1, 60.7, 51.1, 48.9, 39.2, 36.5, 34.9, 34.9, 34.8, 31.5, 31.3, 31.3

LR-ESI-MS (+ve) *m/z* = 1043.6 [M+H]$^+$ for isotopic pattern see Figure S152.

Minor diastereoisomer (*Z*$_m$)-11

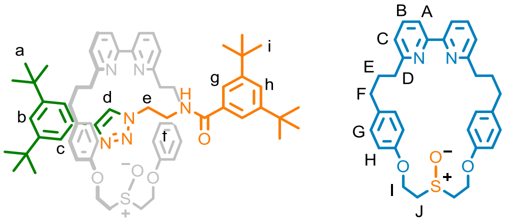

^{1}H NMR (500 MHz, CDCl$_3$) δ: 9.30 (t, *J* = 5.8, 1H, NH), 7.79 (s, 1H, H_d), 7.73 (d, *J* = 1.9, 2H, H_g), 7.66-7.60 (m, 4H, H_b, H_c, H_C), 7.45-7.39 (m, 5H, H_A, H_B, H_h), 6.78 (d, *J* = 8.6, 4H, H_G or H_H), 6.74-6.69 (m, 4H, H_G or H_H), 4.75 (ddd, *J* = 13.4, 10.5, 3.5, 2H, H_I), 4.69-4.61 (m, 2H, $H_{I'}$), 3.98 (ddd, *J* = 13.8, 10.5, 4.7, 2H, H_J), 3.45-3.39 (m, 2H, H_f), 3.14 (dt, *J* = 13.5, 3.6, 2H, $H_{J'}$), 2.75-2.49 (m, 6H, H_e, H_F), 2.50-2.39 (m, 4H, $H_{F'}$, H_D), 2.39-2.27 (m, 2H, $H_{D'}$), 1.91-1.80 (m, 2H, H_E), 1.80-1.67 (m, 2H, $H_{D'}$), 1.40 (s, 18H, H_i), 1.12 (s, 18H, H_a)

^{13}C NMR (126 MHz, CDCl$_3$) δ: 167.5, 162.9, 156.8, 155.5, 151.2, 150.2, 148.2, 137.1, 134.0, 133.6, 130.1, 129.7, 124.9, 121.9, 121.8, 120.3, 120.3, 120.2, 114.6, 60.0, 52.2, 48.3, 39.1, 36.3, 35.0, 34.8, 34.8, 31.5, 31.2, 31.0

HR-ESI-MS (+ve) *m/z* = 1043.6 [M+H]$^+$ for isotopic pattern see Figure S152.

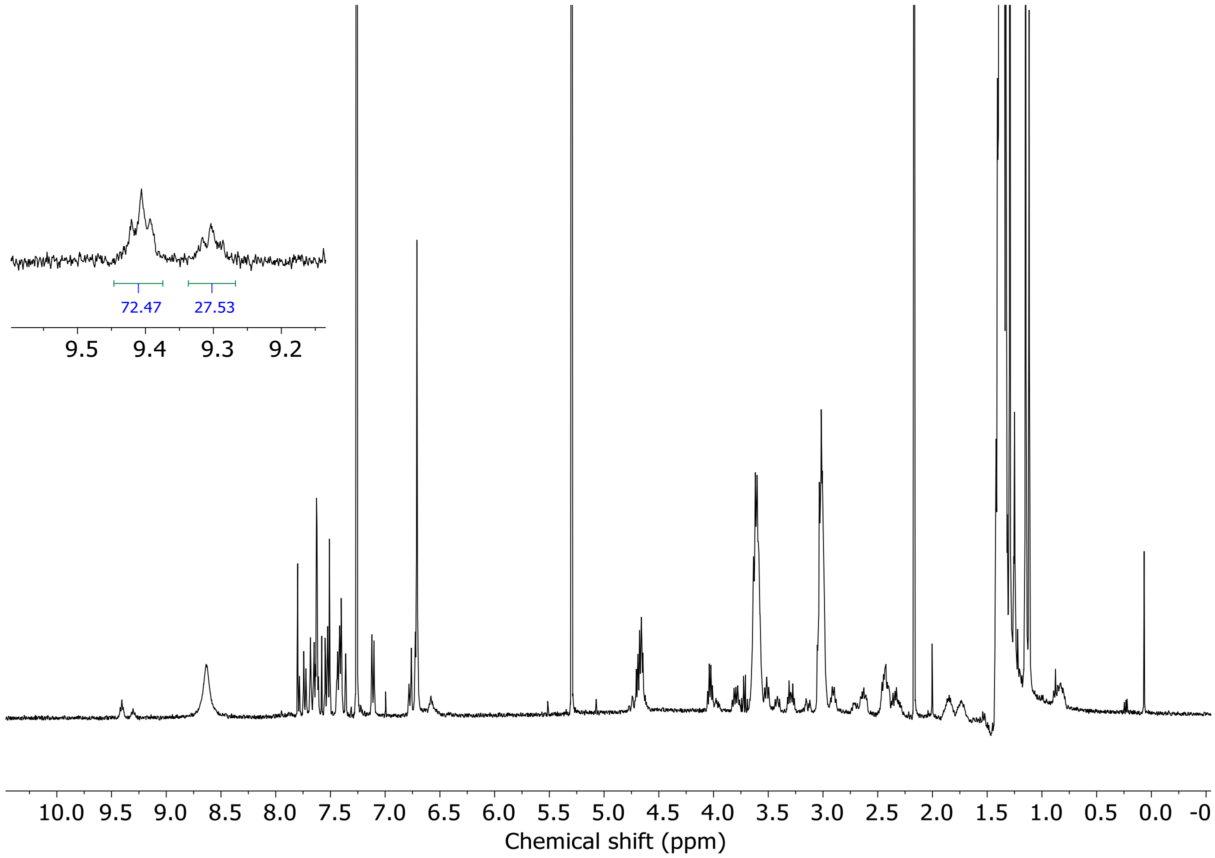

Figure S146: ^{1}H NMR ($CDCl_3$, 400 MHz) of (E_m)-**11** and (Z_m)-**11** prior to chromatography (72 : 28 *dr*).

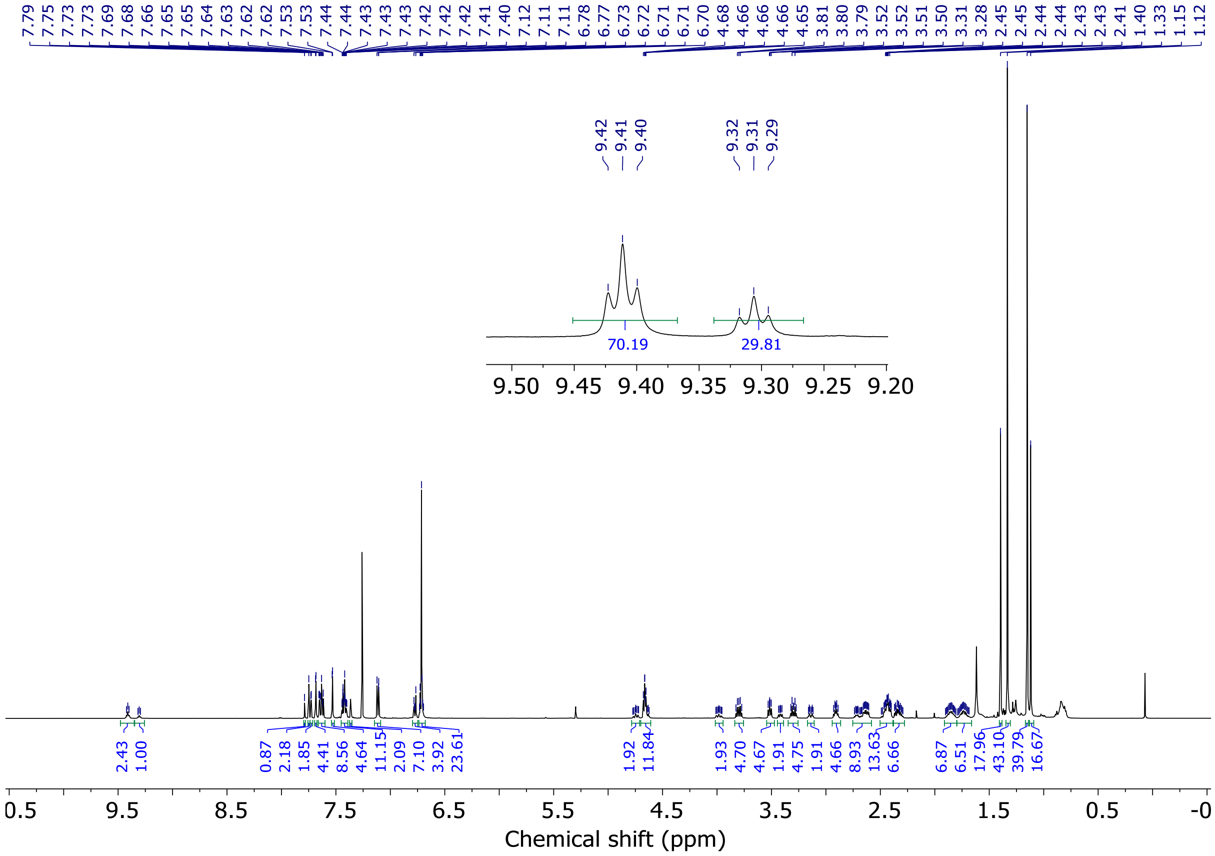

Figure S147: ^{1}H NMR ($CDCl_3$, 400 MHz) of (E_m)-**11** and (Z_m)-**11** (2.4 : 1 *dr*).

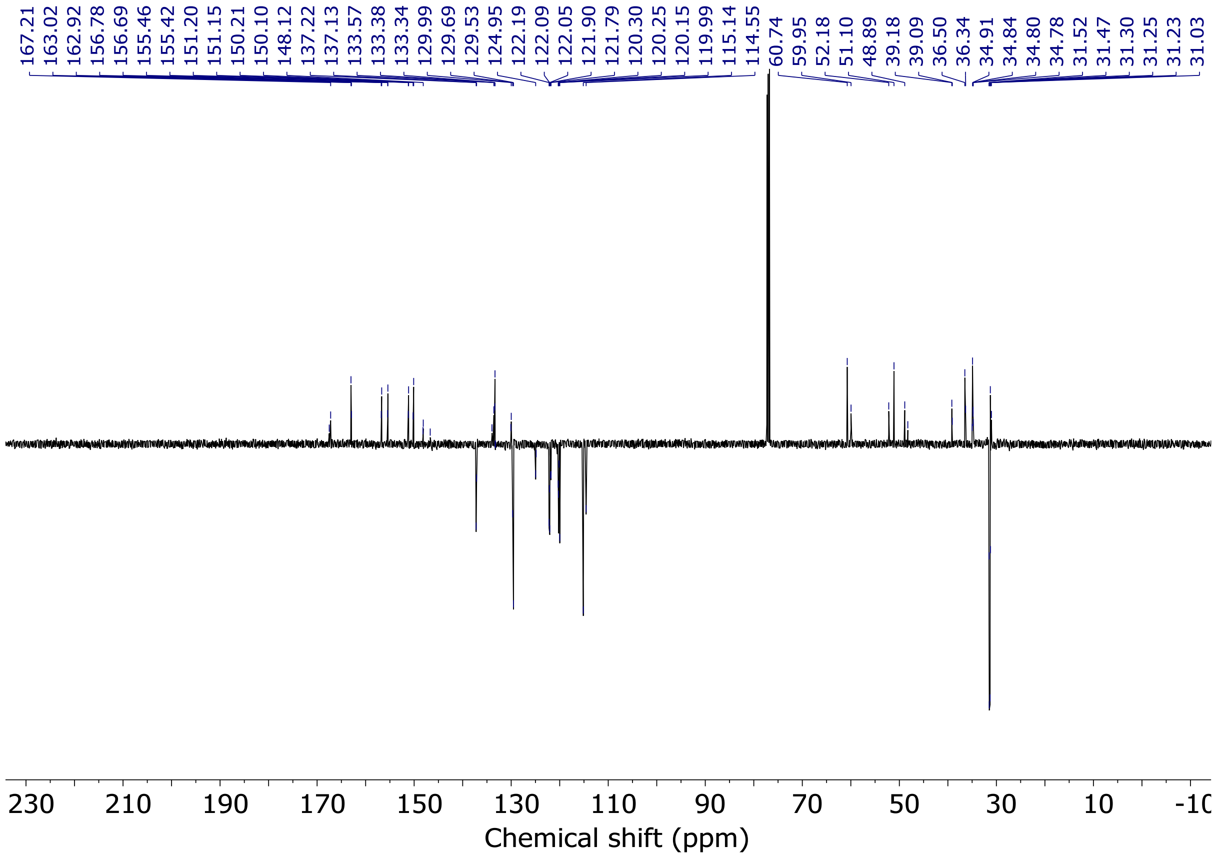

Figure S148: JMOD NMR ($CDCl_3$, 101 MHz) of (E_m)-**11** and (Z_m)-**11** (2.4 : 1 *dr*).

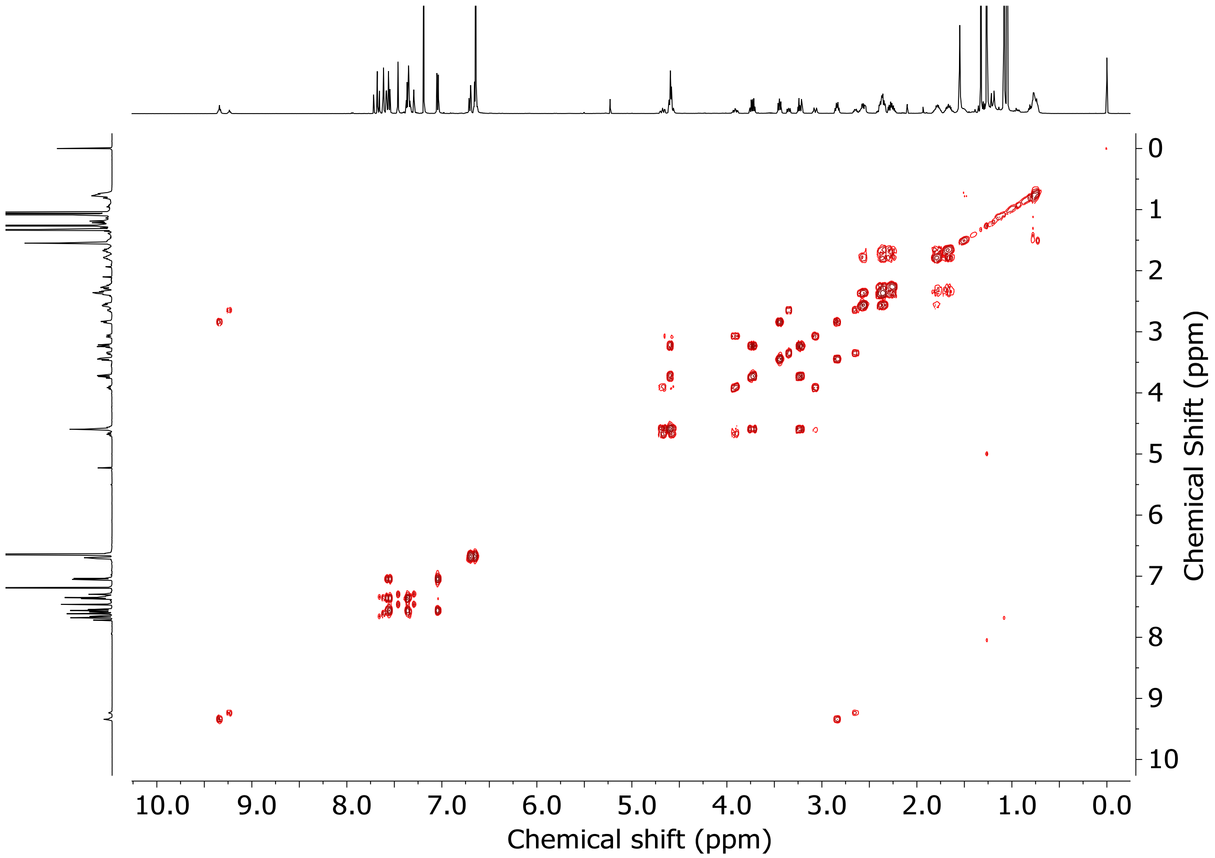

Figure S149: COSY NMR ($CDCl_3$) of (E_m)-**11** and (Z_m)-**11** (2.4 : 1 *dr*).

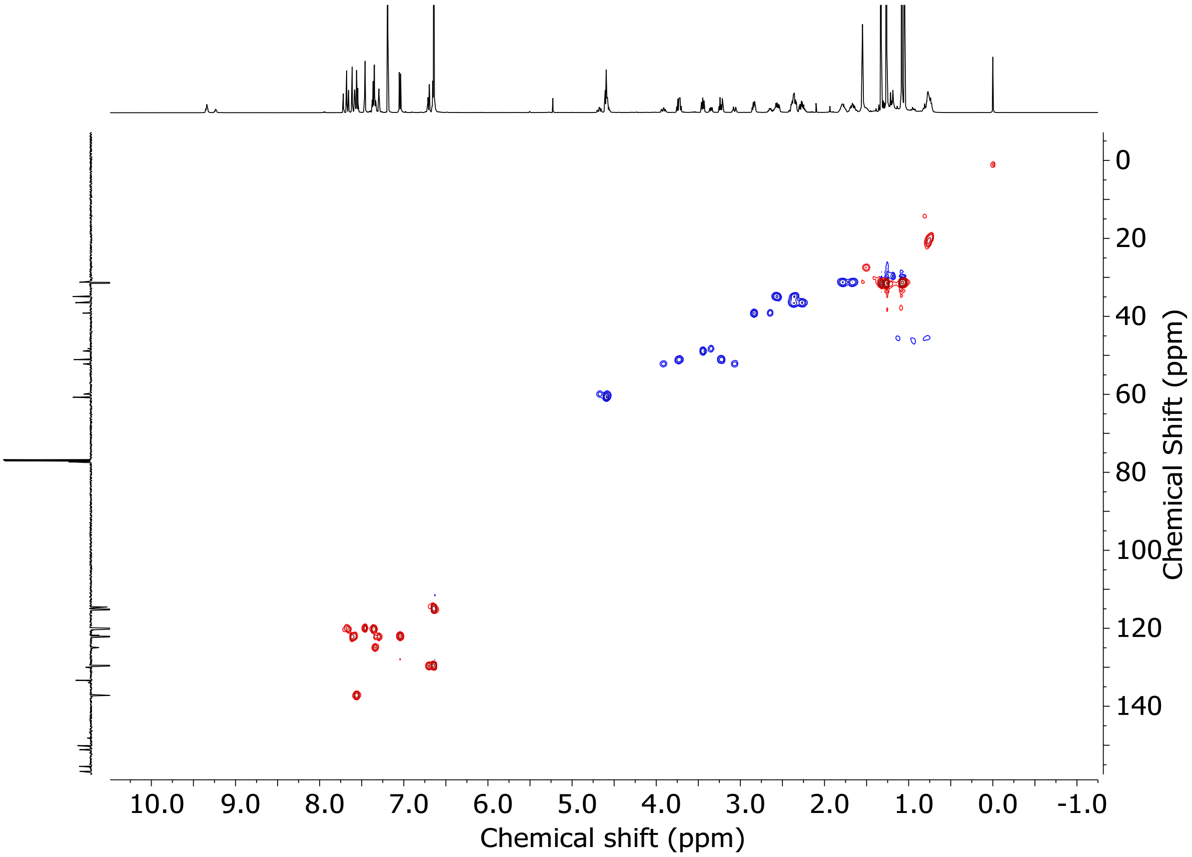

Figure S150: HSQC NMR ($CDCl_3$) of (E_m)-**11** and (Z_m)-**11** (2.4 : 1 *dr*).

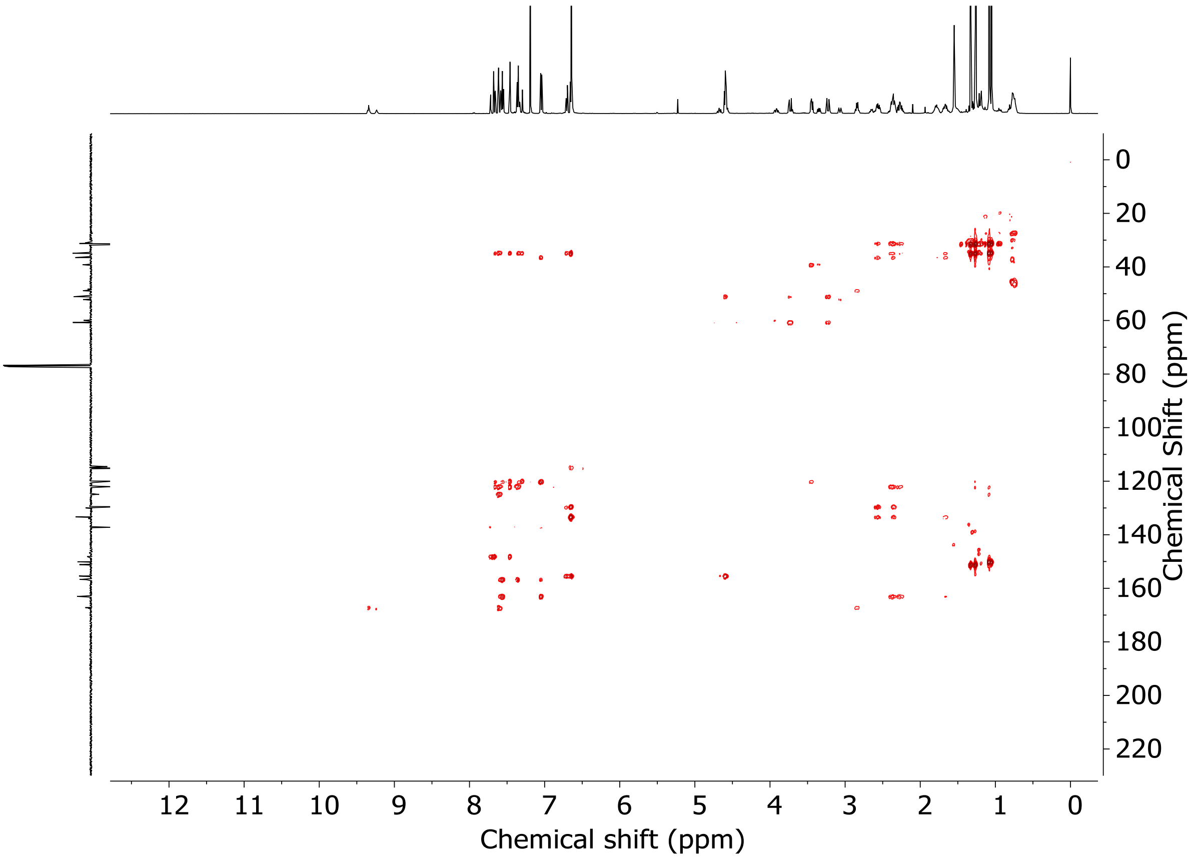

Figure S151: HMBC NMR ($CDCl_3$) of (E_m)-**11** and (Z_m)-**11** (2.4 : 1 *dr*).

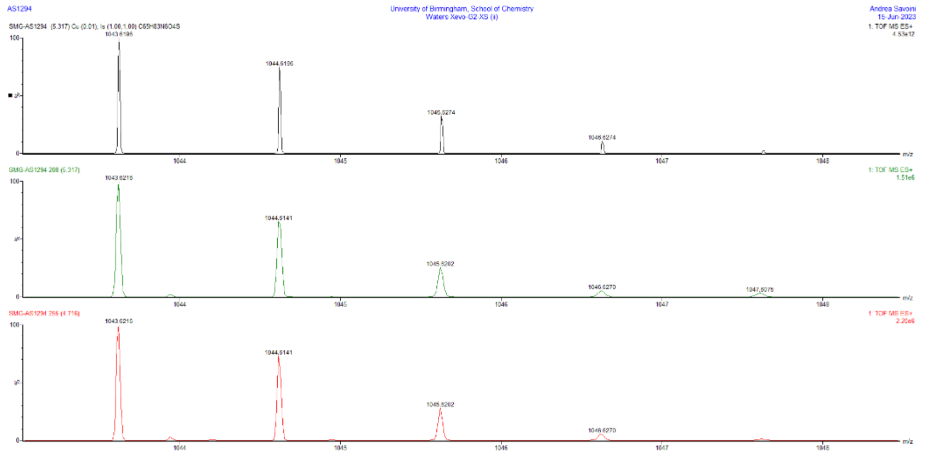

Figure S152: Calculated (top) and observed (middle, bottom) isotopic patterns for (E_m)-**11** and (Z_m)-**11.**

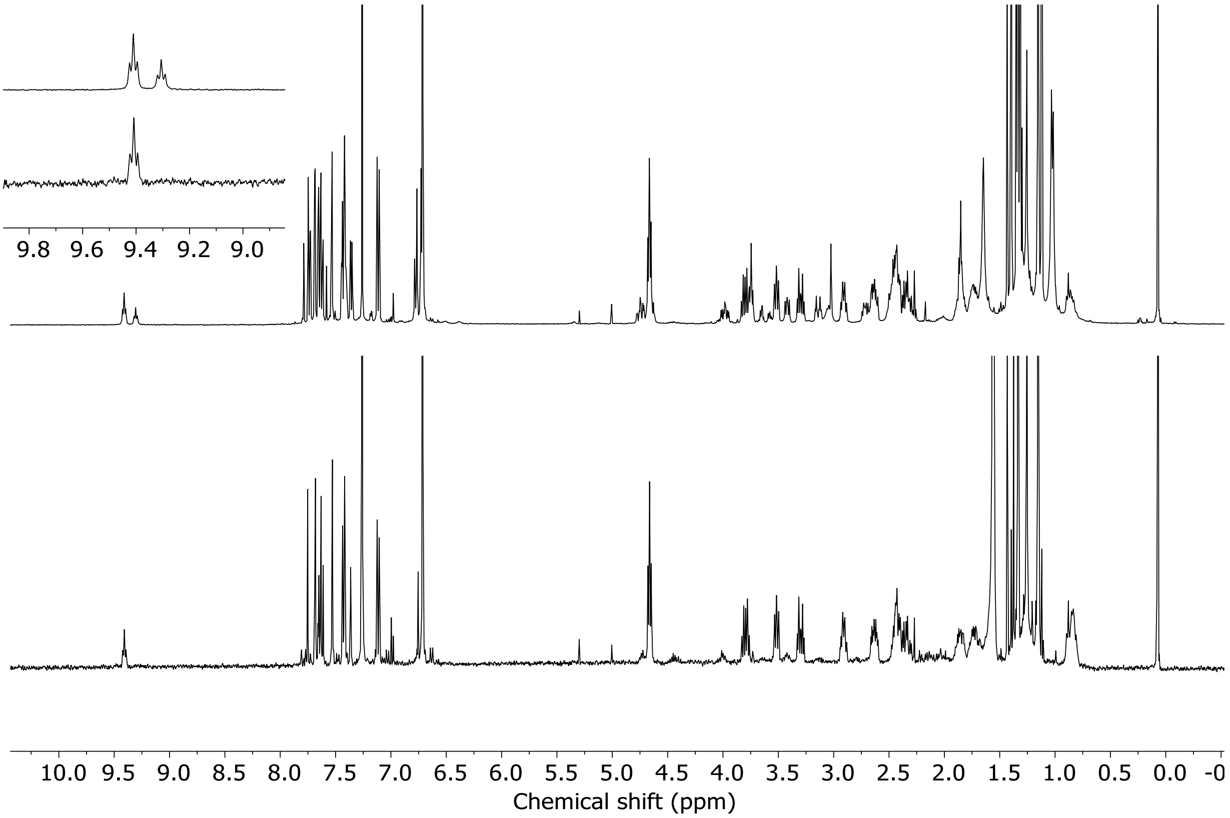

Figure 153: ^{1}H NMR ($CDCl_3$, 400 MHz) of (E_m)-**11** and (Z_m)-**11** (top), analytical sample of (*E_m*)-**11** obtained after second round of chromatography for SC-XRD (bottom, apodised with standard Gaussian function, 1.00 GB [Hz])

Aniline azide S19

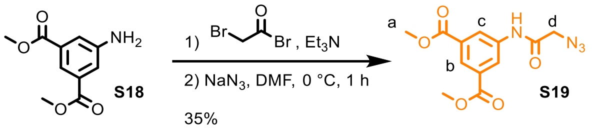

To a solution of **S18** (509.1 mg, 2.4 mmol) in DMF (5 mL) at 0 °C is added Et_3N (0.34 mL, 2.4 mmol) bromoacetyl bromide (0.19 mL, 2.2 mmol) dropwise. The solution turns red and in is stirred at 0 °C for 30 minutes. NaN_3 (238.2 mg, 3.7 mmol) is then added portion wise at 0 °C. The solution is allowed to warm at rt, then it was stirred for 1h. The reaction mixture was then quenched by adding H_2O (10 mL) dropwise. The aqueous and organic phases were separated, and the aqueous phase was then extracted with Et_2O (3 x 20 mL). The combined organic extracts were washed with brine (10 mL), dried ($MgSO_4$) and concentrated *in vacuo*. Chromatography (petrol-Et_2O 0→50%) gave **S19** as a white powder (247.5 mg, 35%).

^{1}H NMR (400 MHz, $CDCl_3$) δ: 8.48 (t, *J* = 1.5, 1H, H*b*), 8.41 (d, *J* = 1.5, 2H, H*c*), 8.21 (bs, 1H, NH), 4.20 (s, 2H, H*d*), 3.95 (s, 6H, H*a*).

^{13}C NMR (101 MHz, $CDCl_3$) δ: 165.8 (HMBC), 164.7 (HMBC), 137.3, 131.6, 127.1, 125.0, 52.9, 52.6.

HR-ESI-MS (+ve) *m/z* = 315.0704 $[M+Na]^+$ (calc. *m/z* for $C_{12}H_{12}NaN_4O_5$ 315.0705).

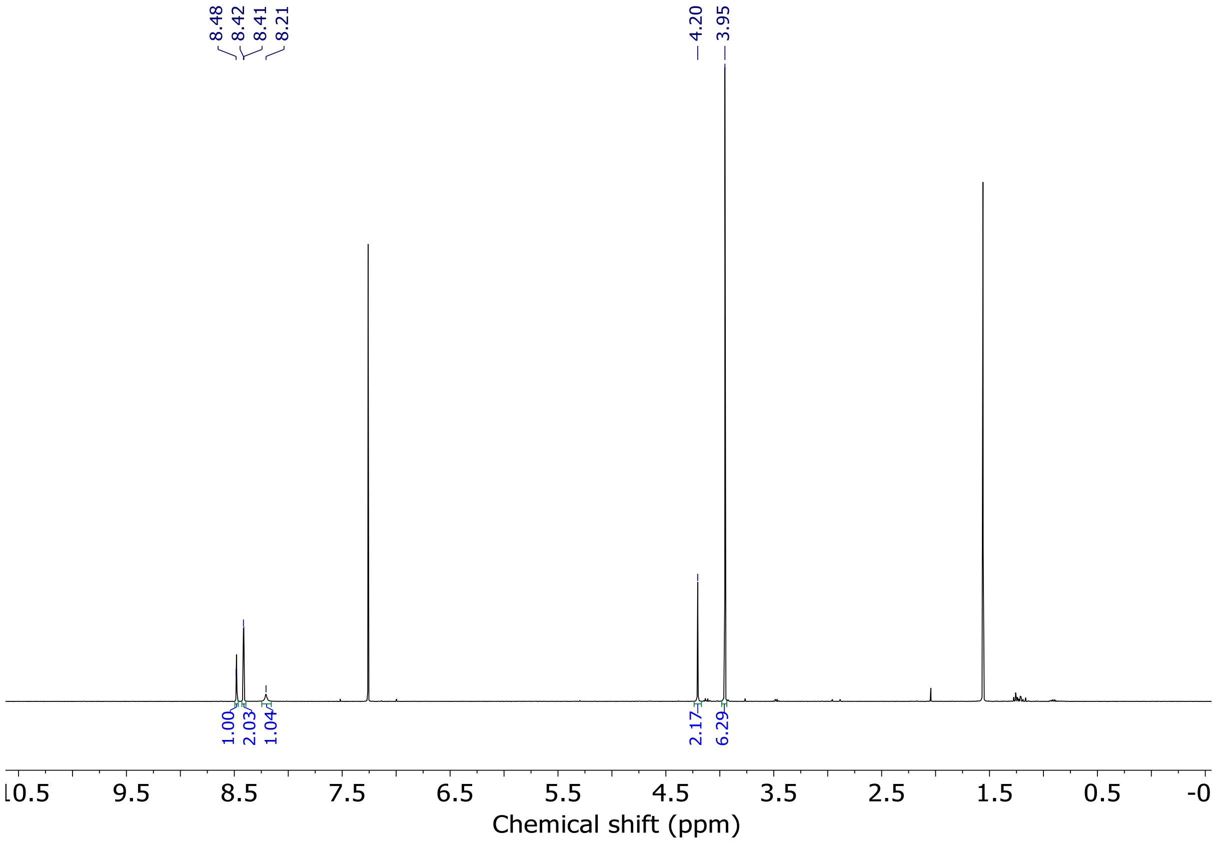

Figure S154: ^{1}H NMR ($CDCl_3$, 400 MHz) of **S19**.

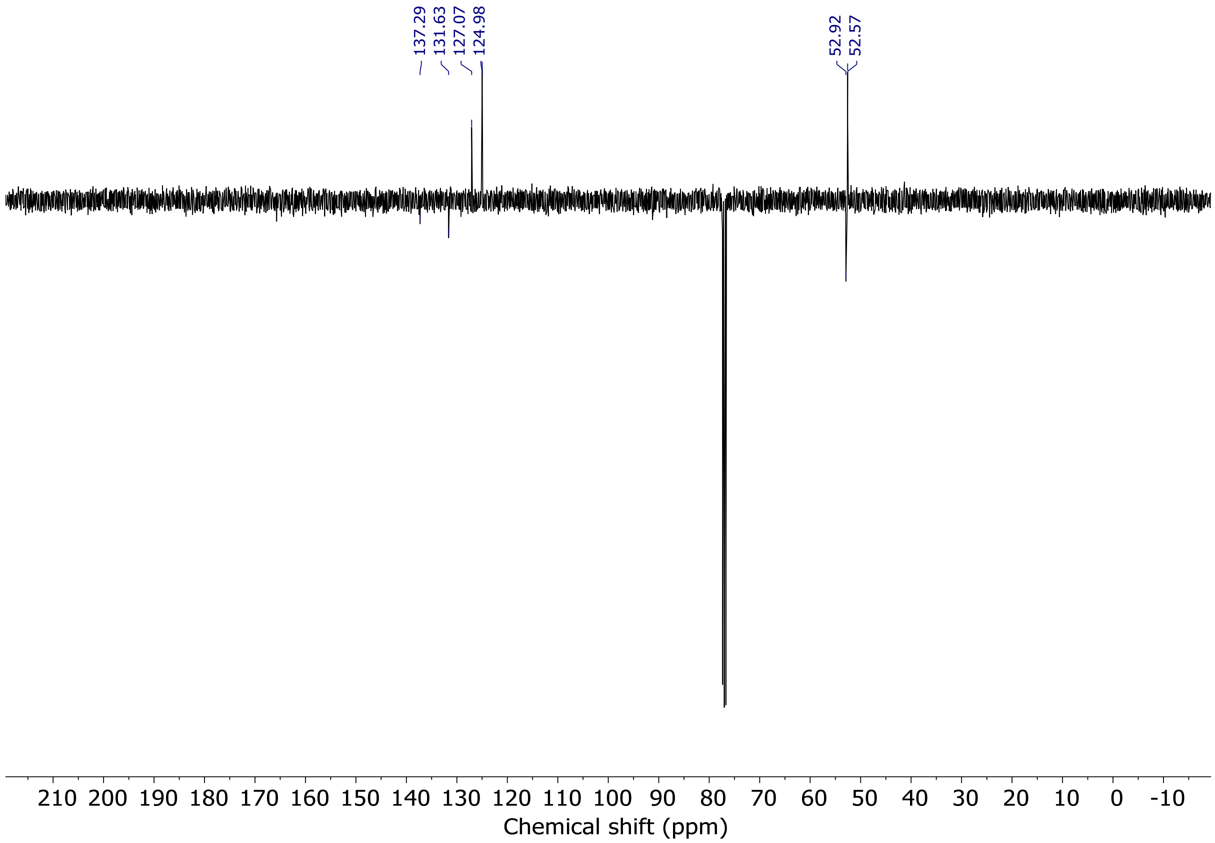

Figure S155: JMOD NMR ($CDCl_3$, 101 MHz) of **S19**.

Aniline Rotaxanes (E_m)-12 and (Z_m)-12

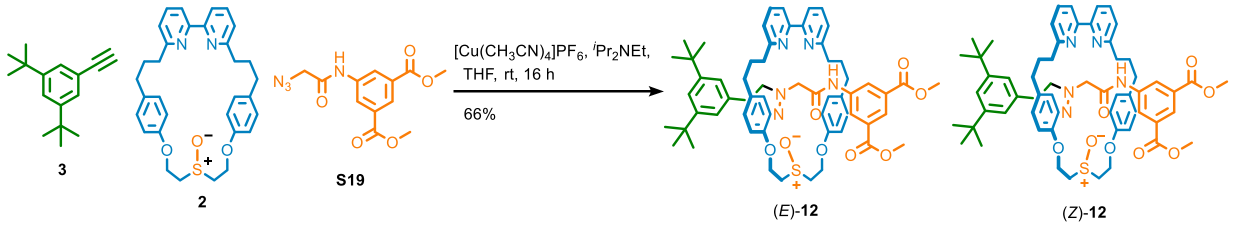

In a CEM vial were added the **3** (2.1 mg, 10.3 µmol), **S19** (3.0 mg, 10.3 µmol), **2** (5.0 mg, 9.4 µmol) and [$Cu(CH_3CN)_4PF_6$] (3.4 mg, 9.1 µmol). The vial was sealed and purged with N_2, then THF was added (1.0 mL), followed by ^{i}Pr$_2$NEt (3.3 µL, 19.0 µmol). The solution was stirred at rt for 16 h. the solution was diluted with CH_2Cl_2 (2 mL), then EDTA-NH_3 (5 mL) was added. The solution was vigorously stirred until complete decolouration. The aqueous and organic phases were separated, and the aqueous phase was then extracted with CH_2Cl_2 (3 x 10 mL). The combined organic extracts were washed with brine (10 mL), dried ($MgSO_4$) and concentrated *in vacuo* to give a sample containing **12** as a mixture of diastereomers (13 : 87 *dr*, Figure S156). Chromatography (CH_2Cl_2-CH_3CN 0→100%) gave **12** as a colourless oil (6.4 mg, 66%) as a mixture of diastereoisomers (1 : 6.1 *dr*, Figure S157).

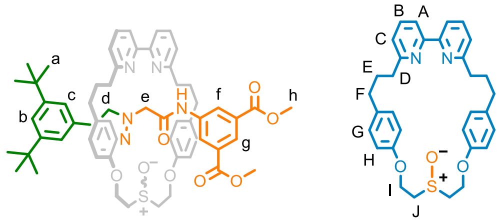

Major diastereoisomer

^{1}H NMR (500 MHz, CDCl$_3$) δ: 11.45 (s, 1H, H_d), 8.40 (t, J = 1.5, 1H, H_g), 8.13 (d, J = 1.6, 2H, H_f) 7.82-7,75 (m, 2H, H_B)7.70 (d, J = 1.9, 2H, H_c), 7.65 (d, J = 7.9, 2H, H_A), 7.46 (t, J = 1.8, 1H, H_b), 7.38 (s, 1H, NH), 7.29-7.23 (m, 2H, H_C superimposed with CDCl$_3$), 6.68 (dt, J = 8.5, 2.5, 4H, H_G), 6.61 (dt, J = 8.5, 2.0, 4H, H_H), 4.65 (s, 2H, H_e), 4.49 (ddd, J = 13.0, 8.8, 4.5, 2H, H_I), 4.32 (dt, J =12.6, 5.1, 2H, $H_{I'}$), 3.96 (s, 6H, H_h), 3.20 (ddd, J = 13.8, 8.8, 5.3, 2H, H_J), 2.75 (dt, J = 13.5, 4.6, 2H, $H_{J'}$), 2.68-2.41 (m, 8H, H_D, H_F), 1.92-1.72 (m, 4H, H_E), 1.42 (s, 18H, H_a)

^{13}C NMR (126 MHz, CDCl$_3$) δ: 166.1, 163.1, 162.5, 156.2, 155.4, 151.6, 148.4, 138.9, 137.9, 133.2, 130.8, 129.7, 129.3, 125.7, 124.4, 122.6, 122.5, 121.1, 120.3, 119.9, 115.4, 60.4, 52.5, 51.2, 36.6, 35.0, 34.8, 31.6, 31.0.

HR-ESI-MS (+ve) *m/z* = 1033.5 $[M+H]^+$ for isotopic pattern see Figure S162.

Minor diastereoisomer

^{1}H NMR (500 MHz, CDCl$_3$) δ: 11.62 (s, 1H, H_d), 8.37 (t, J = 1.5, 1H, H_g), 8.12 (d, J = 1.5, 2H, H_f), 7.82-7,75 (m, 4H, H_B, H_c), 7.64 (d, J = 8.0, 2H, H_A), 7.46 (t, J = 1.8, 1H, H_b), 7.43 (s, 1H, NH), 7.29-7.23 (m, 2H, H_C superimposed with CDCl$_3$) 6.64 (s, 8H, H_G, H_H), 4.70-4.62 (m, 2H, H_I), 4.59 (s, 2H, H_e), 4.45 (ddd, J = 13.0, 8.8, 4.5, 2H, $H_{I'}$) 3.92 (s, 6H, H_h), 3.40 (ddd, J = 14.1, 8.6, 3.9, 2H, H_J), 3.07 (ddd, J = 14.2, 5.5, 3.4, 2H, $H_{J'}$), 2.68-2.41 (m, 8H, H_D, H_F), 1.92-1.72 (m, 4H, H_E), 1.45 (s, 18H, H_a)

^{13}C NMR (126 MHz, CDCl$_3$) δ: 166.0, 163.2, 162.5, 156.2, 155.3, 151.5, 148.6, 139.0, 137.8, 132.9, 130.6, 129.8, 129.4, 125.5, 124.6, 122.6, 121.1, 120.3, 120.2, 114.7, 58.9, 52.4, 52.0, 49.6, 36.7, 34.9, 31.6, 31.4

HR-ESI-MS (+ve) *m/z* = 1033.5 $[M+H]^+$ for isotopic pattern see Figure S162.

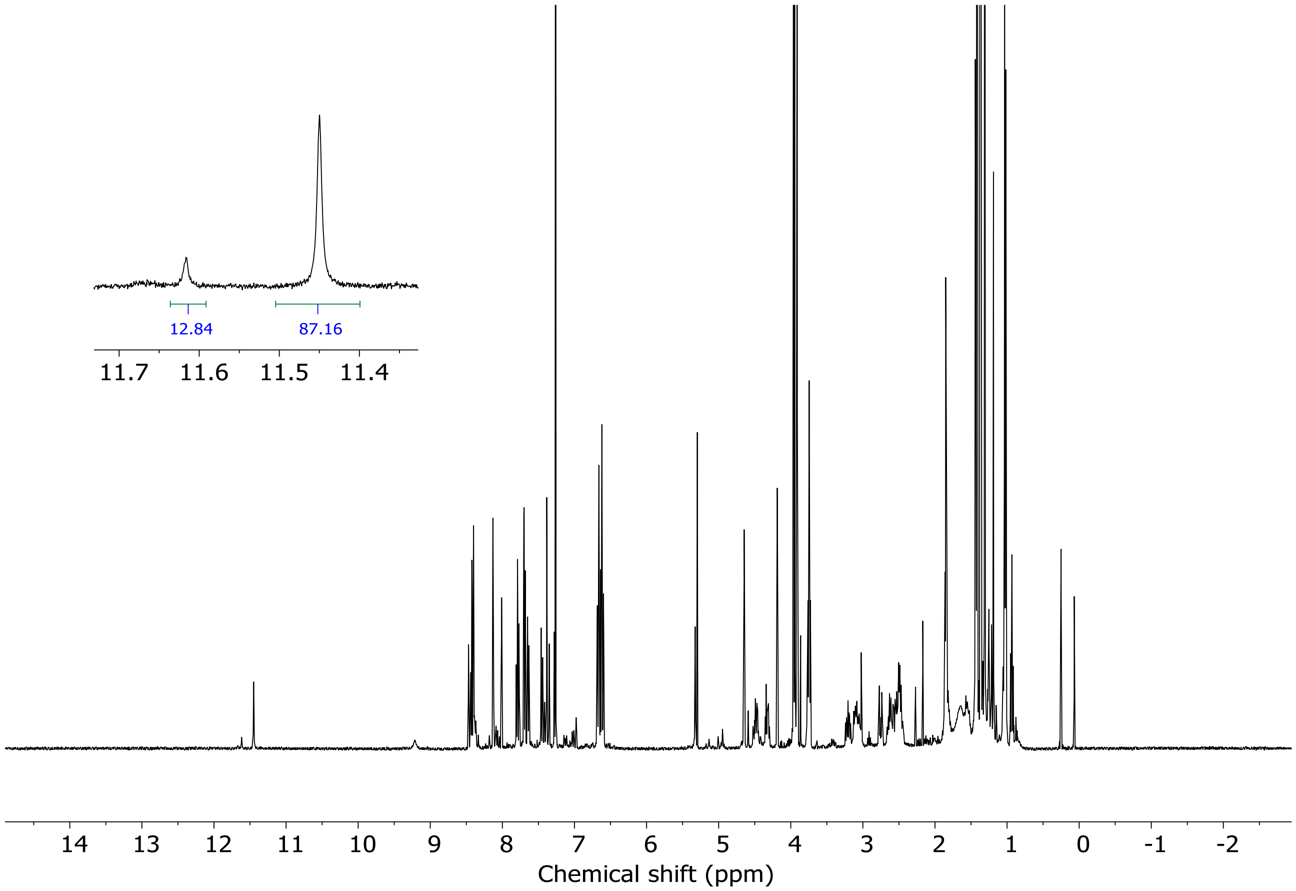

Figure S156 ¹H NMR ($CDCl_3$, 400 MHz) of (E_m)-**12** and (Z_m)-**12** prior to chromatography (13 : 87 *dr*).

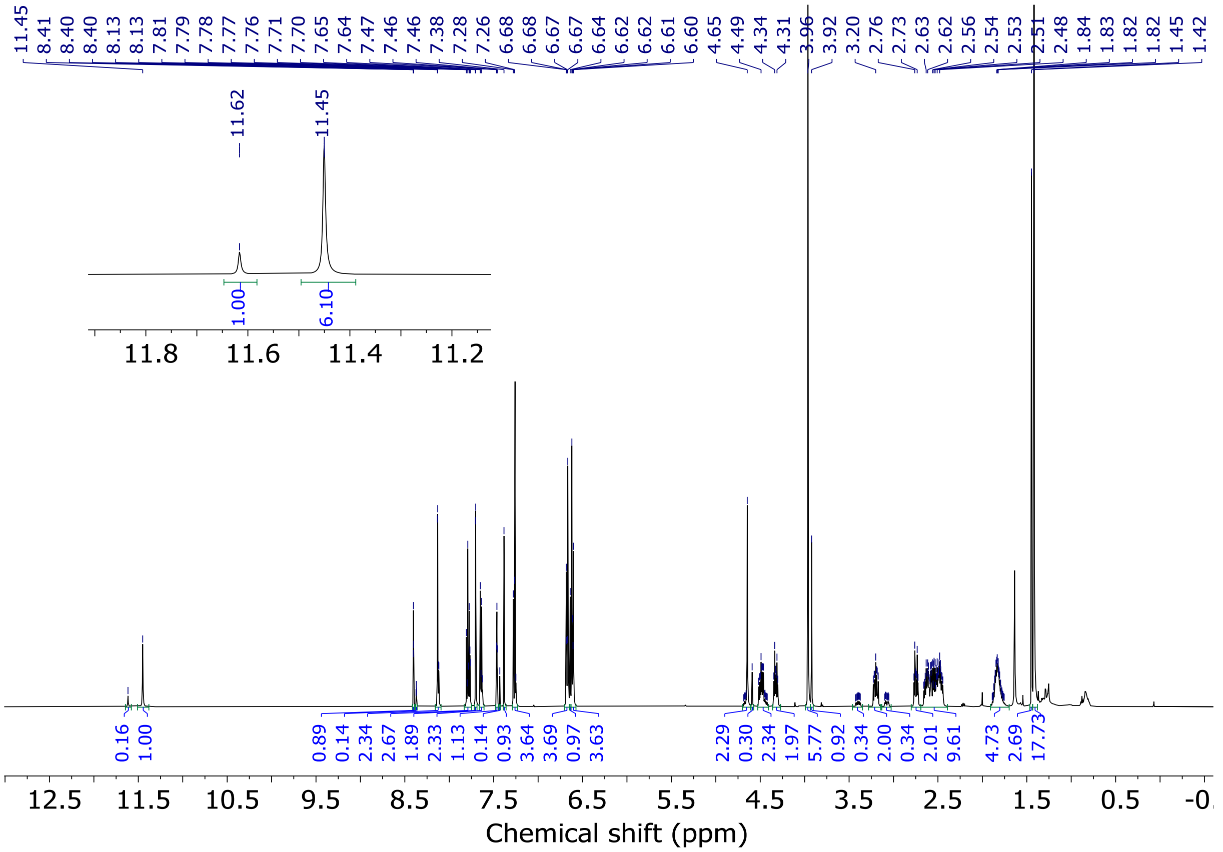

Figure S157: ¹H NMR ($CDCl_3$, 400 MHz) of (E_m)-**12** and (Z_m)-**12** (1 : 6.1 *dr*).

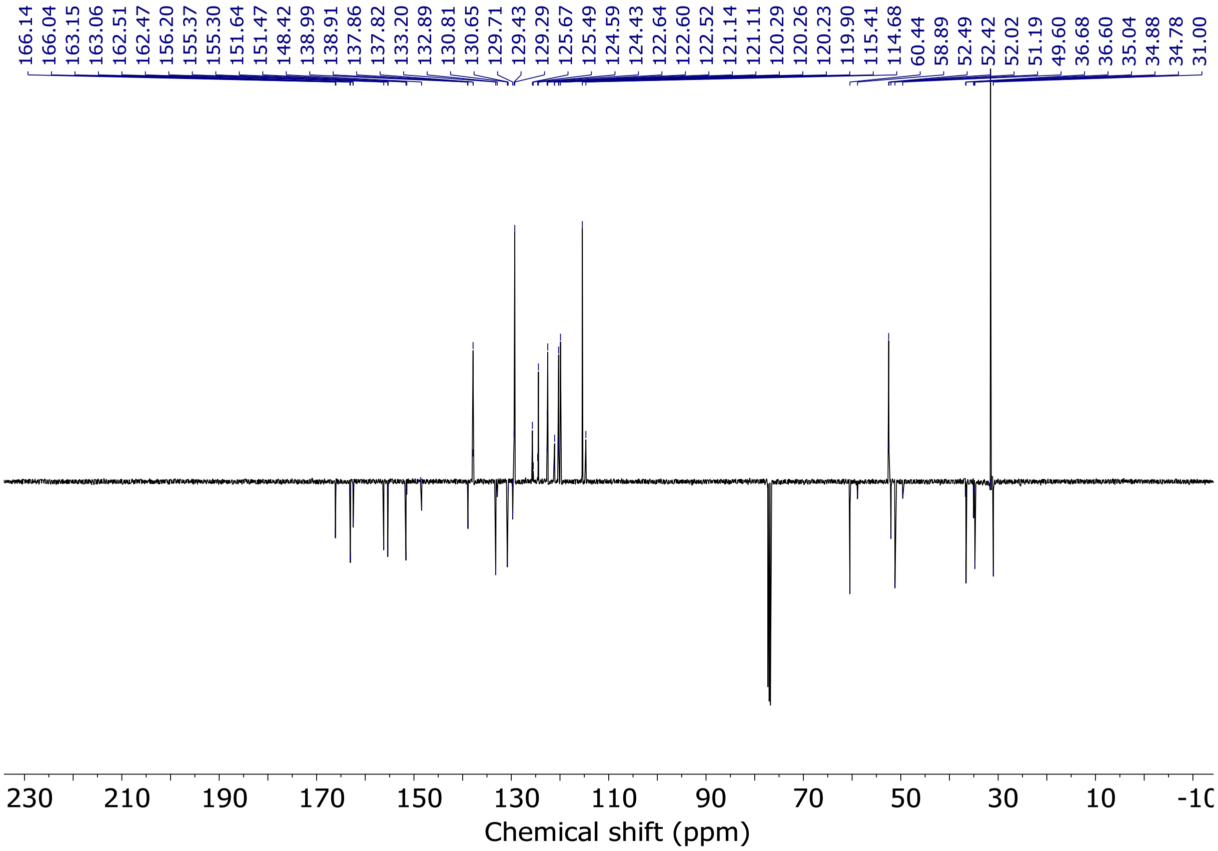

Figure S158: JMOD NMR ($CDCl_3$, 101 MHz) of (E_m)-**12** and (Z_m)-**12** (1 : 6.1 *dr*).

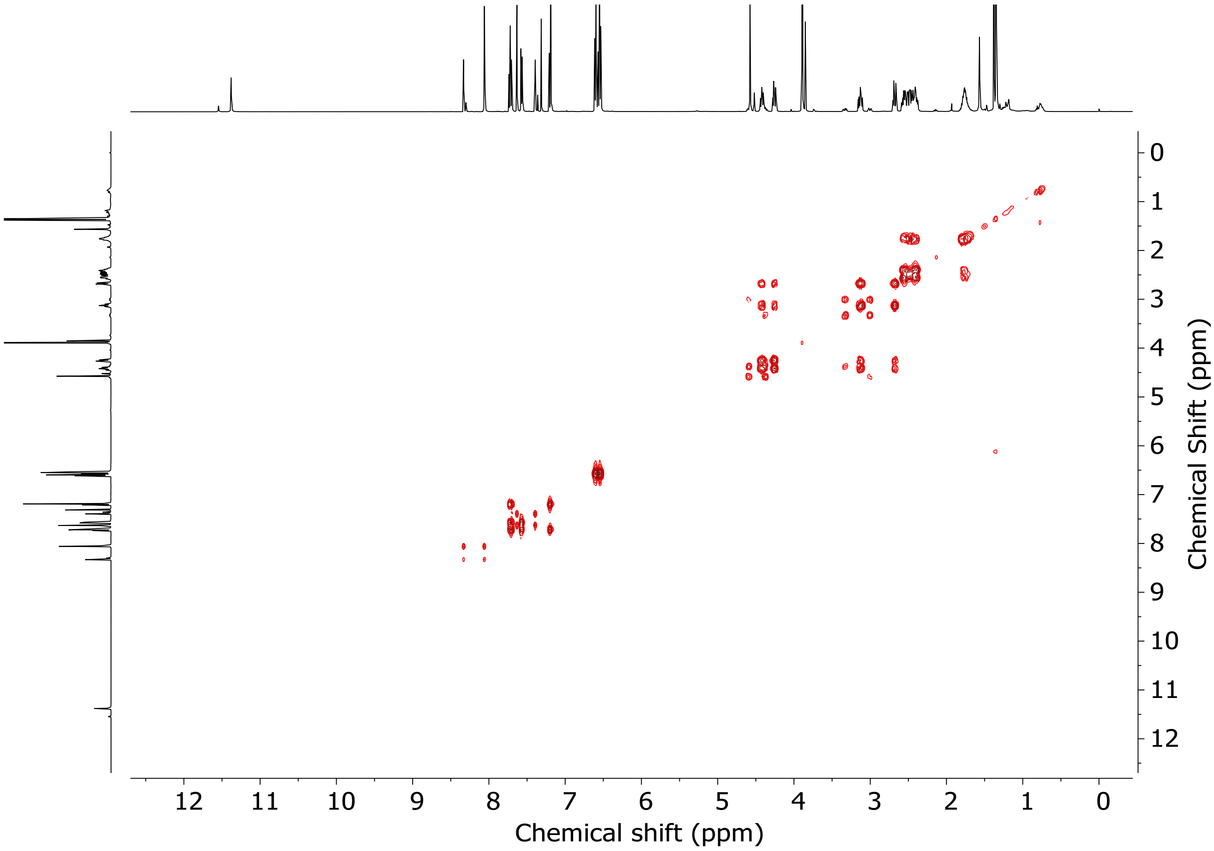

Figure S159: COSY NMR ($CDCl_3$) of (E_m)-**12** and (Z_m)-**12** (1 : 6.1 *dr*).

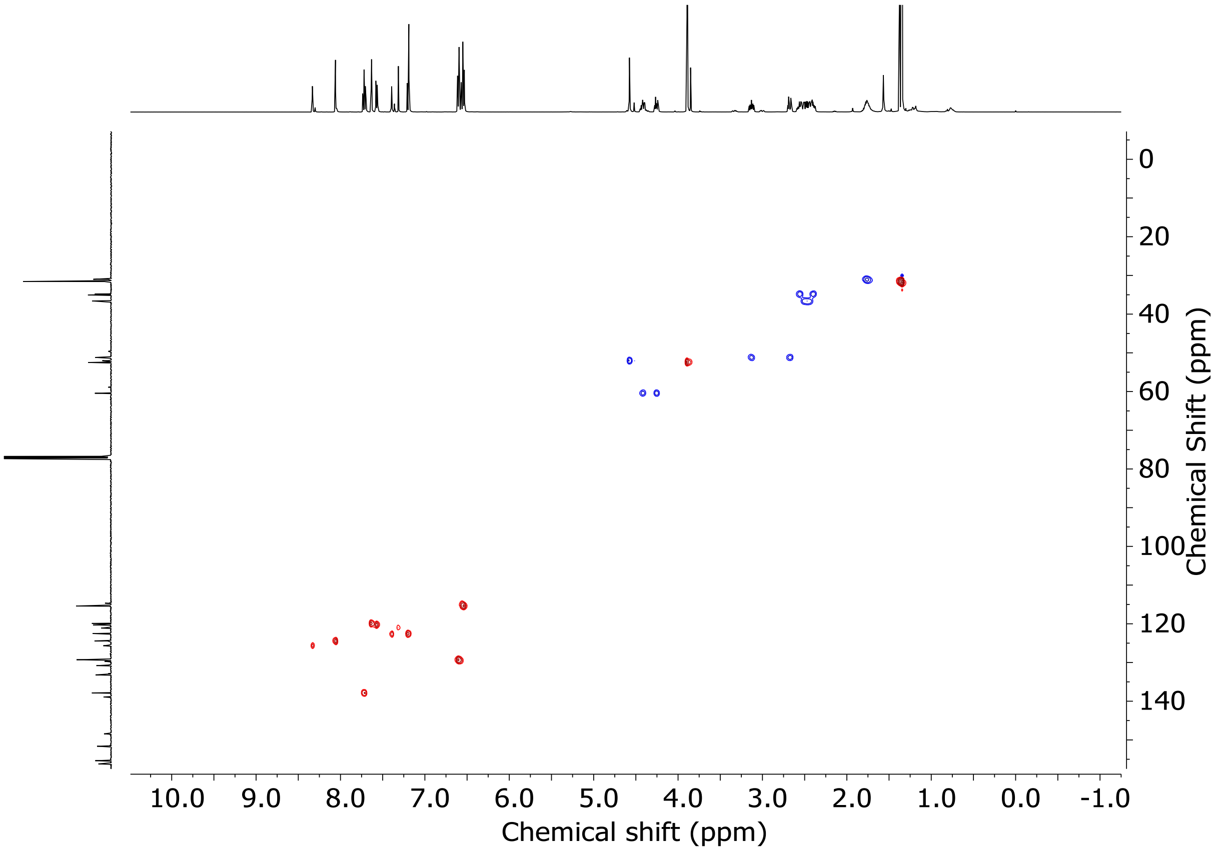

Figure S160: HSQC NMR ($CDCl_3$) of (E_m)-**12** and (Z_m)-**12** (1 : 6.1 *dr*).

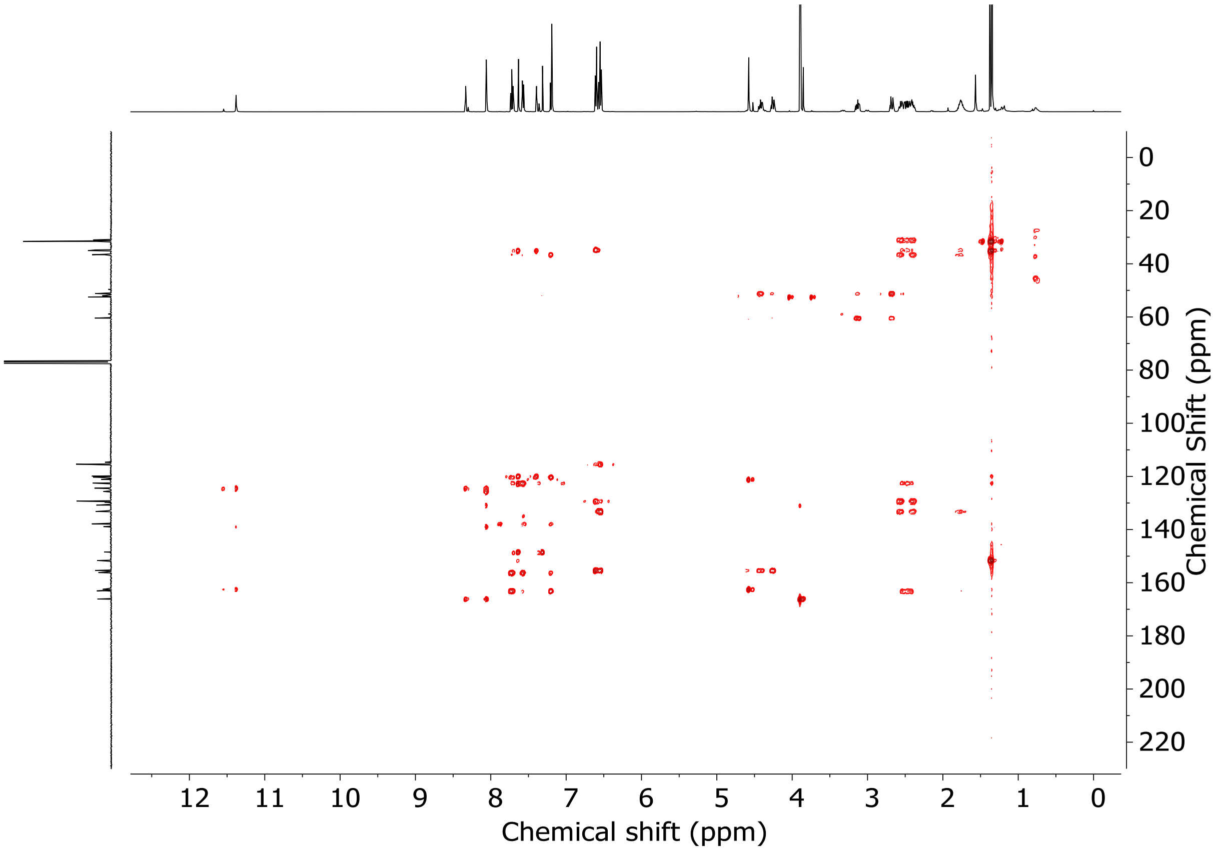

Figure S161: HMBC NMR ($CDCl_3$) of (E_m)-**12** and (Z_m)-**12** (1 : 6.1 *dr*).

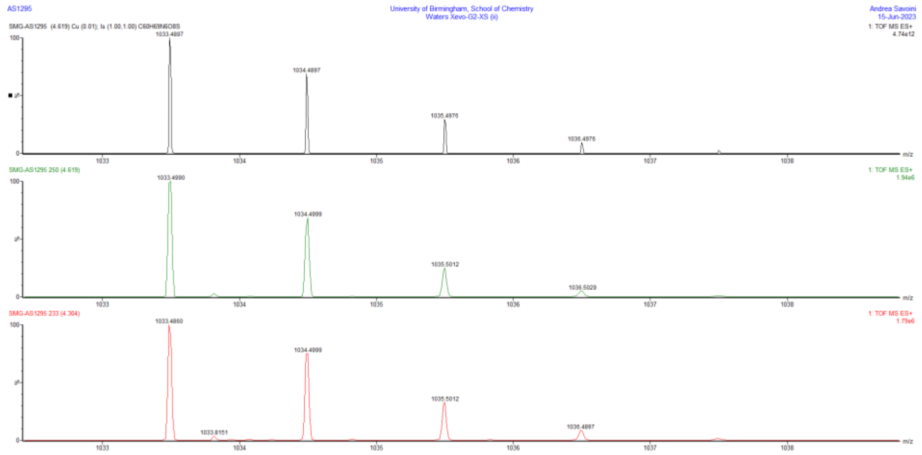

Figure S162: Calculated (top) and observed (middle, bottom) isotopic patterns for (E_m)-**12** and (Z_m)-**12**.

5. Synthesis of catenane 14 and associated compounds (Scheme 4, main text)

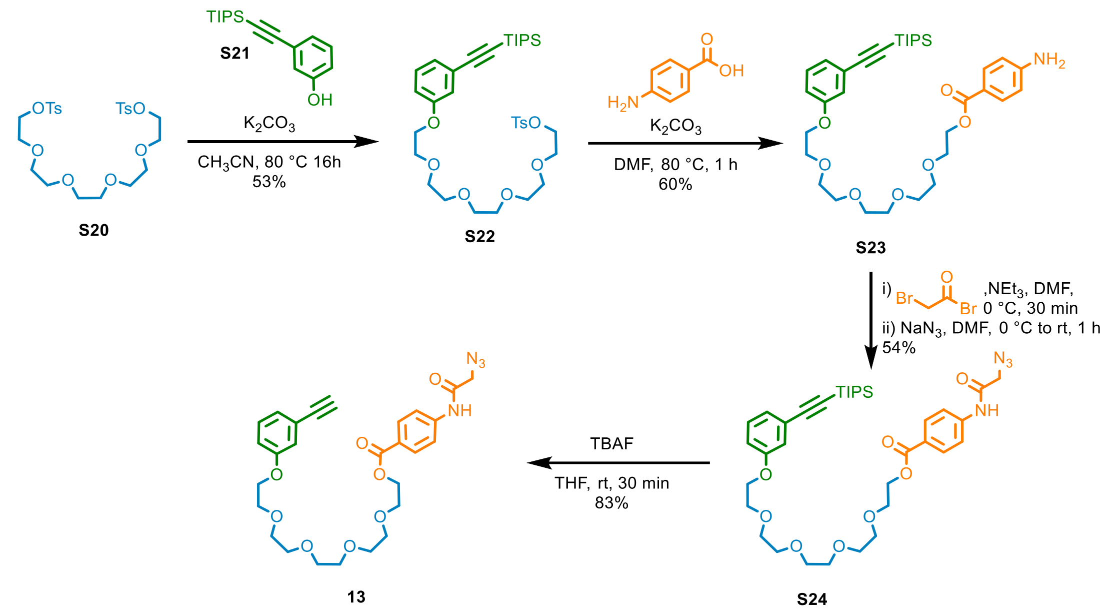

Scheme S2: Synthetic route to macrocycle precursor **13**.

TIPS aryl ether S22

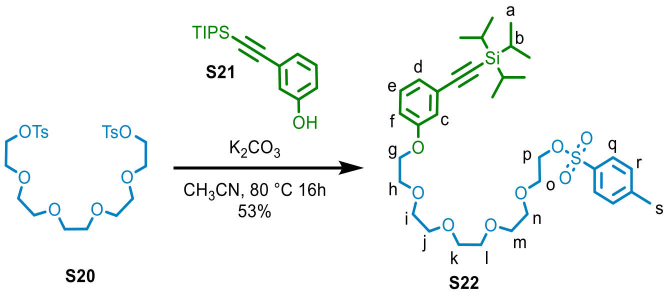

S21 (1.5 g, 4.6 mmol), **S20** (5.0 g, 9.2 mmol) and K_2CO_3 (2.6 g, 18.3 mmol) were suspended in CH_3CN (250 mL) and the resulting suspension was heated at reflux for 16 h. H_2O (100 mL) was added, then the aqueous and organic phases were separated, and the aqueous phase was then extracted with EtOAc (3 x 100 mL). The combined organic extracts were washed with brine (150 mL), dried ($MgSO_4$) and concentrated in vacuo. Chromatography (petrol : EtOAc 0→60%) gave **S22** as a colourless oil (1.6 g, 53%).

^{1}H NMR (400 MHz, CDCl$_3$) *δ*: 7.79 (dt, *J* = 8.3, 1.9, 2H, H_q), 7.39-7.29 (m, 2H, H_r), 7.19 (t, *J* = 7.8, 1H, H_e), 7.07 (dt, *J* = 7.6, 1.2, 1H, H_f), 7.00 (dd, *J* = 2.6, 1.4, 1H, H_c), 6.87 (ddd, *J* = 8.4, 2.7, 1.0, 1H, H_d), 4.21-4.06 (m, 4H, H_g, H_p), 3.87-3.80 (m, 2H, H_h), 3.74-3.49 (m, 14H, H_i, H_j, H_k, H_l, H_m, H_n, H_o), 2.44 (s, 3H, H_s), 1.12 (app.s, 21H, H_a, H_b)

^{13}C NMR (101 MHz, CDCl$_3$) *δ*: 173.9, 158.4, 144.8, 129.8, 129.3, 128.0, 124.8, 124.5 117.7, 115.4, 106.9, 90.4, 70.9, 70.8 (×3), 70.7, 70.5, 69.7, 69.2, 68.7, 67.5, 29.7, 21.6, 18.7, 11.3.

HR-ESI-MS (+ve) *m/z* = 649.3242 [M+H]$^+$ (calc. 648.3225 *m/z* for $C_{34}H_{53}O_8SSi$);

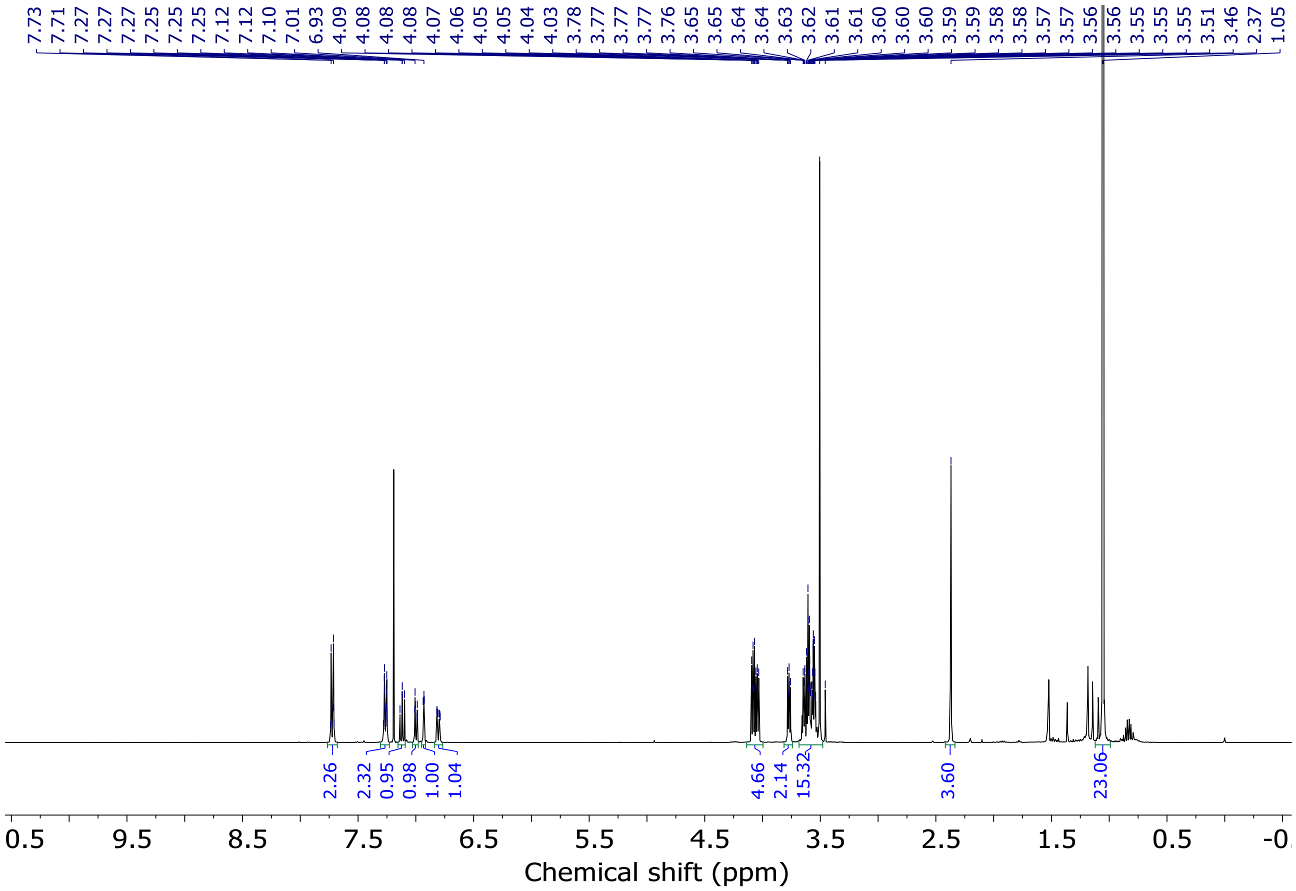

Figure S163: ^{1}H NMR ($CDCl_3$, 400 MHz) of **S22**.

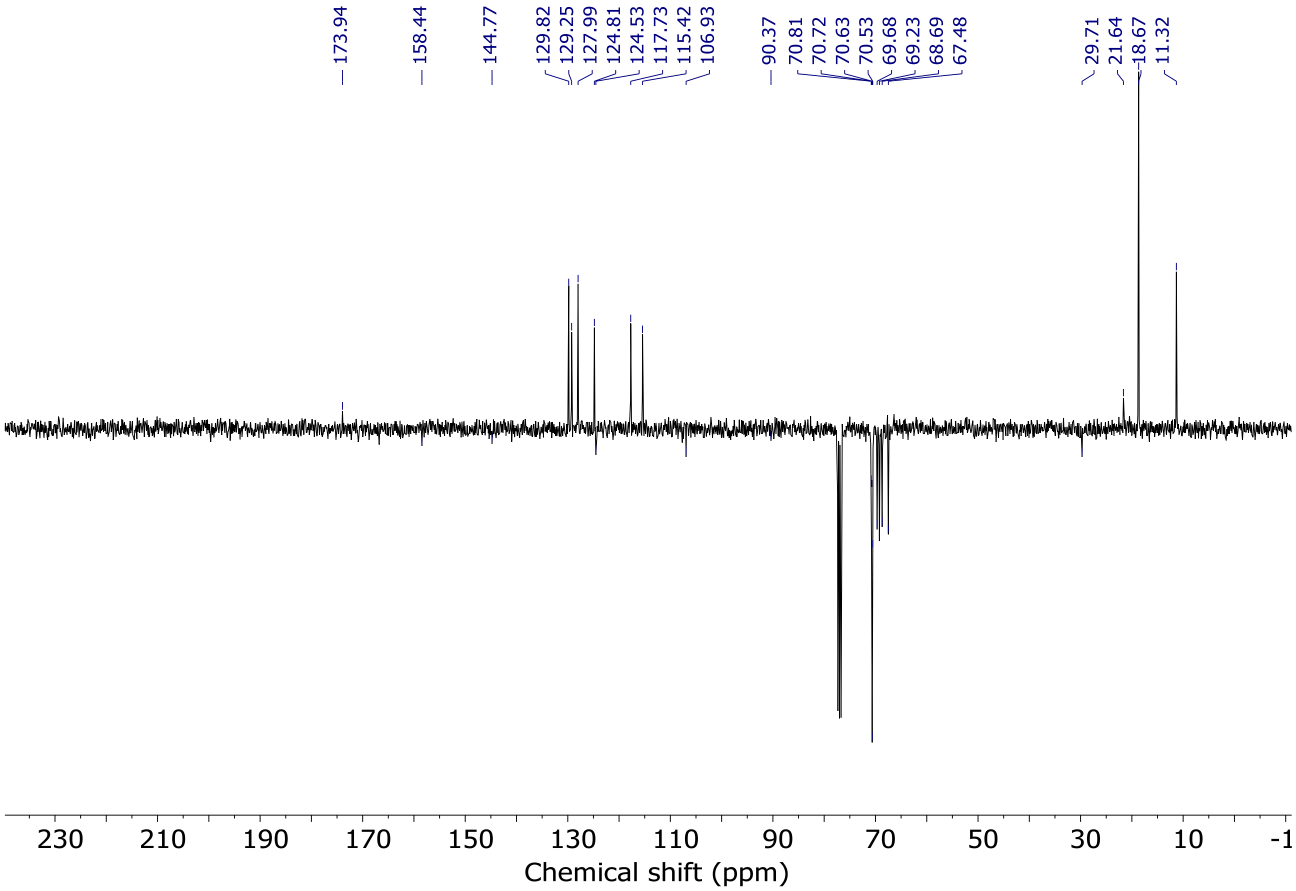

Figure S164: JMOD NMR ($CDCl_3$, 101 MHz) of **S22**.

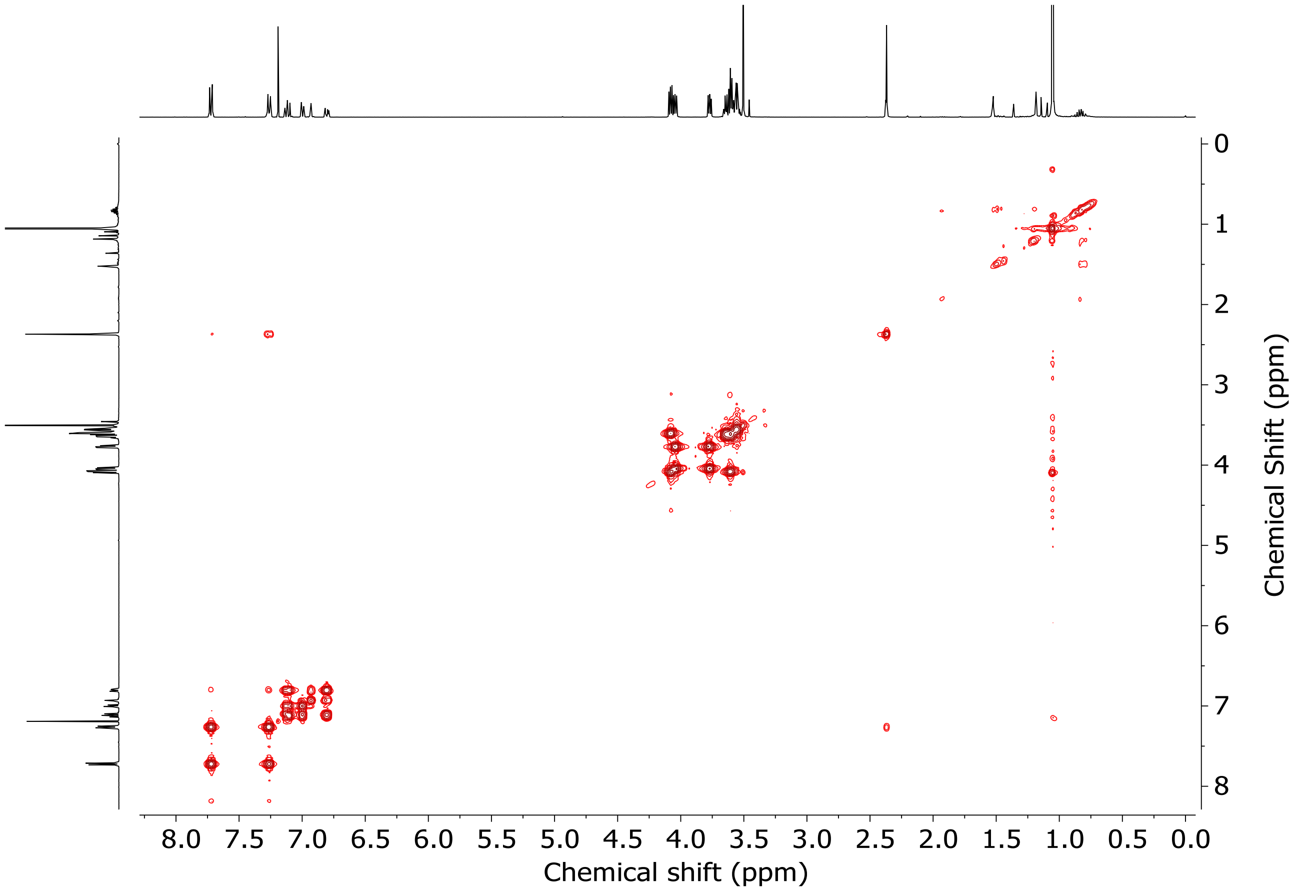

Figure S165: COSY NMR ($CDCl_3$) of **S22**.

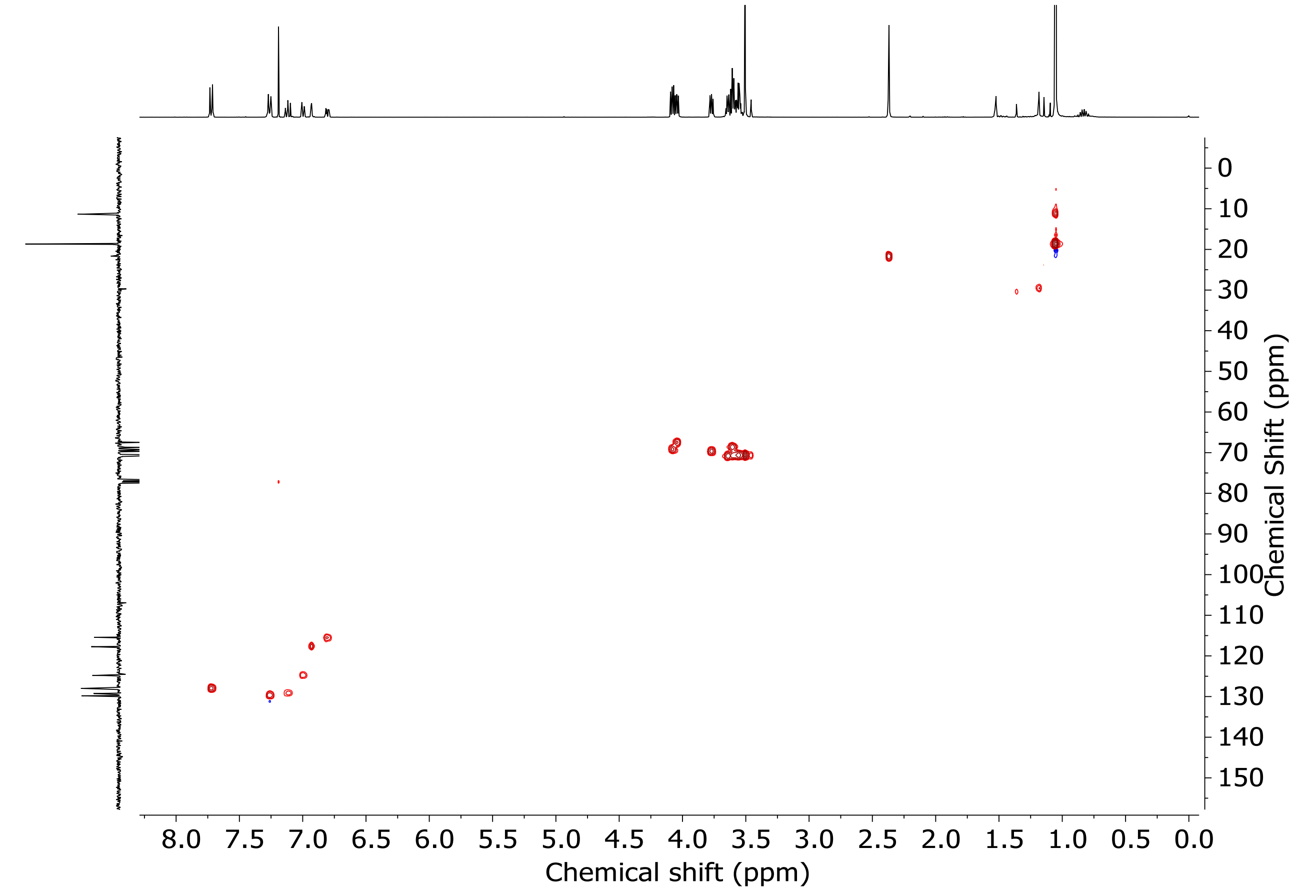

Figure S166: HSQC NMR ($CDCl_3$) of **S22**.

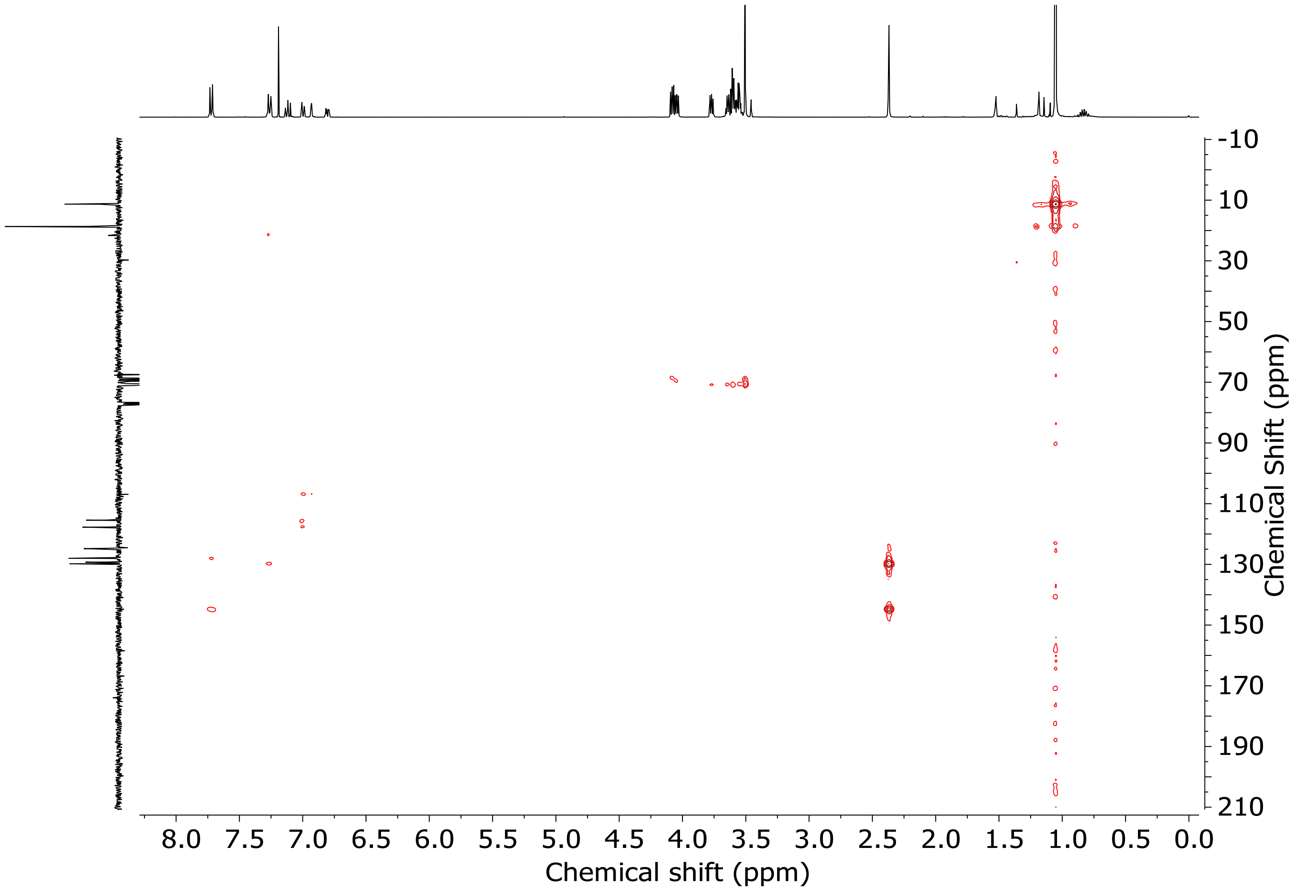

Figure S167: HMBC NMR ($CDCl_3$) of **S22**.

Amine TIPS aryl ether S23

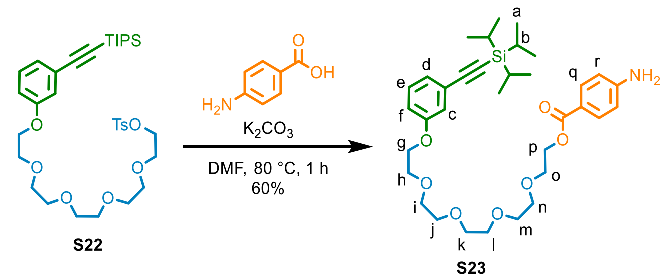

4-aminobenzoic acid (0.82 g, 6.0 mmol) and K_2CO_3 (1.1 g, 8.0 mmol) were suspended in DMF (5 mL) and the resulting suspension was heated at 80 °C for 15 min. A solution of **S22** (1.3 g, 2.0 mmol) in DMF (5 mL) was added and the reaction mixture was stirred at 80 °C for 1 h. The reaction mixture was poured onto sat. $NaHCO_{3\,(aq)}$ (20 mL) then extracted with EtOAc (3 x 20 mL). The organic phases are washed with 5% $LiCl_{(aq)}$ solution (3 x 5 mL), washed with brine (20 mL), dried ($MgSO_4$) and concentrated *in vacuo*. Chromatography (petrol-Et_2O, 50→100%) gave **S23** as a yellow oil (754 mg, 59%).

^{1}H NMR (400 MHz, $CDCl_3$) δ: 7.85 (dt, J = 8.8, 2.1, 2H, H_q), 7.18 (t, J = 8.0, 1H, H_e), 7.06 (dt, J = 7.5, 1.2, 1H, H_f), 7.00 (dd, J = 2.5, 1.4, 1H, H_c), 6.87 (ddd, J = 8.4, 2.7, 1.0, 1H, H_d), 4.43-4.37 (m, 2H, H_p), 4.16-3.98 (m, 4H, H_g, NH_2), 3.86-3.76 (m, 4H, H_o, H_h), 3.74-3.59 (m, 12H, H_i, H_j, H_k, H_l, H_m, H_n), 1.12 (app s, 21H, H_a, H_b)

^{13}C NMR (101 MHz, $CDCl_3$) δ: 166.6, 158.4, 150.9, 131.7, 129.3, 124.8, 124.5, 119.6, 117.7, 115.5, 113.7, 106.9, 90.4, 70.8, 70.6 (×5), 69.7, 69.4, 67.5, 63.6, 18.7, 11.3.

HR-ESI-MS (+ve) m/z = 636.3351 $[M+Na]^+$ (calc. 636.3327 m/z for $C_{34}H_{51}NNaO_7Si$);

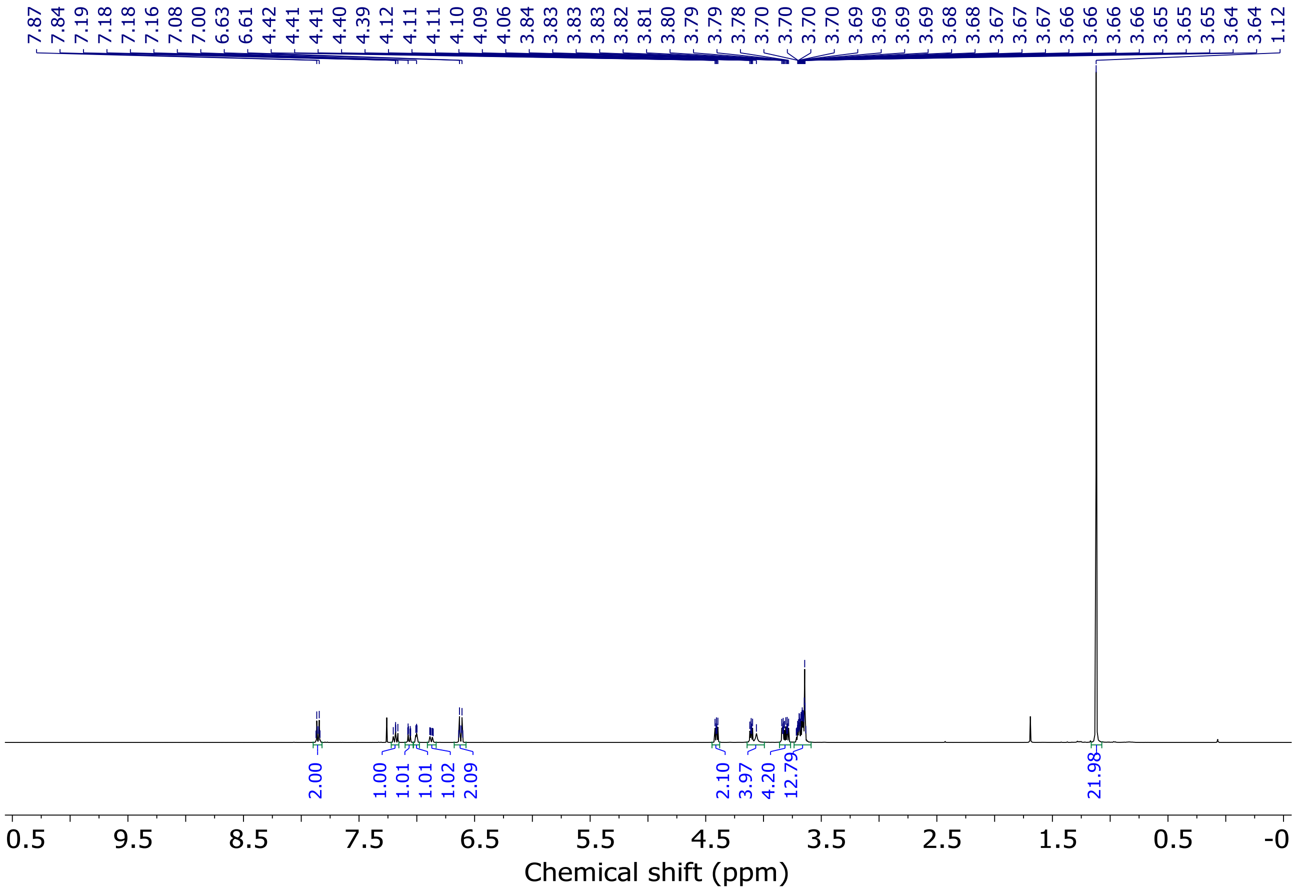

Figure S168: ^{1}H NMR ($CDCl_3$, 400 MHz) of **S23**.

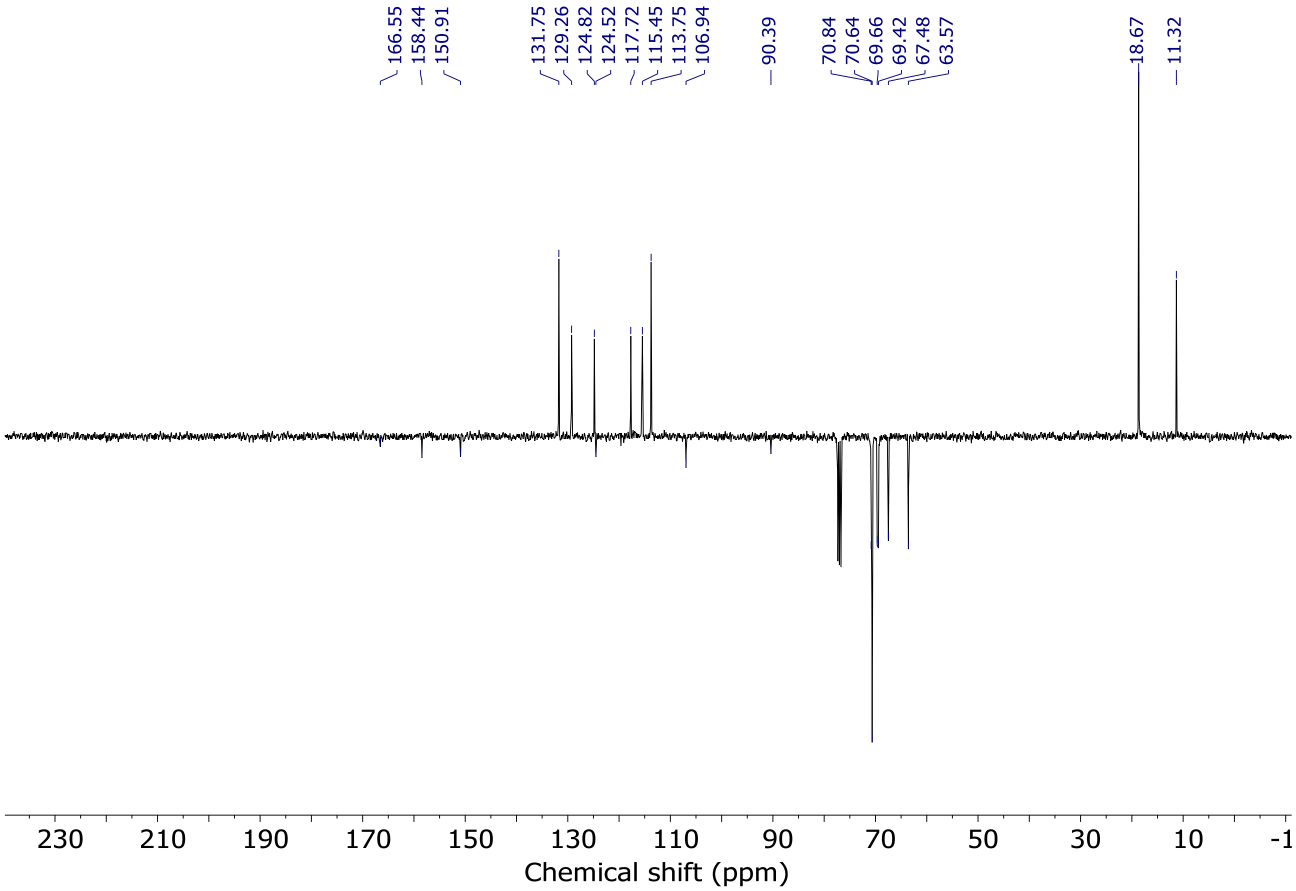

Figure S169: JMOD NMR ($CDCl_3$, 101 MHz) of **S23**.

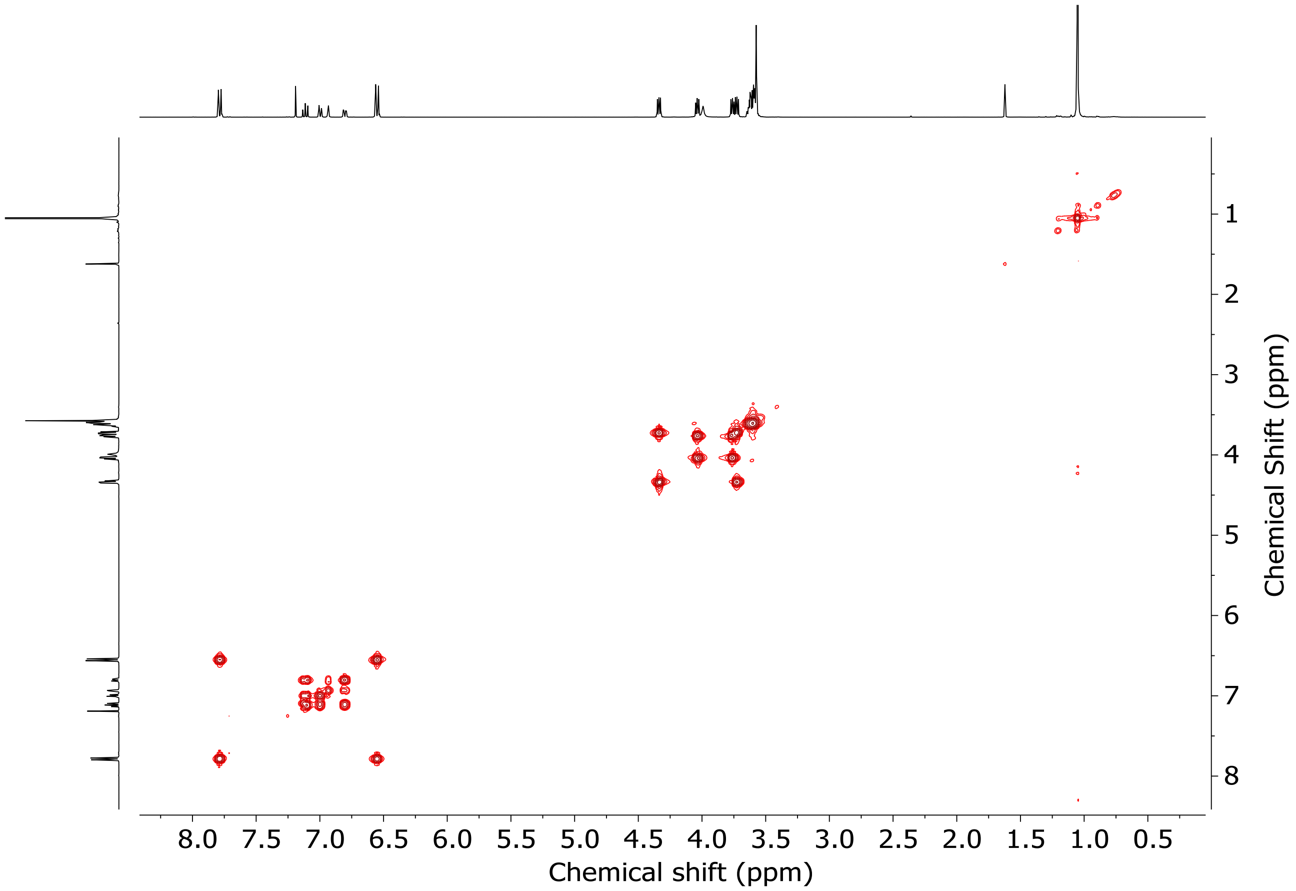

Figure S170: COSY NMR ($CDCl_3$) of **S23**.

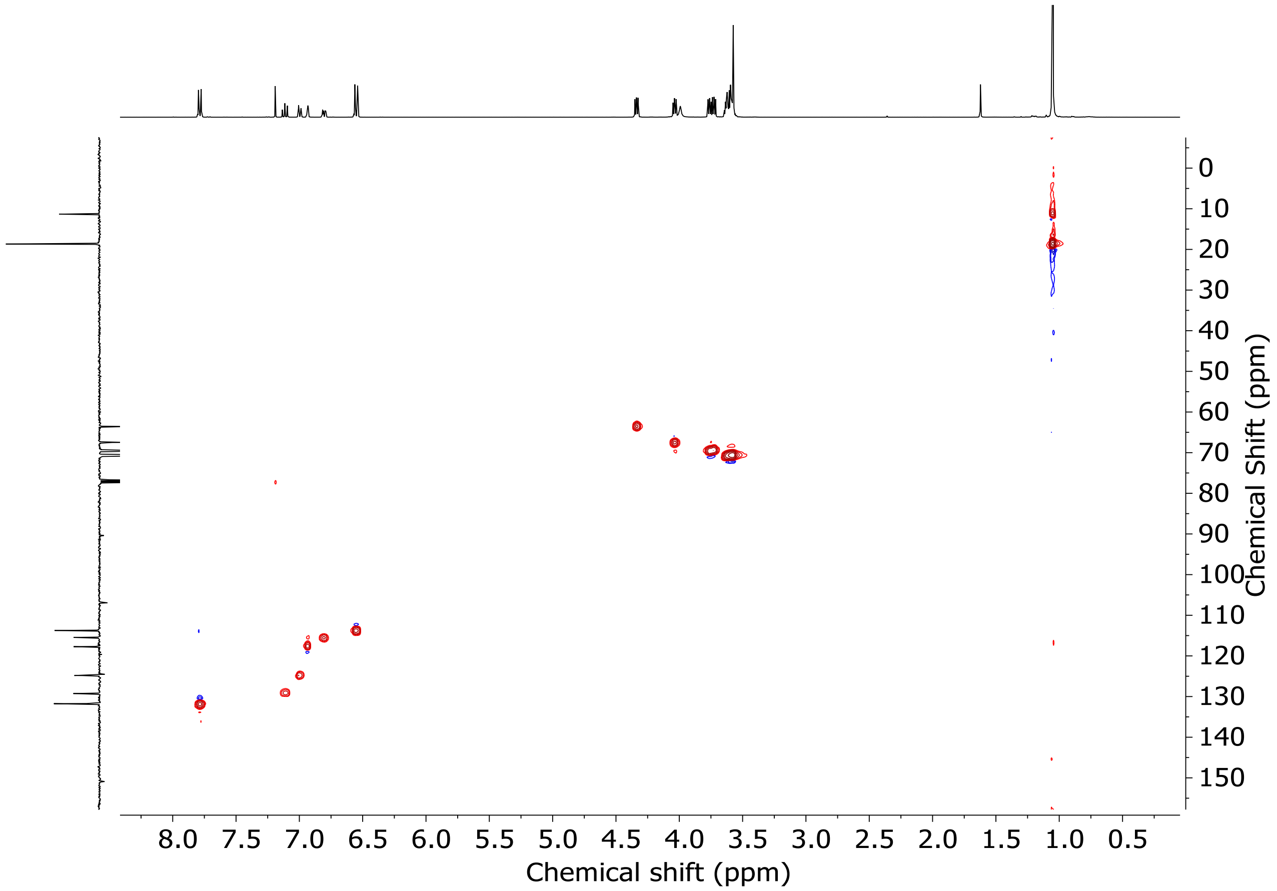

Figure S171: HSQC NMR ($CDCl_3$) of **S23**.

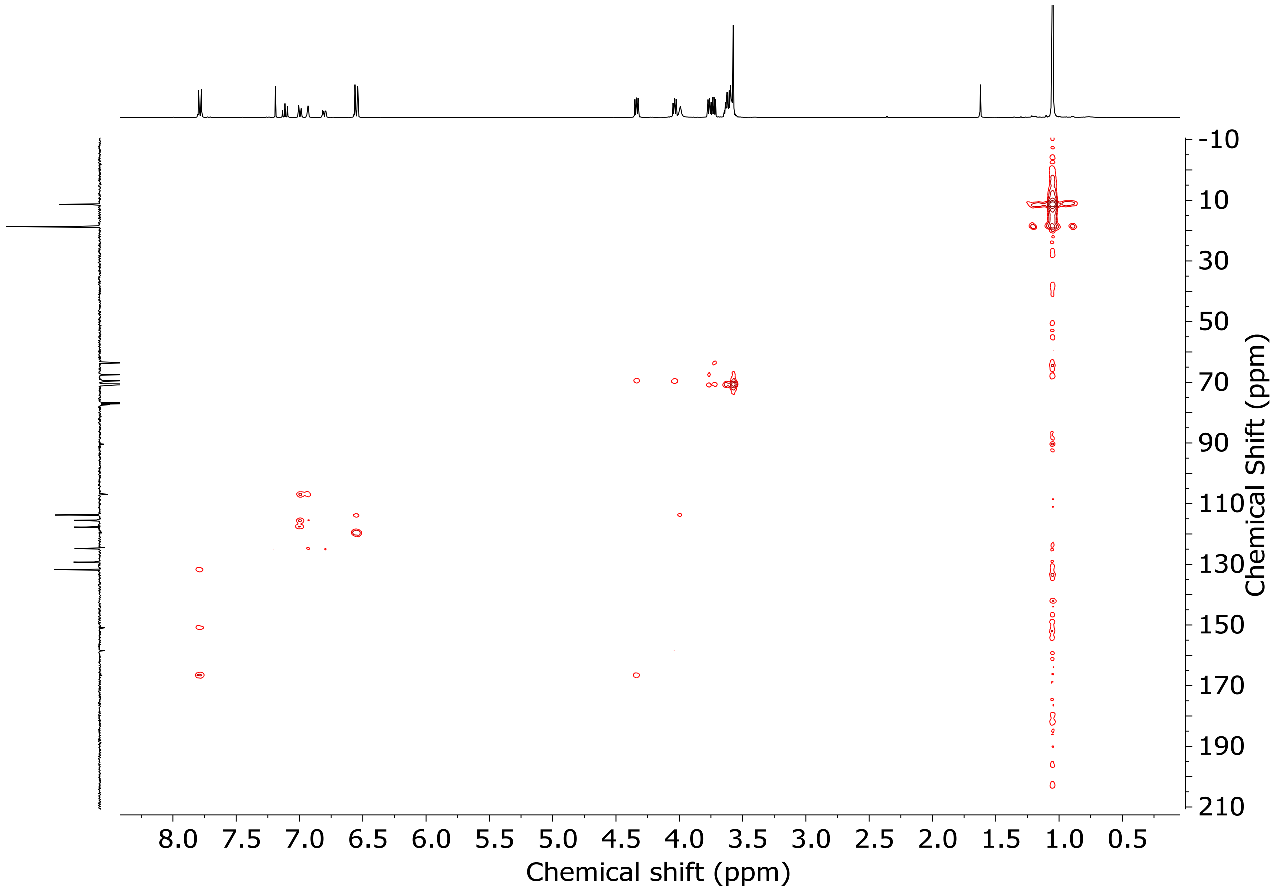

Figure S172: HMBC NMR ($CDCl_3$) of **S23**.

Azide TIPS aryl ether S24

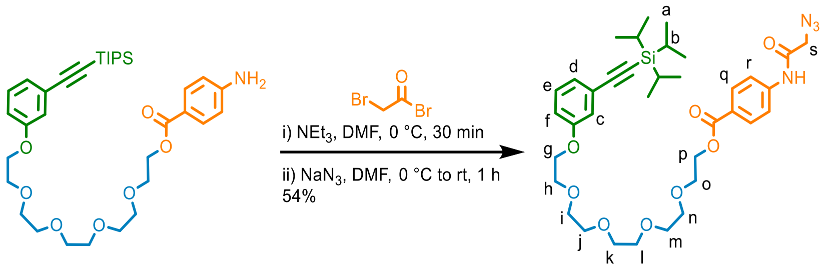

To a solution of **S23** (690 mg, 1.12 mmol) in DMF (5 mL) at 0 °C was added Et_3N (0.63 mL, 4.5 mmol) bromoacetyl bromide (0.15 mL, 1.7 mmol) dropwise. The solution turns red and was stirred at 0 °C for 30 minutes. NaN_3 (219 mg, 3.4 mmol) was then added portion wise at 0 °C. The solution was allowed to warm at rt and it was stirred for 1 h. The reaction mixture was then quenched by adding H_2O (10 mL) dropwise. The aqueous and organic phases were separated, and the aqueous phase was then extracted with EtOAc (3 x 20 mL). The combined organic extracts were washed with 5% $LiCl_{(aq)}$ (3 x 5 mL), brine (10 mL), dried ($MgSO_4$) and concentrated *in vacuo*. Chromatography (petrol-Et_2O 70→100%) gave **S24** as a yellow oil (432.4 mg, 54%).

^{1}**H NMR (500 MHz, CDCl$_3$)** δ: 8.16 (bs, 1H, NH), 8.05 (dt, J = 8.8, 2.0, 2H, H$_q$), 7.64 (dt, J = 8.8, 2.0, 2H, H$_r$), 7.18 (app. t, J = 8.0, 1H, H$_e$), 7.07 (dt, J = 7.5, 1.1, 1H, H$_f$), 7.00 (dd, J = 2.5, 1.4, 1H, H$_c$), 6.87 (ddd, J = 8.4, 2.7, 1.0, 1H, H$_d$), 4.48-4.43 (m, 2H, H$_p$), 4.17 (s, 2H, H$_s$), 4.14-4.09, (m, 2H, H$_g$), 3.86-3.76 (m, 4H, H$_o$, H$_h$), 3.75-3.59 (m, 12H, H$_i$, H$_j$, H$_k$, H$_l$, H$_m$, H$_n$), 1.12 (app s, 21H, H$_a$, H$_b$).

^{13}**C NMR (126 MHz, CDCl$_3$)** δ: 165.9, 164.7, 158.4, 140.9, 131.0, 129.3, 126.4, 124.8, 124.5, 119.1, 117.7, 115.4, 106.9, 90.4, 70.8, 70.7, 70.7, 70.6, 70.6, 69.7, 69.2, 67.5, 64.1, 53.0, 18.7, 11.3.

HR-ESI-MS (+ve) m/z = 697.3598 [M+H]$^+$ (calc. 697.3627 m/z for $C_{36}H_{53}N_4O_8Si$);

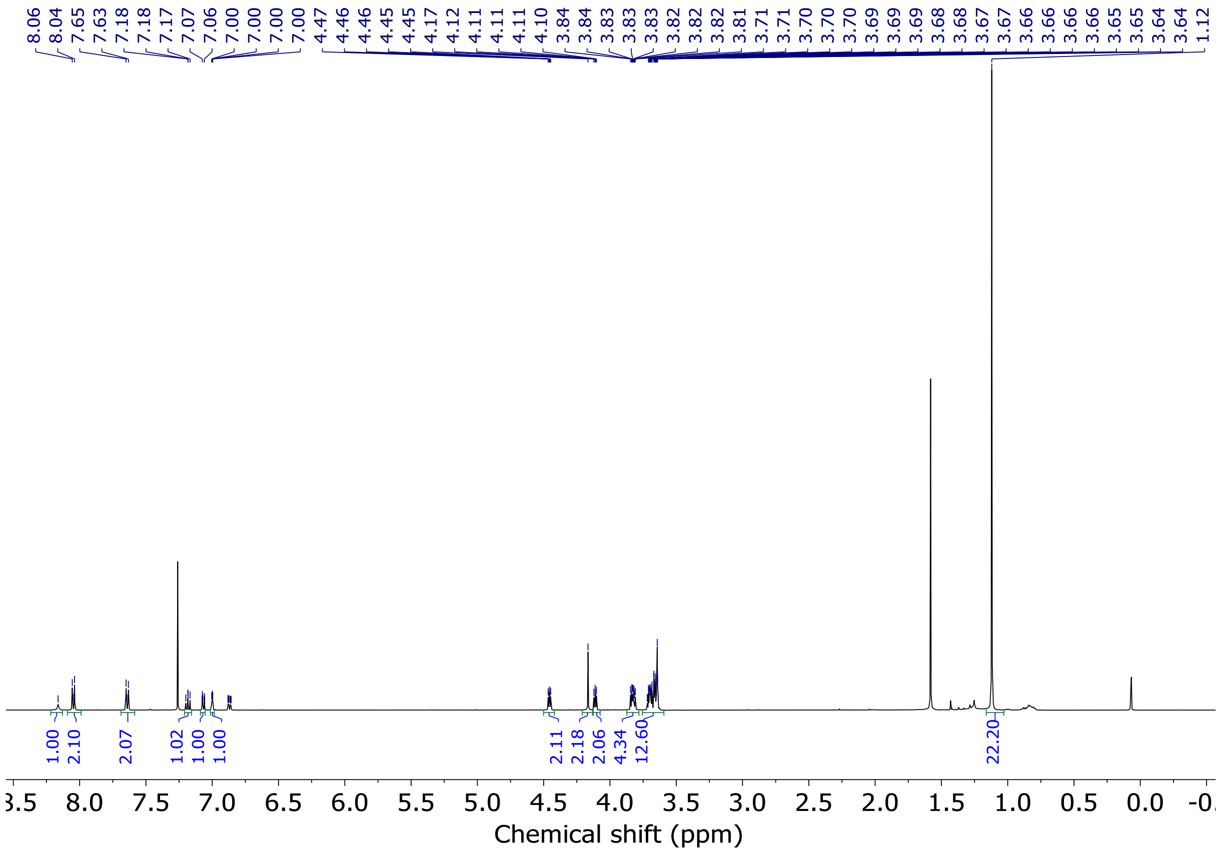

Figure S173: ^{1}H NMR (CDCl$_3$, 500 MHz) of **S24**.

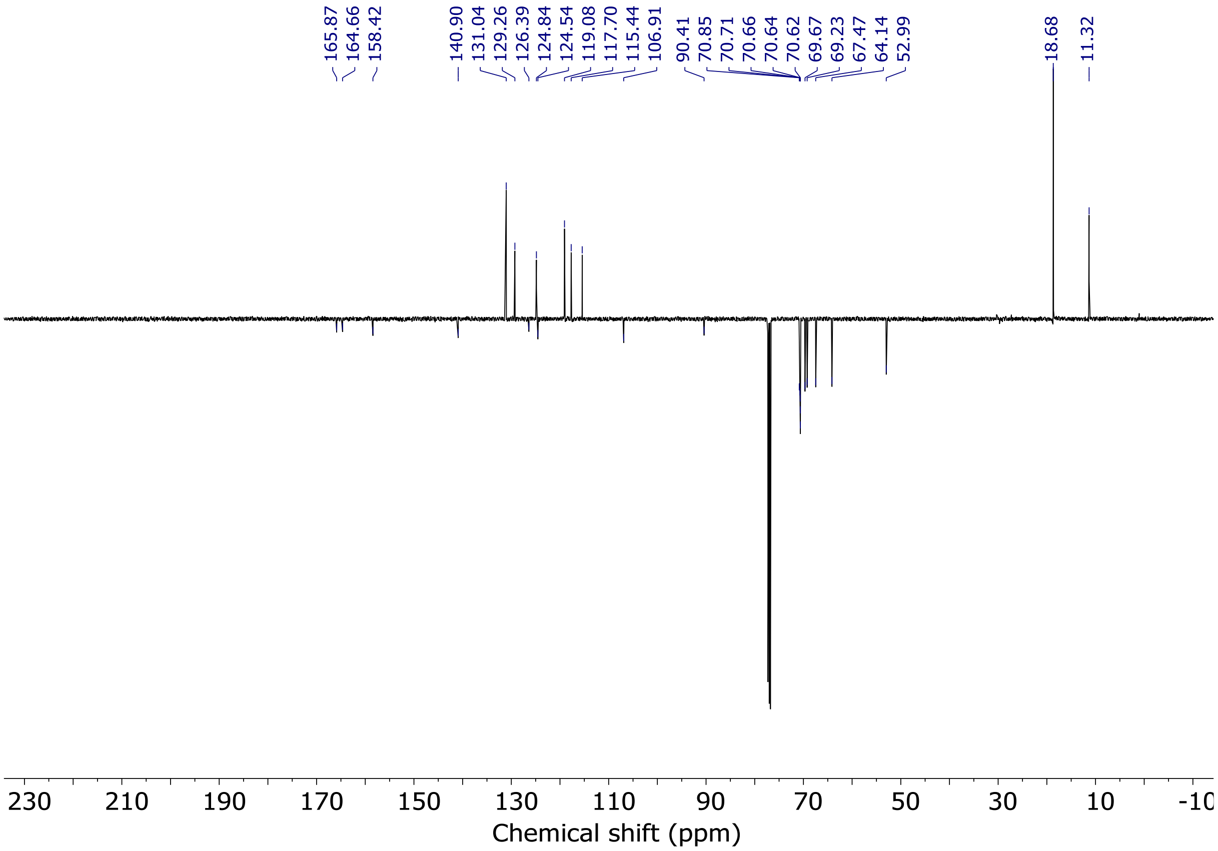

Figure S174: JMOD NMR ($CDCl_3$, 126 MHz) of **S24**.

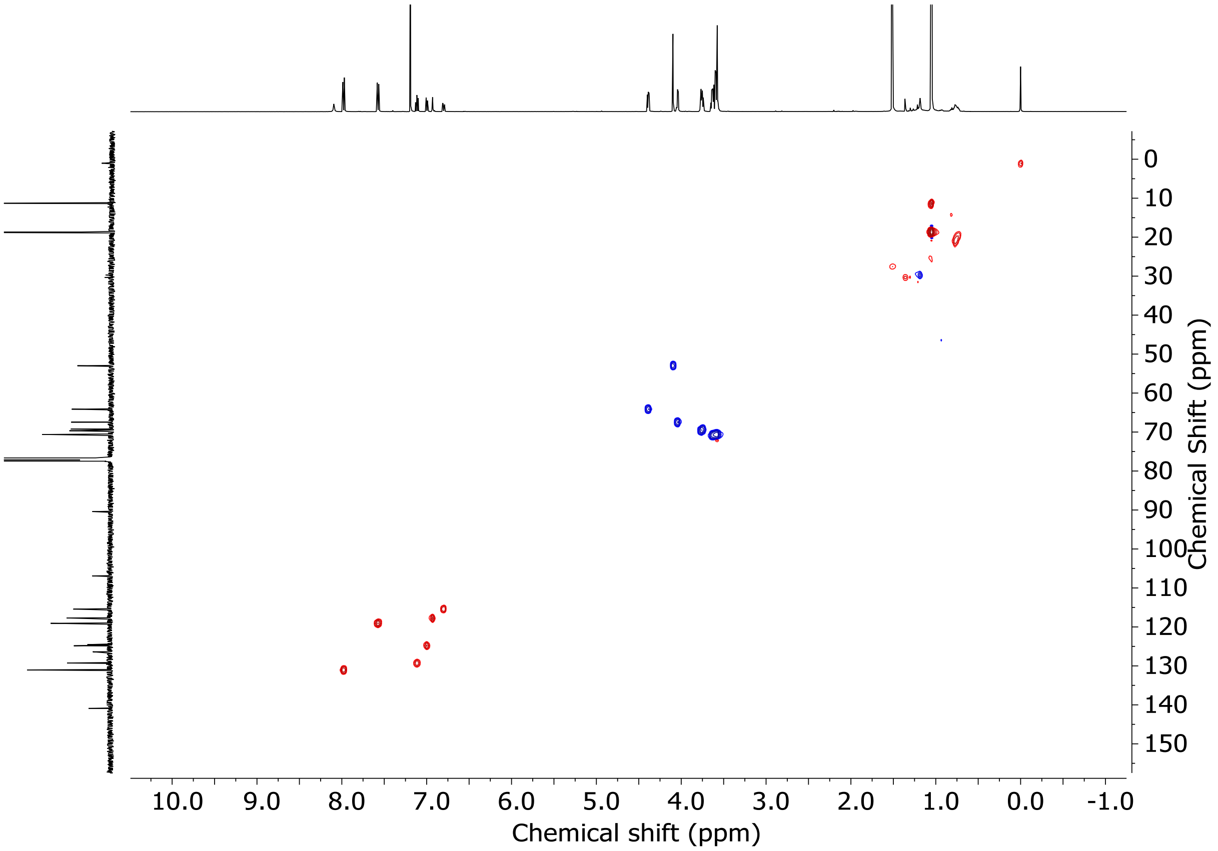

Figure S175: HSQC NMR ($CDCl_3$) of **S24**.

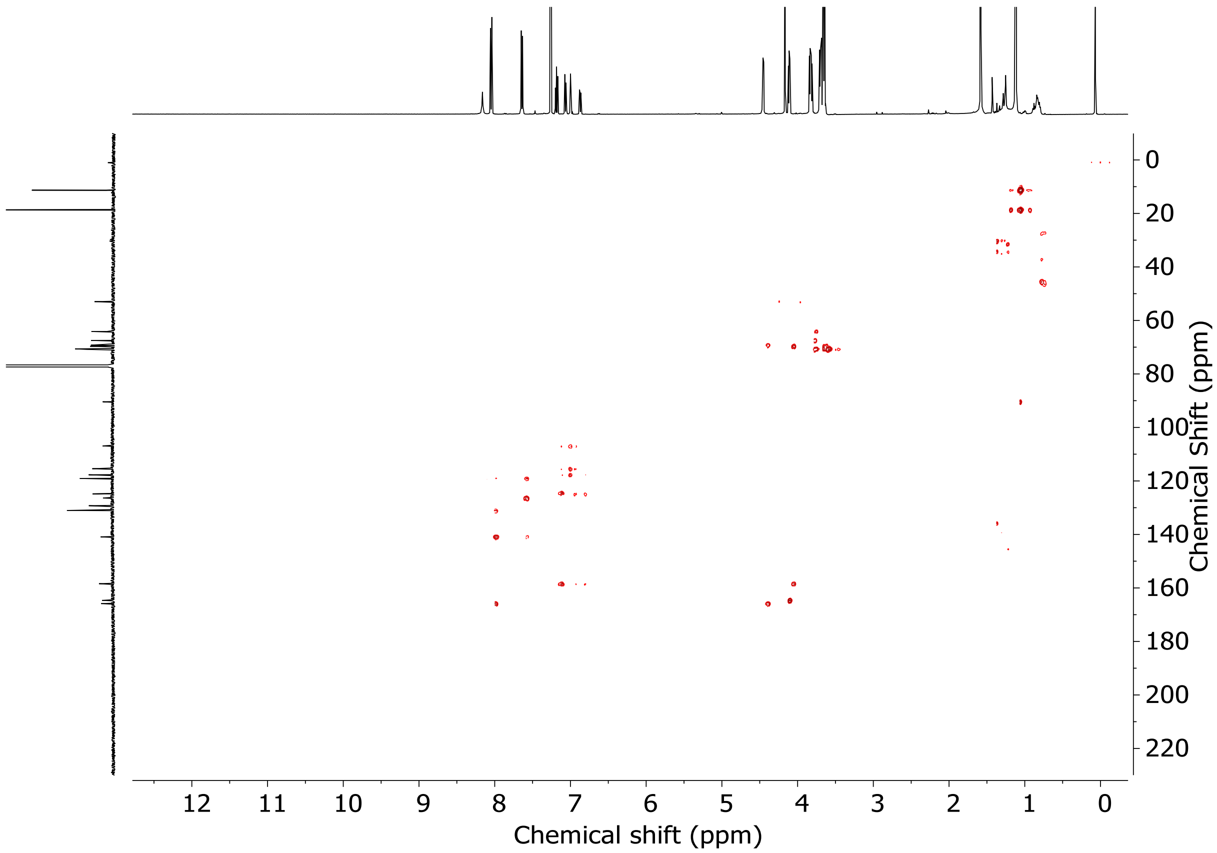

Figure S176: HMBC NMR ($CDCl_3$) of **S24**.

Macrocycle precursor 13

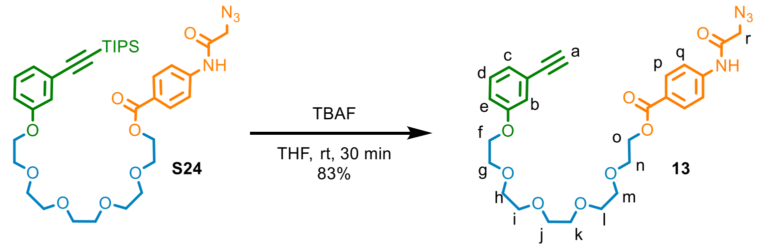

To a solution of **S24** (432 mg, 0.62 mmol) in THF (6 mL) was added a 1M solution of TBAF in THF (0.86 mL, 0.86 mmol). The solution was stirred at rt for 30 min. The reaction mixture was partitioned between H_2O (10 mL) and EtOAc (20 mL), the phases separated, and the organic layer extracted with EtOAc (2 x 20 mL). The collected organic fractions were then washed with brine (20 mL), dried ($MgSO_4$) and the solvent was removed *in vacuo.* Chromatography (*n*-hexane-EtOAC 0 → 60%) gave **14** as a colourless oil (253 mg, 83%).

^{1}H NMR (500 MHz, $CDCl_3$) δ: 8.29 (bs, 1H, NH), 8.01 (dt, *J* = 9.0, 2.2, 2H, H_p), 7.63 (dt, *J* = 8.8, 2.0, 2H, H_q), 7.19 (app. t, *J* = 8.2, 1H, H_d) 7.07 (dt, *J* = 7.6, 1.2, 1H, H_c) 7.00 (dd, *J* = 2.6, 1.4, 1H, H_b) 6.89 (ddd, 8.3, 2.7, 0.9, 1H, H_e), 4.47-4.41 (m, 2H, H_o), 4.13 (s, 2H, H_r), 4.11-4.05 (m, 2H, H_f), 3.84-3.78 (m, 4H, H_g, H_n), 3.73-3.60 (m, 12H, H_h, H_i, H_j, H_k, H_l, H_m) 3.05 (s, 1H, H_a).

^{13}C NMR (126 MHz, $CDCl_3$) δ: 165.9, 164.9, 158.5, 141.1, 131.0, 129.4, 126.2, 124.9, 123.1, 119.1, 117.7, 116.0, 83.5, 70.8, 70.7, 70.6, 70.6, 70.6, 70.6, 69.6, 69.2, 67.5, 64.1, 52.9.

HR-ESI-MS (+ve) *m/z* = 563.2148 $[M+Na]^+$ (calc. 563.2112 *m/z* for $C_{27}H_{32}N_4NaO_8$).

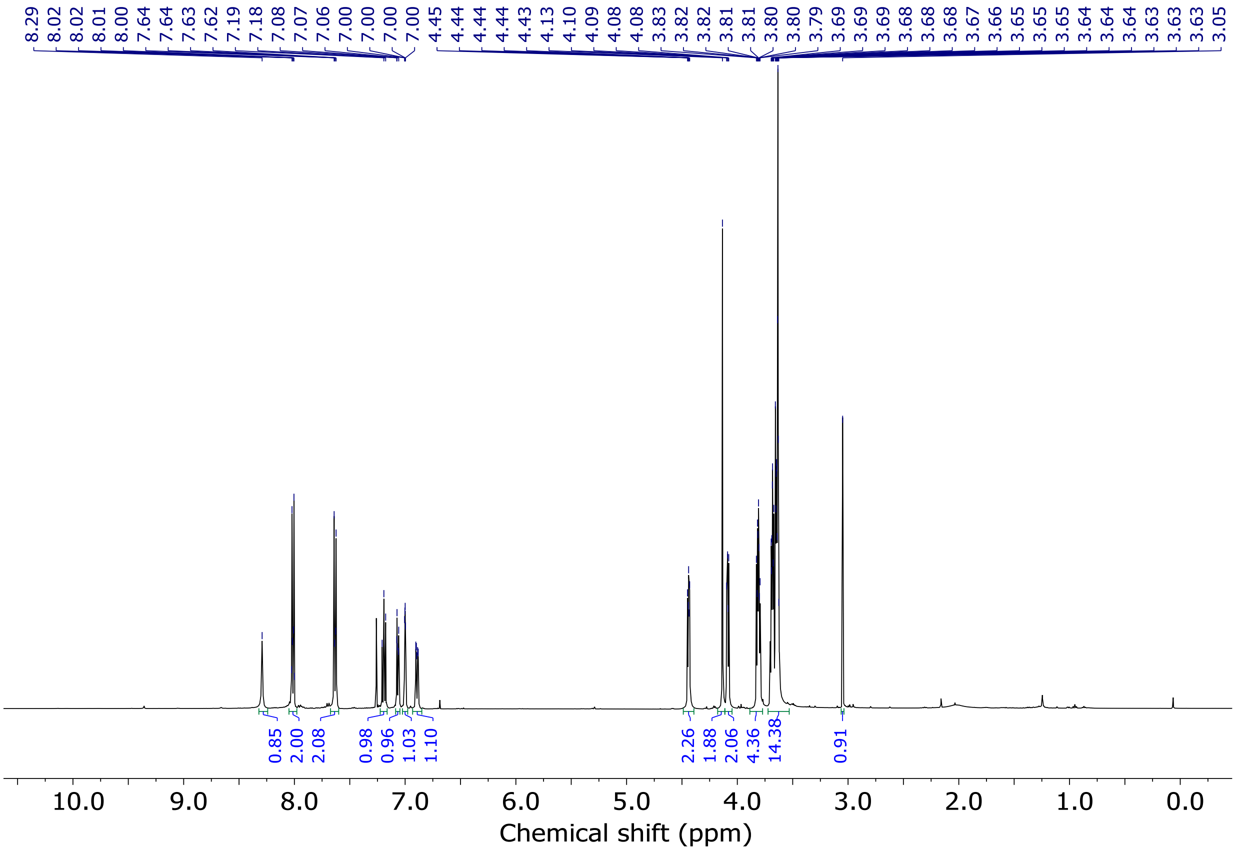

Figure S177: ^{1}H NMR ($CDCl_3$, 500 MHz) of **13**.

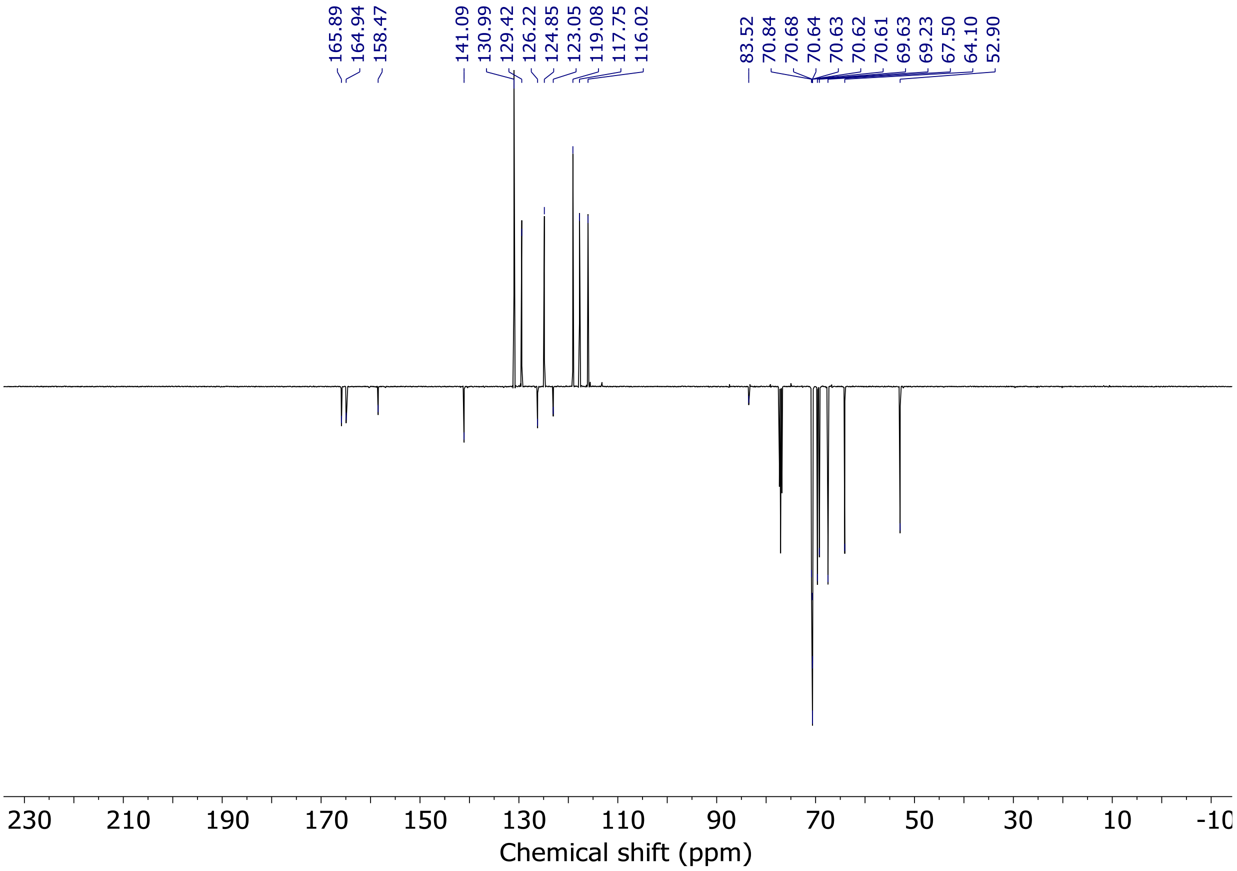

Figure S178: JMOD NMR ($CDCl_3$, 126 MHz) of **13**.

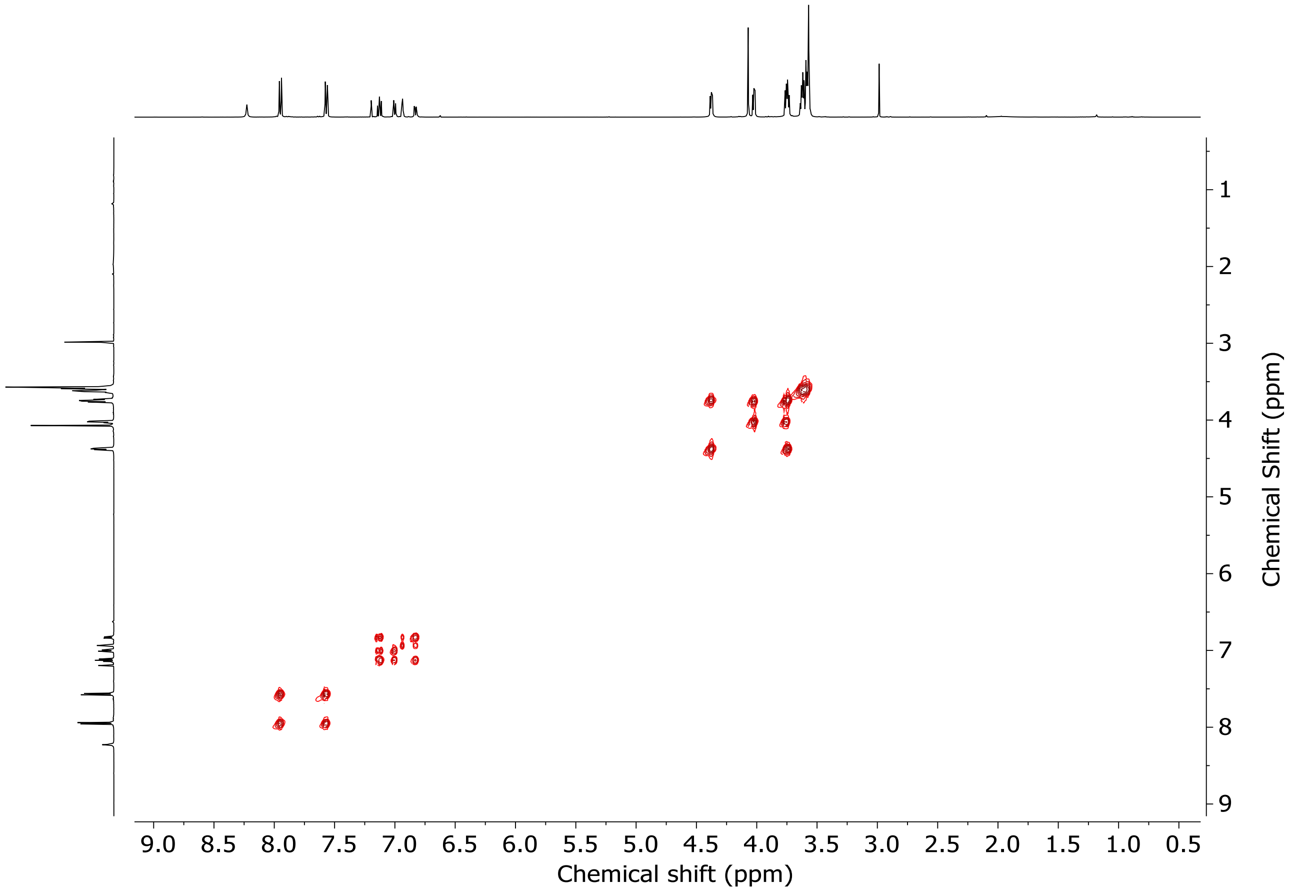

Figure S179: COSY NMR ($CDCl_3$) of **13**.

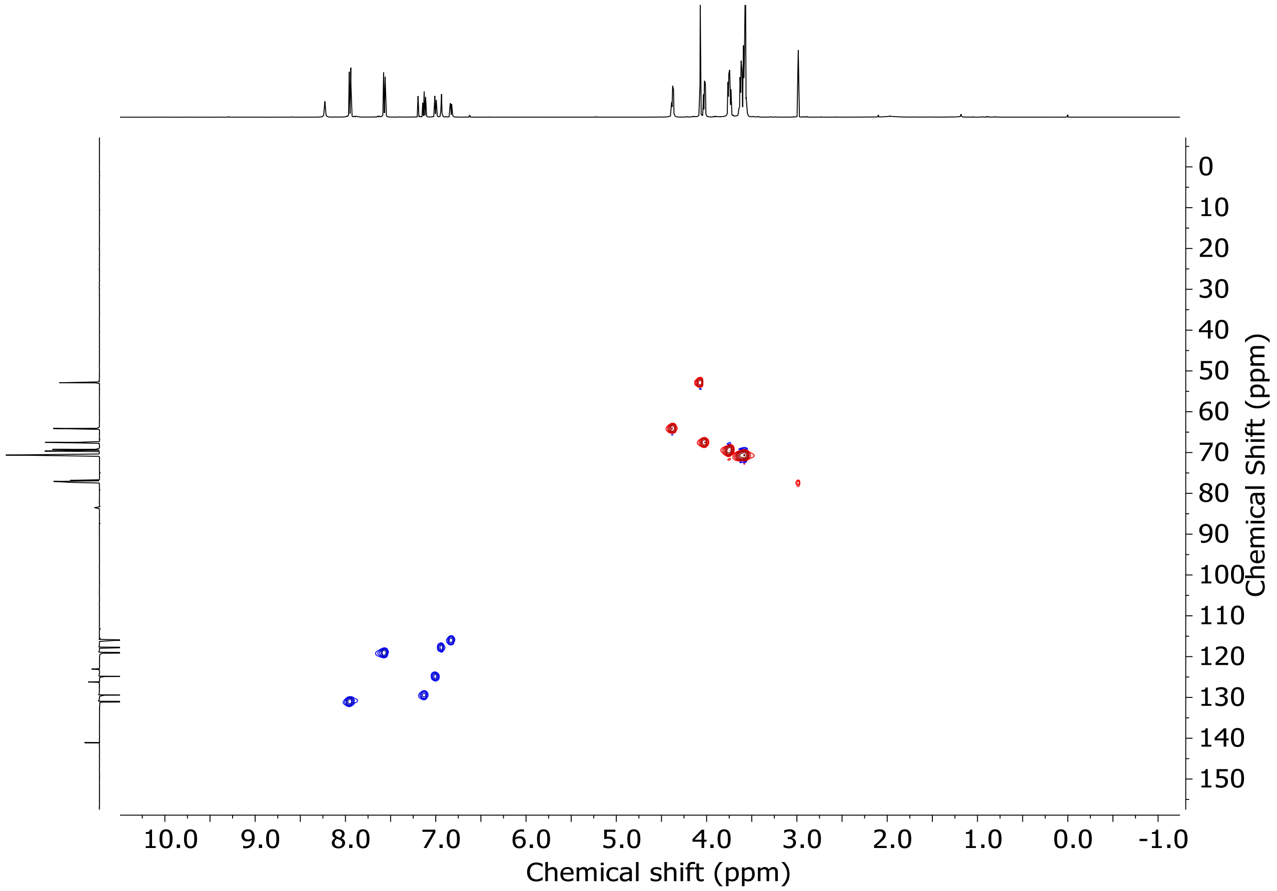

Figure S180: HSQC NMR ($CDCl_3$) of **13**.

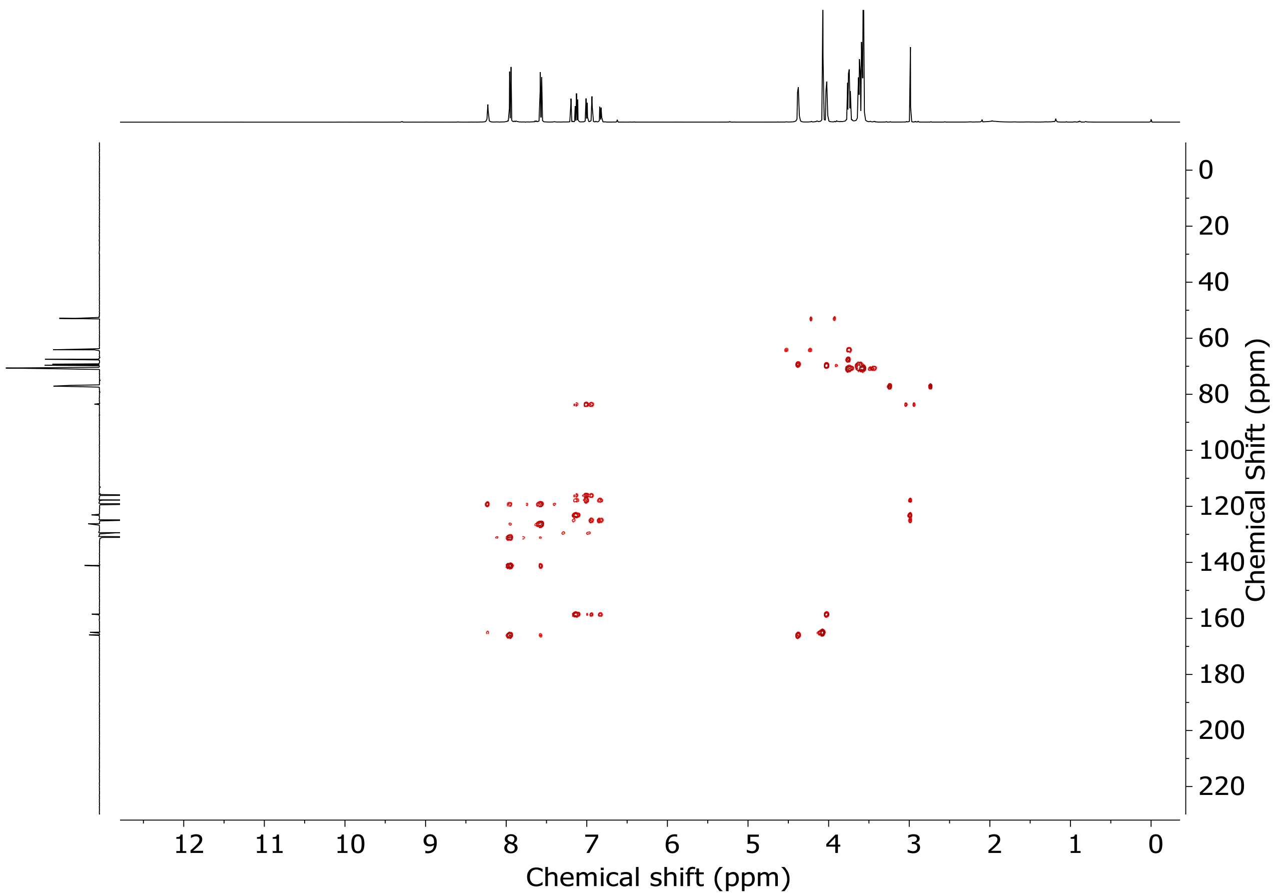

Figure S181: HMBC NMR ($CDCl_3$) of **13**.

Catenanes (E_m)-14 and (Z_m)-14

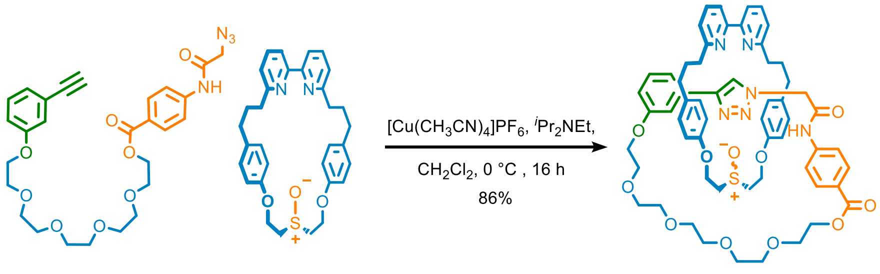

To a solution of **2** (19.0 mg, 36 µmol), $[Cu(CH_3CN)_4]PF_6$ (13.1 mg, 35 µmol) and $^iPr_2NEt$ (25 µL, 140 µmol) in CH_2Cl_2 (3.6 mL) at 0 °C was added a solution of **13** (39.2 mg, 73 µmol) in CH_2Cl_2 over 16 hours. Once addition had finished, to the reaction mixture at 0 °C was added TFA (500 µL) and stirred for 1 hour until complete decolouration. The crude mixture was diluted with CH_2Cl_2 (5 mL) and carefully poured onto EDTA-NH_3 (5 mL), with separation of aqueous and organic phases. The combined aqueous phase was then extracted with CH_2Cl_2 (3 x 5 mL) and the combined organic extracts were washed with brine (10 mL), dried ($MgSO_4$) and concentrated *in vacuo* to give a sample containing **14** as a mixture of diastereomers (95 : 5 *dr*, Figure S182). Chromatography ([1:1 CH_2Cl_2-PhMe]-acetone 0→100% with 2% EtOH) gave catenanes-**14** as a white foam (32.8 mg, 86%) as a mixture of diastereoisomers (4.9 : 1 *dr*, Figure S183).

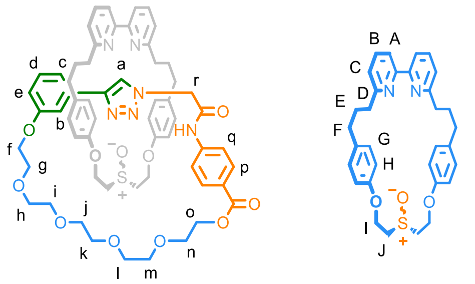

Major diastereoisomer

^{1}H NMR (500 MHz, $CDCl_3$) δ: 11.38 (s, 1H, NH), 7.90 (dt, J = 8.7, 1.9, 2H, H_p), 7.78 (t, J = 7.7, 2H, H_B), 7.67 (dt, J = 7.8, 1.3, 1H, H_b), 7.64-7.58 (m, 2H, H_A), 7.38 (app.t, J = 7.8, 1H, H_d), 7.35-7.22 (m, 5H, H_a, H_C, H_q superimposed with $CDCl_3$), 6.98-6.94 (m, 1H, H_e), 6.88-6.82 (m, 1H, H_d), 6.77-6.69 (m, 8H, H_G, H_H), 4.70-4.55 (m, 4H, H_I, H_r), 4.54-4.48 (m, 2H, H_o), 4.44 (dt, J = 12.9, 4.2, 2H, $H_{I'}$

), 4.14-4.09 (m, 2H, H_f), 3.86-3.74 (m, 4H, H_g, H_n), 3.72-3.57 (m, 2H, H_m), 3.55 (t, J = 5.5, 2H, H_h), 3.51-3.35 (m, 4H, H_i, H_l), 3.35-3.25 (m, 4H, H_j, H_k), 3.24-3.12 (m, 2H, H_J), 2.70-2.57 (m, 4H, H_F, $H_{J'}$), 2.56-2.28 (m, 6H, $H_{F'}$, H_D), 1.88-1.72 (m, 4H, H_E)

^{13}C NMR (126 MHz, $CDCl_3$) δ: 166.4, 163.1, 162.6, 158.9, 156.2, 155.3, 147.4, 142.2, 138.0, 133.1, 131.7, 130.4, 130.2, 129.3, 125.4, 122.6, 120.9, 120.4, 119.7, 117.9, 115.7, 113.6, 112.8, 70.5, 70.5, 70.2, 70.2, 69.7, 69.7, 69.6, 67.3, 64.4, 60.2, 52.9, 52.1, 36.5, 34.7, 31.2.

LR-ESI-MS m/z = 1016.5 $[M+H]^+$ for isotopic pattern see Figure S189

Minor diastereoisomer

^{1}H NMR (500 MHz, $CDCl_3$) 11.36 (s, 1H, NH), 7.84 (dt, J = 8.8, 2.0, 2H, H_p), 7.73 (t, J = 7.8, 2H, H_B), 7.64-7.58 (m, 1H, H_b), 7.55-7.51 (m, 3H, H_A, H_a), 7.35-7.22 (m, 3H, H_c, H_q, superimposed with $CDCl_3$), 7.18 (d, J =7.7, 2H, H_C), 6.98-6.94 (m, 1H, H_e), 6.88-6.82 (m, 1H, H_d), 6.77-6.69 (m, 8H, H_G, H_H), 4.70-4.55 (m, 2H, H_I), 4.54-4.47 (m, 6H, $H_{I'}$, H_o, H_r), 4.03 (t, J = 5.2, 2H, H_f), 3.86-3.74 (m, 4H, H_g, H_n), 3.72-3.57 (m, 8H, H_h, H_i, H_l, H_m), 3.51-3.35 (m, 4H, H_J, H_j or H_k), 3.35-3.25 (m, 2H, H_j or H_k), 3.24-3.12 (m, 2H, $H_{J'}$), 2.70-2.57 (m, 4H, H_F), 2.56-2.28 (m, 8H, H_F, H_D), 1.88-1.72 (m, 4H, H_E)

^{13}C NMR (126 MHz, $CDCl_3$) 166.2, 162.9, 162.7, 158.8, 156.2, 155.6, 147.5, 142.5, 137.9, 133.0, 131.8, 130.1, 129.9, 129.5, 125.3, 122.6, 121.3, 120.5, 119.9, 118.1, 114.9, 114.2, 112.7, 50.5, 70.5, 70.3, 70.2, 69.6, 67.0, 64.4, 59.5, 52.8, 50.2, 36.5, 34.8, 31.3.

HR-ESI-MS (+ve) m/z = 1067.5 $[M+H]^+$ for isotopic pattern see Figure S189

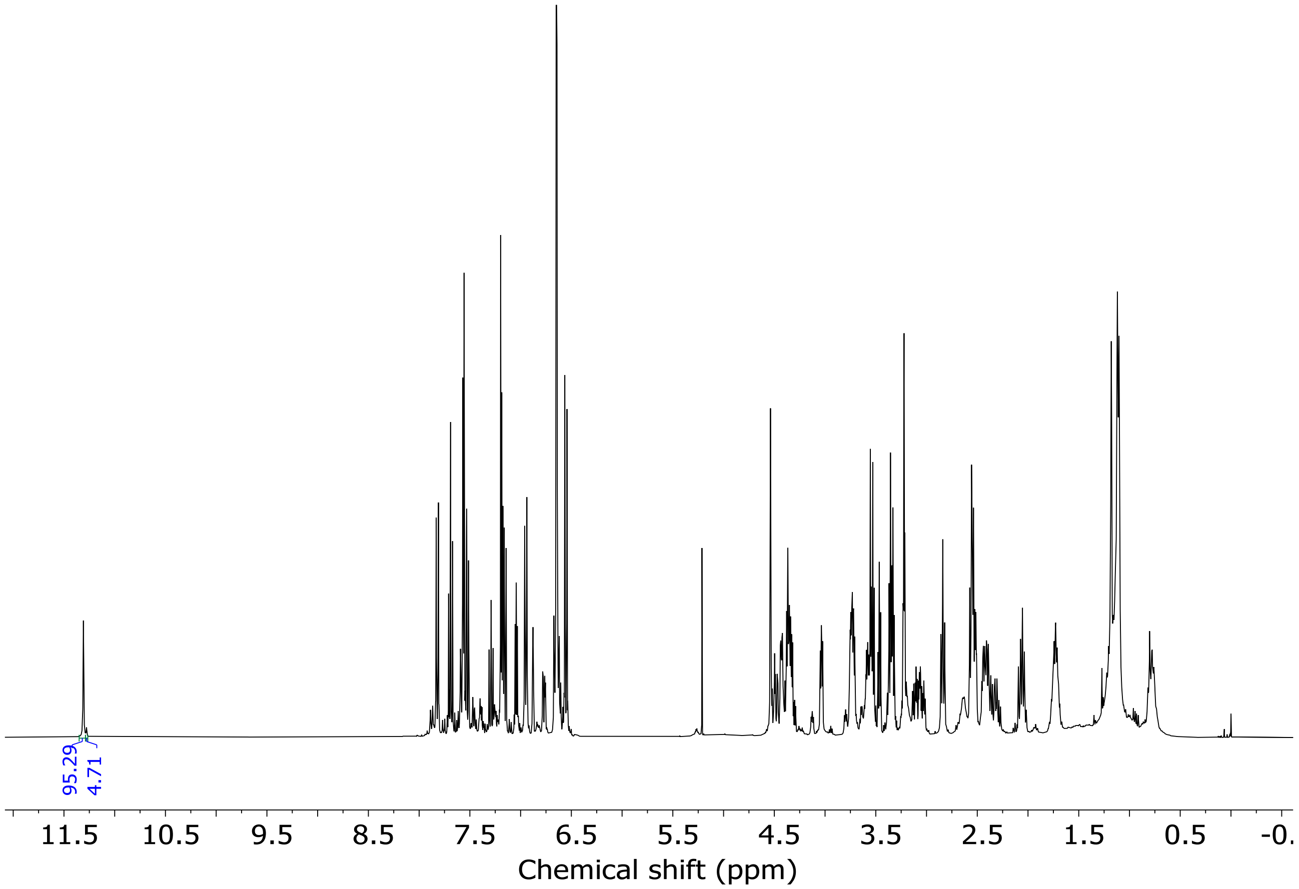

Figure S182: ^{1}H NMR ($CDCl_3$, 500 MHz) of catenanes **14** prior to chromatography (95 : 5 *dr*).

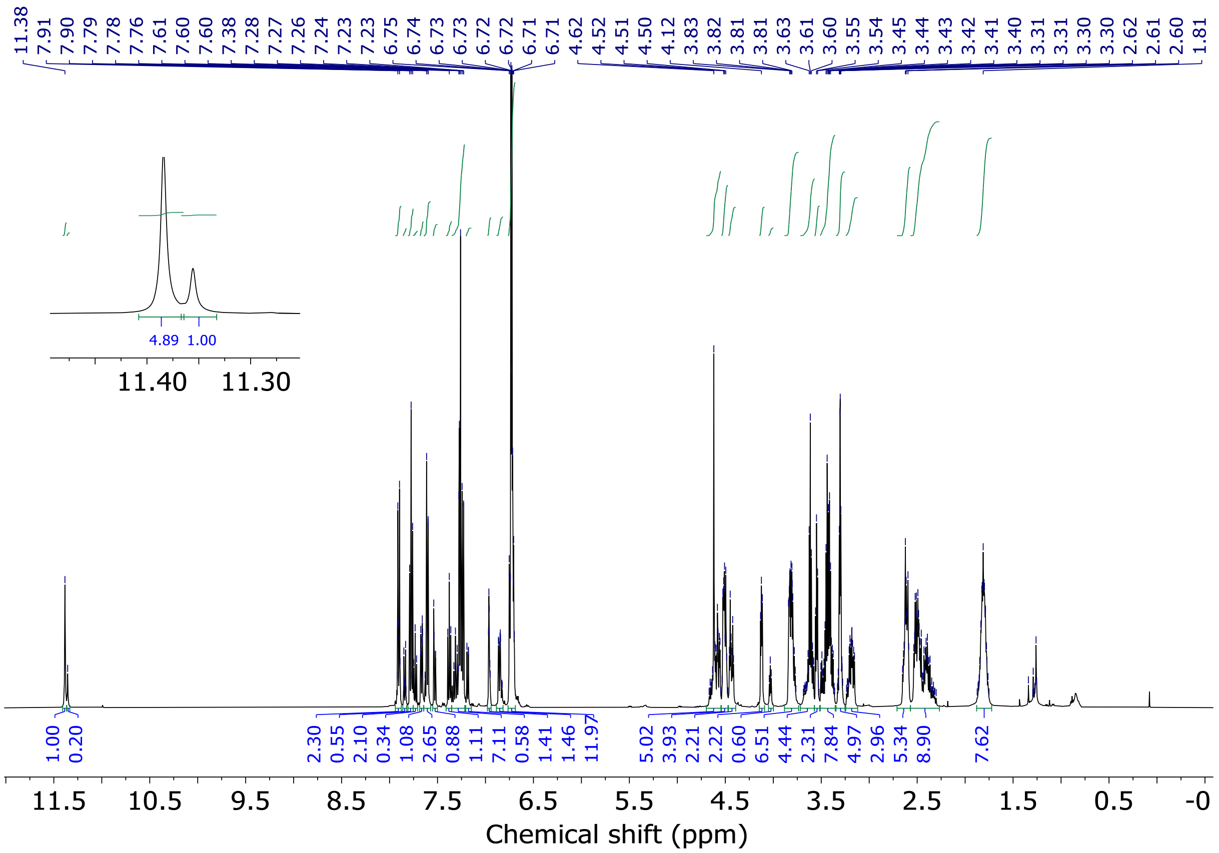

Figure S183: ^{1}H NMR ($CDCl_3$, 500 MHz) of (E_m)-**14** and (Z_m)-**14** (4.9 : 1 *dr*).

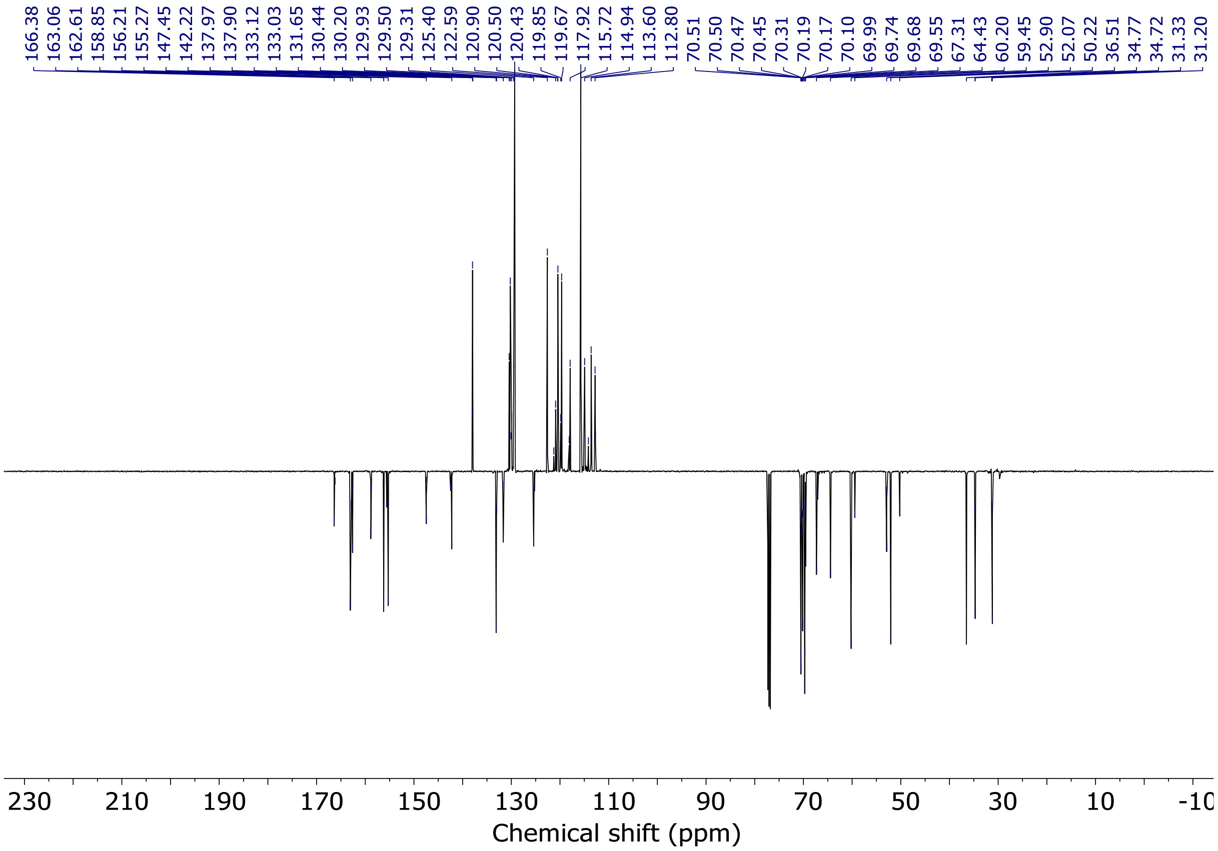

Figure S184: JMOD NMR ($CDCl_3$, 126 MHz) of (E_m)-**14** and (Z_m)-**14** (4.9 : 1 *dr*).

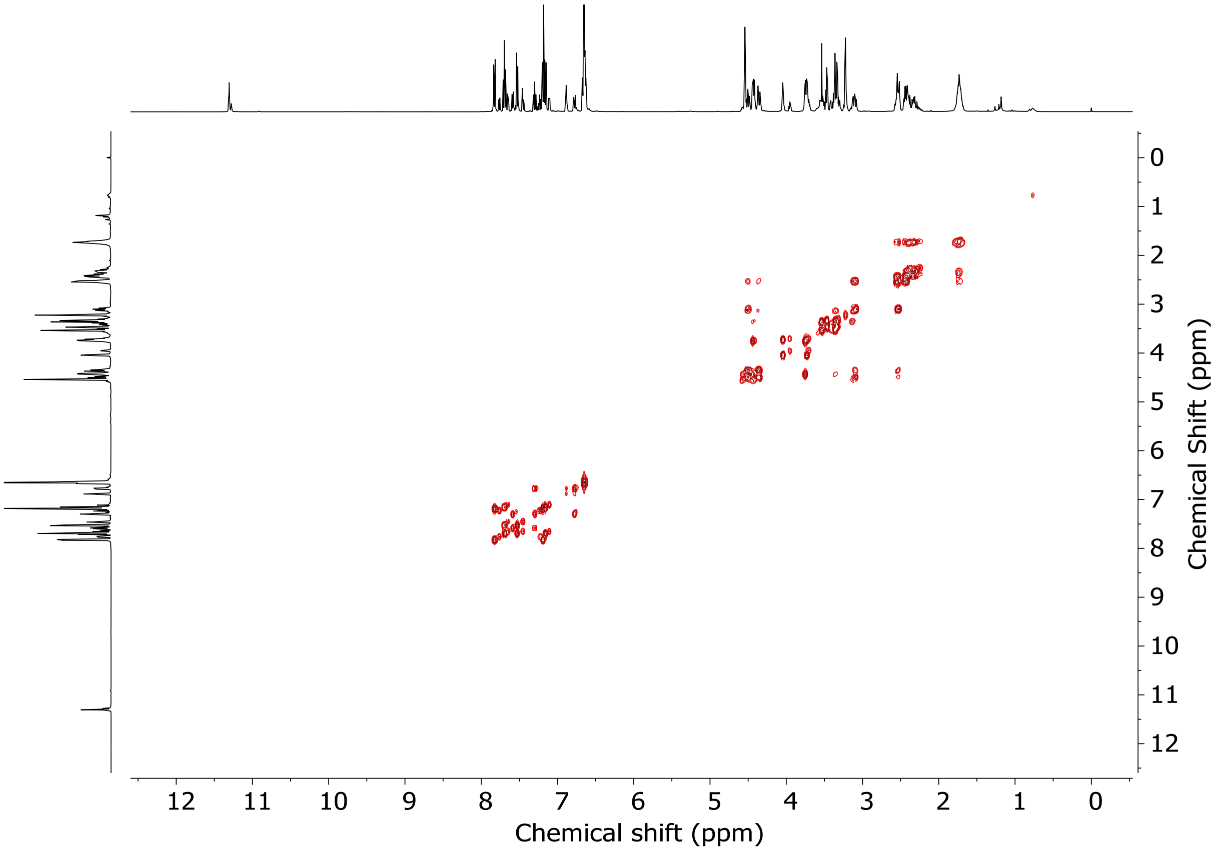

Figure S185: COSY NMR ($CDCl_3$) of (E_m)-**14** and (Z_m)-**14** (4.9 : 1 *dr*).

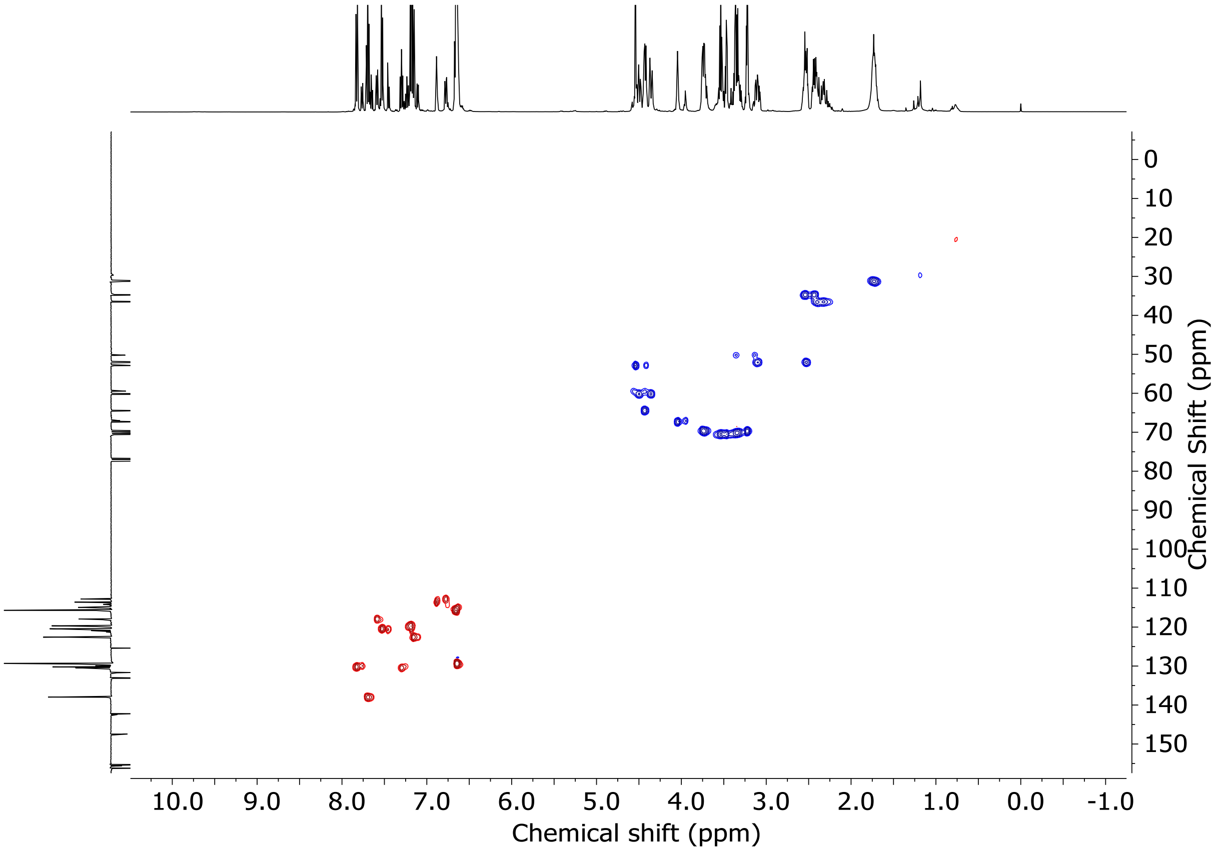

Figure S186: HSQC NMR ($CDCl_3$) of (E_m)-**14** and (Z_m)-**14** (4.9 : 1 *dr*).

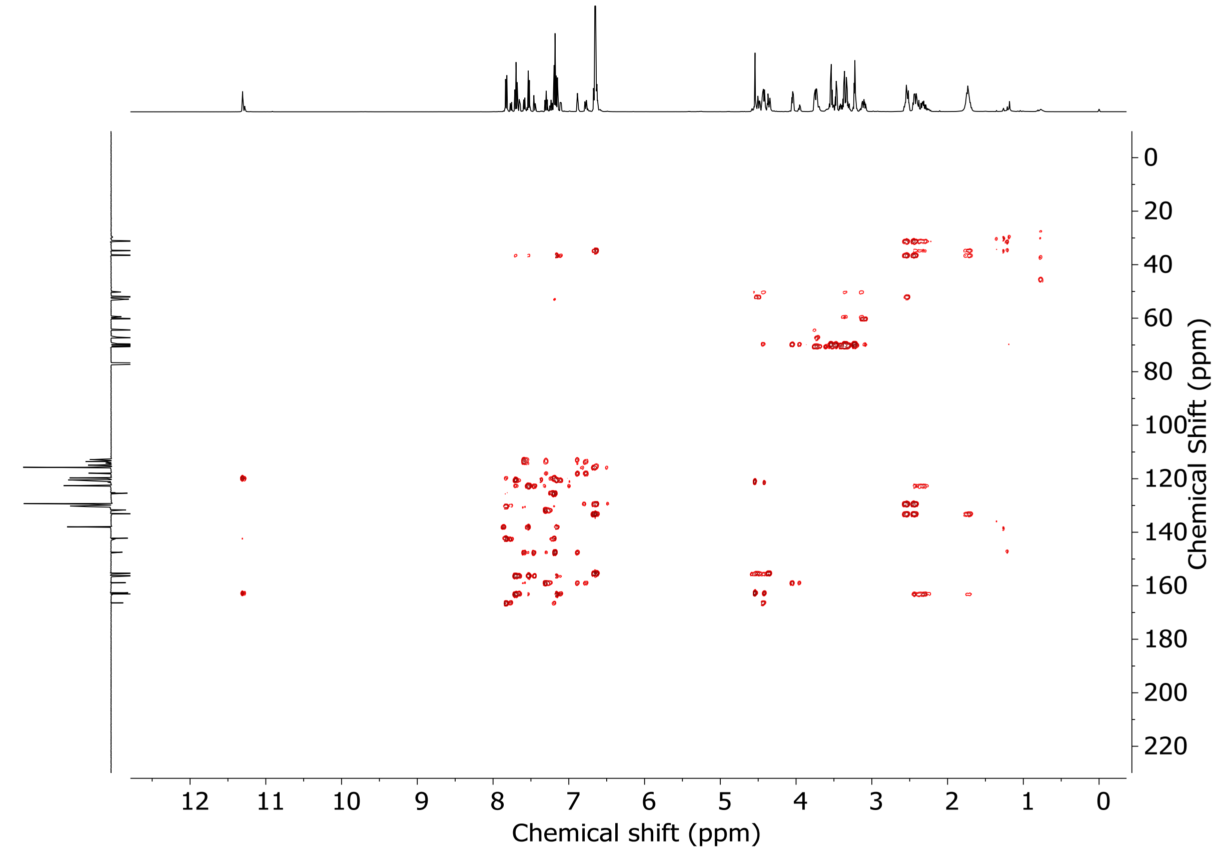

Figure S187: HMBC NMR ($CDCl_3$) of (E_m)-**14** and (Z_m)-**14** (4.9 : 1 *dr*).

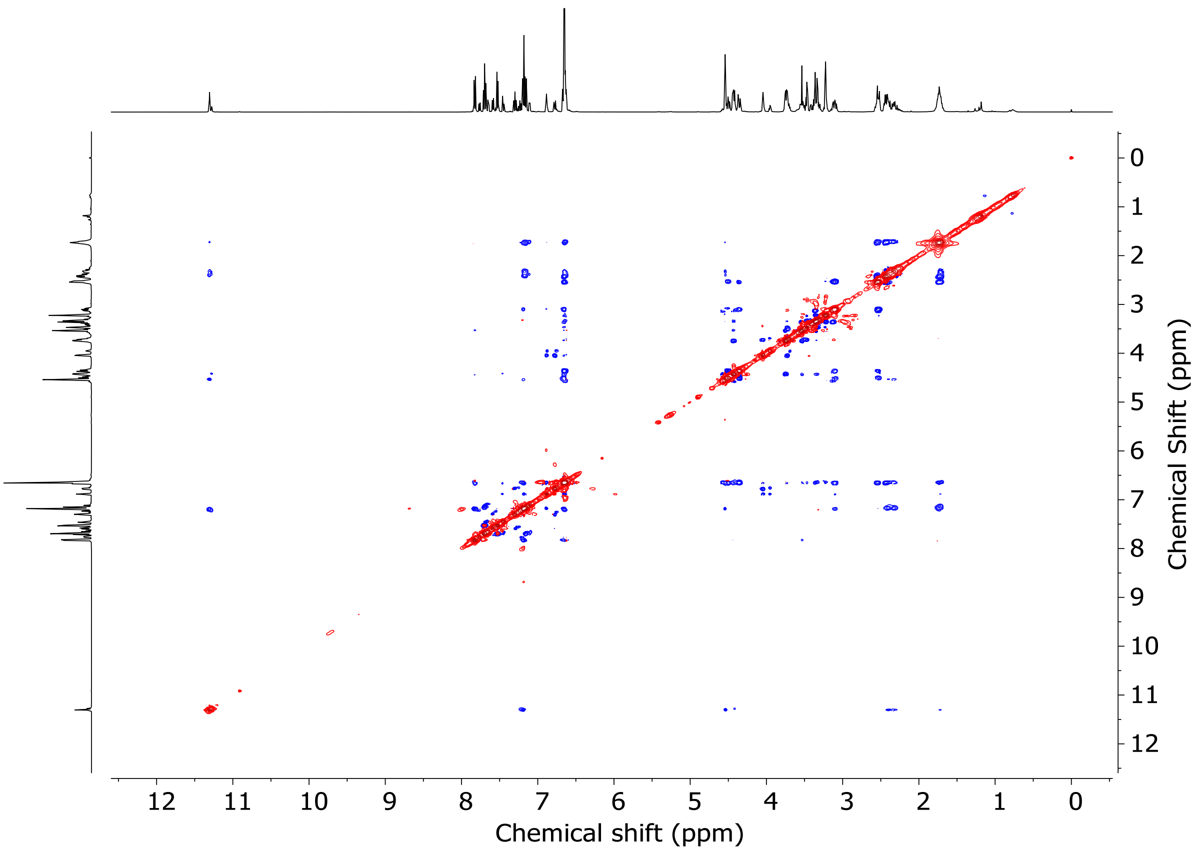

Figure S188: NOESY NMR ($CDCl_3$) of (*E*m)-**14** and (*Z*m)-**14** (4.9 : 1 *dr*).

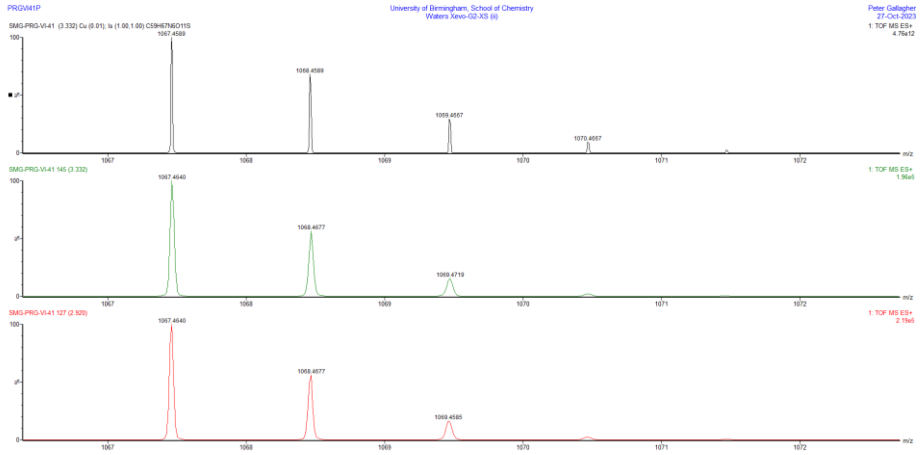

Figure S189: Calculated (top) and observed (middle, bottom) isotopic patterns for (*E*m)-**14** and (*Z*m)-**14.**

6. Rotaxanes 15 and 16, and their precursors (Schemes 5 and Scheme 6, main text)

Amine rotaxane (R_{ma})-15 from (*R*)-1a

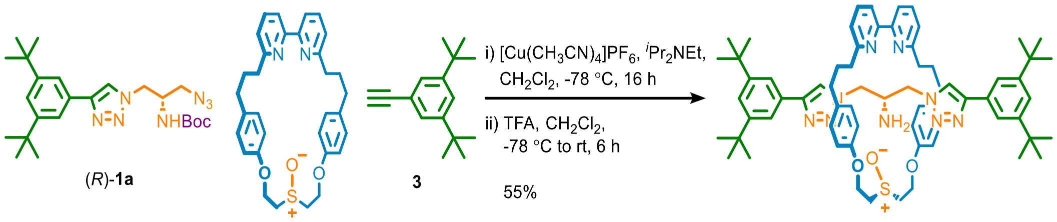

In a CEM vial were added **3** (2.8 mg, 13.1 µmol), (*R*)-**1a** (5.5 mg, 12.1 µmol), **2** (5.8 mg, 11.0 µmol) and [Cu($CH_3CN)_4PF_6$] (3.9 mg, 10.5 µmol). The vial was sealed and purged with N_2, then CH_2Cl_2 was added (250 µL). The solution was cooled to -78 °C, then $^iPr_2NEt$ (4 µL, 23 µmol) was added. The solution was stirred at -78 °C for 16 h. TFA (170 µL, 1 mmol) was added and the resulting mixture was allowed to warm to rt and stirred vigorously for 6h. The crude mixture was diluted with CH_2Cl_2 (5 mL) and carefully poured onto EDTA-NH_3 (5 mL), with separation of aqueous and organic phases. The combined aqueous phase was then extracted with CH_2Cl_2 (3 x 5 mL) and the combined organic extracts were washed with brine (10 mL), dried ($MgSO_4$) and concentrated *in vacuo*. Chromatography (CH_2Cl_2-CH_3CN 0→20% then [(3:1) CH_2Cl_2-CH_3CN]-MeOH 0→10%) gave (R_{ma})-**15** as a white foam (6.7 mg, 55%, 78% *ee* see Figure S191).

All spectroscopic data is consistent with those reported previously.[2]

Amine rotaxane (S_{ma})-**15** (29.9 mg, 68%, 77% *ee*) was synthesised using an identical procedure starting from (*S*)-**1a** (16.9 mg, 44.1 µmol). Analytical data were identical to (R_{ma})-**15** with the exception CSP-HPLC (Figure S191).

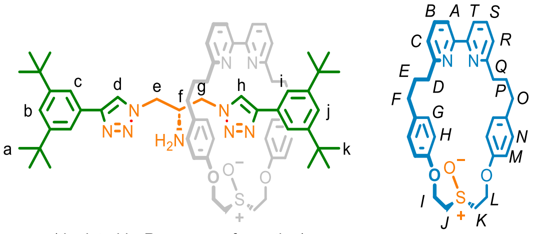

(depicted in $R_{co\text{-}c}$ co-conformation)

^{1}H NMR (500 MHz, $CDCl_3$) δ: 8.58 (s, 1H, H_h), 8.06 (s, 1H, H_d), 7.66 (d, *J* = 1.8 Hz, 2H, H_C), 7.65-7.58 (m, 3H, H_i, H_B), 7.46 (d, *J* = 2.9 Hz, 1H, H_A), 7.44 (d, *J* = 2.9 Hz, 1H, H_A), 7.39 (t, *J* = 1.8 Hz, 1H, H_b), 7.35 (t, *J* = 1.9 Hz, 1H, H_j), 7.12 (d, *J* = 8.0 Hz, 1H, H_C), 7.09 (d, *J* = 7.7 Hz, 1H, H_C), 6.73-6.61 (m, 6H, H_G, H_H), 6.59-6.55 (m, 2H, H_H), 4.73-4.60 (m, 2H, H_I), 4.60-4.45 (m, 2H, H_I), 3.94-3.74 (m, 3H, H_J, H_e), 3.67-3.52 (m, 2H, H_e, H_g), 3.21 (dd, *J* = 13.8, 7.9 Hz, 1H, H_g), 3.17-3.06 (m, 2H, H_J), 2.70-2.62 (m, 1H, H_f), 2.57-2.38 (m, 9H, H_F, H_D), 1.81-1.66 (m, 4H, H_E), (s, 18H, H_a), 1.29 (s, 18H, H_k);

^{13}C NMR (126 MHz, $CDCl_3$) δ: 162.7, 162.7, 157.9, 157.8, 155.8, 155.7, 151.3, 148.3, 148.0, 137.3, 137.2, 134.0, 133.9, 130.4, 130.2, 129.5, 129.4, 122.6, 122.2, 122.1, 122.1, 122.0, 121.3, 120.6, 120.5, 120.3, 120.0, 115.0, 114.9, 61.0, 60.9, 53.7, 53.1, 53.0, 51.5, 37.3, 37.2, 35.1, 35.0, 34.7, 34.7, 31.9, 31.8, 31.7, 31.6.

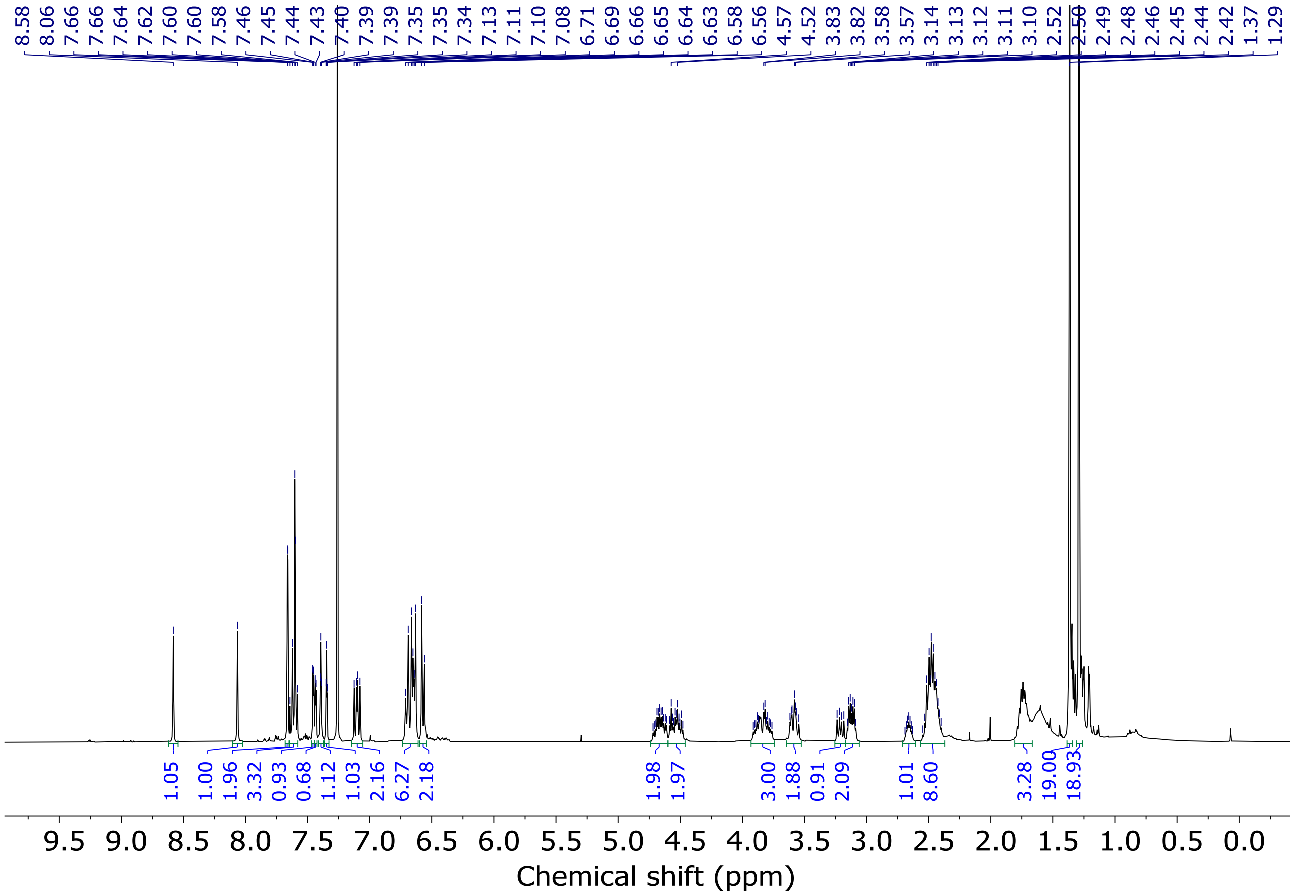

Figure S190. ^{1}H NMR ($CDCl_3$, 400 MHz) of (R_{ma})-**15**.

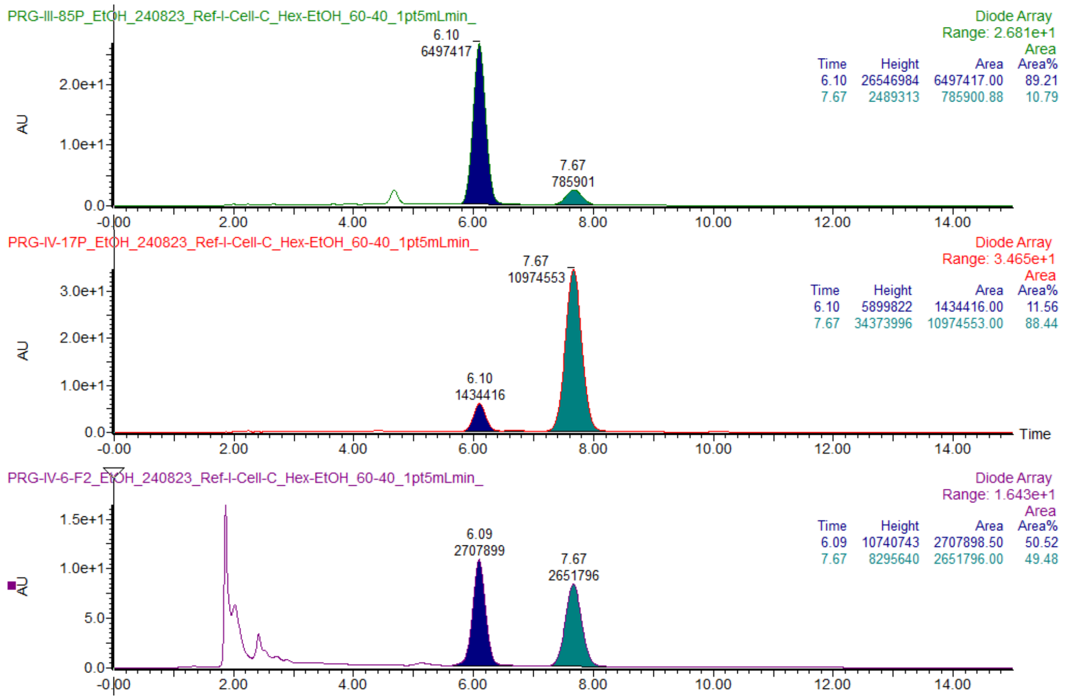

Figure S191. CSP-HPLC of **15** from **1a** (loaded in EtOH). Regis Reflect I-Cellulose C, *n*-hexane-EtOH 60 : 40, flowrate 1.5 mLmin^{-1}. (top) (R_{ma})-**15** from (R)-**1a**; (R_{ma})-**15** (6.10 min, 6497417, 89.21%), (S_{ma})-**15** (7.67 min, 785901, 10.79%). (middle) (S_{ma})-**15** from (*S*)-**1a**; (R_{ma})-**15** (6.10 min, 1434416, 11.56%), (S_{ma})-**15** (7.67 min, 10974553, 88.44%). (bottom) *rac*-**15**; (R_{ma})-**15** (6.09 min, 2707899, 50.52%), (S_{ma})-**15** (7.67 min, 2651796, 49.48%).

Amine rotaxane (R_{ma})-15 from (R)-1f

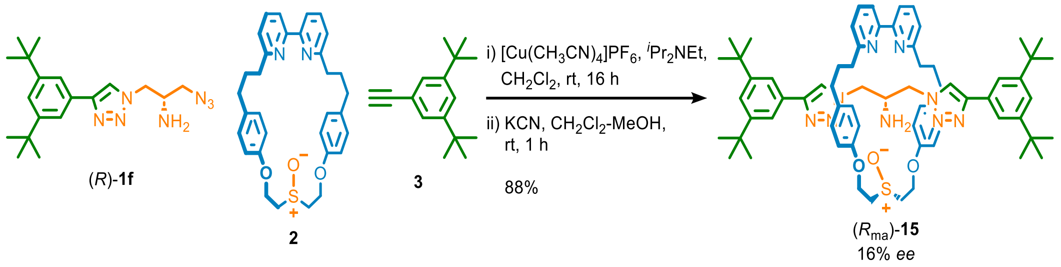

In a CEM vial were added **3** (4.7 mg, 21.9 μmol), (*R*)-**1f** (7.8 mg, 21.9 μmol), **2** (10.5 mg, 20.0 μmol) and [$Cu(CH_3CN)_4PF_6$] (7.1 mg, 19.3 μmol). The vial was sealed and purged with N_2, then CH_2Cl_2 was added (1.0 mL), followed by $^iPr_2NEt$ (7 μL, 40.1 μmol). The solution was stirred at rt for 16 h. MeOH (2 mL mL) and KCN as a solid (13 mg, 0.20 mmol) were added and the resulting mixture was stirred vigorously for until colourless. The crude mixture was diluted with CH_2Cl_2 (5 mL) and washed with H_2O (5 mL) then EDTA-NH_3 (5 mL), with separation of aqueous and organic phases. The combined aqueous phase was then extracted with CH_2Cl_2 (3 x 5 mL) and the combined organic extracts were washed with brine (10 mL), dried ($MgSO_4$) and concentrated *in vacuo*. Chromatography (CH_2Cl_2-CH_3CN 0→20% then [(3:1) CH_2Cl_2-CH_3CN]-MeOH 0→10%) gave (R_{ma})-**15** as a white foam (19.3 mg, 88%, 16.10% *ee* see Figure S192).

All spectroscopic data are identical to those reported for rotaxane (R_{ma})-**5** reported previously.[2]

Amine rotaxane (S_{ma})-**5** (18.1 mg, 83%, 16.70% *ee*) was synthesised using an identical procedure starting from (*S*)-**1f** (7.7 mg, 21.7 μmol). Analytical data were identical to (R_{ma})-**5** with the exception CSP-HPLC (Figure S192).

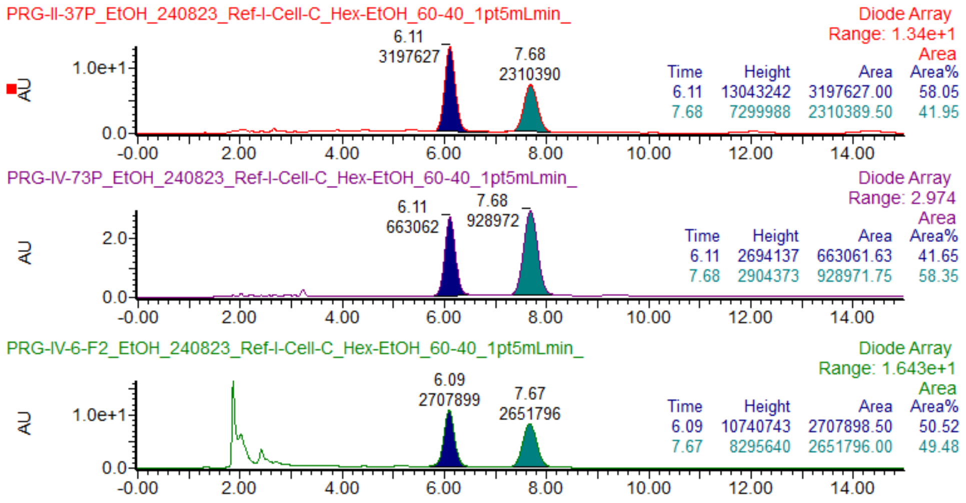

Figure S192. CSP-HPLC of **15** from **1f** (loaded in EtOH). Regis Reflect I-Cellulose C, *n*-hexane-EtOH 60 : 40, flowrate 1.5 mLmin^{-1}. (top) (R_{ma})-**15** from (*R*)-**1f**; (R_{ma})-**15** (6.11 min, 3197627, 58.05%), (S_{ma})-**15** (7.68 min, 2310390, 41.95%). (middle) (S_{ma})-**15** from (*S*)-**1f**; (R_{ma})-**15** (6.11 min, 663062, 41.65%), (S_{ma})-**15** (7.68 min, 928972, 58.35%). (bottom) *rac*-**15**; (R_{ma})-**15** (6.09 min, 2707899, 50.52%), (S_{ma})-**15** (7.67 min, 2651796, 49.48%).

Formamide azide (*R*)-1g

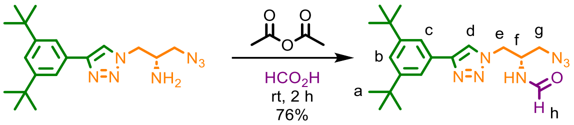

To a solution of (*R*)-**1f** (30.0 mg, 0.084 mmol) in HCO_2H (800 µL) was added acetic anhydride (63.8 µL, 0.68 mmol). The solution was stirred at rt for 2 h, then the solution was diluted with CH_2Cl_2 (5 mL) and H_2O (2 mL) was added. The aqueous and organic phases were separated, and the aqueous phase was then extracted with CH_2Cl_2 (3 x 10 mL). The combined organic extracts were washed with brine (15 mL), dried ($MgSO_4$) and concentrated *in vacuo*. Chromatography (petrol-EtOAc 0→50%) gave (*R*)-**1g** (21.4 mg, 76%) as a white solid.

Formamide azide (*S*)-**1g** (17.4 mg, 82%) was synthesised using an identical procedure starting from (*S*)-**1f** (21.1 mg, 0.055 mmol). Analytical data was identical to (R)-**1g** with the exeption of their CD spectra (Figure S198) and CSP-HPLC (Figure S199).

^{1}H NMR (400 MHz, CDCl$_3$) [note: the labelling scheme used here differs from that in Scheme 6; see main text and associated footnote] δ: 8.20 (s, 1H, H_h), 7.85 (s, 1H, H_d), 7.65 (d, *J* = 1.8, 2H, H_c), 7.44 (t, *J* = 1.8, 1H, H_b), 6.63 (d, *J* = 7.7, 1H, NH), 4.71-4.51 (m, 3H, H_e, H_f), 3.62 (dd, *J* = 12.5, 4.8 Hz, 1H H_g), 3.32 (dd, *J* = 12.5, 6.7 Hz, 1H $H_{g'}$), 1.36 (s, 18H H_a);

^{13}C NMR (100 MHz, CDCl$_3$) δ: 161.3, 151.6, 149.0, 129.1, 122.9, 121.1, 120.2, 50.6, 49.9, 47.6, 35.0, 31.4

HR-ESI-MS (+ve) m/z = 384.2513 $[M+H]^+$ (calc. 384.2506 m/z for $C_{20}H_{30}N_7O$);

Melting point = 152-154 °C.

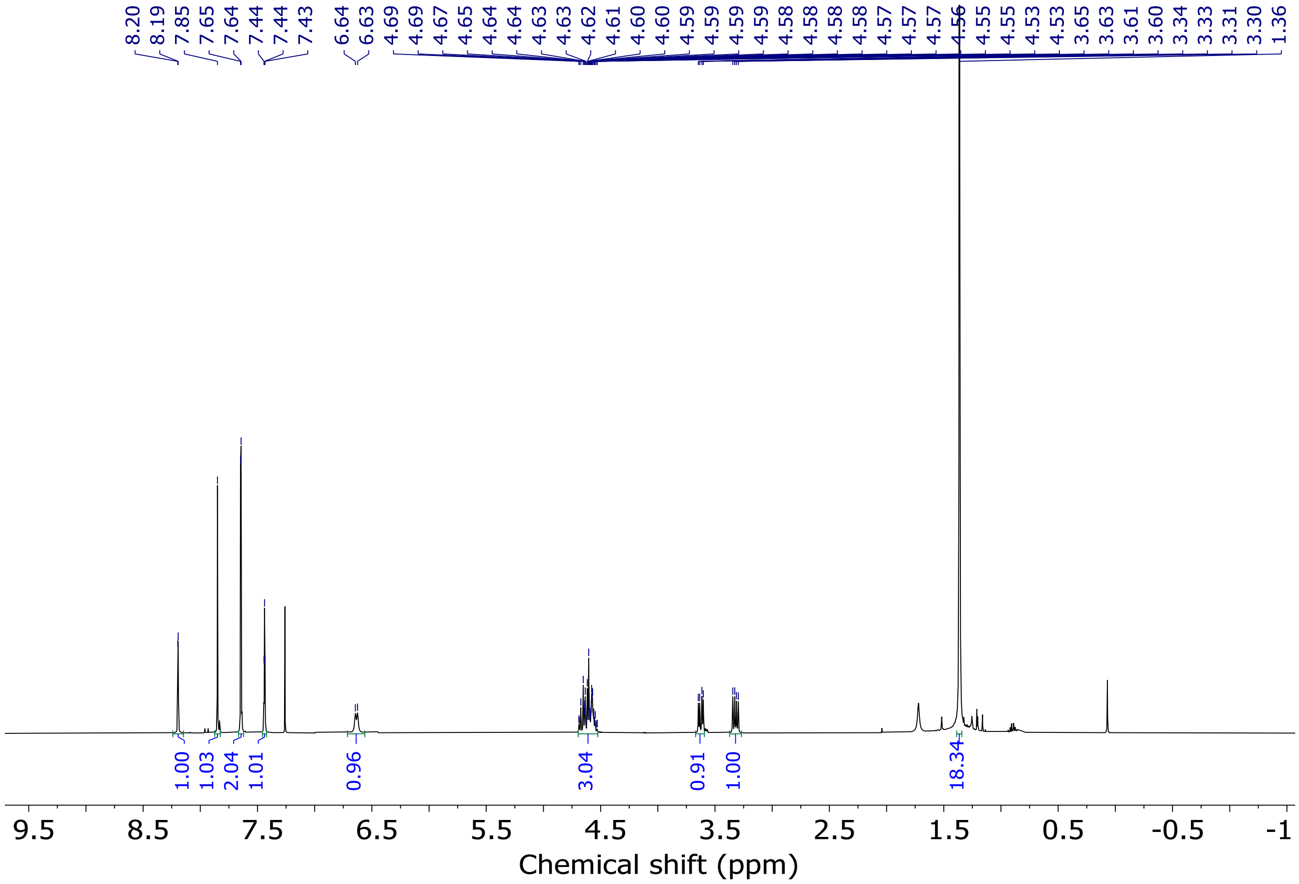

Figure S193. ^{1}H NMR ($CDCl_3$, 400 MHz) of (*R*)-**1g**.

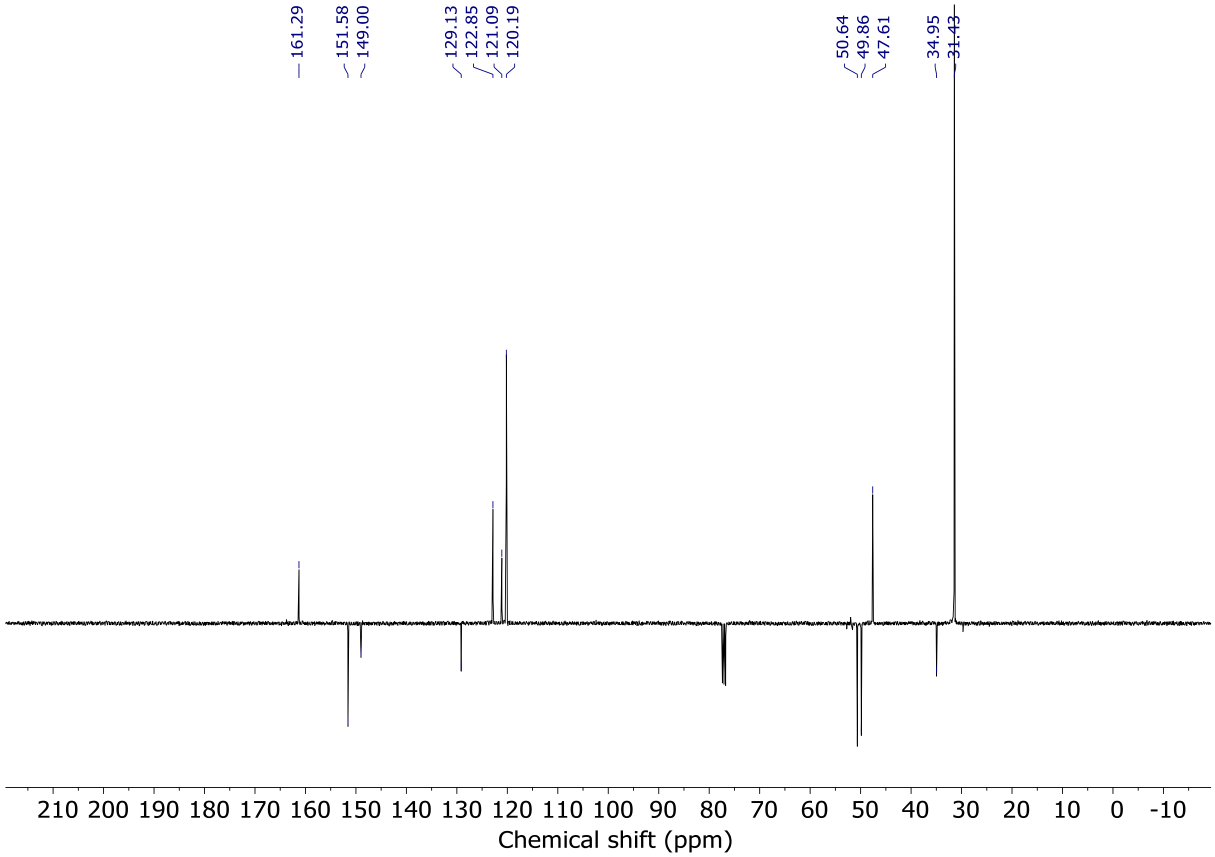

Figure S194. JMOD NMR ($CDCl_3$, 101 MHz) of (*R*)-**1g**.

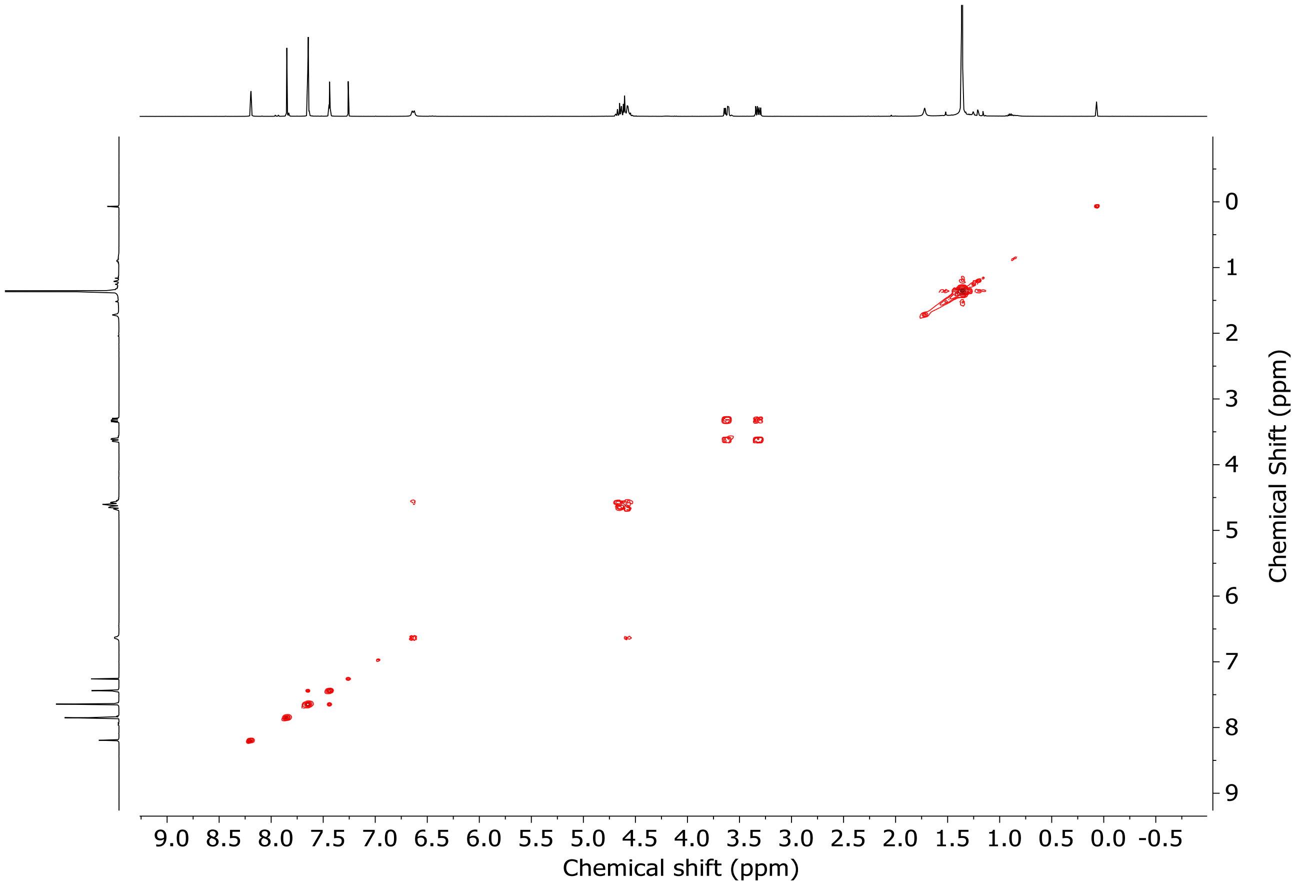

Figure S195. COSY NMR ($CDCl_3$) of (*R*)-**1g**.

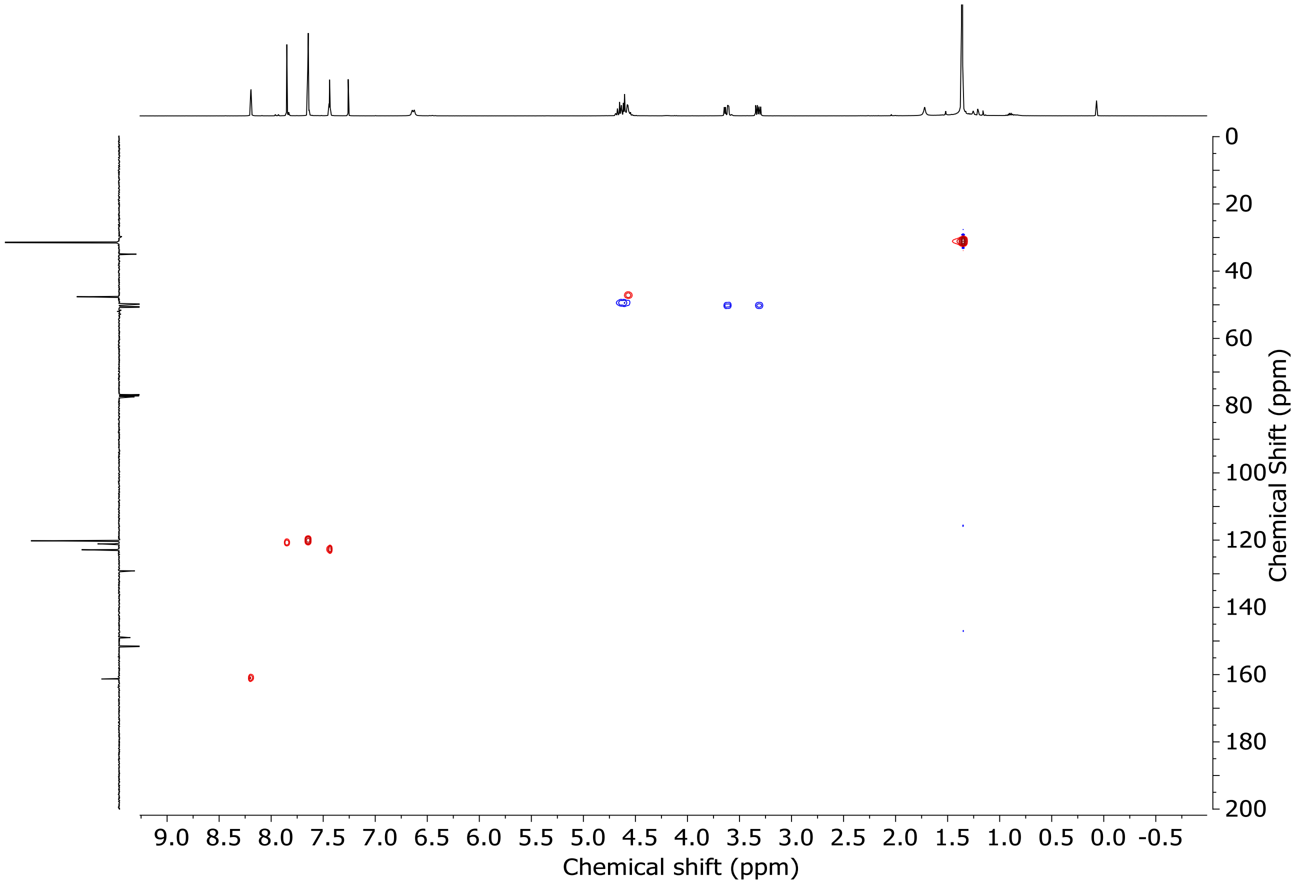

Figure S196. HSQC NMR ($CDCl_3$) of (*R*)-**1g**.

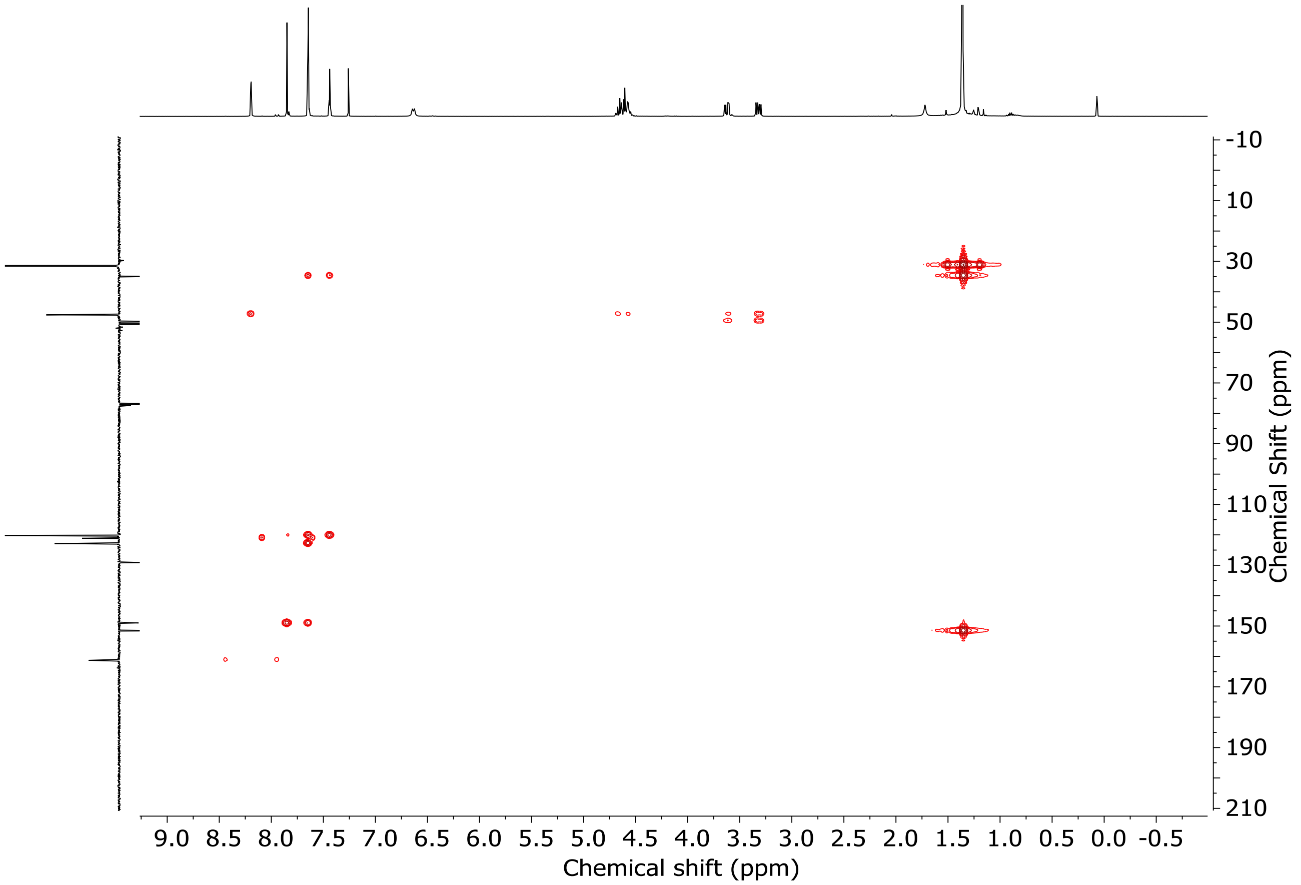

Figure S197. HMBC NMR ($CDCl_3$) of (*R)*-**1g.**

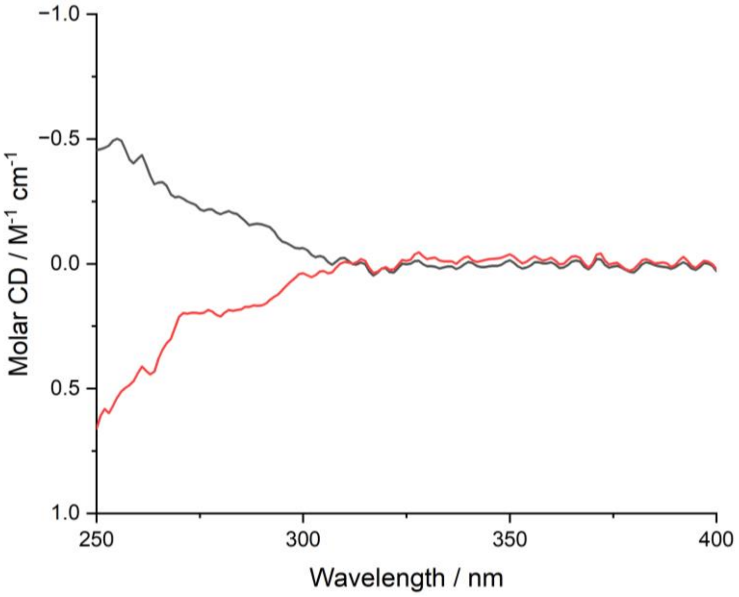

Figure S198. Circular Dichroism Spectra of (*S*)-**1f** (64 µM, grey) and (*R*)-**1f** (82 µM, red) at 293 K in $CHCl_3$.

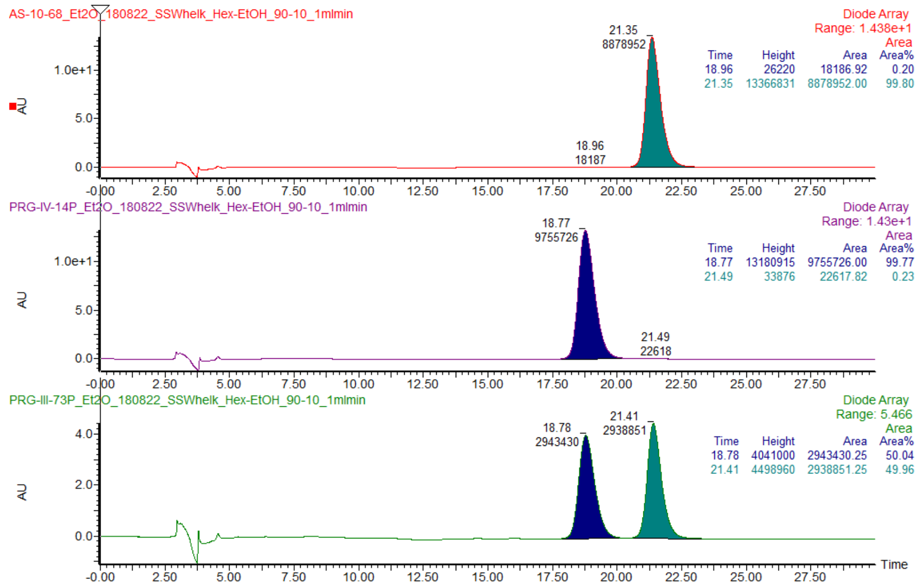

Figure S199. CSP-HPLC of **1g** (loaded in Et_2O). SSWhelk, *n*-hexane-EtOH 90 : 10, flowrate 1 $mLmin^{-1}$. (top) (R)-**1g**; (*S*)-**1g** (18.96 min, 18187, 0.20%), (R)-**1g** (21.49 min, 8878952, 99.80%). (middle) (*S*)-**1g**; (*S*)-**1g** (18.77 min, 9755726, 99.77%), (*S*)-**1g** (21.49 min, 22618, 0.23%). (bottom) *rac*-**1g**, (*S*)-**1g** (18.78 min, 2943430, 50.04%), (R)-**1f** (10.35 min, 2938851, 49.96%).

Formamide rotaxane (R_{ma})-16

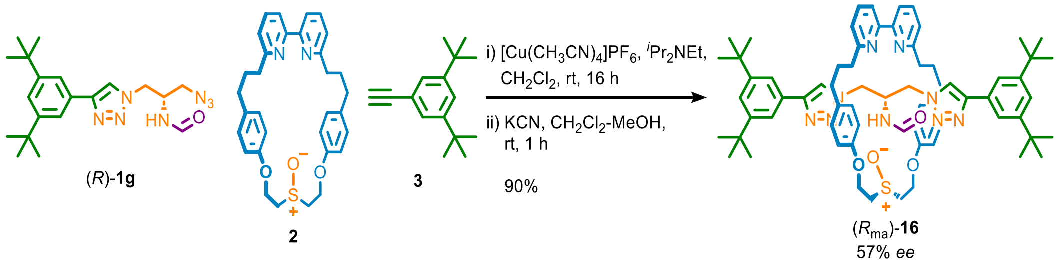

In a CEM vial were added **3** (4.7 mg, 21.9 μmol), (*R*)-**1g** (8.4 mg, 21.9 μmol), **2** (10.5 mg, 20.0 μmol) and [$Cu(CH_3CN)_4PF_6$] (7.1 mg, 19.0 μmol). The vial was sealed and purged with N_2, then CH_2Cl_2 was added (500 μL), followed by $^{i}Pr_2NEt$ (7 μL, 40.1 μmol). The solution was stirred at rt for 16 h. MeOH (2 mL mL) and KCN as a solid (13 mg, 0.20 mmol) were added and the resulting mixture was stirred vigorously for until colourless. The crude mixture was diluted with CH_2Cl_2 (5 mL) and washed with H_2O (5 mL) then EDTA-NH_3 (5 mL), with separation of aqueous and organic phases. The combined aqueous phase was then extracted with CH_2Cl_2 (3 x 5 mL) and the combined organic extracts were washed with brine (10 mL), dried ($MgSO_4$) and concentrated *in vacuo*. Chromatography (CH_2Cl_2-CH_3CN 0→100%) gave (R_{ma})-**16** as a white foam (20.6 mg, 90%, 57% *ee* see Figure S207).

Formamide rotaxane (S_{ma})-**16** (29.9 mg, 68%, 61.84% *ee*) was synthesised using an identical procedure starting from (*S*)-**1g** (16.9 mg, 44.1 μmol). Analytical data were identical to (R_{ma})-**16** with the exception CSP-HPLC (Figure S207).

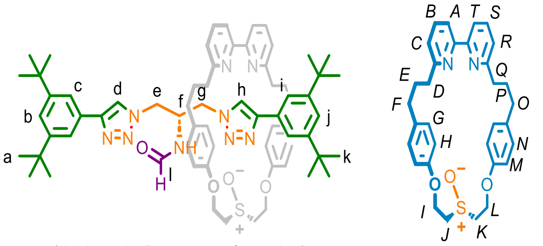

(depicted in $R_{co\text{-}c}$ co-conformation)

^{1}H NMR (500 MHz, CD_2Cl_2) [note: the labelling scheme used here differs from that in Scheme 6; see main text and associated footnote] δ: 9.08 (bs, 1H, H_h), 8.21 (bs, 1H, H_d), 7.68 (t, J = 7.8, 1H, H_B), 7.63 (t, J = 7.6, 1H, H_S), 7.60-7.53 (m, 5H, H_c, H_i, NH), 7.47-7.39 (m, 2H, H_A, H_T), 7.38 (t, J = 1.9, 1H, H_b or H_j), 7.32 (t, J = 1.9, 1H, H_b or H_j), 7.19 (dd, J = 7.8, 1.0, 1H, H_C), 7.13-7.08 (m, 2H, H_l, H_R), 6.78 (d, J = 8.6, 2H, H_G or H_N), 6.75-6.67 (m, 4H, H_G and H_H or H_M and H_N), 6.60 (d, J = 8.5, 2H, H_H or H_M), 4.69-4.55 (m, 2H, H_I and $H_{I'}$ or H_L and $H_{L'}$), 4.52 (dd, J = 12.2, 9.2, 3.6, 1H, H_I or H_L), 4.43 (dt, J = 12.0, 4.7, 1H, $H_{I'}$ or $H_{L'}$), 4.17-4.08 (m, 1H, H_f), 3.98 (dd, J = 14.6, 3.4, 1H, H_e), 3.92-3.65 (m, 4H, $H_{e'}$, H_g, H_J or H_K), 3.61 (ddd, J = 13.8, 9.3, 4.4, 1H, H_J or H_K), 3.21-3.05 (m, 2H, $H_{J'}$, $H_{K'}$), 2.67-2.38 (m, 6H, H_F, H_O, H_D or H_Q), 2.37-2.20 (m, 2H, H_D or H_Q), 1.95-1.67 (m, 4H, H_E, H_P, superimposed with residual H_2O), 1.33 (s, 18H, H_a), 1.26 (s, 18H, H_k)

^{13}C NMR (126 MHz, CD_2Cl_2) δ: 163.0, 162.8, 161.3, 157.4, 157.3, 156.2, 151.3, 151.1, 148.1, 148.0, 137.5, 137.4, 133.8, 133.6, 130.5, 130.2, 129.6, 129.5, 122.9, 122.3, 122.1, 121.8, 121.8, 121.4, 120.8, 120.7, 120.0, 119.8, 115.1, 114.6, 61.4, 60.7, 53.0, 50.1, 49.9, 48.7, 37.0, 36.6, 34.9, 34.8, 34.8, 31.9, 31.3, 31.3, 31.3, 30.2

LR-ESI-MS (+ve) m/z = 1124.7 $[M+H]^+$ (calc. m/z for $C_{68}H_{85}N_9O_4S$ 1124.7);

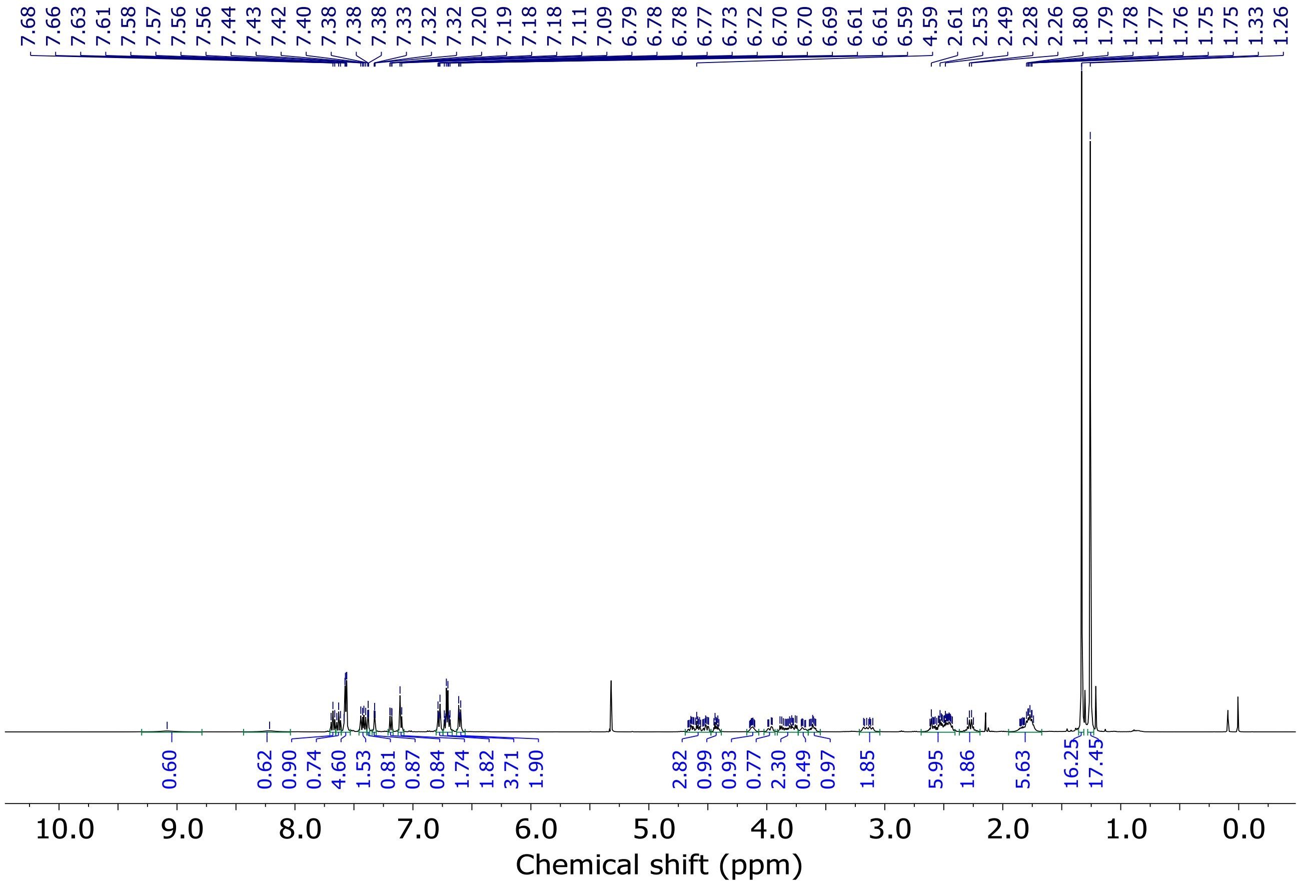

Figure S200. ^{1}H NMR (CD_2Cl_2, 500 MHz) of (R_{ma})-**16**.

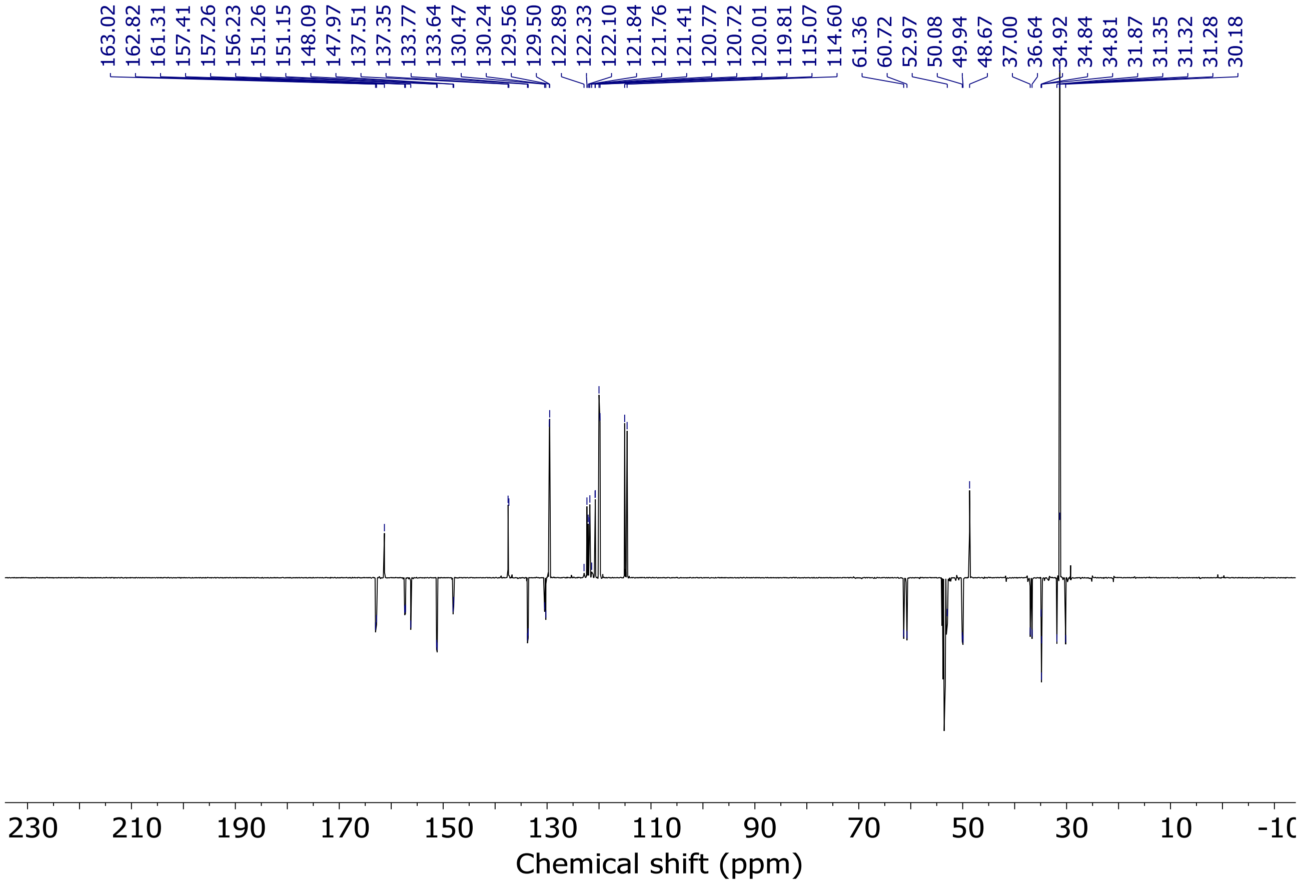

Figure S201. JMOD NMR (CD_2Cl_2, 126 MHz) of (R_{ma})-**16**.

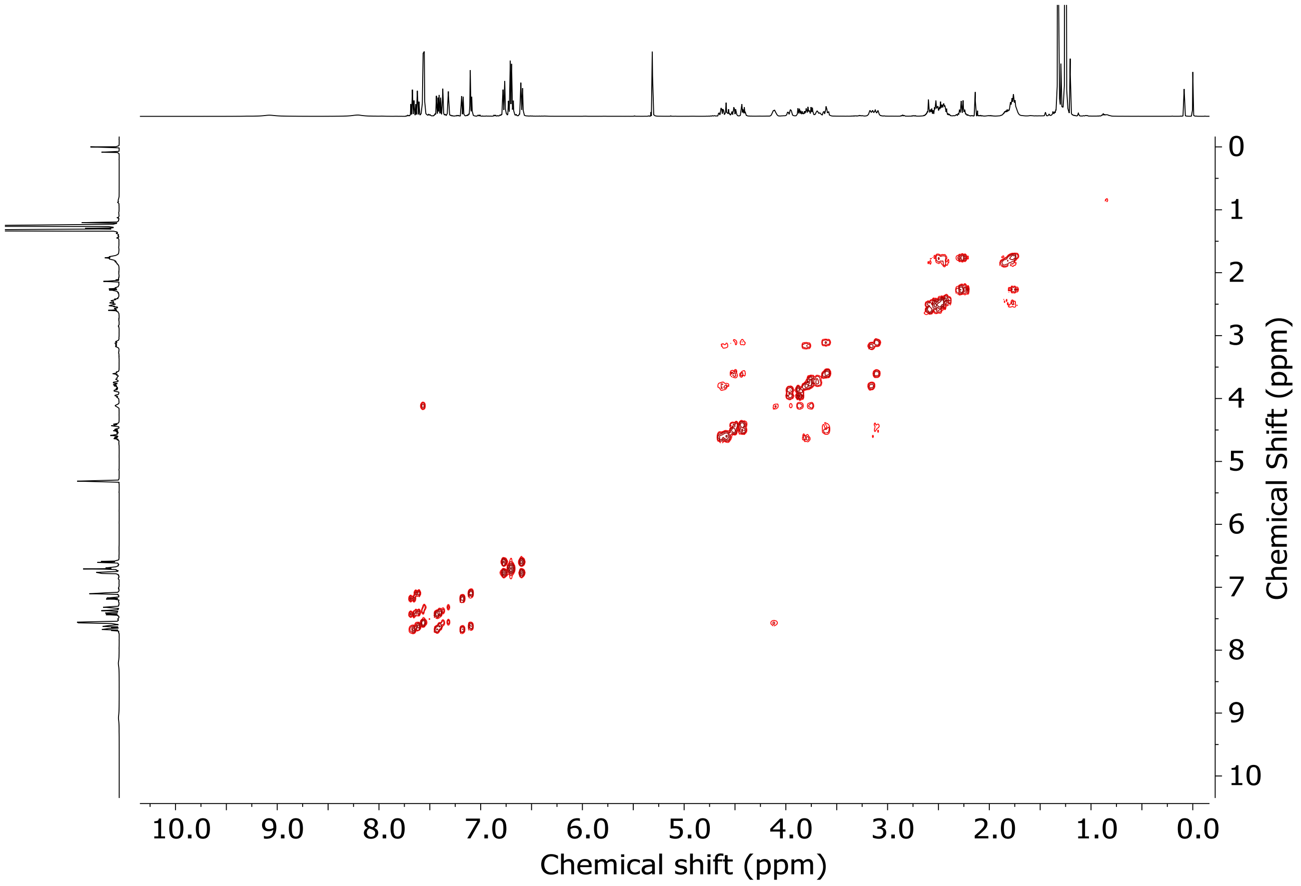

Figure S202. COSY NMR (CD_2Cl_2) of (R_{ma})-**16**.

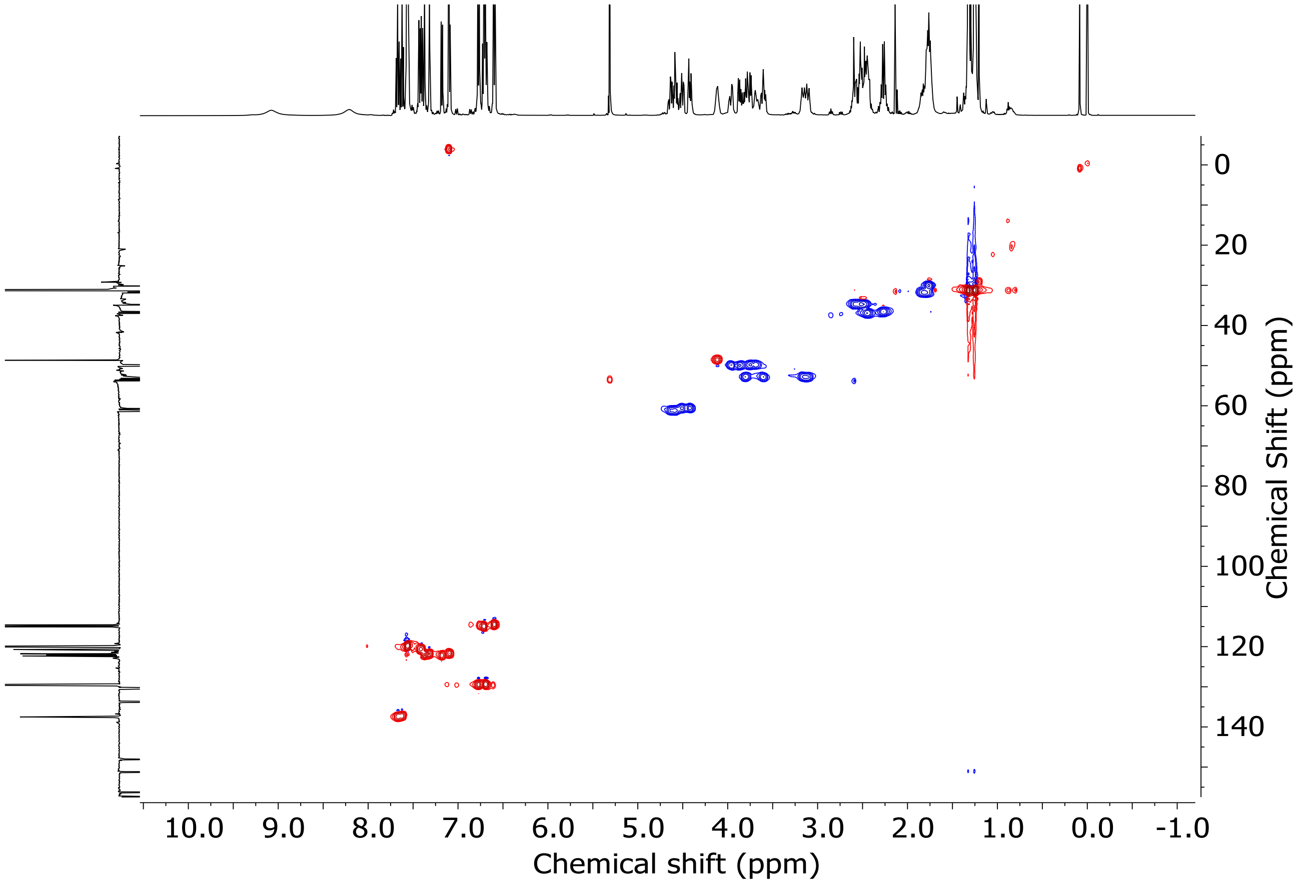

Figure S203. HSQC NMR (CD_2Cl_2) of (R_{ma})-**16**.

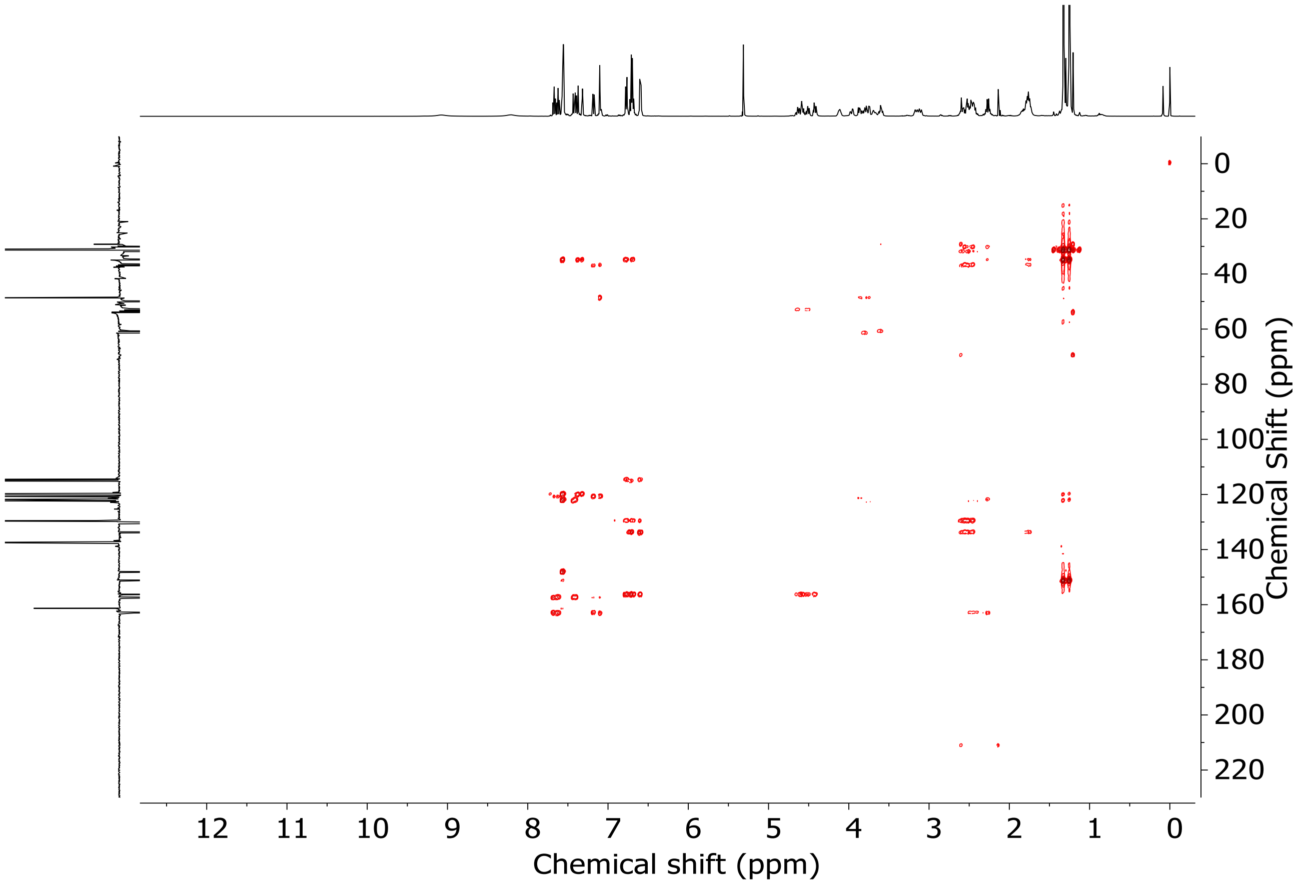

Figure S204. HMBC NMR (CD_2Cl_2) of (R_{ma})-**16**.

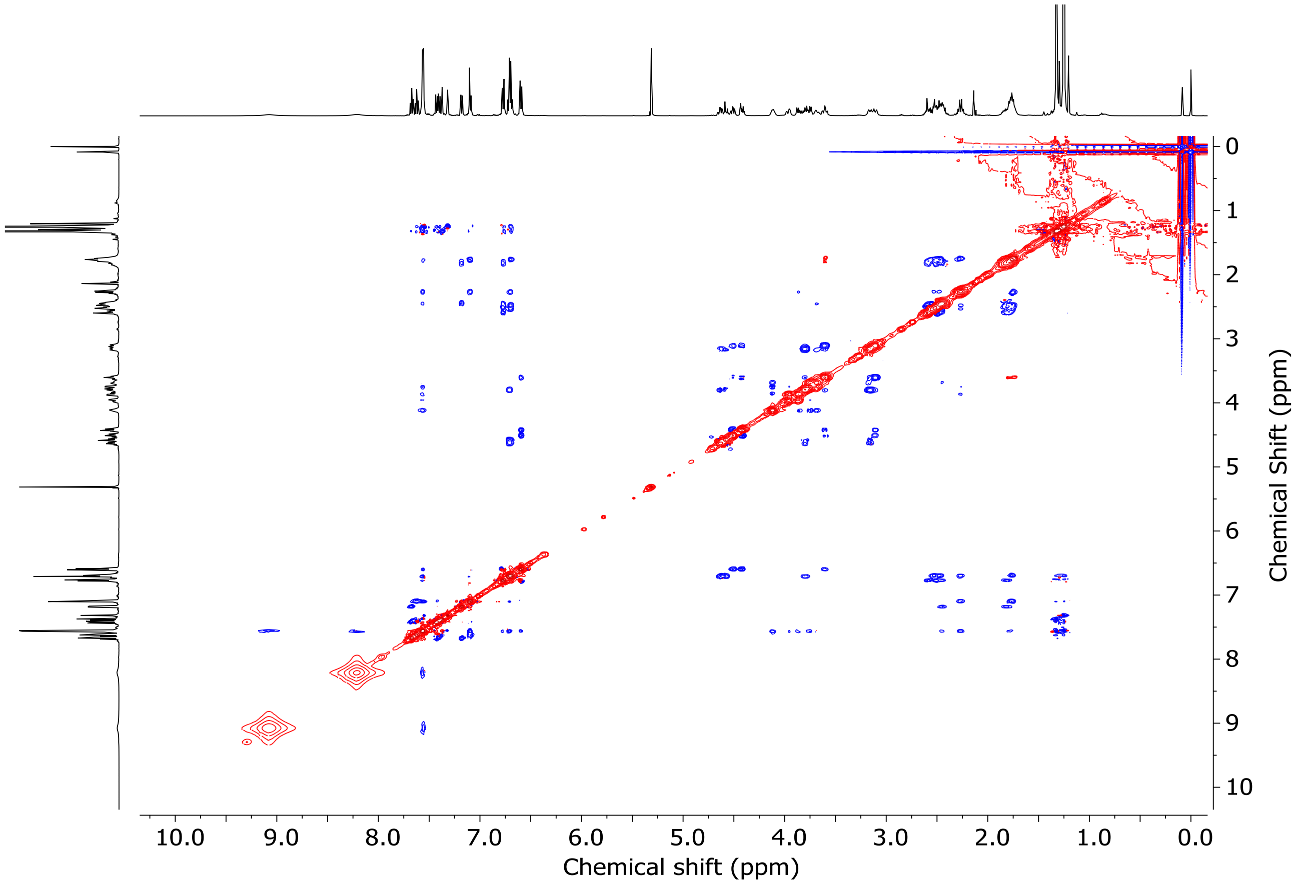

Figure S205. NOESY NMR (CD_2Cl_2) of (R_{ma})-**16**.

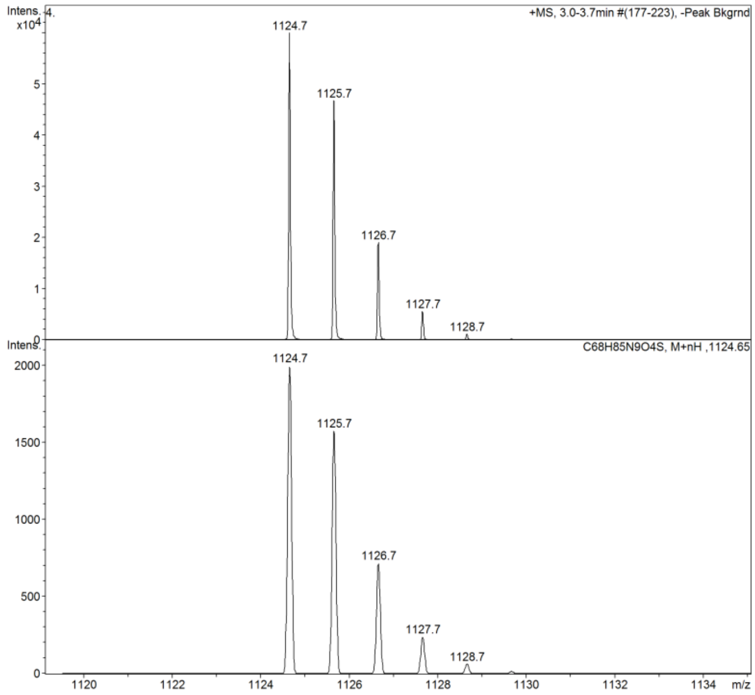

Figure S206. Observed (top) and calculated (bottom) isotopic patterns for rotaxanes **16**.

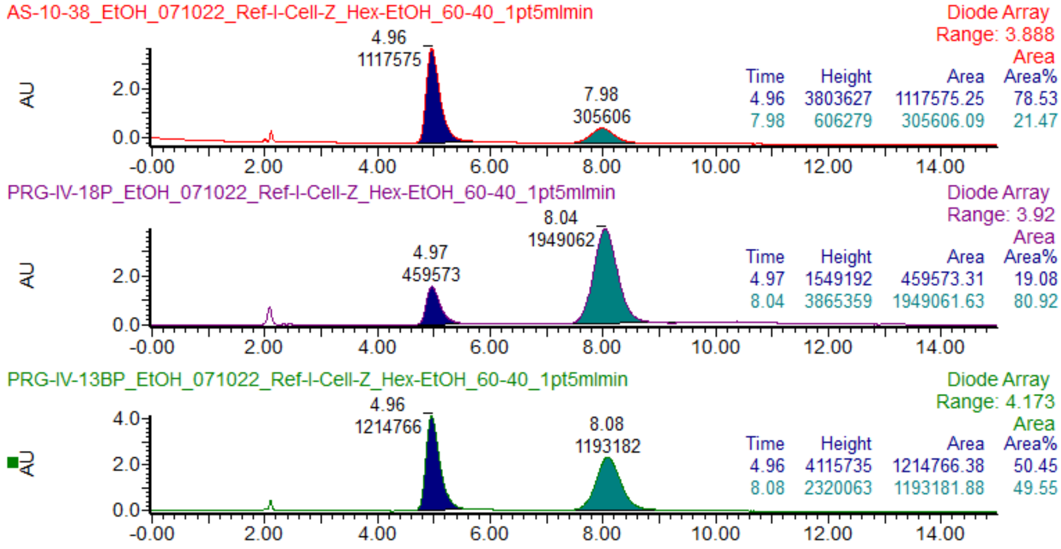

Figure S207. CSP-HPLC of **16** (loaded in EtOH). Regis Reflect I-Cellulose Z, *n*-hexane-EtOH 60 : 40, flowrate 1.5 mLmin^{-1}. (top) (R_{ma})-**16** from (*R*)-**1g**; (R_{ma})-**6** (4.96 min, 1117575, 78.53%), (S_{ma})-**16** (7.98 min, 305606, 21.47%). (middle) (S_{ma})-**16** from (*S*)-**1g**; (R_{ma})-**16** (4.97 min, 459573, 19.08%), (S_{ma})-**16** (8.04 min, 1949062, 80.92%). (bottom) *rac*-**16**, (R_{ma})-**16** (4.96 min, 1214766, 50.45%), (S_{ma})-**16** (8.08 min, 1193182, 49.55%).

7. Determination of the major stereoisomer of rotaxane 16 produced from 1g

In order to determine the absolute stereochemistry of major stereoisomer of rotaxane **16** produced directly from **1g**, an analytical sample of rotaxane (R_{ma})-**16** was prepared formylation of a sample amine rotaxane (R_{ma})-**15** of known stereopurity (21% *ee*), as described below, which allowed us to assign the retention time of the enantiomers of **16**.

Rotaxanes 16 *via* formylation of rotaxanes 15

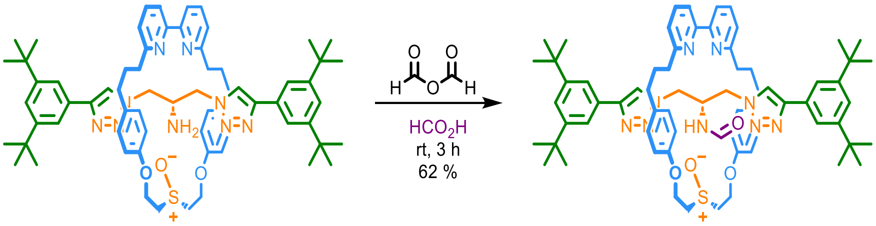

To a solution of (R_{ma})-**15** (3.5 mg, 3.2 µmol, 21% *ee*, Figure S208) in HCO_2H (250 µL) was added acetic anhydride (18.0 µL, 19.0 µmol). The solution was stirred at rt for 3 h, then the solution was diluted with CH_2Cl_2 (5 mL) and H_2O (2 mL) was added. The aqueous and organic phases were separated, and the aqueous phase was then extracted with CH_2Cl_2 (3 x 10 mL). The combined organic extracts were washed with brine (15 mL), dried ($MgSO_4$) and concentrated *in vacuo*. Chromatography (CH_2Cl_2-CH_3CN 0→100%) gave (R_{ma})-**16** (2.2 mg, 62%, +21% *ee*, Figure S208) as a white foam. All spectroscopic data were identical to those reported for rotaxane (R_{ma})-**16** above.

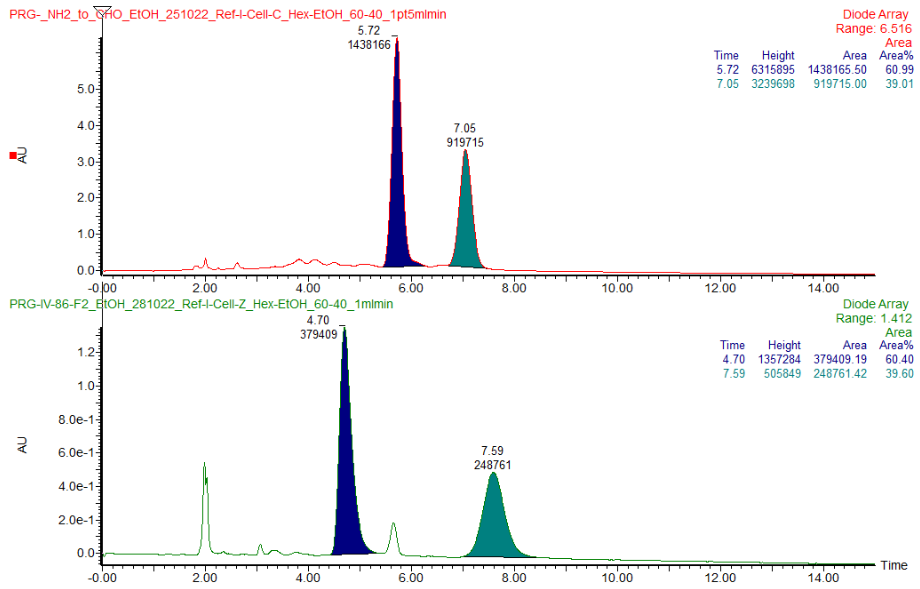

Figure S208. CSP-HPLC to determine the absolute stereochemistry of rotaxanes **16**. (top) amine rotaxane (R_{ma})-**15** starting material (+21.98% *ee*). (bottom) formamide rotaxane (R_{ma})-**16** product (+20.80% *ee*).

8. Absolute stereochemical assignment of interlocked compounds

Methods to assign the absolute stereochemistry of interlocked molecules are still in development. However, we have previously proposed methods for the assignment of absolute stereochemistry in mechanically planar chiral catenanes and rotaxanes, which are based on oriented covalent sub-units[6] and mechanically axially chiral catenanes and rotaxanes, which are based on facially dissymmetric components.[2] In all cases we make use of Cahn-Ingold-Prelog (CIP)-derived atom priorities to unambiguously assign vectors associated the bilateral dissymmetry of the covalent sub-components. Here we extend these rules to mechanical geometric isomers. We also propose a revision to the method for assigning the MAC stereogenic unit of rotaxanes and catenanes. In all cases, the stereochemical assignment is achieved by considering the relative orientation of the vectors associated with the individual components. Those vectors can be identified by a step-by-step approach based on the rules outlined below.

8.1. A note on the assignment of the MAC stereogenic unit – the need for revision

Recently, we proposed a method to assign the absolute stereochemistry of interlocked molecules with mechanical axial stereochemistry.[2] Our original method to assign mechanically axially chiral (MAC) catenanes was based on a previous proposal by Stoddart and Bruns.[6] A particular challenge with assigning the absolute stereochemistry of MAC rotaxanes is that the outcome depends on the relative orientation of the axle within the cavity of the ring (c.f., deciding which orientation to observe a covalent stereocentre to assign the stereolabel). This is obviously an arbitrary decision and originally, we defined the orientation such that the MAC catenane and rotaxane stereogenic units obtained through the notional ring opening of the former to generate the latter were assigned the same stereolabel (i.e., R_{ma} catenane generates R_{ma} rotaxane). However, when the same arbitrary axle orientation convention was applied to a recently identified non-canonical mechanical geometric stereogenic unit, we found that this created a contradiction; the type II mechanical geometric stereogenic unit of a rotaxane generated by ring opening of the corresponding catenane geometric isomer was assigned the opposite stereolabel (i.e., Z_m catenane generates E_m rotaxane).[7]

In light of this, here we propose a revised method for the assignment of absolute stereochemistry of the MAC stereogenic unit of catenanes and rotaxanes that still results in the same stereolabel if a notional ring opening of the former to generate the latter is followed. Importantly, this method allows the same arbitrary axle orientation to be applied in both MAC and MGI rotaxanes and catenanes, with the same rules that have already been established for the assignment of mechanical planar stereochemistry.[5]

The revision changes the position from which the vectors associated with the facial dissymmetry is viewed and thus effectively inverts the stereolabels of all previously assigned MAC rotaxanes and catenanes.

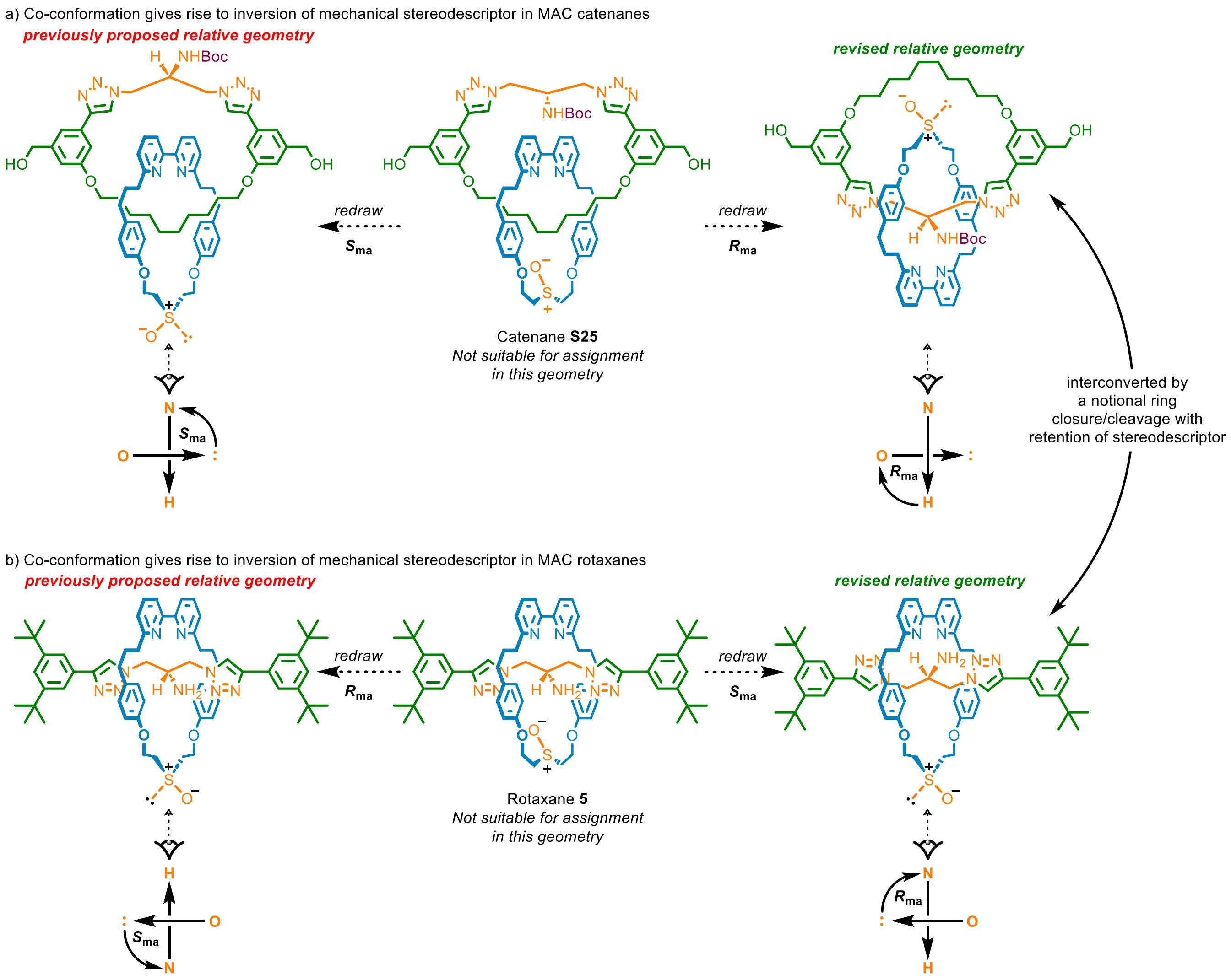

8.2. Revised rules for the assignment of mechanically axially chiral catenanes

To assign the absolute stereochemistry of mechanically axially chiral catenanes it is necessary to consider the relative orientation of the vectors associated with the individual components. These vectors can be identified by a step-by-step approach based on the following rules:

Step 1: In each ring, identify the highest priority prochiral group (or stereogenic unit in meso structures) using the Cahn-Ingold-Prelog priority of the central atom. Redraw the rings such that the in-plane substituents of the prochiral centers point into the center of the respective macrocycle.

Step 2: In each ring, identify the highest priority ligand of the selected center that lie outside of the macrocycle plane, again using CIP priority, and label it as "**A**"; label the lower priority group "**B**".

Step 3: Redraw the molecule such that the prochiral group of each ring lies within the cavity of the other.

Step 4: View the relative orientation of the **A**→**B** vectors at the crossing point of the two rings and consider the direction of rotation from the head of the front vector to the tail of the rear vector. A right-handed path assigned as (R_{ma}) and a left-handed path assigned as (S_{ma}).

Worked example - catenane S25

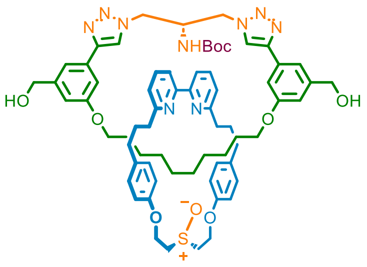

(R_{ma})-**S25**

Step 1: The highest CIP prochiral centers in each component are the carbon bearing the substituted amine in the axle and the sulfoxide sulfur in the macrocycle. Currently the in plane substituents of the prochiral units point out of the cavity. Redraw the structure such that these point into the center of the ring.

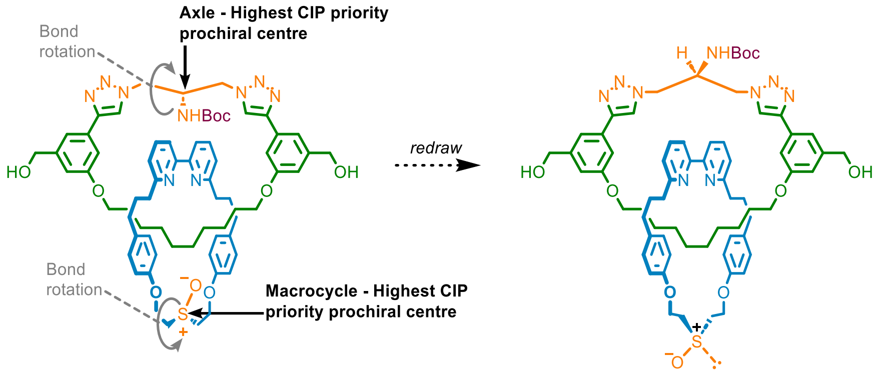

Step 2: The highest priority exocyclic atom bonded to the sulfoxide according to CIP rules is the oxygen, which is labelled as "**A**", while the lone pair is labelled as "**B**". The highest priority exocyclic atom bonded to the prochiral carbon of the triazole-containing macrocycle according to CIP rules is the amine nitrogen, which is labelled as "**A**", while the proton is labelled as "**B**".

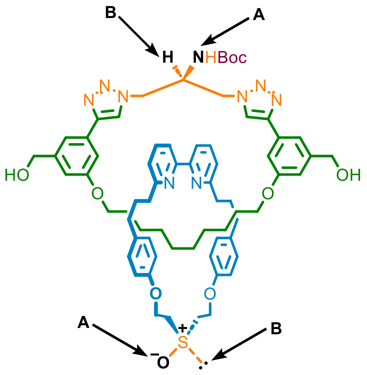

Step 3: Redraw the structure such that the prochiral centre of each ring lies within the cavity of the other.

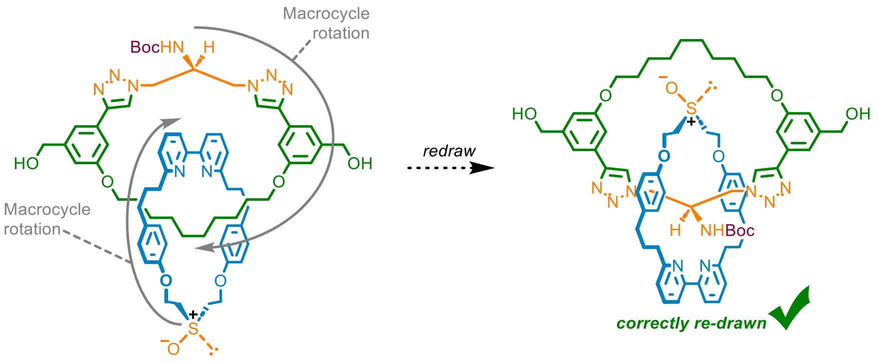

Step 4: View the relative orientation of the **A→B** vectors at the crossing point between the rings. As a clockwise path is taken to the head of the vector defined by the sulfoxide, the stereoisomer of catenane **S25** shown is labelled as (R_{ma})-**S25.**

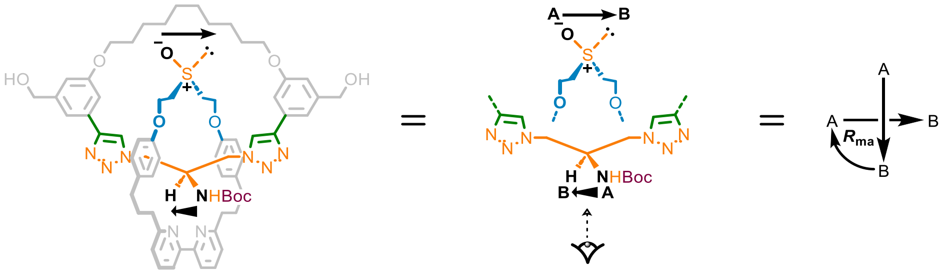

8.3. Revised rules for the assignment of mechanically axially chiral rotaxanes

To assign the absolute stereochemistry of mechanically axially chiral rotaxanes it is necessary to consider the relative orientation of the vectors associated with the individual components. These vectors can be identified by a step-by-step approach based on the following rules:

Step 1: In the ring, identify the highest priority prochiral group (or stereogenic unit in meso structures) using the Cahn-Ingold-Prelog priority of the central atom. Redraw the ring such that the in-plane substituents of the prochiral center points into the center of the macrocycle.

Step 2: Identify the highest priority ligand of the selected center that lies outside of the macrocycle plane, again using CIP priority, and label it as "**A**"; label the lower priority group "**B**".

Step 3: In the axle, identify the highest priority prochiral group (or stereogenic unit in *meso* structures) using the CIP priority of the central atom. Identify the highest priority out of plane ligand of the identified center, again using CIP priority, and label it as "**C**"; label the lower priority group "**D**".

Step 4: View the assembly with the in-plane substituents of macrocycle pointing away from the observer and the in plane substituents of the axle prochiral pointing towards the observer.

Step 5: View the relative orientation of the **A→B** and **C→D** vectors and consider the direction of rotation from the head of the front vector to the tail of the rear vector. A right-handed path assigned as (R_{ma}) and a left-handed path assigned as (S_{ma}).

Worked example – rotaxane 15

Step 1: The highest CIP prochiral center in the macrocycle is the sulfoxide unit. This is then redrawn such that the in-plane methylene units point into the cavity of the ring.

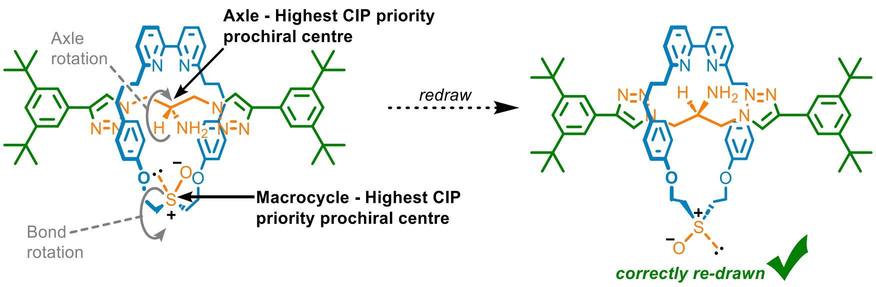

Step 2: The highest priority exocyclic atom bonded to the sulfoxide according to CIP rules is the oxygen, which is labelled as "**A**", while the lone pair is labelled as "**B**".

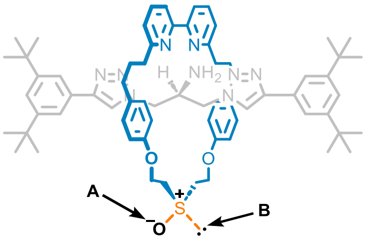

Step 3: The highest priority prochiral center in the axle is the carbon bearing the amine unit. The highest priority exocyclic atom bonded to the prochiral carbon is the amine nitrogen, which is labelled as "**C**", while the proton is labelled as "**D**".

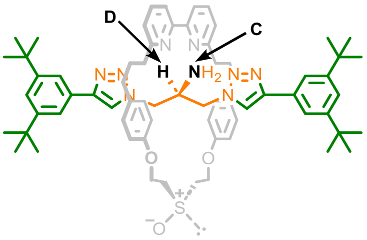

Step 4: Viewing the assembly with the in-plane substituents of the macrocycle pointing away from the observer and those of the axle pointing towards the observer an anticlockwise path is taken to the head of the vector defined by **C→D** vector from the tail of the **A→B** vector. Thus the stereoisomer of rotaxane **5** shown (S_{ma})-**5.**

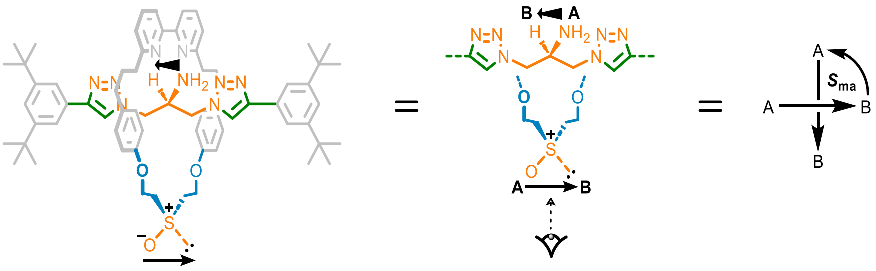

8.4. Absolute stereochemical assignment of type I rotaxane mechanical geometric isomers

Step 1: In the axle, identify the highest priority atom using the (CIP) priority rules and label it as "**A**".

Step 2: Moving outward from **A** in spheres, as per the CIP method for assigning covalent stereogenic centres, determine the highest priority atom (CIP) that can be used to define an orientation of the axle (typically a ligand of **A**) and label it as "**B**". The orientation of the axle is defined by the vector **A**→**B**, which, where relevant, passes through the intervening atoms (i.e., follows the bonds).

Step 3: In the macrocycle, identify the highest priority prochiral group (or stereogenic unit in *meso* structures) using the CIP priority of the central atom. Redraw the macrocycle such that the in-plane substituents of the prochiral centre point towards the center of the ring.

Step 4: Identify the highest priority ligand of the identified center that lie outside of the macrocycle plane, again using CIP priority, and label it as "**C**"; label the lower priority group "**D**".

Step 5: View the relative orientation of the vectors **A**→**B** and **C**→**D**. If the vectors point in the same direction, the molecule will be labelled as (Z_m). If they point toward opposite directions, the molecule will be labelled as (E_m).

Worked example - rotaxane 6

Step 1: The atom of the axle with the highest priority is N_2, which is labelled as **A.**

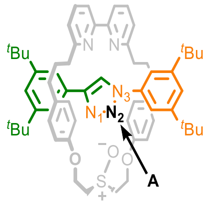

Step 2: The highest priority ligand of **A** is N_3, which allows the orientation of the axle to be assigned, and so is labelled **B**.

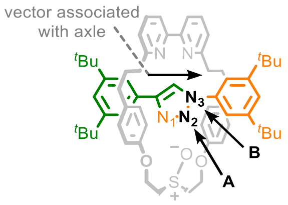

Step 3: The sulfoxide moiety is the only prochiral unit in the macrocycle. The macrocycle is redrawn such that the in-plane methylene substituents of the prochiral centre point towards the axle.

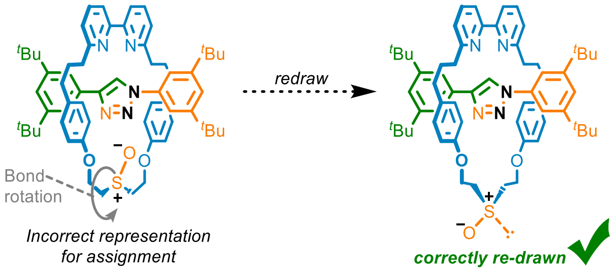

Step 4: The highest priority exocyclic atom bonded to the prochiral unit is the oxygen, which is labelled **C**. The lone pair is labelled **D**, allowing the vector associated with the macrocycle to be defined.

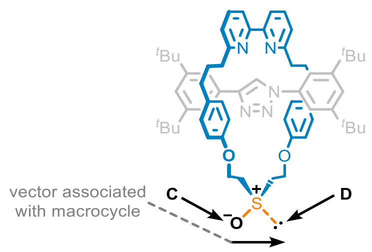

Step 5: The vectors **A**→**B** (associated with the axle) and **C**→**D** (associated with the macrocycle) point in the same direction and so the stereoisomer of rotaxane **6** shown is labelled as (Z_m)-**6**.

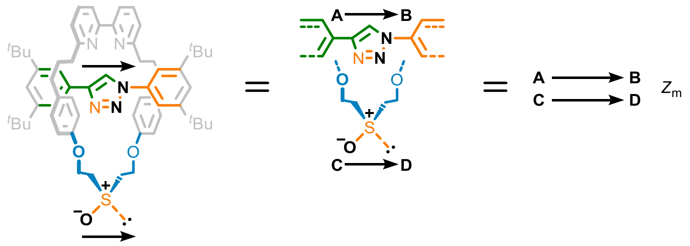

8.5. Absolute stereochemical assignment of catenane mechanical geometric isomers

Step 1: Identify the highest priority atom in the oriented macrocycle using the CIP priority rules and label it as "**A**".

Step 2: Moving outward from **A** in spheres, as per the CIP method for assigning covalent stereogenic centres, determine the highest priority atom (CIP) that can be used to define an orientation of the axle (typically a ligand of **A**) and label it as "**B**". The orientation of the macrocycle is defined by the vector **A**→**B**, which, where relevant, passes through the intervening atoms (i.e., follows the bonds).

Step 3: In the facially dissymmetric macrocycle, identify the highest priority prochiral group (or stereogenic unit in *meso* structures) using the CIP priority of the central atom. Redraw the macrocycle such that the in-plane substituents of the prochiral center point towards the center of the ring.

Step 4: Identify the highest priority exocyclic ligand of the identified prochiral center, again using CIP priority, and label it as "**C**"; label the lower priority group "**D**".

Step 5: Redraw the assembly such that the vector **A**→**B** passes through the cavity of the facially dissymmetric macrocycle and the prochiral center is in the in the cavity of the oriented ring.

Step 6: View the relative orientation of the vectors **A**→**B** and **C**→**D** at the crossing point between the rings. If the vectors point in the same direction, the molecule will be labelled as (Z_m). If they point in opposite directions, the molecule will be labelled as (E_m).

Worked example - catenane 14

Step 1: The highest atom according to the CIP rules in the oriented, triazole-containing macrocycle is the oxygen of the ester moiety, which is labelled as "**A**".

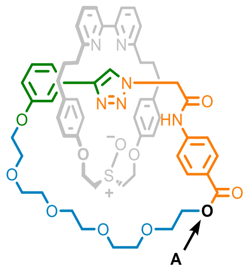

Step 2: Moving outwards, the highest priority atom defining an orientation of the ring is the carbon of the carboxylic moiety. This is labelled as "**B**", and allows to identify the vector **A→B.**

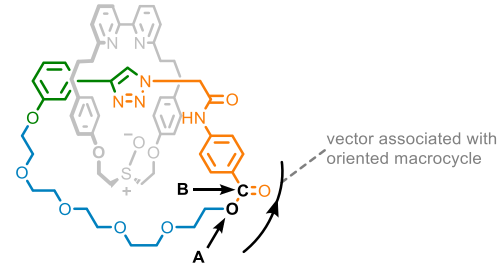

Step 3: The sulfoxide moiety is the only prochiral unit in the facially dissymmetric macrocycle so the macrocycle needs to be drawn such that the methylene units attached to S point into the cavity of the ring.

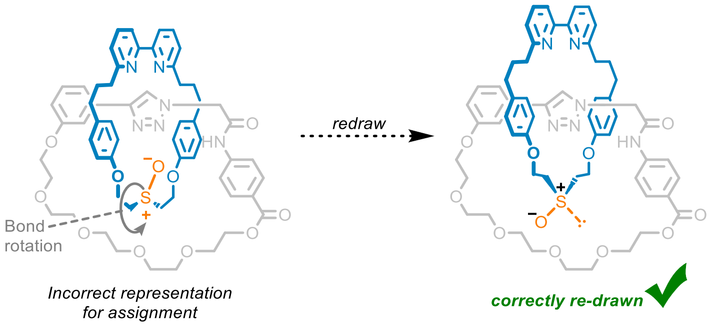

Step 4: The highest priority atom on the prochiral unit lying on the oriented macrocycle plane is the oxygen, which is labelled as "**C**". The lower priority group is the lone pair, which is labelled as "**D**".

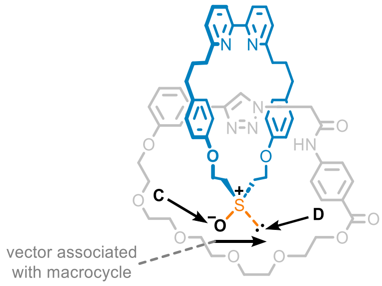

Step 5: Redraw the assembly such that the vector A→B passes through the facially dissymmetric macrocycle and the prochiral unit is in the center of the oriented macrocycle.

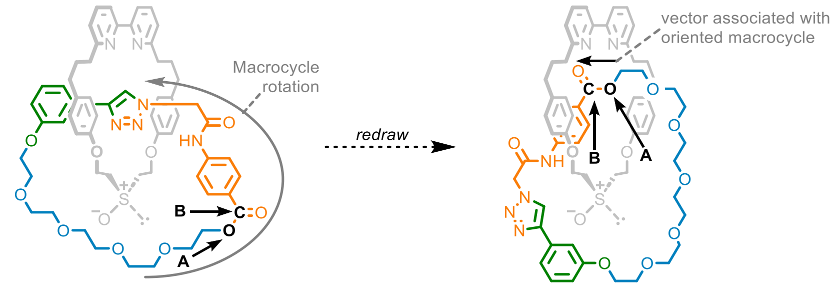

Step 6: View the relative orientations of the vectors **A→B** and **C→D** at the crossing point between the rings. As they point in opposite directions, the stereoisomer of catenene **14** shown is labelled as (E_{m})-**14.**

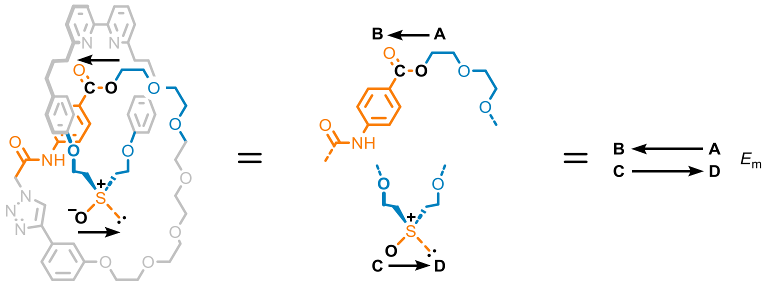

9. Effect of temperature and solvent on the diastereoselective synthesis of rotaxanes 6

Varying the solvent used in the AT-CuAAC synthesis of rotaxanes **6** showed a minor effect on the selectivity, whith the *dr* ranging between 20 and 29% with the exception of CH_3CN, which led to a drop in selectivity (Table S1, entry 4). A stronger effect could be observed by varying the temperature. A higher temperature (60 °C) led to a lower *de* (20%, Table S1, entry 8), while running the reaction at -20 °C led to an increase of the *de* to 40% (Table S1, entry 9). Furtherly lowering the temperature was detrimental to the selectivity, with a 37% *de* observed after running the reaction at -40 °C and a 16% *de* at -78 °C.

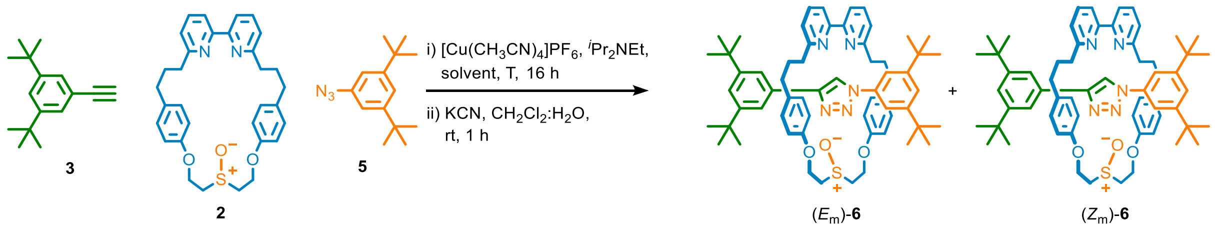

Scheme S3 AT-CuAAC synthesis of rotaxanes **6**

Table S1 Effect of solvent and temperature on the diastereoselectivity of rotaxane **6**

Entry	Solvent	Temperature [°C]	Selectivity[a]
1	CH_2Cl_2	rt	24% *de*
2	$CHCl_3$	rt	22% *de*
3	1:1 PhMe : CH_2Cl_2	rt	24% *de*
4	CH_3CN	rt	10% *de*
5	THF	rt	28% *de*
6	EtOH	rt	29% *de*
7	9:1 THF:H_2O	rt	25% *de*
8	THF	60	20% *de*
9	THF	-20	40% *de*
10	THF	-40	37% *de*
11	THF	-78	16% *de*

[a]Determined by ^1H-NMR analysis of the crude reaction mixture after demetallation

10. Effect of conditions on the enantioselectivity of the AT-CuAAC reaction (Scheme 6)

10.1. Effect of temperature on the direct enantioselective synthesis of rotaxanes 15

Varying the temperature of the AT-CuAAC synthesis of rotaxanes **15** led to an increased enantioselectivity at lower temperatures. This was evident for both enantiomers of **1f** and maximum *ee* values of +41.52% and -41.48% were achieved when (*R*)-**1f** and (*S*)-**1f** were used at -78 °C (Table S2, entries 3 and 6).

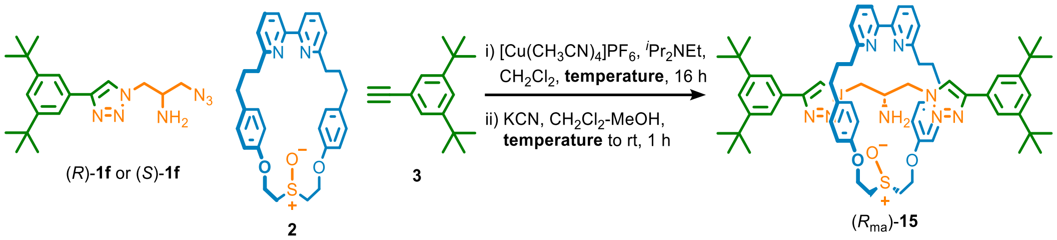

Scheme S4. AT-CuAAC synthesis of rotaxanes **15**

Table S2. Effect of temperature on the enantioselective synthesis of rotaxanes **15**

entry	Substrate	Conditions	Selectivity[a]
1	(*R*)-**1f**	CH_2Cl_2, rt	+16.10% *ee*
2	(*R*)-**1f**	CH_2Cl_2, –40 °C	+28.44% *ee*
3	(*R*)-**1f**	CH_2Cl_2, –78 °C	+41.52% *ee*
4	(*S*)-**1f**	CH_2Cl_2, rt	-16.70% *ee*
5	(*S*)-**1f**	CH_2Cl_2, –40 °C	-25.46 % *ee*
6	(*S*)-**1f**	CH_2Cl_2, –78 °C	-41.48% *ee*

[a]Determined by CSP-HPLC analysis of purified products as seen in Figure S209

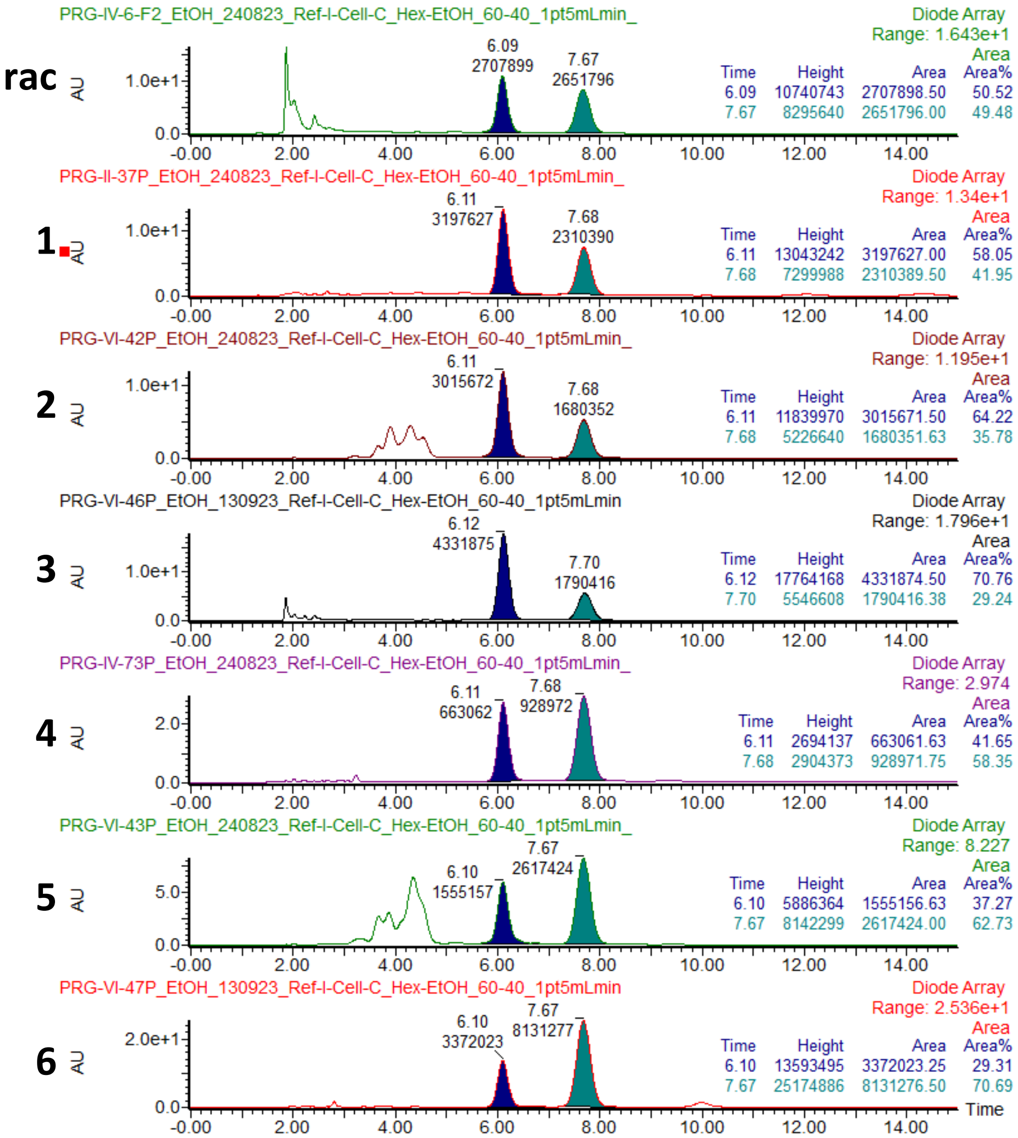

Figure S209. CSP-HPLC analysis of rotaxane **15** produced under different conditions (numbers indicate the relevant entry in Table S2). Samples loaded in EtOH, Regis Reflect I-Cellulose C, *n*-hexane-EtOH 60 : 40, flowrate 1.5 mLmin^{-1}.

10.2. Effect of temperature on the enantioselective synthesis of rotaxanes 16

Varying the temperature of the AT-CuAAC synthesis of rotaxanes **16** led to an increased enantioselectivity at lower temperatures. This was evident for both enantiomers of **1g** and maximum *ee* values of +67.26% and -69.94% were achieved when (*R*)-**1g** and (*S*)-**1g** were used at -40 °C (Table S3, entries 2 and 5).

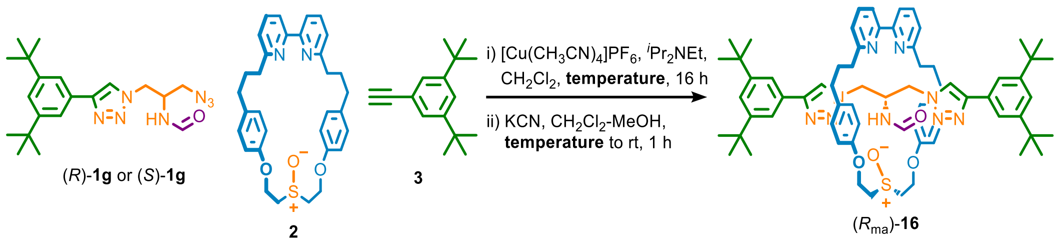

Scheme S5. AT-CuAAC synthesis of rotaxanes **16**

Table S3. Effect of temperature on the enantioselective synthesis of rotaxanes **16**

entry	Substrate	Conditions	Selectivity[a]
1	(*R*)-**1g**	CH_2Cl_2, rt	+57.06% *ee*
2	(*R*)-**1g**	CH_2Cl_2, –40 °C	+67.26% *ee*
3	(*R*)-**1g**	CH_2Cl_2, –78 °C	+58.70% *ee*
4	(*S*)-**1g**	CH_2Cl_2, rt	-61.94% *ee*
5	(*S*)-**1g**	CH_2Cl_2, –40 °C	-69.94 % *ee*
6	(*S*)-**1g**	CH_2Cl_2, –78 °C	-63.60% *ee*

[a]Determined by CSP-HPLC analysis of purified products as seen in Figure S210

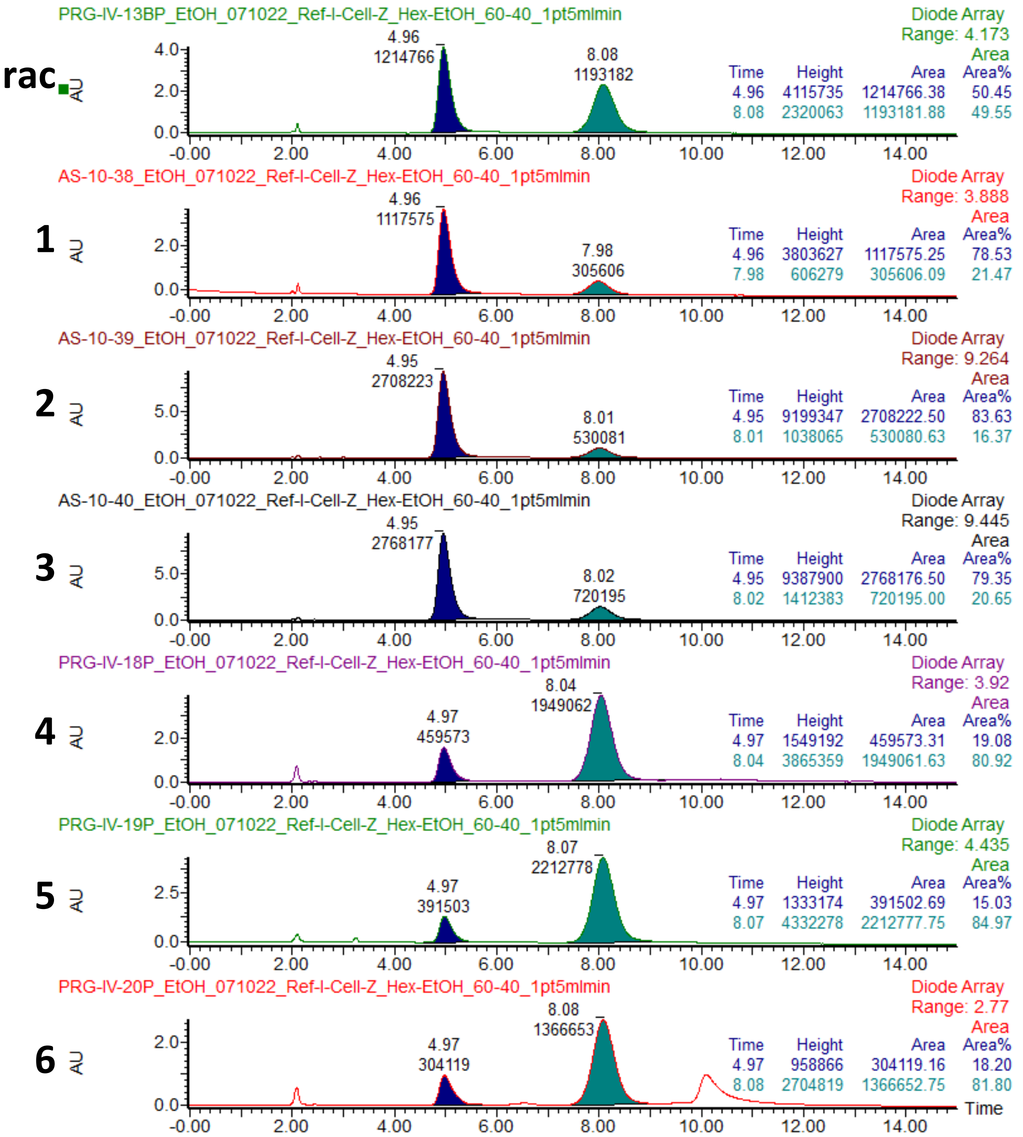

Figure S210. CSP-HPLC analysis of rotaxane **16** produced under different conditions (numbers indicate the relevant entry in Table S3). Samples loaded in EtOH, Regis Reflect I-Cellulose Z, *n*-hexane-EtOH 60 : 40, flowrate 1.5 mLmin^{-1}.

11. Variable temperature NMR analysis of rotaxane 16

Rotaxane **16** can exist as two diastereomeric co-conformations depending on whether the macrocycle is displaced across the amide unit to give render the prochiral centre $R_{co\text{-}c}$ or $S_{co\text{-}c}$. If these co-conformations are in fast exchange, we would expect to see a single set of signals corresponding to weighted average of the spectra of the two diastereomers. If they are in slow exchange, we expect to see two sets of signals corresponding to the two diastereomeric co-conformations, the ratio between which is not required to be unity as they are not necessarily equienergetic.

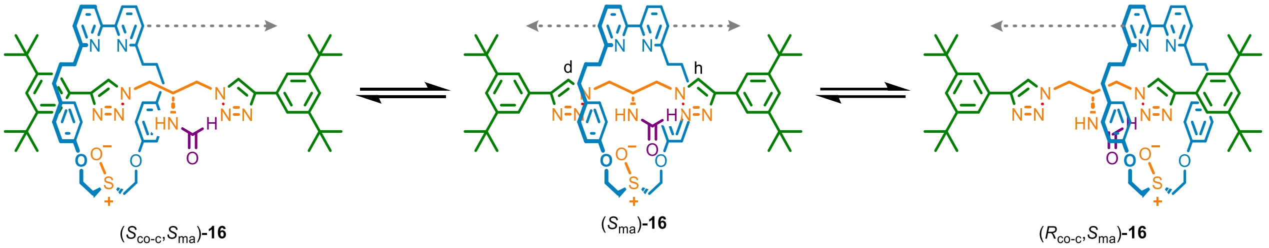

Scheme S6. Illustration of the shuttling process in rotaxane **16** to afford diastereomeric co-conformations.

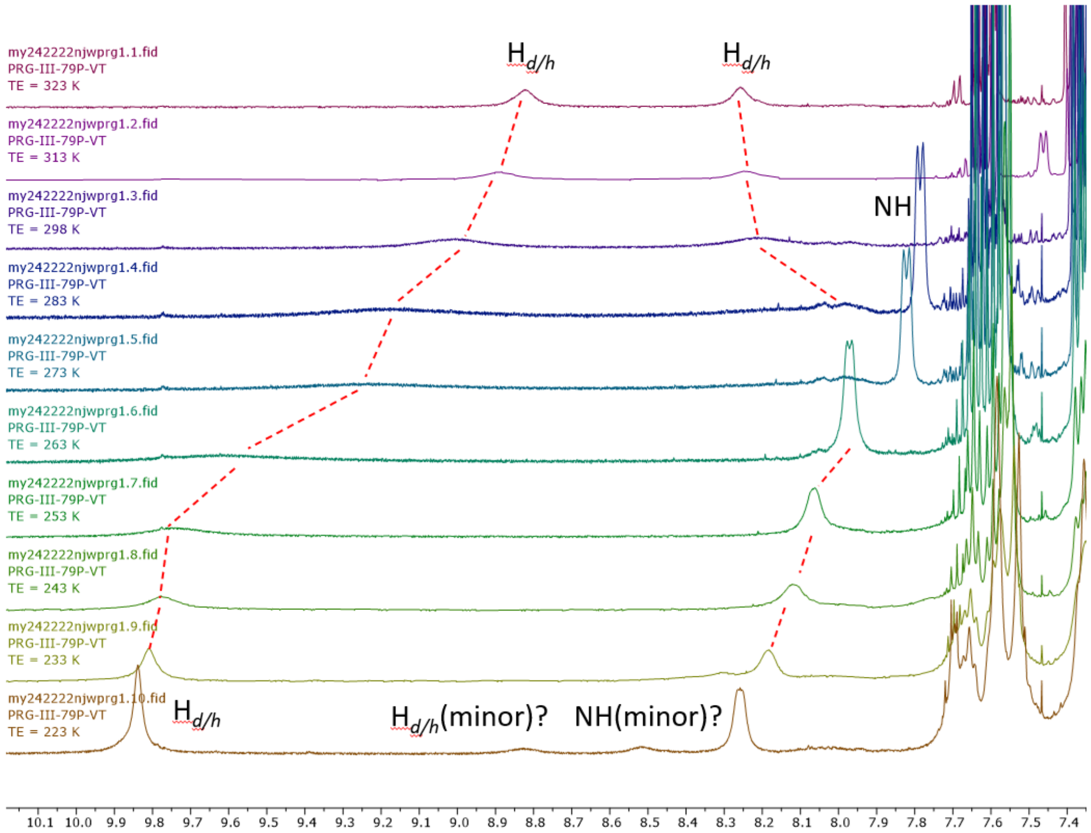

Figure S211. Partial ^{1}H VT-NMR ($CDCl_3$, 500 MHz) of formamide rotaxane **16**.

^{1}H NMR analysis (Figure S211) suggests that at 298 K exchange between these co-conformations is relatively slow on the NMR timescale - the peaks corresponding to triazole protons H_d and H_h appear as two broad resonances rather than two sharp signals (fast exchange) or two sets of two signals (slow exchange). In keeping with this, raising the temperature resulted in a sharpening of the signals corresponding to H_d and H_h, suggesting that at 298 K the spectrum is above the coalescence temperature. Conversely, cooling the sample caused the two signals observed at 298 K to broaden and shift (one to higher ppm, one to lower). At low temperature (223 K) one becomes sharper and stabilises around 9.85 ppm. The other seems to be obscured by other resonances. Also, as

the temperature is lowered, a broad doublet that we assign as the NH of the formamide unit shifts to higher ppm and broadens before sharpening again. At 223 K, a minor second set of signals appears that could correspond to the occupied triazole and NH of the second diastereomeric co-conformation. If this is the correct interpretation, one of the diastereomeric co-conformations of rotaxane **16** is significantly preferred.

Based on this analysis, we are confident that the formamide unit is small enough to permit the macrocycle to shuttle between the compartments at rt, confirming that the only fixed stereogenic unit is the mechanical axial unit. Further work is required to fully establish the co-conformational behaviour of the mechanical axial stereogenic unit.

12. Single Crystal X-ray Diffraction Analysis

12.1. Single Crystal X-ray Diffraction Data for rotaxane (R_{ma},$R_{co\text{-}c}$)-4d

Single crystals of (R_{ma},$R_{co\text{-}c}$)-**4d** were grown by slow vapour diffusion of n-pentane into a concentrated CH_2Cl_2 solution of (R_{ma},$R_{co\text{-}c}$)-**4d**, yielding large colourless plate crystals. Data was collected at 100 K on a Rigaku 007 HF diffractometer equipped with a HyPix 6000HE hybrid pixel array detector. Cell determination, data reduction, cell refinement, and absorption correction were processed by CrysalisPro[8] The structure was solved within Olex2[9] by ShelXT[10] with refinement by ShelXL.[11] The asymmetric cell contained two molecules of $C_{69}H_{84}N_9O_4F_3S$ and disordered solvent. Modelling of the solvent suggested the presence of three dichloromethane molecules and one pentane molecule. The disorder of the solvent, however, was found to preclude a satisfactory solution and so the program SQUEEZE[12] implemented within PLATON[13] was used to account for the electron density within this region of the unit cell. Solvent accessible voids of 1593Å^3 were identified and 469 electrons per unit cell were recovered.

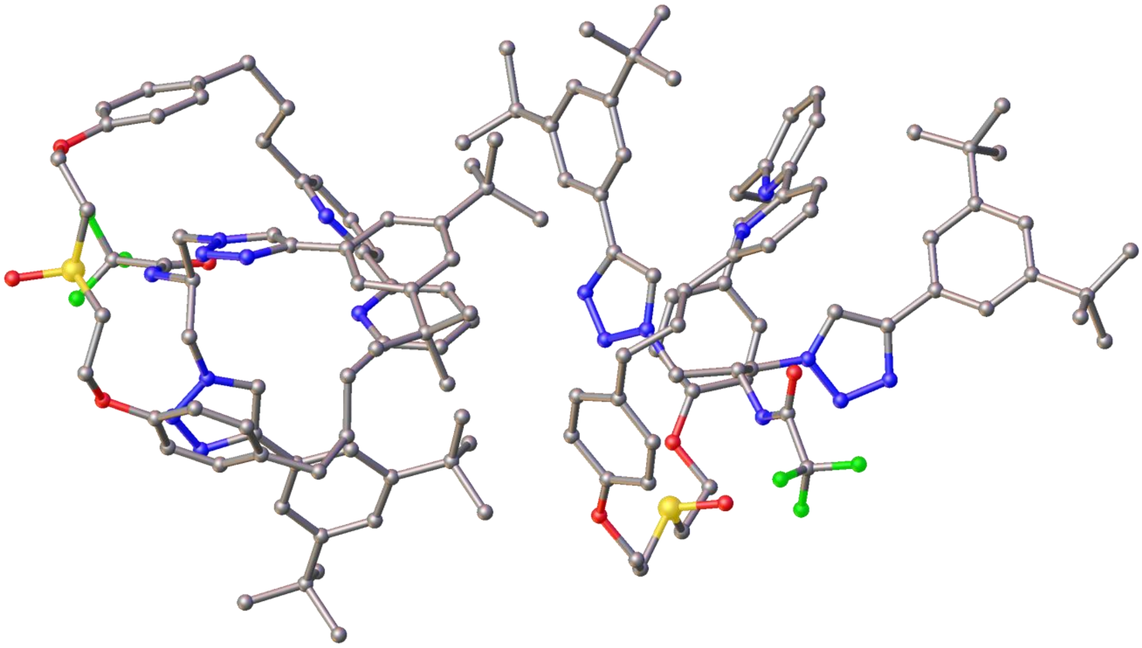

Figure S212. Solid state structurer of (R_{ma},$R_{co\text{-}c}$)-**4d** shown in ball and stick mode with H-atoms and disorder omitted for clarity. N.B. this is a Z'=2 structure with two crystallographically independent rotaxanes in the asymmetric unit.

**Compound**	(R_{ma},$R_{co\text{-}c}$)-**4d**
Formula	$C_{69}H_{84}N_9O_4F_3S$
$D_{calc.}$/ g cm^{-3}	1.039
m/mm^{-1}	0.096
Formula Weight	1192.51
Colour	colourless
Shape	plate
Size/mm^3	0.36×0.22×0.08
T/K	100.00(10)
Crystal System	triclinic
Space Group	P-1
a/Å	18.6219(2)
b/Å	19.1615(2)
c/Å	22.3951(3)
α/°	85.885(1)
β/°	77.745(1)
γ/°	77.691(1)
V/Å^3	7626.55(16)
Z	4
Z'	2
Wavelength/Å	0.71073
Radiation type	Mo K_a
Q_{min}/°	1.6
Q_{max}/°	29.2
Measured Refl's.	148929
Indep't Refl's	34957
Refl's I≥2 s(I)	22248
R_{int}	0.0498
Parameters	1621
Restraints	1084
Largest Peak	1.13
Deepest Hole	-0.88
GooF	1.015
wR_2 (all data)	0.2527
wR_2	0.2266
R_1 (all data)	0.1404
R_1	0.0941
CCDC Dep. No.	2303663

12.2. Single Crystal X-ray Diffraction Data for rotaxane (Z_m)-6

Single colourless block-shaped crystals of (Z_m)-**6** were grown by slow evaporation of an Et_2O-petrol solution. A suitable crystal 0.60×0.19×0.11 mm^3 was selected and mounted on a MITIGEN holder in oil on a Rigaku 007HF diffractometer with HF Varimax confocal mirrors, an AFC11 goniometer and HyPix 6000HE detector. The crystal was kept at a steady T = 100(2) K during data collection. The structure was solved with the ShelXT 2018/2[11] structure solution program using the using dual methods solution method and by using Olex2 1.5-alpha as the graphical interface.[9] The model was refined with version of olex2.refine 1.5-alpha using full matrix least squares minimisation on $\boldsymbol{F^2}$ minimisation.[14] All non-hydrogen atoms were refined anisotropically. Hydrogen atom positions were calculated geometrically and refined using the riding model. Thermal restraints applied to solvent isohexane.

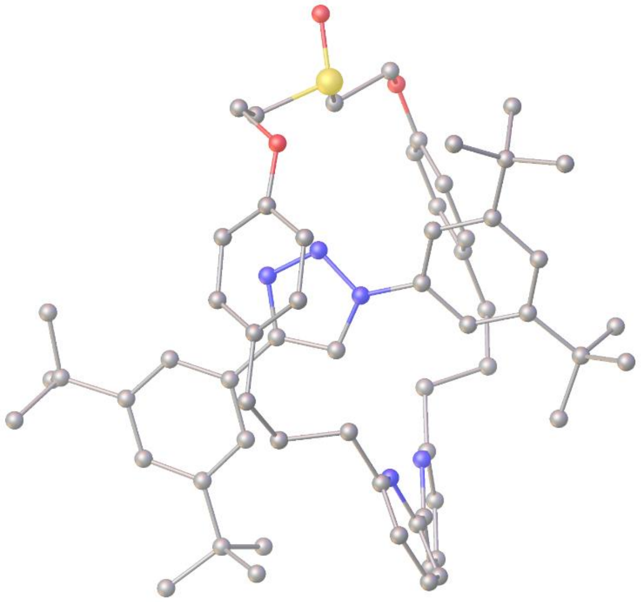

Figure S213. Solid state structure of (Z_m)-**6** shown in ball and stick mode with the solvent isohexane molecule and H-atoms omitted and minor disorder component 'ghosted' for clarity.

**Compound**	(Z_m)-**6**
Formula	$C_{68}H_{91}N_5O_3S$
$D_{calc.}$/ g cm^{-3}	1.115
m/mm^{-1}	0.819
Formula Weight	1058.578
Colour	colourless
Shape	block-shaped
Size/mm^3	0.60×0.19×0.11
T/K	100(2)
Crystal System	monoclinic
Space Group	$P2_1/n$
a/Å	17.5926(1)
b/Å	16.1257(1)
c/Å	22.3794(2)
α/°	90
β/°	96.449(1)
γ/°	90
V/Å^3	6308.70(8)
Z	4
Z'	1
Wavelength/Å	1.54184
Radiation type	Cu K_{α}
Q_{min}/°	3.39
Q_{max}/°	70.33
Measured Refl's.	59812
Indep't Refl's	11798
Refl's I≥2 *s*(I)	10779
R_{int}	0.0302
Parameters	709
Restraints	30
Largest Peak	0.8447
Deepest Hole	-0.6096
GooF	1.0411
Wr_2 (all data)	0.1495
Wr_2	0.1458
R_1 (all data)	0.0567
R_1	0.0531
CCDC Dep. No.	2307121

12.3. Single Crystal X-ray Diffraction Data for rotaxane (E_m)-6

Single colourless needle-shaped crystals of (E_m)-**6** were grown by slow evaporation of an Et_2O-petrol solution. A suitable crystal 0.27×0.02×0.01 mm^3 was selected and mounted on a MITIGEN holder in oil on a Rigaku 007HF diffractometer with HF Varimax confocal mirrors, an AFC11 goniometer and HyPix 6000HE detector. The crystal was kept at a steady T = 100(2) K during data collection. The structure was solved with the ShelXT 2018/2[7] structure solution program using the using dual methods solution method and by using Olex2 1.5-alpha[8] as the graphical interface. The model was refined with version of olex2.refine 1.5-alpha[9] using full matrix least squares minimisation on $\boldsymbol{F^2}$ minimisation. All non-hydrogen atoms were refined anisotropically. Hydrogen atom positions were calculated geometrically and refined using the riding model. Thermal restraints applied to solvent isohexane.

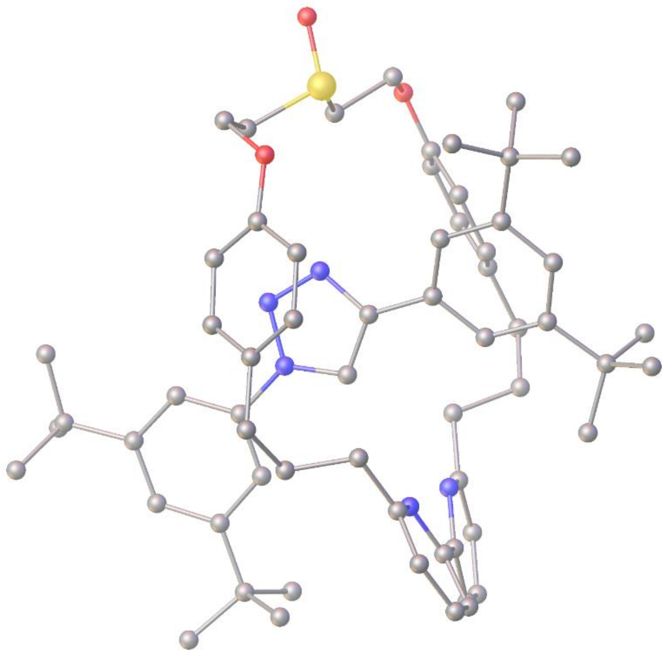

Figure S214. Solid state structure of (E_m)-**6** shown in ball and stick mode with the solvent isohexane molecule and H-atoms omitted and minor disorder component 'ghosted' for clarity.

**Compound**	(E_m)-**6**
Formula	$C_{68}H_{91}N_5O_3S$
$D_{calc.}$/ g cm^{-3}	1.118
m/mm^{-1}	0.822
Formula Weight	1058.578
Colour	colourless
Shape	needle-shaped
Size/mm^3	0.27×0.02×0.01
T/K	100(2)
Crystal System	monoclinic
Space Group	$P2_1/n$
a/Å	17.6111(3)
b/Å	16.0502(3)
c/Å	22.3898(4)
α/°	90
β/°	96.445(2)
γ/°	90
V/Å^3	6288.7(2)
Z	4
Z'	1
Wavelength/Å	1.54184
Radiation type	Cu K_a
Q_{min}/°	3.03
Q_{max}/°	51.80
Measured Refl's.	36644
Indep't Refl's	6846
Refl's I≥2 *s*(I)	6099
R_{int}	0.0506
Parameters	709
Restraints	30
Largest Peak	0.8008
Deepest Hole	-0.4720
GooF	1.0674
Wr_2 (all data)	0.1754
Wr_2	0.1695
R_1 (all data)	0.0684
R_1	0.0625
CCDC Dep. No.	2307122

12.4. Single Crystal X-ray Diffraction Data for rotaxane (Z_m)-9

Single colourless slab-shaped crystals of (Z_m)-**9** were recrystallised from a mixture of ^{n}pentane and CH_2Cl_2 by solvent layering. A suitable crystal was selected and mounted on a MITIGEN holder in oil on a Rigaku 007HF diffractometer equipped with Arc)Sec VHF Varimax confocal mirrors and a UG2 goniometer and HyPix 6000HE detector. The crystal was kept at a steady T = 100(2) K during data collection. The structure was solved with the ShelXT 2018/2[7] structure solution program using the using dual methods solution method and by using Olex2 1.5-alpha[8] as the graphical interface. The model was refined with ShelXL 2018/3 (Sheldrick, 2015)[10] using full matrix least squares minimisation on $\boldsymbol{F^2}$ minimisation. All non-hydrogen atoms were refined anisotropically. Hydrogen atoms positions were calculated geometrically except amine H-atoms which were located in the difference map. All hydrogen atoms were refined with the riding model.

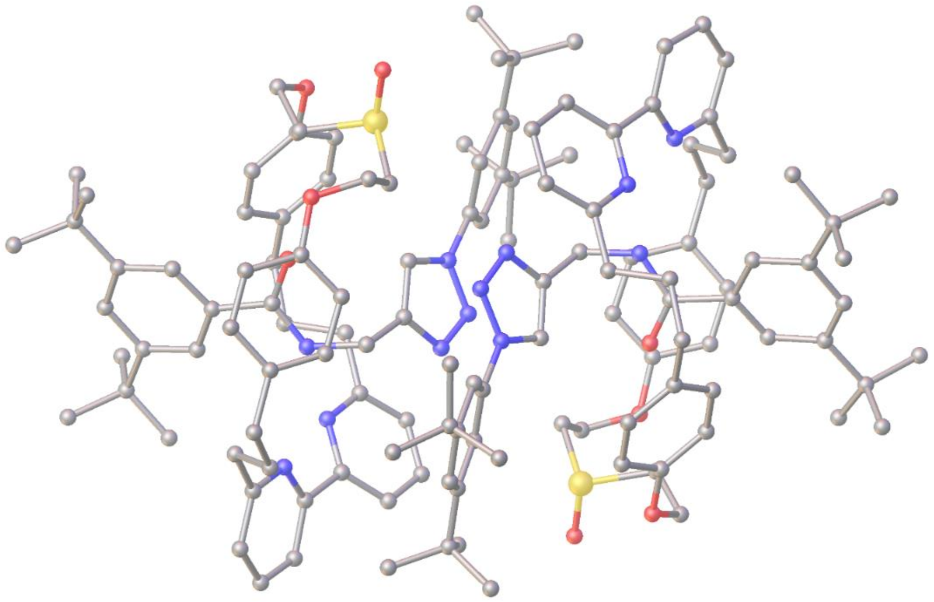

Figure S215. Solid state structurer of (Z_m)-**9** shown in ball and stick mode with H-atoms omitted for clarity. N.B. this is a Z'=2 structure with two crystallographically independent rotaxanes in the asymmetric unit.

**Compound**	(Z_m)-**9**
Formula	$C_{64}H_{80}N_6O_4S$
$D_{calc.}$/ g cm^{-3}	1.185
m/mm^{-1}	0.903
Formula Weight	1029.40
Colour	colourless
Shape	block-shaped
Size/mm^3	0.10×0.09×0.02
T/K	100.00(10)
Crystal System	monoclinic
Space Group	$P2_1/n$
a/Å	26.6812(2)
b/Å	13.15830(10)
c/Å	34.4438(3)
α/°	90
β/°	107.3140(10)
γ/°	90
V/Å^3	11544.57(17)
Z	8
Z'	2
Wavelength/Å	1.54178
Radiation type	Cu K_a
Q_{min}/°	2.490
Q_{max}/°	75.129
Measured Refl's.	123562
Indep't Refl's	23477
Refl's I≥2 *s*(I)	18146
R_{int}	0.0864
Parameters	1381
Restraints	0
Largest Peak	0.600
Deepest Hole	-0.507
GooF	1.024
Wr_2 (all data)	0.1594
Wr_2	0.1452
R_1 (all data)	0.0736
R_1	0.0557
CCDC No.	2307119

12.5. Single Crystal X-ray Diffraction Data for rotaxane (E_m)-11

Single colourless slab-shaped crystals of (E_m)-**11** were grown by slow evaporation of a CH_2Cl_2 solution at room temperature. A suitable crystal was selected and mounted on a MITIGEN holder in oil on a Rigaku 007HF diffractometer equipped with Arc)Sec VHF Varimax confocal mirrors and a UG2 goniometer and HyPix 6000HE detector. The crystal was kept at a steady *T* = 100(2) K during data collection. The structure was solved with the ShelXT 2018/2[7] structure solution program using the using dual methods solution method and by using Olex2 1.5-alpha[8] as the graphical interface. The model was refined with ShelXL 2018/3[10] using full matrix least squares minimisation on $\boldsymbol{F^2}$ minimisation. All non-hydrogen atoms were refined anisotropically. Hydrogen atoms positions were calculated geometrically except amine H-atoms which were located in the difference map. All hydrogen atoms were refined with the riding model. One tBu group was modelled as disordered with equal distance 1,2 and 1,3 geometric restraints applied to equivalent atom pairs of each disorder component. Thermal restraints were applied to all disorder components.

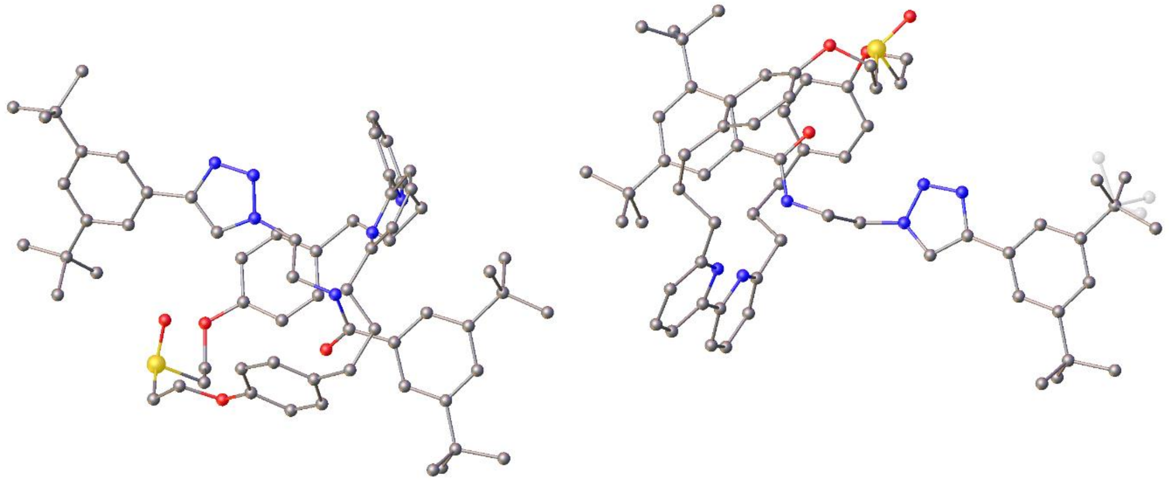

Figure S216. Solid state structure of (E_m)-**11** shown in ball and stick mode with H-atoms omitted and minor disorder component 'ghosted' for clarity. N.B. this is a Z'=2 structure with two crystallographically independent rotaxanes in the asymmetric unit.

**Compound**	(E_m)-**11**
Formula	$C_{65}H_{82}N_6O_4S$
$D_{calc.}$/ g cm^{-3}	1.177
m/mm^{-1}	0.892
Formula Weight	1043.42
Colour	colourless
Shape	slab-shaped
Size/mm^3	0.11×0.11×0.04
T/K	100(2)
Crystal System	orthorhombic
Space Group	*Pbca*
a/Å	16.2520(3)
b/Å	19.8197(3)
c/Å	73.1056(11)
α/°	90
β/°	90
γ/°	90
V/Å^3	23548.0(7)
Z	16
Z'	2
Wavelength/Å	1.54178
Radiation type	Cu K_a
Q_{min}/°	3.719
Q_{max}/°	66.595
Measured Refl's.	102466
Indep't Refl's	20372
Refl's I≥2 s(I)	15040
R_{int}	0.1019
Parameters	1430
Restraints	39
Largest Peak	0.745
Deepest Hole	-0.479
GooF	1.062
wR_2 (all data)	0.2545
wR_2	0.2369
R_1 (all data)	0.1245
R_1	0.0987
CCDC Dep. No.	2307120

12.6. Single Crystal X-ray Diffraction Data for rotaxane *rac*-16

Single colourless lath-shaped crystals of *rac*-**16** were grown by slow evaporation of a Et_2O solution at in a fridge (*ca.* 5 °C). A suitable crystal was selected and mounted on a MITIGEN holder in oil on a Rigaku 007HF diffractometer equipped with Arc)Sec VHF Varimax confocal mirrors and a UG2 goniometer and HyPix 6000HE detector. The crystal was kept at a steady *T* = 100(2) K during data collection. The structure was solved with the ShelXT 2018/2[11] structure solution program using the using dual methods solution method and by using Olex2 1.5-alpha as the graphical interface.[9] The model was refined with olex2.refine 1.5-alpha using full matrix least squares minimisation on $\boldsymbol{F^2}$ minimisation.[14] All non-hydrogen atoms were refined anisotropically. Hydrogen atom positions were calculated geometrically and refined using the riding model. The disorder in the macrocycle and triazole was assumed to be linked and modelled accordingly (ca.78:22). Additional disorder in the iPr groups was modelled independently (ca. 84:16 and ca, 63:37). Equivalent 1,2 and 1,3 pairs of atoms of each disorder component have had equal distance restraints applied. RIGU thermal restraints were applied to all atoms. ISOR thermal restraints were applied to minor disorder component triazole N-atoms.

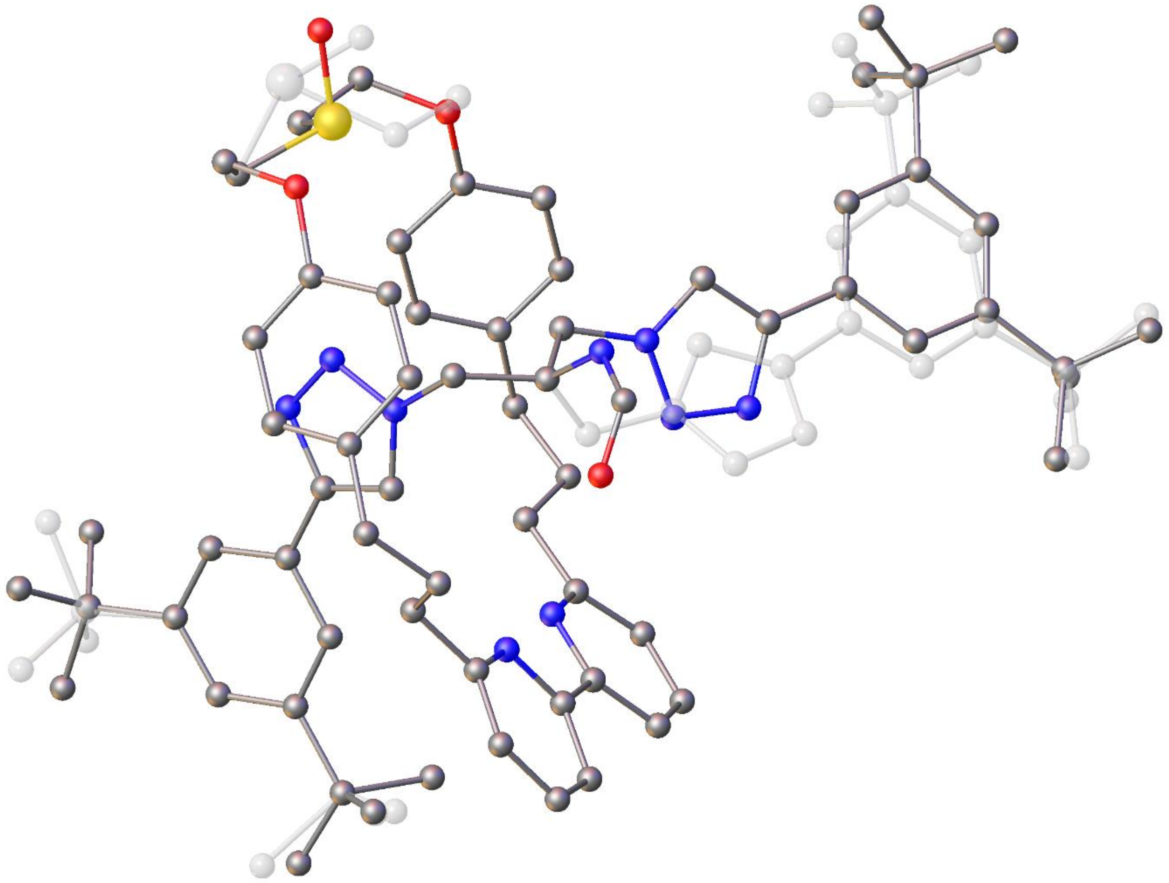

Figure S217. Solid state structurer of *rac*-**16** shown in ball and stick mode with H-atoms omitted and minor disorder component 'ghosted' for clarity.

**Compound**	*rac*-**16**
Formula	$C_{68}H_{84}N_9O_4S$
$D_{calc.}$/ g cm^{-3}	1.193
m/mm^{-1}	0.890
Formula Weight	1123.549
Colour	colourless
Shape	lath-shaped
Size/mm^3	0.65×0.04×0.01
T/K	100.00(10)
Crystal System	monoclinic
Space Group	$C2/c$
a/Å	36.4756(10)
b/Å	10.6634(2)
c/Å	32.8280(7)
α/°	90
β/°	101.629(2)
γ/°	90
V/Å^3	12506.5(5)
Z	8
Z'	1
Wavelength/Å	1.54178
Radiation type	Cu K_{a}
Q_{min}/°	2.75
Q_{max}/°	66.60
Measured Refl's.	52802
Indep't Refl's	11009
Refl's I≥2 s(I)	7468
R_{int}	0.0817
Parameters	1043
Restraints	1141
Largest Peak	1.4952
Deepest Hole	-0.8568
GooF	1.0793
wR_2 (all data)	0.2964
wR_2	0.2612
R_1 (all data)	0.1259
R_1	0.0933
CCDC Dep. No.	2307076

13. References

1. Pigorsch, A.; Köckerling, M., The Crystallization of Extended Niobium-Cluster Framework Compounds: A Novel Approach Using Ionic Liquids. *Crystal Growth and Design* **2016**, *16* (8), 4240.

2. Maynard, J. R. J.; Gallagher, P.; Lozano, D.; Butler, P.; Goldup, S. M., Mechanically axially chiral catenanes and noncanonical mechanically axially chiral rotaxanes. *Nat. Chem.* **2022**, *14* (9), 1038.

3. Ogi, S.; Ikeda, T.; Wakabayashi, R.; Shinkai, S.; Takeuchi, M., A bevel-gear-shaped rotor bearing a double-decker porphyrin complex. *Chem. Eur. J.* **2010**, *16* (28), 8285.

4. Zhu, W.; Ma, D., Synthesis of aryl azides and vinyl azides via proline-promoted CuI-catalyzed coupling reactions. *Chem. Commun.* **2004**, , 888.

5. Jinks, M. A.; de Juan, A.; Denis, M.; Fletcher, C. J.; Galli, M.; Jamieson, E. M. G.; Modicom, F.; Zhang, Z.; Goldup, S. M., Stereoselective Synthesis of Mechanically Planar Chiral Rotaxanes. *Angew. Chem. Int. Ed.* **2018**, *57* (45), 14806.

6. Jamieson, E. M. G.; Modicom, F.; Goldup, S. M., Chirality in rotaxanes and catenanes. *Chem. Soc. Rev.* **2018**, *47* (14), 5266.

7. Savoini, A.; Gallagher, P. R.; Saady, A.; Goldup, S. M., The Final Stereogenic Unit of [2]Rotaxanes: Type 2 Geometric Isomers. *J. Am. Chem. Soc.* **2024**, 10.1021/jacs.3c14594.

8. CrysAlisPro Software System, Rigaku Oxford Diffraction, (2021).

9. Dolomanov, O. V.; Bourhis, L. J.; Gildea, R. J.; Howard, J. A. K.; Puschmann, H., OLEX2: a complete structure solution, refinement and analysis program. *J. Appl. Cryst.* **2009**, *42* (2), 339.

10. Sheldrick, G. M., SHELXT - integrated space-group and crystal-structure determination. *Acta. Cryst. A* **2015**, *A71*, 3.

11. Sheldrick, G. M., Crystal structure refinement with SHELXL. *Acta. Cryst. C* **2015**, *C71*, 3.

12. Spek, A. L., PLATON SQUEEZE: a tool for the calculation of the disordered solvent contribution to the calculated structure factors. *Acta Cryst. C* **2015**, *C71*, 9.

13. A. L. Spek, **2005**, PLATON, A Multipurpose Crystallographic Tool, Utrecht University, Utrecht, The Netherlands.

14. Bourhis, L. J.; Dolomanov, O. V.; Gildea, R. J.; Howard, J. A.; Puschmann, H., The anatomy of a comprehensive constrained, restrained refinement program for the modern computing environment - Olex2 dissected. *Acta Cryst. A* **2015**, *A71*, 59.
